# Highly Reduced Plastid Genomes of the Non-photosynthetic Dictyochophyceans *Pteridomonas* spp. (Ochrophyta, SAR) Are Retained for tRNA-Glu-Based Organellar Heme Biosynthesis

**DOI:** 10.3389/fpls.2020.602455

**Published:** 2020-11-27

**Authors:** Motoki Kayama, Kacper Maciszewski, Akinori Yabuki, Hideaki Miyashita, Anna Karnkowska, Ryoma Kamikawa

**Affiliations:** ^1^Graduate School of Human and Environmental Studies, Kyoto University, Kyoto, Japan; ^2^Institute of Evolutionary Biology, Faculty of Biology, Biological and Chemical Research Centre, University of Warsaw, Warsaw, Poland; ^3^Japan Agency for Marine-Earth Science and Technology, Yokosuka, Japan; ^4^Graduate School of Agriculture, Kyoto University, Kyoto, Japan

**Keywords:** heme biosynthesis, Ochrophyta, plastid genome, reductive evolution, complex plastid

## Abstract

Organisms that have lost their photosynthetic capabilities are present in a variety of eukaryotic lineages, such as plants and disparate algal groups. Most of such non-photosynthetic eukaryotes still carry plastids, as these organelles retain essential biological functions. Most non-photosynthetic plastids possess genomes with varied protein-coding contents. Such remnant plastids are known to be present in the non-photosynthetic, bacteriovorous alga *Pteridomonas danica* (Dictyochophyceae, Ochrophyta), which, regardless of its obligatory heterotrophic lifestyle, has been reported to retain the typically plastid-encoded gene for ribulose-1,5-bisphosphate carboxylase/oxygenase (RuBisCO) large subunit (*rbcL*). The presence of *rbcL* without photosynthetic activity suggests that investigating the function of plastids in *Pteridomonas* spp. would likely bring unique insights into understanding the reductive evolution of plastids, their genomes, and plastid functions retained after the loss of photosynthesis. In this study, we demonstrate that two newly established strains of the non-photosynthetic genus *Pteridomonas* possess highly reduced plastid genomes lacking *rbcL* gene, in contrast to the previous report. Interestingly, we discovered that all plastid-encoded proteins in *Pteridomonas* spp. are involved only in housekeeping processes (e.g., transcription, translation and protein degradation), indicating that all metabolite synthesis pathways in their plastids are supported fully by nuclear genome-encoded proteins. Moreover, through an in-depth survey of the available transcriptomic data of another strain of the genus, we detected no candidate sequences for nuclear-encoded, plastid-directed Fe–S cluster assembly pathway proteins, suggesting complete loss of this pathway in the organelle, despite its widespread conservation in non-photosynthetic plastids. Instead, the transcriptome contains plastid-targeted components of heme biosynthesis, glycolysis, and pentose phosphate pathways. The retention of the plastid genomes in *Pteridomonas* spp. is not explained by the Suf-mediated constraint against loss of plastid genomes, previously proposed for Alveolates, as they lack Suf genes. Bearing all these findings in mind, we propose the hypothesis that plastid DNA is retained in *Pteridomonas* spp. for the purpose of providing glutamyl-tRNA, encoded by *trnE* gene, as a substrate for the heme biosynthesis pathway.

## Introduction

Plastids came into existence by endosymbiosis between a cyanobacterium and an ancient eukaryotic host that is currently believed to be the common ancestor of Archaeplastida. Plastids show the punctate distribution in various branches of the eukaryotic tree of life, as they have been laterally transferred through multiple independent secondary and/or higher order endosymbioses (Archibald, 2009; Keeling, 2010). These photosynthetic organisms are capable of efficiently converting solar energy to biochemical energy, which is subsequently utilized for various metabolic processes, such as carbon fixation, amino acid synthesis, nitrogen and sulfur metabolism, carotenoid synthesis, heme and chlorophyll synthesis, and a wide array of other metabolite synthesis pathways (Yasuno and Wada, 2002; Kleffmann et al., 2004; DellaPenna and Pogson, 2006; Hörtensteiner and Kräutler, 2011; Terashima et al., 2011; Przybyla-Toscano et al., 2018). An overwhelming part of the aforementioned pathways involves redox reactions, and these require proteins containing iron–sulfur (Fe–S) clusters as a cofactor for electron transport (Przybyla-Toscano et al., 2018).

Regardless of the beneficial aspects of photosynthesis as a mechanism to support autotrophic lifestyles by utilizing an easily accessible energy source, many organisms descending from photosynthetic ancestors have lost the ability of photosynthesis, therefore becoming obligate heterotrophs. Such secondarily non-photosynthetic organisms are known in land plants, green and red algae, ochrophytes, dinoflagellates and many other lineages; in fact, haptophytes, glaucophytes, and chlorarachniophytes are the only major plastid-bearing groups with no cases of photosynthesis loss known to date (Maciszewski and Karnkowska, 2019). Within photosynthetic lineages, photosynthesis losses may occur repeatedly, either in distantly related organisms or in close relatives. For example, all of the Apicomplexa (one of the main lineages of Alveolata) are descendants of a single ancestor that lost its photosynthetic capabilities, while in other lineages, such as diatoms, cryptophytes, or euglenids, losses of photosynthesis are documented to have occurred more than once in a single genus (Marin et al., 2003; Kamikawa et al., 2015; Salomaki and Kolisko, 2019).

With only a few exceptions, secondarily non-photosynthetic organisms still retain plastids, along with their genomes. As a prominent part of plastid genomes’ coding content constitutes photosynthesis-related genes, loss of photosynthesis is strictly tied with reductive evolution of plastid genomes, thus resulting in a varying degree of reduction in its size and gene repertoire. It is, however, worth noting that the patterns of gene loss and retention are extremely diverse across the tree of life. In many cases, non-photosynthetic plastids still possess genes for performing processes typically connected to photosynthesis, even though they forfeited the ability to absorb light. For example, certain secondarily non-photosynthetic lineages (e.g., the chlorophytes *Prototheca* spp. and the diatoms *Nitzschia* spp.) have retained the plastid-encoded ATP synthase complex genes (Kamikawa et al., 2015; Severgnini et al., 2018; Suzuki et al., 2018), while the gene for the large subunit of ribulose-1,5-bisphosphate carboxylase/oxygenase (RuBisCO) is present in non-photosynthetic plastids of *Cryptomonas paramecium* (Cryptophyta) and *Euglena longa* (Euglenophyta) (Donaher et al., 2009; Záhonová et al., 2016). In contrast, apicoplast genomes (plastid genomes of apicomplexans) carry no gene related to photosynthesis, but only the *sufB* gene, involved in Fe–S cluster assembly, and a complement of housekeeping genes, enabling transcription and translation of *sufB* (Salomaki and Kolisko, 2019).

There are, however, many secondarily non-photosynthetic organisms whose plastids’ functionality remains undescribed. One of these organisms is *Pteridomonas danica*, a non-photosynthetic, plastid-bearing bacteriovorous species of Dictyochophyceae (Ochrophyta). Knowledge on this organism’s plastid seems to be limited to its *rbcL* gene, retained in a similar manner to the aforementioned *C. paramecium and E. longa* (Sekiguchi et al., 2002), although in contrast to those two, no full plastid genome sequence from *P. danica*, or any other colorless dictyochophycean, has been reported so far. It is noteworthy that the phylogenetic analysis of *P. danica*’s *rbcL* demonstrated that its branch length was comparable to those of its photosynthetic relatives, suggesting that the gene’s evolutionary rate is not significantly increased. Therefore, it has not undergone remarkable degeneration, in contrast to photosynthesis-related genes in many non-photosynthetic organisms (Sekiguchi et al., 2002; Dorrell et al., 2019). Based on this observation, we hypothesized that the plastid genome of *Pteridomonas* might reflect the early stages of the reductive evolution of plastid genomes following the loss of photosynthesis.

Here, we report the complete plastid genomes of two strains of *Pteridomonas* spp. and show that in sharp contrast to the previous expectations, they are among the most functionally reduced plastid genomes reported to date. Our findings also demonstrate that even among very closely related species, the fates of genomes undergoing reduction can vary substantially through differential gene losses. Finally, we propose the most likely functional evolutionary constraint against complete loss of plastid genome after forfeiture of photosynthesis in *Pteridomonas*.

## Materials and Methods

### Isolation, Cultivation, and DNA Sequencing

A plankton net sample was collected at the port of JAMSTEC headquarters, Yokosuka, Kanagawa, Japan (35°19′09′′N, 139°39′02′′E) on January 9, 2013. A small aliquot of the net sample was initially added to the KLB medium (Yabuki and Tame, 2015) and incubated at 19–20°C under the 14-h light/10-h dark cycle condition. A single cell of *Pteridomonas* sp. was isolated from the enriched culture by micropipetting. The established strain YPF1301 was maintained under the same culture conditions and also deposited in the National Institute for the Environmental Sciences (NIES), Tsukuba, Japan as NIES-3357.

The other sample was collected at the Clover Point, Victoria, British Columbia, Canada (48°24′16″N, 123°21′02″W) on April 3, 2017, from the sand just beneath the water line in lower intertidal/upper subtidal layer. The established strain *P. danica* NY0221 was maintained using F/2 saltwater medium (prepared according to the guidelines from UTEX culture collection website), supplemented with vitamin B12 (to a total concentration of 100 μg l^–1^) and a single autoclaved barley seed (*Hordeum vulgare*). The strain was maintained in the same environmental conditions as strain YPF1301.

Using the DNeasy Plant mini kit (QIAGEN) following the manufacturer’s instruction, total DNA of *Pteridomonas* sp. YPF1301 was extracted from 1.32 × 10^7^ cells that were harvested from 15 flasks of 2-weeks-old 75 ml culture in total by 1,000 × *g* for 5 min. The procedure produced 50 μl of the DNA solution with 42.8 ng/μl concentration. The total DNA was sent to Hokkaido System Science Co., Ltd. (Japan) for 100 bp paired-end sequencing by the Illumina HiSeq2500 platform using the 350-bp library constructed with TruSeq Nano DNA Library Prep Kit (Illumina) following the manufacturer’s instructions. The sequencing produced 54.0 million reads. Adapter trimming and quality filtering were performed with FASTX-Toolkit^1^. Reads with quality scores > 20 for at least 75% of their length were retained after quality filtering, resulting in 49.4 million paired-end reads. The filtered short reads were subjected to SPAdes-3.10.0 (Bankevich et al., 2012) with default settings for assembling.

In case of *P. danica* NY0221, total DNA was isolated from cell pellets obtained by centrifugation of two 10 ml culturing tubes using the same kit as above, resulting in a 50 μl DNA sample with 23 ng/μl concentration. This sample was handed to a different external company (Genomed S.A., Warsaw, Poland) for high-throughput sequencing using Illumina MiSeq technology, producing six million paired-end, 300 bp long reads. Raw data quality control was performed using FastQC tool^2^; additional trimming was not necessary due to adapter removal and quality filtering being parts of data handling by the sequencing company. Genome assembly was performed identically as in case of the strain YPF1301.

### Reconstruction of Plastid DNA Sequences

Through homology-based search with plastid-encoded protein sequences of the dictyochophycean alga *Florenciella parvula* as queries, five AT-rich contigs highly likely derived from a plastid DNA was detected from the DNA assembly of *Pteridomonas* sp. YPF1301. By using PCR assays followed by the Sanger sequencing to fill the gap between termini of the contigs, we obtained a single DNA sequence of the plastid DNA with 54,809 bp in length for *Pteridomonas* sp. YPF1301. However, we could not map the genome as circular as no amplification occurred by PCR assays with multiple sets of primers bridging both termini of the single DNA sequence. Protein-coding genes were identified with MFannot^3^. The annotation was confirmed by BLASTP analyses of plastid-encoded protein sequences of *Pteridomonas* sp. YPF1301 against the GenBank non-redundant (nr) database. Transfer RNA genes were identified with MFannot^3^ and tRNAscan-SE (Lowe and Chan, 2016).

In contrast to YPF1301 strain, an analogous plastid-encoded protein homology-based search led to the identification of only one, 33,539 bp-long candidate plastid contig in the assembly of *P. danica* NY0221. A visualization of the contig using Bandage software (Wick et al., 2015) revealed the contig maps as circular, therefore most likely representing a full plastid genome molecule; additionally, attempts to elongate the putative plastid genome assembly using the same software tool failed, as it retrieved no additional contigs matching to any of the ends of the 33,539 bp-long contig. To further confirm that the circularly mapped sequence is the complete plastid genome, we also remapped the reads onto the plastid DNA with Bowtie2 (Langmead and Salzberg, 2012), resulting in >20× coverage observed consistently throughout the sequence, including the original start position. These procedures indicated that there was no additional plastid DNA contig to be assembled, and the plastid DNA of NY0221 was a circularly mapped molecule. Annotation of the contig was carried out using cpGAVAS online tool (Liu et al., 2012), followed by the Annotation Transfer feature available in Geneious v10.2.6 (Kearse et al., 2012), using the four available dictyochophycean plastid genomes (Han et al., 2019) as references, and manual curation.

### Phylogenetic Analysis of Plastid-Encoded Proteins

To see whether the plastid-encoded protein sequences of *Pteridomonas* spp. evolve as rapidly as those of the other non-photosynthetic Ochrophyta species (Kamikawa et al., 2015), we chose 38 proteins encoded in the plastid DNAs of both *Pteridomonas* sp. YPF1301 and *P. danica* NY0221. The same protein sequences were also retrieved from the previously sequenced plastid genomes of 44 species, including five non-photosynthetic species, in GenBank (Supplementary Material). Homologs of each protein were aligned by MAFFT (Katoh and Standley, 2013) with the L-INS-i option. Ambiguously aligned sites were removed manually by BioEdit (Hall, 1999). Datasets of each protein were concatenated, and the resulting dataset, composed of 44 taxa and 6,660 sites, was subjected to phylogenetic analysis using IQ-TREE 1.6.12 (Nguyen et al., 2015), under the LG + C60 + F + Γ-PMSF substitution model with 100 bootstrap analyses.

### Survey of Iron–Sulfur Cluster Proteins and Metabolic Pathways in the Plastid of *Pteridomonas danica* Strain PT

We obtained the assembled transcriptome data of *P. danica* strain PT from the Marine Microbial Eukaryote Transcriptome Sequencing Project (MMETSP; Keeling et al., 2014). Iron–sulfur cluster proteins in *P. danica* PT were surveyed with TBLASTN using queries of homologs from *Arabidopsis thaliana* (Couturier et al., 2013; Przybyla-Toscano et al., 2018) and diatoms (Kamikawa et al., 2017). Detected iron–sulfur cluster homologs were then confirmed by BLASTP against the non-redundant protein database of GenBank. Organellar targeting sequences of the detected homologs were evaluated with ASAFind (Gruber et al., 2015) and MitoFates (Fukasawa et al., 2015).

We also surveyed plastid-targeted protein sequences which require the iron–sulfur cluster as a cofactor, such as iron–sulfur domain-containing protein NEET, flavodoxin, chaperone protein dnaJ C76, translocon at the inner envelope membrane of chloroplasts 55, and ferredoxin, using homologs from *Arabidopsis thaliana* and photosynthetic stramenopiles available in NCBI database. The queries of photosynthetic stramenopiles were obtained from iron–sulfur cluster-containing proteins of *Arabidopsis thaliana* (Przybyla-Toscano et al., 2018) with TBLASTN search (Supplementary Table S1). Detected homologs from photosynthetic stramenopiles were confirmed and evaluated their plastid-targeting signals as described above. Sequences for the other plastid metabolisms (Bock and Khan, 2004; DellaPenna and Pogson, 2006; Hiltunen et al., 2012; Kamikawa et al., 2017; Przybyla-Toscano et al., 2018) were also surveyed.

### Detection of Nuclear-Encoded, Plastid-Targeted TufA

We performed TBLASTN search with *Florenciella parvula* plastid-encoded TufA protein sequence (GenBank accession number: YP_009684446.1) as a query against the DNA assembly of *Pteridomonas* sp. YPF1301. Through this procedure, we detected three contigs carrying genes for TufA. One of them was identified as mitochondrial TufA, as it possessed the N-terminal mitochondrial targeting sequence detected by MitoFates (Fukasawa et al., 2015). The others coded either the N-terminal or the C-terminal half of the TufA protein. Plastid targeting sequences were detected by ASAFind (Gruber et al., 2015) in both sequences. We also surveyed the mitochondria-targeted and plastid-targeted TufA sequences in the transcriptome data of *P. danica* PT by a homology-based search, with *Pteridomonas* sp. YPF1301 TufA protein sequences as queries. Mitochondrial targeting signals were detected by MitoFates (Fukasawa et al., 2015). Only the C-terminal half of TufA protein was detected in the transcriptome data of *P. danica* PT, and the sequence was revealed to have N-terminal plastid-targeting sequence detected by ASAFind (Gruber et al., 2015). Those sequences were aligned with plastid and mitochondria-targeting TufA proteins by MAFFT (Katoh and Standley, 2013). Ambiguously aligned sites were removed manually by BioEdit (Hall, 1999). The resulting dataset, comprising 147 taxa and 369 sites, was used for phylogenetic analysis using IQ-TREE 1.6.12 (Nguyen et al., 2015), under the LG + Γ + F substitution model with 100 bootstrap analyses.

### PCR-Based Survey and Phylogenetic Analysis of the *rbcL* Gene

Total DNA of *Pteridomonas* sp. strain YPF1301 was extracted as described above. Total DNA of *Phaeodactylum tricornutum* UTEX642 was extracted with Wizard Genomic DNA Purification Kit (Promega) according to the manufacturer’s instruction. We designed two primer sets exactly matched to the *rbcL* gene sequence of *P. danica* (Sekiguchi et al., 2002): rbcLF1/R1 and rbcLF2/R2 (Supplementary Table S2). We also designed a degenerated *rbcL* primer set (stramenopiles_RbcL_F/R; Supplementary Table S2) on the basis of RbcL protein sequences from ten Ochrophyta species (*Pedinella* sp. AB081639, *Pteridomonas danica* AB081642, *Pseudochattonella verruculosa* AB280607, *Synura borealis* HG514235, *Phaeodactylum tricornutum* MH064125, *Nitzschia palea* MH113811, *Florenciella parvula* MK518352, *Pseudopedinella elastica* MK518353, *Dictyocha speculum* MK561359, *Rhizochromulina marina* MK561360). The actin primer set was specifically designed for the actin gene sequence retrieved from the DNA assembly data of YPF1301 (Supplementary Table S2). Exact match primers were also designed for the plastid-encoded *rpl36* gene (Supplementary Table S2). For all the primer sets, PCR amplification was conducted for 30 cycles of: a denaturation step at 98°C for 10 s, an annealing step at 55°C for 30 s, and an elongation step at 72°C for 2 min.

The *rbcL* gene sequences of *Pteridomonas danica* (Sekiguchi et al., 2002) were aligned with available sequence data of Dictyochophyceae in the GenBank nucleotide database using MAFFT (Katoh and Standley, 2013). Ambiguously aligned sites were removed manually by BioEdit (Hall, 1999). The resulting dataset, comprising 24 taxa and 1,364 sites, was subjected to phylogenetic analysis using IQ-TREE 1.6.12 (Nguyen et al., 2015), under the GTR + Γ + I + F substitution model with 100 bootstrap replicates.

### Phylogenetic Analysis of 18S rRNA Gene

Nuclear 18S rRNA gene sequences of *Pteridomonas* sp. YPF1301 and *P. danica* NY0221 were retrieved from the DNA assembly data, and that of *P. danica* PT was retrieved from the transcriptome data. The sequences obtained were aligned with available sequence data of Dictyochophyceae in GenBank using MAFFT (Katoh and Standley, 2013). Ambiguously aligned sites were removed manually by BioEdit (Hall, 1999). The resulting dataset, comprising 42 taxa and 1,494 sites, was subjected to phylogenetic analysis using IQ-TREE 1.6.12 (Nguyen et al., 2015), under the GTR + Γ + I substitution model with 100 bootstrap replicates.

## Results and Discussion

### Plastid DNA in *Pteridomonas* spp.

**FIGURE 1:**
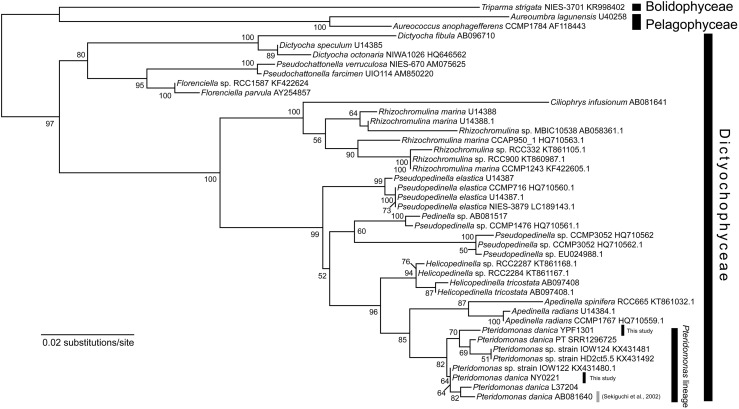
Maximum likelihood tree of 18S rRNA gene sequences of Dictyochophyceae. The tree was inferred with IQ-TREE 1.6.12 under the GTR + Γ + I model. Bootstrap values ≥50 are shown on branches. *P. danica* reported to bear *rbcL* is highlighted by gray line (Sekiguchi et al., 2002).

In the initial stage of this project, we have established two strains of *Pteridomonas* spp., strain YPF1301 and strain NY0221. Both strains are closely related to the *P. danica* investigated in the study presented by Sekiguchi et al. (2002), and to the *P. danica* strain PT, from which transcriptomic data has been obtained in the Marine Microbial Eukaryote Transcriptome Sequencing Project (Keeling et al., 2014) (Figure 1).

**FIGURE 2:**
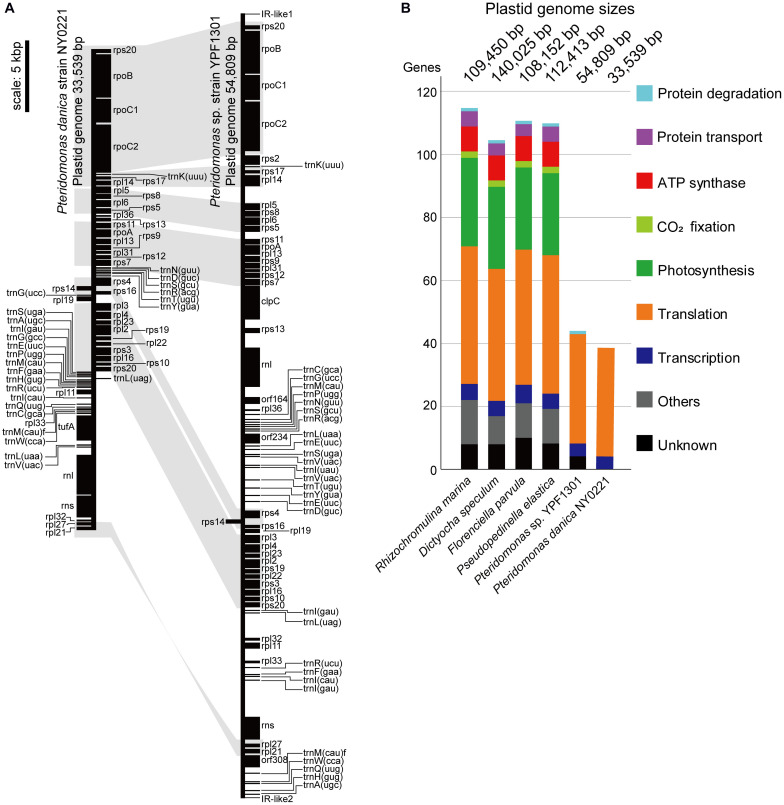
Plastid genomes of *Pteridomonas* sp. strain YPF1301 and *Pteridomonas danica* strain NY0221. **(A)** Plastid genome maps. The genomes are shown as linear for comparison of gene locations although the genome of strain NY0221 is a circularly mapping molecule (Supplementary Figure S1). Protein-coding and ribosomal RNA-coding regions are shown as black boxes. Transfer RNAs are shown as black lines. Conserved gene orders are highlighted in light gray. **(B)** Comparison of plastid-encoded genes and genome sizes among the photosynthetic and non-photosynthetic species of Dictyochophyceae (Han et al., 2019). Any color boxes show functional categories: light blue for protein degradation (*clpC*), violet for transport (*sec*, *tatC*), red for ATP synthase (*atp*), light green for CO_2_ fixation (*rbcL*), green for photosynthesis (*psa*, *psb*, *pet*), orange for translation (*rps*, *rpl*, *tufA*), blue for transcription (*rpo*), gray for other known functions, black for unknown functions (orf, *ycf*).

First, we sequenced the plastid DNA of the non-photosynthetic dictyochophyceaen *Pteridomonas* sp. strain YPF1301. The homology-based search of plastid-encoded proteins against the assembly of *Pteridomonas* sp. YPF1301, followed by PCR and the Sanger sequencing, resulted in a single, 54,809 bp-long, DNA sequence representing the plastid genome (Supplementary Figure S1). Regardless of numerous trials of PCR assay with multiple primer sets, we could not close the gap between termini of the plastid DNA sequence (data not shown). Instead, we detected a short, non-coding inverted repeat at both ends of the plastid genome sequence (Figure 2 and Supplementary Figure S1), similar to the ones reported from other linear-mapping organellar genomes (Nosek et al., 2004; Janouškovec et al., 2013; Oborník and Lukeš, 2015; Salomaki et al., 2015; Kamikawa et al., 2018). As no other contig has been detected as a candidate plastid genome fragment, we conclude that the 55 kb-long DNA covers the almost complete plastid genome of *Pteridomonas* sp. YPF1301. It remains unresolved whether the plastid DNA of *Pteridomonas* sp. YPF1301 is a linear molecule, or a circular molecule with an extended non-coding region, non-detectable by the homology-based methods. Both possibilities can equally well explain failures of amplification by our multiple PCR trials (data not shown). The plastid genome of *Pteridomonas* sp. YPF1301 carries 40 protein-coding genes, 24 transfer RNA genes, and two rRNA genes, as well as three functionally unassigned open reading frames (ORFs; Figure 2 and Table 1). The coding regions occupy 57.8% of the genome, excluding unassigned ORFs (62.3%, including those regions as coding ones) (Table 1). More than one-third of the genome is likely non-coding. The plastid genome is devoid of the tetrapartite structure, composed of a small single-copy region, a large single-copy region, and two inverted repeat copies of an rRNA operon, commonly observed in photosynthetic and non-photosynthetic plastid genomes of Ochrophyta (Han et al., 2019).

**TABLE 1:** Plastid genomes of *Pteridomonas* spp.

Strains		*Pteridomonas* sp. YPF1301	*Pteridomonas danica* NY0221
Lengths (bp)		54809	33539
Coding regions (%)		57.76	90.26
Protein genes^a^		40	39
A + T content (%)		79.63	75.24
Proteins	Translation	35	35
	Transcription	4	4
	Proteinase	1	–
	Unknown ORFs	3	–
RNAs	rRNA	2	2
	tRNA^b^	26	26

*^a^Orfs functionally unassigned were not counted. ^b^Duplicated genes were counted only once. –, not detected.*

Subsequently, we sequenced the total DNA of *Pteridomonas danica* strain NY0221, and in contrast to YPF1301, we assembled a 33,539 bp-long circularly mapping molecule representing the plastid genome (Supplementary Figure S1). The plastid genome of *P. danica* NY0221 carries 39 protein-coding genes, 24 tRNA genes, and two rRNA genes (Table 1), and also lacks inverted repeats of the rRNA operon. The non-coding regions occupy less than 10% of this genome, in contrast with the expanded non-coding regions in the plastid genome of strain YPF1301. The gene-dense plastid genome of NY0221 strain is consistent with the previous observations of gene-dense plastid genomes of other non-photosynthetic members of Ochrophyta, usually with more than 80% of coding regions per genome (Kamikawa et al., 2018; Dorrell et al., 2019). Thus, the plastid genome of strain YPF1301, with less than 62% of coding regions, is an exception among those of the non-photosynthetic members of Ochrophyta.

The striking difference in size and gene density between the two plastid genomes of closely related non-photosynthetic *Pteridomonas* species might represent distinct fate or distinct evolutionary rate of plastid genome erosion after the abandonment of photosynthesis in their last common ancestor.

### Recent Genome Rearrangements

Both genomes contain a similar gene set: the 40 proteins encoded in the YPF1301 plastid genome comprise 35 proteins for translation (i.e., ribosomal proteins), four proteins for transcription (i.e., RNA polymerases), and one gene for protein degradation (i.e., ClpC subunit), the latter missing from the plastid genome of strain NY0221. Apart from *clpC*, the plastid genome of the strain NY0221 also lacks two translation-related genes (*rps2, rpl32*), retained in strain YPF1301, but contains the translation elongation factor Tu gene (*tufA*), which is missing in strain YPF1301. Regardless of the previous report for *rbcL* gene in *P. danica* (Sekiguchi et al., 2002), we obtained no evidence of the corresponding gene sequence in the plastid genomes of our newly established strains (Figures 2A,B).

Interestingly, despite the differences in coding contents and size, the order of protein-coding genes in plastid genomes of *Pteridomonas* spp. is almost identical, with only a single, two protein-coding gene block (*rpl33, rpl11*) having inverse orientation. These genomes, however, are not fully syntenic, as the order of tRNA-encoding genes is drastically different (Figure 2 and Supplementary Figure S2). It would suggest that numerous rearrangements have taken place in their evolutionary past, but in contrast to other dictyochophyceans, they do not seem to affect the protein-coding gene order to a noticeable extent (Han et al., 2019). Although both plastid genomes presented in this work carry a single copy of the ribosomal RNAs, its organization differs substantially between the investigated strains. The small (*rns*) and large (*rnl*) ribosomal RNA subunit genes are clustered together in the same orientation in *P. danica* NY0221, which is a rather common feature in plastid genomes, as it has also been observed in another dictyochophyte, *Dictyocha speculum* (Han et al., 2019), as well as in certain diatoms (Cao et al., 2016), euglenids (Karnkowska et al., 2018) and chlorophytes (Turmel et al., 2017). In *Pteridomonas* sp. YPF1301, however, the ribosomal RNA small and large subunit genes are present in inverse orientation and separated by a cluster of 18 protein-coding genes, constituting almost half of the entire plastid genome; such a “broken” ribosomal operon has been identified so far exclusively in the plastid genome of the non-photosynthetic, parasitic green alga *Helicosporidium* sp. (de Koning and Keeling, 2006). The difference in rDNA organization between the examined strains of *Pteridomonas* can be interpreted as yet another indicator of the divergent evolution of plastid genomes in these two closely related organisms.

### Extraordinarily Functionally Reduced Plastid DNAs of *Pteridomonas* spp.

**FIGURE 3:**
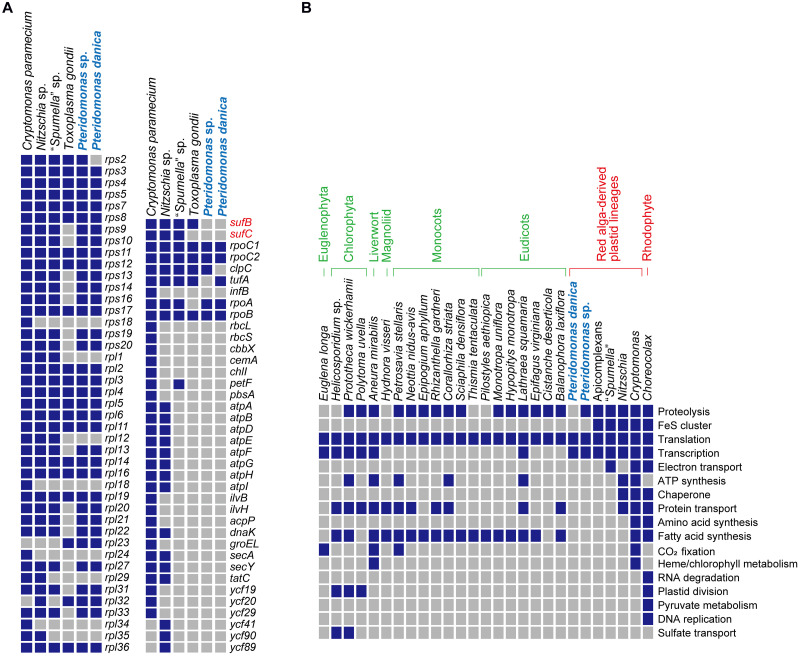
Comparison of non-photosynthetic plastid genomes. **(A)** Comparison of plastid gene contents from non-photosynthetic organisms with red alga-derived plastid. *Nitzschia* sp. NIES-3581 (Bacillariophyceae; GenBank no. AP018508; Kamikawa et al., 2015), *Cryptomonas paramecium* (Cryptophyta; GenBank no. GQ358203; Donaher et al., 2009), *Toxoplasma gondii* (Apicomplexa; GenBank no. U87145; Köhler et al., 1997), “*Spumella*” sp. NIES-1846 (Chrysophyceae; GenBank no. AP019363; Dorrell et al., 2019) were used for comparison. Presence and absence are shown by blue and gray boxes, respectively. **(B)** Comparison of the estimated functions encoded in non-photosynthetic plastid genomes. All the data except for *Pteridomonas* spp. highlighted in light blue are derived from Hadariová et al. (2018) and Dorrell et al. (2019).

When compared to other species of Dictyochophyceae (Han et al., 2019), the plastid genomes of *Pteridomonas* spp. are the smallest in size and gene content (Figure 2B). So far, plastid genomes of four photosynthetic Dictyochophyceae have been sequenced, and their sizes range from 108 to 140 kb, which are two or more times larger than the two *Pteridomonas* spp. plastid genomes analyzed here (Figure 2B). The photosynthetic plastid genomes of Dictyochophyceae sequenced to date carry 37 to 42 genes encoding proteins involved in photosynthesis, carbon fixation, and chlorophyll synthesis, 4 to 5 genes for protein transport, 42 to 44 genes for translation, 5 to 6 genes for transcription, and one gene for protein degradation. In contrast, functions encoded in the *Pteridomonas* spp. plastid genomes are dramatically reduced: all genes for photosynthesis, carbon fixation, chlorophyll synthesis, ATP synthesis/degradation, chaperonins (*groEL*), and Fe–S cluster assembly (*sufB* and *sufC*; Figure 2) are lost, and only genes for translation and transcription (and protein degradation in YPF1301) are retained. Moreover, their sequences are highly divergent and form long-branches on the phylogenetic tree, comparable to branches of other non-photosynthetic species of Ochrophyta (Supplementary Figure S3). The *Pteridomonas* plastid genomes encode fewer functions than other plastid genomes of non-photosynthetic species with red alga-derived plastids, including apicomplexan parasites and non-photosynthetic red algae (Figure 3A). Even among non-photosynthetic species, including green algal lineages and plants (Figure 3B), *Pteridomonas* plastid genomes are one of the most functionally reduced ones.

The most unexpected results are the lack of *rbcL, sufB*, and *sufC* genes in both strains, and the punctate distribution of *tufA* and *clpC* genes in *Pteridomonas* spp. genomes. First, we reveal the punctate distribution of *tufA* gene in the plastid genomes of *Pteridomonas* spp., which is explained by the recent endosymbiotic gene transfer. Second, the strain of *P. danica* reported by Sekiguchi et al., the close relative of *Pteridomonas* spp. YPF1301 and NY0221 (Figure 1), retained the *rbcL* gene (Sekiguchi et al., 2002). Lastly, *sufB* and *sufC* genes are highly conserved among plastid genomes of photosynthetic Dictyochophyceae, and also non-photosynthetic, red alga-derived plastid-bearing species, with only a few exceptions (Janouškovec et al., 2015; Dorrell et al., 2019; Han et al., 2019; see also Figure 3). We discuss the above issues in more details below.

### Recent Endosymbiotic Gene Transfer

Punctate distribution of *clpC* and *tufA* might be a result of gene loss or endosymbiotic gene transfer. We did not detect any candidate sequence of the *clpC* gene in the assembly of strain NY0221. The gene for ClpC has been lost in NY0221 after divergence from the lineage leading to YPF1301. However, we retrieved two GC-rich contigs carrying *tufA* gene fragments, one of which contains a spliceosomal intron, suggesting that the *tufA* gene is most likely encoded in the nuclear genome of YPF1301 strain. Those results imply a recent plastid-to-nucleus endosymbiotic gene transfer of the *tufA* gene in *Pteridomonas* sp. YPF1301. One of the two fragments encodes N-terminal half of the plastid TufA protein, while the other codes its C-terminal half. It would be worth noting that both of the two *tufA* gene fragments encode bipartite plastid-targeting signals, comprising signal peptides followed by transit peptide-like regions (Supplementary Figures S4A,B), targeting the nuclear-encoded plastid proteins to the organelle (Gruber et al., 2015). Presence of the targeting signal in both sequences suggests that *tufA* is encoded in two distinct loci and imported into the plastid as two peptides. The phylogenetic analysis of organellar TufA proteins recovered the plastid-encoded sequence of *P. danica* NY0221 and the nuclear-encoded N-terminal half sequences of *Pteridomonas* sp. YPF1301 as monophyletic although its bootstrap support was moderate (64%; Supplementary Figure S4C). The monophyletic relationship would suggest a recent endosymbiotic gene transfer to the nucleus. Similarly, the nuclear-encoded C-terminal half sequences of *P. danica* strain PT and *Pteridomonas* sp. YPF1301 formed a monophyletic group (bootstrap value = 61%), and the clade was then nested in Ochrophyta, although the position of *Pteridomonas* sequences was not strongly or even moderately supported. Nevertheless, the lower supports on branches in the tree are most likely due to insufficient phylogenetic signals in the single protein dataset used for the analysis. This finding might represent a possible example that reduced non-photosynthetic plastid genomes are shaped not only by gene losses but also gene flow from the organelle to the nucleus, still ongoing even after photosynthesis loss.

### Loss of the Canonical *rbcL* Gene

To investigate whether *rbcL* gene is present in the nuclear genome, it was further surveyed by homology-based search, with the previously reported *rbcL* sequence of *P. danica* (GenBank accession number: AB081642.1) as a query against the genome assembly of YPF1301, but this procedure did not detect any candidate sequence of *rbcL*. Similar results were also obtained in the PCR assays. We designed multiple primer sets, including exact match primers to *P. danica rbcL* and degenerate primers for the conserved regions of RuBisCO large subunit (RbcL; Supplementary Table S2). The PCR assays with these primer sets did not amplify any product for the *Pteridomonas* sp. YPF1301 DNA (Supplementary Figure S5). These experiments show that *rbcL* gene is most likely absent in the nuclear genome of *Pteridomonas* sp. YPF1301 or its sequence is too divergent to be detectable. The *rbcS* gene for RuBisCO small subunit was also undetectable by the homology-based survey. Similarly, by the homology-based survey, the *rbcL* and *rbcS* genes were undetected from the assembled DNA data of *P. danica* NY0221. These findings might raise a possibility that *rbcL* genes of *P. danica* and another non-photosynthetic dictyochophycean species might not be simply derived from a vertical inheritance from photosynthetic ancestors but of certain artifact such as a contamination. Otherwise, these findings might represent ongoing strain-specific *rbcL* gene losses in strain YPF1301, and most likely also in the strain NY0221.

**FIGURE 4:**
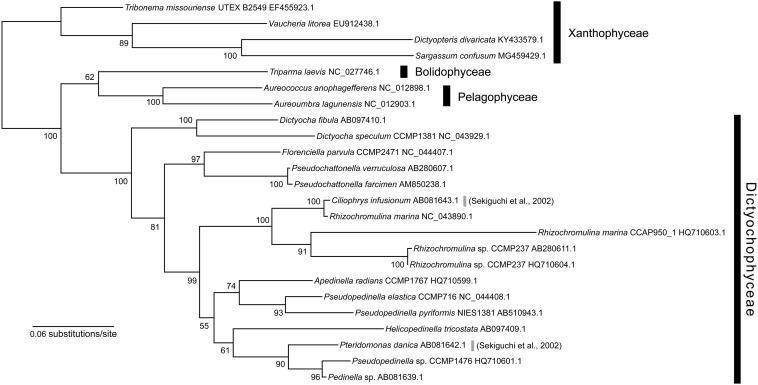
Maximum likelihood tree of rbcL gene sequences of Dictyochophyceae. The tree was inferred with IQ-TREE 1.6.12 under the GTR + Γ + I + F model. Bootstrap values ≥50 are shown on branches.

To evaluate these possibilities, we performed phylogenetic analysis of the *rbcL* gene, with a richer taxon sampling than that of Sekiguchi et al. (2002) (Figure 4). Consequently, the *rbcL* gene of *P. danica* (Sekiguchi et al., 2002) formed a monophyletic clade with those of the photosynthetic dictyochophycean species *Pseudopedinella* sp. CCMP1476 and *Pedinella* sp. (90% bootstrap value; Figure 4), inconsistent with the sister relationship between *Pteridomonas* and *Apedinella* in the 18S rRNA tree (Figure 1). Similarly, the *rbcL* gene of the non-photosynthetic dictyochophycean *Ciliophrys infusionum* nested in the *Rhizochromulina* clade (Figure 4; 100% bootstrap value), and specifically, formed a monophyletic clade with *Rhizochromulina marina* (100% bootstrap value; Figure 4). Given the inconsistency between the organismal (18S rRNA) phylogeny and the *rbcL* phylogeny, we suggest the *rbcL* genes in *P. danica* as well as *C. infusionum* might not be derived from a simple vertical inheritance from their photosynthetic ancestors. As it cannot be concluded whether they are derived of contaminations, possible strain-specific losses of *rbcL* genes in *Pteridomonas* spp. should be investigated after isolation of new strains closely related to the previously reported *rbcL*-bearing *P. danica* (Sekiguchi et al., 2002).

### Loss of Genes for Iron–Sulfur Cluster Assembly

**FIGURE 5:**
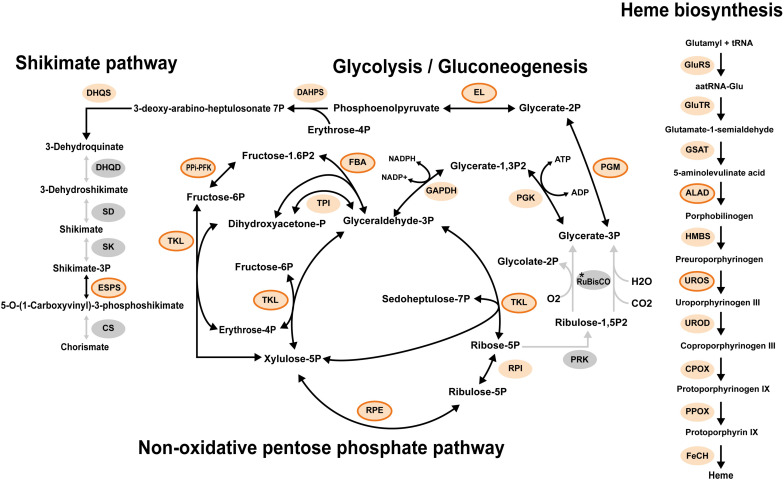
Representative metabolisms of the plastid in *Pteridomonas danica* strain PT. Light orange circles enclosed by an orange line show proteins with the detectable plastid targeting signals. Light orange circles with no line show proteins with no detectable plastid targeting signal probably due to lack of 5′ termini of sequences in the transcriptome data. Gray circles show proteins not detected. The asterisk shows that it is unclear whether RuBisCO is present as both large and small subunits of RuBisCO are plastid-encoded proteins; A plastid genome of strain PT is unavailable. ALAD, delta-aminolevulinic acid dehydratase; CPOX, coproporphyrinogen oxidase; CS, chorismate synthase; DAHPS, 3-deoxy-7-phosphoheptulonate synthase; DHQD, bifunctional 3-dehydroquinate dehydratase; DHQS, 3-dehydroquinate synthase; EL, enolase; ESPS, 3-phosphoshikimate 1-carboxyvinyltransferase; FBA, fructose 1,6-bisphosphate aldolase; FeCH, ferrochelatase; GAPDH, glyceraldehyde 3-phosphate dehydrogenase; GluRS, glutamyl tRNA synthase; GluTR, glutamyl tRNA reductase; GSAT, glutamate-1-semialdehyde aminotransferase; HMBS, hydroxymethylbilane synthase; PGK, phosphoglycerate kinase; PGM, phosphoglycerate mutase; PPi-PFK, pyrophosphate-dependent phosphofructokinase; PPOX, protoporphyrinogen oxidase; PRK, phosphoribulokinase; RuBisCO, ribulose-1,5-bisphosphate carboxylase/oxygenase large and small subunits; RPE, ribulose 5-phosphate 3-epimerase; RPI, ribose 5-phosphate isomerase; SD, shikimate dehydrogenase; SK, shikimate kinase; TAL, transaldolase; TKL, transketolase; TPI, triosephosphate isomerase; UROD, uroporphyrinogen decarboxylase; UROS, uroporphyrinogen III synthase.

Fe–S cluster assembly involves plastid-encoded proteins SufB and SufC, as well as nuclear-encoded SufA, SufD, SufE, and SufS (Lill, 2009; Balk and Schaedler, 2014; Przybyla-Toscano et al., 2018). Given the absence of *sufB* and *sufC* in the plastid contig, we searched the contigs representing nuclear genome for genes encoding Suf system proteins, using homologs from *Arabidopsis thaliana* (Przybyla-Toscano et al., 2018) and homologs of photosynthetic Ochrophyta species as queries. Using this procedure, we did not detect any Suf family homologs in the assembly of *Pteridomonas* sp. YPF1301 or in the MMETSP-originated transcriptome of *P. danica* strain PT (Keeling et al., 2014) (Figure 5). In contrast, genes encoding mitochondrial and cytosolic Fe–S cluster assembly systems were present in the PT transcriptome data (Lill, 2009; Balk and Schaedler, 2014) (Supplementary Figure S6 and Supplementary Table S3). Therefore, we conclude that the Fe–S cluster assembly Suf system is most likely absent from the plastids of *Pteridomonas* spp.

The Fe–S cluster proteins are essential for various metabolic pathways in plastids, such as photosynthesis, amino acid synthesis, nitrogen and sulfur metabolism, carotenoid synthesis, vitamin and thiazole synthesis, chlorophyll synthesis and degradation, or lipoic acid synthesis (Przybyla-Toscano et al., 2018). Therefore, Suf pathway components (*sufB* and *sufC*) are among the most widespread and conserved plastid-encoded proteins. In alveolates, plastid *sufB* gene is proposed to play a crucial role as the constraint against loss of plastid DNA after loss of photosynthesis (Janouškovec et al., 2015, 2019).

To gain insight into the evolution of the Suf system in the non-photosynthetic dictyochophycean *Pteridomonas* spp., we further surveyed the transcriptome of *P. danica* strain PT for plastid-targeted proteins requiring Fe–S clusters (Supplementary Table S1). Intriguingly, we did not identify any of those sequences (Supplementary Figure S7), as none of the pathways reconstructed in the plastid metabolic map of *P. danica* PT, such as chorismate synthesis, heme synthesis, glycolysis, and the non-oxidative pentose phosphate pathway, require Fe–S clusters (Figure 5, Supplementary Table S4, and Supplementary Figure S7). Given these observations, it is most likely that *Pteridomonas* spp. not only lost the plastid Suf system for Fe–S cluster assembly, but also forfeited all plastid pathways requiring Fe–S clusters as a cofactor after the loss of photosynthesis. The functional reduction of plastids in the bacteriovorous *Pteridomonas* spp. is consistent with a previously proposed hypothesis that parasitic and phagotrophic species could achieve a deep functional reduction of plastids after loss of photosynthesis, while osmotrophic species would maintain many plastidial biosynthetic functions (Kamikawa et al., 2017; Dorrell et al., 2019).

### Possible Roles of the *Pteridomonas* Plastid Genome

Our comparative plastid genome analyses provide insight into the evolutionary principle of organellar genome retention even after the loss of photosynthesis. Interestingly, although we sequenced the plastid genome of two non-photosynthetic species of Dictyochophyceae, *Pteridomonas* sp. YPF1301 and *P. danica* NY0221, the only genes they share encode the proteins involved in gene expression (Figure 2).

Based on the observation that *sufB* gene is conserved in almost all non-photosynthetic plastid genomes in Alveolata, it was proposed that this gene would be a possible constraint against loss of plastid genome in non-photosynthetic algae (Janouškovec et al., 2015). Our findings of *Pteridomonas* spp. plastid genomes lacking any genes for the plastid Suf system indicate that *sufB* gene is not always a constraint against loss of plastid DNA. Therefore, which factors might make the plastid genome indispensable in? The strain YPF1301 carries a plastid-encoded *clpC* gene, which was proposed as a possible constraint against loss of the apicoplast genome in the non-photosynthetic apicomplexan parasite *parva* (Janouškovec et al., 2015, 2019). *T. parva* also retains the *clpC* gene but it lacks *sufB* gene and any other genes involved in plastid metabolism (Janouškovec et al., 2015, 2019). Thus, the *clpC* gene might be one of factors mediating the retention of plastid DNA in *Pteridomonas* sp. YPF1301, possibly for correct assembly of the organellar multisubunit complex (Choquet and Vallon, 2000; Wostrikoff et al., 2004). However, this does not apply to *P. danica* strain NY0221, as it lacks the *clpC* gene, as well as any other non-housekeeping protein-coding genes.

For *Pteridomonas* spp., we propose that the plastid-encoded *trnE* gene is the most likely constraint against loss of plastid DNA (Barbrook et al., 2006; Hadariová et al., 2018). The plastid heme biosynthesis pathway begins with tRNA-Glu, synthesized from a *trnE* gene transcript and glutamate by tRNA-Glu synthase (Barbrook et al., 2006). This role of *trnE* is the only gene expression-unrelated function encoded in the entire plastid genome of *P. danica* strain NY0221. As *trnE* gene is conserved in all of the known non-photosynthetic plastid genomes (Barbrook et al., 2006; Wicke et al., 2016; Hadariová et al., 2018), the *trnE*-mediated constraint would be typical for all non-photosynthetic plastid genomes except apicoplasts. Genes for transcription (i.e., *rpoA*, *rpoB*, *rpoC1* and *rpoC2* genes) and for translation (i.e., *tufA*, *rps*, *rpl*, rRNA, and tRNA genes) could be basically retained to transcribe *trnE* gene in *Pteridomonas* sp. NY0221. It is suggested that some non-photosynthetic land plants need to import certain tRNA species from another compartment because they lost the corresponding tRNA genes from the plastid DNA (Barbrook et al., 2006; Wicke et al., 2016). In fact, some plastid-bearing non-photosynthetic algae and land plants completely lack plastid genomes, but retain tRNA-Glu-dependent heme biosynthesis in the plastids, suggesting import of tRNA-Glu into their plastids (e.g., Dorrell et al., 2019). After non-photosynthetic plastids establish efficient import of tRNAs, tRNA genes including *trnE* might become unnecessary to be present in and transcribed from the plastid DNAs, which may be followed by loss of plastid genomes, as seen in a few plastid genome-lacking algae/land plants (e.g., Dorrell et al., 2019).

The reason behind the apicoplasts being exceptional would be that the heme biosynthetic pathway in apicomplexan parasites begins in mitochondria, and it involves synthesis of the precursor of heme from glycine and succinate, but not from tRNA-Glu (Oborník and Green, 2005).

## Conclusion

The data presented in this work can serve as an important insight into the scarcely studied phenomenon of loss of photosynthetic function in plastids of free-living organisms, as opposed to the considerably well-studied remnant plastids of parasites. We demonstrate that *Pteridomonas* spp. possess highly functionally reduced plastid genomes, lacking *sufB* and *sufC* genes, the former of which has been thought to have a role as the possible constraint against the loss of plastid genome. Instead, we propose that the main reason for the retention of the plastid genome in *Pteridomonas* is the tRNA-Glu gene (*trnE*), essential for the plastid heme biosynthesis pathway.

We also observed that *Pteridomonas* spp., despite their close kinship, exhibit a divergent pattern of gene loss and retention in their non-photosynthetic plastid genomes. This pattern, however, is not at all unique in secondarily non-photosynthetic algae and land plants, as it has been described in other groups – e.g., the parasitic land plants Orobanchaceae (Wicke et al., 2013), the cryptophyte genus *Cryptomonas* (Tanifuji et al., 2020), the diatom genus *Nitzschia* (Kamikawa et al., 2018) and the chlorophyte genus *Prototheca* (Figueroa-Martinez et al., 2015; Severgnini et al., 2018; Suzuki et al., 2018). In each of these groups except for Orobanchaceae, loss of photosynthesis occurred more than once, leading to diverse outcomes in relatively closely related organisms, although the case of *Nitzschia* is still controversial (Onyshchenko et al., 2019).

The findings from our study suggest that the existence of universal constraints against genome loss in non-photosynthetic plastids is unlikely. Instead, we are convinced that the fate of the organellar genome and the conservation of its functions vary with each lineage’s evolutionary background, ecological context, and a wide array of unpredictable occurrences. As a result, even in very close relatives, the vestigial plastids can be far from identical.

It is, however, important to note that these observations are based solely on our investigation of organellar genomes and transcriptomic data of a related strain, and do not fully account for the nuclear-encoded components of plastids’ functionality, which still await a detailed examination in a separate study. Additionally, the importance of *trnE* for plastid genome retention must be further evaluated by future biochemical studies on the acquisition of tRNA-Glu for heme biosynthesis in completely DNA-deprived vestigial plastids (Oborník and Green, 2005; Barbrook et al., 2006; Wicke et al., 2016; Hadariová et al., 2018).

## Data Availability Statement

Plastid genome sequences of *Pteridomonas* spp. were deposited to data banks under the following accession numbers (DDBJ LC580440 for *Pteridomonas* sp. YPF1301 and GenBank MT909785 for the *Pteridomonas danica* NY0221). The raw reads of YPF1301 were also deposited to DDBJ (PRJDB10481). The datasets used for the phylogenetic analyses can be found in the Supplementary Material.

## Author Contributions

RK and AK conceived the project. MK, KM, and AY sequenced, assembled, and annotated plastid genomes. MK analyzed the data and performed phylogenetic analyses. AK, HM, and RK provided reagents and materials. MK, KM, AK, and RK wrote the manuscript. All the authors checked and approved the final version.

## Conflict of Interest

The authors declare that the research was conducted in the absence of any commercial or financial relationships that could be construed as a potential conflict of interest.

## Footnotes

1 http://hannonlab.cshl.edu/fastx_toolkit/

2 http://www.bioinformatics.babraham.ac.uk/projects/fastqc/

3 https://megasun.bch.umontreal.ca/cgi-bin/dev_mfa/mfannotInterface.pl
