# Supplementary material for: Highly Reduced Plastid Genomes of the Non-photosynthetic Dictyochophyceans Pteridomonas spp. (Ochrophyta, SAR) Are Retained for tRNA-Glu-Based Organellar Heme Biosynthesis
Source: Front Plant Sci. 2020 Nov 27;11:602455. doi: 10.3389/fpls.2020.602455 (PMC7728698; doi:10.3389/fpls.2020.602455)
Supplement: Supplementary file 3 [file Data_Sheet_3.PDF]

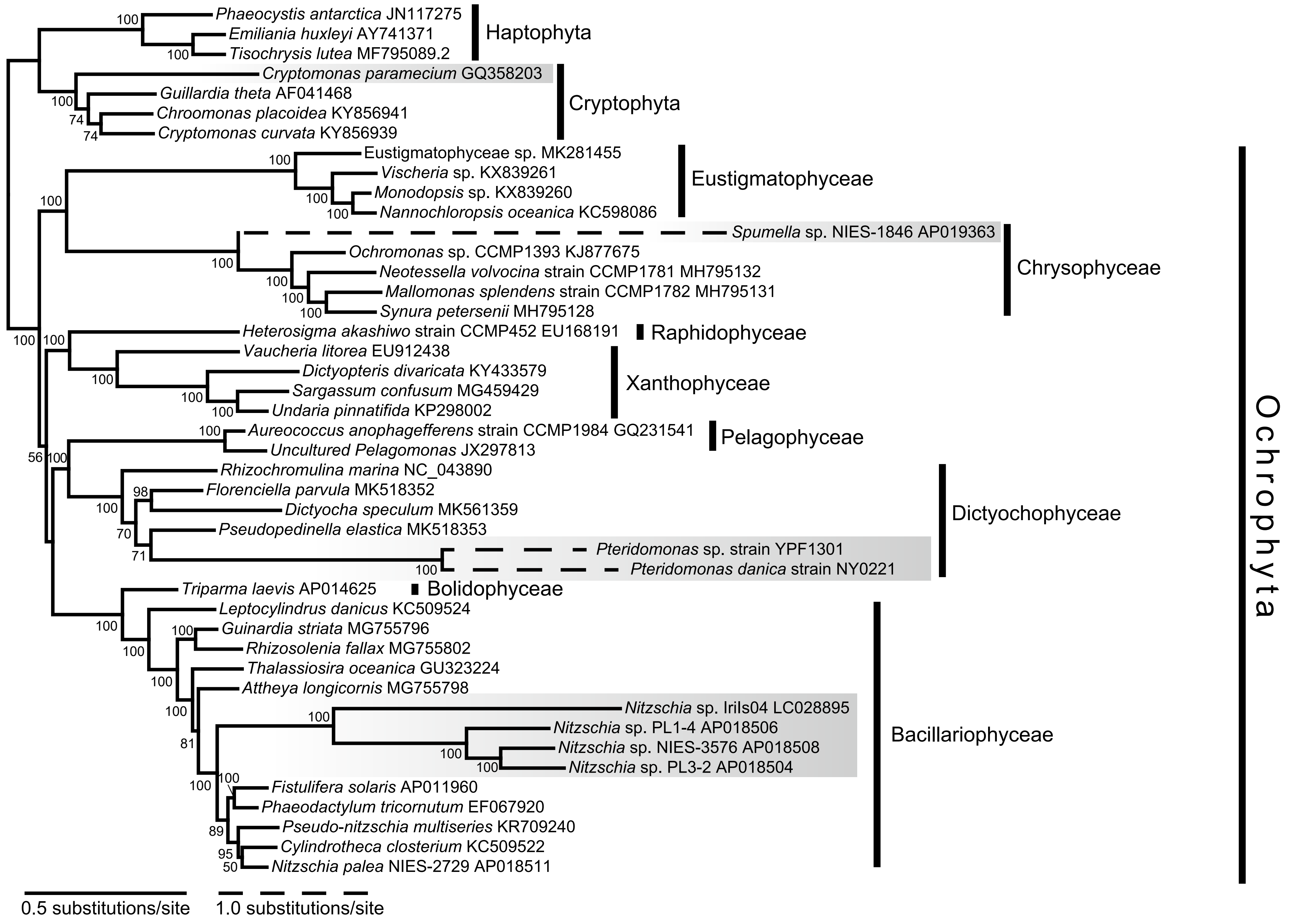

Supplementary Figure 3. Maximum likelihood tree inferred from the 38 plastid protein data set. The 38-protein data set comprised of 44 taxa and 6,660 sites was analyzed with IQ-tree 1.6.12 under LG+C60+F+Γ-PMSF model. Bootstrap values ≥50 are shown on branches. Non-photosynthetic diatoms and *Cryptomonas paramecium*, *Pteridomonas* spp., "*Spumella*" sp. are highlighted in grey.
