# Supplementary material for: Highly Reduced Plastid Genomes of the Non-photosynthetic Dictyochophyceans Pteridomonas spp. (Ochrophyta, SAR) Are Retained for tRNA-Glu-Based Organellar Heme Biosynthesis
Source: Front Plant Sci. 2020 Nov 27;11:602455. doi: 10.3389/fpls.2020.602455 (PMC7728698; doi:10.3389/fpls.2020.602455)
Supplement: Supplementary file 4 [file Data_Sheet_4.PDF]

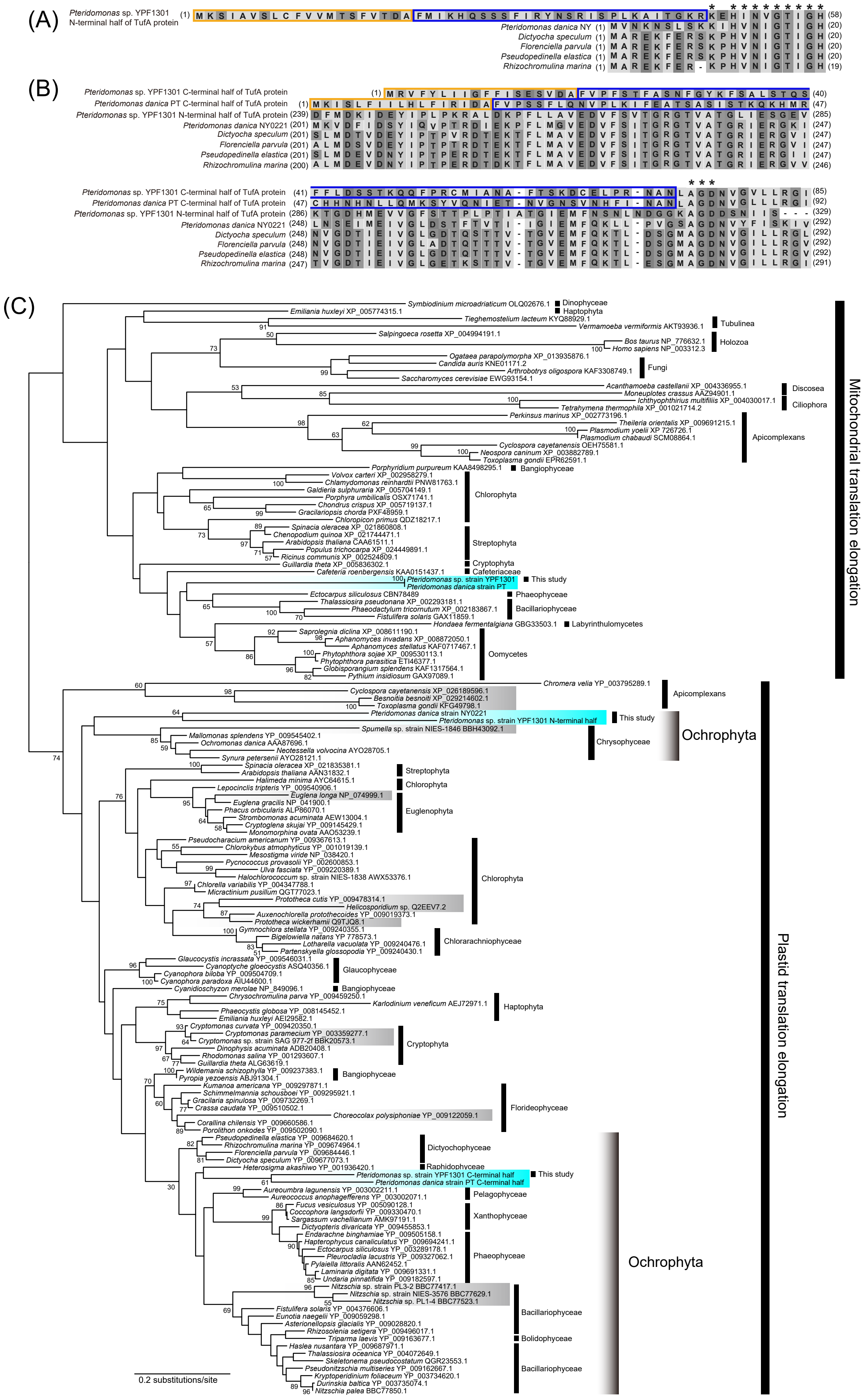

Supplementary Figure 4. TufA phylogeny and *tufA* gene organization. A. Comparison of N-terminal sequences of plastid-encoded dictyochophycean TufA sequences and plastid-targeted N-terminal half of TufA in *Pteridomonas* sp. YPF1301. A signal peptide region is enclosed by an orange line, while a transit peptide-like region is enclosed by a dark blue line. Numbers in parentheses show amino acid numbers of sequences used herein. B. Comparison of dictyochophycean plastid-encoded TufA sequences and plastid-targeted TufA of *Pteridomonas* spp. Other details are mentioned in A. C. Maximum likelihood tree of organellar TufA. The TufA dataset comprised of 147 taxa and 369 sites was analyzed with IQ-tree 1.6.12 under the LG+F+Γmodel. Bootstrap values ≥50 are shown on branches. *Pteridomonas* spp. are highlighted in light blue. Other non-photosynthetic algal species are highlighted in gray.
