# Supplementary material for: Highly Reduced Plastid Genomes of the Non-photosynthetic Dictyochophyceans Pteridomonas spp. (Ochrophyta, SAR) Are Retained for tRNA-Glu-Based Organellar Heme Biosynthesis
Source: Front Plant Sci. 2020 Nov 27;11:602455. doi: 10.3389/fpls.2020.602455 (PMC7728698; doi:10.3389/fpls.2020.602455)
Supplement: Supplementary file 5 [file Data_Sheet_5.PDF]

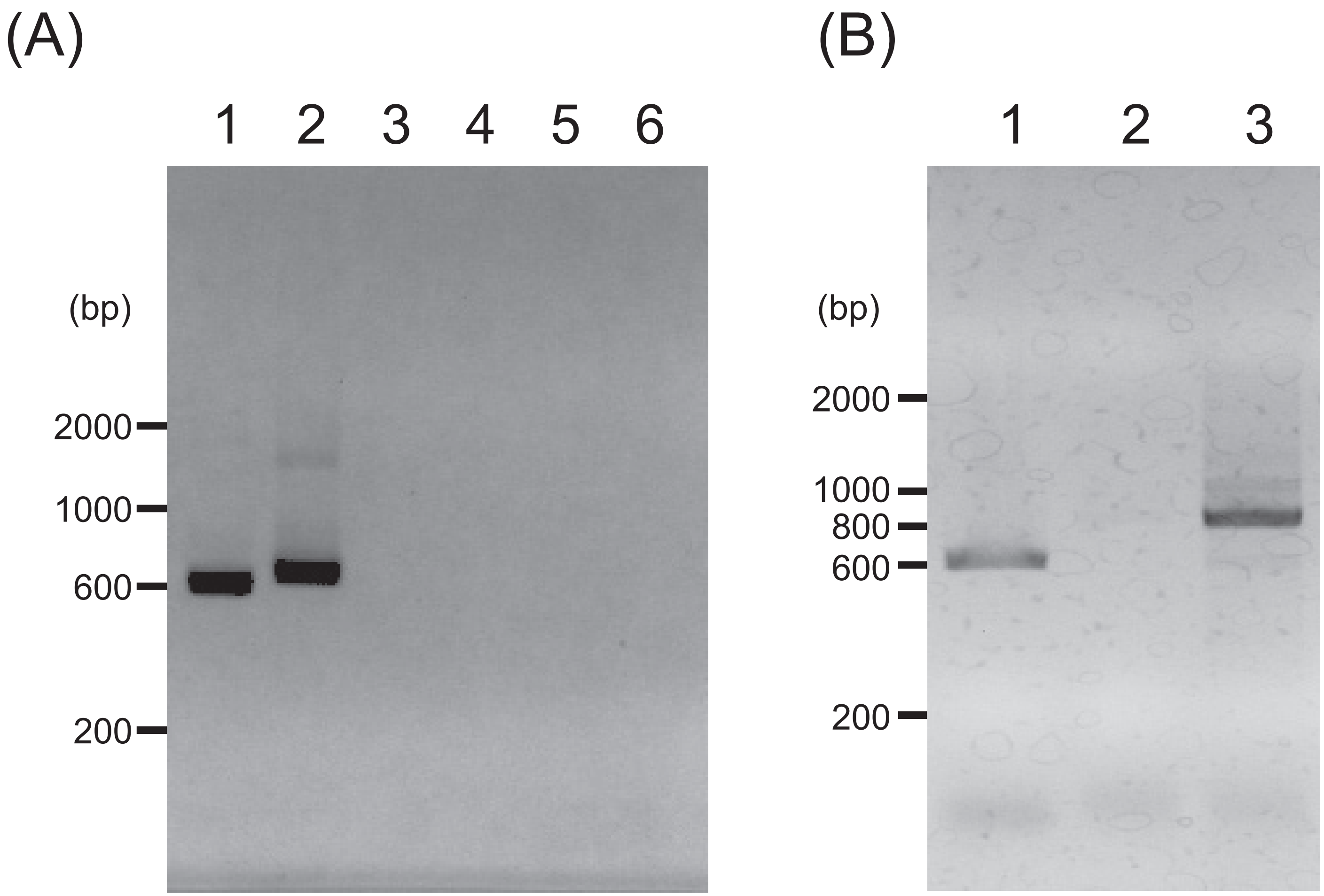

Figure S5. PCR assays for rbcL. A. PCR assays for the total DNA of the rbcL gene of *Pteridomonas* sp. strain YPF1301. Lane 1: nuclear actin gene PCR with the primer set Pterido_Actin_89_F and Pterido_Actin_693_R as a positive control, lane 2: plastid-encoded *rpl36* gene PCR with the primer set rpl36F and rpl36R as a positive con-trol, Lane 3: rbcL PCR with the primer set rbcLF1 and rbcLR1, Lane 4: rbcL PCR with the primer set rbcLF1 and rbcLR1 without DNA as a negative control, Lane 5: rbcL PCR with the primer set rbcLF2 and rbcLR2, Lane 6: rbcL gene PCR with the primer set rbcLF2 and rbcLR2 without DNA as a negative control. B. PCR assays for the total DNA of the *rbcL* gene with degenerate primers designed for *rbcL* of Stramenopiles. Lane 1: nuclear actin gene PCR with the primer set Pterido Actin 89 F and Pterido Actin 693 R as a positive control, Lane 2: rbcL gene PCR with the primer set stramenopiles RbcL F and stramenopiles RbcL R. Lane3: rbcL gene PCR with the primer set stramenopiles RbcL F and stramenopiles RbcL R for the diatom *P. tricornutum* DNA as a positive control.
