# Supplementary material for: Highly Reduced Plastid Genomes of the Non-photosynthetic Dictyochophyceans Pteridomonas spp. (Ochrophyta, SAR) Are Retained for tRNA-Glu-Based Organellar Heme Biosynthesis
Source: Front Plant Sci. 2020 Nov 27;11:602455. doi: 10.3389/fpls.2020.602455 (PMC7728698; doi:10.3389/fpls.2020.602455)
Supplement: Supplementary file 6 [file Data_Sheet_6.PDF]

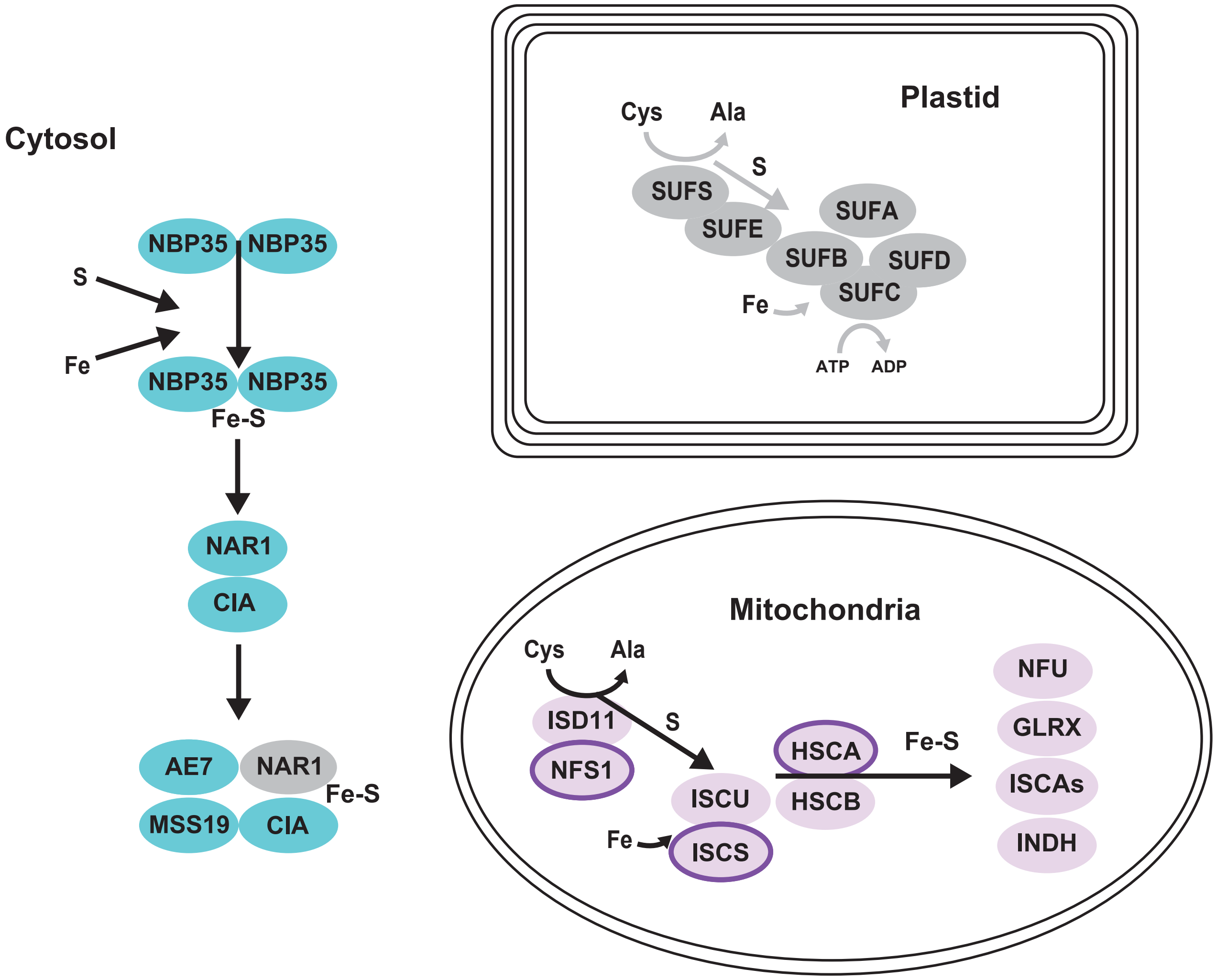

Supplementary Figure 6. Overview of iron-sulfur cluster assembly in *Pteridomonas danica* strain PT. Light purple circles enclosed by a purple line show proteins with the detectable mitochondrial targeting signals in the transcriptome data. Light purple circles with no line show proteins with no detectable mitochondrial-targeting signals probably due to lack of 5' termini of sequences in the transcriptome data. Cytosolic protein homologues are shown by light blue circles. Grey circles show missing protein sequences. AE7, cytosolic iron-sulfur assembly component AE7; Cfd1, Cytosolic Fe-S cluster assembly factor CFD1; Cia1, Cytosolic iron-sulfur protein assembly protein 1; Dre2, Fe-S cluster assembly protein DRE2; GLRX, Glutaredoxin-related protein; HscA, Fe-S protein assembly chaperone HscA; HscB, iron-sulfur cluster co-chaperone protein HscB; INDH, ATP binding protein-like; ISCA1, iron-sulfur cluster assembly 1; ISCA2, iron-sulfur cluster assembly 2; IscS, cysteine desulfurase; IscU, iron-sulfur cluster assembly enzyme; ISD11, Protein ISD11; MMS19, DNA repair/transcription protein MET18/MMS19; Nar1, Protein NAR1; Nbp35, Cytosolic Fe-S cluster assembly factor NBP35; NFS1, Cysteine desulfurase, mitochondrial; NFU4, NifU protein 4 (Balk and Schaedler 2014; Lill 2009; Grosche et al., 2018).
