# Supplementary material for: Highly Reduced Plastid Genomes of the Non-photosynthetic Dictyochophyceans Pteridomonas spp. (Ochrophyta, SAR) Are Retained for tRNA-Glu-Based Organellar Heme Biosynthesis
Source: Front Plant Sci. 2020 Nov 27;11:602455. doi: 10.3389/fpls.2020.602455 (PMC7728698; doi:10.3389/fpls.2020.602455)
Supplement: Supplementary file 7 [file Data_Sheet_7.pdf]

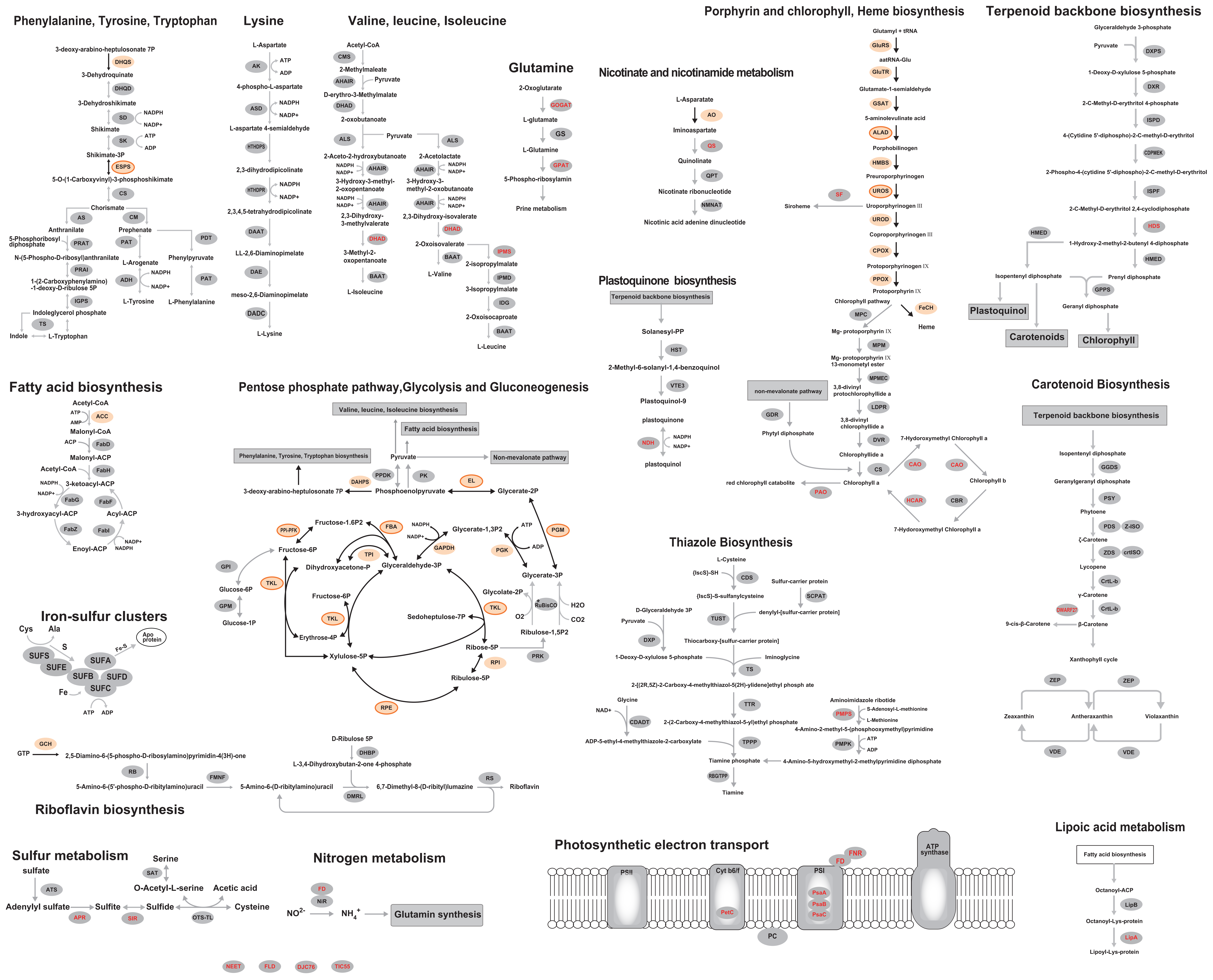
Phenylalanine, Tyrosine, Tryptophan
3-deoxy-arabino-heptulosonate 7P
DHQS
3-Dehydroquinate
DHQD
3-Dehydroshikimate
SD
NADPH
NADP+
Shikimate
SK
ATP
ADP
Shikimate-3P
ESPS
5-O-(1-Carboxyvinyl)-3-phosphoshikimate
CS
Chorismate
AS
CM
Anthranilate
Prephenate
PDT
5-Phosphoribosyl diphosphate
PRAT
PAT
N-(5-Phospho-D-ribosyl)anthranilate
L-Arogenate
Phenylpyruvate
PRAI
ADH
NADPH
NADP+
PAT
1-(2-Carboxyphenylamino)-1-deoxy-D-ribulose 5P
L-Tyrosine
L-Phenylalanine
IGPS
Indoleglycerol phosphate
TS
Indole
L-Tryptophan
Lysine
L-Aspartate
AK
ATP
ADP
4-phospho-L-aspartate
ASD
NADPH
NADP+
L-aspartate 4-semialdehyde
HTHDPS
2,3-dihydrodipicolinate
HTHDPR
NADPH
NADP+
2,3,4,5-tetrahydrodipicolinate
DAAT
LL-2,6-Diaminopimelate
DAE
meso-2,6-Diaminopimelate
DADC
L-Lysine
Valine, leucine, Isoleucine
Acetyl-CoA
CMS
2-Methylmaleate
AHAIR
Pyruvate
D-erythro-3-Methylmalate
DHAD
2-oxobutanoate
Pyruvate
ALS
ALS
2-Aceto-2-hydroxybutanoate
2-Acetolactate
NADPH
NADP+
AHAIR
AHAIR
3-Hydroxy-3-methyl-2-oxopentanoate
3-Hydroxy-3-methyl-2-oxobutanoate
AHAIR
AHAIR
2,3-Dihydroxy-3-methylvalerate
2,3-Dihydroxy-isovalerate
DHAD
DHAD
3-Methyl-2-oxopentanoate
2-Oxoisovalerate
IPMS
BAAT
2-isopropylmalate
BAAT
L-Valine
IPMD
L-Isoleucine
3-Isopropylmalate
IDG
2-Oxoisocaproate
BAAT
L-Leucine
Glutamine
2-Oxoglutarate
GOGAT
L-glutamate
GS
L-Glutamine
GPAT
5-Phospho-ribosylamin
Prine metabolism
Nicotinate and nicotinamide metabolism
L-Asparatate
AO
Iminoaspartate
QS
Quinolinate
QPT
Nicotinate ribonucleotide
NMNAT
Nicotinic acid adenine dinucleotide
Porphyrin and chlorophyll, Heme biosynthesis
Glutamyl + tRNA
GluRS
aatRNA-Glu
GluTR
Glutamate-1-semialdehyde
GSAT
5-aminolevulinate acid
ALAD
Porphobilinogen
HMBS
Preuroporphyrinogen
UROS
SF
Siroheme
Uroporphyrinogen III
UROD
Coproporphyrinogen III
CPOX
Protoporphyrinogen IX
PPOX
Protoporphyrin IX
Chlorophyll pathway
FeCH
MPC
Heme
Mg- protoporphyrin IX
MPM
Mg- protoporphyrin IX 13-monometyl ester
MPMEC
non-mevalonate pathway
3,8-divinyl protochlorophyllide a
LDPR
GDR
Phytyl diphosphate
3,8-divinyl chlorophyllide a
DVR
7-Hydoroxymethyl Chlorophyll a
Chlorophyllide a
CS
CAO
CAO
red chlorophyll catabolite
Chlorophyll a
Chlorophyll b
PAO
HCAR
CBR
7-Hydoroxymethyl Chlorophyll a
Terpenoid backbone biosynthesis
Glyceraldehyde 3-phosphate
Pyruvate
DXPS
1-Deoxy-D-xylulose 5-phosphate
DXR
2-C-Methyl-D-erythritol 4-phosphate
ISPD
4-(Cytidine 5'-diphospho)-2-C-methyl-D-erythritol
CDPMEK
2-Phospho-4-(cytidine 5'-diphospho)-2-C-methyl-D-erythritol
ISPF
2-C-Methyl-D-erythritol 2,4-cyclodiphosphate
HDS
HMED
1-Hydroxy-2-methyl-2-butenyl 4-diphosphate
HMED
Isopentenyl diphosphate
Prenyl diphosphate
GPPS
Plastoquinol
Geranyl diphosphate
Carotenoids
Chlorophyll
Plastoquinone biosynthesis
Terpenoid backbone biosynthesis
Solanesyl-PP
HST
2-Methyl-6-solanyl-1,4-benzoquinol
VTE3
Plastoquinol-9
plastoquinone
NDH
NADPH
NADP+
plastoquinol
Fatty acid biosynthesis
Acetyl-CoA
ATP
AMP
ACC
Malonyl-CoA
ACP
FabD
Malonyl-ACP
Acetyl-CoA
FabH
NADPH
NADP+
3-ketoacyl-ACP
FabG
FabF
3-hydroxyacyl-ACP
Acyl-ACP
FabZ
FabI
NADP+
NADPH
Enoyl-ACP
Pentose phosphate pathway,Glycolysis and Gluconeogenesis
Valine, leucine, Isoleucine biosynthesis
Fatty acid biosynthesis
Phenylalanine, Tyrosine, Tryptophan biosynthesis
Pyruvate
Non-mevalonate pathway
PPDK
PK
DAHPS
EL
3-deoxy-arabino-heptulosonate 7P
Phosphoenolpyruvate
Glycerate-2P
Fructose-1.6P2
FBA
NADPH
PPi-PFK
NADP+
Glycerate-1,3P2
ATP
PGM
Fructose-6P
TPI
GAPDH
ADP
PGK
Dihydroxyacetone-P
Glyceraldehyde-3P
Glycerate-3P
GPI
TKL
Glucose-6P
Fructose-6P
Glycolate-2P
GPM
TKL
RuBisCO
H2O
TKL
Sedoheptulose-7P
O2
CO2
Glucose-1P
Erythrose-4P
Ribulose-1,5P2
Xylulose-5P
Ribose-5P
PRK
RPI
Ribulose-5P
RPE
Thiazole Biosynthesis
L-Cysteine
{IscS}-SH
CDS
Sulfur-carrier protein
{IscS}-S-sulfanylcysteine
SCPAT
D-Glyceraldehyde 3P
TUST
denylyl-[sulfur-carrier protein]
Pyruvate
DXP
Thiocarboxy-[sulfur-carrier protein]
1-Deoxy-D-xylulose 5-phosphate
Iminoglycine
TS
2-[(2R,5Z)-2-Carboxy-4-methylthiazol-5(2H)-ylidene]ethyl phosph ate
Glycine
TTR
Aminoimidazole ribotide
NAD+
S-Adenosyl-L-methionine
CDADT
PMPS
2-(2-Carboxy-4-methylthiazol-5-yl)ethyl phosphate
L-Methionine
4-Amino-2-methyl-5-(phosphooxymethyl)pyrimidine
ATP
ADP-5-ethyl-4-methylthiazole-2-carboxylate
TPPP
PMPK
ADP
Tiamine phosphate
4-Amino-5-hydroxymethyl-2-methylpyrimidine diphosphate
RBG/TPP
Tiamine
Carotenoid Biosynthesis
Terpenoid backbone biosynthesis
Isopentenyl diphosphate
GGDS
Geranylgeranyl diphosphate
PSY
Phytoene
PDS
Z-ISO
ζ-Carotene
ZDS
crtISO
Lycopene
CrtL-b
γ-Carotene
DWARF27
CrtL-b
9-cis-β-Carotene
β-Carotene
Xanthophyll cycle
ZEP
ZEP
Zeaxanthin
Antheraxanthin
Violaxanthin
VDE
VDE
Iron-sulfur clusters
Cys
Ala
S
Fe-S
Apo protein
SUFS
SUFE
SUFA
SUFB
SUFD
SUFC
Fe
ATP
ADP
GCH
GTP
2,5-Diamino-6-(5-phospho-D-ribosylamino)pyrimidin-4(3H)-one
RB
D-Ribulose 5P
DHBP
L-3,4-Dihydroxybutan-2-one 4-phosphate
FMNF
RS
5-Amino-6-(5'-phospho-D-ribitylamino)uracil
5-Amino-6-(D-ribitylamino)uracil
6,7-Dimethyl-8-(D-ribityl)lumazine
Riboflavin
DMRL
Riboflavin biosynthesis
Sulfur metabolism
Serine
sulfate
SAT
ATS
O-Acetyl-L-serine
Acetic acid
Adenylyl sulfate
Sulfite
Sulfide
Cysteine
APR
SIR
OTS-TL
Nitrogen metabolism
FD
NiR
NO2-
NH4+
Glutamin synthesis
Photosynthetic electron transport
FNR
ATP synthase
FD
PSII
Cyt b6/f
PSI
PsaA
PsaB
PetC
PsaC
PC
Lipoic acid metabolism
Fatty acid biosynthesis
Octanoyl-ACP
LipB
Octanoyl-Lys-protein
LipA
Lipoyl-Lys-protein
NEET
FLD
DJC76
TIC55

Supplementary Figure 7. Plastid metabolism of non-photosynthetic *Pteridomonas danica* strain PT. Light orange circles enclosed by an orange line show proteins with the detectable plastid targeting signals. Light orange circles with no line show proteins with no detectable plastid targeting signal probably due to lack of 5' termini of sequences in the transcriptome data. Gray circles show proteins not detected. The asterisk shows that it is unclear whether RuBisCO is present as both large and small subunits of RuBisCO are plastid-encoded proteins; A plastid genome of strain PT is unavailable. ACC, acetyl-coa carboxylase; ALAD, porphobilinogen synthase; AO, L-aspartate oxidase; APR, adenylyl-sulfate reductase; AS, anthranilate s ynthase component I; A TS, ATP s ulfurylas e; CAO , chlorophyll a oxygenase; CBR, chlorophyll b reductase; CDP MEK, 4-diphosphocytidyl-2-C-methyl-D-erythritol kinase; CDS, cysteine desulfurase; CM, chorismate mutase; CMO, choline monooxygenase; CPOX, coproporphyrinogen III oxidase; CPS, chlorophyll synthase; crtISO, prolycopene isomerase; CrtL-b, lycopene beta-cyclase; CS, chorismate synthase; Cyt b6/f, cytochrome b6f complex; D27, beta-carotene isomerase D27; DADC, diaminopimelate decarboxylase; DJC76, Chaperone protein dnaJ C76; DHAD, dihydroxy-acid dehydratase; DHBP, 3, 4-dihydroxy 2-butanone 4-phosphate synthase / GTP cyclohydrolase II; DHQS, 3-dehydroquinate synthase; DVR, divinyl chlorophyllide a 8-vinyl-reductase; DXPS, 1-deoxy-D-xylulose-5-phosphate synthase; DXR, 1-deoxy-D-xylulose-5-phosphate reductoisomerase; EL, enolase; ESPS, 3-phosphoshikimate 1-carboxyvinyltransferase; FabD, [acyl-carrier-protein] S-malonyltransferase; FabF, 3-oxoacyl-[acyl-carrier-protein] synthase II; FabH, 3-oxoacyl-[acyl-carrier-protein] synthase III; FabG, 3-oxoacyl-[acyl-carrier protein; FabZ, 3-hydroxyacyl-[acyl-carrier-protein] dehydratase; FBA, fructose-bisphosphate aldolase class II; FBP, fructose-1, 6-bisphosphatase I; FD, Ferredoxin; FeCH, protoporphyrin/coproporphyrin ferrochelatase; FLD, Flavodoxin ; FNR, ferredoxin-NADP reductase; FTR, ferredoxin-thioredoxin reductase; GAPDH, glyceraldehyde 3-phosphate dehydrogenase; GCH, 3,4-dihydroxy 2-butanone 4-phosphate synthase / GTP cyclohydrolase II; GDR, geranylgeranyl diphosphate reductase; GGDS, geranylgeranyl diphosphate synthase; GluRS, glutamyl-tRNA synthetase; GluTR, glutamyl-trna reductase; GOGAT, glutamate synthase (ferredoxin); GPAT, glutamine phosphoribosylpyrophosphate amidotransferase; GPI, glucose-6-phosphate isomerase; GPPS, geranyl pyrophosphate synthase; GS2, glutamate synthase; GSAT, glutamate-1-semialdehyde 2,1-aminomutase; HCAR, 7-hydroxymethyl chlorophyll a reductase; HDR, 4-hydroxy-3-methylbut-2-en-1-yl diphosphate reductase; HMBS, hydroxymethylbilane synthase; HMED, (E)-4-hydroxy-3-methylbut-2-enyl-diphosphate synthase; HST, homogentisate solanesyltransferase; IGPS, indole-3-glycerol phosphate synthase; IPMI, isopropyl malate isomerase; ISPD, 4-hydroxy-3-methylbut-2-en-1-yl diphosphate reductase; ISPF, 2-C-methyl-D-erythritol 2, 4-cyclodiphosphate synthase; ISPG, 4-hydroxy-3-methylbut-2-enyl diphosphate synthase; KDPHS, 3-deoxy-7-phosphoheptulonate synthase; LDPR, protochlorophyllide reductase; LipA, lipoic acid synthetase; LipB, lipoyl(octanoyl) transferase; MAT, [acyl-carrier-protein] S-malonyltransferase; MGDG, monogalactosyldiacylglycerol synthase; MOD1, enoyl-[acyl-carrier protein] reductase I; MPC, magnesium-chelatase; MPM, Magnesium protoporphyrin IX methyltransferase; MPMEC, magnesium-protoporphyrin IX monomethyl ester (oxidative) cyclase; NDH, NAD(P)H-quinone oxidoreductase; NEET, iron-sulfur domain-containing protein NEET; NIR, Ferredoxin-nitrite reductase; NMNAT, nicotinamide mononucleotide adenylyltransferase; OTS-TL, O-acetylserine (thiol)-lyase; PAO, pheophorbide a oxygenase; PAT, bifunctional aspartate aminotransferase and glutamate/aspartate-prephenate aminotransferase; PC, plastocyanin; PCK, phosphoenolpyruvate carboxykinase; PDS, 15-cis-phytoene desaturase; PetC, cytochrome b6-f complex; PFK1, 6-phosphofructokinase 1; PGK, phosphoglycerate kinase; PGM, phosphoglucomutase; PK, pyruvate kinase; PPDK, phosphate dikinase; PPOX, protoporphyrinogen/coproporphyrinogen III oxidase; PRK, phosphoribulokinase; PQ, plastoquinone; PQH2, plastoquinol; PRA, phosphoribosylanthranilate isomerase; PRAT, anthranilate phosphoribosyltransferase; PsaA, Photosystem I P700 chlorophyll a apoprotein A1; PsaA, Photosystem I P700 chlorophyll a apoprotein; PsaB, Photosystem I P700 chlorophyll a apoprotein A2; PsaC, Photosystem I iron-sulfur center; PSI, Photosystem I; PSII, Photosystem II; PSY, phytoene synthetase; PTC52, Protochlorophyllide-dependent translocon component 52; QS, Quinolinate synthase; QPT, quinolinate phoshoribosyltransferase; RuBisCO, ribulose-1,5-bisphosphate carboxylase/oxygenase large and small subunits; RPE, ribulose-phosphate 3-epimerase; RPI, ribose 5-phosphate isomerase; SAT, serine O-acetyltransferase; SEC61A, SEC61-alpha subunit of ER-translocon; SIR, sulfite reductase; SIRB, sirohydrochlorin ferrochelatase; SUFA, B, C, D, E, S, cysteine desulfurase; TAL, transaldolase; TIC55, translocator of the inner chloroplast envelope membrane 55; THIC, thiamine biosynthesis; TKL, transketolase; TPI, triosephosphate isomerase; TPT, triose phosphate phosphate translocator; UROD, Uroporphyrinogen decarboxylase; UROS, uroporphyrinogen III synthase; VDE, violaxanthin de-epoxidase; VTE3, MPBQ/MSBQ methyltransferase; ZDS, zeta-carotene desaturase; ZEP, zeaxanthin epoxidase; Z-ISO, zeta-carotene isomerase (Bock and Khan, 2004; DellaPenna and Pogson, 2006; Hiltunen et al., 2012; Kamikawa et al., 2017; Przybyla-Toscano et al., 2018).
