# Supplementary material for: Highly Reduced Plastid Genomes of the Non-photosynthetic Dictyochophyceans Pteridomonas spp. (Ochrophyta, SAR) Are Retained for tRNA-Glu-Based Organellar Heme Biosynthesis
Source: Front Plant Sci. 2020 Nov 27;11:602455. doi: 10.3389/fpls.2020.602455 (PMC7728698; doi:10.3389/fpls.2020.602455)
Supplement: Supplementary file 8 [file Data_Sheet_8.PDF]

18S rRNA dataset
>U14388.1_Rhizochromulina_cf_marina
TCTAAGTATAAGCGTTATACTGTGAAACTGCGAGGCTCATAAACCAGTTATAGT
TTATTTGATATTTCCCTACTACTCGGATACCCGTAGTAATTCTAGAGCTAATACG
TGAATGAAGCTCCGACTGGAGTGTCATTATTAGATAGAAACCAATCGCAAGGGT
ATTGTGGTGAGTCATGATAATTAGCGGATCGCATGGCCTGCGCCGGCGACGGA
TCATTCAATTCTGCCCTATCAGCTTGGATGGTACGGTATTGGCCTACCATGGCT
TTAACGGGTAACGGAGAATAGGGTTCGATTCCGGAGGGAGCCTGAGAGACGGC
TACCACATCCAAGGAAGGCAGCAGGCGCGTAAATTACCCAATCCTGACACAGGG
AGGTAGTGACAAGAAATAACTTAGTCGGGCCTTTACGGTCTGACTTTAGGAATG
AGAACAATTTAAATCCCTTATCGAGGATCAATTGGAGGGCAAGTCTGGTGCCAG
CAGCCGCGGTAATTCCAGCTCCAATAGCGTATATTAATGTTGTTGCAGTTAAAA
AGCTCGTAGTTGGATTTCGAATGGAGTGACGGTCGGCCGCAGGGTGTGCGGCA
TTGGGCCCGTCGTTTCTGTGAACAAATTAGAGTGTTCCAAGCAAGGCTTGAATA
CATTAGCATGGAATAATAGATAGGACTTGTGGTATTTTGTTGGTTGCACGAAAG
TAATGATTAATAGGACAGTGGGGGTTTCGTATTCAATTGTCAGAGGTGAAATTC
TTGGATTTCTGGACGAACTACTGCGAAAGCATTTACCAAGGATGTTTTCATTAA
TCAAGAACGAAAGTTAGGGGACGAAGAAGATTAGATACCTTCGTATCTAACCAT
AAACGATGCCGACTCGGGATTGGCGGTCGGACTCCGCAGCACCGTATGAGAAAT
CAAAGTCTTTGGGTTCCGGGGGGAGTATGGTCGCAAGGCTGAAACTTAAAGAAA
TTGACGGAAGGGCACCACCAGGAGTGGAGCCTGCGGCTTAATTTGACTCAACAC
GGGGAAACTACCAGGTCCAGACATAGTAAGGATGACAGATTGAGAGCTCTTTCT
TGATTCTTGGGTGGTGGTGCATGGCCGTTCTTAGTTGGTGGAGTGATTTGTCT
GGTTAATTCCGTAACGAACGAGACCCCCGCCTGCTAAATAGCAGGAATGCATCG
CATGTTCGCTTCTTAGAGGGACTGTCAGTGACTAACTGACGGAAGTTGGGGGCA
ATAACGGTCTGTGATGCCCTTAGATGTCCTGGGCCGCACGCGCGCTACACTGAT
CATGCAACGAGTCTCCTTGGCTGGAAGGCCTGGGAATCTGAACGTGATCGTGAA
GGGATAGATTATTGCAATTATTAATCTTGAACGAGGAATTCCTAGTAACGCAAG
TCATCAGCTTGCATGATTACTCCCGCCCTTGTACACACCGCCCGTCGCCCTACCA
TCGAAGGTAATGATGAGCCCTAGATTGTGGCTTGGGAC
>U14387.1_Pseudopedinella_elastica
TCTAAGTATAAACGCTATACTGTGAAACTGCGAGGCTCATAAACCAGTTATAGTT
TATTTGATATTCCCCTACTACTCGGATACCCGTAGTAATTCTAGAGCTAATACGT
GCGTCAAACGACATTTGTCGTGTCATTATTAGATAGAAACCAATCTTCGGGGTA
TTGTGGTGAGTCATGATAATTAGCGGATCGCATGGTCTGTACCGGCGATGGATC
ATTCAATTCTGCCCTATCAGCTTGGATGGTAGGGTATTGGCCTACCATGGCTTT

AACGGGTAACGGAGAATAGGGTTCGATTCCGGAGGGAGCCTGAGAGACGGCTA
CCACATCCAAGGAAGGCAGCAGGCGCGTAAATTACCCAATCCTGACACAGGGAG
GTAGTGACAAAAAATAACTTAGTCGGGCCGTTTTGGTCTGACTTTAGGAATGAG
AACAATTTAAATCCCTTATCGAGGATCAATTGGAGGGCAAGTCTGGTGCCAGCA
GCCGCGGTAATTCCAGCTCCAATAGCGTATATTAATGTTGTTGCAGTTAAAAAG
CTCGTAGTTGGATTTCTGGATGAGCGGCGGCCTTGCGCACGTTGCGCGGCATT
ACGCCTGTCGTTTCTGTGAACAAATTAGAGTGTTCAAAGCAAGGTTTGAATACA
TTAGCATGGAATAATAGATAGGACTTGTGGTATTTTGTTGGTTGCACGAAAGTA
ATGATTAATAGGACAGTGGGGGTTTCGTATTCAATTGTCAGAGGTGAAATTCTT
GGATTTATGGACGAACTACTGCGAAAGCATTTACCAAGGATGTTTTCATTAATC
AAGAACGAAAGTTAGGGGACGAAGAAGATTAGATACCTTCGTATCTAACCATAAA
CTATGCCGACTCGGGATTGGCGGTCGGACTCCGCAGCACCGTATGAGAAATCAA
AGTCTTTGGGTTCCGGGGGGAGTATGGTCGCAAGGCTGAAACTTAAAGAAATT
GACGGAAGGGCACCACCAGGAGTGGAGCCTGCGGCTTAATTTGACTCAACACGG
GGAAACTACCAGGTCCAGACATAGTAAGGATGACAGATTGAGAGCTCTTTCTTG
ATTCTTGGGTGGTGGTGCATGGCCGTTCTTAGTTGGTGGAGTGATTTGTCTGG
TTAATTCCGTAACGAACGAGACCCCCGCCTGCTAAATAGTTGGAATGCTTCGCA
TGATCGCTTCTTAGAGGGACTTCGTGTGACTAACACGAGGAAGTTGGGGGCAAT
AACGGTCTGTGATGCCCTTAGATGTTCTGGGCCGCACGCGCGCTACACTGATCA
TGCAACGAGTTTCCTTGGCCGGGAGGCCTGGGAATCTGAACGTGATCGTGAAG
GGATAGATTATTGCAATTATTAATCTTGAACGAGGAATTCCTAGTAACGCAAGTC
AACAGCTTGCATGATTACTCCCGCCCTTGTACACACCGCCCGTCGCCCTACCATC
GAAGGTAATGATGAGCCCCAGACAGTCGATTGGCGC
>U14384.1_Apedinella_radians
TCTAAGTATAAACGCTATACTGTGAAACTGCGAGGCTCATAAACCAGTTATAGTT
TATTTGATATTCCCCTACTACTTGGATACCCGTAGTAATTCTAGAGCTAATACAT
GCGTCAAGCGACATTTGTCGTGTCATTATTAGATAGAAACCAATCTTCGGGGAA
TTGTGGTGAATCATGATAATTAGCGGATCGCATGGCCTGCGCCGGCGATGGAT
CATTCAATTCTGCCCTATCAGCTTGGATGGTAGGGTATTGGCCTACCATGGCTT
TAACGGGTAACGGAGAATAGGGTTCGATTCCGGAGGGAGCCTGAGAGACGGCT
ACCACATCCAAGGAAGGCAGCAGGCGCGTAAATTACCCAATCCTGACTCAGGGA
GGTAGTGACAAAAAATAACTTAGTCGGGCCGTTTTGGTCTGACTTTAGGAATGA
GAACAATTTAAATCCCTTATCGAGGATCAATTGGAGGGCAAGTCTGGTGCCAGC
AGCCGCGGTAATTCCAGCTCCAATAGCGTATATTAATGTTGTTGCAGTTAAAAA
GCTCGTAGTTGGATTTCTGGGTCTTCGCTGCTTTGCCGCAGGCTATGCGGTAT
TAAGCCTGTCGTTTCTGTGAACAAATTAGAGTGTTCAAAGCAAGGTTTGAATAC

ATTAGCATGGAATAATAGATAGGACTTGTGGTATTTTGTTGGTTGCACGAAAGT
AATGATTAATAGGACAGTGGGGGTTTCGTATTCAATTGTCAGAGGTGAAATTCT
TGGATTTATGGACGAACTACTGCGAAAGCATTTACCAAGGATGTTTTCATTAAT
CAAGAACGAAAGTTAGGGGACGAAGAAGATTAGATACCTTCGTATCTAACCATA
AACGATGCCGACTCGGGATTGGCGGTCGGTCTCCGCAGCACCGTATGAGAAATC
AAAGTCTTTGGGTTCCGGGGGGAGTATGGTCGCAAGGCTGAAACTTAAAGAAAT
TGACGGAAGGGCACCACCAGGAGTGGAGCCTGCGGCTTAATTTGACTCAACACG
GGGAAACTACCAGGTCCAGACATAGGAAGGATGACAGATTGAGAGCTCTTTCTT
GATTCTTGGGTGGTGGTGCATGGCCGTTCTTAGTTGGTGGAGTGATTTGTCTG
GTTTATTCCGTAACGAACGAGACCCCTGCCTGCTAAATAGCCGGAATGCTTCGC
ATGCTCGCTTCTTAGAGGGACTGTATGGGACTACCATACGGAAGTTGGGGGCAA
TAACGGTCTGTGATGCCCTTAGATGTTCTGGGCCGCACGCGCGCTACACTGATC
GTGCAACAAGTTTCCTTGGCCGAGAGGCCTGGGAATCTGAACGCGATCGTGAAG
GGATAGATTATTGCAATTATTAATCTTGAACGAGGAATTCCTAGTAACGCAAGTC
ATCAGCTTGCATGATTACTCCCGCCCTTGTACACACCGCCCGTCGCCCTACCATC
GAAGGTAATGATGAGCCGCAGACCGTCGGTTTGGCC
>Triparma_strigata_strain_NIES-3701_KR998402
TCTAAGTATAAACGTTATACTGTGAAACTGCGAGGCTCATATATCAGTTATAGTT
TATTTGATAATCTCTTACTACTTGGATAACCGTAGTAATTCTAGAGCTAATACAT
GCGTAAACACCCAACTGTTTGGTATTTATTAGATTGAAACCAATCTTCGGAGATT
TGTGGTGATTCATAATAACTTGCGGATCGCATGGCCTGTGCCGGCGACAGATCA
TTCAATTCTGCCCTATCAGCTTGGATGGTAGGGTATTGGCCTACCATGGCTTTA
ACGGGTAACGGAGAATAGGGTTCGATTCCGGAGGGAGCCTGAGAGACGGCTAC
CACATCCAAGGAAGGCAGCAGGCGCGTAAATTACCCAATCCTGACACAGGGAGG
TAGTGACAATAAATAACAATGCCGGGCCTTTTAGGTCTGGCAATTGGAATGAGA
ACAATTTAAATCCCTTATCGAGGATCAATTGGAGGGCAAGTCTGGTGCCAGCAG
CCGCGGTAATTCCAGCTCCAATAGCGTATATTAAAGTTGTTGCAGTTAAAAAGC
TCGTAGTTGGATTTCTGGTGGGACGTTGGCCGGCCGTAGGTTGGTTGCCATTA
GCGTCATCGTTTCTGTGAAAAAATTAGAGTGTTCAAAGCAGGCTTTGAATACAT
TAGCATGGAATAATAGATAGGACCCGTACTATTTTGTTGGTTGCGTATAGGTAA
TGATTAATAGGGCAGTGGGGGTTTTGTATTCCGTTGTCAGAGGTGAAATTCTTG
GATTTACGGACAAACTACTGCGAAAGCATTTACCAAGGATGTTTTCATTAATCAA
GAACGAAAGTTAGGGGACGAAGATGATTAGATACCATCGTATTTAACCATAAAC
TATGCCGACTAGGGATTGGCGGTCGGACTCCGCAGCACCTTATGAGAAATCAAA
GTCTTTGGGTTCCGGGGGGAGTATGGTCGCAAGGCTGAAACTTAAAGAAATTG
ACGGAAGGGCACCACCAGGAGTGGAGCCTGCGGCTTAATTTGACTCAACACGGG

AAAACTACCAGGTCCAGACATAGTAAGGATGACAGATTGAGAGCTCTTTCTTGA
TTCTTGGGTGGTGGTGCATGGCCGTTCTTAGTTGGTGGAGTGATTTGTCTGGT
TAATTCCGTAACGAACGAGACCCCCGCCTGCTAAATAGTGCTAATGAATTTCATG
CCGTCTTCTTAGAGGGACTTTGCGTGACTAACGCAAGGAAGTTGGGGGCAATAA
CGGTCTGTGATGCCCTTAGATGTTCTGGGCCGCACGCGCGCTACACTGATCATT
CAACGAGTTTCCTTGACCGAGAGGTCTGGGAATCTAAACGTGATCGTGAAGGGA
TAGATTATTGCAATTATTAATCTTGAACGAGGAATTCCTAGTAACGCAGTTCATC
AGACTGCATGATTACTCCCGCCCTTGTACACACCGCCCGTCGCCCTACCATTGA
ATGGTCCGGTGAAATTCGGACTGAGCGATTCTGA
>Rhizochromulina_marina_U14388
TCTAAGTATAAGCGTTATACTGTGAAACTGCGAGGCTCATAAACCAGTTATAGT
TTATTTGATATTTCCCTACTACTCGGATACCCGTAGTAATTCTAGAGCTAATACG
TGAATGAAGCTCCGACTGGAGTGTCATTATTAGATAGAAACCAATCGCAAGGGT
ATTGTGGTGAGTCATGATAATTAGCGGATCGCATGGCCTGCGCCGGCGACGGA
TCATTCAATTCTGCCCTATCAGCTTGGATGGTACGGTATTGGCCTACCATGGCT
TTAACGGGTAACGGAGAATAGGGTTCGATTCCGGAGGGAGCCTGAGAGACGGC
TACCACATCCAAGGAAGGCAGCAGGCGCGTAAATTACCCAATCCTGACACAGGG
AGGTAGTGACAAGAAATAACTTAGTCGGGCCTTTACGGTCTGACTTTAGGAATG
AGAACAATTTAAATCCCTTATCGAGGATCAATTGGAGGGCAAGTCTGGTGCCAG
CAGCCGCGGTAATTCCAGCTCCAATAGCGTATATTAATGTTGTTGCAGTTAAAA
AGCTCGTAGTTGGATTTCGAATGGAGTGACGGTCGGCCGCAGGGTGTGCGGCA
TTGGGCCCGTCGTTTCTGTGAACAAATTAGAGTGTTCCAAGCAAGGCTTGAATA
CATTAGCATGGAATAATAGATAGGACTTGTGGTATTTTGTTGGTTGCACGAAAG
TAATGATTAATAGGACAGTGGGGGTTTCGTATTCAATTGTCAGAGGTGAAATTC
TTGGATTTCTGGACGAACTACTGCGAAAGCATTTACCAAGGATGTTTTCATTAA
TCAAGAACGAAAGTTAGGGGACGAAGAAGATTAGATACCTTCGTATCTAACCAT
AAACGATGCCGACTCGGGATTGGCGGTCGGACTCCGCAGCACCGTATGAGAAAT
CAAAGTCTTTGGGTTCCGGGGGGAGTATGGTCGCAAGGCTGAAACTTAAAGAAA
TTGACGGAAGGGCACCACCAGGAGTGGAGCCTGCGGCTTAATTTGACTCAACAC
GGGGAAACTACCAGGTCCAGACATAGTAAGGATGACAGATTGAGAGCTCTTTCT
TGATTCTTGGGTGGTGGTGCATGGCCGTTCTTAGTTGGTGGAGTGATTTGTCT
GGTTAATTCCGTAACGAACGAGACCCCCGCCTGCTAAATAGCAGGAATGCATCG
CATGCTTGCTTCTTAGAGGGACTGTCAGTGACTAACTGACGGAAGTTGGGGGCA
ATAACGGTCTGTGATGCCCTTAGATGTCCTGGGCCGCACGCGCGCTACACTGAT
CATGCAACGAGTTCCCTTGGCTGGAAGGCCTGGGAATCTGAACGTGATCGTGAA
GGGATAGATTATTGCAATTATTAATCTTGAACGAGGAATTCCTAGTAACGCAAG

TCATCAGCTTGCATGATTACTCCCGCCCTTGTACACACCGCCCGTCGCCCTACCA
TCGAAGGTAATGATGAGCCCTAGATTGTGGCTTGGACG
>Pteridomonas_sp._strain_IOW124_KX431481
--------------CTATACTGTG-
AACTGCGAGGCTCATAAACCAGTTATAGTTTATTTGATATTCCCCTACTACTTGG
ATACCCGTAGTAATTCTAGAGCTAATACATGCGTCAAGCGACATATGTCGTGTC
ATTATTAGATAGAAACCAATCTTCGGGGAATTGTGGTGAGTCATGATAATTAGC
GGATCGCATGGCTTACGCCGGCGATGGATCATTCAATTCTGCCCTATCAGCTTG
GATGGTAGGGTATTGGCCTACCATGGCTTTAACGGGTAACGGAGAATAGGGTT
CGATTCCGGAGGGAGCCTGAGAGACGGCTACCACATCCAAGGAAGGCAGCAGG
CGCGTAAATTACCCAATCCTGACTCAGGGAGGTAGTGACAAAAAATAACTTAGTC
GAGCCGTTTTGGTTTGACTTTAGGAATGAGAACAATTTAAATCCCTTATCGAGG
ATCAATTGGAGGGCAAGTCTGGTGCCAGCAGCCGCGGTAATTCCAGCTCCAATA
GCGTATATTAATGTTGTTGCAGTTAAAAAGCTCGTAGTTGGATTTCTGGTCGGA
CGTCGGCCGGCCGCAGGATGTGCGGTATTACGCCTGTCGTTTCTGTGAACAAAT
TAGAGTGTTCAAAGCAAGGTTTGAATACATTAGCATGGAATAATAGATAGGACT
TGTGGTATTTTGTTGGTTGCACGAAAGTAATGATTAATAGGACAGTGGGGGTTT
CGTATTCAATTGTCAGAGGTGAAATTCTTGGATTTATGGACGAACTACTGCGAA
AGCATTTACCAAGGATGTTTTCATTAATCAAGAACGAAAGTTAGGGGACGAAGA
AGATTAGATACCTTCGTATCTAACCATAAACCATGCCGACTCGGGATTGGCGGT
CGGACTCCGCAGCACCGTATGAGAAATCAAAGTCTTTGGGTTCCGGGGGGAGTA
TGGTCGCAAGGCTGAAACTTAAAGAAATTGACGGAAGGGCACCACCAGGAGTGG
AGCCTGCGGCTTAATTTGACTCAACACGGGGAAACTACCAGGTCCAGACATAGT
AAGGATGACAGATTGAG---

>Pteridomonas_sp._strain_IOW122_KX431480.1

CTGCGAGGCTCATAAACCAGTTATAGTTTATTTGATATTCCCCTACTACTTGGAT
ACCCGTAGTAATTCTAGAGCTAATACATGCGTCAAGCGACATATGTCGTGTCAT
TATTAGATAGAAACCAATCTTCGGGGAATTGTGGTGAGTCATGATAATTAGCGG
ATCGCATGGCCTGTGCCGGCGATGGATCATTCAATTCTGCCCTATCAGCTTGGA
TGGTAGGGTATTGGCCTACCATGGCTTTAACGGGTAACGGAGAATAGGGTTCG
ATTCCGGAGGGAGCCTGAGAGACGGCTACCACATCCAAGGAAGGCAGCAGGCG

CGTAAATTACCCAATCCTGACTCAGGGAGGTAGTGACAAAAAATAACTTAGTCGA
GCCGTTTTGGTTTGACTTTAGGAATGAGAACAATTTAAATCCCTTATCGAGGAT
CAATTGGAGGGCAAGTCTGGTGCCAGCAGCCGCGGTAATTCCAGCTCCAATAGC
GTATATTAATGTTGTTGCAGTTAAAAAGCTCGTAGTTGGATTTCTGGTCGAACG
CCGGCCGGCCGCAGGGTGCTCGGTATTACGCCTGTCGTTTCTGTGAACAAATTA
GAGTGTTCAAAGCAAGGTTTGAATACATTAGCATGGAATAATAGATAGGACTTG
TGGTATTTTGTTGGTTGCACGAAAGTAATGATTAATAGGACAGTGGGGGTTTCG
TATTCAATTGTCAGAGGTGAAATTCTTGGATTTATGGACGAACTACTGCGAAAG
CATTTACCAAGGATGTTTTCATTAATCAAGAACGAAAGTTAGGGGACGAAGAAG
ATTAGATACCTTCGTATCTAACCATAAACCATGCCGACTCGGGATTGGCGGTCG
GACTCCGCAGCACCGTATGAGAAATCAAAGTCTTTGGGTTCCGGGGGGAGTATG
GTCGCAAGGCTGAAACTTAAAGAAATTGACGGAAGGGCACCACCAGGAGTGGAG
CCTGCGGCTTAATTTGACTCAACACGGGGAAACTACCAGGTCCAGACATAGTAA
GGATGACAGATTGAGAGCTCTT---

>Pteridomonas_sp._strain_HD2ct5.5_KX431492
TCTAAGTATAAACGCTATACTGTGAAACTGCGAGGCTCATAAACCAGTTATAGTT
TATTTGATATTCCCCTACTACTTGGATACCCGTAGTAATTCTAGAGCTAATACAT
GCGTCAAGCGACATATGTCGTGTCATTATTAGATAGAAACCAATCTTCGGGGAA
TTGTGGTGAGTCATGATAATTAGCGGATCGCATGGCTTACGCCGGCGATGGAT
CATTCAATTCTGCCCTATCAGCTTGGATGGTAGGGTATTGGCCTACCATGGCTT
TAACGGGTAACGGAGAATAGGGTTCGATTCCGGAGGGAGCCTGAGAGACGGCT
ACCACATCCAAGGAAGGCAGCAGGCGCGTAAATTACCCAATCCTGACTCAGGGA
GGTAGTGACAAAAAATAACTTAGTCGAGCCGTTTTGGTTTGACTTTAGGAATGA
GAACAA--

>Pteridomonas_danica_YPF1301_18SrRNA
TCTAAGTATAAACGCTATACTGTGAAACTGCGAGGCTCATAAACCAGTTATAGTT
TATTTGATATTCCCCTACTACTTGGATACCCGTAGTAATTCTAGAGCTAATACAT
GCGTCAAGCGACATATGTCGTGTCATTATTAGATAGAAACCAATCTTCGGGGAA
TTGTGGTGAGTCATGATAATTAGCGGATCGCACGGCTTATGCTGGCGATGGATC
ATTCAATTCTGCCCTATCAGCTTGGATGGTAGGGTATTGGCCTACCATGGCTTT
AACGGGTAACGGAGAATAGGGTTCGATTCCGGAGGGAGCCTGAGAGACGGCTA
CCACATCCAAGGAAGGCAGCAGGCGCGTAAATTACCCAATCCTGACTCAGGGAG
GTAGTGACAAAAAATAACTTAGTCGAGCCGTTTTGGTTTGACTTTAGGAATGAG
AACAATTTAAATCCCTTATCGAGGATCAATTGGAGGGCAAGTCTGGTGCCAGCA
GCCGCGGTAATTCCAGCTCCAATAGCGTATATTAATGTTGTTGCAGTTAAAAAG
CTCGTAGTTGGATTTCTGGTCAAACACCGGCCGGCCGCAGGGTGCTCGGTATTA
CGCCTGTCGTTTCTGTGAACAAATTAGAGTGTTCAAAGCAAGGTTTGAATACAT
TAGCATGGAATAATAGATAGGACTTGTGGTATTTTGTTGGTTGCACGAAAGTAA
TGATTAATAGGACAGTGGGGGTTTCGTATTCAATTGTCAGAGGTGAAATTCTTG
GATTTATGGACGAACTACTGCGAAAGCATTTACCAAGGATGTTTTCATTAATCAA
GAACGAAAGTTAGGGGACGAAGAAGATTAGATACCTTCGTATCTAACCATAAAC
CATGCCGACTCGGGATTGGCGGTCGGACTCCGCAGCACCGTATGAGAAATCAAA
GTCTTTGGGTTCCGGGGGGAGTATGGTCGCAAGGCTGAAACTTAAAGAAATTG
ACGGAAGGGCACCACCAGGAGTGGAGCCTGCGGCTTAATTTGACTCAACACGGG
GAAACTACCAGGTCCAGACATAGTAAGGATGACAGATTGAGAGCTCTTTCTTGA
TTCTTGGGTGGTGGTGCATGGCCGTTCTTAGTTGGTGGAGTGATTTGTCTGGT
TAATTCCGTAACGAACGAGACCCCCGCCTGCTAAATAGTCATAATGCTTCGCAT
GGTCGCTTCTTAGAGGGACTGTATGAGACTATCATACGGAAGTTGGGGGCAATA
ACGGTCTGTGATGCCCTTAGATGTTCTGGGCCGCACGCGCGCTACACTGATCGT
GCAACAAGTTTCCTTGGCCGGGAGGCCTGGGAATCTGAACGCGATCGTGAAGG
GATAGATTATTGCAATTATTAATCTTGAACGAGGAATTCCTAGTAACGCAAGTCA
TCAGCTTGCATGATTACTCCCGCCCTTGTACACACCGCCCGTCGCCCTACCATC
GAAGGTAATGATGAGCCGCAGACATCTGATTTTGCC
>Pteridomonas_danica_PT_SRR1296725
TCTAAGTATAAACGCTATACTGTGAAACTGCGAGGCTCATAAACCAGTTATAGTT
TATTTGATATTCCCCTACTACTTGGATACCCGTAGTAATTCTAGAGCTAATACAT
GCGTCAAGCGACATATGTCGTGTCATTATTAGATAGAAACCAATCTTCGGGGAA
TTGTGGTGAGTCATGATAATTAGCGGATCGCATGGCTTATGCCGGCGATGGATC

ATTCAATTCTGCCCTATCAGCTTGGATGGTAGGGTATTGGCCTACCATGGCTTT
AACGGGTAACGGAGAATAGGGTTCGATTCCGGAGGGAGCCTGAGAGACGGCTA
CCACATCCAAGGAAGGCAGCAGGCGCGTAAATTACCCAATCCTGACTCAGGGAG
GTAGTGACAAAAAATAACTTAGTCGAGCCGTTTTGGTTTGACTTTAGGAATGAG
AACAATTTAAATCCCTTATCGAGGATCAATTGGAGGGCAAGTCTGGTGCCAGCA
GCCGCGGTAATTCCAGCTCCAATAGCGTATATTAATGTTGTTGCAGTTAAAAG
CTCGTAGTTGGATTTCTGGTCGGACGTCGGCCGGCCGCAGGGTGCTCGGTATT
ACGCCTGTCGTTTCTGTGAACAAATTAGAGTGTTCAAAGCAAGGTTTGAATACA
TTAGCATGGAATAATAGATAGGACTTGTGGTATTTTGTTGGTTGCACGAAAGTA
ATGATTAATAGGACAGTGGGGGTTTCGTATTCAATTGTCAGAGGTGAAATTCTT
GGATTTATGGACGAACTACTGCGAAAGCATTTACCAAGGATGTTTTCATTAATC
AAGAACGAAAGTTAGGGGACGAAGAAGATTAGATACCTTCGTATCTAACCATAAA
CCATGCCGACTCGGGATTGGCGGTCGGACTCCGCAGCACCGTATGAGAAATCAA
AGTCTTTGGGTTCCGGGGGGAGTATGGTCGCAAGGCTGAAACTTAAAGAAATT
GACGGAAGGGCACCACCAGGAGTGGAGCCTGCGGCTTAATTTGACCCAACACGG
GGAAACTACCAGGTCCAGACATAGTAAGGATGACAGATTGAGAGCTCTTTCTTG
ATTCTTGGGTGGTGGTGCATGGCCGTTCTTAGTTGGTGGAGTGATTTGTCTGG
TTAATTCCGTAACGAACGAGACCCCCGCCTGCTAAATAGTCATAATGCTTCGCAT
GGTCGCTTCTTAGAGGGACTGTATGAGACTATCATACGGAAGTTGGGGGCAATA
ACGGTCTGTGATGCCCTTAGATGTTCTGGGCCGCACGCGCGCTACACTGATCGT
GCAACAAGTTTCCTTGGCCGAGAGGCCTGGGAATCTGAACGCGATCGTGAAGG
GATAGATTATTGCAATTATTAATCTTGAACGAGGAATTCCTAGTAACGCAAGTCA
TCAGCTTGCATGATTACTCCCGCCCTTGTACACACCGCCCGTCGCCCTACCATC
GAAGGTAATGATGAGCCGCAGACCACTGATTTTGCC
>Pteridomonas_danica_L37204
TCTAAGTATAAACGCTATACTGTGAAACTGCGAGGCTCATAAACCAGTTATAGTT
TATTTGATATTCCCCTACTACTTGGATACCCGTAGTAATTCTAGAGCTAATACAT
GCGTCAAGCGACATATGTCGTGTCATTATTAGATAGAAACCAATCTTCGGGGAA
TTGTGGTGAGTCATGATAATTAGCGGATCGCATGGCCTGTGCCGGCGATGGAT
CATTCAATTCTGCCCTATCAGCCTGGATGGTAGGGTATTGGCCTACCATGGCTT
TAACGGGTAACGGAGAATAGGGTTCGATTCCGGAGGGAGCCTGAGAGACGGCT
ACCACATCCAAGGAAGGCAGCAGGCGCGTAAATTACCCAATCCTGACTCAGGGA
GGTAGTGACAAAAAATAACTTAGTCGAGCCGTTTTGGTTTGACTTTAGGAATGA
GAACAATTTAAATCCCTTATCGAGGATCAATTGGAGGGCAAGTCTGGTGCCAGC
AGCCGCGGTAATTCCAGCTCCAATAGCGTATATTAATGTTGTTGCAGTTAAAAA
GCTCGTAGTTGGATTTCTGGTCGAACGCCGGCCGGCCGCAGGGTGCTCGGTAT

TGAGCCTGTCGTTTCTGTGAACAAATTAGAGTGTTCAAAGCAAGGTTTGAATAC
ATTAGCATGGAATAATAGATAGGACTTGTGGTATTTTGTTGGTTGCACGAAAGT
AATGATTAATAGGACAGTGGGGGTTTCGTATTCAATTGTCAGAGGTGAAATTCT
TGGATTTATGGACGAAGTACTGCGAAAGCATTTACCAAGGATGTTTTCATTAAT
CAAGAACGAAAGTTAGGGGACGAAGAAGATTAGATACCTTCGTATCTAACCATA
AACCATGCCGACTCGGGATTGGCGGTCGGACTCCGCAGCACCGTATGAGAAATC
AAAGTCTTTGGGTTCCGGGGGGAGTATGGTCGCAAGGCTGAAACTTAAAGAAAT
TGACGGAAGGGCACCACCAGGAGTGGAGCCTGCGGCTTAATTTGACTCAACACG
GGGAAACTACCAGGTCCAGACATAGTAAGGATGACAGATTGAGAGCTCTTTCTT
GATTCTTGGGTGGTGGTGCATGGCCGTTCTTAGTTGGTGGAGTGATTTGTCTG
GTTAATTCCGTAACGAACGAGACCCCCGCCTGCTAAATAGTCATAATGCTTCGC
ATGGTCGCTTCTTAGAGGGACTGTATGAGACTATCATACGGAAGTTGGGGGCAA
TAACGGTCTGTGATGCCCTTAGATGTCCTGGGCCGCACGCGCGCTACACTGATC
GTGCAACAAGTATCCTTGGCCGGGAGGCCTGGGAATCTGAACGCGATCGTGAA
GGGATAGATTATTGCAATTATTAATCTTGAACGAGGAATTCCTAGTAACGCAAG
TCATCAGCTTGCATGATTACTCCCGCCCTTGTACACACCGCCCGTCGCCCTACCA
TCGAAGGTAATGATGAGCCCCAGACCGCTGATTTAGCC
>Pteridomonas_danica_AB081640
TCTAAGTATAAACGCTATACTGTGAAACTGCGAGGCTCATAAACCAGTTATAGTT
TATTTGATATTCCCCTACTACTTGGATACCCGTAGTAATTCTAGAGCTAATACAT
GCGTCAAGCGACATATGTCGTGTCATTATTAGATAGAAACCAATCTTCGGGGAA
TTGTGGTGAGTCATGATAATTAGCGGATCGCATGGCCTGTGCCGGCGATGGAT
CATTCAATTCTGCCCTATCAGCTTGGATGGTAGGGTATTGGCCTACCATGGCTT
TAACGGGTAACGGAGAATAGGGTTCGATTCCGGAGGGAGCCTGAGAGACGGCT
ACCACATCCAAGGAAGGCAGCAGGCGCGTAAATTACCCAATCCTGACTCAGGGA
GGTAGTGACAAAAAATAACTTAGTCGAGCCGTTTTGGTTTGACTTTAGGAATGA
GAACAATTTAAATCCCTTATCGAGGATCAATTGGAGGGCAAGTCTGGTGCCAGC
AGCCGCGGTAATTCCAGCTCCAATAGCGTATATTAATGTTGTTGCAGTTAAAAA
GCTCGTAGTTGGATTTCTGGTCGGACGCCGGCCGGCCGCAGGGTGCTCGGTAT
TGAGCCTGTCGTTTCTGTGAACAAATTAGAGTGTTCAAAGCAAGGTTTGAATAC
ATTAGCATGGAATAATAGATAGGACTTGTGGTATTTTGTTGGTTGCACGAAAGT
AATGATTAATAGGACAGTGGGGGTTTCGTATTCAATTGTCAGAGGTGAAATTCT
TGGATTTATGGACGAACTACTGCGAAAGCATTTACCAAGGATGTTTTCATTAAT
CAAGAACGAAAGTTAGGGGACGAAGAAGATTAGATACCTTCGTATCTAACCATA
AACCATGCCGACTCGGGATTGGCGGTCGGACTCCGCAGCACCGTATGAGAAATC
AAAGTCTTTGGGTTCCGGGGGGAGTATGGTCGCAAGGCTGAAACTTAAAGAAAT

TGACGGAAGGGCACCACCAGGAGTGGAGCCTGCGGCTTAATTTGACTCAACACG
GGGAAACTACCAGGTCCAGACATAGTAAGGATGACAGATTGAGAGCTCTTTCTT
GATTCTTGGGTGGTGGTGCATGGCCGTTCTTAGTTGGTGGAGTGATTTGTCTG
GTTAATTCCGTAACGAACGAGACCCCCGCCTGCTAAATAGTCATAATGCTTCGC
ATGGTCGCTTCTTAGAGGGACTGTATGAGACTATCATACGGAAGTAGGGGGCAA
TAACGGTCTGTGATGCCCTTAGATGTCCTGGGCCGCACGCGCGCTACACTGATC
GTGCAACAAGTATCCTTGGCCGGGAGGCCTGGGTATCTGAACGCGATCGTGAA
GGGATAGATTATTGCAATTATTAATCTTGAACGAGGAATTCCTAGTAACGCTAG
TCATCAGCTTGCATGATTACTCCCGCCCTTGTACACACCGCCCGTCGCCCTACCA
TCGAAGGTAATGATGAGCCGCAGACCGCTGATTTAGCC
>Pterido_NY_18S_2_reads_from_NODE_assembled_using_Geneious
TCTAAGTATAAACGCTATACTGTGAAACTGCGAGGCTCATAAACCAGTTATAGTT
TATTTGATATTCCCCTACTACTTGGATACCCGTAGTAATTCTAGAGCTAATACAT
GCGTCAAGCGACATATGTCGTGTCATTATTAGATAGAAACCAATCTTCGGGGAA
TTGTGGTGAGTCATGATAATTAGCGGATCGCATGGCCTGTGCCGGCGATGGAT
CATTCAATTCTGCCCTATCAGCTTGGATGGTAGGGTATTGGCCTACCATGGCTT
TAACGGGTAACGGAGAATAGGGTTCGATTCCGGAGGGAGCCTGAGAGACGGCT
ACCACATCCAAGGAAGGCAGCAGGCGCGTAAATTACCCAATCCTGACTCAGGGA
GGTAGTGACAAAAAATAACTTAGTCGAGCCGTTTTGGTTTGACTTTAGGAATGA
GAACAATTTAAATCCCTTATCGAGGATCAATTGGAGGGCAAGTCTGGTGCCAGC
AGCCGCGGTAATTCCAGCTCCAATAGCGTATATTAATGTTGTTGCAGTTAAAAA
GCTCGTAGTTGGATTTCTGGTCGAACGCCGGCCGGCCGCAGGGTGCTCGGTAT
TACGCCTGTCGTTTCTGTGAACAAATTAGAGTGTTCAAAGCAAGGTTTGAATAC
ATTAGCATGGAATAATAGATAGGACTTGTGGTATTTTGTTGGTTGCACGAAAGT
AATGATTAATAGGACAGTGGGGGTTTCGTATTCAATTGTCAGAGGTGAAATTCT
TGGATTTATGGACGAACTACTGCGAAAGCATTTACCAAGGATGTTTTCATTAAT
CAAGAACGAAAGTTAGGGGACGAAGAAGATTAGATACCTTCGTATCTAACCATA
AACCATGCCGACTCGGGATTGGCGGTCGGACTCCGCAGCACCGTATGAGAAATC
AAAGTCTTTGGGTTCCGGGGGGAGTATGGTCGCAAGGCTGAAACTTAAAGAAAT
TGACGGAAGGGCACCACCAGGAGTGGAGCCTGCGGCTTAATTTGACTCAACACG
GGGAAACTACCAGGTCCAGACATAGTAAGGATGACAGATTGAGAGCTCTTTCTT
GATTCTTGGGTGGTGGTGCATGGCCGTTCTTAGTTGGTGGAGTGATTTGTCTG
GTTAATTCCGTAACGAACGAGACCCCCGCCTGCTAAATAGTCATAATGCTTCGC
ATGGTCGCTTCTTAGAGGGACTGTATGAGACTATCATACGGAAGTTGGGGGCAA
TAACGGTCTGTGATGCCCTTAGATGTTCTGGGCCGCACGCGCGCTACACTGATC
GTGCAACAAGTATCCTTGGCCGGGAGGCCTGGGAATCTGAACGCGATCGTGAA

GGGATAGATTATTGCAATTATTAATCTTGAACGAGGAATTCCTAGTAACGCAAG
TCATCAGCTTGCATGATTACTCCCGCCCTTGTACACACCGCCCGTCGCCCTACCA
TCGAAGGTAATGATGAGCCGCAGACCGCTGATTTAGCC
>Pseudopedinella_sp._culture_CCMP3052_HQ710562
TCTAAGTATAAACGCTATACTGTGAAACTGCGAGGCTCATAAACCAGTTATAGTT
TATTTGATATTCCCCTACTACTCGGATAACCGTAGTAATTCTAGAGCTAATACGT
GCGTAAAGCGGCATATGCCGTGTCATTATTAGATAGAAACCAATCCTTAGTATT
TTGTGGTGAGTCATGATAATTAGCGGAGCGCATGGACTGTTCCGGCGCTGGAT
CATTCAATTCTGCCCTATCAGCTTGGATGGTAGGGTATTGGCCTACCATGGCTT
TAACGGGTAACGGAGAATAGGGTTCGATTCCGGAGGGAGCCTGAGAGACGGCT
ACCACATCCAAGGAAGGCAGCAGGCGCGTAAATTACCCAATCCTGACTCAGGGA
GGTAGTGACAAAAAATAACTTAGTCGGGCCTTTTGGTCTGACTTTAGGAATGA
GAACAATTTAAATCCCTTATCGAGGATCAATTGGAGGGCAAGTCTGGTGCCAGC
AGCCGCGGTAATTCCAGCTCCAATAGCGTATATTAATGTTGTTGCAGTTAAAAA
GCTCGTAGTTGGATTTCTGGTCGGGCGTTGGCCGGCCGCAGGGTTCACGGTAT
TAAGCCTGTCGTTTCTGTGAACAAATTAGAGTGTTCAAAGCAAGGTTTGAATAC
ATTAGCATGGAATAATAGATAGGACTTGTGGTATTTTGTTGGTTGCACGAAAGT
AATGATTAATAGGACAGTGGGGGTTTCGTATTCAATTGTCAGAGGTGAAATTCT
TGGATTTATGGACGAACTACTGCGAAAGCATTTACCAAGGATGTTTTCATTAAT
CAAGAACGAAAGTTAGGGGACGAAGAAGATTAGATACCTTCGTATCTAACCTTA
AACTATGCCGACTCGGGATTGGAGGTCGGACTCCTCAGCACCGTATGAGAAATC
AAAGTCTTTGGGTTCCGGGGGGAGTATGGTCGCAAGGCTGAAACTTAAAGAAAT
TGACGGAAGGGCACCACCAGGAGTGGAGCCTGCGGCTTAATTTGACTCAACACG
GGGAAACTACCAGGTCCAGACATAGTAAGGATGACAGATTGAGAGCTCTTTCTT
GATTCTTGGGTGGTGGTGCATGGCCGTTCTTAGTTGGTGGAGTGATTTGTCTG
GTTAATTCCGTAACGAACGAGACCCCCGCCTGCTAAATAGCCGGAATGCTCTGC
ACGGTCGCTTCTTAGAGGGACTTTGTGTGACTAACACAATGAAGTTGGGGGCAA
TAACGGTCTGTGATGCCCTTAGATGTTCTGGGCCGCACGCGCGCTACACTGATC
ATGCAACGAGTTTCCTTGGCCGAGAGGCCTGGGAATCTGAACGTGATCGTGAAG
GGATTGATTATTGCAATTATTAATCATGAACGAGGAATTCCTAGTAACGCAAGTC
ATCAGCTTGCATGATTACTCCCGCCCTTGTACACACCGCCCGTCGCCCTACCATC
GAAGGTAATGATGAGCCCCAGACAGTTGTTGCATCC
>Pseudopedinella_elastica_U14387
TCTAAGTATAAACGCTATACTGTGAAACTGCGAGGCTCATAAACCAGTTATAGTT
TATTTGATATTCCCCTACTACTCGGATACCCGTAGTAATTCTAGAGCTAATACGT
GCGTCAAACGACATTTGTCGTGTCATTATTAGATAGAAACCAATCTTCGGGGTA

TTGTGGTGAGTCATGATAATTAGCGGATCGCATGGTCTGTACCGGCGATGGATC
ATTCAATTCTGCCCTATCAGCTTGGATGGTAGGGTATTGGCCTACCATGGCTTT
AACGGGTAACGGAGAATAGGGTTCGATTCCGGAGGGAGCCTGAGAGACGGCTA
CCACATCCAAGGAAGGCAGCAGGCGCGTAAATTACCCAATCCTGACACAGGGAG
GTAGTGACAAAAAATAACTTAGTCGGGCCGTTTTGGTCTGACTTTAGGAATGAG
AACAATTTAAATCCCTTATCGAGGATCAATTGGAGGGCAAGTCTGGTGCCAGCA
GCCGCGGTAATTCCAGCTCCAATAGCGTATATTAATGTTGTTGCAGTTAAAAAG
CTCGTAGTTGGATTTCTGGATGAGCGGCGGCCTTGCGCACGTTGCGCGGCATT
GAGCCTGTCGTTTCTGTGAACAAATTAGAGTGTTCAAAGCAAGGTTTGAATACA
TTAGCATGGAATAATAGATAGGACTTGTGGTATTTTGTTGGTTGCACGAAAGTA
ATGATTAATAGGACAGTGGGGGTTTCGTATTCAATTGTCAGAGGTGAAATTCTT
GGATTTATGGACGAACTACTGCGAAAGCATTTACCAAGGATGTTTTCATTAATC
AAGAACGAAAGTTAGGGGACGAAGAAGATTAGATACCTTCGTATCTAACCATAAA
CTATGCCGACTCGGGATTGGCGGTCGGACTCCGCAGCACCGTATGAGAAATCAA
AGTCTTTGGGTTCCGGGGGGAGTATGGTCGCAAGGCTGAAACTTAAAGAAATT
GACGGAAGGGCACCACCAGGAGTGGAGCCTGCGGCTTAATTTGACTCAACACGG
GGAAACTACCAGGTCCAGACATAGTAAGGATGACAGATTGAGAGCTCTTTCTTG
ATTCTTGGGTGGTGGTGCATGGCCGTTCTTAGTTGGTGGAGTGATTTGTCTGG
TTAATTCCGTAACGAACGAGACCCCCGCCTGCTAAATAGTTGGAATGCTTCGCA
TGCTCGCTTCTTAGAGGGACTTCGTGTGACTAACACGAGGAAGTTGGGGGCAAT
AACGGTCTGTGATGCCCTTAGATGTTCTGGGCCGCACGCGCGCTACACTGATCA
TGCAACGAGTTTCCTTGGCCGGGAGGCCTGGGAATCTGAACGTGATCGTGAAG
GGATAGATTATTGCAATTATTAATCTTGAACGAGGAATTCCTAGTAACGCAAGTC
AACAGCTTGCATGATTACTCCCGCCCTTGTACACACCGCCCGTCGCCCTACCATC
GAAGGTAATGATGAGCCCCAGACAGTCGATTGGGCC
>Pseudochattonella_verruculosa_strain_NIES_670_AM075625
TCTAAGTATAAACGCTATACTGTGAAACTGCGAGGCTCATAAACCAGTTATAGTT
TATTTGATATTCCCCTACTACTCGGATACCCGTAGTAATTCTAGAGCTAATACGT
GCACCAACACCCGACGAAGTGGTCATTATTAGATAGAAGCCAATCGCAAGTGTA
TTGTGGTGAGTCATGATAATGTGCGGACTGCACGGCCTGTGCCGGCGGTGGAT
CATTCAATTCTGCCCTATCAGCTTGGATGGTAGTGTATTGGACTACCATGGCTT
TAACGGGTAACGGAGAATGGGGTTCGATTCCGGAGGGAGCCTGAGAGACGGCT
ACCACATCCAAGGAAGGCAGCAGGCGCGCAAATTACCCAATCCTGACACAGGGA
GGTAGTGACAAAAAATAACAATGCCGGGCGTTTTCCGTCTGGCAATTGGAATGA
GAGCGATTTAAATCCCCCATCGAGGATCAATTGGAGGGCAAGTCTGGTGCCAGC
AGCCGCGGTAATTCCAGCTCCAATAGCGTATATTAAAGTTGTTGCAGTTAAAAA

GCTCGTAGTTGGATTTCTGGCGCGCCGCCGGTCGGTCGAAGGGTCGTCGGCAT
TGAGCTCGTCGTTTCTGTGAACAAATTAGAGTGTTCAAAGCAGGCTTTGAATAC
ATTAGCATGGAATAATAGATAGGACTTGTGGTATTTTGTTGGTTGCACGGAAGT
AATGATTAATAGGGCGGTGGGGGTTTCGTATTCAATTGTCAGAGGTGAAATTCT
TGGATTTATGGACGAACTACTGCGAAAGCATTTACCAAGGATGTTTTCATTAAT
CAAGAACGAAAGTTAGGGGACGAAGAAGATTAGATACCTTCGTATTTAACCATA
AACCATGCCGACTCGGGATGGGCGGTCGGGCTCCGCCGCACCGTATGAGAAAT
CAAAGTCTTTGGGTTCCGGGGGGAGTATGGTCGCAAGGCTGAAACTTAAAGAAA
TTGACGGAAGGGCACCACCAGGAGTGGAGCCTGCGGCTTAATTTGACTCAACAC
GGGGAAACTACCAGGTCCAGACATAGTAAGGATGACAGATTGAGAGCTCTTTCT
TGATTCTTGGGTGGTGGTGCATGGCCGTTCTTAGTTGGTGGAGTGATTTGTCT
GGTTAATTCCGTAACGAACGAGACCCCCGCCTGCTAAATAGTCGGAATGCTTCG
CATGCTTGCTTCTTAGAGGGACTTTCGGTGACTAACCGAAGGAAGTTGGGGGCA
ATAACGGTCTGTGATGCCCTTAGATGTCCTGGGCTGCACGCGCGCTACACTGAC
CATGCAACGAGTCTCCTTGGCTGGAAGGCCTGGGAATCTGAACGTGGTCGTGAA
GGGATAGATTATTGCAATTATTAATCTTGAACGAGGAATTCCTAGTAACGCAAG
TCATCAGCTTGCATGATTACTCCCGCCCTTGTACACACCGCCCGTCGCCCTACCA
TTGAATGGTTCGGTGAGGTCCAGATCGTGGCTTAGACC
>Pseudochattonella_farcimen_strain_UIO114_AM850220
TCTAAGTATAAACGCTATACTGTGAAACTGCGAGGCTCATAAACCAGTTATAGTT
TATTTGATATTCCCCTACTACTCGGATACCCGTAGTAATTCTAGAGCTAATACGT
GCACCAACACCCGACGAAGTGGTCATTATTAGATAGAAGCCAATCGCAAGTGTA
TTGTGGTGAGTCATGATAATGTGCGGACTGCACGGC-
TACGCCGGCGGTGGATCATTCAATTCTGCCCTATCAGCTTGGATGGTAGGGTAT
TGGCCTACCATGGCTTTAACGGGTAACGGAGAATGGGGTTCGATTCCGGAGGG
AGCCTGAGAGACGGCTACCACATCCAAGGAAGGCAGCAGGCGCGCAAATTACCC
AATCCTGACACAGGGAGGTAGTGACAAAAAATAACAATGCCGGGCGTTTTCCGT
CTGGCAATTGGAATGAGAGCGATTTAAATCCCCCATCGAGGATCAATTGGAGGG
CAAGTCTGGTGCCAGCAGCCGCGGTAATTCCAGCTCCAATAGCGTATATTAAAG
TTGTTGCAGTTAAAAAGCTCGTAGTTGGATTTCTGGCGCGCCGCCGGTCGGTC
GAAGGGTCGCCGGCATTGAGCTCGTCGTTTCTGTGAACAAATTAGAGTGTTCAA
AGCAGGCTTTGAATACATTAGCATGGAATAATAGATAGGACTTGTGGTATTTTG
TTGGTTGCACGGAAGTAATGATTAATAGGGCGGTGGGGGTTTCGTATTCAATT
GTCAGAGGTGAAATTCTTGGATTTATGGACGAACTACTGCGAAAGCATTTACCA
AGGATGTTTTCATTAATCAAGAACGAAAGTTAGGGGACGAAGAAGATTAGATAC
CTTCGTATTTAACCATAAACCATGCCGACTCGGGATGGGCGGTCGGGCTCCGCC

GCACCGTATGAGAAATCAAAGTCTTTGGGTTCCGGGGGGAGTATGGTCGCAAG
GCTGAAACTTAAAGAAATTGACGGAAGGGCACCACCAGGAGTGGAGCCTGCGGC
TTAATTTGACTCAACACGGGGAAACTACCAGGTCCAGACATAGTAAGGATGACA
GATTGAGAGCTCTTTCTTGATTCTTGGGTGGTGGTGCATGGCCGTTCTTAGTT
GGTGGAGTGATTTGTCTGGTTAATTCCGTAACGAACGAGACCCCCGCCTGCTAA
ATAGTCGGAATGCTTCGCATGCTCGCTTCTTAGAGGGACTTTCGGTGACTAACC
GAAGGAAGTTGGGGGCAATAACGGTCTGTGATGCCCTTAGATGTCCTGGGCTG
CACGCGCGCTACACTGACCATGCAACGAGTCTCCTTGGCTGGAAGGCCTGGGAA
TCTGAACGTGGTCGTGAAGGGATAGATTATTGCAATTATTAATCTTGAACGAGG
AATTCCTAGTAACGCAAGTCATCAGCTTGCATGATTACTCCCGCCCTTGTACACA
CCGCCCGTCGCCCTACCATTGAATGGTTCGGTGAGGTCCAGATCGTGGCTTAGA
CC
>Pedinella_sp._AB081517
TCNAAGTATAAACACTNTACTGTGAAACTGCGAGGCTCATAAACCAGTTATAGTT
TATTTGATATTCCCCTACTACTCGGATACCCGTAGTAATTCTAGAGCTAATACGT
GCGTCAAACAGCATTTGCTGTGTCATTATTAGATAGAAACCAATTCTCGGGGTA
TTGTGGTGAGTCATGATAATTAGCGGATCGCATGGCTTACGCCGGCGATGGAT
CATTCAATTCTGCCCTATCAGCTTGGATGGTAGGGTATTGGCCTACCATGGCTT
TAACGGGTAACGGAGAATAGGGTTCGATTCCGGAGGGAGCCTGAGAGACGGCT
ACCACATCCAAGGAAGGCAGCAGGCGCGTAAATTACCCAATCCTGACACAGGGA
GGTAGTGACAAAAAATAACTTAGTCGGGCCGTTTTGGTCTGACTTTAGGAATGA
GAACAATTTAAATCCCTTATCGAGGATCAATTGGAGGGCAAGTCTGGTGCCAGC
AGCCGCGGTAATTCCAGCTCCAATAGCGTATATTAATGTTGTTGCAGTTAAAAA
GCTCGTAGTTGGATTTCTGGTCGAACGCTGTCCGGCCGCAGGGTGCACGGTAT
TGAGCCTGTCGTTTCTGTGAACAAATTAGAGTGTTCAAAGCAAGGTTTGAATAC
ATTAGCATGGAATAATAGATAGGACTTGTGGTATTTTGTTGGTTGCACGAAAGT
AATGATTAATAGGACAGTGGGGGTTTCGTATTCAATTGTCAGAGGTGAAATTCT
TGGATTTATGGACGAACTACTGCGAAAGCATTTACCAAGGATGTTTTCATTAAT
CAAGAACGAAAGTTAGGGGACGAAGAAGATTAGATACCTTCGTATCTAACCATA
AACTATGCCGACTCGGGATTGGCGGTCGGACTCCGCAGCACCGTATGAGAAATC
AAAGTCTTTGGGTTCCGGGGGGAGTATGGTCGCAAGGCTGAAACTTAAAGAAAT
TGACGGAAGGGCACCACCAGGAGTGGAGCCTGCGGCTTAATTTGACTCAACACG
GGGAAACTACCAGGTCCAGACATAGTAAGGATGACAGATTGAGAGCTCTTTCTT
GATTCTTGGGTGGTGGTGCATGGCCGTTCTTAGTTGGTGGAGTGATTTGTCTG
GTTAATTCCGTAACGAACGAGACCCCCGCCTGCTAAATAGCGCGTATGCTT-
GCAATCTCGCTTCTTAGAGGGACTTTGTGTGACTAACACAAGGAAGTTGGGGGC

AATAACGGTCTGTGATGCCCTTAGATGTTCTGGGCCGCACGCGCGCTACACTGA
TCATGCAACGAGTCTCCTTGGCCGGGAGGCCTGGGAATCTGAACGTGATCGTG
AAGGGATAGATTATTGCAATTATTAATCTTGAACGAGGAATTCCTAGTAACGCAA
GTCAACAGCTTGCATGATTACTCCCGCCCTTGTACACACCGCCCGTCGCCCTAC
CATCGAAGGTAATGATGAGCCCCAGACAGTTGTTTGGGTC
>LC189143.1_Pseudopedinella_elastica_NIES-3879
TCTAAGTATAAACGCTATACTGTGAAACTGCGAGGCTCATAAACCAGTTATAGTT
TATTTGATATTCCCCTACTACTCGGATACCCGTAGTAATTCTAGAGCTAATACGT
GCGTCAAACGACATTTGTCGTGTCATTATTAGATAGAAACCAATCTTCGGGGTA
TTGTGGTGAGTCATGATAATTAGCGGATCGCATGGTCTGTACCGGCGATGGATC
ATTCAATTCTGCCCTATCAGCTTGGATGGTAGGGTATTGGCCTACCATGGCTTT
AACGGGTAACGGAGAATAGGGTTCGATTCCGGAGGGAGCCTGAGAGACGGCTA
CCACATCCAAGGAAGGCAGCAGGCGCGTAAATTACCCAATCCTGACACAGGGAG
GTAGTGACAAAAAATAACTTAGTCGGGCCGTTTTGGTCTGACTTTAGGAATGAG
AACAATTTAAATCCCTTATCGAGGATCAATTGGAGGGCAAGTCTGGTGCCAGCA
GCCGCGGTAATTCCAGCTCCAATAGCGTATATTAATGTTGTTGCAGTTAAAAAG
CTCGTAGTTGGATTTCTGGATGAGCGGCGGCCTTGCGCACGTTGCGCGGCATT
ACGCCTGTCGTTTCTGTGAACAAATTAGAGTGTTCAAAGCAAGGTTTGAATACA
TTAGCATGGAATAATAGATAGGACTTGTGGTATTTTGTTGGTTGCACGAAAGTA
ATGATTAATAGGACAGTGGGGGTTTCGTATTCAATTGTCAGAGGTGAAATTCTT
GGATTTATGGACGAACTACTGCGAAAGCATTTACCAAGGATGTTTTCATTAATC
AAGAACGAAAGTTAGGGGACGAAGAAGATTAGATACCTTCGTATCTAACCATAAA
CTATGCCGACTCGGGATTGGCGGTCGGACTCCGCAGCACCGTATGAGAAATCAA
AGTCTTTGGGTTCCGGGGGGAGTATGGTCGCAAGGCTGAAACTTAAAGAAATT
GACGGAAGGGCACCACCAGGAGTGGAGCCTGCGGCTTAATTTGACTCAACACGG
GGAAACTACCAGGTCCAGACATAGTAAGGATGACAGATTGAGAGCTCTTTCTTG
ATTCTTGGGTGGTGGTGCATGGCCGTTCTTAGTTGGTGGAGTGATTTGTCTGG
TTAATTCCGTAACGAACGAGACCCCCGCCTGCTAAATAGTTGGAATGCTTCGCA
TGATCGCTTCTTAGAGGGACTTCGTGTGACTAACACGAGGAAGTTGGGGGCAAT
AACGGTCTGTGATGCCCTTAGATGTTCTGGGCCGCACGCGCGCTACACTGATCA
TGCAACGAGTTTCCTTGGCCGGGAGGCCTGGGAATCTGAACGTGATCGTGAAG
GGATAGATTATTGCAATTATTAATCTTGAACGAGGAATTCCTAGTAACGCAAGTC
AACAGCTTGCATGATTACTCCCGCCCTTGTACACACCGCCCGTCGCCCTACCATC
GAAGGTAATGATGAGCCCCAGACAGTCGATTGGCGC
>KT861168.1_Helicopedinella_sp._RCC2287

GCTCGTAGTTGGATTTCTGGTCGATCGCCGGCCGGCCGCAGGGTGCGCGGTAT
TACGCCTGTCGTTTCTGTGAACAAATTAGAGTGTTCAAAGCAAGGTTTGAATAC
ATTAGCATGGAATAATAGATAGGACTTGTGGTATTTTGTTGGTTGCACGAAAGT
AATGATTAATAGGACAGTGGGGGTTTCGTATTCAATTGTCAGAGGTGAAATTCT
TGGATTTATGGACGAACTACTGCGAAAGCATTTACCAAGGATGTTTTCATTAAT
CAAGAACGAAAGTTAGGGGACGAAGAAGATTAGATACCTTCGTATCTAACCATA
AACTATGCCGACTCGGGATTGGCGGTCGGGCTCCGCAGCACCGTATGAGAAATC
AAAGTCTTTGGGTTCCGGGGGGAGTATGGTCGCAAGGCTGAAACTTAAAGAAAT
TGACGGAAGGGCACCACCAGGAGTGGAGCCTGCGGCTTAATTTGACTCAACACG
GGGAAACTACCAGGTCCAGACATAGTAAGGATGACAGATTGAGAGCTCTTTCTT
GATTCTTGGGTGGTGGTGCATGGCCGTTCTTAGTTGGTGGAGTGATTTGTCTG
GTTAATTCCGTAACGAACGAGACCCCCGCCTGCTAAATAGTCGGAATGCTTTGC
ATGGGTGCTTCTTAGAGGGACTGTGTGAGACTATCACACGGAAGTTGGGGGCA
ATATCG--

>KT861167.1_Helicopedinella_sp._RCC2284

GCTCGTAGTTGGATTTCTGGTCGATCGCCGGCCGGCCGCAGGGTGCGCGGTAT
TACGCCTGTCGTTTCTGTGAACAAATTAGAGTGTTCAAAGCAAGGTTTGAATAC
ATTAGCATGGAATAATAGATAGGACTTGTGGTATTTTGTTGGTTGCACGAAAGT
AATGATTAATAGGACAGTGGGGGTTTCGTATTCAATTGTCAGAGGTGAAATTCT
TGGATTTATGGACGAACTACTGCGAAAGCATTTACCAAGGATGTTTTCATTAAT
CAAGAACGAAAGTTAGGGGACGAAGAAGATTAGATACCTTCGTATCTAACCATA
AACTATGCCGACTCGGGATTGGCGGTCGGGCTCCGCAGCACCGTATGAGAAATC
AAAGTCTTTGGGTTCCGGGGGGAGTATGGTCGCAAGGCTGAAACTTAAAGAAAT

TGACGGAAGGGCACCACCAGGAGTGGAGCCTGCGGCTTAATTTGACTCAACACG
GGGAAACTACCAGGTCCAGACATAGTAAGGATGACAGATTGAGAGCTCTTTCTT
GATTCTTGGGTGGTGGTGCATGGCCGTTCTTAGTTGGTGGAGTGATTTGTCTG
GTTAATTCCGTAACGAACGAGACCCCCGCCTGCTAAATAGTCGGAATGCTTTGC
ATGGGTGCTTCTTAGAGGGACTGTGTGAGACTATCACACGGAAGTTGGGGGCA
ATAACGGTCTGTGATGCCCTTAGATGTCCTGGGCCGCACGCGCGCTACACTGAT
CGTGCAACGAG---

>KT861105.1_Rhizochromulina_sp._RCC332
TCTAAGTATAAGCGTTATACTGTGAAACTGCGAGGATCATAAACCAGTTATAGTT
TATTTGATATTTCCCTACTACTCGGATACCCGTAGTAATTCTAGAGCTAATACGT
GCATAGAGCCCCATTTGGGGTGCAATTATTAGATAGAAACCAATCGCAAGGGTA
TTGTGGTGAGTCATGATAATAAGCGGATCGCATGACCCGAGTCGGCGACGGATC
ATTCAATTCTGCCCTATCAGCTTGGATGGTACGGTATTGGCCTACCATGGCTTT
AACGGGTAACGGAGAATAGGGTTCGATTCCGGAGGGAGCCTGAGAGACGGCTA
CCACATCCAAGGAAGGCAGCAGGCGCGTAAATTACCCAATCCTGACGCAGGGAG
GTAGTGACAAGAAATAACTTAGTCGGGCCCATACGGTCTGACTTTAGGAATGAG
AACAATTTAAATCCCTTATCGAGGATCAATTGGAGGGCAAGTCTGGTGCCAGCA
GCCGCGGTAATTCCAGCTCCAATAGCGTATATTAATGTTGTTGCAGTTAAAAAG
CTCGTAGTTGGATTTCCAATGGAGCGGCGGTCGGCCGCAGGGTGTCAGGCATT
AGTCCCGTCGTTTCTGTGAACAAATTAGAGTGTTCCAAGCAAGGCTTGAATACA
TTAGCATGGAATAATAGATAGGACTTGTGGTATTTTGTTGGTTGCACGAAAGTA
ATGATTAATAGGACAGTGGGGGTTTCGTATTCAATTGTCAGAGGTGAAATTCTT
GGATTTCTGGACGAACTACTGCGAAAGCATTTACCAAGGATGTTTTCATTAATC
AAGAACGAAAGTTAGGGGACGAAGAAGATTAGATACCTTCGTATCTAACCATAAA
CGATGCCGACTCGGGATTGGCGGTCGGACTCCGCAGCACCGTATGAGAAATCAA
AGTCTTTGGGTTCCGGGGGGAGTATGGTCGCAAGGCTGAAACTTAAAGAAATT
GACGGAAGGGCACCACCAGGAGTGGAGCCTGCGGCTTAATTTGACTCAACACGG
GGAAACTACCAGGTCCAGACATAGTAAGGATGACAGATTGAGAGCTCTTTCTTG
ATTCTTGGGTGGTGGTGCATGGCCGTTCTTAGTTGGTGGAGTGATTAGTCTGG
TTAATTCCGTAACGAACGAGACCCCCGCCTGCTAAATAGCTGGAATGCATCCCA
TGGAGGCTTCTTAGAGGGACTGTCAGTGACTAACTGACGGAAGTTGGGGGCAA
TAACGGTCTGTGATGCCCTTAGATGTCCTGGGCCGCACGCGCGCTACACTGATC
ATGCAACGAGTTTCCTTGGCTGGAAGGCCTGGGAATCTGAACGTGATCGTGAAG
GGATAGATTATTGCAATTATTAATCTTGAACGAGGAATTCCTAGTAACGCAAGTC

ATCAGCTTGCATGATTACTCCCGCCCTTGTACACACCGCCCGTCGCCCTACCATC
GAAGGTAATGATGAGCCCCAGATTGGAGCTTGGATC
>KT860987.1_Rhizochromulina_sp._RCC900
-------------------------AACTGCG-
GGCTCATAAACCAGTTATAGTTTATTTGATATTTCCCTACTACTCGGATACCCGT
AGTAATTCTAGAGCTAATACGTGCATAGAGCCCCATTTGGGGTGCAATTATTAG
ATAGAAACCAATCGCAAGGGTATTGTGGTGAGTCATGATAATAAGCGGATCGCA
TGACCTGAGTCGGCGACGGATCATTCAATTCTGCCCTATCAGCTTGGATGGTAC
GGTATTGGCCTACCATGGCTTTAACGGGTAACGGAGAATAGGGTTCGATTCCGG
AGGGAGCCTGAGAGACGGCTACCACATCCAAGGAAGGCAGCAGGCGCGTAAATT
ACCCAATCCTGACACAGGGAGGTAGTGACAAGAAATAACTTAGTCGGGCCCATA
CGGTCTGACTTTAGGAATGAGAACAATTTAAATCCCTTATCGAGGATCAATTGG
AGGGCAAGTCTGGTGCCAGCAGCCGCGGTAATTCCAGCTCCAATAGCGTATATT
AATGTTGTTGCAGTTAAAAAGCTCGTAGTTGGATTTCGAATGGAGCGGCGGTCG
GCCGCAGGGTGTCAGGCATTAGTCCCGTCGTTTCTGTGAACAAATTAGAGTGTT
CCAAGCAAGGCTTGAATACATTAGCATGGAATAATAGATAGGACTTGTGGTATT
TTGTTGGTTGCACGAAAGTAATGATTAATAGGACAGTGGGGGTTTCGTATTCAA
TTGTCAGAGGTGAAATTCTTGGATTTCTGGACGAACTACTGCGAAAGCATTTAC
CAAGGATGTTTTCATTAATCAAGAACGAAAGTTAGGGGACGAAGAAGATTAGAT
ACCTTCGTATCTAACCATAAACGATGCCGACTCGGGATTGGCGGTCGGACTCCG
CAGCACCGTATGAGAAATCAAAGTCTTTGGGTTCCGGGGGGAGTATGGTCGCAA
GGCTGAAACTTAAAGAAATTGACGGAAGGGCACCACCAGGAGTGGAGCCTGCGG
CTTAATTTGACTCAACACGGGGAAACTACCAGGTCCAGACATAGTAAGGATGAC
AGATTGAGAGCTCTTTCTTGATTCTTGGGTGGTGGTGCATGGCCGTTCTTAGTT
GGTGGAGTGATTTGTCTGGTTAATTCCGTAACGAACGAGACCCCCGCCTGCTAA
ATAGCTGGAATGCATCGCATGGAGGCTTCTTAGAGGGACTGTCAGTGACTAACT
GACGGAAGTTGGGGGCAATAACGGTCTGTGATGCCCTTAGATGTCCTGGGCCG
CACGCGCGCTACACTGATCATGCAACGAGTTTCCTTGGCTGGAAGGCCTGGGAA
TCTGAACGTGATCGTGAAGGGATAGATTATTGCAATTATTAATCTTGAACGAGG
AATTCCTAGTAACGCAAGTCATCAGCTTGCATGATTACTCCCGCCCTTGTACACA
CCGCCCGTCGCCCTACCATCGAAGGTAATGATGAGCCCCAGATTGGAGCTTGGA
TC
>KF422605.1_Rhizochromulina_cf._marina_CCMP1243
TCTAAGTATAAGCGTTATACTGTGAAACTGCGAGGCTCATAAACCAGTTATAGT
TTATTTGATATTTCCCTACTACTCGGATACCCGTAGTAATTCTAGAGCTAATACG
TGCATAGAGCCCCATTTGGGGTGCAATTATTAGATAGAAACCAATCGCAAGGGT

ATTGTGGTGAGTCATGATAATAAGCGGATCGCATGACCTGAGTCGGCGACGGAT
CATTCAATTCTGCCCTATCAGCTTGGATGGTACGGTATTGGCCTACCATGGCTT
TAACGGGTAACGGAGAATAGGGTTCGATTCCGGAGGGAGCCTGAGAGACGGCT
ACCACATCCAAGGAAGGCAGCAGGCGCGTAAATTACCCAATCCTGACACAGGGA
GGTAGTGACAAGAAATAACTTAGTCGGGCCCATACGGTCTGACTTTAGGAATGA
GAACAATTTAAATCCCTTATCGAGGATCAATTGGAGGGCAAGTCTGGTGCCAGC
AGCCGCGGTAATTCCAGCTCCAATAGCGTATATTAATGTTGTTGCAGTTAAAAA
GCTCGTAGTTGGATTTCGAATGGAGCGGCGGTCGGCCGCAGGGTGTCAGGCAT
TAGTCCCGTCGTTTCTGTGAACAAATTAGAGTGTTCCAAGCAAGGCTTGAATAC
ATTAGCATGGAATAATAGATAGGACTTGTGGTATTTTGTTGGTTGCACGAAAGT
AATGATTAATAGGACAGTGGGGGTTTCGTATTCAATTGTCAGAGGTGAAATTCT
TGGATTTCTGGACGAACTACTGCGAAAGCATTTACCAAGGATGTTTTCATTAAT
CAAGAACGAAAGTTAGGGGACGAAGAAGATTAGATACCTTCGTATCTAACCATA
AACGATGCCGACTCGGGATTGGCGGTCGGACTCCGCAGCACCGTATGAGAAATC
AAAGTCTTTGGGTTCCGGGGGGAGTATGGTCGCAAGGCTGAAACTTAAAGAAAT
TGACGGAAGGGCACCACCAGGAGTGGAGCCTGCGGCTTAATTTGACTCAACACG
GGGAAACTACCAGGTCCAGACATAGTAAGGATGACAGATTGAGAGCTCTTTCTT
GATTCTTGGGTGGTGGTGCATGGCCGTTCTTAGTTGGTGGAGTGATTTGTCTG
GTTAATTCCGTAACGAACGAGACCCCCGCCTGCTAAATAGCTGGAATGCATCGC
ATGGAGGCTTCTTAGAGGGACTGTCAGTGACTAACTGACGGAAGTTGGGGGCA
ATAACGGTCTGTGATGCCCTTAGATGTCCTGGGCCGCACGCGCGCTACACTGAT
CATGCAACGAGTTTCCTTGGCTGGAAGGCCTGGGAATCTGAACGTGATCGTGAA
GGGATAGATTATTGCAATTATTAATCTTGAACGAGGAATTCCTAGTAACGCAAG
TCATCAGCTTGCATGATTACTCCCGCCCTTGTACACACCGCCCGTCGCCCTACCA
TCGAAGGTAATGATGAGCCCCAGATTGGAGCTTGGATC
>HQ710563.1_Rhizochromulina_marina_voucher_CCAP_950/1

TCTTGGATTTCTGGACGAACTACTGCGAAAGCATTTACCAAGGATGTTTTCATT
AATCAAGAACGAAAGTTAGGGGACGAAGAAGATTAGATACCTTCGTATCTAACC

ATAAACGATGCCGACTCGGGATTGGCGGTCGGACTCCGCAGCACCGTATGAGAA
ATCAAAGTCTTTGGGTTCCGGGGGGAGTATGGTCGCAAGGCTGAAACTTAAAGA
AATTGACGGAAGGGCACCACCAGGAGTGGAGCCTGCGGCTTAATTTGACTCAAC
ACGGGGAAACTACCAGGTCCAGACATAGTAAGGATGACAGATTGAGAGCTCTTT
CTTGATTCTTGGGTGGTGGTGCATGGCCGTTCTTAGTTGGTGGAGTGATTTGT
CTGGTTAATTCCGTAACGAACGAGACCCCCGCCTGCTAAATAGCTGGAATGCAT
CGCATGATCGCTTCTTAGAGGGACTGTCAGTGACTAACTGACGGAAGTTGGGG
GCAATAACGGTCTGTGATGCCCTTAGATGTCCTGGGCCGCACGCGCGCTACACT
GATCATGCAGCGAGTCTCCTTGGCTGGAAGGCCTGGGAATCTGAACGTGATCG
TGAAGGGATAGATTATTGCAATTATTAATCTTGAACGAGGAATTCCTAGTAACG
CAAGTCATCAGCTTGCATGATTACTCCCGCCCTTGTACACACCGCCCGTCGCCC
TACCATCGAAGGTAATGATGAGCGCCAGATTGGAGCCAGGGCC
>KT861032.1_Apedinella_spinifera_strain_RCC665

TGCGGTATTACGCCTGTCGTTTCTGTGAACAAATTAGGGTGTTCAAAGCAAGGT
TTGAATACATTAGCATGGAATAATAGATAGGACTTGTGGTATTTTGTTGGTTGC
ACGAAAGTAATGATTAATAGGACAGTGGGGGTTTCGTATTCAATTGTCAGAGGT
GAAATTCTTGGATTTATGGACGAACTACTGCGAAAGCACTTACCAAGGATGTTT
TCATTAATCAAGAACGAAAGTTAGGGGACGAAAAAGATTAGATACCTTCGTATCT
AGCCATAAACGATGCCGACTCGGGATTGGCGGTCGGTCTCCGCAGCACCGTATG
AGAAATCAAAGTCTTTGGGTTCCGGGGGGAGTATGGTCGCAAGGCTGAAACTTA
AAGAAATTGACGGAAGGGCACCACCAGGAGTGGAGCCTGCGGCTTAATTTGACT
CAACACGGGGAAACTACCAGGTCCAGACATAGTGAGGATGACAGATTGAGAGCT
CTTTCTTGATTCTTGGGTGGTGGTGCATGGCCGTTCTTAGTTGGTGGAGTGAT
TTGTCTGGTTAATTCCGTAACGAACGA---

>HQ710562.1_Pseudopedinella_sp._CCMP3052
TCTAAGTATAAACGCTATACTGTGAAACTGCGAGGCTCATAAACCAGTTATAGTT
TATTTGATATTCCCCTACTACTCGGATAACCGTAGTAATTCTAGAGCTAATACGT

GCGTAAAGCGGCATATGCCGTGTCATTATTAGATAGAAACCAATCCTTGGTATT
TTGTGGTGAGTCATGATAATTAGCGGAGCGCATGGACTGTTCCGGCGCTGGAT
CATTCAATTCTGCCCTATCAGCTTGGATGGTAGGGTATTGGCCTACCATGGCTT
TAACGGGTAACGGAGAATAGGGTTCGATTCCGGAGGGAGCCTGAGAGACGGCT
ACCACATCCAAGGAAGGCAGCAGGCGCGTAAATTACCCAATCCTGACTCAGGGA
GGTAGTGACAAAAAATAACTTAGTCGGGCCTTTTTGGTCTGACTTTAGGAATGA
GAACAATTTAAATCCCTTATCGAGGATCAATTGGAGGGCAAGTCTGGTGCCAGC
AGCCGCGGTAATTCCAGCTCCAATAGCGTATATTAATGTTGTTGCAGTTAAAAA
GCTCGTAGTTGGATTTCTGGTCGGGCGTTGGCCGGCCGCAGGGTTCACGGTAT
TACGCCTGTCGTTTCTGTGAACAAATTAGAGTGTTCAAAGCAAGGTTTGAATAC
ATTAGCATGGAATAATAGATAGGACTTGTGGTATTTTGTTGGTTGCACGAAAGT
AATGATTAATAGGACAGTGGGGGTTTCGTATTCAATTGTCAGAGGTGAAATTCT
TGGATTTATGGACGAACTACTGCGAAAGCATTTACCAAGGATGTTTTCATTAAT
CAAGAACGAAAGTTAGGGGACGAAGAAGATTAGATACCTTCGTATCTAACCTTA
AACTATGCCGACTCGGGATTGGAGGTCGGACTCCTCAGCACCGTATGAGAAATC
AAAGTCTTTGGGTTCCGGGGGGAGTATGGTCGCAAGGCTGAAACTTAAAGAAAT
TGACGGAAGGGCACCACCAGGAGTGGAGCCTGCGGCTTAATTTGACTCAACACG
GGGAAACTACCAGGTCCAGACATAGTAAGGATGACAGATTGAGAGCTCTTTCTT
GATTCTTGGGTGGTGGTGCATGGCCGTTCTTAGTTGGTGGAGTGATTTGTCTG
GTTAATTCCGTAACGAACGAGACCCCCGCCTGCTAAATAGCCGGAATGCTCTGC
ACGATCGCTTCTTAGAGGGACTTTGTGTGACTAACACAATGAAGTTGGGGGCAA
TAACGGTCTGTGATGCCCTTAGATGTTCTGGGCCGCACGCGCGCTACACTGATC
ATGCAACGAGTTTCCTTGGCCGAGAGGCCTGGGAATCTGAACGTGATCGTGAAG
GGATTGATTATTGCAATTATTAATCATGAACGAGGAATTCCTAGTAACGCAAGTC
ATCAGCTTGCATGATTACTCCCGCCCTTGTACACACCGCCCGTCGCCCTACCATC
GAAGGTAATGATGAGCCCCAGACAGTTGTTGCAGTC
>HQ710561.1_Pseudopedinella_sp._CCMP1476
TCTAAGTATAAACACTATACTGTGAAACTGCGAGGCTCATAAACCAGTTATAGTT
TATTTGATATTCCCCTACTACTCGGATACCCGTAGTAATTCTAGAGCTAATACGT
GCGTCAAACAGCATTTGCTGTGTCATTATTAGATAGAAACCAATTCTCGGGGTA
TTGTGGTGAGTCATGATAATTAGCGGATCGCATGGCTTACGCCGGCGATGGAT
CATTCAATTCTGCCCTATCAGCTTGGATGGTAGGGTATTGGCCTACCATGGCTT
TAACGGGTAACGGAGAATAGGGTTCGATTCCGGAGGGAGCCTGAGAGACGGCT
ACCACATCCAAGGAAGGCAGCAGGCGCGTAAATTACCCAATCCTGACACAGGGA
GGTAGTGACAAAAAATAACTTAGTCGGGCCGTTTTGGTCTGACTTTAGGAATGA
GAACAATTTAAATCCCTTATCGAGGATCAATTGGAGGGCAAGTCTGGTGCCAGC

AGCCGCGGTAATTCCAGCTCCAATAGCGTATATTAATGTTGTTGCAGTTAAAAA
GCTCGTAGTTGGATTTCTGGTCGAACGCTGTCCGGCCGCAGGGTGCACGGTAT
TACGCCTGTCGTTTCTGTGAACAAATTAGAGTGTTCAAAGCAAGGTTTGAATAC
ATTAGCATGGAATAATAGATAGGACTTGTGGTATTTTGTTGGTTGCACGAAAGT
AATGATTAATAGGACAGTGGGGGTTTCGTATTCAATTGTCAGAGGTGAAATTCT
TGGATTTATGGACGAACTACTGCGAAAGCATTTACCAAGGATGTTTTCATTAAT
CAAGAACGAAAGTTAGGGGACGAAGAAGATTAGATACCTTCGTATCTAACCATA
AACTATGCCGACTCGGGATTGGCGGTCGGACTCCGCAGCACCGTATGAGAAATC
AAAGTCTTTGGGTTCCGGGGGGAGTATGGTCGCAAGGCTGAAACTTAAAGAAAT
TGACGGAAGGGCACCACCAGGAGTGGAGCCTGCGGCTTAATTTGACTCAACACG
GGGAAACTACCAGGTCCAGACATAGTAAGGATGACAGATTGAGAGCTCTTTCTT
GATTCTTGGGTGGTGGTGCATGGCCGTTCTTAGTTGGTGGAGTGATTTGTCTG
GTTAATTCCGTAACGAACGAGACCCCCGCCTGCTAAATAGCGCGTATGCTTCAT
ACGCTCGCTTCTTAGAGGGACTTTGTGTGACTAACACAAGGAAGTTGGGGGCAA
TAACGGTCTGTGATGCCCTTAGATGTTCTGGGCCGCACGCGCGCTACACTGATC
ATGCAACGAGTCTCCTTGGCCGGGAGGCCTGGGAATCTGAACGTGATCGTGAA
GGGATAGATTATTGCAATTATTAATCTTGAACGAGGAATTCCTAGTAACGCAAG
TCAACAGCTTGCATGATTACTCCCGCCCTTGTACACACCGCCCGTCGCCCTACCA
TCGAAGGTAATGATGAGCCCCAGACAGTTGTTTGGGTC
>HQ710560.1_Pseudopedinella_elastica_CCMP716

CTATACTGTGAAACTGCGAGGCTCATAAACCAGTTATAGTTTATTTGATATTCCC
CTACTACTCGGATACCCGTAGTAATTCTAGAGCTAATACGTGCGTCAAACGACAT
TTGTCGTGTCATTATTAGATAGAAACCAATCTTCGGGGTATTGTGGTGAGTCAT
GATAATTAGCGGATCGCATGGTCTGTACCGGCGATGGATCATTCAATTCTGCCC
TATCAGCTTGGATGGTAGGGTATTGGCCTACCATGGCTTTAACGGGTAACGGAG
AATAGGGTTCGATTCCGGAGGGAGCCTGAGAGACGGCTACCACATCCAAGGAAG
GCAGCAGGCGCGTAAATTACCCAATCCTGACACAGGGAGGTAGTGACAAAAAAT
AACTTAGTCGGGCCGTTTTGGTCTGACTTTAGGAATGAGAACAATTTAAATCCC
TTATCGAGGATCAATTGGAGGGCAAGTCTGGTGCCAGCAGCCGCGGTAATTCCA
GCTCCAATAGCGTATATTAATGTTGTTGCAGTTAAAAAGCTCGTAGTTGGATTT
CTGGATGAGCGGCGGCCTTGCGCACGTTGCGCGGCATTACGCCTGTCGTTTCT
GTGAACAAATTAGAGTGTTCAAAGCAAGGTTTGAATACATTAGCATGGAATAATA
GATAGGACTTGTGGTATTTTGTTGGTTGCACGAAAGTAATGATTAATAGGACAG
TGGGGGTTTCGTATTCAATTGTCAGAGGTGAAATTCTTGGATTTATGGACGAAC
TACTGCGAAAGCATTTACCAAGGATGTTTTCATTAATCAAGAACGAAAGTTAGG

GGACGAAGAAGATTAGATACCTTCGTATCTAACCATAAACTATGCCGACTCGGG
ATTGGCGGTCGGACTCCGCAGCACCGTATGAGAAATCAAAGTCTTTGGGTTCCG
GGGGGAGTATGGTCGCAAGGCTGAAACTTAAAGAAATTGACGGAAGGGCACCAC
CAGGAGTGGAGCCTGCGGCTTAATTTGACTCAACACGGGGAAACTACCAGGTCC
AGACATAGTAAGGATGACAGATTGAGAGCTCTTTCTTGATTCTTGGGTGGTGGT
GCATGGCCGTTCTTAGTTGGTGGAGTGATTTGTCTGGTTAATTCCGTAACGAAC
GAGACCCCCGCCTGCTAAATAGTTGGAATGCTTCGCATGATCGCTTCTTAGAGG
GACTTCGTGTGACTAACACGAGGAAGTTGGGGGCAATAACGGTCTGTGATGCCC
TTAGATGTTCTGGGCCGCACGCGCGCTACACTGATCATGCAACGAGTTTCCTTG
GCCGGGAGGCCTGGGAATCTGAACGTGATCGTGAAGGGATAGATTATTGCAAT
TATTAATCTTGAACGAGGAATTCCTAGTAACGCAAGTCAACAGCTTGCATGATTA
CTCCCGCCCTTGTACACACCGCCCGTCGCCCTACCATCGAAGGTAATGATGAGC
CCCAGACAGTCGATTGGCGC
>HQ710559.1_Apedinella_radians_culture_CCMP1767
TCTAAGTATAAACGCTATACTGTGAAACTGCGAGGCTCATAAACCAGTTATAGTT
TATTTGATATTCCCCTACTACTTGGATACCCGTAGTAATTCTAGAGCTAATACAT
GCGTCAAGCGACATTTGTCGTGTCATTATTAGATAGAAACCAATCTTCGGGGAA
TTGTGGTGAATCATGATAATTAGCGGATCGCATGGCCTGCGCCGGCGATGGAT
CATTCAATTCTGCCCTATCAGCTTGGATGGTAGGGTATTGGCCTACCATGGCTT
TAACGGGTAACGGAGAATAGGGTTCGATTCCGGAGGGAGCCTGAGAGACGGCT
ACCACATCCAAGGAAGGCAGCAGGCGCGTAAATTACCCAATCCTGACTCAGGGA
GGTAGTGACAAAAAATAACTTAGTCGGGCCGTTTTGGTCTGACTTTAGGAATGA
GAACAATTTAAATCCCTTATCGAGGATCAATTGGAGGGCAAGTCTGGTGCCAGC
AGCCGCGGTAATTCCAGCTCCAATAGCGTATATTAATGTTGTTGCAGTTAAAAA
GCTCGTAGTTGGATTTCTGGGTCTTCGCTGCTTTGCCGCAGGCTATGCGGTAT
TACGCCTGTCGTTTCTGTGAACAAATTAGAGTGTTCAAAGCAAGGTTTGAATAC
ATTAGCATGGAATAATAGATAGGACTTGTGGTATTTTGTTGGTTGCACGAAAGT
AATGATTAATAGGACAGTGGGGGTTTCGTATTCAATTGTCAGAGGTGAAATTCT
TGGATTTATGGACGAACTACTGCGAAAGCATTTACCAAGGATGTTTTCATTAAT
CAAGAACGAAAGTTAGGGGACGAAGAAGATTAGATACCTTCGTATCTAACCATA
AACGATGCCGACTCGGGATTGGCGGTCGGTCTCCGCAGCACCGTATGAGAAATC
AAAGTCTTTGGGTTCCGGGGGGAGTATGGTCGCAAGGCTGAAACTTAAAGAAAT

>Helicopedinella_tricostata_AB097408
TCTAAGTATAAACGCTATACTGTGAAACTGCGAGGCTCATAAACCAGTTATAGTT
TATTTGATATTCCCCTACTACTCGGATACCCGTAGTAATTCTAGAGCTAATACGT
GCGTCAAACGACATATGTCGTGTCATTATTAGATAGAAACCAATTCTCGGGGAA
TTGTGGTGAGTCATGATAATTAGCGGATCGCATGGCCTGCGCCGGCGATGGAT
CATTCAATTCTGCCCTATCAGCTTGGATGGTAGGGTATTGGCCTACCATGGCTT
TAACGGGTAACGGAGAATAGGGTTCGATTCCGGAGGGAGCCTGAGAGACGGCT
ACCACATCCAAGGAAGGCAGCAGGCGCGTAAATTACCCAATCCTGACTCAGGGA
GGTAGTGACAAAAAATAACTTAGACGGGCCTTTTCGGTCTGTCTTTAGGAATGA
GAACAATTTAAATCCCTTATCGAGGATCAATTGGAGGGCAAGTCTGGTGCCAGC
AGCCGCGGTAATTCCAGCTCCAATAGCGTAT-
TTAATGTTGTTGCAGTTAAAAAGCTCGTAGTTGGATTTCTGGTCGATCGCCGGC
CGGCCGCAGGGTGCGCGGTATTACGCCTGTCGTTTCTGTGAACAAATAAGAGT
GTTCAAAGCAAGGTTTGAATACATTAGCATGGAATAATAGATAGGACTTGTGGT
ATTTTGTTGGTTGCACGAAAGTAATGATTAATAGGACAGTGGGGGTTTCGTATT
CAATTGTCAGAGGTGAAATTCTTGGATTTATGGACGAACTACTGCGAAAGCATT
TACCAAGGATGTTTTCATTAATCAAGAACGAAAGTTAGGGGACGAAGAAGATTA
GATACCTTTGTATCTAACCATAAACTATGCCGACTCGGGATTGGCGGTCGGGCT
CCGCAGCACCGTATGAGAAATCAAAGTCTTTGGGTTCCGGGGGGAGTATGGTC
GCAAGGCTGAAACTTAAAGAAATTGACGGAAGGGCACCACCAGGAGTGGAGCCT
GCGGCTTAATTTGACTCAACACGGGGAAACTACCAGGTCCAGACATAGTAAGGA
TGACAGATTGAG-G-
TCTTTCTTGATTCTTGGGTGGTGGTGCATGGCCGTTCTTAGTTGGTGGAGTGA
TTTGTCTGGTTAATTCCGTAACGAACGAGACCCCCGCCTGCTAAATAGCCGGAA
TGCTTTGCATGCGTGCTTCTTAGAGGGACTGTGTGAGACTATCACACGGAAGTT
GGGGGCAATAACGGTCTGTGATGCCCTTAGATGTCCTGGGCCGCACGCGCGCT
ACACTGATCGTGCAACGAGTATCCTTGGCCGGGAGGCCTGGGAATCTGAACGCG
ATCGTGAAG---

>Florenciella_sp._RCC1587_KF422624

TGTGAAACTGCGAGGCTCATAAACCAGTTATAGTTTATTTGATATTCCCCTACTA
CTCGGATACCCGTAGTAATTCTAGAGCTAATACGTGCATCAACCCCCAACTGAG
GGGTCATTATTAGATAGAAGCCAATCGCAAGTGTATTGTGGTGAGTCATGATAA

TGTGCGGATCGCAGGGCCTGCGCCGGCGATGGATCATTCAATTCTGCCCTATCA
GCTTGGATGGTAGGGTATTGGCCTACCATGGCTTTAACGGGTAACGGAGAATG
GGGTTCGATTCCGGAGGGAGCCTGAGAGACGGCTACCACATCCAAGGAAGGCA
GCAGGCGCGTAAATTACCCAATCCTGACACAGGGAGGTAGTGACAAAAAATAAC
AATGCCGGGCTTTTTCAGTCTGGCAATTGGAATGAGAACAATTTAAATCCCTTA
TCGAGGATCAATTGGAGGGCAAGTCTGGTGCCAGCAGCCGCGGTAATTCCAGCT
CCAATAGCGTATATTAAAGTTGTTGCAGTTAAAAAGCTCGTAGTTGGATTTCTG
GTGGGCTGTCGCCTGGCCGAAGGGTTTGCGGCATTGAGCTCGTCGTTTCTGTG
AACAAATTAGAGTGTTCAAAGCAGGCTTTGAATACATTAGCATGGAATAATAGAT
AGGACTTGTGGTATTTTGTTGGTTGCACGGAAGTAATGATTAATAGGGCGGTG
GGGGTTTCGTATTCAATTGTCAGAGGTGAAATTCTTGGATTTATGGACGAACTA
CTGCGAAAGCATTTACCAAGGATGTTTTCATTAATCAAGAACGAAAGTTAGGGG
ACGAAGAAGATTAGATACCTTCGTATTTAACCATAAACTATGCCGACTAGGGATT
GGCGGTCGGGCTCCGCAGCACCTTATGAGAAATCAAAGTCTTTGGGTTCCGGG
GGGAGTATGGTCGCAAGGCTGAAACTTAAAGAAATTGACGGAAGGGCACCACCA
GGAGTGGAGCCTGCGGCTTAATTTGACTCAACACGGGGAAACTACCAGGTCCAG
ACATAGTAAGGATGACAGATTGAGAGCTCTTTCTTGATTCTTGGGTGGTGGTGC
ATGGCCGTTCTTAGTTGGTGGAGTGATTTGTCTGGTTAATTCCGTAACGAACGA
GACCCCCGCCTGCTAAATAGTCGGAATGCTTCGCATGCCCGCTTCTTAGAGGGA
CTTTTGGTGACTAACCAAAGGAAGTTGGGGGCAATAACGGTCTGTGATGCCCTT
AGATGTCCTGGGCTGCACGCGCGCTACACTGACCATGCAACGAGTCTCCTTGGC
TGGAAGGCCTGGGAATCTGAACGTGGTCGTGAAGGGATAGATTATTGCAATTAT
TAATCTTGAACGAGGAATTCCTAGTAACGCAAGTCATCAGCTTGCATGATTACTC
CCGCCCTTGTACACACCGCCCGTCGCCCTACCATTGAATGGTTCGGTGAGGTCC
AGATCGTGGCTTAGACC
>Florenciella_parvula_AY254857
TCTAAGTATAAACGCTATACTGTGAAACTGCGAGGCTCATAAACCAGTTATAGTT
TATTTGATATTCCCCTACTACTCGGATACCCGTAGTAATTCTAGAGCTAATACGT
GCATCAACCCCCAACTGAGGGGTCATTATTAGATAGAAGCCAATCGCAAGTGTA
TTGTGGTGAGTCATGAAAATGTGCGGATCGCAGGGCCTGCGCCGGCGATGGAT
CATTCAATTCTGCCCTATCAGCTTGGATGGTAGGGTATTGGCCTACCATGGCCT
TAACGGGTAACGGAGAATGGGGTTCGATTCCGGAGGGAGCCTGAGAGACGGCT
ACCACATCCAAGGAAGGCAGCAGGCGCGTAAATTACCCAATCCTGACACAGGGA
GGTAGTGACAAAAAATAACAATGCCGGGCTTTTTCAGTCAGGCAATTGGAATGA
GAACAATTTAAATCCCTTATCGAGGATCAATTGGAGGGCAAGTCTGGTGCCAGC
AGCCGCGGTAATTCCAGCTCCAATAGCGTATATTAAAGTTGTTGCAGTTAAAAA

GCTCGTAGTTGGATTTCTGGTGGGCTGTCGCCTGGCCGAAGGGTTTGCGGCAT
TGAGCTCGTCGTTTCTGTGAACAAATTAGAGTGTTCAAAGCAGGCTTTGAATAC
ATTAGCATGGAATAATAGATAGGACTTGTGGTATTTTGTTGGTTGCACGGAAGT
AATGATTAATAGGGCGGTGGGGGTTTCGTATTCAATTGTCAGAGGTGAAATTCT
TGGATTTATGGACGAACTACTGCGAAAGCATTTACCAAGGATGTTTTCATTAAT
CAAGAACGAAAGTTAGGGGACGAAGAAGATTAGATACCTTCGTATTTAACCATA
AACTATGCCGACTAGGGATTGGCGGTCGGGCTCCGCAGCACCTTATGAGAAATC
AAAGTCTTTGGGTTCCGGGGGGGGTATGGTCGCAAGGCTGAAACTTAAAGAAAT
TGACGGAAGGGCACCACCAGGAGTGGAGCCTGCGGCTTAATTTGACTCAACACG
GGGAAACTACCAGGTCCAGACATAGTAAGGATGACAGATTGAGAGCTCTTTCTT
GATTCTTGGGTGGTGGTGCATGGCCGTTCTTAGTTGGTGGAGTGACTTGTCTG
GTTAATTCCGTAACGAACGAGACCCCCGCCTGCTAAATAGTCGGAATGCTTCGC
ATGCCCGCTTCTTAGAGGGACTTTTGGTGACTAACCAAAGGAAGTTGGGGGCAA
TAACGGTCTGTGATGCCCTTAGATGTCCTGGACTGCACGCGCGCTACACTGACC
ATGCAACGAGTCTCCTTGGCTGGAAGGCCTGGGAATCTGAACGTGGTCGTGAA
GGGATAGATTATTGCAATTATTAATCTTGAACGAGGAATTCCTAGTAACGCAAAT
CATCAGCTTGCATGATTACTCCCGCCCTTGTACACACCGCCCGTCGCCCTACCAT
TGAATGGTTCGGTGAGGTCCAGATCGTGGCTTAGACC
>EU024988.1_Pseudopedinella_sp._FU44-42M

TCCAAGGAAGGCAGCAGGCGCGTAAATTACCCAATCCTGACTCAGGGAGGTAGT
GACAAAAAATAACTTAGTCGGGCCTTTTAGGTCTGACTTTAGGAATGAGAACAA
TTTAAATCCCTTATCGAGGATCAATTGGAGGGCAAGTCTGGTGCCAGCAGCCGC
GGTAATTCCAGCTCCAATAGCGTATATTAATGTTGTTGCAGTTAAAAAGCTCGT
AGTTGGATTTCTGGTCGGGCGTTGGCCGGCCGCAGGGTTCACGGTATTACGCC
TGTCGTTTCTGTGAACAAATTAGAGTGTTCAAAGCAAGGTTTGAATACATTAGC
ATGGAATAATAGATAGGACTTGTGGTATTTTGTTGGTTGCACGAAAGTAATGAT
TAATAGGACAGTGGGGGTTTCGTATTCAATTGTCAGAGGTGAAATTCTTGGATT
TATGGACGAACTACTGCGAAAGCATTTACCAAGGATGTTTTCATTAATCAAGAAC
GAAAGTTAGGGGACGAAGAAGATTAGATACCTTCGTATCTAACCTTAAACTATG
CCGACTCGGGATTGGAGGTCGGACTCCTCAGCACCGTATGAGAAATCAAAGTCT
TTGGGTTCCGGGGGGAGTATGGTCGCAAGGCTGAAACTTAAAGAAATTGACGG
AAGGGCACCACCAGGAGTGGAGCCTGCGGCTTAATTTGACTCAACACGGGGAAA

CTACCAGGTCCAGACATAGTAAGGATGACAGATTGAGAGCTCTTTCTTGATTCT
TGGGTGGTGGTGCATGGCCGTTCTTAGTTGGTGGAGTGATTTGTCTGGTTAAT
TCCGTAACGAACGAGACCCCCGCCTGCTAAATAGCCGGAATGCTCTGCACGATC
GCTTCTTAGAGGGACTTTGTGTGACTAACACAATGAAGTTGGGGGCAATAACGG
TCTGTGATGCCCTTAGATGTTCTGGGCCGCACGCGCGCTACACTGATCATGCAA
CGAGTTTCCTTGGCCGAGAGGCCTGGGAATCTGAACGTGATCGTGAAGGGATT
GATTATTGCAATTATTAATCATGAACGAGGAATTCCTAGTAACGCAAGTCATCAG
CTTGCATGATTACTCCCGCCCTT---

>Dictyocha_speculum_U14385
TCTAAGTATAAACGTTATACTGTGAAACTGCGAAGCTCATAAACCAGTTATAGTT
TATTTGATATTCCTCTACTACTCGGATACCCGTAGTAATTCTAGAGCTAATACGT
GCATCAAATCCCAACTGCTACGTCATTATTAGAAAGAAGCCAATCGCAAGGGTAC
TGTGGTGAGTCATGATAATGTACGGATCGTATGGCCTGCGCTGACGATGGATCA
TTCAATTCTGCCCTATCAGCTTGGATGGTAGGGTATTGGCCTACCATGGCTTTA
ACGGGTAACGGAGAATAGGGTTCGATTCCGGAGGGAGCCTGAGAGACGGCTAC
CACATCCAAGGAAGGCAGCAGGCGCGTAAATTACCCAATCCTGATACAGGGAGG
TAGTGACAAAAAATAACAATGCCGGGCTTTTTTAGTCTGGCAATTGGAATGAGA
ACAATTTAAATCCCTTATCGAGGATCAATTGGAGGGCAAGTCTGGTGCCAGCAG
CCGCGGTAATTCCAGCTCCAATAGCGTATATTAATGTTGTTGCAGTTAAAAAGC
TCGTAGTTGGATTTCTGATGGATCGACGGTCGGCCGAAGGGTTTTCGGCATTG
AGCTCGTCGTTTCTGTGAACAAATTAGAGTGTTCAAAGCAGGCTTTGAATACAT
TAGCATGGAATAATAGATAGGACTTGTGGTATTTTGTTGGTTGCACGGAAGTAA
TGATTAATAGGACAGTGGGGATTCCGTATTCGATTGTCAGAGGTGAAATTCTTG
GATTTATGGACGAACTACTGCGAAAGCATTCATCAAGGATGTTTTCATTAATCAA
GAACGAAAGTTAGGGGACGAAGAAGATTAGATACCTTCGTATTTAACCATAAAC
TATGCCGACTAGGGATTGGCGGTCGGGCTCCGCAGCACCTTATGAGAAATCAAA
GTCTTTGGGTTCCGGGGGGAGTATGGTCGCAAGGCTGAAACTTAAAGAAATTG
ACGGAAGGGCACCACCAGGAGTGGAGCTTGCGGCTTAATTTGACTCAACACGGG
AAAACTACCAGGTCCAGACATAGTAAGGATGACAGATTGAGAGCTCTTTCTTGA
TTCTTGGGTGGTGGTGCATGGCCGTTCTTAGTTGGTGGAGTGATTTGTCTGGT
TAATTCCGTAACGAACGAGACCCCCGCCTGCTAAATAGTCGGAATGCTTTGCAT
GCTCACTTCTTAGAGGGACTTTCGGCGACTAGCCGAAGGAAGTTGGGGGCAATA
ACGGTCTGTGATGCCCTTAGATGTCCTGGGCTGCACGCGCGCTACACTGATCAT
GCAACGAGTTTCCTTGGCTGAGAGGCCTGGGAATCTGAACGTGATCGTGAAGG
GATTGATGATTGCAACTATTCATCATGAACGAGGAATTCCTAGTAACGTGAGTC

ATCAGCTCACATGATTACTCCCGCCCTTGTACACACCGCCCGTCGCCCTACCATT
GAATGGCTCGGTGAGGCCCAGATTCTGGTCTGAACC
>Dictyocha_octonaria_strain_NIWA1026_HQ646562
TCTAAGTATAAACGTTATACTGTGAAACTGCGAAGCTCATAAACCAGTTATAGTT
TATTTGATATTCCTCTACTACTCGGATACCCGTAGTAATTCTAGAGCTAATACGT
GCATCAAATCCCAACTGCTACGTCATTATTAGAAAGAAGCCAATCGCAAGGGTAC
TGTGGTGAGTCATGATAATGTTCGGATCGTATGGCCTGCGCTGACGATGGATCA
TTCAATTCTGCCCTATCAGCTTGGATGGTAGGGTATTGGCCTACCATGGCTTTA
ACGGGTAACGGAGAATAGGGTTCGATTCCGGAGGGAGCCTGAGAGACGGCTAC
CACATCCAAGGAAGGCAGCAGGCGCGTAAATTACCCAATCCTGATACAGGGAGG
TAGTGACAAAAAATAACAATGCCGGGCTTTTTAGTCTGGCAATTGGAATGAGA
ACAATTTAAATCCCTTATCGAGGATCAATTGGAGGGCAAGTATGGTGCCAGCAG
CCGCGGTAATTCCAGCTCCAATAGCGTATATTAATGTTGTTGCAGTTAAAAAGC
TCGTAGTTGGATTTCTGATGGATCGACGGTCGGCCGAAGGGTTTTCGGCATTG
AGCTCGTCGTTTCTGTGAACAAATTAGAGTGTTCAAAGCAGGCTTTGAATACAT
TAGCATGGAATAATAGATAGGACTTGTGGTATTTTGTTGGTTGCACGGAAGTAA
TGATTAATAGGACAGTGGGGATTCCGTATTCGATTGTCAGAGGTGAAATTCTTG
GATTTATGGACGAACTACTGCGAAAGCATTCATCAAGGATGTTTTCATTAATCAA
GAACGAAAGTTAGGGGACGAAGAAGATTAGATACCTTCGTATTTAACCATAAAC
TATGCCGACTAGGGATTGGCGGTCGGGCTCCGCAGCACCTTATGAGAAATCAAA
GTCTTTGGGTTCCGGGGGGAGTATGGTCGCAAGGCTGAAACTTAAAGAAATTG
ACGGAAGGGCACCACCAGGAGTGGAGCTTGCGGCTTAATTTGACTCAACACGGG
AAAACTACCAGGTCCAGACATAGTAAGGATG-
CAGATTGAGAGCTCTTTCTTGATTCTTGGGTGGTGGTGCATGGCCGTTCTTAGT
TGGTGGAGTGATTTGTGTGGTTAATTCCCTAACGAACGAGACCCCCGCCTGCTA
AATAGTCAGAATGCTTTGCAT-CTCACTTCTTAGAGG---
TTTCGGCGACTAGCCGAAGGAAGTTGGGGGCAATAACGGT-
TGTGATGCCCTTAGATGTCCTGGGTTGCACGCGCGCTACACTGATCATGCAACG
AGTTTACTTGGCTGAGAGGCCTGGGAATATGAACGTGATCGTGAA-----------------
----

>Dictyocha_fibula_AB096710
TCTAAGTATAAACGTTATACTGTGAAACTGCGAAGCTCATAAACCAGTTATAGTT
TATTTGATAGTCCCTTACTACTCGGATACCCGTAGTAATTCTAGAGCTAATACGT
GCATCAAATCCCAACTGCTANGTCATTATTAGAAAGAAGCCAATCGCAAGGGTAC
TGTGGTGAGTCATGATAATGTACGGATCGTATGGCCTGCGCCGACGATGGATCA

TTCAATTCTGCCCTATCAGCTTGGATGGTAGGGTATTGGCCTACCATGGCTTTA
ACGGGTAACGGAGAATAGGGTTCGATTCCGGAAGGAGCCTGAGAGAC-
GCTACCACATCCAAGGAAGGCAGCAGGCGCGTAAATTACCCAATCCTGATACAG
GGAGGTAGTGACAAAAAATAACAATGCCGGGCTTTTTAGTCTGGTAATTGGAA
TGAGAACAATTTAAATCCCTTATCGAGGATCAATTGGAGGGCAAGTCTGGTGCC
AGCAGCCGCGGTAATTCCAGCTCCAATAGCGTATATTAA-
GTTGTTGCAGTTAAAAAGCTCGTAGTTGGATTTCTGACTGGACGGCGGTCGGT
CACGGAGCGTGTGGCAGCGACCTGGACCTTTCTGTGAACAAATTAGAGTGTTCA
AAGCAGGCTCGGAATATATTAGCATGGAATAATAAATAGGACTTGCGGTATTTT
GTTGGTTGGACGACAGTAATGATTAATAGGACAGTGGGGATTTCGTATTCAATT
GTCAGAGGTGAAATTCTTGGATTTATGGACGAACTACTGCGAAAGCATTCATCA
AGGATGTTTTCATTAATCAAGAACGAAAGTTAGGGGACGAAGAAGATTAGATAC
CTTCGTATTTAACCATAAACTATGCCGACTAGGGATTGGCGGTCGGGCTCCGCA
GCACCTTATGAGAAATCAAAGTCTTTGGGTTCCGGGGGGAGTATGGTCGCAAG
GCTGAAACTTAAAGAAATTGACGGAAGGGCACCACCAGGAGTGGAGCTTGCGGC
TTAATTTGACTCAACACGGGAAAACTACCAGGTCCAGACATAGTAAGGATGACA
GATTGAGAGCTCTTTCTTGATTCTTGGGTGGTGGTGCATGGCCGTCCTTAGTT
GGTGGAGTGATTTGTCTGGTTAATTCCGTAACGAACGAGACCCCCGCCTGCTAA
ATAGTCAGAATGCTTTGCATGCTCACTTCTTAGAGGGACTTTCGGCGACTAGCC
GAAGGAAGTTGGGGGCAATAACGGTCTGTGATGCCCTTAGATGTCCTGGGCTG
CACGCGCGCTACACTGATCATGCAACGAGTTTCCTTGGCTGAGAGGCCTGGGAA
TCTGAACGTGATCGTGAAGGGATTGATGATTGCAACTATTCATCATGAACGAGG
AATTCCTAGTAACGTGAGTCATCAGCTCACATGATTACTCCCGCCTTTGTACACA
CCGCCCGTCGCCCTACCATTGAATGGCTCGGTGAGGCCCAGATTTTGGTCTAAA
CC
>Ciliophrys_infusionum_AB081641
TCTAAGTATAAGCATTATACAGCGAAACTGCGAGGCTCATAAACCAGTTATAGTT
TCTTTGATAGTTCCCTACTACTCGGATAACCGTAGTAATTCTAGAGCTAATACGT
GCATAAGCCTTTCTTTTCGGTGTCATTATTAGATAGAAACCAATCGCAAGGGTA
TTGTGGTGAGTCATGATAATTCGCGGATCGTATGGCCTGCGCCGACGACGGAT
CATTCAATTCTGCCCTATCAGCTTGGATGGTACGGTATTGGGCTACCATGGCTT
TAACGGGTAACGGGGAATAGGGTTCGATTCCGGAAGGAGCCTGAGAGACGGCT
ACCACATCCAAGGAAGGCAGCAGGCGCGTAAATTACCCAATCCTGACACAGGGA
GGTAGTGACAAGAAATAACGTAGTCGGGCGCTTATTGTCTGACTTTCGGAATGA
GAACAATTTAAATCCCTTATCGAGGATCAATTGGAGGGCAAGTCTGGTGCCAGC
AGCCGCGGTAATTCCAGCTCCAATAGCGTATATTAATGTTGTTGCAGTTAAAAA

GCTCGTAGTTGGATTTCGGACTGAGAGGCGGCCGGCCGCAGGGTTTTCGGCAT
TTGACCCGTCGTTTCTGTGAACAAATTAGAGTGTTCCAAGCAGGGTTTGAATAC
ATTAGCATGGAATAATAGATAGGACTTGTGGTATTTTGTTGGTTGCACGAAAGT
AATGATTAATAGGACAGTGGGGGTTTCGTATTCAATTGTCAGAGGTGAAATTCT
TGGATTTCTGGACGAATTACTGCGAAAGCATTTACCAAGGATGCGTTCATTAAT
CAAGAACGAAAGTTAGGGGACGAAGAAGATTAGATACCTTCGTATTTAACCATA
AACGATGCCGACTCGGGATTGGCGGTCGGACTCCGCAGCACCGTATGAGAAATC
AAAGTCTTTGGGTTCCGGGGGGAGTATGGTCGCAAGGCTGAAACTTAAAGAAAT
TGACGGAAGGGCACCACCAGGAGTGGAGCCTGCGGCTTAATTTGACTCAACACG
GGGAAACTACCAGGTCCAGACATAGTAAGGATGACAGATTGAGAGCTCTTTCTT
GATTCTTGGGTGGTGGTGCATGGCCGTTCTTAGTTGGTGGAGTGATTTGTCTG
GTTAATTCCGTAACGAACGAGACCCCCGCCTGCTAAATAGCACGAATGCTTAGC
ATGATCGCTTCTTAGAGGGACTGTCGGTGACTAATCGACGGAAGTTGGGGGCA
ATAACGGTCTGTGATGCCCTTAGATGTCCTGGGCCGCACGCGCGCTACACTGAT
CATGCAACGAGTTTCCTTGGCCGGAAGGCTTGGGAATCTGAACGTGATCGTGAA
GGGATTGATTTTTGCAATTCTGAATCATAAACGAGGAATTCCTAGTAACGCAAG
TCATCAGCTTGCATGATTACTCCCGCCCTTGTACACACCGCCCGTCGCCCTACCA
TCGAAGGTAATGATGAGCCCTAGATTGTCATTCTCGCA
>Aureoumbra_lagunensis_U40258
TCTAAGTATAAATGTTGTACAGTGAAACTGCGAGGCTCATATATCAGTTATAGT
TTATTTGATAGTCCCTTACTACATGGATAACCGTAGTAATTCTAGAGCTAATACA
TGCTTCAACTCCCAACTGCTACGTATTTATTAGATTGAAATCAATGGCAA-
GGTATTGTGGTGAGTCATGATAACTTTCGAATCGCATGGCTTATGCCGGCGATG
GATCATTCAATTCTGCCCTATCAGCTT-
GATGGTAGGGTATTGGCCTACCATGGCATTTACGGGTAACGGAGAATGGGGTT
CGATTCCGGAGGGAGCCTGAGAAACGGCTACCACATCCAAGGAAGGCAGCAGGC
GCGTAAATTACCCAATCCTGACACAGGGAGGTAGTGACAAGAAATAACAATGCC
GGGCTCTTTCAGTCTGGCAATTGGAATGAGAACAATTTAAATCCCTTATCGAGG
ATCCAGTGGAGGGCAAGTCTGGTGCCAGCAGCCGCGGTAATTCCAGCTCCACTA
GCGTATATTAATGTTGTTGCAGTTAAAAAGCTCGTAGTTGGATTCCTGGCAGGG
GTATGGTCCGTTGCAAGATGGTCGGCATTGAGTCTAACGTTTCTGTGAAAAAAT
CAGAGTGTTCAAAGCAGGCTTTGAATACATTGGCATGGAATAATAGATAGGACC
TGCGGTATTTTGTTGGTTGCACGCAGGTAATGATTAACAGGGCGGTGGGGTTT
TCGTATTCAATTGTCAGAGGTGAAATTCTTGGATTTATGGACGAACTGCTGCGA
AAGCGTCGAACAAGGACGTTCTCATTAATCAAGAACGAAAGTTAGGGGACGAAG
ATGATTAGATACCATCGTATTTAACCATAAACTATGCCGATTCAGGATTGGCGG

TCGGACTCCGCAGCACTGTATGAGAAATCAAAATCTTTGGGTTCCGGGGGGAGT
ATGGTCGCAAGGCTGAAACTTAAAGAAATTGACGGAAGGGCACCACAAGATGTG
GAGCTTGC-
GCTTAATTTGACTCAACACGGGGAAACTACCAGGTCCAGACATAGTAAGGATGA
CAGATTGAGAGCTCTTTCTTGATTCTTGGGTGGTGGTGCATGGCCGTTCTTAGT
TGGTGGAGTGATTTGTCTGGTTAATTCCGTAACGAACGAGACCCCCGCCTGCTA
AATAGCCGGAATGCCTTGCATGCCGTCTTCTTAGAGGGACTTTTGGTGACTAAC
CAGAGGAAGCTGGGGGCAATAACGGTCTGTGATGCCCTTAGATGTCCTGGGCC
GCACGCGCGCTACACTGGTCACGCAACGAGTCTCCTTGGTCGGAAGGCCTGGG
AATCCGAACGTGACCGTGATGGGATAGATTGTTGCAATTTTCAATCTTGAACGA
GGAATATCTAGTAACGCAAGTCATCAGCTTGCATGATTACTCCCGCCCTTGTAC
ACACCGCCCGTCGCCCTACCATTGAACGATTCGGTGAAATCTCGACTTTAGCAC
GCAGC
>Aureococcus_anophagefferens_strain_CCMP1784_AF118443
TCTAAGTATAAACGTTGTACTGTGAAACTGCGAGGCTCATATATCAGTTATAGT
TTATTTGATAGTCCCTTACTACATGGATACCCGTAGTAATTCTAGAGCTAATACA
TGCATCGACTCCCAACTGCTACGTATTTATTAGATTGAAACCAATGGGAGAGGT
ATTGTGGTGAGTCATGATAACTTGCGAATCGCATGGCCTGTGCCGGCGATGGA
TCATTCAATTCTGCCCTATCAGCTTGGATGGTAGGGTATTGGCCTACCATGGCA
TTAACGGGTAACGGAGAATGGGGTTCGATTCCGGAGGGAGCCTGAGAGACGGC
TACCACATCCAAGGAAGGCAGCAGGCGCGTAAATTACCCAATCCTGACACAGGG
AGGTAGTGACAAGAAATAACAATGCCGGGCTCTTACAGTCTGGCAATTGGAATG
AGAACAATTTAAATCCCTTATCGAGGATCCATTGGAGGGCAAGTCTGGTGCCAG
CAGCCGCGGTAATTCCAGCTCCAATAGCGTATATTAATGTTGTTGCAGTTAAAA
AGCTCGTAGTTGGATTCCTGGCAGGGGTACGGTCCGTTGCAAGGTATCCGGCA
TTGATCGCGACGTTTCTGTGAAAAAATCAGAGTGTTCAACGCAGGCTTTGAATA
CATTAGCATGGAATAATAGATAGGACCTGCGGTATTTTGTTGGTTGCACGGAGG
TAATGATTAAAAGGGCGGTGGGGTTTTCGTATTCAATTGTCAGAGGTGAAATTC
TTGGATTTATGGACGAACTGCTGCGAAAGCGTCGAACAAGGACGTTCTCATTAA
TCAAGAACGAAAGTTAGGGGACGAAGATGATTAGATACCATCGTATTTAACCAT
AAACTATGCCGATTCAGGATTGGCGGTCGGACTCCGCAGCACTGTATGAGAAAT
CAAAATCTTTGGGTTCCGGGGGGAGTATGGTCGCAAGGCTGAAACTTAAAGAAA
TTGACGGAAGGGCACCACCAGGAGTGGAGCCTGCGGCTTAATTTGACTCAACAC
GGGGAAACTACCAGGTCCAGACATAGTAAGGATGACAGATTGAGAGCTCTTTCT
TGATTCTTGGGTGGTGGTGCATGGCCGTTCTTAGTTGGTGGAGTGATTTGTCT
GGTTAATTCCGTAACGAACGAGACCCCCGCCTGCTAAATAGTTGGAATGCCTTG

CATGCCAACTTCTTAGAGGGACTTTCGGTGACTAACCGAAGGAAGCTGGGGGCA
ATAACGGTCTGTGATGCCCTTAGATGTCCTGGGCCGCACGCGCGCTACACTGGC
CACGCAACGAGTCTCCTTGGTCGGCAGGCCTGGGAATCTGAACGTGGCCGTGA
TGGGATTGATTGTTGCAATTTTCAATCATGAACGAGGAATTCCTAGTAACGCGA
GTCATCAGCTCGCATGATTACTCCCGCCCTTGTACACACCGCCCGTCGCCCTAC
CATTGAATGGTTCGGTGAAACCCCGACTGGGGTTCGCAGC
>AB097408.1_Helicopedinella_tricostata
TCTAAGTATAAACGCTATACTGTGAAACTGCGAGGCTCATAAACCAGTTATAGTT
TATTTGATATTCCCCTACTACTCGGATACCCGTAGTAATTCTAGAGCTAATACGT
GCGTCAAACGACATATGTCGTGTCATTATTAGATAGAAACCAATTCTCGGGGAA
TTGTGGTGAGTCATGATAATTAGCGGATCGCATGGCCTGCGCCGGCGATGGAT
CATTCAATTCTGCCCTATCAGCTTGGATGGTAGGGTATTGGCCTACCATGGCTT
TAACGGGTAACGGAGAATAGGGTTCGATTCCGGAGGGAGCCTGAGAGACGGCT
ACCACATCCAAGGAAGGCAGCAGGCGCGTAAATTACCCAATCCTGACTCAGGGA
GGTAGTGACAAAAAATAACTTAGACGGGCCGTTTTGGTCTGTCTTTAGGAATGA
GAACAATTTAAATCCCTTATCGAGGATCAATTGGAGGGCAAGTCTGGTGCCAGC
AGCCGCGGTAATTCCAGCTCCAATAGCGTAT-
TTAATGTTGTTGCAGTTAAAAAGCTCGTAGTTGGATTTCTGGTCGATCGCCGGC
CGGCCGCAGGGTGCGCGGTATTACGCCTGTCGTTTCTGTGAACAAATAAGAGT
GTTCAAAGCAAGGTTTGAATACATTAGCATGGAATAATAGATAGGACTTGTGGT
ATTTTGTTGGTTGCACGAAAGTAATGATTAATAGGACAGTGGGGGTTTCGTATT
CAATTGTCAGAGGTGAAATTCTTGGATTTATGGACGAACTACTGCGAAAGCATT
TACCAAGGATGTTTTCATTAATCAAGAACGAAAGTTAGGGGACGAAGAAGATTA
GATACCTTTGTATCTAACCATAAACTATGCCGACTCGGGATTGGCGGTCGGGCT
CCGCAGCACCGTATGAGAAATCAAAGTCTTTGGGTTCCGGGGGGAGTATGGTC
GCAAGGCTGAAACTTAAAGAAATTGACGGAAGGGCACCACCAGGAGTGGAGCCT
GCGGCTTAATTTGACTCAACACGGGGAAACTACCAGGTCCAGACATAGTAAGGA
TGACAGATTGAG-G-
TCTTTCTTGATTCTTGGGTGGTGGTGCATGGCCGTTCTTAGTTGGTGGAGTGA
TTTGTCTGGTTAATTCCGTAACGAACGAGACCCCCGCCTGCTAAATAGCCGGAA
TGCTTTGCATGGGTGCTTCTTAGAGGGACTGTGTGAGACTATCACACGGAAGTT
GGGGGCAATAACGGTCTGTGATGCCCTTAGATGTCCTGGGCCGCACGCGCGCT
ACACTGATCGTGCAACGAGTATCCTTGGCCGGGAGGCCTGGGAATCTGAACGCG
ATCGTGAAG--

>AB058361.1_Rhizochromulina_sp._MBIC10538

TCTAAGTATAAGCGTTGTACTGTGAAACTGCGAGGCTCATAAACCAGTTATAGT
TTATTTGATAGTTCCCTACTACTCGGATACCCGTAGTAATTCTAGAGCTAAGAC
GTGAATCAAGCTCCGCCTGGAGTGTCATTATTAGATAGAAACCAATCGCAAGGG
TATTGAGGTGAGTCATGATAATTAGCGGATCGCATGGCCTGCGCCGGCGACGG
ATCATTCAATTCTGCCCTATCAGCTTGGATGGTACGGTATTGGCCTACCATGGC
TTTAACGGGTAACGGAGAATAGGGTTCGATTCCGGAGGGAGCCTGAGAGACGG
CTACCACATCCAAGGAAGGCAGCAGGCGCGTAAATTACCCAATCCTGACACAGG
GAGGTAGTGACAAGAAATAACTTGGTCGGGCCCTTCCGGTCTGACTTTAGGAAT
GAGAACAATTTAAATCCCTTATCGAGGATCAATTGGAGGGCAAGTCTGGTGCCA
GCAGCCGCGGTAATTCCAGCTCCAATAGCGTATATTAATGTTGTTGCAGTTAAA
AAGCTCGTAGTTGGATTTCGAATGGAGCGGCGGTCGGCCGCAGGGTTGGCGGC
ATTGGTCCCGTCGTTTCTGTGAACAAATTAGAGTGTTCCAAGCAAGGCTTGAAT
ACATTAGCATGGAATAATAGATAGGCCTTGTGGTATTTTGTTGGTTGCACATAA
GGAATGATTAATAGGACAGTGGGGGTTTCGTATTCAATTGTCAGAGGTGAAATT
CTTGGATTTCTGGACGAACTACTGCGAAAGCATTCACCAAGGATGCGTTCATTG
ATCAAGAACGAAAGTTAGGGGACGAAGAAGATTAGATACCTTCGTATCTAACCA
TAAACGATGCCGACTCGGGATTGGCGGTCGGACTCCGCAGCACCGTATGAGAAA
TCAAAGTCTTTGGGTTCCGGGGGGAGTATGGTCGCAAGGCTGAAACTTAAAGAA
ATTGACGGAAGGGCACCACCAGGAGTGGAGCCTGCGGCTTAATTTGACTCAACA
CGGGGAAACTACCAGGTCCAGACATAGTAAGGATGACAGATTGAGAGCTCTTTC
TTGATTCTTGGGTGGTGGTGCATGGCCGTTCTTAGTTGGTGGAGTGATTTGTC
TGGTTAATTCCGTAACGAACGAGACCCCCGCCTGCTAAATAGCGGGAATGCATC
GCATGTTCGCTTCTTAGAGGGACTGTCAGTGACTAACTGACGGAAGTTGGGGG
CAATAACGGTCTGTGATGCCCTTAGATGTCCTGGGCCGCACGCGCGCTACACTG
ATCATGCAACGAGTCTCCTTGGCCGGAAGGCCTGGGAATCTGAACGTGATCGTG
AAGGGATAGATTATTGCAATTATTAATCTTGAACGAGGAATTCCTAGTAACGCAA
GTCATCAGCTTGCATGATTACTCCCGCCCTTGTACACACCGCCCGTCGCCCTAC
CATCGAAGGTAATGATGAGCCCTAGATTGTGGCTTGGGAC

TufA protein dataset
>YP_778573.1_Bigelowiella_natans
FERKPHVNIGTIGHVDHGKTTLTAAITMALAS-G-
KKKKYDDIDSAPEEKARGITINTAHVEYETETRHYAHVDCPGHADYVKNMITGAAQ
MDGAILVVSGADGPMPQTKEHLLLAKQVGVPSIVVFLNKEDQVDDEELLELVELEIR
EMLDTYDFPGDSTPIIKGSALMALQADKILTLMDNVDEYIPTPERETDKPFLMAVED
VFSITGRGTVATGRVERGGVKIGDVVEIVGLTRSTTVTGLEMFQKMLSIAGDNVGML
LRGIQKADIQRGMVVAQPGSITPHVSFDAQVYILTKEEGGRHTPFFKGYRPQFYVRTT
DVTGKIMVVPGDRVTMSVELVQPIAIEGMRFAIREGGRTVGAGVVSNVV
>YP_009732269.1_Gracilaria_spinulosa
FERKPHVNIGTIGHVDHGKTTLTAAISATLAAANDKAKKFDEIDAAPEEKARGITINTA
HVEYETQNRHYAHVDCPGHADYVKNMITGAAQMDGAILVVSAADGPMPQTREHIL
LAKQVGVPNIVVFLNKQDQLDDEELLELVELEVRELLVQYDFPGDDIPFVAGSALLAL
EKDKIHSLMDAVDEYIPTPIRDVEKTFLMAVEDVFSITGRGTVATGRIERGIIKVGDTIE
IVGLTATTTITGLEMFQKTLGMAGDNIGILLRGIQKKDIERGMVLAQPGTITPHTQFE
AEVYILTKEEGGRHTPFFSGYRPQFYVRTTDVTGTIMVMPGDRIKMSAELINPIAIEG
MRFAIREGGKTVGAGVVSKIL
>YP_009694241.1_Hapterophycus_canaliculatus
YDRKPHINIGTIGHVDHGKTTLTAAITAVLSLSGDSAKKYEDIDAAPEERARGITINTA
HVEYETETRHYAHVDCPGHADYVKNMITGAAQMDGAILVVSAADGPMPQTREHILL
SKQVGVPHIVVFLNKEDQVDDLELVELVELEVRELLSNYDFPGDDIPIVTGSALQALD
ADKIYSLMESVDNYIPTPIRDVDKAFLMAIEDVFSITGRGTVATGKIDRGIIKVGETVD
LVGLTKSTTVTGVEMFQKTLGVAGDNVGILLRGLQKGEIERGMVLAKPGTITPHNTF
ESELYILTKEEGGRHTPFFPGYRPQFYVRTTDVTGEIMVMPGDRVKMTAKLISLIAIEG
MRFAIREGGRTIGAGVVSKII
>YP_009691331.1_Laminaria_digitata
YDRKPHINIGTIGHVDHGKTTLTAAITAVLSLTGDNAKKYEDIDAAPEERARGITINTA
HVEYETESRHYAHVDCPGHADYVKNMITGAAQMDGAILVVSAADGPMPQTREHILL
SKQVGVPHIVVFLNKEDQVDDLELVELVELEVRELLSNYDFPGDDIPILTGSALQALD
ADKIYSLMDSVDSYIPTPIRDVDKPFLMAIEDVFSITGRGTVATGKIDRGIVKVGETVD
LVGLTKSTTVTGVEMFQKTLGVAGDNVGILLRGLQKDDIERGMVLSKPGTITPHNT
FESELYILTKEEGGRHTPFFPGYRPQFYVRTTDVTGEIMVMPGDRVKMTAKLISLIAIE
GMRFAIREGGRTIGAGVVSKII
>YP_009687971.1_Haslea_nusantara
FERKPHVNIGTIGHVDHGKTTLTAAITATLSLEGTAVKDYADIDGAPEERARGITINT
AHVEYETANRHYAHVDCPGHADYVKNMITGAAQMDGAILVVSAADGPMPQTREHI

LLSKQVGVPHIVVFLNKEDQVDDAELIELVELEVRELLSAYDFPGDDIPICPGSALQAI
EADKIFALMDAVDDYIPTPERDTEKTFLMAIEDVFSITGRGTVATGRIERGVVKVGDN
VEIVGVTQSTTITGIEMFQKTLGYAGDNVGILLRGVTREDIERGMVLAQPGTITPHTS
FESEVYVLTKDEGGRHTPFFTGYRPQFYVRTTDVTGAIMVMPGDRIKMTAELIYPVAI
EGMRFAIREGGRTIGAGVVSKIV
>YP_009684620.1_Pseudopedinella_elastica
FERKPHVNIGTIGHVDHGKTTLTAAITAVLSTIGNTLKNYADIDAAPEEKARGITINTA
HVEYETESRHYAHVDCPGHADYVKNMITGAAQMDGAILVVSAADGPMPQTREHILL
AKQVGVPNIVVFLNKEDQVDDEELIELVELEVRELLSNYDFPGDDIPIVPGSALQALEA
DKIFNLMDEVDNYIPTPERDTEKTFLMAVEDVFSITGRGTVATGRIERGIINVGDTIEI
VGLTQTTTVTGVEMFQKTLGMAGDNVGILLRGVQKTDIERGMVLSKPGTITPHTLF
EGEVYILTKEEGGRHTPFFTGYRPQFYVRTTDVTGNIMVMPGDRIKMTAELISAIAIE
GMRFAIREGGRTIGAGVVSKIL
>YP_009684446.1_Florenciella_parvula
FERKPHVNIGTIGHVDHGKTTLTAAITATLSTVGTELKNYADIDASPEEKARGITINTA
HVEYETETRHYAHVDCPGHADYVKNMITGAAQMDGAILVVSAADGPMPQTREHILL
AKQVGVPNLVVFLNKEDQVDDDELIELVEMETRELLNNYDFPGDDIPITPGSALQAL
EKDKIFALMDSVDEYIPTPTRDTEKTFLMAVEDVFSITGRGTVATGRIERGVINVGDTI
EIVGLTQTTTVTGVEMFQKTLGMAGDNVGILLRGVQKEQIERGMVLSEPGTITPHT
QFEGEVYILTKEEGGRHTPFFTGYRPQFYVRTTDVTGNIMVMPGDRIKMTAELISAIA
IEGMRFAIREGGRTIGAGVVSKIL
>YP_009677073.1_Dictyocha_speculum
FERKPHVNIGTIGHVDHGKTTLTAAITATLSTLGNELKNYADIDAAPEEKARGITINTA
HVEYETLDRHYAHVDCPGHADYVKNMITGAAQMDGAILVVSAADGPMPQTREHILL
AKQVGVPNLVCFLNKEDQVDDEELIELVELEVRELLSNYDFPGDDIPITVGSALQALE
TDKIFSLMDTVDEYIPTPVRDTEKTFLMAVEDVFSITGRGTVATGRIERGVINVGDTIE
IVGLTQSTTVTGVEMFQKTLGMAGDNVGILLRGLQKTDIERGMVLSKPGTITPHTEF
EGEVYILTKEEGGRHTPFFTGYRPQFYVRTTDVTGDIMVMPGDRIKMTAELISAIAIE
GMRFAIREGGRTIGAGVVSKIL
>YP_009674964.1_Rhizochromulina_marina
FERKPHVNIGTIGHVDHGKTTLTAAITATLSTLGNQLKNYADIDAAPEEKARGITINT
AHVEYETDNRHYAHVDCPGHADYVKNMITGAAQMDGAILVVSAADGPMPQTREHI
LLAKQVGVPNIVVFLNKEDQVDDAELIELVELEVRELLSNYDFPGDDIPIVAGSALQAL
EKDKIFALMDEVDNYIPTPERDTDKTFLMAVEDVFSITGRGTVATGRIERGVVTVGD
TIEIVGLTKSTTVTGVEMFQKTLGMAGDNVGILLRGIQKTDIERGMVLSKPGTITPHT
LFEGEVYILTKEEGGRHTPFFTGYRPQFYVRTTDVTGNIMVMPGDRIKMTAELISAIAI

EGMRFAIREGGRTIGAGIVSKIL
>YP_009660586.1_Corallina_chilensis
FERKPHVNIGTIGHVDHGKTTLTAAISATLAAANNTAKKFDEIDAAPEEKARGITINTA
HVEYETENRHYAHVDCPGHADYVKNMITGAAQMDGAILVVSAADGPMPQTREHILL
AKQVGVPNVVVFLNKQDQVDDDELLELVELEVRELLSQYDFPGDDIPFVAGSALLAL
EEDKIHSLMTAVDDYIPTPERDVDKTFLMAVEDVFSITGRGTVATGRIERGIIKVGDTI
EIVGLTKSTTITGLEMFQKTLGMAGDNIGILLRGIQKNEIERGMVLAQPNTITPHTQF
EAEVYILTKDEGGRHTPFFSGYRPQFYVRTTDVTGTIMVMPGDRIKMSAELINAIAIEG
MRFAIREGGRTVGAGVVSKII
>YP_009546031.1_Glaucocystis_incrassata
FERKPHVNIGTIGHVDHGKTTLTAAITTALAANGKKARKYDEIDAAPEEKARGITINT
AHVEYETDKRHYAHVDCPGHADYVKNMITGAAQMDGAILVVSAADGPMPQTREHI
LLAKQVGVPNMVVFLNKEDIIDDPDILELVELEVRELLSKYDFPGDEVPFVTGSALIAL
EADKIHALMDAVDDYIATPERDVDKSFLMAIEDVFSITGRGTVATGRIERGQIKVGDT
VELVGLTRNTTVTGLEMFQKTLGLAGDNIGILLRGVQKTDIERGMVLAKPGSITPHT
QFEAEVYVLKKDEGGRHTPFFTGYRPQFYVRTTDVTGRIMVMPGDRIKMNVTLVHP
IAIEGMRFAIREGGRTVGAGVVSKIL
>YP_009545402.1_Mallomonas_splendens
FERKPHINIGTIGHVDHGKTTLTAAITATLST-N-
AVKKYEDIDAAPEERARGITINTAHVEYETEVRHYAHVDCPGHADYIKNMITGAAQM
DGAILVVSAVDGAMPQTREHILLARQVGVKKLVVFLNKADQVDDPELLELVELELRE
LLQSYDYPGDEIPFVPGSALLALEADKIFELMDAVDSYIPTPEREVDKPFLLAIEDVFSI
TGRGTVATGRIERGTVNVGDTVELVGLTKTTTVTGLEMFQKTLGIAGDNIGILLRGV
QKTDVLRGMVLSKPGSINPHTKFEAEVYILTKDEGGRHTSIFKGYRPQFYVRTTDVT
GQIMVMPGDNVNMKIELIAPIAIEGMRLAIREGGRTVGSGLVTKII
>YP_009540906.1_Lepocinclis_tripteris
FERKPHVNIGTIGHVDHGKTTLTAAITMALAAKGNKAKKYDEIDSSPEEKARGITINT
AHVEYETEKRHYAHVDCPGHADYVKNMITGAAQMDGAILVVSGADGPMPQTKEHI
LLAKQVGVPNIVVFLNKEDQVDDKELLELVELEIRETLNNYEFPGDEIPIVSGSALLSV
EADKILELMDQVDSYIPTPSRDVDKDFLLAVEDVFSITGRGTVATGRIERGKVKVGET
VELVGLTRSTTVTGLEMFQKSLALAGDNVGILLRGIQKNDVERGMVIAKPGTIKPHT
KFDSQVYILKKEEGGRHTPFFEGYRPQFYVRTTDVTGKIMVMPGDRIKMEVELIQPIA
IEGMRFAIREGGRTVGAGVVLTII
>YP_009510502.1_Crassa_caudata
FERKPHVNIGTIGHVDHGKTTLTAAISATLAAANDKAKKFDEIDAAPEEKARGITINTA
HVEYETQNRHYAHVDCPGHADYVKNMITGAAQMDGAILVVSAADGPMPQTREHIL

LAKQVGVPNIVVFLNKQDQVDDEELLELVELEVRELLVQYDFPGEDIPFVAGSALLAL
EKDKIHSLMDAVDEYIPTPVRDVEKTFLMAVEDVFSITGRGTVATGRIERGVIKVGDT
IEIVGLTATTTITGLEMFQKTLGMAGDNIGILLRGIQKKDIERGMVLAQPGTITPHTQ
FEAEVYILTKEEGGRHTPFFSGYRPQFYVRTTDVTGTIMVMPGDRIKMSAELINPIAIE
GMRFAIREGGKTVGAGVVSKIL
>YP_009505158.1_Endarachne_binghamiae
YDRKPHINIGTIGHVDHGKTTLTAAITAVLSLSGDNAKKYEDIDAAPEERARGITINTA
HVEYETETRHYAHVDCPGHADYVKNMITGAAQMDGAILVVSAADGPMPQTREHILL
SKQVGVPHIVVFLNKEDQVDDLELVELVELEVRELLSNYDFPGDDIPIVTGSALQALD
ADKIYSLMESVDSYIPTPVRDVDKSFLMAIEDVFSITGRGTVATGKIDRGMIKVGETVD
LVGLTKSTTVTGVEMFQKTLGVAGDNVGILLRGLQKSEIERGMVLAKPGTITPHNTF
ESELYILTKEEGGRHTPFFPGYRPQFYVRTTDVTGEIMVMPGDRVKMTAKLISLIAIEG
MRFAIREGGRTIGAGVVSKII
>YP_009504709.1_Cyanophora_biloba
FERKPHVNIGTIGHVDHGKTTLTAAITTALASQGKKARKYDEIDAAPEEKARGITINT
AHVEYETDARHYAHVDCPGHADYVKNMITGAAQMDGAILVVSAADGPMPQTREHI
LLAKQVGVPNMVVFLNKEDQIDDADLLELVELEVRELLNKYDFPGDQIPFVSGSALL
ALESDKILALMDAVDEYIPTPERPIDKSFLMAIEDVFSITGRGTVATGRIERGQIKVGET
VELVGLTKSTTVTGLEMFQKTLGMAGDNIGILLRGVQKIDIERGMVLAKPGSITPHT
QFESEVYVLTKDEGGRHTPFFSGYRPQFYVRTTDVTGSIMVMPGDRIKMTVSLVHPI
AIEGMRFAIREGGRTIGAGVVSKIL
>YP_009502090.1_Porolithon_onkodes
FERKPHVNIGTIGHVDHGKTTLTAAISATLAAANNTAKKFDEIDAAPEEKARGITINTA
HVEYETKNRHYAHVDCPGHADYVKNMITGAAQMDGAILVVSAADGPMPQTREHIL
LAKQVGVPNIVVFLNKKDQIDDEELLELVELEVRELLTQYDFPGDNIPFVAGSALLAL
DEDKIHELMGAVDDYIPTPERDVDKTFLMAVEDVFSITGRGTVATGRIERGIIKVGDT
IEIVGLTRSTTITGLEMFQKTLGMAGDNIGILLRGIQKNDIERGMVLAQPNTITPHTQ
FEAEVYILTKEEGGRHTPFFSGYRPQFYVRTTDVTGTIMVMPGDRIKMNAELINAIAIE
GMRFAIREGGRTVGAGVVSKII
>YP_009496017.1_Rhizosolenia_setigera
FERKPHVNIGTIGHVDHGKTTLTAAITATLALNGSTVKDYSDIDSAPEERARGITINTA
HVEYETETRHYAHVDCPGHADYVKNMITGAAQMDGAILVVSAADGPMPQTREHILL
AKQVGVPDIVVFLNKEDQVDDAELLELVELEVRELLSSYDFPGDDIPICPGSALQALE
ADKIFALMDAVDEYIPTPERDTEKTFLMAVEDVFSITGRGTVATGRIERGIVKVGDTIE
IVGVTKSTTITGIEMFQKTLGFAGDNVGILLRGVTREDIERGMVLSQPGTITPHTSFES
EVYVLTKEEGGRHTPFFTGYRPQFYVRTTDVTGSIMVMPGDRIKMTAELIYPVAIEG

MRFAIREGGRTIGAGVVSKIV
>YP_009478314.1_Prototheca_cutis
FERKPHINIGTIGHVDHGKTTLTAAISMALAA-G-
KGKKYDDIDSAPEEKARGITINTAHVEYETKVRHYAHVDCPGHADYVKNMITGAAQ
MDGAILVVSGADGPMPQTKEHILLAKQVGVPHIVVFINKEDQVDDVELLELVELEVR
EILEHNNFPGEDTPMITGSALLALNADKIYQLMDIVDSYIPTPKRETEKPFLMAIEDVF
SITGRGTVATGRVERGTIKIGDSVEIIGFTKTSIVTGLEMFQKTLSIAGDNVGILLRGIQ
KKDIQRGMVLAKPNSITPHTSFEAQVYVLKKDEGGREKPFFAGYRPQFYLRTTDVTG
QIMIAPGDYIKLTVELIQPIAIENMRFAIREGGKTIGAGVVGRII
>YP_009459250.1_Chrysochromulina_parva
FARKPHINIGTIGHVDHGKTTLTAAISATLAIYNRLAKKISEIDAAPEEKARGITINTAHI
EYETAGRHYAHVDCPGHADYVKNMITGAAQMDGGILVVSAADGPMPQTREHILLA
KQVGVPYLVVFLNKVDQVDDEELLELVKLEVQELLESYDFPGGEIPFVSGSALLALQA
DLIFALLDAVDNYIPTPERDTEKKFLMAIEDVFSITGRGTVATGRIERGQVNVGDTVE
LVGLTRSTTVTGIEMFQKTLGMAGDNVGILIRGIQKTEIERGMVLALPGSITPHKKFE
AEVYVLSKEEGGRHTPFFTGYRPQFYVRTTDVTGTIMVMPGDRIKMTTELLNPIAIEG
MRFAIREGGRTVGAGVVSKIL
>YP_009455853.1_Dictyopteris_divaricata
FDRKPHLNIGTIGHVDHGKTTLTAAITAVLSLVGDTAKKYEDIDAAPEERARGITINT
AHVEYETEVRHYAHVDCPGHADYVKNMITGAAQMDGAILVVSAADGPMPQTREHIL
LSKQVGVPHIVVFLNKEDQVDDVELIELVELEVRELLSNYDFPGDDIPIVAGSALQALD
ADKIYNLMESVDNYIPTPVRDIDKAFLMAIEDVFSITGRGTVATGKIDRGIIKVGETVE
LVGLTKSTTVTGVEMFQKTLGVAGDNVGILLRGLQKNEIQRGMVLSKPGTITPHNK
FESEVYILTKEEGGRHTPFFTGYRPQFYVRTTDVTGQIMVMPGDRIKMTAGLISLIAIE
GMRFAIREGGRTIGAGVVSKII
>YP_009420350.1_Cryptomonas_curvata
FERKPHVNIGTIGHVDHGKTTLTAAISATLASYTGITKKFDEIDSAPEERARGITINTAH
IEYETEKRHYAHVDCPGHADYVKNMITGAAQMDGAILVCSAADGPMPQTREHILLA
KQVGVPHIVVFLNKADMVDDAELLELVQLEVQELLSKYDFPGDEIPFVSGSALLALEA
DTIYELMDKVDSYIPTPEREVDKSFLMAVEDVFSITGRGTVATGRVERGQVKVGDTIE
IVGLTRNTTITGLEMFQKSLALAGDNVGILLRGIQKVDIERGMVLSKPGSITPHTKFEG
EVYVLTKEEGGRHTPFFTGYRPQFYVRTTDVTGTIMVMPGDRIKMTAQLIHPIAIEG
MRFAIREGGRTVGAGVVSKII
>YP_009367613.1_Pseudocharacium_americanum
FERKPHVNIGTIGHVDHGKTTLTAAITMALAAQTGKAKRYDEIDSAPEEKARGITINT
AHVEYETEKRHYAHVDCPGHADYVKNMITGAAQMDGAILVVSGADGPMPQTKEHI

LLAKQVGVPNIVVFLNKEDQVDDPELLELVELEVRETLENYEFPGDEIPIVSGSALLAL
EADKIYDLMDQVDSYIPTPVRENDKPFLMGVEDVFSITGRGTVATGRVERGTIKVGD
SIEVVGLTRTTTVTGLEMFQKTLCVAGDNVGVLLRGIQKTDIERGMVLAQPGTITPH
TKFEAQVYVLTKEEGGRHTAFFPGYRPQFYVRTTDVTGKIMVMPGDRVKMVVELIQ
PIAIEEMRFAIREGGRTVGAGVVSNIL
>YP_009330470.1_Coccophora_langsdorfii
FDRKPHINIGTIGHVDHGKTTLTAAITAVLSLSGSNAKKYEDIDAAPEERARGITINTA
HVEYETETRHYAHVDCPGHADYVKNMITGAAQMDGAILVVSAADGPMPQTREHLL
LSKQVGVPHIVVFLNKEDQVDDLELIELVELEVRELLSNYEFPGDDIPIVAGSALQALE
ADKIYNLMEEVDNYIPTPIRDTEKTFLMAIEDVFSITGRGTVATGKIDRGIIKVGETVEL
VGLTKSTTVTGVEMFQKTLGVAGDNVGILLRGLQKTEIERGMVLSKPGTITPHNTFE
SEVYVLTKEEGGRHTPFFVGYRPQFYVRTTDVTGEIMVMPGDRVKMTAGLISLIAIEG
MRFAIREGGRTIGAGVVSKIL
>YP_009327062.1_Pleurocladia_lacustris
YDRKPHINIGTIGHVDHGKTTLTAAITAVLSLSNGTLKKYEDIDAAPEERARGITINTA
HVEYETEARHYAHVDCPGHADYVKNMITGAAQMDGAILVVSAADGPMPQTREHILL
SKQVGVPHIVVFLNKEDQVDDLELVELVELEVRELLSNYDFPGDDIPIITGSALQALD
ADKIYSLMDSVDNYIPTPIRDIEKSFLMAIEDVFSITGRGTVATGKIDRGIVKVGESVDL
VGLTKSTTVTGVEMFQKTLGVAGDNVGILLRGVQKSEIERGMVLAKPGTITPHNTFE
SELYILTKEEGGRHTPFFPGYRPQFYVRTTDVTGEIMVMPGDRVKMTAKLISLIAIEG
MRFAIREGGRTIGAGVVSKII
>YP_009297871.1_Kumanoa_americana
FERKPHVNIGTIGHVDHGKTTLTAAISATLAALGNKLKKFDEIDAAPEEKARGITINTA
HVEYETNNRHYAHVDCPGHADYVKNMITGAAQMDGAILVVSAADGPMPQTREHIL
LAKQVGVPNIVVFLNKEDQVDDKELLELVQLEVTELLGQYDFPGEEIPFVSGSALLAL
NQDKIYDLMDAIDSYIPTPVRDIDKTFLMAVEDVFSITGRGTVATGRIERGIIKVGDSIE
IVGLTRTTTITGLEMFQKTLGMAGDNIGILLRGIQKKDIERGMVLAQPGTITPHTEFE
AEVYILTQEEGGRHTPFFSGYRPQFYVRTTDVTGTIMVMPGDRIKMSAQLINPIAIEG
MRFAIREGGRTVGAGVVSKIL
>YP_009295921.1_Schimmelmannia_schousboei
FERKPHVNIGTIGHVDHGKTTLTAAISATLAASSDKAKKFDEIDAAPEEKARGITINTA
HVEYETDNRHYAHVDCPGHADYVKNMITGAAQMDGAILVVSAADGPMPQTREHIL
LAKQVGVPNIVVFLNKEDQVDDEEILELVELEVRELLGQYDFPGDDIPFVAGSALLAL
EEDKIHELMDSVDDYIPTPIRDVEKTFLMAVEDVFSITGRGTVATGRIERGIIKVGDTI
EIVGLTITTTITGLEMFQKTLGMAGDNIGILLRGLQKKDIERGMVLAQPGTITPHTQF
EAEVYILTKEEGGRHTPFFSGYRPQFYVRTTDVTGTIMVMPGDRIKMSAQLINPIAIEG

MRFAIREGGKTVGAGVVSKIL
>YP_009240476.1_Lotharella_vacuolata
FERKPHVNIGTIGHVDHGKTTLTAAITMALSV-G-
KQKKYDDIDSAPEEKARGITINTSHVEYETKNRHYAHVDCPGHADYVKNMITGAAQ
MDGAILVVSGADGPMPQTKEHLLLAKQVGVPSIVVFLNKEDQVDDEELLELVELEIR
EMLDTYDFPGESTPIIKGSALMALQSEKILSLMQNVDDYIPTPDRETQKPFLMAVEDV
FSITGRGTVATGRVERGVVKIGDIVEIVGLTRSTTVTGLEMFQKMLSIAGDNVGMLLR
GIQKADIQRGMVIAEPGSITPHHSFEAQVYILTKEEGGRHKPFFKGYRPQFYFGTTDV
TGNIMVVPGDRVTMSVELVQPIAIEGMRFAIREGGRTVGAGVVSNIA
>YP_009240430.1_Partenskyella_glossopodia
FERKPHVNIGTIGHVDHGKTTLTAAITMALSS-G-
KQKKYDDIDSAPEEKARGITINTAHVEYETESRHYAHVDCPGHADYVKNMITGAAQM
DGAILVVSGADGPMPQTKEHLLLAKQVGVPSIVVFLNKEDQVDDEELLELVELEIRE
MLDTYDFPGDSIPIIKGSALMALQVDKILSLMDSVDDYIPTPDRETEKPFLMAVEDVF
SITGRGTVATGRVERGGVKIGDLVEIVGLTRSTTVTGLEMFQKMLSIAGDNVGMLLR
GIQKADIQRGMVIAKPGSITPHVSFEAQVYVLTKEEGGRHTPFFKGYRPQFYVRTTDV
TGKVMVVPGDRVSMSVELVQPIAIEGMRFAIREGGRTVGAGVVSTVI
>YP_009240355.1_Gymnochlora_stellata
FERKPHVNIGTIGHVDHGKTTLTAAITMALST-G-
KQKKYDDIDSAPEEKARGITINTAHVEYETETRHYAHVDCPGHADYVKNMITGAAQ
MDGAILVVSGADGPMPQTKEHLLLAKQVGVPSIVVFLNKEDQVDDEELLELVELEVR
EMLDNYDFPGDDTPIITGSALLALQADKILTLMDNVDEYIPTPERETEKPFLMAVEDV
FSITGRGTVATGRVERGGIKIGDTVEIVGLTRSTTVTGLEMFQKMLSIAGDNVGMLLR
GIQKTDIQRGMVIAQPGSITPHVSFEAQVYVLTKEEGGRHTPFFSGYRPQFYVRTTDV
TGKVMVVPGDRVTMSVELVQPIAIEGMRFAIREGGRTVGAGVVSNVI
>YP_009237383.1_Wildemania_schizophylla
FERKPHVNIGTIGHVDHGKTTLTAAISATLSTLGSSAKRFDEIDAAPEEKARGITINTA
HVEYETDNRHYAHVDCPGHADYVKNMITGAAQMDGAILVVSAADGPMPQTREHIL
LAKQVGVPTLVVFLNKEDQVDDEELLELVELEGRELLSQYDFPGDDVPFVAGSALLA
LEADKIFSLMEAVDTYIPTPERDVDKTFLMAVEDVFSITGRGTVATGRIERGIIKVGDT
IEIVGLTRTTTITGLEMFQKTLGLAGDNIGILLRGVQKKDIERGMVLAKPGTITPHTK
FEAEVYILTKEEGGRHTPFFPGYRPQFYVRTTDVTGTIMVMPGDRIKMTAELINAIAIE
GMRFAIREGGRTVGAGVVSKIL
>YP_009220389.1_Ulva_fasciata
FERKPHVNIGTIGHVDHGKTTLTAAITMALQKFSGTGKKYDEIDSAPEEKARGITINT
AHVEYETENRHYAHVDCPGHADYVKNMITGAAQMDGAILVVSGADGPMPQTKEHL

LLAKQVGVPSIVVFLNKEDQVDDPELLELVQLEVQETLEAYEFPSDEVPIVTGSALLAL
EA-
KIYELMKEVDSYIPTPERETDKTFLMAVEDVFSITGRGTVATGRVERGVLKTNETVDL
VGLTKNVTVTGLEMFQKTLTVAGDNVGVLLRGVQKDEIQRGMVIAAPNSIEPHTKF
EAQVYVLTKEEGGRHTPFFPGYRPQFYVRTTDVTGKIMVIPGDRVKMVVELIQPIAIE
NMRFAIREGGRTVGAGVVSKIL
>YP_009182597.1_Undaria_pinnatifida
YDRKPHINIGTIGHVDHGKTTLTAAITAVLSLSGDNAKKYEDIDAAPEERARGITINTA
HVEYETASRHYAHVDCPGHADYVKNMITGAAQMDGAILVVSAADGPMPQTREHILL
SKQVGVPHIVVFLNKEDQVDDLELVELVELEVRELLSNYDFPGDDIPILTGSALQALD
ADKIYNLMESVDSYIPTPIRDVDKPFLMAIEDVFSITGRGTVATGKIDRGIVKVGETVD
LVGLTKSTTVTGVEMFQKTLGVAGDNVGILLRGLQKGDIERGMVLSKPGTITPHNT
FESELYILTKEEGGRHTPFFPGYRPQFYVRTTDVTGEIMVMPGDRVKMTAKLISLIAIE
GMRFAIREGGRTIGAGVVSKII
>YP_009163677.1_Triparma_laevis
FERKPHVNIGTIGHVDHGKTTLTAAITATLSLGGTISKAYDEIDAAPEEKARGITINTA
HVEYETETRHYAHVDCPGHADYVKNMITGAAQMDGAILVVSAADGPMPQTREHILL
SKQVGVPDIVVFLNKEDQVDDAELLELVELEVRELLSNYDFPGDDIPIRAGSALQAIEA
DKIHALMDAVDEYIPTPERDVEKTFLMAIEDVFSITGRGTVATGRVERGVLKVGDTV
EIVGITQSTTITGIEMFQKTLAFAGDNCGILLRGVTREDIERGMVVSQPGTITPHTNFE
SEVYVLTKEEGGRHTPFFTGYRPQFYVRTTDVTGSIMVMPGDRIKMTAELIYPVAIEG
MRFAIREGGRTIGAGVVSKIS
>YP_009162667.1_Pseudonitzschia_multiseries
FERKPHVNIGTIGHVDHGKTTLTAAITATLSLEGGAIKDYADIDGAPEERARGITINTA
HVEYETQTRHYAHVDCPGHADYVKNMITGAAQMDGAILVVSAADGPMPQTREHIL
LSKQVGVPDIVVFLNKQDQVDDDELLELVELEVRELLSAYDFPGDDIPICPGSALQAI
EADKIFALMDAVDEYIPTPERDTEKTFLMAIEDVFSITGRGTVATGRIERGVVKVGEN
VEIVGVTQTTTITGIEMFQKTLGFAGDNVGILLRGVTREDIERGMVLSQPGTITPHTV
FESEVYVLTNDEGGRSTPFFTGYRPQFYVRTTDVTGAIMVLPGDRIKMTAELIYPVAIE
GMRFAIREGGRTIGAGVVSKIV
>YP_009145429.1_Cryptoglena_skujai
FERKPHINIGTIGHVDHGKTTLTAAITMALAATGKKAKRYEDIDSAPEEKARGITINTA
HVEYETDKRHYAHVDCPGHADYVKNMITGAAQMDGAILVVSGADGPMPQTKEHIL
LAKQVGVPNIVVFLNKEDQVDDKELLELVEMEVRETLNNYEFPGDEIPVVSGSALLSV
EADKILDLMDQVDSYIPTPKRDTDKDFLMAIEDVFSITGRGTVATGRVERGTVKVGE
TVELVGLTKTTTVTGLEMFQKSLALAGDNVGVLLRGVQKTDIERGMVISKPGTINPH

TKFNSQVYILTKEEGGRHTPFFEGYRPQFYVRTTDVTGKIMVMPGDRIKMQVELIQPI
AIEGMRFAIREGGRTVGAGIVLDII
>YP_009122059.1_Choreocolax_polysiphoniae
FKRKPHINIGTIGHVDHGKTTLTAAISAILAVN--
VVKKIDEIDAAPEEKTRGITINTTHIEYETNKRHYAHIDCPGHSDYIKNMITGAAQMD
AAILVVSAIDGAMPQTREHILLAKQVGVPNIIVFLNKQDQIEDNELHELVELEVRELLE
KYDFPGESTPFITGSALLALEEDKIYSLMDVIDLYIQVPKRDIEKNFLMAVEDVFSITGR
GTVATGKIERGTIKVGDIIEIIGLTKKTTITGLEMFNKKLGIAGDNIGILLRGIQKLDIHR
GMVLAQPNTITPHTQFEAEVYILTKEEGGRHTPFFVGYKPQFYVRTTDVTGIIMVIPG
DRIKIKAKLIHPIAIESMKFAIREGGKTVGAGIISKIL
>YP_009059298.1_Eunotia_naegelii
FERKPHVNIGTIGHVDHGKTTLTAAITATLALDGNEFKAYEDIDGAPEERARGITINT
AHVEYETDKRHYAHVDCPGHADYVKNMITGAAQMDGAILVVSAADGPMPQTREHI
LLAKQVGVPDIVVFLNKEDQVDDPELLELVELEVRELLSAYEFPGDDIPICPGSALQAI
EADKIYALMDAVDEYIPTPIRDIEKTFLMAIEDVFSITGRGTVATGRIERGIIKVGETVEI
VGITKTTTVTGIEMFQKTLGFAGDNVGILLRGVTRENIERGMVLAKPGTITPHTSFES
EVYILTKDEGGRHTPFFTGYRPQFYVRTTDVTGAIMVMPGDRIKMTAELIYPVAIEG
MRFAIREGGRTIGAGVVSKIV
>YP_009028820.1_Asterionellopsis_glacialis
FERKPHVNIGTIGHVDHGKTTLTAAITATLSLDGTVAAAVADIDGAPEERARGITINT
AHVEYETANRHYAHVDCPGHADYVKNMITGAAQMDGAILVVSAADGPMPQTREHI
LLSKQVGVPDIVVFLNKEDQVDDAELIELVELEIRELLSDYDFPGDDIPICPGSALQAIE
ADKIYALMDAVDEYIPTPERDTEKSFLMAIEDVFSITGRGTVATGRIERGIIKVGETVEI
VGVTQTTTITGVEMFQKTLGFAGDNVGILLRGVTREDIERGMVLAKPGTITPHTNFE
AEVYVLTKDEGGRHTPFFTGYRPQFYVRTTDVTGAIMVMPGDRIKMTAELIYPVAIE
GMRFAIREGGRTIGAGVVSKII
>YP_009019373.1_Auxenochlorella_protothecoides
FERKPHVNIGTIGHVDHGKTTLTAAITMALAARDGKGKKYDDIDSAPEEKARGITINT
AHVEYETENRHYAHVDCPGHADYVKNMITGAAQMDGAILVVSGADGPMPQTKEHI
LLAKQVGVPNIVVFINKEDQVDDIELIELVELEVRETLQRYDFPGDEVPMLPGSALMA
LSADKIYKLMDTVDSYIPTPKRNVEKPFLMAIEDVFSITGRGTVATGRVERGVVKIGD
TVEIIGFTKVTTVTGLEMFQKTLSMAGDNVGILLRGIQKADIQRGMVLAKPKSIKPHT
SFEAQVYVLTKDEGGRDTPFFKGYRPQFYVRTTDVTGKIMVLPGDRIQMTVELIQPI
AIENMRFAIREGGKTVGAGVVVKIL
>YP_008145452.1_Phaeocystis_globosa
FERKPHINIGTIGHVDHGKTTLTAAISATLAISTEKSKKFDEIDSAPEEKARGITINTAH

VEYETETRHYAHVDCPGHADYVKNMITGAAQMDGAILVVSAADGPMPQTREHILLA
KQVGVPHLVIFLNKADQVDDEELLELVELEVQELLENYDFPGDEIPFVSGSALMALQA
DLIFKLMDSIDEYIPTPIRDTEKTFLMAIEDVFSITGRGTVTTGRIERGVIKVGDTVDIV
GLTQTTTVTGIEMFQKTLGQAGDNVGILIRGIQKTDVERGMVMAQPGSINPHKKFE
AEVYILGKDEGGRHTPFFTGYRPQFYVRTTDVTGTIMVMPGDRIKMTAELINPIAIEG
MRFAIREGGRTVGAGVVSKIL
>YP_005090128.1_Fucus_vesiculosus
FDRKPHINIGTIGHVDHGKTTLTAAITAVLSLDGSNAKKYEDIDAAPEERARGITINTA
HVEYETETRHYAHVDCPGHADYVKNMITGAAQMDGAILVVSAADGPMPQTREHLL
LSKQVGVPHIVVFLNKEDQVDDLELIELVELEVRELLSNYEFPGDDTPIVAGSALQALE
ADKIYNLMEEVDKYIPTPIRDTDKTFLMAIEDVFSITGRGTVATGKIDRGIVKVGETVE
LVGLTKSTTVTGVEMFQKTLGVAGDNVGILLRGLQKTEIERGMVLSKPGTITPHNTF
ESEVYVLTKEEGGRHTPFFVGYRPQFYVRTTDVTGEIMVMPGDRVKMTAGLISLIAIE
GMRFAIREGGRTIGAGVVSKIL
>YP_004376606.1_Fistulifera_solaris
FERKPHVNIGTIGHVDHGKTTLTAAITSTLSLEGQ--
KAYDEIDGAPEERQRGITINTAHVEYETETRHYAHVDCPGHADYVKNMITGAAQMD
GAILVVSAADGPMPQTREHILLSKQVGVPHIVVFLNKEDQVDDAELLELVELEVRELL
SAYDFPGEEIPICPGSALQAMEADKIFALMDAVDSYIPTPERDTEKTFLMAVEDVFSIT
GRGTVATGRIDRGIVKVGESVEIVGITKTTTVTGIEMFQKTLGFAGDNVGILLRGVTR
DDIERGMVLAKPGTITPHTNFESEVYVLTKEEGGRHTPFFSGYRPQFYVRTTDVTGAI
MVMPGDRIKMTAELIHPVAIEGMRFAIREGGRTIGAGVVSKIV
>YP_004347788.1_Chlorella_variabilis
FERKPHVNIGTIGHVDHGKTTLTAAITMALAARGGKGRKYDDIDSAPEEKARGITINT
AHVEYETEKRHYAHVDCPGHADYVKNMITGAAQMDGAILVVSGADGPMPQTKEHL
LLAKQVGVPNIVVFLNKEDQVDDAELLELVELEIRETLDKYEFPGDEIPIIAGSALLALE
ADKIYNLMDQVDSYIPTPERETEKPFLMAIEDVFSITGRGTVATGRVERGCVKIGDTV
ELVGLTKTTTVTGLEMFQKTLSVAGDNVGILLRGVQKIDIERGMVLAKPGSIKPHTK
FEAQVYVLTKEEGGRHTPFFPGYRPQFYVRTTDVTGKIMVMPGDRIKMIVELIQPIAI
EGMRFAIREGGRTVGAGVVSTIV
>YP_004072649.1_Thalassiosira_oceanica_CCMP1005
FERKPHVNIGTIGHVDHGKTTLTAAITATLSLEGDVAKDYADIDGAPEERARGITINT
AHVEYETKDRHYAHVDCPGHADYVKNMITGAAQMDGAILVVSAADGPMPQTREHI
LLAKQVGVPHIVVFLNKQDQVDDDELLELVELEVRELLSAYDFPGDDIPICPGSALQA
IEADKIYALMDSVDAYIPTPERDVEKTFLMAIEDVFSITGRGTVATGRIERGVVKVGDN
VEIVGVTQTTTITGIEMFQKTLGFAGDNVGILLRGVTREDIERGMVLSQPGTITPHTN

FESEVYVLTKEEGGRHTPFFTGYRPQFYVRTTDVTGSIMVMPGDRIKMTAELIYPVAI
EGMRFAIREGGRTIGAGVVSKIV
>YP_003795289.1_Chromera_velia
FDRKPHLNIGTIGHVDHGKTTLTAAIATILSRGTKAARSYAEIDSAPEEKARGITINTA
HVEYETELRHYAHVDCPGHADYIKNMITGAAQMDGAILVVAATDGIMPQTTEHLLL
ARQVNVPYIVCFLNKEDLLDDPELLEIVEAELQEELEKYQFSTD-
VPFVSGSALKALEYDRIIQLMNVVDEYIKTPERDVTKPLLLSIESACSVTGRGTVVTGKI
DRGRVVTGQTVNLLGFKKSVTITGLEMFRKTLALAGDDVGALLRGVQLKEVKRGMV
LASPKTLFSSATFIGSVLIISTTDGGRSKPFNVGYKPQFYLRTADCTGRVFALPGSSYYL
FIEFATKMPLEGLQFAIREGGITVGAGQIVQVA
>YP_003735074.1_Durinskia_baltica
FERKPHVNIGTIGHVDHGKTTLTAAITATLALESGVVKGYADIDAAPEERARGITINTA
HVEYETSNRHYAHVDCPGHADYVKNMITGAAQMDGAILVVSAADGPMPQTREHILL
SKQVGVPDIVVFLNKQDQVDDDELLELVELEVRELLSAYDFPGDDIPICPGSALQAIE
ADKIFALMDAVDEYIPTPERDTEKTFLMAIEDVFSITGRGTVATGRIERGVIKVGDSVE
IVGITKTTTITGIEMFQKTLGFAGDNVGILLRGVTRENIERGMVLAKPGTITPHTNFES
EVYVLTSEEGGRRTPFFTGYRPQFYVRTTDVTGAIMVMPGDRIKMTSELIYPVAIEGM
RFAIREGGRTIGAGVVSKIV
>YP_003734620.1_Kryptoperidinium_foliaceum
FERKPHVNIGTIGHVDHGKTTLTAAITATLALETGSVKEYADIDGAPEERARGITINTA
HVEYETANRHYAHVDCPGHADYVKNMITGAAQMDGAILVVSAADGPMPQTREHIL
LSKQVGVPNIVVFLNKQDQVDDDELLELVELEVRELLSAYDFPGDDIPICPGSALQAIE
ADKIFALMDAVDDYIPTPERDTEKTFLMAIEDVFSITGRGTVATGRIERGVVKVGDNV
EIVGITKSTTITGIEMFQKTLGFAGDNVGILLRGVTREDIERGMVLAKPGTITPHTDFE
SEVYVLTSDEGGRRTPFFTGYRPQFYVRTTDVTGAIMVMPGDRIKMTSELIYPVAIEG
MRFAIREGGRTIGAGVVSKIV
>YP_003359277.1_Cryptomonas_paramecium
FERKPHVNIGTIGHVDHGKTTLTAAISATLAIYTGITKKFDEIDSAPEERARGITINTAH
IEYETEKRHYAHVDCPGHADYVKNMITGAAQMDGAILVCSAADGPMPQTREHILLA
KQVGVPHVVVFLNKVDMVDDSELLELVQLEIQELLSKYDFPGDKIPFVSGSALLALEA
DTIYNLMDKIDAYIPTPEREIDKNFLMAVEDVFSITGRGTVATGRIERGKVKLGETIEIV
GLTRTTTITGLEMFQKSLAIAGDNVGILLRGIQKVDIERGMVLSKPGSITPHTKFEGEV
YVLTKEEGGRHTPFFTGYRPQFYVRTTDVTGTIMVMPGDRIKMTAQLIHPIAIEGMR
FAIREGGKTVGAGIVSQIL
>YP_003289178.1_Ectocarpus_siliculosus
YDRKPHINIGTIGHVDHGKTTLTAAITAVLALAGDNAKKYEDIDAAPEERARGITINT

AHVEYETATRHYAHVDCPGHADYVKNMITGAAQMDGAILVVSAADGPMPQTREHI
LLSKQVGVPHIVVFLNKEDQVDDLELVELVELEVRELLSNYDFPGDDIPIVTGSALQA
LDADKIYSLMDSVDNYIPTPVRDIDKAFLMAIEDVFSITGRGTVATGKIDRGMVKVGE
TVDLVGLTKSTTVTGVEMFQKTLGFAGDNVGILLRGIQKGEIERGMVLAKPGTITPH
NTFESELYILTKEEGGRHTPFFPGYRPQFYVRTTDVTGEIMVMPGDRVKMTAKLISLI
AIEGMRFAIREGGRTIGAGVVSKII
>YP_003002211.1_Aureoumbra_lagunensis
FERKPHINIGTIGHVDHGKTTLTAAITMTLALAGGTAKNYEDIDAAPEERARGITINT
AHVEYETEKRHYAHVDCPGHADYVKNMITGAAQMDGAILVCSAADGPMPQTREHIL
LSKQVGVPHIVVFLNKEDQVDDEELLELVELEVRELLSNYDFPGDDIPCVSGSALMAI
EADKIFALMDAVDEYIPTPVRDTEKTFLMAIEDAFSITGRGTVATGRIERGIVKVGETV
EIVGLTRQTTVTGIEMFQKTLGFAGDNVGLLLRGIQKTDIQRGMVLAKEGSITPHTE
FEAEVYILTKEEGGRHTPFFTGYRPQFYVRTTDVTGSIMVMPGDRIKMTAELISAIAVE
GMRFAIREGGRTIGAGVVSKIV
>YP_003002071.1_Aureococcus_anophagefferens
FERKPHINIGTIGHVDHGKTTLTAAITMTLALGTDAAKNYEDIDAAPEERARGITINT
AHVEYETENRHYAHVDCPGHADYVKNMITGAAQMDGAILVCSAADGPMPQTREHI
LLSKQVGVPHIVVFLNKEDQVDDDELLELVELEVRELLSSYDFPGDDIPCVAGSALMA
IEADKIYGLMDAVDDYIPTPVRDTEKTFLMAIEDAFSITGRGTVSTGRIERGTVKVGET
VEIVGLTRETTVTGIEMFQKTLGLAGDNVGLLLRGIQKTDIERGMVLAASGTITPHTL
FEAEVYILTKEEGGRHTPFFTGYRPQFYVRTTDVTGNIMVMPGDRIKMTAELISAIAIE
GMRFAIREGGRTIGAGVVSKIV
>YP_002600853.1_Pycnococcus_provasolii
FERKPHVNIGTIGHVDHGKTTLTAAITMAMSALSGGGKNYDEIDSAPEEKARGITINT
AHVEYETETRHYAHVDCPGHADYVKNMITGAAQMDGAILVVSGADGPMPQTKEHI
LLAKQVGVPNIVVFLNKEDQVDDPELLELVELEIRETLSNYDFPGDDLPIVSGSALLAL
EADRIFNLMDEVDSYIPTPERETDKMFLMAVEDVFSITGRGTVATGRVERGVLKVGD
TIEIVGLTQTTTVTGLEMFQKTLTLAGDNVGVLLRGVQKEDIERGMVLAAPGTITPH
TRFESQVYILTKEEGGRHTPFFPGYRPQFYVRTTDVTGKIMVMPGDHIKMTVELIQPI
AIEQMRFAIREGGRTVGAGVVSQII
>YP_001936420.1_Heterosigma_akashiwo
FERKPHVNIGTIGHVDHGKTTLTAAITATLSLGGTQLKNYEDIDAAPEERARGITINT
AHVEYETDNRHYAHVDCPGHADYVKNMITGAAQMDGAILVVSAADGPMPQTREHI
LLAKQVGVPHIVVFLNKEDQVDDEELLGLVELEVRDLLSNYDFPGDDIPCIPGSALQA
LQADKIYQLMEAVDEYIPAPERDVDKTFLMAVEDVFSITGRGTVATGRIERGVVKVGE
TVQIVGLTRETTVTGIEMFQKTLGMAGDNVGILLRGVQKEDIERGMVLAKPGTINPH

TNFESEVYILRKEEGGRHTPFFAGYRPQFYVRTTDVTGKIMVVPGDRIKMTAELISPIA
IEGMRFAIREGGRTIGAGVVSKIL
>YP_001293607.1_Rhodomonas_salina
FERKPHVNIGTIGHVDHGKTTLTAAISTVLAANSSPGKKFDEIDSAPEERARGITINTA
HVEYETAARHYAHVDCPGHADYVKNMITGAAQMDGAILVCSAADGPMPQTREHILL
AKQVGVPHIVVFLNKADMVDDEELLELVELEVQELLSKYDFPGDEIPFVAGSALLALE
TDTIFELMDKIDEYIPTPERETDKSFLMAVEDVFSITGRGTVATGRIERGQVKVGDTIE
IIGLTRTTTITGLEMFQKSLALAGDNVGILVRGIQKTDIERGMVLAAPGSITPHTKFEG
EVYVLTKEEGGRHTPFFTGYRPQFYVRTTDVTGTIMVMPGDRIKMTAQLIHPIAIEG
MRFAIREGGRTVGAGVVSKIL
>YP_001019139.1_Chlorokybus_atmophyticus
FERKPHVNIGTIGHVDHGKTTLTAAITMALAASTGKGKRYDEIDAAPEERARGITINT
AHVEYETEKRHYAHVDCPGHADYVKNMITGAAQMDGAILVVSGADGPMPQTKEHI
LLAKQVGVPNVVVFLNKEDQVDDAELLELVELEVRETLSDYDFPGDEVPVVAGSALL
ALESDKIYSLMDQVDAYIPTPERDTDKPFLMAVEDVFSITGRGTVATGRVERGTVKV
GEAIEIVGLAPVTSVTGLEMFQKTLSVAGDNVGILLRGIQKKDIERGMVLAKPGTIKP
HKSFEAQVYILNKEEGGRHTPFFQGYRPQFYVRTTDVTGKIMVMPGDRIKMVVQLIQ
PIAIEGMRFAIREGGRTVGAGVVFNIL
>XP_726726.1_Plasmodium_yoelii
FERKPHMNIGTIGHVDHGKTTLTAAITKVCSKYDRTFKSYEDIDKTPEEQKRGITINA
THVEYETEKRHYSHIDCPGHLDYIKNMITGTSQMDGSILVVSAYDGLMPQTKEHVLL
SRQIGINKIIVYLNKIDMCEDQELVDLVELEVRELLSFHKYDGDNIPFIKGSALKALNDP
SILKLLDACDNYIDEPKRKIDLPFLMSIDDVLQISGKGTVATGRVEQGTIKINESVDILG
ISIKTVITGIEMFRKTLAQAGDQIGVMLKNVKKNDISRGMVVTKIPNMKTYKKFESDIY
VLKNEEGGRKNPFSSYYRPQVYIRTADVNCAVIANPGDNIKCTIELMYPLAISGLRFSL
REGGKTVASGIITKVL
>XP_029214602.1_Besnoitia_besnoiti
FKKKPHINIGTIGHVDHGKTTLTAAITYVLAKQNQKVKTYNEIDCAPEEIARGITIKTS
HIEYETNTRHYAHIDCPGHADYIKNMITGAAQMDGAILVVSAVDGPMPQTKEHLLLA
KQIGISNIIVFLNKIDLIDDNEILELVELETRELLDKYNFSSDTTPVITGSALKALDSNKIY
ELLTALDSYIPLPKRDLDKPFLLAIEDIFSITGRGTVVTGKIERGCIKLGDTVTLLGFSK
NVVIIGLEMFQKTLGEAGDNVGILLRGIQKTDVKRGMILAKPSTMTLHSIFQADVYIL
TVAEGGREKPIFEGYCPQFYLYTINITGSIMILPGDRVKLTVTLIYSIAIEGIRFAIREGGR
TIGAGIITDII
>XP_026189596.1_Cyclospora_cayetanensis
FKKKTHLNIGTIGHVDHGKTTLTAAITSYLSKINNKAKSYSEIDSAPEEKARGITINTSH

VEYETSLRHFAHIDCPGHADYIKNMITGAAQMDGAILVVSATDGPMPQTREHLLLAK
QVGVPSIIVFLNKVDMVEDNELLELVELEIRELLDTYEFNGNSVKIIQGSALKALEANN
LKELIDALDISIIEPTRDLNKPFLLPIEDIFSITGRGTVVTGKIERGSIKINDTVNILGFSKI
ATVIGIEMFQKTLAEAGDNVGILLRGIQKEEVRRGMVLALPSSILTYCNFEADIYILSAS
EGGRKTPFFEGYKPQFYFYTTDVTGTIMILPGDKIKLKVTLMYSIALEGMRFAIREGG
KTIGAGIITNLI
>XP_024449891.1_Populus_trichocarpa
FTRKPHVNVGTIGHVDHGKTTLTAAITKVLAEEGKKAIAFDEIDKAPEEKKRGITIATA
HVEYETAKRHYAHVDCPGHADYVKNMITGAAQMDGGILVVSAADGPMPQTKEHIL
LARQVGVPSLVCFLNKVDVVSDPELIELVEMEVRELLSFYKFPGDEIPIVRGSALSALQ
GKAILKLMDAVDEYIPDPVRQLEKPFLMPIEDVFSIQGRGTVATGRVEQGTIKVGEEV
EILGLPKRTTVTGVEMFKKLLGQAGDNVGLLLRGLKREDVQRGQVIAKPGTVKTYK
RFEAEIYSLTKDEGGRHTAFFSNYRPQFYLRTADITGKVMVMPGDNVTAIFELILPVPL
EGQRFALREGGRTVGAGVVSKVL
>XP_021860808.1_Spinacia_oleracea

MITGAAQMDGGILVVSAPDGPMPQTKEHILLARQVGVPSLVCFLNKVDAVDDPELL
ELVEMELRELLSFYKFPGDDIPIVRGSALSALQGQAILKLMDAVDEYIPDPVRVLDKPF
LMPVEDVFSIQGRGTVVTGRVEQGMIKVGEDVEILGLPVKTTVTGVEMFKKLLGQA
GDNVGLLLRGLKREDVQRGQVVAKPGSCKTFKKFEGEIYVLTKDEGGRHTHFVTNY
KPQFYLRTADVTGSIMVLPGDNVSATFELIAPVPLEGQRFAFREGGRTVGAGVVTKVI
>XP_021835381.1_Spinacia_oleracea
FERKPHVNIGTIGHVDHGKTTLTAALTMALASMGNAPKKYDEIDAAPEERARGITIN
TATVEYETENRHYAHVDCPGHADYVKNMITGAAQMDGAILVCSGADGPMPQTKEH
ILLAKQVGVPNMVVFLNKQDQVDDEELLQLVELEVRELLSNYDFPGDDIPIISGSALL
ALEEDKIYQLMDAVDEYIPIPTRQTDLPFLMAIEDVFSITGRGTVATGRVERGTVKTG
ETVDIVGMTRNTTVTGVEMFQKILALAGDNVGLLLRGIQKIDIQRGMVLAKPGTITP
HTKFSAIVYVLKKEEGGRHSPFFVKYRPQFYMRTTDVTGEVMVMPGDRVKMEVTLI
HPVACEGMRFAIREGGKTVGAGVIQSII
>XP_021744471.1_Chenopodium_quinoa
FTRKPHVNVGTIGHVDHGKTTLTAAITKVLSEEGNKAVAFDEIDKAPEEKKRGITIAT
AHVEYETKKRHYAHVDCPGHADYVKNMITGAAQMDGGILVVSAPDGPMPQTKEHI
LLARQVGVPSLVCFLNKVDAVDDPELIELVEMELRELLSFYKFPGDDIPIIRGSALSALQ
GQAILKLMDAVDDYIPDPVRVLDKPFLMPVEDVFSIQGRGTVVTGRVEQGMIKVGE
DVEILGLPVKTTVTGVEMFKKILGQAGDNVGLLLRGLKREDVQRGQVVAKPGSCKT
FKRFEAEIYVLTKDEGGRHTAFVSNYRPQFYLRTADVTGKIMVLPGDNVTATFELISP

VPLEGQRFALREGGRTVGAGVVSKVI
>XP_013935876.1_Ogataea_parapolymorpha
FDRKPHVNIGTIGHVDHGKTTLTAAITKVLSENGGSFMDYSAIDKAPEERARGITISTA
HVEYETPNRHYSHVDCPGHQDYIKNMITGAAQMDGAIIVVAATDGQMPQTREHLLL
ARQVGVQKLVVFVNKVDTIDDPEMLELVEMEMRELLSSYGFDGDETPVIMGSALCAL
EGQAIEKLMNAVDEYIPTPVRDLEQPFLLPIDEVFSISGRGTVVSGTVERGVLKKGEEV
EIVGGPLKTTVTGIEMYHKELAMAGDTPGILLRGMKREQIQRGMILAKVGSLKSYKKF
LASMYILTKEEGGRHTPFSENYRPQMFVRTTNVSVTLQVMPGDNVEMVCEMLHPVA
LEGQRFNLRESGKTVGTGMITRLY
>XP_009691215.1_Theileria_orientalis_strain_Shintoku
FVRKEHLNIGTIGHVDHGKTTLTAALTKVCSLSGVEYVPYDSIDKAPEERKRGITICAT
HVEYETDKRHYGHVDCPGHADYIKNMISGAAQMDGAILVVSAPDGPMPQTREHILL
AKQIGVPKLVVYLNKMDLVEDPEIVELVELEINELLSEYNY-GDS-
PIVKGSATKALMNNSIKQLLAKCDEYLATPDRKEDLPLLIAVDEVLAIPGKGTVATGRI
EQGRVKVGDAVEIVGGAKKSTVNSLEMFRKTLGIAGDQVGFLLKNVKRDDIARGYVV
SCPGTYFSYDSFDADLYVLTHEEGGRKNGFGSNYRPQAFLRTGDVSCTVLAMPGDSL
KCTIKLLNHMPLHGLRFALREGGKTVASGIISKLH
>XP_009530113.1_Phytophthora_sojae_mitochondrial
FDRKPHLNVGTIGHVDHGKTTLTAAITKVLSETGGEFTSYADIDKAPEERARGITISTA
HVEYETAKRHYAHVDCPGHADYVKNMITGAAQMDGGILVVSAGDGPMPQTREHIL
LARQVGVPALVVFMNKVDQVDDEELLELVEMEIRELLEAYDFPADDIPVVKGSALAA
VEGDAVLQLMDEVDAYIPDPVRDFEKPFLMPVEDVFSISGRGTVVSGRVEQGVINTG
DEVELVGLSTKTTCTGVEMFKKSLGQAGDNVGLLLRGLKRDEVLRGQVLCKPGTIN
PHTKFEAEVYVLKKEEGGRHTPFFSNYRPQFFFRTADVTGNIMVMPGDNTAIDIELIH
PIALDGMKFSIREGGRTIGAGVISKVD
>XP_008872050.1_Aphanomyces_invadans
FERKPHLNVGTIGHVDHGKTTLTAAITKVLSEQGGKFVAFEDIDKAPEERARGITISTS
HVEYETENRHYAHVDCPGHADYVKNMITGAAQMDGGILVVSATDGPMPQTREHIL
LSRQVGVPALVVFLNKVDMVDDEELLELVEMEIRELLDSYDFPGDDIPVVRGSALQA
VQGDAILELMKQVDEYIPEPVRDFDRPFLLPVEDVFSISGRGTVVSGRIETGVIKPGDE
VELVGITSKSTCTGVEMFRKSLGQAGDNVGVLLRGLKREDVLRGQVLCKPGSINAHT
KVTAQVYVLKKEEGGRHTPFFNNYRPQFFFRTADITGNVMVMPGDNLTLNIELIHPV
AIDGMKFSIREGGRTIGAGVVATVV
>XP_008611190.1_Saprolegnia_diclina_VS20
FDRKPHLNVGTIGHVDHGKTTLTAAITRVLSEQGGKFIAFEDIDKAPEERARGITISTS
HVEYETEKRHYAHVDCPGHADYVKNMITGAAQMDGGILVVSATDGPMPQTREHILL

ARQVGVPALVVFLNKVDMVDDEELLELVEMEIRELLDVYDFPGDDIPIVRGSALAAVE
GDAVIELMNQVDEYIPDPTRDFEKPFLLPVEDVFSISGRGTVVSGRVETGVINTGDEV
ELVGIPTKTTCTGVEMFRKSLGQAGDNVGVLLRGLKRDDVLRGQVLCKPGSISPHTK
VEAGVYVLKKEEGGRHTPFFTNYRPQFFFRTADITGNIMVMPGDNPTLRIELIHPVAI
DGMKFSIREGGRTIGAGVVSKII
>XP_005836302.1_Guillardia_theta_CCMP2712
YERKPHLNIGTIGHVDHGKTTLTAAITRVMAEQNKKAMAFDDIDKAPEEKARGITIEA
AHVEYETEKRHYAHVDCPGHADYVKNMITGAAQMEGAILVVSAPDGPMPQTREHIL
LARQVGVPAMVVFLNKCDAVDDPELIDLVEMEVRELLTFYKFPGDEIPIVRGSALHAL
NGDKIVELMKAVDDYIPEPARDMDKPFLMPVEDIFSIAGRGTVVTGRIETGTIKVGED
IEIIGIPVKTTCIGVEMFKKELGQAGDNCGVLLRGIKREDVTRGQVLAKPGSAKTATK
FSAEIYCLSKEEGGRHTPFFKKYKPQFFFRTADVTGTIMVMPGDNVSCTVELICPIVME
GLQFAIREGGKTVGAGVVSEIL
>XP_005774315.1_Emiliania_huxleyi_CCMP1516
WNRKPHINIGTIGHVDHGKTTLTAAITKA--

SASMDGAILVVSAPDGPMPQTREHVLLAKQVGLPRMVVFMNKCDMADDEELLELVE
MEVRELLQLHDFPADDTPFVRGSALCALE----------
VDDFIPTPQRETDKAFLMPIEDTFSIAGRGTVVTGRVESGKIKTGEEAEIVGLTISTTV
TGVEMFHKNLGEAGENVGCLLRGLKRDDVQRGQVLCKPGTVKPVTKFKASVYALTK
EEGGRHTPFFTNYRPQFFFRTAD--
>XP_005719137.1_Chondrus_crispus
FQRKPHLNVGTIGHVDHGKTTLTAAITKVLSEAGGTFTDYDQIDKAPEEKKRGITIST
AHVEYETPKRHYAHVDCPGHADYVKNMITGAAQMDGAILVVSAADGPMPQTREHIL
LARQVGVPHIVVFLNKCDVVDDEELIELVEMEVRDLLSFYKFPGDDTPIVRGSALEAL
QGDAVLKLMGEVDEYIPEPKRDLDKPFLMSVEDVFSIQGRGTVVTGRVEQGTIKAGQ
DVEVVGLTIKVACTGVEMFKKSLGQAGDNLGCLLRGIKREDVTRGQVLCAPGSIKSH
NKFEAEIYCLTKEEGGRHKPFFTNYAPQFYFRTADVTGKVMVMPGDNVTCKAEMLS
KIAMVGLRFAVREGGRTIGAGVVTKVM
>XP_005704149.1_Galdieria_sulphuraria
FERKPHINVGTIGHVDHGKTTLTAAITKVLSESGAKFMDYDQIDKAPEEKARGITIASS
VVEYETAKRHYAHVDCPGHADYVKNMITGAAMMDGAILVVSAADGPMPQTREHILL
ARQVGVPHIVVYLNKCDLVSDPELVELVEMEVRELLSFYDFKGEEAPIIRGSALLALKG
KSIIQLMDAVDETIPEPVRALDKPFLLSIEETFSIQGRGTVVTGRVEQGVIKVGDEVEIV
GLPMKTTVIGVETFKKSLGQAGDNVGCLLRGVKREDVRRGQILCKPGSIKPHKKFEA
QMYVLKKEEGGRHTAFFSKYRPQFFFRTADVTGTVMAMPGDNLTSVTELFEPVAME

GLRFAVREGGRTVGAGVVSKVL
>XP_004994191.1_Salpingoeca_rosetta
FVRKPHVNIGTIGHVDHGKTTLTAAITKVLSEEGHQYTDYSNIDKAPEERVRGITISTA
HVEYETDKRHYAHVDCPGHADYIKNMITGAAQMDGAILVVSATDGQMPQTREHILL
AKQVGVERIVVYINKADMVDDEELLELVEMEIRELLSSYGYDGDETPVVTGSALCAIE
GDSIKALMNAVDEWIPDPERDLDKPFLMPVENAFSISGRGTVVTGKVERGVINKGDE
VEIIGYTIKTTVTGVEMFHKQLGQAGDNLGALCRGLKREEIRKGQVMCKPGTVKSHT
KFQTQLYVLSKEEGGRHTPFVDGYRPQLFTRTGDITCTVMVMPGEDASCEIELITDIP
LEGQRFTVREGHKTVGTGIVSKII
>XP_004336955.1_Acanthamoeba_castellanii
TYRKPHFNICTIGHVDHGKTTLTAAITKVLADTGLKFRDYQSIDKAPEEIRRGITINAS
HVEYETDKRHYGHVDNPGHAEFIKNMITGTSLTDGAILVVDCSTGPMPQTREHILLA
RQVGVKNIVVWLNKCDLIPDKELQDMVAMEIREELTRYEYNGDSTPIIHGSALEAIEL
QAIRKLLATVDE-
LPQPERQTDKPFLMAIEALYTISGRGTVATGVVEQGKVKIGDEVEVLGMKIKGVVTGI
ETFHKQMGFAGDSIGLLLRGPNRDDLKRGQVVGKPGTLSAHKKFEANVYVLTKEEG
GRHTPFGAGYAPQMFFRTANVTGKVVAVPGETMNVGFETIWPMPLSGLKFSCREGG
MTVAAGVITKVF
>XP_004030017.1_Ichthyophthirius_multifiliis
FVRKPHLNVGTIGHIDHGKTTLTAAITKILAQKQQTFMEYSQIDKAPEEKARGITINT
ATVEYETETRHYGHVDCPGHIDYVKNMITGAAKMDAGILVCSATDGVMPQTREHIL
LCRQVGVNTIIVFVNKCDVAKDSEIQELVEMEVRELLAKYEYDGEKSPVIFGSALCALN
GDKVVQLLKIMDEQIPIPQRLTDKPFLMSIEGTYQIAGRGTVVTGTVDAGKVKVGED
VDVAGYINRTTITGIETFRKQLAEAGDNVGLLLRGATRDDVRRGQVLCKPNSQQIYK
KVEVNLYILTDAEGGRKKPFPNGYRPQFYLRTADVAAEIVGMPGDNITADLDLHFPL
PVSGQRFALREGGKTIAAGIVTKAM
>XP_003882789.1_Neospora_caninum
FQRKPHLNIGTIGHVDHGKTTLTAAITKVLADLGQDFKSYAEIDKSPEEQKRGITINA
THVEYETGKRHYGHVDCPGHADYVKNMITGAAQMDGAILVVSAYDGPMPQTREHI
LLSKQVGVPRLVVYLNKMDMVEDQELVELVEMEVRELLSFYDFPGDDTPFVKGSALK
ALNGQTIKDLMQACDDFIPEPERKADLPLVIPVESVLSIPGKGTVATGRVEQGTAKM
NEAVEIVGGPLKAQIAALEMFRKTLAQAGDQVGCLLKGIKRDEVKRGMVLGAPGYLK
TFKKFEADLYVLKEEEGGRKKPFFSHYRPQAFIRTGDMACTIMAMPGDRVTCTVELL
HPIALHGLRFALREGGRTVASGIVTKPV
>XP_002958279.1_Volvox_carteri
NSRKPHLNVGTIGHVDHGKTTLTAAITKVLSETNGKAVSYDQIDKAPEEKARGITINS

THVEYQTTNRHYAHVDCPGHADYVKNMITGAAQMDGAILVVSAADGPMPQTREHI
LLAKQVGVPRIVVFLNKCDVVEDKELQELVEMEVRELLNFYKFPGDEVPVIRGSALSA
LKGNSIMKLMQAVDEYITVPPRVTDKPFQMPVEDIFSIAGRGTVLTGRIEQGVIKPGE
DVEIVGLAIKSTVTGVEMFKRSLGQAGDNVGLLIRGIKREDVSRGQVVCKVGSLKTYK
RFEAEVYALTKEEGGRHTPFTSKYKPQFFIRTADVSGQIMVMPGDNFRATIQLSAPTA
LEGLRFAIRDSGKTVGAGVVAKVI
>XP_002773196.1_Perkinsus_marinus_ATCC_50983

GHVDHGKTTLTAAITKILSDKGLSFQTYEAIDRAPEEKRRGITINQTHVEYSTEKRHY
GHVDCPGHADYVKNMITGAAQVDGAILVVSAFDGPMPQTREHILLAKQIGVPRLVV
FLNKMDQVDDPELVELVELEVRELLEFYKYPGEETPFVKGSALKALRGEAILKLMDAV
DEYIPEPPRLQDKPFLLPIETVVNIQGKGYVVTGRIEQGLVKVGDALEIVGQKFKSQC
MGVEMFHKTLGMAGDQCGVMLKGVKKNQIRRGMVLTKPGAAKTYTEFESDLYVLK
EDEGGRKNPFHSEYRPQAYIRTGDCSCRIMAMPGDSIKATLKLDRPQAVEGLRFALRE
GGKTVASGLITACN
>XP_002524809.1_Ricinus_communis
FTRKPHVNVGTIGHVDHGKTTLTAAITKVLAEEGKKAVAFDEIDKAPEEKKRGITIAT
AHVEYETAKRHYAHVDCPGHADYVKNMITGAAQMDGGILVVSAPDGPMPQTKEHI
LLARQVGVPSLVCFLNKCDAVDDPELLELVEMELRELLSFYKFPGDEIPIIRGSALSAL
QGKAILKLMDAVDEYIPDPVRQLDKPFLMPIEDVFSIQGRGTVATGRVEQGTIKVGEE
VEILGLPLKTTVTGVEMFKKILGQAGDNVGLLLRGLKREDVQRGQVIAKPGSVKTYK
KFEAEIYVLTKDEGGRHTAFFSNYRPQFYMRTADITGKVMVMPGDNVTATFELILPV
PLEGQRFALREGGRTVGAGVVSKVI
>XP_002293181.1_Thalassiosira_pseudonana
FNRKPHVNIGTIGHVDHGKTTLTQAITKVLSEKGWKAMSYEDIDRAPEEKARKITINT
SHIEYETANRHYGHIDCPGHADYVKNMITGAAQMDGGILVVAATDGPMPQTREHIL
LAKQVGMPKLVVFLNKCDMVDDEELLELVEMEIRELLDFYDFNGDETPIIRGSALAA
AEGNAVLELMAAVDETIPEPTRDLDKPFLMPIEDVFSIAGRGTVVTGRIEQGKVNVG
DDLEVVGFNAKTTCTGVEMFKKLLGMAGDNVGALLRGLKREDVERGQVLCKPGSIS
TAKKFEAEIYCLSQDEGGRHTPFFSNYRPQFFFRTADVTGDLMVMPGDNTTLDVELI
TPVPIEGLRFNMREGGRTVGTGIVTKVH
>XP_002183867.1_Phaeodactylum_tricornutum
FSRKPHVNIGTIGHVDHGKTTLTQAITKVLSEKGWQAMTYEQIDKAPEEKARKITINT
SHIEYETANRHYGHIDCPGHADYVKNMITGAAQMDGGILVVAATDGPMPQTREHIL
LAKQVGIPNLVVFLNKVDLVDDEELLELVEMEIRELLDFYDFPGDDIPIIRGSALAAAE
GEKILELMAAVDEKIPEPMRDLDKDFLMPIEDVFSIAGRGTVVTGRVQQGKVNVGDE

LDVIGLNHKTICTGVEMFKKLLGMAGDNIGALLRGLKREDVRRGQILCKPGSMKTSK
KFEAEVYALKKDEGGRHTPFMTNYRPQFFFRTADITGSLMVMPGDNTTVDVELISPV
ALEGLRFNMREGGMTVGTGIVTKVS
>XP_001021714.2_Tetrahymena_thermophila
FQRKPHLNVGTIGHIDHGKTTLTAAITKICADKKLEFMAYDSIDKAPEEKARGITINTA
TVEYETETRHYGHVDCPGHIDYVKNMITGAAKMDAGILVCSATDGVMPQTREHILL
CRQVGVKTIIVFVNKCDMAKDPEIQELVEMEVRELLSKYEYNGDEAPVIFGSALCALN
GNKINTLLDTMDKQIALPERTVDKPFMMSVEGTYQIPGRGTVVTGTVDTGKVKTGE
DIEIVGYVLKTTITGIETFRKQLAEAGDNVGLLLRGVTRDDVRRGQVLSKPGTQESHK
KIEANLYILTEQEGGRKKPFPDGYRPQL--
>SCM08864.1_Plasmodium_chabaudi
FERKPHMNIGTIGHVDHGKTTLTAAITKVCSKYDRTFKSYEDIDKTPEEQKRGITINA
THVEYETEKRHYSHIDCPGHLDYIKNMITGTSQMDGSILVVSAYDGLMPQTKEHVLL
SRQIGINKIIVYLNKIDMCEDQELVDLVELEVRELLSFHKYDGDNIPFIKGSALKALNDP
SILKLLDACDNYIDEPQRKIDLPFLMSIDDVLQISGKGTVATGRVEQGTIKINEPVDIL
GIPIKTVITGIEMFRKTLAQAGDQIGVMLKNIKKNDISRGMVVTKVPNMKTYKKFESD
IYVLKNEEGGRKNPFSSYYRPQVYIRTADVNCAVIANPGDNIKCTIELMYPLAVSGLRF
SLREGGKTVASGIITKVL
>QGT77023.1_Micractinium_pusillum
FERKPHVNIGTIGHVDHGKTTLTAAITMALAARGGKGRKYADIDSAPEEKARGITINT
AHVEYETENRHYAHVDCPGHADYVKNMITGAAQMDGAILVVSGADGPMPQTKEHL
LLAKQVGVPNIVVFLNKEDQVDDAELLELVELEIRETLDKYEFPGDEIPIIAGSALLALE
ADKIYNLMDQVDSYIPTPARETEKPFLMAIEDVFSITGRGTVATGRVERGCVKIGDTV
ELIGLTKTITVTGLEMFQKTLSVAGDNVGILLRGVQKLDIERGMVLAKPGSIKPHTKF
EAQVYVLTKEEGGRHTPFFPGYRPQFYVRTTDVTGKIMVMPGDRIKMIVELIQPIAIE
GMRFAIREGGRTVGAGVVSTIV
>QGR23553.1_Skeletonema_pseudocostatum
FERKPHVNIGTIGHVDHGKTTLTAAITATLALDGDVAKDFADIDGAPEERARGITINT
SHVEYQTATRHYAHVDCPGHADYVKNMITGAAQMDGAILVVSAADGPMPQTREHI
LLARQVGVPHIVVFLNKEDQVDDEELLELVELEVRELLSVYDFPGDDIPIKPGSALQAI
EADKIFALMDAVDEYIPTPERDVEKTFLMAIEDVFSITGRGTVATGRIERGVVKVGET
VEIVGVTQTTTITGIEMFQKTLGFAGDNVGILLRGVTREDIERGMVLAKPGTIAPHTN
FESEVYILTKDEGGRHTPFFTGYRPQFYVRTTDVTGSIMVMPGDRIKMTAELIYPVAIE
GMRFAIREGGRTIGAGVVSKIV
>QDZ18217.1_Chloropicon_primus
NARKPHMNVGTIGHVDHGKTTLTAAITRVLAEAGQEQVAFDAIDKAPEEKARGITIS

TAHVEYETENRHYAHVDCPGHADYVKNMITGAAQMDGGILVVSATDGPMPQTREH
ILLARQVGVPSLVCFLNKVDAVDDEELIDLVEMELRELLSFYKYPGDDIPIVRGSALAA
LEGNAVLELMKNIDEYMPAPERALDKPFAMPVEDVFSIQGRGTVVTGRIEQGIIKVGE
EVEIIGIPTKTTVTGVEMFKKSLGQAGDNCGLLLRGIKREDVTRGQVIVKPGSMKTAK
EFQGEIYILTKDEGGRHTPFFNGYRPQFFLRTADITGTVMVMPGDNVTATFNLIAPV
PVQGLKFAIREGGRTVGAGVIVGDK
>Q9TJQ8.1_Prototheca_wickerhamii
FERKPHVNIGTIGHVDHGKTTLTAAITMALAA-G-
KGKKYAEIDSAPEEKARGITINTAHVEYETESRHYAHVDCPGHADYVKNMITGAAQM
DGAILVVSGADGPMPQTKEHILLAKQVGVPNIVVFINKEDQVDDIELIELVELEVRETL
QRYDFPGDEVPMIPGSALMALTADKIYNLMDIVDSYIPTPKRNIEKPFLMAIEDVFSIT
GRGTVATGRVERGVVKIGDSVEIVGLTKITTVTGLEMFQKTLSIAGDNVGILLRGIQK
TEIQRGMVLAKPKSITPHTNFEAQVYVLNKEEGGRDTPFFSGYRPQFYVRTTDVTGKI
MVLPGDRIKMKAELIQPIAIENMRFAIREGGKTVGAGVVGKIL
>Q2EEV7.2_Helicosporidium_sp.
FERKPHINIGTIGHVDHGKTTLTAAITMALAS-G-
KGKNYADIDSAPEEKARGITINTTHVEYETAKRHYAHVDCPGHADYIKNMITGAAQM
DGAILVVSGADGPMPQTREHIVLAKQVGVPSMVVFINKEDQVDDPEILELVELEVRD
LLTSYKFEGEEVPVITGSALLALEADKIYNLMDSVDSYIPTPVREIDKPFLMAIEDVFSIS
GRGTVATGRIERGKIKMGDSIEIIGG-
KTTTVTGIEMFQKTLGVAGDNVGILMRGIQKKEIDRGMVLTKPKSIDPLTSFEAQVY
LLTKEEGGRSKGFTIGYRPQFYVRTTDVTGAIIASPGDRITMSVKLIQPIALENMRFAIR
EGGKTVGAGVVSKLI
>PXF48959.1_Gracilariopsis_chorda
FQRKPHINVGTIGHVDHGKTTLTAAITKVLAETGGTFTDYEQIDKAPEEKQRGITIST
AHVEYETTKRHYAHVDCPGHADYVKNMITGAAQMDGAILVVSAADGPMPQTREHI
LLARQVGVPHIVVFLNKCDMVDDEELIELVEMEVRELLSFYKFPGDDIPVVKGSALQA
LQGDAILKLMSNVDDYIPEPQRALDKPFLMSVEDVFSIQGRGTVVTGRVEQGTIKAG
QEVEIVGLTIKTACTGVEMFKKSLGQAGDNLGCLLRGIKRDDVHRGQVLCEPGTIRP
YSKFEAEVYCLTKEEGGRHKPFFSNYSPQFYVRTADVTGKFMVMPGDNVTCKAEILS
KVAMAGLRFAVREGGRTIGAGVVTKLI
>Pteridomonas3_mito
YERLPHMNIGTIGHVDHGKTTLTAAISKVLGEAGCAAQKYEDIDKAPEEKARGITINS
AHVEYSTQNRHYAHVDCPGHADYVKNMIMGAAQMDGGILVVSATDGPMPQTREHI
LLARQVGIPSLVVFLNKCDMVDDEELLELVEMEVRELLDKYDFPGDDTPIIRGSALAA
ATGQAVLKLMETVDSYIPEPVRLLDKPFVMPIEGVHSIPGRGTVCTGRVDQGIVKVG

DELEIFGLTTKTVCTGVEMFKKSLGRAGDNLGLLLRGIKREEVFRGQVVAVPGGQEV
GEEFEAELYVLTKDEGGRHTPIFSKYRPQFFTRTSDITGEIMIMPGDNATLSVKLIYPV
AIEGQKFSLREGGKTVGHGLVTKVK
>Pteridomonas2

AGDNVGVLLRGITRNDIRRGHVLAAPNTIKPYTTFESETYILTKEEGGRHTPFFTGYQ
PQFYVRTTDVTGKIMVLPGDRIKMTVKLGSPIAIQGSRFAVREGGKTIGAGVVTKIL
>Pteridomonas_PT

AGDNVGLLLRGIQKHEVRRGMVLAKPGTIIAYQEFIGETYILTKEEGGRHTPFFVGYR
PQFYVRTTDVTGRIMVLPGDRIKMTVELVAPIAIEGSRYAIREGGKTIGAGIVTKII
>Pteridomonas_NY
LSKKIHLNIGTIGHVDHGKTTLTAAITAVLSTQGTICKTYQDIDSAPEEKLRGITINTS
HVEYETELRHYAHVDCPGHADYIKNMITGAAQMDGGILVVSALDGPMPQTREHILL
AKQVGVPTLVIFLNKEDQIDDIEILELVELELRELLISYDFPGNDLPLISGSALLALEADK
IFMLVDFIDSYIQVPTRDIEKPFLMGVEDVFSITGRGTVATGRIERGKILNSEIMEIVGL
TFTVTIIGIEMFQKLLGSAGDNVYFISKIVKS--

>Pteridomonas_danica_strain_PT_Mito
YERLPHMNIGTIGHVDHGKTTLTAAISKVLGEAGCAAQKYEDIDKAPEEKARGITINS
AHVEYSTQNRHYAHVDCPGHADYVKNMIMGAAQMDGGILVVSATDGPMPQTREHI
LLARQVGIPSLVVFLNKCDMVDDEELLELVEMEVRELLDKYDFPGDDTPIIRGSALAA
ATGQAVLKLMETVDSYIPEPVRLLDKPFVMPIEGVHSIPGRGTVCTGRVDQGIVKVG
DELEIFGLTTKTVCTGVEMFKKSLGRAGDNLGLLLRGIKREEVFRGQVVAVPGGQEV
GEEFEAELYVLTKDEGGRHTPIFSKYRPQFFTRTSDITGEIMIMPGDNATLSVKLIYPV
AIEGQKFSLREGGKTVGHGLVTKV-
>Pteridomonas
TGKKEHINVGTIGHVDHGKTTLSAAITAVLSLKGLTPRNFEEIDCSPEERTRGITISTS
HLEYETETRHYAHVDCPGHADYIKNMITGTAQMDGGILVVSALDGPMPQTREHILL
AKQVGVPRLIVFLNKEDQVEDEEMLELIEMELRELLSDYGFPGDEIPICPGSALMALN
ADKIFDFMDKIDEYIPLPKRALDKPFLLAVEDVFSVTGRGTVATGLIESGEVKTGDHM
EVVGFPLPTIATGIEMF--

>PNW81763.1_Chlamydomonas_reinhardtii
NSRKPHLNVGTIGHVDHGKTTLTAAITKVLAETGGKVVAYDQIDKAPEEKARGITIN
ATHVEYQTEKRHYAHVDCPGHADYVKNMITGAAQMDGAILVVSAADGPMPQTREH
ILLAKQVGVPRIVVFLNKCDVVEDKELQELVEMEVRELLSFYKFPGDEIPVVRGSALAA
IKGASILKLMQAVDDYVLVPERATDKPFQMPIEDVFSIAGRGTVVTGRIEQGIIKAGED
IEIVGLTIKSTVTGVEMFKKSLGQAGDNVGLLVRGIKREDVSRGQVMAKPGSIKTYKQ
FEAEVYALTKEEGGRHTPFTTKYKPQFFIRTADVAGQIMVMPGDNFRATIDLSAPVAL
EGLRFAIRDSSKTVGAGVVTKCV
>OSX71741.1_Porphyra_umbilicalis
FERKKHANIGSIGHVDHGKTTLTSAITKVLSETGGTFVDYGNIDKAPEEKARGITISTA
HVEYETANRHYAHVDCPGHADYVKNMITGAAQMDGAILVVSAVDGPMPQTREHIL
LARQVGVPHIVVFLNKVDMVDDSELTDLVEMEVRDLLTFYKFPGDDIPIIRGSALKA
MKGDAVLSLMAAVDEYIPNPTRDLDRPFLMPVEDVFSIPGRGTVVTGRVERGIVKPG
EDVEVLGLPIKTTVTGVEMFKKSLGEAGDNVGCLLRGLKRDDVTRGQVLCKPASVK
TYTKFEAEVYCLTKEEGGRHKPFFSNYQPQFYIRTGDVTGTFMVMPGDNVTASVELL
TPIAMDGLRFAVREGGRTVAAGVVSKLV
>OLQ02676.1_Symbiodinium_microadriaticum
VRQKDHINIGTIGHVDHGKTTLSAAISLVCGQFDDSKKSYEEIDNAPEERARGITINAS
HIEYETESRHYCHVDCPGHADYVKNMITGACQMDGGILVISSPDGPMAQTREHILLS
KQVGVPALVCFMNKVDMMDDEELLELVELETREMLSQYGFPGDDTPFIQGSALQAL
EQDKILELMETVDEYIPTPERETDKPFLLAVEDVFSISGRGTVCTGRVEQGVVKKGDE
VEILGRPQKSVITGIRMFNTDLGPAGYTVGVLCRGIDKDAVFRGQVICAPGATKTHT
KFKANIYFAKKDEGGRSNPVMPGYMPVFYFRTCDVTGKIMAMPGDNITCTCELIAGT
PIEGMRFAMREGGRTIGQGLIEETM
>OEH75581.1_Cyclospora_cayetanensis
FERKPHMNIGTIGHVDHGKTTLTAAITKVLSTRGKEFKSYAEIDKSPEEQKRGITINAT
HVEYETANRHYGHVDCPGHADYVKNMITGAAQMDGAILVVSAYDGPMPQTREHIL
LSKQVGVPRLVVYLNKMDMAEDMELVDLVEMEVRELLSFYEFPGDDTPFIRGSALKA
LNGDSVLKLMEACDAFIPEPERKADLPLILPVEQVLSIPGKGTVLTGRIEQGTLKGGE
AVEIVGVPIKAQIAALEMFKKTLARAGDQVGALVKGVKRDDIKRGMVVGAPGYLKTY
KKFSADIYVLKEDEGGRKKPFFSYYKPHAFIRTGDMACTIMAMPGDSVQVEVELLHPV
ALQGLRFALREGGKTVASGLVTRPI
>NP_849096.1_Cyanidioschyzon_merolae
FERKPHVNIGTIGHVDHGKTTLTAAISAVLASKDNQLKKFEEIDSAPEERARGITINTS
HVEYQTEKRHYAHVDCPGHADYVKNMITGAAQMDGAILVVSAADGPMPQTREHIL

LAKQVGVPSIVVFLNKADMVDDPELLELVELEVRELLSKYDFPGDTIPFVTGSALLALE
ADKIFELMKIVDEYIPTPQRDVDKSFLMAVEDVFSITGRGTVATGRIERGRVKVGETIE
IVGLTKTTTVTGLEMFQKTLGIAGDNVGVLLRGVQKTDIERGMVLAKPGSITPHTKF
EAEVYVLTKEEGGRHTPFFPGYRPQFYVRTTDVTGTIMVMPGDRIKMCVNLIYPVAIE
GMRFAIREGGRTVGAGVVTKIL
>NP_776632.1_Bos_taurus
YVRKPHVNVGTIGHVDHGKTTLTAAITKILAEGGGKFKKYEEIDNAPEERARGITINA
AHVEYSTAARHYAHTDCPGHADYVKNMITGTAPLDGCILVVAANDGPMPQTREHLL
LARQIGVEHVVVYVNKADAVQDSEMVELVELEIRELLTEFGYKGEETPIIVGSALCALE
QKSVQKLLDAVDTYIPVPTRDLEKPFLLPVESVYSIPGRGTVVTGTLERGILKKGDECE
FLGHNIRTVVTGIEMFHKSLAEAGDNLGALVRGLKREDLRRGLVMAKPGSIQPHQKV
EAQVYILTKEEGGRHKPFVSHFMPVMFSLTWDMACRILAMPGEDLKLTLILRQPMIL
EGQRFTLRDGNRTIGTGLVTDTP
>NP_074999.1_Euglena_longa
FERKPHINIGTIGHVDHGKTTLTAAITMALSV-G-
KSKKYEEIDSSPEEKARGITINTAHVEYETKNRHYAHVDCPGHADYIKNMITGAAQMD
GAILVISATDGPMPQTKEHILLAKQVGVPNLVVFLNKEDQIDDNELLELIELEIRETLN
NYEFPGDEIPIITGSALLAIEADKILDLMDKIDSYIPTPIRDTDKDFLLAIEDVLSITGRG
TVATGRIERGKIKVGETVELIGLIKSTTITGLEMFQKSLAIAGDNVGVLLRGIQKNEVE
RGMVIAKPGTIQPHIKFNSQVYILTKEEGGRHTPFFEGYKPQFYVRTTDVTGKIMVMP
GDKIKMIVELVQPIAIEGMRFAIREGGKTVGAGVIINII
>NP_041900.1_Euglena_gracilis
FERKPHINIGTIGHVDHGKTTLTAAITMALAATGNKAKRYEDIDSAPEEKARGITINTA
HVEYETKNRHYAHVDCPGHADYVKNMITGAAQMDGAILVVSAADGPMPQTKEHIL
LAKQVGVPNIVVFLNKEDQVDDSELLELVELEIRETLSNYEFPGDDIPVIPGSALLSVEA
DKILNLMDQVDSYIPTPTRDTEKDFLMAIEDVLSITGRGTVATGRVERGTIKVGETVE
LVGLTRSTTITGLEMFQKSLALAGDNVGVLLRGIQKNDVERGMVLAKPRTINPHTKF
DSQVYILTKEEGGRHTPFFEGYRPQFYVRTTDVTGKIMVMPGDRIKMKVELIQPIAIE
GMRFAIREGGRTVGAGVVLSII
>NP_038420.1_Mesostigma_viride
FERKPHINIGTIGHVDHGKTTLTAAITMALAVGTGSGKRYDEIDAAPEEKARGITINTA
HVEYETETRHYAHVDCPGHADYVKNMITGAAQMDGAILVVSGADGPMPQTKEHIL
LAKQVGVPNMVVFLNKEDQVDDPELLELVELEVRETLNSYDFPGDEIPVVAGSALMA
LEADKIYALMDQVDKYIPTPQRDTDKPFLMAIEDVFSITGRGTVATGRVERGKVVVG
ENVDIVGLPQNTTVTGLEMFQKTLSVAGDNVGVLLRGIQKDQVERGMVLAKPNTIK
PHIRFESEVYVLAKEEGGRHTPFFPGYRPQFYVRTTDVTGKIMVMPGDRVKMVVSLI

QPIAIEGMRFAIREGGRTVGAGIVSEIL
>NP_003312.3_Homo_sapiens
YVRKPHVNVGTIGHVDHGKTTLTAAITKILAEGGGKFKKYEEIDNAPEERARGITINA
AHVEYSTAARHYAHTDCPGHADYVKNMITGTAPLDGCILVVAANDGPMPQTREHLL
LARQIGVEHVVVYVNKADAVQDSEMVELVELEIRELLTEFGYKGEETPVIVGSALCAL
EGKSVQKLLDAVDTYIPVPARDLEKPFLLPVEAVYSVPGRGTVVTGTLERGILKKGDE
CELLGHNIRTVVTGIEMFHKSLAEAGDNLGALVRGLKREDLRRGLVMVKPGSIKPHQ
KVEAQVYILSKEEGGRHKPFVSHFMPVMFSLTWDMACRILAMPGEDLKFNLILRQPM
ILEGQRFTLRDGNRTIGTGLVTNTL
>KYQ88929.1_Tieghemostelium_lacteum
FERKPHVNVGTIGHVDHGKTTLTAAITKFLSDKGLNFKSYSQIDKSPEERSRGITITAS
HIEYESPLRHYAHIDCPGHQHYIKNMITGAAQMDGAILVVSAPDGPQEQTREHVILSR
EVGIPAIVVFLNKMDNA-
DPELVEIVEMEVRELLSKYGFDGENTPFVKGAAAVALAEQAIEKLVEVLDTKIPTPHRA
IDKPFLMPVEEVFSISGRGTVATGRIEAGVVKVGDEISIVGIIPKVAVTGLEIFGKILAQA
GETVGALLRGLKRDDVVRGEVLSKPGTIKPYTKFQAKTYILTEGEGGRKKGFATNYR
PQFFIRTANVTGRIMAMPGDNLEFDVELISPIPLNGLRFAIREGQLTVGAGIISKVI
>KNE01171.2_Candida_auris
FDRKPHVNIGTIGHVDHGKTTLTAAITKVLSEKGGSFLDYGSIDRAPEERARGITISTA
HVEYQTDKRHYAHVDCPGHADYIKNMITGAAQMDGAIIVVAASDGQMPQTREHLL
LARQVGVQNLVVFVNKVDTIDDPEMLELVEMEMRELLTTYGFDGDETPVIMGSALC
ALEGKAIMKLLDAVDEYIPTPQRDLEQPFLMPVEDVFSISGRGTVVTGRVERGSLKKG
EEIEIVGDTFKATVTGIEMFKKELAMAGDNAGILLRGVKRDEVSRGDVLAKPGTVSPH
TKILASLYVLTKEEGGRHNPFGENYKPQLFLRTTNVTGTMMVNPGDNVEMVIELGK
KCPIENQRFNLREGGKTVATGLVTRII
>KFG49798.1_Toxoplasma_gondii
FKKKPHINIGTIGHVDHGKTTLTAAITYVLAKNNQKLKTYKEIDCAPEEIARGITIKTS
HIEYETAVRHYAHIDCPGHADYIKNMITGAAQMDGAILVVSAVDGPMPQTKEHLLLA
KQIGISNIIVFLNKIDLIDDNEILELVELETRELLDKYNFSSD-
TPIITGSALKALDNDKIYELLTALDSYIPLPKRDLDKPFLLAIEDIFSITGRGTVVTGKIE
RGSIKLGDTVTILGFSKNVVVIGLEMFQKTLGEAGDNVGILLRGIQKTEVKRGMILSK
PLTMTLHSIFQADVYILTVAEGGREKPIFEGYCPQFYLYTINITGSIMILPGDRVKLNVT
LIYSIAIEGMRFAIREGGRTIGAGIITDII
>KAF3308749.1_Arthrobotrys_oligospora
FERKPHVNIGTIGHVDHGKTTLTAAITKVQASKGLNFLDYASIDKAPEEKARGITISTA
HLEYETPSRHYSHVDCPGHADYIKNMITGAASMDGAIIVVAASDGQMPQTREHLLLA

RQVGVQKIVVFVNKIDAVEDPEMLELVEMEMRELLGTYGFDGDETPIIMGSALCALN
GSRIGQLMEAVDTWIPTPERDLDKPFLLSVEDVFSISGRGTVATGRVDRGQLKKGEE
VELVGMPIKTTVTGMETFKKELAQAGDNSGLLLRGVKREDVKRGMVIAKPGTIKAA
NKFMCSMYVLSKEEGGRHTGFGNHYRPQMYIRTADVAVGLMVMPGDNIEMVADLH
VPMAIEGLRFNIREGGRTVATGLVTQIL
>KAF1317564.1_Globisporangium_splendens
FERKPHLNVGTIGHVDHGKTTLTAAITKVLAETGGTFTSYEDIDKAPEERARGITISTA
HVEYETANRHYAHVDCPGHADYVKNMITGAAQMDGGILVVSAGDGPMPQTREHIL
LARQVGVPALVVFMNKVDQVDDEELLELVEMEIRELLDVYDFPADEIPVVRGSALAA
VEDDAVLKLMEEVDTYIPNPTRDFEKPFLMPVEDVFSISGRGTVVSGRVEQGIINTGD
EVELVGISTKTTCTGVEMFKKQLGQAGDNVGLLLRGLKRDDVLRGQVLCKPGTIAP
HTKFEAEVYVLKKEEGGRHTPFFSNYRPQFFFRTADVTGNIMVMPGDNTAIDIELISPI
ALEGMKFSIREGGRTIGAGVVSKVE
>KAF0717467.1_Aphanomyces_stellatus
FERKPHMNVGTIGHVDHGKTTLTAAITKVMSEQGGKFVAFEDIDKAPEERARGITIST
SHVEYETEKRHYAHVDCPGHADYVKNMITGAAQMDGGILVVSATDGPMPQTREHIL
LSRQVGVPALVVFLNKVDMVDDEELLELVEMEIRELLDSYDFPGDDIPVVRGSALQA
VQGASILELMNQVDEYIPEPVRDLDRPFLLPVEDVFSISGRGTVVSGRIEMGVIKPGDE
VEIVGISSKSTCTGVEMFRKSLGEAGDNVGVLLRGLKREEVLRGQVVCKPGSITAHTK
VQAQVYVLKKDEGGRHTPFFNNYRPQFFFRTADITGNVMVMPGDNLTLNIELIHPV
AIDGMKFSIREGGRTIGAGVVAKVV
>KAA8498295.1_1_Porphyridium_purpureum
YERKPHLNVGTIGHVDHGKTTLTAAITSVLAQSGGTAMAYDQIDRAPEEKARGITIN
AATVEYETTNRHYAHTDCPGHADYVKNMITGAAQMDGAILVVSGPDGPMPQTREH
ILLARQVGVPSLVVFINKTDMVDDPELLELVEMEVRDVLNFYKFDGDAVPFVRGSAL
KALNGQNVLTLMKTVDEAIPTPQRALDKPLLFAVETSHLISGRGTVATGSIEQGVVK
VGDEVEIVGFPVKTTVTGVEMFRKSLGEAGDVVGLLLRGLKREDLERGMVICAPGAI
KPINKFKAEIYALTKDEGGRHKPFFTGFCPSFFIRTATVTGKVMVMPGDNFRCQVELL
SKVPLDGQRFAVRESGRTVAAGVVTEVL
>KAA0151437.1_Cafeteria_roenbergensis
FDRKPHANIGTIGHVDHGKTTLTAAITKVLAELGGEARAYDEIDKAPEEKARGITINT
AHVEYETENRHYAHVDCPGHADYVKNMITGAAQMDGAILVVSAPDGPMPQTREHIL
LAKQVGVPAMVVFLNKCDMMDDDELLELVEMEIRELLSFYDFDGDDIPITRGSALAA
IEDNAIVELMRTVDEHIPVPPRALDKDFLMPIEDVFSISGRGTVVTGRIEQGVVTVGD
ELEVSGLSLSTTCTGVEMFRKTLGEAGDNVGVLVRGLKRDEINRGQVIAKPGSVTSH
TKFEAEVYVLKKDEGGRHTPFMVGYKPQFFFRTSDITGGVMVMPGDNTSITVQLGV

PVPMAGLRFAVREGGRTVGAGVVSKIV
>GBG33503.1_Hondaea_fermentalgiana
FVRLPHCNVGTIGHVDHGKTTLTAAITKVLSDMNPTFVDYASIDKAPEERARGITIST
AHVEYETENRHYSHVDCPGHADYVKNMITGAATMDGGILVVSASDGPMPQTREHL
LLSKQVGVPALVVFLNKVDQVDDEELLEIVEMEIRELLDNHDFPGDDTPIVRGSALAA
VEGDAVVELMKNVDSFIPQPTRDLDKPFLLPIEDTFSISGRGTVVTGRIEAGCVNAGE
EIEVVGGKIRTTCTGVEMHRKTLGEAGDNVGILLRGVKREDVSRGQVVTHPGNITQ
NTKFLCQVYVLTQKEGGRHSPCFDGYSPQFFIRTADVTGSVMIMPGDDCTLEVTLQS
PTPMNGLRFAMREGGRTIGAGTISKII
>GAX97089.1_Pythium_insidiosum
FDRKPHVNIGTIGHVDHGKTTLTAAITKVLAEKGGKFTSYEDIDKAPEERARGITISTA
HVEYETENRHYAHVDCPGHADYVKNMITGAAQMDGGILVVSAGDGPMPQTREHIL
LARQVGVPALVVFMNKVDQVDDEELLELVEMEIRELLDVYEFPSDEIPVVRGSALAAV
EDDAVLKLMDEVDSYIPTPTRDFEKPFLMPVEDVFSISGRGTVVSGRVEQGVINVGDE
VELVGISSKSTCTGVEMFKKQLGQAGDNVGLLLRGLKRDEVLRGQVLCKPGTIAPHT
NFEAEVYVLKKEEGGRHTPFFTNYRPQFFFRTADITGSIMVMPGDNTAIDIELISPVAL
EGMKFSIREGGRTIGAGVVSKVA
>GAX11859.1_Fistulifera_solaris
FNRKPHVNIGTIGHVDHGKTTLTQAITKVLSEKGWKSMSYEDIDRAPEEKARKITINT
SHIEYETANRHYGHIDCPGHADYVKNMITGAAQMDGAILVVGATDGPMPQTREHIL
LAKQVGIPNLVVFLNKCDLVDDEELLELVEMEIRELLSFYKFDGDAIEIVRGSALAAAN
GDAILKLMDAVDQHIPEPVRETDKPFLMPIEDTFSIAGRGTVVTGRVEQGIVNVNDE
LEVIGLNKKTVCTGVEMFRKLLGRAGDNIGALLRGLKRDDVRRGQVLCKPGSIKEAK
KFEAEIYALNKEEGGRHKPFMTNYRPQFFFRTADVTGSLMVMPGDNTSITVDLIAPIA
LEGLRFNMREGGMTVGTGIVTKVL
>EWG93154.1_Saccharomyces_cerevisiae
FDRKPHVNIGTIGHVDHGKTTLTAAITKTLAAKG-N-
LDYAAIDKAPEERARGITISTAHVEYETAKRHYSHVDCPGHADYIKNMITGAAQMDG
AIIVVAATDGQMPQTREHLLLARQVGVQHIVVFVNKVDTIDDPEMLELVEMEMREL
LNEYGFDGDNAPIIMGSALCALEGQAIMKLLDAVDEYIPTPERDLNKPFLMPVEDIFSI
SGRGTVVTGRVERGNLKKGEELEIVGHPLKTTVTGIEMFRKELAMAGDNAGVLLRGI
RRDQLKRGMVLAKPGTVKAYTKILASLYILSKEEGGRHSGFGENYRPQMFIRTADVTV
VMQVMPGDNVEMECDLIHPTPLEGQRFNIREGGRTVGTGLITRII
>ETI46377.1_Phytophthora_parasitica
FDRKPHLNVGTIGHVDHGKTTLTAAITKVLSETGGEFTSYADIDKAPEERARGITISTA
HVEYETSKRHYAHVDCPGHADYVKNMITGAAQMDGGILVVSAGDGPMPQTREHILL

ARQVGVPALVVFMNKVDQVDDEELLELVEMEIRELLEAYDFPADDIPVVQGSALAAV
EGDAVLKLMDEVDAYIPDPVRDFEKPFLMPVEDVFSISGRGTVVSGRVEQGVINTGD
EVELVGLSTKTTCTGVEMFKKSLGQAGDNVGLLLRGLKRDEVLRGQVLCKPGTINP
HTKFEAEVYVLKKEEGGRHTPFFSNYRPQFFFRTADVTGNIMVMPGDNTAIDIELIHP
IALDGMKFSIREGGRTIGAGVVSKVD
>EPR62591.1_Toxoplasma_gondii
FQRKPHLNIGTIGHVDHGKTTLTAAITKVLADMGQDFKSYAEIDKSPEEQKRGITINA
THVEYETSKRHYGHVDCPGHADYVKNMITGAAQMDGAILVVSAYDGPMPQTREHIL
LSKQVGVPRLVVYLNKMDMVEDQELVELVEMEVRELLSFYDFPGDDTPFVKGSALK
ALNGKTIQDLMQACDDFIPEPERKADLPLIIPVESVLSIPGKGTVATGRVEQGTAKPN
EAIEIVGGPLKAQIAALEMFRKTLAQAGDQVGCLLKGIKRDEVKRGMVLGAPGYLKT
FKKFEADLYVLKEEEGGRKKPFFSHYRPQAFIRTGDMACTIMAMPGDRVSCTVELLH
PTALHGLRFALREGGRTVASGIVTKPM
>CBN78489_Ectocarpus_siliculosus
FERKPHVNIGTIGHVDHGKTTLTAAITKVLSDKGLKARSYTDIDNAPEEKARKITINTA
HVEYETANRHYAHVDCPGHADYVKNMITGAAQMDGGILVVSVTDGPMPQTREHIL
LAHQVGVPELVVFLNKVDLLEDAELQELVEMEIRELLSFYKFDGDDIPLVAGSALAAV
EGDAIMELMAAVDEHIPTPTRALDKPFLMPVEDVFSIAGRGTVVTGRVEQGVVKVG
DELEISGIVVKTTCTGVEMFKKLLGQAGDNIGALLRGLKREDVQRGQVIHKPNSVSV
HKKFKAEVYVLTKDEGGRHTPFFSNYRPQFFVRTADVTGSVMVMPGDTVSLTIDLIS
PVVMQGLRFALREGGRTVGAGVVSSIV
>CAA61511.1_Arabidopsis_thaliana
FTRKPHVNVGTIGHVDHGKTTLTAAITKVLAEEGKKAIAFDEIDKAPEEKKRGITIATA
HVEYETAKRHYAHVDCPGHADYVKNMITGAAQMDGGILVVSGPDGPMPQTKEHIL
LARQVGVPSLVCFLNKVDVVDDPELLELVEMELRELLSFYKFPGDDIPIIRGSALSALQ
GQAILKLMDAVDEYIPDPVRVLDKPFLMPIEDVFSIQGRGTVATGRIEQGVIKVGEEV
EILGLSTKSTVTGVEMFKKILGQAGDNVGLLLRGLKREDIQRGMVIAKPGSCKTYKKF
EAEIYVLTKDEGGRHTAFFSNYRPQFYLRTADITGKVMVMPGDNVTAVFELIMPVPL
EGQRFALREGGRTVGAGVVSKVM
>BBK20573.1_Cryptomonas_sp.
FERKPHVNIGTIGHVDHGKTTLTAAISATLAIYTGITKKFDEIDSAPEEKARGITINTAH
IEYETEKRHYAHVDCPGHADYVKNMITGAAQMDGAILVCSAADGPMPQTREHILLA
KQVGVPHIVVFLNKADMVDDEELLELVQLEVQELLSKYDFPGDEIPFVSGSALLALEA
DTIYNLMDKVDSYIPTPEREVDKSFLMAVEDVFSITGRGTVATGRVERGQVKVGDTIE
IVGLTRNTIITGLEMFQKSLAIAGDNVGILLRGVQKVDIERGMVLSKPGSITPHTKFEG
EVYVLTKEEGGRHTPFFTGYRPQFYVRTTDVTGTIMVMPGDRIKMTAQLIHPIAIEG

MRFAIREGGRTVGAGVVSKII
>BBH43092.1_Spumella_sp._NIES-1846
YERKPHINIGTIGHVDHGKTTLTAAITATLSLIGGKLRKYEDIDAAPEERTRGITINTA
HVEYETEVRHYAHVDCPGHADYIKNMITGAAQMDGAILVVSASDGAMPQTREHILL
ARQVGVKNLIVFINKSDLIDDQEIFELIEMELRELLQKYEYNSEEIPFIYGSALSAFNADR
IFDLLDTIDKYIPTPERDINKPLLMTIEDVFSITGRGTVATGKIERGIINMGDSIELIGFK
CITTITGIEMFQKSLGIAGDNIGVLLRGLEKKDVIRGMILAKPGSIKPHTKFYAEVYLLT
KDEGGRHTGLNVGYRPQFYIRTTDITGQIIAMPGDTITIAIELIAAIAIEEMRFAIREGG
KTVGSGIITNII
>BBC77850.1_Nitzschia_palea
FERKPHVNIGTIGHVDHGKTTLTAAITATLALESGVVKGYADIDAAPEERARGITINTA
HVEYETANRHYAHVDCPGHADYVKNMITGAAQMDGAILVVSAADGPMPQTREHIL
LSKQVGVPDIVVFLNKQDQVDDDELLELVELEVRELLSAYDFPGDDIPICPGSALQAI
EADKIFALMDAVDEYIPTPERDTEKTFLMAIEDVFSITGRGTVATGRIERGVIKVGDSV
EIVGITKTTTITGIEMFQKTLGFAGDNVGILLRGVTRENIERGMVLAKPGTITPHTNFE
SEVYVLTSEEGGRRTPFFTGYRPQFYVRTTDVTGAIMVMPGDRIKMTSELIYPVAIEG
MRFAIREGGRTIGAGVVSKIV
>BBC77629.1_Nitzschia_sp._NIES3576
FERKPHVNIGTIGHVDHGKTTLTAAITAVLSLEGNKIKNYADIDGAPEERERGITINTA
HIEYETSHRHYAHVDCPGHADYIKNMITGAAQMDGAILVVSAADGPMPQTREHILLS
KQVGIPKIIVFLNKEDQVDDSELLELVELEVRELLSNYDFPGEETPICIGSALKAIESDKI
FNLMDIVDNYIPTPQRDTEKSFLMAIEDIFSIIGRGTVATGRIERGIIKIGDSIEIVGITKL
TTIIGIEMFQKTLGVAGDNVGILLRGINRENIERGMVLASPNSITPHTTFESEVYILTQE
EGGRHTPFFKGYRPQFYIRTTDVTGVIMVMPGDRIKMTVEFIHPIAIETMRFAIREGG
RTIGAGVVSKII
>BBC77523.1_Nitzschia_sp._PL14
FERKPHVNIGTIGHVDHGKTTLTAAITTVLSLEGNKIKSYADIDGAPEERERGITINTA
HIEYETLNRHYAHVDCPGHADYIKNMITGAAQMDGAILVVSAADGPMPQTREHILLS
KQVGIPKILVFLNKEDQVDDEELLELVELEVRELLSSYDFPGDDTPICIGSALKAIEADK
IFNLMDIVDTYIPTPERDVEKPFLMAIEDIFSIIGRGTVATGRIERGVIKIGDPIEIVGITK
STTIIGIEMFQKTLGVAGDNVGILLRGVNRENIERGMVLANPNTITPHTKFESEVYILT
HEEGGRHTPFFKGYRPQFYVRTTDVTGTIMVMPGDRIKMTVEFIYPIAIESMRFAIRE
GGKTIGAGVVSKIL
>BBC77417.1_Nitzschia_sp._PL32
FERKPHVNIGTIGHVDHGKTTLTAAITAALSLEGNKIKNYADIDGAPEERERGITINTA
HIEYETHLRHYAHVDCPGHADYIKNMITGAAQMDGAILVVSAADGPMPQTREHILLS

KQVGIPKIIVFLNKEDQVDDVELLELVELEVRDLLSSYDFPGEETPICVGSALKAIESDK
IFNLMDTVDEYIPTPKRDVDKTFLMAIEDIFSIMGRGTVATGRIERGIIKVGDSVEIVGI
TKLTTIIGIEMFQKTLGVAGDNVGILLRGINRENIERGMVLANPGTITPHTIFESEVYIL
TKEEGGRHTPFFKGYRPQFYVRTTDVTGVIMVMPGDRIKMTVELIHPIAIETMRFAIR
EGGKTIGAGVVSKII
>AYO28705.1_Neotessella_volvocina
FDRKPHVNIGTIGHVDHGKTTLTAAITAVLST-G-
KAKDYGDIDAAPEERARGITINTAHVEYETNSRHYAHVDCPGHADYVKNMITGAAQ
MDGAILVVSAVDGAMPQTREHILLARQVGVQKLVVFLNKADQVDDQELLELVELEL
RELLDSYEYPGQEIPFISGSALLALEAAKVFDLMDAVDTYIPTPTRETELPFLLAIEDVF
SITGRGTVATGRIERGEVVIGDTVELIGLTKTVTVTGLEMFQKTLGMAGDNIGVLLR
GVQKNEVLRGMVLAKPGSIKAHTVFEAEVYILTKEEGGRHTAIFEGYRPQFFVRTTDV
TGQIMVMPGDNINMRIELITSIAIEGMRLAIREGGRTVGSGLVTTII
>AYO28121.1_Synura_petersenii
FERKPHINIGTIGHVDHGKTTLTAAITATLSTLGNVLKRYEDIDAAPEERARGITINTA
HVEYETEFRHYAHVDCPGHADYVKNMITGAAQMDGAILVVSAADGAMPQTREHILL
ARQVGVQKLVVFLNKADQVDDAELLELVELELRELLQSYDYPGDEIPFISGSALLALE
ADKIFQLMDSVDEYIPTPERETDKPFLMAIEDVFSITGRGTVSTGKIERGSVLLGDTVE
LVGLTKTTTVTGLEMFQKTLGLAGDNIGILLRGVQKNEVLRGMVLSKPGTINPHTKF
EAEVYVLTKDEGGRHTSIFEGYRPQFYVRTTDVTGQIMVMPGDNVTMLIELIAPIAIE
GMRLAIREGGRTVGSGLVTKII
>AYC64615.1_Halimeda_minima
FERKPHLNIGTIGHVDHGKTTLTAAITMALAATGSKAKNYADIDSAPEEKARGITINT
AHVEYETEKRHYAHVDCPGHADYVKNMITGAAQMDGAILVVSGADGPMPQTKEHI
LLAQQVGVPAIVVFLNKIDQVDDDDLLELVELEIRDTLNKYDFPGDDIPIISGSALAAV
EADKIYKLMDVIDEEIPLPLRNTDKDFLMAIENVVSITGRGTVATGRVERGQIKVGQTI
EIVGLTKETTVIGLEMFQKTLSVAGDNVGVLLRGVQKNEIQRGMVLAKPGSITPHTR
FKAQVYVLKKDEGGRHTSFVAGYRPQFYVRTTDVTGKIMVMPGDRVKIIVELIQPIAI
EGMRFAIREGGRTVGAGIVSEIL
>AWX53376.1_Halochlorococcum_sp.
FERKPHVNIGTIGHVDHGKTTLTAAITMALQKNSDVGKKYDEIDSAPEEKARGITINT
AHVEYETENRHYAHVDCPGHADYVKNMITGAAQMDGAILVVSGADGPMPQTKEHL
LLAKQVGVPHIVVFLNKEDQVDDAELLELVDLEVRETLDAYEFPGDEIPIISGSALLAL
EANKIYELMEQVDSYIPTPQRDTDKTFLMAVEDVFSITGRGTVATGRVERGVLKTGE
NVEIVGLTQTVTVTGLEMFQKTLTVAGDNVGVLLRGVQKEDIQRGMVLAQPKTIMP
HTKFEAQVYVLTKEEGGRHTPFFPGYRPQFYVRTTDVTGKIMVIPGDRVKMVVELIQ

PIAIEGMRFAIREGGRTVGAGVVSKIL
>ASQ40356.1_Cyanoptyche_gloeocystis
FERKPHVNIGTIGHVDHGKTTLTAAITTALAANGRKARRYDEIDAAPEEKARGITINT
AHVEYETENRHYAHVDCPGHADYVKNMITGAAQMDGAILVVSAADGPMPQTREHI
LLAKQVGVPNMVVFLNKEDQIDDQDIMELVELEVRELLSQYDFPGDAIPFVSGSALLA
LENDKILSLMDAVDTYIPTPQRAVDKPFLMAIEDVFSITGRGTVATGRIERGKVKVGE
VVELVGLTRSTTVTGLEMFQKTLGMAGDNIGILLRGIQKDDIARGMVLAKPGSITPH
TKFEAKVYILRKEEGGRHKPFFCGYRPQFYVRTTDVTGTIMVMPGDRIKMTVSLVHPI
AIEGMRFAIREGGRTVGAGVVFSIV
>AMK97191.1_Sargassum_vachellianum
FDRKPHINIGTIGHVDHGKTTLTAAITAVLSLAG-
NAKKYEDIDAAPEERARGITINTAHVEYETETRHYAHVDCPGHADYVKNMITGAAQ
MDGAILVVSAADGPMPQTREHLLLSKQVGVPHIVVFLNKEDQVDDLELIELVELEVR
ELLSNYEFPGDDIPIVAGSALQALEADKIYNLMEEVDNYIPTPIRDTEKTFLMAIEDVFS
ITGRGTVATGKIDRGIIKVGETVELVGLTKSTTVTGVEMFQKTLGVAGDNVGILLRGL
QKTEIERGMVLSKPGTITPHNTFESEVYVLTKEEGGRHTPFFVGYRPQFYVRTTDVT
GEIMVMPGDRVKMTAGLISLIAIEGMRFAIREGGRTIGAGVVSKIL
>ALP86070.1_Phacus_orbicularis
FERKPHINIGTIGHVDHGKTTLTAAITMALAATGNKAKKYEDIDSAPEEKARGITINTA
HVEYETSNRHYAHVDCPGHADYVKNMITGAAQMDGAILVVSGADGPMPQTKEHIL
LAKQVGVPNIVVFLNKEDQVEDKELLDLVELEIRETLSNYEFPGDEIPIVSGSALLSVEA
DKILDLMDQVDAYIPTPKRDTDKDFLMAIEDVFSITGRGTVATGRVERGTIKVGESVE
LVGLTKSTTVTGLEMFQKSLALAGDNVGVLLRGIQKNDVERGMVISKPGTINPHVKF
DSQVYILTKEEGGRHTPFFEGYRPQFYVRTTDVTGKIMVMPGDRIKMEVELIQPIAIE
GMRFAIREGGRTVGAGVVLSII
>ALG63619.1_Guillardia_theta
FERKPHVNIGTIGHVDHGKTTLTAAISAVLSTLSGSSKKFDEIDSAPEERARGITINTAH
VEYETPKRHYAHVDCPGHADYVKNMITGAAQMDGAILVCSAADGPMPQTREHILLA
KQVGVPHIVVFLNKADMVDDEELLELVQLEVQELLSKYDFPGDDIPFVSGSALLALET
DTIFELMEKIDEYIPTPVREVDKSFLMAVEDVFSITGRGTVATGRIERGAVKVGDTIEII
GLTRTTTITGLEMFQKSLAMAGDNVGILVRGIQKTDIERGMVLAAPGSITPHTKFEG
EVYVLTKEEGGRHTPFFTGYRPQFYVRTTDVTGTIMVMPGDRIKMTAQLIHPIAIEG
MRFAIREGGRTVGAGVVSKIL
>AKT93936.1_Vermamoeba_vermiformis
FVRKPHCNIGTIGHVDHGKTTLTAAITKILAKNKGKFMDYSDIDRHKEERERGITIVA
THVEYETDKRHYSHIDCPGHQHYIKNMITGATQMEGAILVVSVTDGPQVQTREHVIL

AKEIGIPAMVVFVNKMDALKDKDMVELVELETRELLNTYSYPYD-
LPIIFGAARVALEEESILKLMSTVDSYIPQPVRPVNDPFLMPIEDVFSITGRGTVVTGKV
ERGTIKVGEEIELVGPVLKSTCTGLEMYHKFLAQAGENVGALIRGVASDAVKRGYVLA
KPGSLIPVTTFEAKAYILTKKEGGRSKPFISNYKPQFFFRTANVTGAVIVMPGDTVNFK
VELIEKAPISGLRFTLREGSLTIGAGVITKIL
>AIU44600.1_Cyanophora_paradoxa
FERKPHVNIGTIGHVDHGKTTLTAAITTALASQGKKARKYDEIDAAPEEKARGITINT
AHVEYETEKRHYAHVDCPGHADYVKNMITGAAQMDGAILVVSAADGPMPQTREHIL
LAKQVGVPNMVVFLNKEDQIDDADLLELVELEVRELLSKYDFPGDQIPFVSGSALLAL
ESDKILALMDAVDEYIPTPERPIDKSFLMAIEDVFSITGRGTVATGRIERGAIKVGETVE
LVGLTKSTTVTGLEMFQKTLGMAGDNIGILLRGVQKTEIERGMVLAKPGSITPHTQF
ESEVYVLTKDEGGRHTPFFSGYRPQFYVRTTDVTGSIMVMPGDRIKMTVSLVHPIAIE
GMRFAIREGGRTIGAGVVSKIL
>AEW13004.1_Strombomonas_acuminata
FERKPHINIGTIGHVDHGKTTLTAAITMALAASGNKAKRYEDIDSAPEEKARGITINTA
HVEYETKNRHYAHVDCPGHADYVKNMITGAAQMDGAILVVSGADGPMPQTKEHIL
LAKQVGVPNIVVFLNKEDQVEDKELLELVELEVRETLSNYEFPGDEIPVVSGSALLSVE
ADKILELMDQVDAYIPTPTRDTDKDFLMAVEDVFSITGRGTVATGRVERGTVKVGET
VELVGLTRTTTVTGLEMFQKSLALAGDNVGILLRGIQKADIERGMVISKPGTINPHTK
FDSQVYILTKEEGGRHTPFFEGYRPQFYVRTTDVTGKIMVMPGDRIKMEVELIQPIAIE
GMRFAIREGGRTVGAGIVLEII
>AEJ72971.1_Karlodinium_veneficum
FKRKPHINIGTIGHVDHGKTTLTAAISGALTMYSSYRKKFDEIDSAPEEKARGITINTA
HIEYETVKRHYAHVDCPGHADYVKNMITGAAQMDAGILVVSASDGAMPQTREHILL
AYQVGVPYLVIFLNKVDRVEDEELIQMVGEEVLDLLKGYNFPIKNLPILSGSALLAVKA
DNIYKLMKTVDNYIPTPYRDIQKPFLLAIEDVFSITGRGTVATGRIERGQVHVGDIIEII
GLKYTTTVTGLEMFQKTLGIAGDNIGVLLRGIQKSEIDRGMVLAFPGSITPHSKFIAEA
YILTEEEGGRHTPFFTGYRPQFYLRTTDVTGIIMVLPGDRIRLTVELINPVAVEGMRFA
IREGGRTVGAGVVSHVL
>AEI29582.1_Emiliania_huxleyi
FERKPHVNIGTIGHVDHGKTTLTAAISATLAVYSGSKKDISLIDSAPEEKARGITINTAH
VEYETETRHYAHVDCPGHADYVKNMITGAAQMDGGILVVSAADGPMPQTREHILLA
KQVGVPHLVVFLNKADQVDDEELLELVELEVQELLENYDFPGDDIPFVSGSALLALQ
ADRIFDLMESVDNYIPAPERDTEKTFLMAVEDVFSITGRGTVATGRIERGILKIGDSIEI
VGLTQTTTVTGIEMFQKTLGMAGDNVGILIRGVQKTDIERGMVLAQPGTISPHKKFE
AEVYVLGKDEGGRHTPFFTGYRPQFYVRTTDVTGTIMVMPGDRIKMTAELINPIAIE

GMRFAIREGGRTVGAGVVSKIL
>ADB20408.1_Dinophysis_acuminata

MITGAAQMDGAILVCSAADGPMPQTREHILLAKQVGVPYIIVFLNKADMVDDEELLE
LVQLEVQELLDKYDFPGGEIPFVSGSALLALEAKTIFSLMEKVDEYIPDPEREVDKPFL
MAVEDVFSITGRGTVATGRIERGKATVGDTIEIVGLTRNTTITGLEMFQKSLALAGDN
VGVLVRGIQKTDIERGMVMAAPGAITPHTKFEGEVYVLTKEEGGRHTPFFTGYRPQF
YVRTTDVTGTIMVMPGDR------------------------------------
>ABJ91304.1_Pyropia_yezoensis
FERKPHVNIGTIGHVDHGKTTLTAAISATLSTLGSAAKKFDEIDAAPEEKARGITINTA
HVEYETDNRHYAHVDCPGHADYVKNMITGAAQMDGAILVVSAADGPMPQTREHIL
LAKQVGVPTLVVFLNKEDQVDDEELLELVELEGRELLSQYDFPGDDIPFVAGSALLAL
EADKIFSLMEAVDTYIPTPERDVDKTFLMAVEDVFSITGRGTVATGRIERGIIKVGDTI
EIVGLTRTTTITGLEMFQKTLGLAGDNIGILLRGVQKKDIERGMVLAKPGTITPHTQF
EAEVYILTKEEGGRHTPFFPGYRPQFYVRTTDVTGTIMVMPGDRIKMTAELINAIAIE
GMRFAIREGGRTVGAGVVSKIL
>AAZ94901.1_Moneuplotes_crassus
FDRLEHLNVGTIGHIDHGKTTLTAAITKYLSSTGGSFHDYSEIDKAPEERSRGITINSTT
IEYSSESRHYGHVDCPGHADYVKNMITGAARMDGGILVVSATDGAMPQTREHILLCR
QVGVKNIIIFLNKCDQMDDEEMHELVEMEVRELLEDYEYS-
DDVPLIKGSALLALEGSAIQELISTMDSYFEAPTRPIDKDFFMSVESSFNIPGRGTVATG
TIDQGICKIGDDVHLIGIPVATTIVGVESFKKTLGEAGDNVGVLLRGLNREDVLRGAA
LVKPGKFTVNRNFNAEIYVLNTDEGGRNKPFFSGYRPQCFIRTADMACAVMAMPGD
NLSVALKLDRPLAIEGNRFALREGGRTVASGVITEVV
>AAO53239.1_Monomorphina_ovata

IGHVDHGKTTLTAAITMALAVTGNKAKRYEDIDSSPEEKARGITINTAHVEYETKNRH
YAHVDCPGHADYVKNMITGAAQMDGAILVVSGADGPMPQTKEHILLAKQVGVPNIV
VFLNKEDQVDDKELLELVELEVRETLNNYEFPGDEIPVVAGSALLSVEADKILDLMDK
VDEYIPTPIRDTEKDFLMAVEDVFSITGRGTVATVRVERGTVKVGETVELVGLTRTTT
VTGLEMFQKSLALAGDNVGVLLRGIQKTDIERGMVISKPGTINPHTTFDSQVYILTKE
EGGRHTPFFEGYRPQFYVRTTDVTGKIMVMPGDRIKMQVELIQPIAIEGLRFAIREGG

>AAN62452.1_Pylaiella_littoralis

GKTTLTAAITAVLSLSGLNAKKYEDIDAAPEERARGITINTAHVEYETETRHYAHVDC

PGHADYVKNMITGAAQMDGAILVVSAADGPMPQTREHILLSKQVGVPHIVVFLNKE
DQVDDLELVELVELEVRELLSNYDFPGDDIPIITGSALQALDADKIYSLMDSVDSYIPT
PVRDVDKAFLMAIEDVFSITGRGTVATGKIDRGIVKVGETVDLVGLTKSTTVTGVEM
FQKTLGVAGDNVGILLRGLQKGEIERGMVLAKPGTITPHNTFESELYILTKEEGGRHT
PFFPGYRPQFYVRTTDV--
>AAN31832.1_Arabidopsis_thaliana
FERKPHVNIGTIGHVDHGKTTLTAALTMALASIGSVAKKYDEIDAAPEERARGITINTA
TVEYETENRHYAHVDCPGHADYVKNMITGAAQMDGAILVVSGADGPMPQTKEHIL
LAKQVGVPDMVVFLNKEDQVDDAELLELVELEVRELLSSYEFNGDDIPIISGSALLAVE
TDKIYELMDAVDDYIPIPQRQTELPFLLAVEDVFSITGRGTVATGRVERGTVKVGETV
DLVGLTRSYTVTGVEMFQKILALAGDNVGLLLRGIQKADIQRGMVLAKPGSITPHTK
FEAIIYVLKKEEGGRHSPFFAGYRPQFYMRTTDVTGKVMVMPGDRVKIVVELIVPVAC
EGMRFAIREGGKTVGAGVIGTIL
>AAA87696.1_Ochromonas_danica

KNMITGAAQMDGAILVVSAADGAMPQTREHILLARQVGVKKLVVFLNKADQVDDPE
IISLVELELRDLLQSYDYPGDEIPFVAGSALLALEADKIFELMDAVDNYIPTPEREVDKP
FLMAIEDVFSITGRGTVATGRIERGTILLGDTVELVGLTKTTTVTGLEMFQKTLGMA
GDNIGILLRGIQKTDVLRGMVLSKPGSIKPHTKFEAEVYILTK---------------------------
----

Plastid phylogenomics dataset

>Pteridomonas_sp._YPF1301

MIISQIPTIKKICINRCLGNLAQNKVILQKSINEFCLITGQYPRSTYAKKSISGFKLREKTL
IGLTTTLRKNRMYSFLIRLIHFALPQLRDFKGFSLKQFDTFGNYHFGIKNQVIFPELNS
EDIPYLFGFNISIITSITNLNENISLLKSLDFPFMIRIKRGNIANVRRKKILKLAKGFFAAHS
RLFRIAHSKILKAFKNSFIGRKQKKRFFRRLWIIRLNAALKAVNYSFLFFYLKKQNIFLNR
KILSQLTIIDPRTFTKLLM--KIFR------
KNDRIASFATIKNSKPLKSLTYYKHHSNGRNNQGKITIRHHGGSHKKLYRIIDFKRDKI
KMIGIVKKITYDPNRNAKIALIFYSDGEKRYILYPQNLLLYSKITSGISTTLVIGNTLQLQ
DIPIGFYIHNIELYNNIGGQLVRAAGTSAKLLLKKKKYGIIRLPSKEIRLISITAKATIGVIA
SIP--
KQKNNKAGRMRWFGIRPTVRGVAMNACDHPHGGGEGRSPIGRKQPLTLWGKLAN
GPKTRKQHLRYIIKRMSRLGKIPLIIPSNIKIILTRRTCKIQGPYGMLIQNIPKQLNLSNK
LFITKNFSKQQGLIRSLLKNIIKGVIFPFEITLQIIGVGYRAMIQQQHLILTLGYSHKVVL
LIPTDLSLKIVNNIYLIIQGINKKLVSFFGSKIRAFKPPEPYKGTGIKYLNEIIIRKIGKSTN
MTFLSSHNFKKWYLINAENQILGRFASQIIKILVGKHKLTYSPFLPSNTRLIIINAKKICIK
LYKKKYFHYSGYPGKTFFELQSFQSEKLIRTTILGMLPKTGRKMIKQLYIYSGNKHYHS
AQFPKALMIKKKIAYLKLELKAGNATPLPPIGPALGQYGIDIASFCKEYNNKTKNLDQT
IIPIVITIFIDKSYSFILKTPPTTKLLLQAINVSKGSIEPKKKIIGSISNIELEKIAKIKLPDFNT
TNIKKIIKSILGTAKNMGINLMLKPKKIKYNKQHRGRLKGQYHKKSLLKFGSYGLQAK
ESCWLTAKQIEALRRVITRVIKREGTLWFRIFPHKPISTRVPESRMGAGKGAITHWVAT
IKDGIILCEISDISPELAKKVLTLANHKLPIKTKIIRNMINIQTRLTVIDNTGVKKILCIRIL
GSTKSIGIIGDLIIGTIKEAIPQSKLKKSEIIRGLIVCSKQLFRRFNGTHICFEDNAVVLVT
KDNTPIGTRIFGSIAQ-----------------
MYAIIILDGRQLCLMEGNFVIISHIMKKTLLLFNKILLVKSLYIGKPYLTLILGEIIEHVSR
SKLIIFKMRSKKKYRHHLGYRMKITKITIMSYAIGKYIRISPKKVRKVLLQLNRKSYLYAL
MCLEFMPYKACEIIWKLIYSASANALYKFNTKKELLVISSVQVQKGPILKRFRIRAKGNI
VAIQKVTSHIKITIM-
KKLIHPTWFLKANVYCNGIFIKQVSSTKSILSVDIWSGTHPFFIKKK-
TKYLKNQTDRFLKKYKIMAHKKGASSTKNGRDSISKRLGIKIYGGAIIKAGNIILRQRGL
KYKAGKNIICGKDFTLVAKKSGYVLYEVYLKMKVKSSIKKICKLCRILKRKNILRIICKNP
KHNQRQSMAVPKKRTSLIKKRHRHSIWVKHAQFFFNKAFLLSSSVNKNFYNVYMIKQI
KSTTETSHFIISELFLGNGLTIGNSLRRMLLLNLKGTALIAIKISNICHEFSSIYGIREDILE
ILLNLKEITLKTQYGLLTADGPGIITGNCLSFNSNIKIINPNQYIATLYLSTKITFEVIAMS
NKGYRLNFLNIDAIFMPILKVNYKIITKTESLLLELTTNGSIAPYKAFHEASFLLKRLFSS-
----

QNRTFKKTSVKNFIYSAYTNYNKTQNLHLLNELKNLGFFYATKSGLSLGISDFKIPVIK
KQLIIKSYSISFNANIAFLNGTITFVERFQQIFDIWSKTNFILINSIINYLTKVDPFNSLRL
MVFSGARGNIDQLRQLIGIRGLMADSNGQLIDIPIIHNFKEGLTLTDYLIASFGARKGV
VDTALKTAEAGYLTRRLITTTTDMIIRDNDCFTTFFLRIIIGRISAETIYIIYINTQITENIA
LLLFMKPYICVYSILTCLLDHSICKKCYGWDISRQKGIKLGESIGVIAAQSIGEPAIQLTM
RTFHTGGSFTNRSRQIRINNNGLIFYKQIKNSVTVRTNSGILAQFVKTKLNIQLIFTINF
LLPPNIYLYIKSTSFISKNSLLAEIIDLKKELIAPHSGEIFLAITLCILKGKVYDLPRNFSLIS
NFNYKFIFYSKICVLINGFLKVFLLDLGLFIPCEFFSTSIPSFFINTYLLEIFSGYFSKNRGF
FQKFFTRGTIKPCNLRFSGEIIGEHQIISFLCLIELLRPIYDVNILNFIHSNLLILKILPQIK
NLEYVRMNSIIGLSSFFINEPSIKKIRIVKPRILYIKNNHLYRYFFEQGKINYFKSSKMGKIL
SNTSFSTLLHQGTPFFITSNIRLYYYPKNLIKKDDIFGIIMFDQTISGDIIKGLPKIEEILEI
QTISKIKNNKIQVGQTLTVNIKDLLNIIFVYFSSVYSTFKRLQILLTKQILEIYLIQKIYIVS
KHIEILLFQLTSKIKIICDDSNTFIIGEIVDLSNIFTIMQIYHTNYYKPYIVGISSFSKYERSF
ISAASFQNTTMILSRAAIEGRLDWLHGLKENVITGRCISFGTRMFVTTLPSFIAAQCSSF
CWFLSQGILETFHILPFNILEQLFDLNFYCEHYFFTHSKNTILDAKTYNTTFALKVYIPI
TFVFRKILEHELLFLGEIPLMTNKGTFIINGYEKIIIHYFIKKQGLIYIYSALLIPKTGVWV
KLELNELTICINKEKKLNIFDFFKLFYLSDLCLLFPCFKLLNFRLFNQLFNVCFYELNVN
IRDEFKKTF-
FHSSNQSTTLTLQDIFSIINHLFFLRSHNDEPDHLTNKVIIPVGKILQKQFLFSLHHLKR
QLTSRLLNHLISIFNIFFSSSQLVQFFDQTNLLAELAHRNRLNTLSIGTM----
ISLKARDIHTSQFGRICPLETPEGFKTGIILALSIYSQINNSGSIEIPFFIVKNGYILTQFPAI
YLTAKEEECKKIAMADIRIGKIIQKYVTIRLYQKFYVVLSNEVELIAVSTQQLFSFGASLI
PFIEHNDGNRALMGANMQRQSTPLIYPHKPIIGTGLEMQFASD--
LAIKSFSSGIIFDIFHNFVIILYFIQKYIRTNQDTFNNQRFIIWKGEFVKSGQILADEATME
QGEIALGQNLLVAYMSWNGYNFEDSILINEHLLYSDLLTSIKITQFTLFVKYTINGIETK
NIPEVTKNLDLNGIIKKGIFVQPNDLIIGKRTPFTEKDIQNYSFLLTVFEGYNTSIYATV
LDVLICSNIFFAQIRKITVGDKIAGRHGNKGIISKILANQDMPFLPNGKIIDLILNPLGVP
SRMNVGQLFEGLLGFAGTILERRFKILPFDERFGDHASYLLIFKYLQKAKSKNLWIFNK
TIPGKIIVRDGKTGNCFDNSIVIGTSYFIKLIHFSEDKLHFRSTGPYSLITQQPLHGKSA
DGGQRFGEMEVWALEAFGAAYTLQEMLNVKSDDVKNRNKLLNSITFSGIPESFKLLIL
ELKALGLDLLAFKSNNK---KNFVFMFDFLSIQLASPTQIKKWGTYRRT-
NGEFGQILTDEILAPGTLQPSPDGLFCEKIFGPTTQDKCFCGKEIFCETCNVEFSSSQT
RRYRMGYILLKIPVTHIWYLYGMPSYLSLLLLLSTEGANLLKYLLNNLVLESEIFYCKLL
LHLYKEPRLKLIKRIRLLENFIATEANPSWLILDILPVLPPMLRPLRVDEQGTVILSDLT
HLYLNILKANNNLIKKLNSQEPLIFINNAQRFLQEAVDNLIDNERRNITIKTDNNSNLA
SLSSLLGSKYGRFRENLLGKRVDYSGRSVIVVAPFLKLTQCGLPYRLAIELFKPFLLLLR

LNLQIFLTESFIIKLLIIIRTRFILLNRAPTLHRLGIQAFTPILIEGYAIGLHPLICSSLNADF
DGDQVAIHLPLTLKAQIEAKTLIAIPNNILSITTGEPIIIPTQDMIIGYNYLTLVSF-
NRYGTIYYFNDYQDILNAYEQQVITLQSPIWVRHTFNSYFLTTFGRLLLNKAFFKILMS
RYRGPKNRIINKLGLPGLKSKVTKKKNNKKTEYCTRLNEKQKLRFNYGVSEKQLYNYV
K----KRTIL-
TTFITALEMRLDSIVYNFGFAKSILSARQLVNHGKIRVNLIKINIPSFQCKKGDIIALNFIK
QQQ-----NITPLQNFIKITKFVGKIKENINK--
TPFSINEQLIIAFYAKLGQKVNPSGFRLIIDKNKNSLWFEKISNYSLALKEDSLIRTFFHK
RIYKINLKRNKFIYITISTIIPKSIFYELKKILTSIKLTFIKTTYPKLKPKLFLNFIEKQIKNRI
AFYRITQQLLKQLNKGIKIQIAGRLNGIEKARTQWYLIGQLPLHTLKANIKYFKKSIYTK
FGVIGIKIWILKMINDSISNFLTCLRNGLLAKRQIIKIPFTNLVYKILKILKMENFIKFFKV
L-
LNLIFILIEYKGPKKISPIKKLLRISKLSLRQFITLKTLQVLANGKSICIISTSKGIMLDRHA
KELNLGGELICLISMWKHKIIEIRRVTKVGKGHKRFKFRVIVIIGNGAGYIGLGIGKHTEI
SVAKLKAINNASKHLITFFITKNYTILHFLTAQFRTCKIILKPALPGVGLIASKTIRIICEL
AGIHNISAKQLGSNNILNNAVTTFKALQGFMPRSTLLRKTKIILDSEYDSFLLYLFTQQ
LLKNGKNLLAKRIIYKTCLYIEEQTKKDALKILEKSIIKAKPIIEVKIT---
SKKSKLPKEIPSFRGILLAIRYIIQIIDKHQSFSFSIKLANAIILTSCGLGNAIKHREESLALF
KATKMLTFYERMIRLQLKSFNYKKLLITVLRILSIIQNKKNIAKGPVSLPTKKRIYCLLRS
PHINKKSREHFEIRSYTKILEIYIQPQALTFF--
LPSSVNLKFMPTLNQLIRINRKLKYKKKKVPALAGCPQRRGICLRVYVVTPKKPNSALR
KVARVRLTTGFEVTAYIPGVGHNLQEHSLVLLRGGRAKDLPGVRYHIIRGVLDTIGVK
NRKQGRSKYGTKKYIMIKQRFIKKFGLIYMNLTLHNCIISITDLQGNVFLWSSTGRIGF
KGARKRTTFANQVTIKTIINEAINIGLCFVKFFIKQTGGSLEPLMKILKASKLEILSIWDI
TAISHNGCRSPKKRKIMIRFIGINLSKYKKIQYALIKIYGIGIRRATEILNTLKISELRLMEL
SKNLIIQIRHYIENYLVENLLKRKISLHITRLKEIQCFRGKRHLLQLPVRGQRTRTNART
KKGKKKTKNMLLKNKLGYVISTNMKKTIVVLLIESCKHVIYKKRIKRKRYVLVHDENN
SCFIGDFIYIKKIKPLSKLKYYCLCMAKKSIIQRSIKKFLIKQKYFYKRKYLKTTKKSCLNL
LSSFKIQKSFQKFPKNSSQTRFKRFCWKTGRNRGFFRDFNLSRHSFREMSHNGVLPGL
TKSSWMIHVNFKSPFIYYKLLKKQNFIKNISIKTWSRNSTILPCMLNTLFILYNGKKHFPI
LISEYFIGHKLGEFVLTKNFYSHKKDKKITLMLKLLTKSVKKIQRTTIKNKKYKSRIKTF
TKKCLLYIKKYIFKNLNLAFSALDKCLKLNILHKRNIAKKKSKLQLCVNQMTQLFESTG
KIAIVTVLKIGPCFISQIKQNKFLNLTTIQLGYNSVNKITKANVRHFDTKNIILLQFLREY
TLPFKNLFLGKSYTTILLKKNDLICIRSYSIGKGFQGTRKRYNFNKGPMTHGSKNHKK
PGSIGQGTTPGRVFVGKKLAGHLGIKKNTLTNIQIVKINYKENLLFVKGTIGGKYGNL
VTIFM-LNKKLRKIITLECLACKNNQKRH--

ISRYTTFKNKQNTPKKLELKKFCAFCKKHMNYRELKMTQLFESTGKIAIVTVLKIGPCF
ISQIKQNKFLNLTTIQLGYNSVNKITKANVRHFDTKNIILLQFLREYTLPFKNLFLGKSY
TTILLKKNDLICIRSYSIGKGFQGTRKRYNFNKGPMTHGSKNHKKPGSIGQGTTPGRV
FVGKKLAGHLGIKKNTLTNIQIVKINYKENLLFVKGTIGGKYGNLVTIFM--FFIYSIL----
-
LKPNILKKKNYLIHKIIHSTKINYYLKLNTTKTRANVRGGGKKPLKQKGTGHARIGSLR
SPLRKGGGVIWGPKHKIFPKKRNIKELKLTFQTILYNYRFNLFILDKLISYQTKNIFS--
QNFKHCTLLIYLIYNNTLNYCIKNIKNCIYLPINKINLNLLLISKKLLFEENTLFFFKSIYM
LKIRLKRYGQKHNPIYNVGIMLSQTKRNGLTIKTVGFYNPQTKEFKLNVNIIVQFLYT
GVQPTSKLIYLLKKILMIKKISILTDKATILLTINKYSFIIDKLFNKLILKKILEQAFNINIQ
KINTMRLIKKKTYNTLYKKIIITIKKNENINLFSM-
TVKYNLQNIDIGDYITLGIDTLEKHQIRIYEYSGFIIGQKNKGVNKTLILRNICKGISIDR
YVLIYSPEILFIKINNSVLLDRSKGYTLYT-----------F
>Attheya_longicornis_MG755798.1
MLKTEYPKLKKIQINRGLGLAAQNSNILKKSIQEFTAITGQKPVITRSKKAIAGFKIRED
MELGLSVTLRGEKMYSFITKLIFFTFAQIRDFRGLSVRSFDKAGNYTLGLKEQLIFPEIE
YEDVDQTQGFSITIVMSNSSPKNGIILFEFLRFPLMVRVKRGNVARKRRKKVLQLAKG
YRGAHSRLFRVANQQVMKALRYSYIGRKQKKRVFRKIWITRINAASRLLSYSRLIHNFK
KSNIELNRKMLSQVAVLDTLTFKELIMSIRLYKSYTPGTRNRALSAFTEITKSTPEKKLI
RKNHRNKGRNNRGVITIRHRGGGHKRRYRLIDFRRNKYNMEGLVASVEYDPNRNARI
ALIHYTDGEKRYILHPKNLNVGDKIHSGSGSALQIGNTLPIEEIPLGTSIHNIELIPNRG
GQIVRAAGTSAKILAKEGNYVTLRLPSKEIRLIRKECFATIGEIGNNDTFLVQSGKAGRT
RWLGKRPTVRGSVMNPCDHPHGGGEGRAPIGRTRPLTPWGKPALGMKTRKKKKSY
VLRRMSRIGKLPIKVPENVAVTCDGLNITVKGKFGTLENKLPEVLGVDGTLIVSRTTR
ALHGLYRSLINNMVIGVSEQFALTLNLQGVGYRAAVQGKCLTLNLGYSHPVKMDIPE
EISVEVVQNTTINLKACNKESLGLFAANVRSWRPPEPYKGKGIIYQGEIIKRKAGKSGK
MTFIPDSNKRKWYVIDCQNQKLGRLLSTIVPLLTGKVKPYYHPALDIGDCIILINAEAL
TLSDFRKFHVYCPGRPGSSLKQVLNNLPQQIIERSIRCMMPNGEKNLAKRLKIYEGPN
HPHIAQNPIELMPKKITALIKLALPAGKATPAPPVGPALGQHGVNIAAFCKEYNARTS
DKSGLIVPVEISVYEDRSYTFVLKTPPASVLLANAAQIKKGSSTPNKLNVGSVTTDQLK
EIATIKLPDLNTTKLDSAMKIVEGTARNMGISIMLSPKRTKYRKYHRGRMKGKATRGN
EISFGDFALQALEPTWITSRQIEAARRTITRYTKRGASLWIRIFPDKSVTARAAESRMGS
GKGSVDYWVAVVKPGTILFEIGSVPEEIARAALSLASYKLPIKTKFIIRMIYPQTILTVAD
NTGARKIMCIRILGGNKKYAKIGDTIIGVVKEAVPNMPIKRSDIVRAIVVRTCKTIRRQD
GMYIRFDDNAAVIVNLDNNPRGTRVFGPVAREIRDKNFSKIVSLAPEVLMYAIVEISGR
QFWIETGKYYDLNRIPTGKQITLNRVLLVNEILIGQPYLEVVKGKILEHLRGRKTIVYK

MRPKKKTRKKQGHRQELTRVLIMVKAVAKYIRMSPHKVRRVLNQIRGRSYQEALMIL
EFLPYDAGGPVWQVVHSVAANAKNNYGLDKKKLIIDEVFADEGPKMKRIRPRAQGRA
FKILKPTCHITVVVMPKSDIHPKWFKNTPILCDGKPLCYVGSTKSQLQIDVWLANHPF
YTKSQIIVDSEGRVERFMKKYQLMAHKKGAGSTKNGRDSNAKRLGVKRFGGQVVKA
GNILIRQRGMKFKPGLNVGCGKDFTLFALKDGVVKFDVNIIMKVRPSVKKMCEKCRV
IKRHGKILVICSNPKHKQRQGMAVPKKRTSKAKKNARKANWKKKADKAAQKSLSLAK
SLLKTTSFLYMIKCLKSEKEYGQFLINPLQPGQGITIGNLLRRVLLSDLGGTTITAIRIA
GVKDEFSIISGVREDILEILLNLKGVILKNKFGRLKVQGPAVVTADSIEVPTDLEIVNPN
HYIATISTSNVLEIEFKFEYGIGYKLAFLQTDAIFMPVQKVDFKIENITERLFLDIWTNGS
ISPDEAVFSASKFIIDFFNILITIEELQLSVRAYNCLKRAQINTIGDLLQYSPLQELFGRKS
ADEVFATLKNRNTLISKKQLKQLLAWSFTKYDSMQACSLADELKYLGFKYASQAGISIS
IEDLKVPFIKQLMLQKANQEILNAEKICLKGKITDVERFQKIIDTWSMTSESLKNEVVS
YFKSYDPLNSVYIMAFSGARGNLSQVRQLVGMRGLMSDPSGEIMKLPIKKNFREGLTI
TDYLMSGYGARKGIVDTALKTANSGYLTRRLIDVAQDIIVREKDCLTTHSFLILIGRLL
NKPIYIAELNTQITAQLVRTFTNINNFYIRSPFTCNLYRSICQKCYGWDLASENLVDIGE
AIGIIAGQSIGEPGTQLTMRTFHTGGIFTEASQQITSPTDGIIKFSKILKTIILRTNRGED
VVITKSSGSLIIIEVVHIELLRNTILFVKQNQYVKRNSIIGELTNNEKQIFTNTSGEIFMNS
LLWILSGQLYKAPYNLYPDHKINKSYIYRTKLINHYSGIIKNCIFQGGLILPQETYTLNC
DMLLVGNFIFEIIPGIFSKTSGIVLILVHTVSIKCGKVFFPGELLLNNIKITKPSFCELIRPL
DIYEIVNLVSNILNFIDSSLLVEKNQFVDRYVILGYLESVVNALEVVKFKSKIRQIFLISNK
DCLTIEKKYNKKDLIDLNATGKILVNNNKRVTIQKGRPYFFKELNTVYCKNGEFIEDG
ETLGLLNFEKEITGDIVQGLPRIEELLEARKNQTNQKKVQKLGTTIQINPHNFLKVYF
NYYEASYRSFKKVQALILNSVQAVYISQGVSIADKHLEVVIKQMTTKVLITYEGNTPLL
PREVIDLYHIQYINKIVKNHFYVPLLLGITKASLNNPSFISAASFQETTRVLTKAAIEGRI
DWLRGLKENIIIGHLIPAGTGMYITALPDFLAMQRVSFCWFITQGLNEELSNFARMRD
FANTQYILFGQEYTLVKPVYKITKAKKYITNYSVQLIVPLEVRNTKVRYHNQFSVINLP
LMTTSATFIINGCERVIVSQIIRSPGVYFLYTAILIPEYGSWIRFGFKKIIIQLDKITQKPIIH
LLKEMGLTEICKNLQYSDFFYFSEFSRIFDETYYKLGKIGRFKLNNRLNLKLNQQLYTL
TYEDIFAIIDQLLALSLSKDDIDHLKNRRVRSIGELLQNLFQVSFQKLQRILQNQINKFII
TTIREFFGSSQLSQYMDQTNPLSSLTHRRRISGLGPGGFDRDRISFAVRDIHPSHYGRI
CPIETPEGQNVGLIASLTTCARVNKSGFLETPFWRVLNGKVMKTGNPIYLTADIEDFY
KIAPADISTNYLTKNLISVRYKQDFINVTPFDVDFIAISPIQVVSIAASLIPFFEHDDANR
ALMGSNMQRQSVPLILPQKPIVGTGLENQVAIDSGMTFNARFSGIVEFVTADRIILKYS
LQKYQRSNQETCINNRPIVWCGEKVKSGQILTDGPGIVTNELALGQNVLVAYMPWQ
GYNFEDAILINERLVYEDVFTSIHIERYEIEIDRTLEICETNNIPNLLQYLNEDGIVSIGTF
VKPGDILVGKIIAKDDSEQLPESKLLRAIFVKDTSFRMPVVETLTFNRIFIAQIRKIQVG

DKIAGRHGNKGIISRILPRQDMPFLPDGTPVDIILNPLGVPSRMNVGQLYECLLGLAG
HKLNRRFKILPFDEMYGQEISRILINKKLRQASNNEAWLFNPYAPGKMVLIDGRTGHE
FENPITVGNAYMLKLIHLVDDKMHSRATGPYSLVTQQPLGGKAQHGGQRFGEMEV
WALEGFGAAFTLKELLTIKSDDMDGRNETLNAIPPSGIPESFKVLLQELRSIGLDMSTY
KIEKFADVEVNLIMFDYIKIKLASPVKILQWSYRQLP-
NGLVGEVHKSDTINYRTFKPEMDGLFCERIFGPSKSLECACGKYKICERCGVELTESRV
RRHRMGHINLIYPVTHVWYVHSRPNYMALLLIKRIKGSALIKNQLEKLDLYFELSKSRK
FIRKISKLRNLEIKRIRILENLLATGSQPAWMILTILPVIPPALRPMIQLEGGRFATSDLN
ELYRRIITRNNRLLRLLEIDAPQLIIRNEKRMLQEAVDTLIDNGKRGKIALSANNRPLKS
LSDIIKGKHGRFRQNLLGKRVDYSGRSVIVVGPSLKLNQCGLPYEMALELFQPFIILIN
QGAKLIQKNSFLDGVLKVLSNHPIFLNRAPTLHRLGIQAFEPILVQGRAIKLHPLVCSA
FNADFDGDQMAVHIPLSLEAQAECYMLMLAPYNFLSPANGEPIILPSQDMVLGCYYL
TVDNISGLLGSTHYFSGLEDVLLAYNQDKIELHSSIWVRYVLVQYLQTTTGRVIFNYT
VQKSLMSRYRGPKLRITRRLGLPGLTQKSSKKQNNKKTEYGLRLEEKQKLKFNYGLTE
GQLFRYIKEARRREGVTGLILLQLLETRLDTICFVLGFAPTIASARQLVNHGHVTVNG
KVISIPSFQCRINDVVGSKPKSSSKNLIENNLYPTHLEFDKTKLEGTVKNYCERSELLLE
LDELLVIEYYSRMGQKTHPLGFRLGITQEHRSTWYANFNQYATLLKEDDSIRTYLQKL
ISNVQINRNNQIELNIETGIPALVLLNIKKILYQLTINIIEVEKANLNASLLADLIVNQLE
QRIPFKRAMREALQSAENGIKIQVSGRLNGAEIARSEWLREGRVPLQTLRADIDYAAKE
ANTIYGVLGIKVWVFKMVTDTISDMLTRIRNAHMVKHQIVEIPATKMSIAISSILKEEG
FIQDFESYLKKYLLLSLKYVEKSREPVIANLQRVSKPGLRVYANSKKLPKVLNNLGIAIIS
TSKGVMTNLKAKELGIGGEVLCYIWMFVERLIKISRVSKVTKGGKKLSFRAIVVVGDEN
GQVGVGVAKADDVVNAFKKAKTDGRKNLIKIPITKSLSIPHKVTGNFGACKLIMRPSI
EGSGVIAGGAVRSVLEVAGIKNVIAKQLGSNNLLNNARASIVALNSLMSRRNIAKKRFP
EPDSTYNSYLVSLLINRILKAGKKNIAQNIVNNAFEIIKTKTNEDPLVVFEKAVKNASPIV
EVKARRIGGSTYQVPIEVSSFRATNLSLRWIIQYSRQRVGRTMSIKLANEIIDTANDIGN
TIKKKEETHKMADANKAFAHFRRMIRVRLESFNHELLTLSCRKIMDTIQLNPLNSVGLI
PLPTNKRIYCVLRSPHVDKDSREHFEVRVHRRILDIFYDPDIIDLLSDLPSGVLYKIMPT
IQQLVRSRRIQIKKKTKCPALVSCPQRRGVCTRVYTTTPKKPNSAIRKVARVRLTSGFE
VTAYIPGIGHNLQEHSVVLIRGGRVKDLPGVRYHIVRGALDSGGVKDRTQGRSKYGV
KKPKMKKNKNSYINGVVHIQSTFNNTIVTITNSTGDTISWASAGSSGFKGARKSTPFA
AQTAAEKAALDALSTGMKSVEIFVKGQGAGRETAIRSIQGAGFEITAIQDITPVPHNG
CRPPKRRRVMVRLIGVDLPRNKRIAYALTYIHGIGLESAKKIIELANISETRTDDLTTEE
TIALRDTLEDLKLEGDLRRFNGLNIKRLNEINCFRGKRHRNSLPVRGQRTRTNARSKR
GSKKTKKMPVKERIGIVVSNKMQKTIVVKVENRYSHPIYSKTMVKTKKYLAHDEMSEC
NIGDQVLVQECRPLSKRKRWVLAMAKKSMIEREKKRIKLNNKYESKRVSLLNEYNSIT

DFDLKLAAHSKIQQLPRNSSKIRIRNRCWKTGRPRGFYRDFGISRHVLREMAHQCLLP
GVTKSSWMSRSLKKAPFVAYHLLKKVNTMKKDVIKTWSRSSTILPSMIGHTIAVYNGK
QHVPIFISDQLVGHKLGEFVSTRTFRSHKTDRKTKRMNKSAVKRILINKRNKVQNRFY
KSSVRTLTKMFLKTVENYAQTILNSIYSKIDKGLKRNVFHKNTAARKKSQLAASLKSM
TQIFDESGNIIPVTILKIGPCVVTQIKTVLNDGYNAIQIGYGDSKALTQPRLGHLQKSNI
QPLKYLKEFRVTNEEFEVGQILNVDLLNDNKFVNVRGKTIGKGFSGLQKRHNFTRGP
MTHGSKNHRAPGSIGMGTTPGRVLPGKKMSGQLGNKIKNIRKVKVIQLNEKDNLLIV
KGSVPGKPGNLLSIIMAKNKGSRILITLECTECRSNNKRSAGVSRYTTQKNRRNNPERL
ELKKYCAHCNKPTIHKEIKMTQIFDESGNIIPVTILKIGPCVVTQIKTVLNDGYNAIQIG
YGDSKALTQPRLGHLQKSNIQPLKYLKEFRVTNEEFEVGQILNVDLLNDNKFVNVRG
KTIGKGFSGLQKRHNFTRGPMTHGSKNHRAPGSIGMGTTPGRVLPGKKMSGQLGN
KIKNIRKVKVIQLNEKDNLLIVKGSVPGKPGNLLSIIMQKFVTYNSFKNGQKLELNVLN
SGNYLIHKDILRHQISQKQGTASSKTRSEVRGGGRKPWQQKGTGRARAGSNSSPLWK
GGGVTFGPKPKNVVLKLNKKERKLALQTLLYNRRNNILVIDNLIEPKTKTFSELCINLE
QKVLIIVSEKTNNLKLSTRNIKNIELILASNLNTLSLLKAKQILCTSAAIQNIKEIYMLKLR
LKRSGRKRQPSYRLVVMENSSRRDGRPVEQVGYYNPITKQSHFDIAKIQKWLDCGVK
PTETVANLLTKILMIIKSPIITDKATRLLEKNQYSFIVNPSSDKITIKATIEYLFNVKVIKIN
TCHLPKKMVRKSHYKKAIVKLAAGDKIELFAMTLEKKSLPKVSIGDNVKIGVKIIEGNK
ERIQFYEGTVIAKKNSSINCTITVRKVLQGIGIERIFLIHSPKVDSITVLRSSKVRRSKLYY
LRNLKGKASRLKQRF
>Aureococcus_anophagefferens_strain_CCMP_1984_GQ231541
MLRSEYPKLVKIKINRGLGLSAQNSAVLNKTINEFRVITGQQPVVTLARNSVASFKIREE
MPLGVTVTLRGDKMYSFLDRFINIALPRSRDFQGLNPKGFDKFGNYTVGLTEQLLFP
EISYDSVDQARGFNITFVTNAKTRDAGAMLLREFGLPIMVRIKRGNVARKRRKAVLKL
AKGYKGAHSRLFRVSNQQVMKARRYSYVSRKLFKRVQRRNWITCINSQVRRTTYSQV
CSKFKQNKIAINRKMLSEIIINDPLTFDYLL---

MSRIGKLPITLPKGVTLKVDDNMVSIKGPLGELSRQIPPEILIEEKAIVTKRTRALHGLV
RTLVNNMVVGVHTKFEIDLELKGVGYRCQAAKDKVTLSLGFSHPIVLPLPKGVEVSV
EANTNIKVSGIDKEEVGFIASKIRSFRPPEPYNGKGVLYKNEVILRKAGKAGKMN--
PLADNKKWYVIDAKGKTLGRVSTLIATIISGKHKSIYNPSFNCGDYVIAINVEEIQVKAS
QKIYRRHSGRPGETFEKLQERLPEKILENAVRGMLPKGGRELYKNLKVYKGESHPHTS
QNPELLMPKKIVAIIKLALPAGKATPAPPVGPALGQHGLNIMNFCKEYNAKTGDKGD
LIIPVEISAYEDRSFSFILKTPPASVLLAKAANVAKGSGEPNKTNVGSITQEQLEEIAKTK

LPDLNARKLSSAMKIVEGTARNMGITIMLSPKRTKFRKQQRGRMKGRALRNNTVAFG
EYGLQVQECGWLTSRQLEATRRTITRYTKRGGKLWIKVFPDKSITARAAESRMGSGK
GTPEYWVAVVKPGNIIFEIAGVKPDVARQAMNLAAYKLPLKTKFISK-------------------
----
MYAVIEIGGKQLLVEEGKYYATNRLPHGSSLSLQRILLCNRRLVGHPYLEVIKATVLEH
FKDSKITVFKMKPKKKTRWTKGHRQAQTRIMIMAQAVARYIRMSPTKVRRVLNQIRG
CTYEEALILLEFLPYRACEPVWQVVQSAAANVQSKHGLNKQDLIIREVFVSPGPVLKRF
RPRAQGRAFAIRKPTCHITAVVMPKKDLHPEWYENSEVYCDGQLVYTVSSTKEKLNV
DIWSGNHPYYTGSKKLLDTEGRVDRFMKKYNLMAHKKGAGSTKNGRDSNAKRLGIK
CCGGQYVTSGNILMRQRSLKIKPGLNVGCGRDYTLYALSDGYVKFEVNIVMKVRPSV
KKMCPKCRVIRRFGVIRVICPNPKHKQRQG---
MFEKIESYSDSSRFLLKELTQGQGLTIGNTLRRVLLTELEGTAITAVRINGINNEFATIP
GVREDVLEILLNLKQIKFTGLFTHVSVQGPQLVTAANISLPDGLSLLNSNHYIAAVAEQ
TNFEIELKIESGTGYNFTFLSLDSIFTPVKNVNYQIKDITEILDLEIITNGSITPNDALNK
AALVLQTLFGSLILIEELQLSVRAYNCLKRVNIKTIADLVQYSVLKDIFGQKSANEVVEK
LQDYNKTINKKELKSIVHSTFQSYGIIKATNLAEKLKKTGFAFATQAGISISIEDLKVPPT
KDTLFAKNNTQINLAYFYEKRGNINEIERFQKVIDTWHTTSEILKNQLVDFFKTSDPL
NPVYMMAFSGARGNLSQVRQLVGMRGLMSDPNGQIIDLPIKTNFREGLSITDYVISSY
GARKGIVDTALRTADSGYLTRRLVDVAQHVIIRELDCQTKNGVRLYLGRVLAQPVIIL
ERNTTLTVAELKKIKF-
TNFVLRSPLICESSRSICQKCYGWNLSQGQLVELADAVGVIAAQSIGEPGTQLTMRTF
HTGGVFTESSNQIRSTVSGQILFSANLKTVTSRTVYGEVLLKAQNSSEFFIFTLKRYPVK
PEMLIFVDNKSFVTVGDVIAELPLANKRVFTTVSGEVQFIDLIWIAEGEIYDLFSKMRG-
SKIVKNAIAQTKIISPIGGMVNLFLLNPHLLCPEKMDVSSSK--------------------------
PQTDIESFKIVYPGEVICDSISISKLSYCYFIVPLKQGIIFAEVRLEFSTTSVSNFISENQFID
PYSVIATLNIIADTLAIENVKNVPNRFLISLSKSYKECAPISSKFDKMISDYSGYVIRQKSS
HVKIRLSTPFFVSQGTRILINHGSLIRQGESLCQLVYTRVISDDIVTGLPRIEQLLESRLE
KKNNDQIEKVAQPVDIHPHNVLNVYFQYYKATKRSVTSIQILLLNLVQDVYRSQGIYIS
DKHVEIIVRQITSKVKIKYTSETTFPVGEFIEFEQVQYVNRAFHLTEYEPVLLGITKVSLL
TESFISAASFQETTRILTQAAVEGKVEWLRGLKENVILGRLIPAGTGLFVTTIPHFAEAN
LNSFCWFLESGLSDELRNFSSALNLQKLNIKIYSDEFILKRPKYSAVQCKRYDLTYAIRL
YVPIELIEEDSVNECLAFFGEIPLMTEEGTFIINGCERVIINQIIRSPGIYYLYSATLISDRG
SWLKFEFETVQIRLNKTTTVDFYQLLETCGLVTILNSAAKTEITSDSSFLQIFNEKIYSL
GKIGRKKLNSKLGLSISDSVTRVTIEDVIAISNYLVNLHGNNDDIDNLRNRRVRSIGELL
QIQFGIGLSRLERSINERLVIAIIASIKEFFGSSQLSQFMDQTNPIASLTHKRRISSLGPGG
LNRDRLSLAVRDIHPSHYGRICPIETPEGQNAGIIASLSCYARINESGFLETPFFKVKNG

KVQNDELPTYLTTEEEELILTAPADRVLNSFKQQLVPVRYKNEFSLVPINQVKLVSVSP
LQNFSIATALIPFLEHDDANRALMGSNMQRQSVPLLYPHKPIVGTGLEHQLAADSGA
VVISKFAGDVKYVSSRLISIRYKLQKYVRSNQDTCVNQRPIVWPSEKVESGQVIADGPG
TNGGELALGQNLLVAYMPWEGYNYEDAILVNERLVQEDLFTSIHIEKFDMEVRQTSF
GVETRDLPNIISNLDRNGIICKGTFVKSGDILIGKVTPKDETDQLPEGRLLRAIFVSDTS
LRVPVINVRVFRRIFIAQIRKIQVGDKIAGRHGNKGIVSRILPQQDMPFLPNGVPVDILL
NPLGVPSRMNVGQIFECLLGLAGSKLNRRFKVMPFDEMYGVETSRNLVNRYLNMAA
SQEPWVFNSYLPGRVALRDGRTGQLFDNPVLVGKTYMLKLIHQVDDKMHARSTGPY
SLITQQPLGGKSRHGGQRFGEMEVWALQAFGCAYTLQELLTLKSDDMEGRNEVLNA
IPRPGIPESFKVLIRELHSLGLDISLYKMDKANETEVDLLMFDYIKINLASPERIKQWAC
HTLR-
DDTAGEVTKSETINYRTFKPEMGGLFCEKIFGPVKSWECHCGKYKICERCGVEVTESR
VRRHRMGYIQLEYPVAHIWYVRGFPSYLSLLLFRQFDGAELIFDLLSSLDLEKVVEECR
TAYRTASSDKEKIIKRVRILENFIATKSNPTWMLLSVIPVLPPGLRPMVQLEGGRFATS
DLNELYRRVINRNNRLKRFFAIYAPEIIIRNEKRMLQEAVDALIDNGHREKKAVGLNNR
PLKSLSDILSGKQGRFRQNLLGKRVDYSGRSVIVVGPELKLNQCGLPYEIACELFQPFLI
LIQLGAKLIQEKIRIWEGLNVLDGFPIFLNRAPTLHRLGIQAFEPIIVDGRAIKLHPLVCP
AFNADFDGDQMAIHVPLSIDAQAESYLLMLGPNNFMSPATGEPILLPSQDMVLGSHY
LTSQNRPSAKGNHHYFANMDSVLQAYNCDKIALHSSIWLKFILVTYILTTPGRVLFNN
LLNSVVMSRYRGPKIRIVRRLGLPGLTAKTTTREKQNNSSYSIRLQEKQKLRYNYGLTE
KQLFSYVKEARRLKGSTGSVLIQLLEMRLDNIVYRLGIGNTIPASRQIVNHGHIYVNGK
KVTIPSFQCSPNDVIEVRNRVTSKELAKQYLVPQYLEFEKDNLKGKVKRVAENTETNL
NINELLIVEYYSRMGQKVHPLGFRLNTTQKHKSTWFAQINQYSSLLEQDAKIREYITA
NISDIEIERTNNIFLKIYVAKPVIILTKLKSLLNKISIDVFEVAEPDTHANLLAQFIANQLV
RRVAFKRAVRKAIDRAAKGIKVQIAGRLNGAEIARTEWVKEGRMPLQTLRAEIDYATA
SAKTIYGILGVKVWLFKMVNDVISDMLTRIRNASKVKHHLVQVPFTKMTLAIALVLKE
ENFIEDFQEFSRKFLLILLKYKGKGRQPAITTLKRISKPGLRVYSNVKDLPVVLGNFGVA
LVSTSKGVMTNFKAAKLGVGGEVLCYIWMWVRKVVKVRRITKVVKGGKKLRFGALVI
IGNEKGLVGVGVGKADDVVEAVKKAANDGRRNLIMVPLTKTFSIPHVSTGQFGASNV
LLKPASAGSGVIAGGSVRTLLEAAGVQNIMAKQLGSNNLLNNARATVCALQALMSRR
KIIKKRFPSPDPVYNNYLVSILVSRLIKDGKKTLAQNIVCDALEIIAYKMETDPIEIFEKA
VKNVTPLVEVKARRFGGATYQVPMEVTSFRGSNLALRWIVKASRKRTGKTIALKLAAE
LMDASKSSGDAIRKKQESHKMAEANKAFAHFRRMTRVRLESFDHKLLTIACNSLIDGI
SSINGIAKGPIPLPTKKRIYCVLRSPHVDKKSREHFEIKIHKRLLEIYTPQNIADSFLAIPP
GVSIKLMPTIQQLIRTKRQSLKKKTKSPALKNCPQRRGVCTRVYTTTPKKPNSATRKV
ARVRLTSGFEVTAYIPGIGHELQEHASVLIRGGRVKDLPGVRYHIIRGTLDSGAVKNRT

QGRSKYGVKKPSMKKQKKNIVSGVVHINSTFNNTIITITDLAGNTISWSSAGSVGFKGA
RKGTPFAAQTATEAASTAAMGQGLRKVEVLIRGQGSGRETSVRALEVLGLDIVSIKDI
TPIPHNGCRPPKRRRVMVRISGIDLPNSKRIEYALTSVYGIGLTTSRSILAVADIDSKRT
NVLGDTEVMALREVIENYKVEEDLRRQTKQNIVRLSQINCVKGRRHRQNLPVRGQRT
RTNSRTRRGARLM--
MARRQKIGIVVSDKMEKSIVVATETRYKHNLYGKIMSKTKRYLVHDPENTCTIGDTVI
VEEHPPISAKKRWIFKMAKKSMIEREKKRQRLVLKYREKRRTLLNAIKTSESFSETLTLQ
KKLQKLPLNSATNRLRNRCWKTGRSRAVYRDFGLSRHELREMAHEGLLPGVTKSSW
MGRSIKKGPFVAYHLLKKVDALKLEVIKTWSRASTIMPSMIGHTIAVYNGRIHVPVLIS
EQLIGHKLGEFAPTRTFRSHKADKKARR--

MTQIVDDLGNLVPVTILRAGPCTVTQIKTVETDGYSAAQLGYAQSKVLTKPQIGHLE
KVGAPLLRYLHEYKISDSELSLGQTIGVDIFSAGSVVDVTGKSIGKGFAGLQKRYNFGR
GPMTHGSKNHRAPGSIGAGTTPGRVYPGKKMAGHLGAKQVTIKKLKVLIVDAENNLI
IVKGAVPGKPGNLISIQMAKTKGIRVIITLECTDCRTNNKRSAGISRYTTMKNRRNTPN
RLEIKKFCPNCNRHTVHKEIKMTQIVDDLGNLVPVTILRAGPCTVTQIKTVETDGYSA
AQLGYAQSKVLTKPQIGHLEKVGAPLLRYLHEYKISDSELSLGQTIGVDIFSAGSVVDV
TGKSIGKGFAGLQKRYNFGRGPMTHGSKNHRAPGSIGAGTTPGRVYPGKKMAGHLG
AKQVTIKKLKVLIVDAENNLIIVKGAVPGKPGNLISIQMQKFVVYDSVITGQKLKLNVV
KSGNYLIYKDLLRHQISNKQGTASTKTRSEVRGGGRKPWKQKGTGRARAGSNRSPLW
KGGGVIFGPKPQKIILKLNKKERKLALQTLLYNKRNLILIIDNLLEPKTKTFLNICLNLN
QKILVIVSEKTKFLKLSTRNIKNIKLISASNLNTLSLLKAKQILITSSAITYIKEIYMLKLRL
KRGGRKGQPAYRVVIMESNSRRDGRPIEDLGYYNPISKKFLVSFDRISVRLKQGVQPT
QAVKNLLKRIAMEIKQISTTKKALWSLEDKKYTFLASPFVTKEWMKRAIEICFNVRVIK
VNTSNLPTKKKRKAKLKKVIVTLDPNDEIPLFS---

>Chroomonas_placoidea_KY856941.1
MLRSDYPKVTKIVINRGLGEASKNNKALENSVKEIALITGQQPVVTKARKSVAGFKIRE
GVPVGIAVTLRKNLMYSFLERFTNLSLPRIRDFKGISIKSFDGRGNYNIGIKEQLMFPEI
EYDRVDQIRGLDIAITTTAKTQQEGIALLKALGMPFMARVKRGNVARKRRKKILSLAK
GFRGSSSKLFRIANQQVMKALRYAYVGRKRKKREFRSLWITRINAAARLTNYSSLINTL
KVSKVSINRKMLAQIAVSDRGSFTQILMAIRIYKAYTPGTRSRSNSDFSEITKKKPEKSLL
AKKVSTGGRNNRGVITTRHKGGGHKQRYRIIDFKRKKYDIEAKVAAIEYDPNRNARIA
LLHYKDGEKRYILQPRTLNVGDMISSGENAPLEVGNALPLNNIPLGISVHNIELIPGKG
GQLARSAGSYAQLVAKDGDFVTLKLPSNEVRLVSNKCYATIGQVGNVEHNNIRSGKA
GRHRWLGIRPAVRGVVMNPVDHPHGGGEGRSPIGRARPVTPWGKPALGVKTRKRN

KSCIIRRMSRIGKIPVKIPDKINVTIEGDQITVKGPKGQLSREIPDNILVIENIQVSIKSQQ
IYGLYRRLVANMVQGVSEGFQKKLTLQGVGYRSQVQGKKLILSVGYSHQVEIPAPDGI
IISVEANTNVLVSGIDKELVGQVAANIRSIRPPEPYKGKGIRYEGEYVRQKVGKAGKMT
ILNTTETYKWYLIDAESKKLGRLSSEISKILLGKNAPEYSPNHNIRHSVIVINAEKVEVKR
TDKFYRRHSGRPGETFQELQDRIPTRIIEKSVKGMLPKTGRTLFSHLKVYSGNVHPHIS
QKPELLMAKKVIGMIKLAIEAGKATPAPPIGPALGQKGVNIVMFCKEYNARTADKAGY
IIPVEITVYEDKSYTFILKTPPASVLLVKAAGISKGSGTPKEKKVGNITTQQLKEIAETKL
PDLNTKKVEAAMKIIEGTARNMGITIMLSPKRTKYRKPHRGRLRGTATRGNSVVFGD
YGLQALEPTWLTSRQIEATRRSITRFVKRSGKLWIRVFPDKSITARAAESRMGSGKGAP
DYWVAVIKPGHILFEIDGVPQLTAYQALKNASYKLPIKTKFIGRMIQTQTYLNVADNS
GAKKIMCIRILGSNKKYASIGDVIIGVVKVASPNMPIKRSDVVRAVVMRTKKTIKRKDG
MSIRFDDNAAVIINKENNPRGTRVFGPVAKELRDKDFTKIVSLAPEVLMYAIIEASGRQ
FWVEPGRFYDFNHLNLGDKIALTRVLLVNNVSVGRPCLDAVEATVLGHIKSRKVTVY
KMRPKKKTRKKQGYRSNLTRVMIMVKAVAKYIRMSPNKVRRVLNQIRGKSYKEALML
LEFMPYSACKPVLQVLQSAAANAQNNNGMQKSELLVNKAYADQGPVLKRFRPRAQG
RGFKIKKPTCHITVYVMPKKDIHPTWYPESKVYCDGQLIMTVGSTKAELNVDIWSGN
HPFYTGSQRILDSEGRVERFMRKYGMMAHKKGSGSTKNGRDSNSKRLGVKKYGGEA
VLAGNILVRQRGTTFKPGNNVGIGRDNTLYSLVTGKVCFELSVYMKVVSSIKNRSKDC
QIVKRRGRTYVICSDPRLKVRQGMAVPKKRTSKTKSKSRLANWTHKANIQAKRALSLA
KSVASSTSFVYMIECIDSRRDYDKFILEPLDQGQGITVGNSLRRVLLADLQGTAIVSVRI
AGVNHEFSTIPGIREDVLEILLNLKEIIFKSDIGRVRVQGPAIVTANDFELPPELTLVDP
KQYVATICGNNVLEMEFRIEKGKGYRLVFLQIDSIFMPVKKVNYIVEDIQDRLILEVWT
NGSISPKEAISQSSNILTNLFNPLILIEELQLSVRAYNCLKRAQIHSVADLLDYSQEDLLF
GQKSAEEVIDALKKTNKVIDKKELKKLMSWAFSNYGTGRSSYLADKLKDLGFHYATR
AGISLSVEDLKVPPTKRDLLNKTNEEIKFTEQKYQRGEITTVERFQKVIDTWNNASESL
KDEVINYFKETDPLNPIYMMAFSGARGNISQVRQLVGMRGLMSDPQGQIIDLPIKSNF
REGLTTTEYIISSYGARKGLVDTALRTADSGYLTRRLVDVSQDIIIREIDCGTTRGILLL
LGRVLFETIYIAHINQDLDYETATKITSVKSVIVRSPLTCDSSRSVCQFCYGWNLAHGS
LVDLGEAIGIIAAQSIGEPGTQLTMRTFHTGGVFTELAEQIKAPFDGLLRFPTDVRTRL
IRTRHGEEAVVLEEGLQLTLYSKSKLELKQGTMLFLSDNEQFKKGQVIAEMSARTKDL
STEISGQVYFSDLLWILEGDVYTVPSFSQIAVKEQDDVLAFTQVVSDYGGIVRLFLMT
GGIIISEETHEINKDLLYVEQGQQEILKDLYNNIEGYVSVIVREVVIKPGYIVEIGEEIIPG
KIATERMFVELVRPIKNYLIVEVLKTYLILTVTTLSVQDNQKISSGTIVAQTKLLCASSG
QVKSNNASRKILVSTPANEKRIELNGLTANNIIASNSGQIVYLDTNTIIIRSGCPYLISSG
AILQITNLDLVQRGDILAILVFERSKTGDIVQGLPRIEEILEGRKPKEPCKLSIGLASPITP
NPHEMLSIFFGFYDAAKFSLQKVQLYLVNEVQSVYQSQNVDISDKHIEVIVRQMTSKV

KVEDGGDTTLLPGELVELQQIDNINEAMTLTTYSPMLLGITKSSLNTDSFISAASFQE
TTRVLTEAAIEGKADWLRGLKENVIIGRLIPAGTGMSTFILPDLVEIQRASFCWFLEEG
LSEEINSFSPITDTGNLELYLLGDKYKLKCPKYSITESKRRDSTYSVQVYVPARLRETGV
IKEQEVFIGDLPLMTDRGTFVINGAERVIVNQIVRSPGIYYTYSASLISNRGAWVKFEID
KVWVRIDKTRKIPAHVFLKAMGLSDIYNGLKNPDYLKKTELSRFFDPKRYDLGKVGRY
KLNKKLRLSIPESIRVLTPQDALSSLDYLINLKFEIDDIDHLGNRRVRSIGELLQNQVRV
GLNRLERIIRERMTIKIIAAIREFFGSSQLSQFMDQTNPLAELTHKRRVSALGPGGLSRD
RAGFAVRDIHPSHYGRICPIETPEGPNAGLIGVLATHARINAYGFIETPFYKVEAGRVL
NEVPPIYLTADQENDIWVAPGDIPLDTIIGDAIPVRYRQEFTTSRPDQVDYIGVSPLQV
ISIATSLIPFLEHDDANRALMGSNMQRQAVPLLFPESPLVGTGLEAQAARDSGMVTVS
RSKGTVTYVSADEICITYHLHKYQRSNQDTCINQRPSVWLGEEVVPGQVLADGAATE
GGELALGQNILVAYLPWEGYNFEDAFLISERLVYNDVYTSVHIEKFEIEARQTKLGSET
RELPNVLRKLDDNGIINVGSWVEAGDILVGKVTPKGESDQPPEGKLLRAIFVRDTSLR
VPILDIRIFTRVYIAQSRKIQVGDKMAGRHGNKGIISKILPRQDMPYLPDGTPVDLVLN
PLGVPSRMNVGQIFECLLGLAAENLNQRFKIIPFDEMNGAEASRVLINEKLIKATSGKD
WIFDLRHPGKTPLFDGRTGERFDNPVMLGISYMLKLVHLVDDKIHARSTGPYSLVTQ
QPLGGKAQHGGQRLGEMEVWALEAFGASYTLQELLTVKSDDMQGRNETLNAIPRP
GTPESFKVLMRELQSLCLDIGAYKIENLRGVEVDLMMFDYVKINLASPERIKKWGERT
LP-
NGVVGEVTKPETINYRTLKPEMDGLFCERIFGPIKDWECHCGKYKVCERCGVEVAES
KVRRHRMGYVQLAAAVTHVWYLKGLPSFISILLDISLKGAEAIQKLLKDIDLEVEAESL
REEVLSAGLKRDKAIKRLRVIDNFIATRSDPSWMILTILPVIPPDLRPMVQLDGGRFAT
SDLNDLYRRVLNRNNRLIRLQEILAPEIIIRNEKRMLQESVDALIDNGRRGRTVIGANN
RPLKSLSDIIEGKQGRFRQNLLGKRVDYSGRSVIVVGPQLQLNQCGLPREMALELFQP
FVILIHQGAKMIQRNPVVWDVLDVMQGHPVLLNRAPTLHRLGIQAFEPILVEGRAIKL
HPLVCTAFNADFDGDQMAVHIPLSLEAQAEARLLMLAPYNSLSPATGEPIIMPSQDM
VLGCYYLTTENLSQNNNQLYYFSSLDDVLLAYEQKKIRLHSYVWVRYMIAQYLFSTPG
RILFNKVVQDSLMSRYRGAVLRIVRRLGLPGLTRKVSKRSPRKPSEYAIRLEEKQKIRFN
YGLTEKQLLRYVRDAKRIKGSTGEALLQLLEMRLDNIAFRLGMAPTIPAARQLVNHG
HILVNGKRVSIASYQCRTGDSISIRNNPKSRKLVENYLIPSHLELDKPNFTGKVNGIIER
DWVALQLNELLIVEYYSRMGQKTHPLGFRLGITQTHRSSWFEVAKNYPKTIEEDFIIRS
YVEKELSKILINRKDQIEIDIHASRPGVIVLDLNKLTKQIRINVSELSKPDGDAALIAEFIA
QQLEKRVAFRRATRQAIQKARKGIKVQVSGRLNGAEIARTEWVREGRVPLQTLRADI
DYATKKAFTIYGILGIKVWVFNMVNDTIADMLTRVRNANLARHQIVQVPATKMTKNI
AAVLHEEGFVQSVEEVGAQQLLISLKYKGKTRQPVITALKRISKPGLRIYANRKELPRV
LGGLGIAVISTSQGVMTDAKARSQGLGGEVLCYIWMWEERVVQIRRVTKVVKGGKK

LSFRAILVIGNEKGQVGVGVGKASDVIGAVKKAVTDGKKNLVTIPMTKDSSIPHIITGR
SGAAKVIMRPSAPGSGVIAGGAVRTMLELGGVKNILAKQLGSGNPLNNARAAADALA
KLMSRRTTAKKRLAVPDPIYNSRLVSMLTVRILQEGKKHLAQRIIYQALDIIKEKTNED
PLTVLETAVRKVTPLVEVKAKRVGGSTYQVPMEVRAFRGTNLALRWITKFSRERSGKS
MAMKLANELMDSANETGNSIRKREETHRMAEANKAFAHFRRMIRIRLKAYEPSILTQS
CEKILETASRTKGLTMGPVPLPTNRKIYCVLRSPHVDKTAREHFEIRIHKRIIDIYDPSQ
TIDSLLDLPSGVDISVMPTIQQLIRQERQQIKKKTKSPALKSCPQRRGVCTRVYTTTPK
KPNSALRKVARVRLTSGFEVTAYIPGIGHNIQEHSVVLIRGGRVKDLPGVRYHIVRGTL
DASGVKNRKQSRSKYGAKKPKMKKNKKTAPSGVAHIQSTFNNTIVTITTNVGDTVSW
ASSGAIGFKGAKKGTPFAAQIAAEKASKEAINQGMKEAEVFVSGPGAGRETAIRALQA
AGLEVTLIRDITAVPHNGCRPPKKRRVMARIAGVDLPKNKRVEIALTYIYGIGLSTSQSI
LSKANVSDVRCKDLEDSDVMTIREIIEEYPVEGAAKRVESLNIKRLMEVNCIRGRRHRA
GLPLRGQRTRTNARTRRGSKKTKKMALKERYGTVVSDKMDKTIVVSIENKITHKRYG
KIISKTKRYKVHDENNECKMGDYVIISETRPLSKTKRWQLKMAKKSMIEREKKRQKLVI
KHETKRIELKNAIKKSQSYEERLALYSKLQSLPKNSFPTRLRNRCWMTGRSRGYYRDF
GLSRHVLREMAQQCLLPGVKKASWMSRSLRKGPFVASHLAKKIDSLKATIIKTWSRSS
TILPNMVGHTIAVYNGKQHVPVYVSEQMVGHKLGEFSPTRTFRSHKSDKKAKRMNK
SAKKRIEINERNRLRNKAYKSTIKTIYKKCRVAIESSVEKLISLCHSKIDKAVQKGVLHSN
AGASKKATLDKYFKKMTQIFDESGFATPVTVIKAGPCIVTQIKNVEKEGYNAIQIGYAQ
EKKLTKPELGHFHKSNLPPLKHLKEYKVTSEDIKIADKVTVNIFSIGQLVSVSGKSIGKG
FAGTIKRYNFTRGPMTHGSKNHREPGSIGQGSTPGRVFPGKKMPGRLGGGQTMIKN
LKVIHINTENNLLVIKGTVPGKAGNLLSVRMAKSKGARVVITLEC---RSAE----
GVYRYTTTKNRRNNPNKLELKKYSPVSKKHEIFKEIKMTQIFDESGFATPVTVIKAGPC
IVTQIKNVEKEGYNAIQIGYAQEKKLTKPELGHFHKSNLPPLKHLKEYKVTSEDIKIAD
KVTVNIFSIGQLVSVSGKSIGKGFAGTIKRYNFTRGPMTHGSKNHREPGSIGQGSTPG
RVFPGKKMPGRLGGGQTMIKNLKVIHINTENNLLVIKGTVPGKAGNLLSVRMEKILT
FTIKLKGDTLKVKDLNTNNYVIQRAYLTQRINERQGTANTLTRAEVRGGGSKTWRQ
KGMGRARAGSNNSPLWKGGGVSFGPKPKLYLNKINRKEWKLGLRNLLIAKENNITVV
ENIITYKTKNIIKMLIDLAEDTLIILPMIQKELLQSTNNIKTIKLTLANNLNLKKILLAKN
LLIMKDSLKIIEDTYMIKLRLKKYGRKQQACYRIVVMPSTSKRDGRAIEELGFYNPQTN
ESNLNMERIKIRLSQGAQPTETVKNLLKRAKMLIKYPIITDKATRLLESNQYSFATDSK
AAKTDIKLAIEYLFQVKVASVNTYNTPKKTRRKANYKRAIVTLASGDSINLFSMEYLNP
NVPDIRVGDTVKLGVSITEGNKERIQFAEGVVIAKRNGGINTTITMRRVMQGVGVER
LYLVHSPKLKSIEILRRSIVRRSKLYYLRSRVGKATRLQQSF
>Cryptomonas_curvata_KY856939.1
MLRVYYPKITKITINRGLGDASKNNKALDTSVQELALISGQQPVITKAEKSIAVFKIRDG

MPVGIAVTLRKQLMYAFLERFIHLSLPRIRDFKGISVKGFDGRGNYNIGIREQLIFPEIEY
DKVDQIRGMDISITTTAKTQQEGIALLKALGMPFMVRVKRGNVARNRRNKILKIAKG
FRGTHSKLFRMANQQVMKALRYAYVGRKRRKREFRSLWIVRINAAARESNYSVLISAL
KKSEIALNRKMLAQIAVVDKDCFTKVIMAIRIYRPYTGGTRNRSVSDFSELTSTKPEKT
LLTKKISGGGRNNRGVITTRHKGGGHKQRYRIIDFKRNKLNIDGIVATIEYDPNRNARI
ALIHYKDGEKRYILHPKSLAVGSKISSGISAPLEIGNSLPLQSIPLGTDVHNVELIPGRGG
QIVRAAGTSAQIVAKDGRFVTLKLPSSEVRMVPRECYATIGQIGNIDYNNITSGKAGRS
RWLGIRPTVRGVVMNPCDHPHGGGEGRSPIGRARPVTPWGKPALGVKTRAKTKSYI
VRGMSRIGKLPIKVPDKVNVTISPTIIMIKGPKGELSRELPAAIKIIKTISVCTNSYQLYG
TYRSLINNMIVGVTTGFQKKLELQGVGYRSQLEGKNLILSVGYSHQVKILSPEGIKLSV
EGNTNVTVAGIDKELVGQIAANIRLVRPPEPYKGKGIRYQGEYVRKKVGKAGKMTLF
PTKDNCSWFLFDAESKTLGRLSTEISRVLLGKNDAQYNPSSSINHGVIVINVEKVDVKI
DDKFYYRHSGRPGETFREVQNRIPSRILEKAVKGMLPKGGRKLFTRLKVYKGPLHPH
QAQNPKQIMAKKVVAIVKLALDAGKATPAPPVGPALGQRGVNIVMFCKEYNAKTAD
KIGFIIPVEITVYDDRSYTFVLKTPPASVLLVKAAGIPKGSGDPQKKKVGSITRKQLEEIA
ETKLPDLNTKNKEAAMRIISGTAKNMGITIMLSPKRTKFRKPHRGRLGGVASKGNTIV
FGEYGLQALEPVWLTSRQIEATRRTITRYVKRTGKLWIRVFPDKSISSKPPETRMGSGK
GAPDYWVAVIKPGHVLFEMNGVPQALAYQALKNASYKLPIKTKFVSKMIQTQTYLNV
ADNSGAKKIMCIKVLGSNRRYASIGDVIIGVVKDASPNMPVKRSDVVRAVVMRTKKTI
RRSDGMSIRFDDNAVVIINKENNPRGTRVFGPIARELRDKDFSKIVSLAPEVLMYAIIEA
SGKQFWIEPGKFYDLNYIDLGDKISLVRVLLLNNVVVGHPCLETVEATVLGHIRSRKIT
VYKMRPKKKTRKKQGHRDNLTRVMIMVKAIAKYIRMAPNKVRRVLNQIRGKSYKEAL
MILEFMPYSACKPVLQVLQSAGANAENNNGLKKIDLEIVEACADAGTILKRFRPRAQG
RGYKIQKPTCHIRVKLMPKKDIHPKWYKDSKVYCDGQLVMTVGSTKPELNVDIWSG
NHPFYTGSQRLLDTEGRVERFLRKYKLMAHKKGSGSTRNGRDSNAKRLGVKRYGGE
YVLAGNILVRQNGTKFKLGNNVGLGRDYTIYSLIDGTVTFELSVYMKVVSSIKNRSKD
CQVVRRRGRLYVISTDPRLKVRQGMAVPKKRTSKSKKNSRLAKWTNKANLQAKKALS
LAKSILKSTSFISMIECVESKRDYGRFTLQPLDQGQGITIGNALRRVLLSDLEGTAIVAV
RIAGINHEFSTIPGVREDVLEILLNLKEVVLKSHIGRLRVQGPAIVTASDFDVTSEIEIID
PKQYIATICNNNILEMEFRIEGGKGYQIVFLQVDAIFMPVKKVNYSVEEIRDKLILEVW
TNGSISPEEAIGQGSTILTNLFNPLILIEELQLSVRAYNCLKRAQIHSVADLLDYSQEDL
LFGQKSAEEVIDALQKTNKIIDKKELKRLMAWAFSNYGTGRASYMADRLKELGFHYA
TRAGISLSVEDLRVPPTKENYF-
KTNEEIKFTEQRYDRGEITAVERFQKVIDTWNNSSESLKDEVIKYFKETDPLNPVYIMA
FSGARGNISQVKQLVGMRGLMSDPHGQIIDLPIKSNFREGLTVTEYVISCYGARKGLV
DTALRTADSGYLTRRLVDVAQDIIIREVDCGTTRGIVLLLGRVLFETLYIAHINQDLDS

ILAKQVSSIRSVLVRSPLTCESSRSVCQFCYGWSLAHGSLVDLGEAVGIIAAQSIGEPGT
QLTMRTFHTGGVFTELAEQIRAPFNGVLRFPQSIRARPTRTRHGEEALILEESIKVDLY
SKSKRSLNKGQFI-
VSDNEEFKDKQVIGEISARTKDLSTNISGEVYFSDLLWILAGDVYNIPTNAEINVRIVND
VLATTEIISDYGGTVRLFLLTGGFVIPEETHEINKDLLMVSNGERELIKDLFNSNSGLV
CIIVKEITIKPGYIFEKGQEVCRNFKASETSFIELIRPVDKYIIVNLLKTYLVLTSSDIIVKD
GDKIDAGAKVAETKLLCRSGGEVRNNKSVRNILLLTESHKKVISIPGKTAEGIIASDSG
QIIELNQEKITIRSGRPYLVSSGAILQVNHLDLIQTGDILAILVFERSKTGDIVQGLPRIE
EILEARKPKEPCRLCISLGEPLTPNPHEMLGIFFNFYEAAKLSLQKVQIYLVNEVQTVY
QSQNVDISDKHIEVIVRQMTSKVRVEDGGDTTLLPGELVELQQIENINEAMLLTTYSP
VLLGITKSSLNTDSFISAASFQETTRVLTEAAIEGKADWLRGLKENVIIGRLIPAGTGMS
TFILPDLVEIQRASFCWFLEEGLAEEIKSFSPITDTGNLELHFFGDQYKLKCPKYNVNES
KRRDSTYSVQVYVPARLKDTGVIKEQEVFVGDLPLMTDRGTFIINGAERVIVNQIVRS
PGIYYTYSGSLISNRGAWVKFETDRVWVRVDKTRKIPAHVFLKAMGLSDIYNGLRHPE
YLKKTELSRFFDPKRYDLGKVGRYKLNKKLNLNIPENIRVLTPQDTLAAIDYLVNLKFE
IDDIDHLGNRRVRSVGELLQNQVRIGLNRLERIIRERMTIKIIASIREFFGSSQLSQFMD
QTNPLAELTHKRRVSALGPGGLSRDRAGFAVRDIHPSHYGRICPIETPEGPNAGLIGV
LATHARINSYGFIETPFYKVNDGRAMLDAGVSYLTADQEDDFRIAPGDIAMDYLKKEI
IPIRYRQEFTTAFTTQIDYRAVSPIQVISIATSLIPFLEHDDANRALMGSNMQRQAVPLL
YPESPLVGTGLESQAARDSGMVTVSMSVGKVTYVSADKICVTYYLQKYQRSNQDTCI
NQRPLVWLGENVTSGQVLADGAATEGGELALGQNILVAYLPWEGYNYEDAFLVSER
LVYNDVYTSVHIEKYEIEARQTKLGSETREIPNVLRKLDDNGIIVIGSWVESGDILIGKV
TPKGESDQPPEGKLLRAIFVRDSSLRVPVLDVRIFTRVYVAQSRKIQVGDKMAGRHGN
KGIISKILPRQDMPYLPDGTPVDIVLNPLGVPSRMNVGQIFECLLGLAAENLNKRFKLF
FFDEMHGAEASRILVNNTLMEALTNKDWIFDNHHPGKMTIFDGRTGEKFDNPVTVG
ISYMLKLVHLVDDKIHARSTGPYSLVTQQPLGGKAQHGGQRLGEMEVWALEAFGAS
YTLQELLTVKSDDMQGRNETLNAIPRPGTPESFKVLMRELQSLGLDIGAYKIENLRGI
EVDLMMFDYVKINLASPERIKKWGERTLP-
NGTVGEVTKPETINYRTLKPEMDGLFCERIFGPVKDWECHCGKYKICERCGVEVAES
KVRRHRMGYVELAAPVTHVWYLKGSPSYISLLLDIPLKGAEAIQKLLKDIDLEVEAESL
REEVLSTGAKKDKAIKRLRVLDNFIATRSDPSWMVLTVLPIIPPDLRPMVQLDGGRFA
TSDLNDLYRRVINRNNRLRRLQEILAPEIIIRNEKRMLQESVDALIDNGRRGRVVVGAN
NRPLKSLSDIIEGKQGRFRQNLLGKRVDYSGRSVIVVGPQLQLNQCGLPREMALELFQ
PFVILIQQGAKMIQKNTLVWNVLEVIQGHPVLLNRAPTLHRLGIQAFEPLLVEGRAIK
LHPLVCPAFNADFDGDQMAVHIPLSLEAQAEARLLMLAPYNFLSPATGEPIIMPSQD
MVLGCYYLTADNPSQQTDYPRYFSNLDDCLMAYEQGVVPLHAFVWVRFLLVQYILT

TPGRIFLNKILQDSLMSRYRGAVLRIVRRLGLPGLTRKITKRNNRKPSEYSIRLEEKQKI
RFNYGLTEKQLLKYVRTARRIKGSTGEALLQLLEMRLDNTVFRLGMAPTIPAARQLV
NHGHICVNGRRVSIPSYQCETADVITVRSSTKSKQLVEGYLIPTHLEIDKSNLTGKVNG
IIEREWVALQLNELLVVEYYSRMGQKTHPLGFRLGITQTHRSSWFEPKESYPSMLEED
YKIRTYIEKTISKIEIHRKDQIELEIHTSRPGVIVDDLTKNLRQIRINVTELAKADSDASLI
AEFIAQQLEKRVAFRRATRQAIQKARQGIKVQVSGRLNGAEIARSEWVREGRVPLQTL
RADIDYATKRAQTTYGVLGVKVWVFNMVNDTISDMLTRLRNANLAKHQIVEVPSTK
MTRNIANILLEEGFIQSFEEVGNTQLLLSLKYKGRHREPVITALRRVSKPGLRVYANRK
ELPRVLGGLGIAVISTSQGVMTDNKARHQGLGGEVLCYIWMWQERIVQVRRVTKVV
KGGKKLSFVLFLIIGNEKGQVGVGVGKASDVIGAVKKAVTDGKKTSYRRPITKDNSIP
HIITGRSGAAKVIIRPSAPGSGVIAGGAVRTILELVGVKNILAKQLGSNNPLNNARAAT
NALLQLMSRRTTAKKRLALPDPIYNSRLVSMLTVRILQEGKKHLAQRIIYQALDIIKEK
TGEEPLAVLETAIRKVTPLVEVKAKRIGGSTYQVPMEVRAFRGTNLALRWITKFSRERS
GKTMAMKLANELMDSANETGNSIRKREETHRMAEANKAFAHYRRMIRIKLKSYNSSL
LNLSCEKIIETASRTESLTMGPIPIPTKRKIYCVLRSPHVNKDSREHFETRTHKRIIDVYD
PSKTIDSLLDLPSGVHIEVMPTIQQLVRSEREQIKKK----
SLKSCPQRRGVCTRVYTTTPKKPNSALRKVARVRLTSGFEVTAYIPGIGHNIQEHSVVL
IRGGRVKDLPGVRYHIIRGTLDASGVKNRKQSRSKYGAKKPKMKKNKKAAPIGVAHIQ
STFNNTIVTITTTTGETISWASAGAIGFKGAKKGTPFAAQIAAEKATKEALVQGVKKAE
VLVCGPGAGRETAIRALQAAGLEITLIRDITPVPHNGCRPPKKRRIMARIAGVDLPRNK
RIEIGLTYIYGIGLSSSQNILTKAQVNDIRCKDLTDQDISIIREIIKEYQVEGAARRIESVNI
KRLIEINCVRGRRHRLGLPLRGQRTRTNARTRRGSKKTKKMPIKERSGVVISNKMEKTI
VVSIENRITHKKIWKVIAKTKKYKAHDEKNECQVGDVVTITETRPLSKTKRWNLKMA
KKNMIEREKKRQCLVIKYGKKRTEIKESIKASTSFQEKLAFYGKFQTLPRNSFPSRLRNR
CWMTGRSRGYYRSFGISRHVLREMAHSCLLPGVTKSSWMSRSLSKGPFISSNLSKKIDL
LKKDIIKTWSRASTILPSMVGHTIAVYNGKQHVPVYINDQMVGHKLGEFSPTRNFRSH
KSDKKAKRMTKSAYKRIEIAERNRLRNKAYKSTVKTLIKKTLVSISSSVKKLMSLTYSKI
DKAVQKGVLHSNNGDSKISFGQKFLKNMTQIFDDSGLAIPVTIIKAGPCTVTQIKTVE
TDGYNSVQLGYLESKLLNKPELGHLQKTGSSPLKHLKEYRVDSPNTAIGSIISISSFSIG
DKIAVTGKTIGKGFAGTVKRHNFTRGPMTHGSKNHREPGSIGMGTTPGRVYPGKRM
AGRLGGKQTTIKNLQVILINTENNLLVVKGAIPGKLGNLLSIKMAKSKGVRIVITLEC--
-KTSA----
GVYRYTTTKNRRNTPNRLELKKYCPLSQTHEIFKEIKMTQIFDDSGLAIPVTIIKAGPC
TVTQIKTVETDGYNSVQLGYLESKLLNKPELGHLQKTGSSPLKHLKEYRVDSPNTAIG
SIISISSFSIGDKIAVTGKTIGKGFAGTVKRHNFTRGPMTHGSKNHREPGSIGMGTTPG
RVYPGKRMAGRLGGKQTTIKNLQVILINTENNLLVVKGAIPGKLGNLLSIKMVKQICC

DVRQPTITLNLKTCEVSKFFFHRILTIQNKNQRCKTACSKTRAEVRGGGRKPWKQKG
TGRARAGSSVSPLWKGGGVVFGPKPTVYNKKINFKERQLAIFTGLYACSYKIVIVRNFV
SPRTKAFLGLLSLLSKRILILVPKATETLYSSCRNVEKITIGSRKTLTLRDIFLADSILIAEE
ALPEFLLV-
MIKLRLKKYGRKGQISYRIVVMTSSTKRDGRAIEELGFYNPRTKEVNINLSRIKIRLNQG
AQATPTVENLLKK--
MLIKYPIITDKATKLLELNQYTFAVDPKSNKLHIKKAIEYLFNVKVISVNTANPPAKQRR
KTQYKKAILTLATGNSINLFSMKFLNRNLPLIRVGDTLKLGVSITEGNKERVQFTEGV
VIAKKSSGLDATITLRRVMQGVGVERVYLINSPKLKSIEILRSSTVRRSKLYYLRLRFGKA
TRLQQRF
>Cryptomonas_paramecium_GQ358203.1
MLRNYYPKLIKITLNRGLGEASKNNKSLDTSVHEFELITGQHPVITKAKKSVAGFKIRE
GMPVGIMVTLRKELMFSFLQRLIHLALPRIRDFKGVSITGFDGHGNYNFGIKEQLIFPE
IDYDKVDHTRGLNISIVTTASTQQEGAMLLKCLGMPFMTRTKRGNIARKRRKSILTAA
KGFRGSHSKLFRIANQQVMKSLQYSYIGRKRRKREFRSLWITRINAAVRNLTYSSFISEL
KNHNIGLNRKMLCQMAIKDSEGFNKLI--

MSRISKLEVAIPDKIDFSVNENKIKIKGPKGELSRQIPAGVTIARSLQLVKNSVELCGTYR
GLIINMIVGVSSGFKKKLELQGVGYKAHLDNNTLILSVGYSHQVFLKLPLGIQIAIENN
TNISVLGIDKELVGHMAATIRLVKPPEPYKGKGIRYQGEHIRKKIGKAGKMTIFPKQAH
PQWFIIDAQEQTLGRISTKIAQTLLGKNNILYTPGYCAQQRVIVVNAKMVKVKKLHKL
YCRHTGRPGKTFEEITHLSPFYTIEQSVKRMLPKNGRKLFTKLEIYKDSHHPHTHKKL
EKLMAKKITAIVKLALNAGKATPAPPIGPALGQRGVNIVAFCKEYNAKTSEKVGLIIPV
EITIYEDKSYTFILKTPPASVLLTKAAGIQKGSGEPKKKKVGFVTKKQLEEIAEEKMPDL
NAKSKESAIKTISGTAKNMGITIMLYPKKVKFRKPHRGRLGGTIVSENGVIFGDFGLQS
LGPVWLTSRQIEATRRTIARYVKKTGRLWIRIFPDKSISSKPPETRMGSGKGSPDYWVA
VVKPGQVLFEINGVSQVMAYEALKNASYKLPIKTKFISKMIQTQTLLNVADNSGPKKV
MCIKVLGGNKKHASLGDIIIGVVKDASPNMPIKRSDIVRAVVVRVKKSVRREDGMHISF
DDNAVVIINKENNPRGTRVFGPIARELREANFSKIISLAQEVVMYAVVSAGGGQLWVE
PERVYDIQPVPFGDRVLLPRVLLMNKFFLGGPYTLVIKAVILGHAEAKKVKILKMQSK
KKTRKRQGYRAPFSRILVMSKAVAKYIRVAPNKIRRVVNQIRGKSYSEALIILEFMPYSA
CKPVLSLLHSAGSNAENNDGVKKSVLYVSEVYVDSGPSLKRLRPRAQGRAYTVRKRFS
HISLQLMPKKNIHPKWHKESKVYCNGAFIMTTSSTQPKITVDIWSGNHPFYTKSQRLI
DTEGRVEKFLKKYKFMAHKKGGGSTKNGRDSQPKRLGVKVFGGEVVSAGCILIRQRG

TVFKLGRNVGLGRDFTVYSLVNGRVVFEVSVHMKVVSSIKHRSKDCQIVRRRGRLYVI
SSDPRLKVRQG--
MIKYIESETSYGRFTFQPLKLGQGLTIGNALRRVLLSDLEGIAITSARVSGSVHEFSEIPG
TREDVLEILLNLKKIVLKGSIARLKVQGPCIVTASDFELPSFVKAVDPSQYIATICDSNV
LEIEVSISAGRGYKLVSLELDAVFMPVNKVNYTIEEIEEKLTLEIWTNGSLTPEESLMQA
ATVLTDILAPILPIEDLKLSVRAYNCLKQINIDYTEDLLTRSQEELLFGKKCCEEVTQAL
KNTNKVVDKKKLKQLMSWMFTTYKAGVTSHIVDKVKELGFQYATEAGISISIEDLKVP
PKKQYIVKSATQQNSIIEKKYTRGETTIIERLQNTITSWGRASESIKNEVIKYFEVNEPLN
PVYMMASSGARGNMSQVRQLVGMRGLMSDPHGQIIGLPIKSNFKEGLTATEYIISCY
GARKGVVDTALKTANSGYLTRRLVDVVQDIVVREKNCNSTHGVLIMRGRCLFDTLCI
ARQNECLNNLLTQNISKINSISIRSPLTCISSQFVCQLCYGWNLTTESLVDLGEAVGVL
AAQSIGEPGTQLTMRTFHTGGVFTEFAEQINAPFNGIIEIPNHAKTNPIKTRHGNDAL
LVEKNTHIRLWGNTTLEIKPGSLLFLTNNEQFKKGQILAEQPLKTKELINETIGEVFLS
NSLWILYGDVYSIPTHAKIIATEKGDALAVYETKSEQSGIIKLFLLTGGLIIPEETHIINKP
LLIATNQSQEIVKGIKSCHDGFLSIYLKEVSVKPGELFFPGEEIVPGLIAKDTCYVELLRT
AIIYTVITLVSTYITVVKNKLHVHNGQRITPGITIAESKLLCEKDGIITLNQLENKLLVLT
KKHNETISIGYNTANGVFSPCNGYIMSIKNNLITIRHGTPYLTSCNSTIYVDHLGVVDK
NNILAKFTLEQPKTGDIIQGLPRIEEILEARKPKEICQLLVNVASKLTPNLHEVLSIFFTY
YSATRLALLKTRRYLVNEVQNVYQSQGVHISDKHIEIIAKRMTSKVQVNSSGDTTLLP
NELIELEQFEEANGAILESTYSPVLLGITKTSLNTNSFLSAAGFQETTRVLSRAAIEGRK
DSLTGLKENVSTGRLIPAGTGMYHLRLPNLTELQRSSFCWLLQEGLAEVIRNFSPISNE
ENLEIHILSDKYKLKYPKNNPSEAIRKNSTYCTEIHIPVIIKEKKVTKEQEICFGELPLMT
ERGTFIINGTERVIINQIIRSPGIYYTYSGNLISNRGAWIKFELDKIWARIDKSRRVLIHIFL
KALGLTDIYHGVKNPNLLKKTKISRFFDPKRYDLGKVGRYKLNKKLQLSVPRSITVLT
PQDILACIDYLINLKLEPDNIDHLSNRRVRSVGELLQNQIRIGLQRLEKFTREQMISKIQ
ASIREFFGSSQLSQFMDQTNPLAEITHKRRISALGLGGLHRDRIGSSVRDIHPSHYGRIC
PIETPEGQNAGLIGVLSIYARVNTYGFLETPFNKIVNSREIQNNSPTYLTADQEDLLYIA
PADTITNQIQKHTLTAKHQQEFIQILTTKTNWKPVSPIQTISIATSLIPFLEHNDANRSL
MGSNMQRQAVPLLFPEKPLVGTGFEAQIARDSKTVVTSLTKGNVTYVSSKKVCIEYH
LQKYERTNQDTCINQKPLVWYGDSIIKGQVIADGLATEGGELALGQNVLVAYMPWE
GYNYEDAILVSDRLIYDNVYTSVHIEKYEIEARRTKLGHETKKVPNTLRNLDKNGIIAR
GSWVEGGDVLVGKITPKKDAEQPLLSKLMKAILVKDTSLRMPILDVRVLTRIYVAQTR
KLKVGDKMAGRHGNKGIVSKILPRQDMPYLPDGTPIDIVLNPLGVPSRMNVGQIFEC
LLGLAAEHLGKRFRILPFDEMQGREASRVLINNSLLDAKTGKNWLFSLDHPGKMILY
DGRTGEPFKNPITVGVSYILKLAHLVDDKMHARSTGPYSLVTQQPLGGKAQHGGQR
LGEMEVWALEAYGASYTLQEFLTIKSDDMEGRNEALRAMPKPRTPESFKVLLKELLSL

GLDVTAHKTNQQKGIKNTKTMFNCLNIKLASPERIKKWAERLLP-
TGIIGEVTAPETISHRTLRPRTNGLFCEKIFGPIKSWECKCGKQKVCKQCGVELIESRV
RRHRMGYINLAAPVTHVWYLKGSTSYISTVLDFSEKGAEAIKELLEKINLELEVESLRTI
LPSTTTPRNKIIKRLRILSNFVATQSKPSWMILTVLPVIPPDLRPMVQLDGGRFAASDL
NDLYLRIINRNIRLQKLGKTTAPEVIIMNEKRMLQESVDALIANGKRGKAVVGANNRP
LKSLSEVIEGKQGRFRQNLLGKRVDYSGRSVIIIGPKLKLNQCGLPKEMALELFRPFVIL
IVQGAKLLQEKMAIWDVLGIIYNHPILLNRAPTLHRLGIQAFEPTLIEGRAIKLHPLVCP
AFNADFDGDQMAVHVPLTTEAQTEARVLMMAPLNFLSPATGEPIMTPGQDVVLGC
YYLTSEDTSGNYPPTHYFSCQDDCLKAYEQKILRLHSQIWVRMIETQYVLTTPGRILL
HKTICDSIMSRYRGAALRIVRRFGLPGLTRKTTNRKHRRLSEYAIRLDEKQKIKFNYGL
SERQLFNYVKKARRIQGSTGQALLQLLEMRLDNTVFRLGMAPTIPAARQLVTHGHIY
LNTKRVSIPSYQCSAGDIIKIKDSPKSKKLVTDYIAPTHLELDKTTFTGVVKGIVEREWV
GINLNELLVVEHYSRMGQKVHPLGFRLGVVYRHRSSWVADKGNYPSYLKEDFLLRKY
FSSRISTIEIHRRTYIDLYLYASRPNALMKEIKEYLRNLRVHVLALLHPDLDAYLLATFIS
QQLEKRISFRKVMQQAIRRAKKGIKVQISGRLGGAEISRSEWKREGRVPLQTLYADINY
AAKEAHTNYGVLGVKVWIFRMSNDTISDMLTRIRNANLAKRSVAQVPSTKMTRNIASI
LLEEKFISRVEEIKTSWLLLSLIYGKIGKSPILSIIKRVSKPGLRVYANRKELPKVLGGIGIA
IVSTSKGVMSDHKARHLGLGGEILCYICMWQEKILQVNRVTKVVKGGKKLSFRVVMII
GNRKGSVGVGLGKANDVASAVKKAGADAKKHILTIPISRSDSIPHMTMGKAGAASVII
RPSSPGSGVTAGGSLGMILRLAGVRNVLAKRLGSSNLLNNAHATVDALSKVMSRHVP
KKRELPSPDPVYNSRLISMLSTKIIIGGKKRLAQRIIYDALNLIGEKTGQEPLQIFEDAVK
KVTPLIEVKAKRVGGSTCQVPIEVRAFRGTYLALKWITKFSRERPGKKMATKLANEIID
SSNELGNSIKRKEETHKMAEANKAFAHYNRMLKLILKSYEISALKKSCEQIINVSYRTGC
SSTGPISLPTKRRIYCVLRSPHVNKDSREHFETRTHKKVIYLHNPPETIQSLLDLPPSVH
LKIMPTIQQLISLERLHVRKKTKSSALKGCPQRRGVCTRVYTTTPKKPNSALRKVARV
KLTSGFEVTAYIPGIGHNIQEHSVVLIRGGRTKDLPGVRYHIIRGTLDASGVKNRKQSR
SKYGSKKPKMKRDKKVHPKGVAHVQSTFNNTIITITNLLGDTIAWSSAGSVGFKGAKK
STPFGAQVAAENAAKSVISKGLKEIEVFVDGPGAGREAAIRALKTTGLEIKLIHDVTST
PHNGCRPPKKRRIMTRIAGVDLPKNKRVEIALTYMYGIGLSTSQLILAKSNVDNVRCR
DLTDHDVSSIKETIKHYLVEGAIKRIESISIKRLIEINCTRGKRHTLNLPVRGQRTRTNA
RTRKGVKKTKKMPIKERTGVVVSNKMDKTAVVSVVSRGTHGKYGKIVCKTKLYKVH
DETNICSIGDLVTINESKPLSKTKRWILAMARKSILEREKKRRHLVAIYSNKREKIKEQIK
KHTLVTEKIKLNAEFQLIPRNAFPSRLKNRCWVTGRGRGYYRDFGLARHSLREMAHA
GLLPGVTKSSWMTRSLSKGPFINSRLIGRIERLKKVILKTWSRACTIIPTMVGHTVAVY
NGKQHVPVFIRDQMIGHKLGEFSPTRVFRSHKGDKKVKKMLKSSCKRALLSEKRRLR
NKNYLSTVSTLMKKTLLAMLKTISNLISITYSKIDKAVQKGIIQLNKGNSKKSKLAIELK

NMTNVFDPSGVAIPGTIVKVGPCIVTQVKTVPKEGYSSIQLGYFSKASLSKPELGHLKI
SNSQAVRYLREFRVDSDENLLGQEVTVSKFSVGDRVSVAGKSVGKGFSGTVKRHHFT
RGPMTHGSKNHREPGSIGMGTTPGRVFPGKRMAGRLGGKKVTIKNLRVVFVSLENN
FLVLKGSVPGKKGSLLRILMAKNKSLRIVVTLES---KGYE----
GVFRYTTTKNKKNTPNRLELKKYCPLTKKHELFKEVKMTNVFDPSGVAIPGTIVKVG
PCIVTQVKTVPKEGYSSIQLGYFSKASLSKPELGHLKISNSQAVRYLREFRVDSDENLLG
QEVTVSKFSVGDRVSVAGKSVGKGFSGTVKRHHFTRGPMTHGSKNHREPGSIGMGT
TPGRVFPGKRMAGRLGGKKVTIKNLRVVFVSLENNFLVLKGSVPGKKGSLLRILMQKI
IEYNSLSTGQIFKLNILDSGNYLIHRDILRHQISQKQGTVSTKTRSEVRGGGKKPWKQK
GTGRARAGSSRSPLWRGGGVTFGPKPKRTTLKLNKKERNLALQTLFYNKRNNISVIG
ELINPKTKVFCDICLDLNQKILLVVAKKTPYLKLSTQNLKNVELISASNLNTLSLLKAK
QILLTPLAVNDIKEIYMLKLRLKKCGRKRQPSYRLVVMRSTTKRDGKAIEELGFYNPRT
KELSVNLERAIVRIAQGSKPTSVVQNLLLKIGMFIKYPILSDKSTELLKLRQYCFLVDKR
LHKLEAKRAVEFLFAVRVSSVNSCNRSPKCRQKPSYKKIIVTLAPGNSISLFAMLFLVPA
LVKVRVGDTVKILVSWSEGPKEKSHSVEGLVVAIKHSGLDKTVTLRRIIHGVGVDRVY
FLSSPRLKSVEVVATSKVRRSKLYYLRSKFGKDARLPQKF
>Cylindrotheca_closterium_KC509522.1
MIKQEYPKLKKIIINRGLGLAAQNTNILKKNIEEFEKITGQKPMITRAKKAIAGFKIREN
MELGLSVTLRGEKMYAFLTKLLFFTFAQIRDFRGLSLRSFDKAGNYTFGLKEQLIFPEV
DYDEVDQTQGVTINIVLEHSSPKNGMILFKFLRFPLMVRIKRGNVARKRRKKILAMAS
GYRGAHSVLFRVANQQVMKALRYSYVGRKQKKRIFRRIWISRINAASRFITYSQLINKFK
KSNIDLNRKILSQIAILDTSTFKSLIMSIRLYKSYTPGTRNRALSGFNEITKSKPEKSLIKK
NHRNKGRNNRGVITIRHRGGGHKRRYRIIDFKRNKYGVEGIVASIQYDPNRNARIALIH
YKDGEKRYILHPKNLNVGDIILSGSDSPLTIGNTLPLAEIPLGSSIHNIELIPNKGGQLVR
AAGTSAKVLAKEGNYVTLRLPSKEIRLIRQECLATIGEISNNDAFLIQSGKAGRTRWLG
KRPTVRGSVMNPCDHPHGGGEGRAPIGRTRPLTPWGKPALGMKTRKRKQSYILRRM
SRIGKLPITIPNSVEVNYNDAKIIIKGKFGTLETKIPETILIDNILSVSRSLRSLHGLYRTLI
NNMVIGVSEQFKLTLILKGVGYRAIVQGKEIVLNLGYSHPVNIEIPELISVEVVQNTIINL
ESCDKELLGLFAANIRAWRRPEPYKGKGILYKNEQILRKAGKAGKLTFLPTQTNRNWF
LIDCKGQSLGRLATLITNLLKGKIKPQYHPAVDTGDYIILINAELIVIEDSKHYLVYNPG
RPGRSLKNVSDCLPELTIKRAVKRMLSKTTKRLMKRLKIYNDQNHLHQAQNPIQIMA
KKITALIKLALPAAKATPAPPVGPALGQHGVNIAAFCKEYNAKTNDKSGLVIPVEISVY
EDRSYTFILKTPPASVLLVNAAKIKKGSSTPNRVSVGSITKAQLEEIANVKLPDLNTTKL
TSAMKIVEGTARNMGISIMLSPKRTKYRKYHRGRMRGKATRGNQICFGNFALQALEP
NWITSRQIEAARRTITRYTKRGAKLWIRIFPDKTVTARAAESRMGSGKGAVDYWVAVV
KPGTIIFEISSVPEEIAKSALSLASYKLPLKTKFINKMIYPQTILTVADNTGAKKVMCIRIL

GGNKKYAKIGDTIIAVVKESIPNMPIKRSDIVKAIVVRTKKVIRRQDGMYIRFDDNAVVI
VNTDNNPRGTRVFGPVAREIRDKNFSKIVSLAPEVLMYAIIEISGRQFWIETGKYYDFN
RIPTGKQVTLNRVLLVNNVLIGKPYLEIIKAKILEHLRGKKTIIYKMRPKKKTRKKAGH
RQELTRVFIMVKAVAKYIRMSPHKIRRVIDQIRGRSYQEALMILEFLPYDAGGPIWQVI
HSGAANAKHNYGLDKKKLIIDQIFANEGPKLKRIRPRAQGRAYKILKPTCHITVVMMP
KSNLHPKWFNDTIVLCDGKALCITSSTKKELQVDMWLANHPFYSKSQLIIDSEGRVER
FMKKYGLMAHKKGAGSTKNGRDSNAKRLGVKKFGGERVRAGNILIRQRGMKFKPGA
NVGCGKDFTLFALMNGTIKFDVNVIMKVRPSVKKMCDKCRVIKRHGRIMVICQNPK
HKQRQG---
MITCLKSEQEHGQFLIHSLKPGQGITIGNQLRRVLLNDLGGVAISAVRIAGVSHEFSILP
GVREDILEILLNLKGIILKSKFGRLKIQGPAVITADLIQLPSNLEIVNPNHYIMTISTSNIL
EIEFKFEYGTGYKLAYLQLDTIFMPVEKVDFKIENISERIFLDIWTNGSISPNEALKSAAQ
LTIDLFSFLIAIEELQLSVRAYNCLKKAQINTVGDLLHYSPLQELFGRKSADEVFSTLKN
QNDLISKKQLRQLLAWSFTHYDSMQACSLADELKYLGFKYATQAGISISIEDLKVPFV
KNLMLNKSNHEIQNAEKIYLKGRITQSERFQKIIDTWSLTSESLKDQIIYYFKNYDPLNS
VYIMAFSGARGNLSQVRQLVGMRGLMSDPSGQIMNLPIKKNFREGLTITDYLMSGYG
ARKGIVDTALKTANSGYLTRRLIDVAQDILIREKDCATTHSFLFVVGRILSHDIFVSNK
NTQITPQLIKKFHKIKQFYVRSPLTCSLYRAVCQKCYGWDLASENLVDIGEAIGILAGQ
SIGEPGTQLTMRTFHTGGIFTEARQQIISPLNAVIQFYNILKTVVLRTNRGEDVLVTKN
SGSIILINLVSIEVLRNTILFPKNNQYVTKDTVIGELINQNKQILSNTSGEIFINNLIWILS
GQLYNSPNNFYPDSKLNNSYIFRTKIINHYRGSVNTHILNGGITLGEETYQVNCEILFV
GDFIFELLPGLYSKTSGLVTLIIKTISIKSGKVYYPGEIILEHLSVKELSFCELVRPIQIYEFL
TLISTFLKFIKSCFLIQPNQFLNNYSILGYLESVTNSLEIVKFKTRIKQIFLISNKDCLSLKK
KQNKKGFINVAKTGKIIIENDEVVTIQLGRPYFFQEVNSVFCRNAEFIEAGKTIGLLNLE
KEITGDIVQGLPRIEEILEARKKNISQKKIQKLATTLSINPHKLLKVYFNYYEGTYKTFK
KVQLFILNSVQSVYQSQGVTINDKHLEVIVKQLTTKVLITYEGDTPLSRREVIDLYHIQ
YINQIIKSEYYIPLLLGITKAALNNPSFISAASFQETTRVLTRSAIEGRIDWLRGLKENIIIG
HLIPAGTGMYINALPDFLAMQRVSFCWFITQGLNEELTLFSKIYDFKNTEYQMFGDEY
SLIKPPYSLSIARKYSGNYRAQLVIPIEVRSKIICYQAQFPVVTLPLMTTYATFIINGCERV
VVSQIIRSPGIYFLYTATLIPDYGSWIRFGFQKVTIQLDKINHKPVLGLLKEMGLTEIYQ
NLHYSDFFYFSEFSRIFDPNYYRLGRVGRLKLNTRLNLNITEHLQVITYQDLFAIIDKLI
NLTISKDDIDHLKNRRVRSVGELLQNLFRIGFQRLVRKLRNQTNKFIVATIREFFGSSQ
LSQYLDQTNPLSSLTHRRRISGLGPGGFDRDRISFAVRDIHPSHYGRICPIETPEGQNV
GLIASLTTCARVNKLGFLETPFWRVINGKVIKTGNPIYLTADIEDLYKIAPADIATNYLK
KHLIPVRYKQDFINISPSEVDFIAISTVQVVSVAASLIPFFEHDDANRALMGSNMQRQS
VPLIFPQKPIVGTGLENQIAIDSGMTLNAQISGIVNAVTANKIIIQYKLQKYLRSNQQTC

INHRPIVWKGEKIQSGQILTDGPAITNSELSLGQNVLIAYMPWHGYNFEDAILISERLIY
DDVFTSIHIERYKIEIDRNSEMSEMKMIPNLLNHLNDDGIVMVGTFVKPGDILVGKVIS
NNTSEQLPESKLLRAIFVKDNSYRMPVIETVTFNRIFIAQIRKIQVGDKIAGRHGNKGII
SRILPRQDMPFLPDGTPIDIILNPLGVPSRMNVGQLYECLLGFAGDKLNSRFKILPFDE
MYGLEMSRILINKKLRQASRNESWLFNPYTPGKMLLIDGRTGKEFENPITVGNAYMLK
LIHLVDDKMHARATGPYSLITQQPLRGKAQHGGQRFGEMEVWALEGFGAAFTLKEL
LTIKSDDMQGRNETLNAIPQFGIPESFKVLLQELRSIGLDMSTYKIERFSEIEVNLIMFN
YIKIKLASPFRVLQWANRKLP-
NGFVGEVQKSETINYRTFKPEMDGLFCERIFGPSKSLECACGKYKICERCGVELTESRV
RRHRMGYINLIYPVTHVWYINSRPNFIALLLIKRIKGAPLIKRELEKLKLKSEIFRTRSFVV
ICTKIREQAIKRIRILENLIGTGSRPSWMILSILPVIPPALRPMIQLEGGRFATSDLNELYR
RIITRNNRLLRLLEIDAPQLIIRNEKRLLQEAVDTLIDNGKRGKIALSANNRPLKSLSDII
KGKHGRFRQNLLGKRVDYSGRSVIVVGPSLKLNQCGLPYEMAIELFQPFIILINQGAKL
IQQNLVVEPVLKILANHPIFLNRAPTLHRLGIQAFEPVLVMGRAIKLHPLVCSAFNADF
DGDQMAVHIPLSLNAQAECYMLMLAPYNFLSPASGEPIIMPSQDMVLGCYYLTVNNI
KGLLGSNHYFANLNDVILAYEQNRIEIHSSIWVRYIIVQYVKTTTGRVLLNYTIQETLM
SRYRGPKLRISRRLGLPGLTTKKSKKLVGKKSEYAVRLEEKQKLKYNYGLTENQLFRYV
KEARRRQGVTGLILLQLLEMRLDTLCFTLGFAKTIRQARQLVNHGHITINGKVLNIPS
FQCRLNDVIGVKDRTVSKNLISNNLISYNLKFDPGELKATVLDYCDRNAILLPLDELLV
IEYYSRMGQKTHPLGFRLGITQEHKSTWYANLNQYANVLEEDDKIRTYINTIISNIQIC
RNDQIQLNIKTGKPILVILDIKKLLRQITIKVFEIENVDLDAKLLSELIAEQLEKRVAFKR
AMREALQRSKNGIKIQVSGRLNGAEMARSEWIREGRVPLQTLRADIDYATTEANTIYG
VLGIKIWLFKMVTDTIADMLTRIRNANMVKHQIVQIPVTKISIAIAAILREEGFIEDFNN
YNKQYLLLSLKYIGKSRKPVISKIRRISKPGLRVYSKSNNLPKVLDNLGIAVMSTSKGIM
TNLKAKELGIGGEVLCYIWMLVERLIKISRVSKVTKGGKKLSFRAVVVVGDENGKVGV
GVAKAADVVNAFKKAKTDGRKNLIELPLTKALSIPHNVSGRFGACKIILRPSIEGSGVIA
GGAVRIVLEVAGVKNVIAKQLGSNNLLNNARASINALQNLMSRRNISKKRLPQPDSIY
NSYLVSLLITRILKSGKKTIAKKIVYEAFEIIKQKTNEDPLAIFEKAICKGSPVVEVKARRV
GGSTYQVPIEVSGFRATNLSVRWIIQYSKQRVGRSMSIKLANEIIDTANEIGNTIKKREE
THKMAEANKAFAHFRRMIRVRLESFNAELLVSSCQKIVETLGNTSLNSIGMITLPTAKR
IYCVLRSPHVDKDSREHFEIRVHKRILEIYYEPPIFDLLADLAPGIFYRIMPTIQQLVRSR
RIQIKKKTKSPALLNCPQRRGVCTRVYTETPKKPNSALRKVARVRLTSGFEVTAYIPGI
GHNLQEHSVVLIRGGRVKDLPGVRYHIIRGTLDTGGVKDRTQGRSKYGVKKPKMKK
NRNSFTTGVVHIQSTFNNTIVTITNLMGDTISWASAGSSGFKGARKSTPFAAQTAAEK
AALDALSTGMKNVEILVKGQGSGRETAIRAIEGAGFEITSIQDITAVPHNGCRPPKRRR
VMVRLVGVDLPRNKKIAYALTYIHGIGLTSAKEIIAQANITETRVYDLTTEQTVGLRQT

LEDLKLEGDLRRFNGLNIKRLNEINCHRGKRHRNSLPVRGQRTRTNARSRRGAKKTK
KMPVKQQIGIVISNKMDKTIVVKVENRYSHPIYSKTLVKTNKYLAHDESEQSNIGDQV
LLHECRPLSKRKCWKLVMAKKSMIEREKKRIRLEKKYATKRITLLKSYQNEQDFTKKL
ELHSKIQKLPRNSSKTRIRNRCWKTGRPRGVFRDFGTSRHIFREMAHQCLLPGVTKSS
WMTRSLKKSPFVAYHLLKKINKMKKDIITTWSRSSTILPNMVGLTIAVHNGKQHVPIFI
SDQFVGHKLGEFVSTRNFKTHKTDRKGKRMTKSSKKRIDINKRNRLQNKYYKTSVRT
LIKLFFKNLEIYVHEILSLIYCMMDKGIKKNIFHKNMVARKKSKLACYLKKMTQIFDES
GNIIPVTILKVGPCVITQIKTIAKDGYDGIQIGYKNSKSLNQPQLGHLQKSNIQPLKYLK
EFRVVKHDFEIGQVLTVDKLSLGQLVNIQGKTIGKGFAGLQKRHNFTRGPMTHGSK
NHRAPGSIGMGTTPGRVLPGKKMAGQLGNKITTVKKLKVIQFNLQENILVVKGSVPG
KPGNLLSIVMAKNKGTRILITVECTECRSNNKRSSGVSRYHTQKNRRNNPERLELKKY
CPNCNRATIHKEIKMTQIFDESGNIIPVTILKVGPCVITQIKTIAKDGYDGIQIGYKNSK
SLNQPQLGHLQKSNIQPLKYLKEFRVVKHDFEIGQVLTVDKLSLGQLVNIQGKTIGK
GFAGLQKRHNFTRGPMTHGSKNHRAPGSIGMGTTPGRVLPGKKMAGQLGNKITTV
KKLKVIQFNLQENILVVKGSVPGKPGNLLSIVMSVLLTFPVNFKTKELKTKNLTKINYV
IHRAYITQRYNQRHGSANTLTRAEVRGGGRKPWRQKGTGRARAGSNRSPLWRGGGV
SFGPKSKVYLNKINRKEWRLALRSLLVQKHKDITILEDIYQNKTNTFIQNFIKFNQKTLI
IIDSEIKALQKITKNIKTIKVLPANRLNIKEVIYSKQLFITKDSLKLIQETYMLKLRLKRIG
RKRSPSYRLVIMENTSRRDGRPIEEVGYYNPISKNYKFDQEKIIKWLQYGVKPTETVSN
LLKKIIMIIKYPIITDKATRLLENNQYSFIVDRSSNKPVIKATIEYLFNVKVIRINTCHLPR
KQKRKPQYKKAIVRLAEGDVINLFTMNIEKDDLPILRIGDNVKLGIKIIEGNKERTQFY
EGTIIAKKNTSINTTITVRKTLQGIGVERIFLIHSPKVESIDVLRSSKVRRSKLYYLRSLKG
KASRLKQAF
>Dictyopteris_divaricata_KY433579
MLKNLYPKLVKIQINRGLGLEGQNNKLLQKSVDEIRLITGQQPVLTKAKKSIAGFKVR
EHMTLGITVTLRQTKMYTFLEKLIHLVLPRIRDFRGLSTKNFDRNGNYHFGLQEQLV
FPEISYENVDQIRGLNISIVTTAKTQAEGIALLKEFGFPLMVRVKRGNIARKRRNKILKR
AKGFFGAHSNSFRIANQQVLKSLRYEYVGRKTKKRVFRQLWIIRINAAVKKLCYSQFIF
KLKQASILLNRKMLSQLAILDPKAFSSLIMAIRFCKVYTPGTRNTILSSFKEITKPSSEKK
LTKKNHRKKGRNNQGLITSRHHGGGHKKLYRLIDFKRRKHNIVGFVASIEYDPNRNA
RIALINYRDGQKSYIICPEEVRIGSRIMSGPTAPLEIGNALPLDKIPLGTSVHNIELSYNK
GGQIVRAAGTFARILAKENDYVTLRLPSKEIRLVHRTCYATIGIVSNRDNNNIRLGKAG
RKRWLGVRPTVRGSVMNPCDHPHGGGEGRATIGRPKAYTPWGKPALGVKTRKKER
SFIVRSMSRIGKLPIAIPENVEVEVKEQIINITGPHGKLVREISSTISINNFLTLKRFTNQL
YGLSRTLINNMIIGVSKKFDKTLMLEGVGYRAQIVNKDLVLNLGFSHPVLLLIPDGIEV
VLEKNGIIIIRGFDKELVGQFAATIKSKRPPEPYKGKGILFKGEIIKRKVGKSGKMTFIPS

QTKEKWYIVDAKNKCLGRVATKITNLLRGKNKITFTPSQNIGDYIIIINAEQVKVKFTQ
KTYFRHSGRPGENFETLQSRLPERVFEKAIKGMLPKNGRKLFTKLKVYSGPNHPHSAQ
KPQFIMGKKVTTIIKLALSAGKAVPAPPVGPALSQHGVNISAFCKEYNGRTADQTGLII
PVEISVYEDRSYTFVLKTPPASFLLIKALNKTKGASEPNKQSIGAITEADLRKIAETKLPD
LNTKNITSAIKIIEGTAKNMGINIMLSPKKTKFKKQQRGRLKGKASSGNTINFGDYAIQ
ALEPTWLTSRQIEATRRTITRYTKRGGKLWITVFPDKPITARAAESRMGSGKGALDYW
VAKVKPGTILFELSGVNPTLAKEAMKIASYKLPIKTKFITKMIQAQTFLTVADNTGAKK
IMCIRVLGGRRQYATLGDIIIGVVKEALPNMATKRSEIVKAVVIRTKKTVSRKDGTNIRF
DDNAAVIINNDKNPKGTRIFGPIAREIRDKDFTKIVSLAPEVIMYAIIEASGRQFWIEPKK
FVEFNRLLLGSTIILKRILFVKTTFVGQPYITIIKGIISKHFLGPKILVFKMKPKKKYRKKF
GHRQKLTRLFI-
VKAINKYIRISSTKVRKVLDQIRGRSYLEALTLLKFLPYKACGPVWQVLNSAASNAEKN
NGLNKENLKIKAIFADQGPVLKRFRPRAQGRGFQIRKPTCHITVILMPKKNLHPKWFS
ESKVYYDGQLIMIVGSTKPELNVDIWSGNHPFYTGSQRIIDTEGRVERFLKKYGMMAH
KKGAGSTKNGRDSNSKRLGVKCFHGQKVQAGNILIRQRGLHFKPGLHVGVGKDYTL
YALKQGMTVYSVSIIMKVRPSVKKMCAKCRIIRRHGRVQIICINLKHKQRQG------------
----

MCICRELSLNYGHFVFTSLDHAEGITLGNMVRRTLLSNLPGSKIVGVKISNVNHEFSD
VEGIREDILEIFLNLKEVIIKNPYGKVKIQGPAIVTAGLILLPNDVLILNPQQYLFTISEQI
FVELEVKIECGKSYILAFFPIDANFTPILNVEYHIQPSREELHMIISTNGTLLPQEALLIAT
NNLSQLILGCIDIEITCLPNRILTTLKKAKINVMADLLKYSTLQKILGPVSVKKIKDQVE
LFNKTINKKELKRILSWAFESFGQRKAAYFIDQLKGLGFEYATNSGISISLEDLRVPPIKK
ALMQTASQSISLTQAQAKNGEITEVERFQKIIYIWNLTSETLKERLIEFFKKKDPLNSVY
IMAFSGARGNIGQVRQLVGMRGLMSDPNGQIIDNAISANFREGLSITDYIISSYGARKG
IVDTAIKTADSGYLTRRLVEITQGIIVSELDCKTLRGVVLAIGRILSHQVIIAGINQEITL
DLIKIFLLIEEIIVRSPLTCECRRAVCQKCYGLNMASGELVELGETIGLIAAQSIGEPGTQ
LTMRTFHTGGVFTELARQARANCTGYVRFVPTSSLKPFRTSYGQDAFLSERESYLRIL
RIIEVKIDARTIILAKNHSCIKVDDVLFEASPNLNDISAQESGEIILKNIFWVLSGQVFSIP
GKARKFERVIKQSIAQSKVLTTISGFVEKCKAYGGIFVPQSTVQTICKYFNFGSYKSEIFP
YYITNFSGFISFTIKTITLKPGEVYFPGELFLECYDIKFLSYLEYFIPITRYEVIQFVSYPIVF
VIVDLLIEDKQFIEAYTTVANLNLLMWTDYIYSIKTKNARLLFTTKSDYKSLFFEDKSLR
VFLLKESGLFRKISGNYFLFQLAYPYLFSKGAIIRKLPGDFVQKQESLGQLVYERLKTG
DIIQGLPKVDEILEVRKPKLNPQRTIKVGQPLTINPHTLLQVYFRYFESAYRSIKKLQTL
ILRSVQTIYLSQGVVIADKHVELIVKEITKKVYVEYPGETNFLPGDIINFDQVNYINKCL
HKRLYRPILLGITKSSLKMEGFLAAASFQETTRVLTHAAIEGKIDWLQGLKENAITGRL
IPAGTGMLTINLPDLSEIQRLSFCWFLTEGLPQELANFPLILNKSGIQLVLYGHEYKIYY

PKFSVLNALKKGSDYNLKIYVLMNLALPKSGFKEYVFLGEVPLMTPKGTFIFNGCERII
VSQIVRSPGVYYFYEATIISNYGSWLSFELEYIWVKIDKQYKIPINWFLVSLGLTEIFESLR
NYDFLQKKELTRLFNNSSYDLGEIGRKKLNKRLGINLPLNVKFLTSLDILKTIDNLIDVS
FYNDEIDSLENRRVRSIGELIQSQVRIALTRLGKNFSRNEKLSILSLIREFFGLSPLSQYFD
EINPLAQLTHKRRISSLGPGGLNSDHVSFTARDIHPTQYGRLCPIETPEGQRAGLITSL
ATHAKIDQYGFIKTPFFKVYKGQVLKNGVPIYLTAEKEALYKVAAADIALNFFKEHIIPV
RFNNEFILTDSFNVNFVAISPIQILAAGASLIPFLEHNDANRALMGSNMQRQALPLLQA
QKPIVGTGIECQIALDSLVSVLNLEEGVVESVSADRIIVLYFLDKFRRSNQETIINQQPIV
WPGQLVKAGQIIADGPATEEGELALGQNLRVAYLPWEGYNYEDAIIVSERLIHDDLFS
SLHIEKYELYLMENNQGLETRDIPRVLSKLDKNGLVKKGTFVRGGDILVGKITPIIASE
ELPESKLLRAVFPDDTSLKVPVIDISIASRISIAQIKKIKIGDKISGRHGNKGVISKIVPIED
LPFLPDGNTIDVLLNPLGVPSRMNVGQIFECLLGFAGDYLNQRYKVLPFDEMYCSEA
SRILVNKTLCKASTKNCWLFNPSSPGKILLTDGRTGENFDNPILVGKPYLLKLMHLVD
KKMHARSTGPYSLVTQQPLKGKSNNGGQRFGEMEVWALQAYGVAYTLQELLTLKS
DDMKGRNEVYKAIPKPRIPESFRVLLRELNSLGLDLTAYQLEKLNNVDINLMMYQCIQ
INLASSERIKKWSEITLP-
NGIVGEITESDTINYRTHKPERNGLFCQKIFGPIKDWECICGLYKICEVCGVAITESRVR
RHRMGYINLLHPVVHIWYLRGIPNFLSIILYEEFEGAEIIYKKLKRLDLKEEIKNNRLIMV
SSKKAVEDAIKRVRIFENFLATKTKPEWMIFNVLPVLPPGLRPIVQLDNGRLATSDLNE
FYRKILIRTQRLGRLLSVQAPSMIIFTEKRMIQESVDALIDNGKRGPKVLDIHKRPLKSL
AEMIEGKQGRFRQNLLGKRVDYSGRSVIIVGPQLHLNQCGLPYEMAVELFLPFLIIFFL
KAKIIYSNSFIWYLLEILNKHPIILNRAPTLHRLGVQAFDPVLVTGRAIQLHPLVCPAFN
ADFDGDQMAVHIPLSLESQLETRLCLLATNNFLSPATGEPNILPSQDMVLGFYYLTT
QNRIKLKGNNNYFSTFNEVISAYQQKKLHLHSSIWVRCFLTQYLRTTPGRIIFNRRINEI
LMVRYRGPKKKIVNKFGLAGLTQKTKREQKKDKSAYKIRLNEKQKLRYYYGVNESQLI
RYVKEAKRRKGLTGFVLMQLLEMRLDNIIFRLNLAPTIPAARQLVNHGHVYINKKQV
NIPSFQCEPGDSISFNNRPEILDMLKSNLINDHLEFDSNTLLGQVNSLIEEHNLIFTIND
MLVIEYYSRMGHKTHPLGFRLGILNPDQSFWYSSPNSYTLNLKEDYEIRKLIFKLVTNII
IKRSKKIYIEIKSVAPQIVLDKISSLVKTILINIIKITKPYTEATLLVDVLIFNLQKRISFRKCI
RKILERYINGIKIQIAGRLNGAEIARTEWVREGRVPLQTLRADIDYCYKTAQTSYGILGI
KIWIFKMTTDIIADMLTRIRNANLVRHRLVQVIKTNLTISVTQILKEEGYIADYEEIKKS
YLLLSLKYYNQKRQPIITGLKRVSKPGLRIYVNKNNLPIVINNLGIAILSTSKGILTNNKA
KKLGVGGEVLCYIWMWEKRVVKVSRVSKVVKGGKKISFRATVVIGDKKNKVGVGMG
KAVEVSNAIKKAETNAKKNVITITVESGQTISHSILGAAGGSKVFIRPAVPGTGVIAGGS
VRSVLELAGIKNILSKQLGSNNALNNARATINGLRNLMSRRINKKRKFPIADPLYDSYL
VNLMILKILKSGKKRVAQNILYESFTIIKRKTKTHPLKIFEKAIKNVSPSVKIQAKRVGGS

TYQVPSEVKKFRGINLGLSWILKYAKTRSGKTIAIKLANEIVEASKGFGNSIRKKEETHR
MARSNKAFAHFRRMLRIILQSFNHKILNESCNKIINLISQEEIKFWGPISLPTTKRSYCVL
RSPHVNKDSREQFEIRRYKKIIDVSPSSELVKVLFDIPPGVSSKLMPTIQQLIRVRRKKIQ
ARGKSPALKNCPQRRGVCTRVHTITPKKPNSAIRKVARVRLTSGFEVTAYIPGIGHNL
QEHSVVLIRGGRVKDLPGVRYHIIRGTLDTVGVKDRRQGRSKYGMKKPVMKKKKKP
KFTGTMHVHATFNNTIVTISDANGDTISWASAGTVNFKGSRKGTPFASQKAAEQAIL
KAYDYNVKKVNVILKGSGPGREPCIRAVHQKGIRVLSIEDITAIPHNGCRPPKRRRIMV
RLRGVDLAKEKKVKYALTSIYGIGVSRAENILEKCGLSGLRVKDLSDDDISNLRKVVED
YQLESDLVRLNQLNLKRLMENGCIKGRRHRAGLPVRGQRTRTNSRTKRKAIKSKKMK
KQQRVGIIISNKMQKSVVVAIEYRYKHKIYSKIIVKTKRYLAHDELNNCNIGDEVLVEAS
RPLSKKKRWVVRMAKQSMIEREKKRVKLVNRYNEKRKLLLSEFKESKTFLDKQVIHQK
IEKLPRNSSNIRIRNRCWQTGRSRGVYRDFGLCRHMLREMAHNGMLPGVRKASWMA
RSYRKGPFVAYHLLQKIDELKKVVVKTWSRSSTIIPAMIGHTIAIYNGKKHFPLFISEQII
GHKLGEFIPTHNFRSHKSDRRLKKMTNSAKKRIRTNRRNNERNTIYKSRSKTFIKNYLA
LTQSYVKNSLNLALSQLDKAAKKNVYHKNNIARKKSNLLKVYNQMTQIFNEEGSALP
VTVIRVGPCLITQIKNLKTDGYNAVQIGYSKPKNIKKSQLCHFQKSGLPAFSHLCEYKN
LEQDYLLGQEITVDIFKMGQLVNVTGKSVGKGYSGNQKRHKFKRGPMTHGSKNHR
APGSIGPGTTPGRVFPGKLMSGQLGNKQITISKLEILGINLNENLLVLKGNVPGKPGN
LLKVTMAKAKDIRIIITLKCTECNKNSQSKRKQITYSTTKNRRNTPERLELKKFCPNCN
SHTLQKELKMTQIFNEEGSALPVTVIRVGPCLITQIKNLKTDGYNAVQIGYSKPKNIKK
SQLCHFQKSGLPAFSHLCEYKNLEQDYLLGQEITVDIFKMGQLVNVTGKSVGKGYSG
NQKRHKFKRGPMTHGSKNHRAPGSIGPGTTPGRVFPGKLMSGQLGNKQITISKLEIL
GINLNENLLVLKGNVPGKPGNLLKVTMQKYITYIPLINGKQLQLKVLSSGNYLLHKDL
LRHVNSQNQGTVSTKTRSEVRGGGRKPWRQKGTGRARAGSNRSPLWKGGGVTFGP
KPKTTSLKLNKKERRIALQTLLYNKKNNILLIENLIPSKTKTFLKICVNLEQKVLVIVSKK
TDSLKLSTQNLQNVELISAANLNTFSLLKAKQIILTPSAIKDIKEIYMLKIRFKRGGRKK
HPFYRIVVINNRTKRDGKVIEELGSYNPLTKIIKINLVRTIIRLEDGAQPTEVVKNLFRKL
FMLIKQPLTTLKTSQLFEKNQYTFLVDPKLDKPTIKKAIEFLFSVKIIKINTCNLPKKTR
RRPRYKKVVVKLAKENTIDFFSMSVEKNDIKMVLVGDTVKIGISIKEGNKDRIQYYQGI
VISKKNTGINLKIVVRKTFQGIGVERQFLLHSPKFQSVQVLKSARIRRSKLYYLRNISGK
ASRLKQKF
>Emiliania_huxleyi_AY741371.1
MTKNLYPKLLKISINRGLGEEARSSKEMDANLKELAVIAGQQPTVNKARKSIAGFKIRD
GMPVGASVTLRQDRMYAFLERLIHITLPRVRDFRGISAEGFDGRGNYNLGIKDQLIFP
EISYDDVNQLQGFDISIVTSANTDEEAYSLLKKFGMPLMVRVKRGNVAAKRRKKILKL
AKGFKGAHSRLFRTANGQVMKALVYSYVGRKRRKRDFKRLWICRVNAASRSLNYSKL

RNLMKQASLNINLKMLAQLVLFDKKAFSQIIMAIRLYRAYSPGTRSRSSVYYTDITQQK
PEKSLVFGKKACSGRNNRGIITLKGRGGAHKRKHRIIDFKRKSIATTARVATIEYDPNR
NARIALLHYENGLKRYILAPRSLKVGMEVCSGPEAPIEIGNSLPLAAIPLGSTVHNIELT
LGKGGQIARSAGTYAQIIAKEGDFVTLKLPSNEVRLVYKECYATLGQVGNIDAINTCL
GKAGRSRWLGKRPKVRGVVKNPIDHPHGGGEGRSPIGRAKPVTPWGQPALGIKTRK
KTKSYILRGMSRIGKQYISIPDKVNIRLEGQKVFVDGPKGKLTRILPSFITIDKKVFLNKL
SQALYGLSRTLLSNMVTGVSSGWHKKLTIAGVGYRAQLDGKDLILNMGYSHPVKMV
TPPALSVSVESPTSITVSGLEKEAVGEFAAKIRSVRPPEPYKGKGIAYEGEVIRRKAGKT
GK--

MLLPKRTKFRKMHRGRLKGVATRANTVVFGEFGIQALEPVWLTSRQIEATRRSITRYV
RRTGKIWIRVFPDRSVTERAAESRMGAGKGAVSYWVAVVKPGTVLFEINGLSEEMSH
QVLKSASYKLPIKTKIISRMIQPQTCLNVADNSGAKKLMCIRVLGTNRRYGHVGDVIIG
VVKDATPNLTVKRSDVVRAVIVRTKQSIRRKDGSRLRFDDNATVIINKENNPRGTRVF
GPIARELKDNGFTKIVSLAPEVLMYAIVEACGKQMWIEEGKFYDFDKLPFGDVFSLSDI
LLVKSAEIGQPYLTKVELRVLQHFSGPKIRVYKMRSKKKTRKTFGHRSKLTRVLVMTM
AVARYVRMGPNKVRRVLRQITGKSYSEALLLLEFLPYKSCDPIIKVLRSAVANAKNNLG
YDTTKLVIKKAFADQGPVMKRFRPRAQGRAYRILKPTSHITIVVMPKSAIHPDWYPNA
KVYCDGQLVMKVGATKPTLNVDIWSGNHPFYTGSQTIIDTEGRVERFMRKYGMMA
HKKGAGSTKNGRDSNSKRLGVKVYGNQAVKAGGIIIRQRGLTFKPGSNVGVGKDYTL
FALKEGLVKFEVSLDMKVVSSIKKRSKDCQVVKRRGRIYLICSDPRLKVRQG------------
----

MVQCLESATEVAKFCIEPLSKGQGITIGNALRRTLLSNIPGTAIVGTRISGVDHEFSVIP
GVKEDALEILLNLKQLVFKGNITRLNIQGPCIVTAGDIDIPTDLELIDPQQYIATITDFS
HLEMEFILEQGESYVLSFLAVDAVFTPIKKVNFFVETSKERLIIEIETNGSILPLEALNLA
AERLSSLFKLVVLIEELELSVRAYNCLKRAQIHTLGELLKYSKENLLFGQKSANEVCENL
HEENNLINKRSLKDLMYQAFLNYGIVKSSIIADRVKNLTFHFATQSGVSLSVEDLRVPA
KKRELIGLTRNEVETTQKRYEIGSITSVEKFQKTIDIWNNANNFLKDDVLTYFRESDPL
NPLYIMAFSGARGNISQVRQLVGMRGLMSNPQGQIIDLPIKSNFREGLTVTEYIISSYG
ARKGLVDTALRTADSGYLTRRLVDVAQDIIVRESDCGTTEGLWALRGRLLAEPIFFAP
ANTEITETFLQTLTLHSPIKVRSSLTCTSTRSVCQNCYGWHLSHSKLVDLGEAVGIVA
AQSIGEPGTQLTMRTFHTGGVFSDLTRQVRSPMEGKIEYWEGHKASLFRTMHGKMG
FRLKEEVNLIIKNTISFEIPAESLLLVNTDQYVYENEIIAEIQKDQKEIYSETSGEVFFNAL
IWILQGSFYELPAFSETLLKPKNTLLSRTPLYNKYPGWVQIFQLTGGIVIPEETFKFSTL
LSFSKNESEEIMPNTFSKVGGFFQIAEQEVTIKPGEFVKPGNFIVGKIITQQLVYLELAR

PVQVFEVINLLQTFIVFTTTRNFVSNKQYVASNTLIGQIETTAGLVNKLANSGNMKEV
LILNPSDLKKVFCPNVENDNVKSPYSGQILEIFEDHILIRLSQPYLISAGTILHATSNNLV
KAGDILATLVYETIKTTDIVQGLPKVEELLEARKVLHNALLFVEVSSALTISPHTKLETL
FSYFEACKLSFKLLQLFLIGEIQRTYRSQGVDIADKHVELIVKQMTSKVCIEDSGTTTFL
PGEVLNFQKMADIALVAENKSYVPLLLGITKASLNSDSFISAASFQETTRVLTEAAIEGR
KDWLTGLKENVIIGRLIPAGTGMYKYLLPNLLDVQRASYCWFLEKGFVQELRTFSVIR
DLGDLELNFATKFYTINPPRYTLEEAKRKDATYSVRIFIRAQLLEGADPQEKQVFLGDI
PLMTDSGTFLVNGIERIIINQIVRSPGIYYIYTASLISNRGTWVKFEIDKVYIKIDKAKKFS
IFVFLKALGLDEIFNSIENINYFRKTDLAKFFDSKKYDLGYVGRHKLNQALMLDIDSDV
RILTSLDLIGIINQLIRFRFVSDDIDHLGNRRIRSVGELMANQIRIGLNRLERVVRERMAI
KLAASIREFFGSNPLSQFMDQTNPLAELTHKRRISVLGPGGIARDRAGFVVRDIHPSQY
GRICPVETPEGPNAGLIGSLSTYGKINAYGFIETPFYKVKQGKVLKDLPPIYLDAIAEEQ
YKLAAGDVATDVLKQPDVPVRYQQNIVTVSKNEVDYIAVSPIQVVSLATSLIPFLEHD
DANRALMGSNMQRQSVPLLYADAPLVGTGLESQTARDSGMAILSSNCGKVVTLSDE
QIVVQYNLTKYKRSNQDTCINQKPCVSLYEKVRVGQLLADGTSTETGELAVGQNILIA
YMPWEGYNYEDAFVVNERLLYDDLHTSIHIEKFEIESRQTKLGAETRDLPNVLSKLDE
NGIIHIGSWVEPGDILVGKLTPKGESDYLPEGKLLRAIFVRNTSLKLHVLDVKIFSKVYI
AQIRKIQIGDKVAGRHGNKGIISKILPRQDMPYLPDGTPVDILLNPLGVPSRMNVGQI
FECLLGLAAEHLNARFKVIPFDEMNGVEASRGFVNNTLMEAAEKQPWVFSTQHPGK
MMLTDGQTGEMFDNPITVGRAYVLKLVHLVDDKIHARSTGPYSLVTQQPLGGRAQ
QGGQRLGEMEVWALEAFGAAYTLQELLTIKSDDMQGRNETLNAIPRPGTPESFKVLL
RELQALCLDIAAYKIEDTEDKEINLM-FDYVKINLASPSRIREWGERKLP-
NGVVGEITKPETINYRTLKPEMNGLFCERVFGPVNDWECHCGKYKVCERCGVEIIDS
KVRRHRMGFIELASSVTHVWYVKGRPSKIALILGMTVKGAEAIKTLLKNVDLQQSASEI
RELLPFARFFSEKLIRRLRVINQFIASKFDPTWMILDILPVLPPDLRPMVQLDGGRFATS
DLNDLYRRVINRNNRLARLQEIVAPELIIRNEKRMLQEAVDALIDNGKRGRAVVGLNN
RPLKSLSDIIEGKQGRFRQNLLGKRVDYSGRSVIIVGPELKLNQCGLPSEMAIELFQPFV
ILIYEGAKIIQKNPLAWEILNVMEGHPILLNRAPTLHRLGIQSFEPILVSGRAIRLHPLVC
PAFNADFDGDQMAVHIPLSLEAQSEARLLMLAPNNFLSPATGDAILTPSQDMVLGCF
YLTANNPSQQLNKNHYFSDFEDAILAYQNSQIHLHTFIWVRCTKTKYIRTTPGRILLN
EIFQ---
MARYRGAKLRITRRLGLPGLTSKIAKRTQKKPTQYGIRLEEKQKLRFNYGISEKQLMN
YIRQAKGIKGTTGTILLQLLEMRLDNLIFRLGLAPTIAAARQLVSHKHIQVNNTRVSIP
SYQCQPGDVLSVRDNAASKSLVNTYLSPQHLDFDKKNLTAKVLGIVDREWVALKLNE
LFVIEYYSRMGQKTHPTGFRIGINKSHDSTWFANYGTYGEILKEDYKIRKFFENDVSKV
EIKRKNQLELLIHAARPKAIAAQIKKLTKQTRIKVIQVNKMETESVLVARALAEQLEKR

VAFKRAIRLVAQRLKKGFKIQVSGRLNGAEMARDEWVREGRVPLQTLRADISYATARA
YTTYGVLGIKVWIFNMVNDTIADMLTRIRNANLAKHQIVQIPLTKVTKSIAQVLLREE
LINSFEELRQSSLLLSLKYTGKTRTPSIQKIQRISKPGLRIYSGAKKMPRVLGGFGTAIIS
TSNGLMTDTEARQKNVGGEILCYITMWEERVVSVQRVTKVVKGGKKLSFRAVVVVG
DQQGKVGVGVGKASDVSTAVRKGVTDGKKNVITVPLTSSNSIPHKINGRFGAAKLVL
RPSAPGCGVIAGGAPRIVLELAGIKNILSKQLGSNSLLNNARATIDGLSNLMSRRSKSKR
VLAQPDPIYNSRLVNLLVIRVLKSGKKSVAQKIVYTALEIIAEKTNENPILLLEKAVTNV
TPQVEVKARRVGGSTYQVPIEIRAYRGTNISLKWLIEFARERSGRGMASKLANEIIDAA
KNAGGSIRRKEQTHKMAEANKAFAHFRRMLRIALKAYETSLLNDSCTQIINAVETGGV
KAIGPIPLPTKRRIYCVLRSPHVNKDAREHFEMRTHKKIIDVYKPTDVMENLLDLAAG
VDVEIMPTIQQLVRFERQTSEKKTKSPALKSCPQRRGVCTRVYTTTPKKPNSALRKVA
RVRLTSGFEVTAYIPGIGHNIQEHSVVLIRGGRVKDLPGCRYHVVRGSLDASGVKDRK
QGRSKYGGKRPKMKKSKKTVNAGIVHIKSTFNNTIVNITDKQGNTLFWASAGGCGFK
GAKKSTPFAAQSAAEKVGSMAVEQGMKQAEVIISGPGNGRETAIRALQSCGLEVLLIK
DITPVPHNGCRPPKKRRVMVRISGVDLPRNKRIQIGLTSIFGIGNTSADKILTTANLSDI
RCSDLTDEQVSSLRTIIESYQTEGDLRRVYSLNIKRLTEIGSAAGRRHRVNLPVRGQRT
RTNARTRKGKVKTKKMVAKEKIGTVVSDKMLNTRIVAVSDRVSHKNYGKVITRTKRY
VAHDIESNSKIGDKVKIQETRPISKSKNWILVMAKQSMIQRELKRERLISKYSAKRQLLK
EELKTVSSYNDKLAIYKKLEKLPRNSSPNRHRNRCWATGRSRAYYRDFGLSRHVLREM
AHEGLIPGLTKSSWMTRSLKKGPFVAAQLMDKINKMKKDSITTWSRASTILPTMIGH
TFAVYNGRQHVPVFVTDQMVGHKLGEFSPTRTFRSHKKDKKSKR------------------------
----

MTQIFDKDGSAIPVTLVKADPCQVCQIKTTKTDGYDAIQVGYLELSKISKPIQGHLQK
CGSTGYRKFGEFRVEDETYNLGDQITTSAFSVGQKVRVTGTSIGKGFAGNQKRHNFS
RGPMTHGSKNHRLPGSIGAGSTPGRVYPGKKMAGHLGNTKTTIKNSEILFVSEKENIL
ILKGSLPGKAKNILRIAMAK-KGARVQVTLEH---KCEQ----
GVYRYHTTKNRRNTTDRLELKKYSPVTKQHEIFKEIKMTQIFDKDGSAIPVTLVKADP
CQVCQIKTTKTDGYDAIQVGYLELSKISKPIQGHLQKCGSTGYRKFGEFRVEDETYNL
GDQITTSAFSVGQKVRVTGTSIGKGFAGNQKRHNFSRGPMTHGSKNHRLPGSIGAGS
TPGRVYPGKKMAGHLGNTKTTIKNSEILFVSEKENILILKGSLPGKAKNILRIAMDKVL
TFPIKLVGVDLQVKGRETENYIIYRAYIAQRINKRQGTVSTLTRAEVRGGGRKPWRQK
GTGRARAGSNRSPLWRGGGVSFGPKPKLYSNKLNRKEWQLAVRSLIMLKEPIIKVIND
FSLIKTKVFINLFINFSQKITFIVPKIEESLFKATRNIKTVKLMRADTLNLTTILQSDHLFI
MKDSLKIIEETYMLKIRLKRFGRKAQPCYRIVVTDSRVKRDGKALEEVGFYNPLTDET
HLKFNRIVERLKTGAQPTETVRNIFLKAKMLIKYPILTEKTSRLIEQNQYSFAVDRKAD
KISIKSAVEALFDVQVVAVNTANQPLKKRRKSRVKRAVVTLAPENSITFFEMSYKKENL

PKFRVGDTVKVTLYLAKKGKERIQVYQGVIISTHNNPTNATITVRKMFQGGGIEKVFL
LNSPWLKTVEVLTSAKVRRGKLYYLRDRVGKSARLKRLF
>Eustigmatophyceae_sp._MK281455
MYKKIYPKLLKIQINRGLGLGAQNRMILQKTVEEIRFITGQQPIVTKAKNSIAGFKTRE
GMDLGVTVTLRNDFMYAFLEKLINLVLPRIRDFRGLNPNGFDEHGNYNLGIREQLVF
PEIDYNMIDQVRGYTISIVTSAKTQDEARTLLQEFGFPFMVRVKRGNTARKYRKKILN
FSKGFRGAHSRLFRVANQQVLRSWRNSYIGRKNKKRFFRRLWIVRINIASRKLPYSRFIA
QLKQEQIGINRKMLAHMATMDPKTLSILSMGIRFFKPYTPSTRHSIRSDFKELTGSKPE
KRLLVKFHSAQGRNNKGRITIRHRGGGHKRRYRLIDFKRNKFNIRGKVISVQYDPNRN
ARIALVAYEDGEKRYIIQPDNLTSGNLVESGPNSSLNIGNALPLENIPLGYEVHNVELYP
SRGAQLARSAGTSAKILAKEGDFVTLKLPSKEVRLVRKECYATIGRVGNTSEMNFTIGK
AGRSRWLGRRPTVRGVVMNPCDHPHGGGEGRSPIGRKHPCTPWGKPALGVKTRTR
KKTFIIRKMSRIGKQSLNIPKQVEVKINESSIFVKGPKGELTQIIPDSLEIDNTLQVLRLS
RALHGLIRSLVSNMLIGVSQGFNKVLDLKGVGYRASVDKGVLNLTVGYTHPVKIAPPP
GIKISVDGNTTVKVEGVDKEKVGLIAQQIRFVRPPEPYKGKGIMYRGEIIQRKVGKSSK
MTFIPLSKSITWFLVDAADQTLGRLSTQVANLLQGKTQVTYTPGSDSKTYVIIINASKI
KVKATQKFYYSHTGKPGRSFSELSDKNPEKLLELSIKRMLPNGKSNLSKRLKVYKDIQ
HPHTSQQPKLIMGKKVVAIVKLALKAGKATPAPPVGPALGQHGVNIALFCKEYNAKT
ADKGDLIIPVEISVFEDRSFSFILKTPPASVLLKVALQIKKGSSKPNRIKVGSIDKKALAEI
AKTKLPDLNTSNLESAIKIIEGTAKNMGITIMLSPKRTKFRKQQRGRLRGKTIQKKHLV
FGDFGLQAQEPVWLTARQIEATRRTITRYTKRGGKLWIMVFPDKPVTARAEESRMGS
GKGAPQYWVSVVKPGKVLFELSGVPESIASQALKMAAYKLPIKTKFITKMIQPQSYLN
VVDNTGAKKIMCIKVLGSNRRYGKIGDVIIGVVKEAIPNMPIKRSDIVRAVIVRTKHLIQ
RNDGTALQFDDNAAVIINLDNNPRGTRVFGPVAREIREKNFVKIASLAFEVVMYSIIES
CGRQFWVEPQKFYDFYDFRNADKILFDQVLILTDVYLGHPFLDFVEASLLPGIKQSKL
YVFKMRSKKRFRRKIGYRMTSQRIKFMSKATARMIRMSPLKVRRVLKQIIGCSYEEALIL
LRFLPYRACHPIAKVLKSAGSNAIKNNFIPESYLQVCEGYVDKGPILKRIRPRAKGRAYP
IKKYTSHISISVMIKKLIHPKWFPTTKVYCDGKQIMTIASTKPELHVDIWSGNHPFFTGS
QKIIDTEGRVERFLKKYNIMAHKKGAGSTKNGRDSQAKRLGVKMIGSQPIKVGQIIAR
QRGYTIKAGKNVGVGRDFTLYALKDGHVNFTVSVKMKVRPSVKKICEKCRLIRRAGKI
RVICKNPKHKQRQGMAVPKKRTSKTKTRSRQANWLAKARNQVQKAWSLAKSVANST
SFLLMRLKIELQKRAFQFQIGPFKKSMASTIGSALRRTLLSLSETVSITSA-----
FGNLYNGNSLREDLFELSLNLQKVTIKSSVARIRKKGPAIITAQDILVQEGLKIVNPYQY
ICTLNESYTLDLSIIISSPTMNQGIIILVDPIYSSIQSCSFEVTQTDEFLQFVLVSRGAIEPV
EAIQFAANELRNDLIIFFDIRHLSLPPKLELFLRRQGFVSLENLISIPVFKNILTQDEFKTI
ESSIKNFNQIITKKDLEEILSWMFKNYGVRKTCVLAELFKESGFQFATQGGISLNLEDL

KVPTLKKQLVSVTKKNLINSEIKYKRAELTTSEWFQKQVNEWNATSELLKDEITSFFED
TDPLNPLYMMAFSGARGNLSQVRQLIGMRGLMADQKGEMIGTAIQKNFREGLTVID
FLISSYGARKGLVDTAIRTADSGYMTRRLIDIAQDVIIRQFDCKTANGILILFGRILCKPII
IAHRGQEIDFNLSKRISLIESVSLRSPITCQSYRSVCQICFGWDISHHRLIEIGEAVGIIAA
QSIGEPGTQLTMRTFHTGGVFSELSHQIRSKFSGQVRFAPNLKTQFLRSNEGDYGQVI
RNASFFEIIQVVRVWVERDDLLMINDNDFIKKGQPIVKSSSNLEELSTKFPGEIFYEYIL
WVLAGNLYTLPEKVIRSNKSKNNLLGKTKLVIRSNPFTLYIINSNGGILPEETYLGLSTL
NYIGSFIQKLTKKIISPINGIIRRKVQQIILNSGGFFFPGEIVLDKFLVKKVIFTQLIRPVCF
YSIRNIVEKGLIVSLIKVLSRPKQVLEPYTVFGEFQLSTLIQKIELVKTKLQELMIVTNDN
VRNFYLDENPFDMLLLTVGGKIMNQVGSLTKFRSGLPYLFTEGAKIYKNHEDFAPEN
TVLGFVTYRIFKSQDIIQGLPKIEEILEARGSLAHARSQIELSEKFHVDPQQILETYFEYF
KAVLRSLYKLASIFIKSIQAVYENQGIGIIDRHLEIIIRQMIAKSKVEHPGGTPLVSGEYLE
NYHLDLLNQMLSKKYYKPTILGLTKAALTTESFISAASFQETVRILTQAAIDGKVDWL
RGLKEKVIVGGLIPSGTGMFLANIPDLVAIQKTSFCWFLEEGLANELSELSKLADFETY
EVRFFTNEYYFYYPFRTYNECRRDKLTYSIRMYIPIEVEDRRITESHVICLCELPLMTNR
ATFMINGCERVILGQIVRSPGVYYSINATLISDRGSWLYFEYDRIWIKTDRLERIPLSILL
DDIGNEGVLGHLENAHYFEYWELEEIFDPRYYNLGKVGRFKLNKKLSLSIPTNIHGLTI
HDLVEIIDYLIGLRVLIDDIDSLENRRIRSVGELLQAQFNQAFMRFGSFLEETRDMPPV
SAVDEFFVTNPLSQYLDQINPLAEVIHKRRVTVLGPGGIPFEKASLAMRDIHPSYYGRL
CPIDTPEGEKAGLVASLATFVDTDTNGFLKTPFFAIKKGQICGLS---
QLSVADEEGRSIAMGDVLVGYLLTDRIETKQNDEFNSLAAYEIDYLSISPFQFFSPAIGL
IPFFEHDDANRTLMGAHMQRQAVPLVRPQKPIVGTGIEYQLAIESGCLILSYSDGIVRS
VSSTSIWIIYKLEKFQSSNQATVLNQRPIVWSGERVKSGQVIGDGPSTDEGELSLGKNV
TVAYMPWGGYNYEDAVVVSERLVYENVFTSVHVEKLEVEIQNHDRGPDTREIPEILR
NLDENGIVYVGAYVQPGDALVGKLSPKRRDNT--
YGRLLEELFVNDSSLRVPTLEVRILDQVFIAHIRRIQVGDKISGRHGNKGVISLIAPRAD
LPFLSDGTIVDIILNPLGVPSRMNVGQLFECLMGWAGDKLNERYKIFPFDEVYESENS
RILVYEKLRQVHSKKTFSSKTLGAGKVKLYDGRSGQPFDNLITVGKPYILKLIHLVDDK
IHARSIGPYSIITQQPLGGRSREGGQRFGEMEVWALEAFGTAHTLQEVLTIKSDDIHG
RLGAYRAIPSPGIPESFRVLLRELRSLAVDIRALRFVTHYPGIKSVRMFEMVGVKLASPN
RIREWSERRLP-
NGIVGEIRKPDTMNYRTGRPEAEGLVCEKIFGPLKSWECGCGQYKYCPNCGVEVTES
HVRRHRMGHVVLNYPVTHTWYLKGSPSYLSIILIISNTGSKLILSMLEELELPNEISSLR
QQILTSTSKRERLLKRLRLLETFVATQSEPSWMVLTVLPVLPPALRPVFELPNGTYMSS
EFNEHYRRIVMRNNRLLRFCEQVVPDFIVINEKILIQDAVDDLIDNGSRGRDSYGPRN
KPLRCLNDLIAGKEGRFRQNLLGKRVDYSGRSVIVVGPTLMVDQCSLPYEMALDLFG

PYLIIIPRALKALERNPIVWIFLLIVSMTLVLLNRAPTLHRLGIQAFRPILTTGRAIQLHP
LVCPGFNADFDGDQMGVHLPLTRKTQLEAYR-
MLSSNNLLSPASGKPILTPGQDIVLGWYYLTTRTLKNLPSSNTYFTSFAEVSDALENKK
LTIHSPIWVRYIILCYICTTPGRILLNEIIGVAIMSRYRGPRLRLQRRLGLTHLTRKRVKN
KKKIRSEYRMRLEEKQKLRFNYSITESQLFKYVQEARRIKGSTGLIILQLLEMRLDTIVFR
LNFSPTILSARQLVSHGHVKVNGRVVTIPSFQCQPGDTIELKNKIVTSDPKYQANLPY
HLAGSQKDLKGGIKRIVDRRDIKLPVNELLVVEYYSRMGQKVHPIGFRLGITEEHKSR
WYAKPSNYSQQLQVDNELRQIWTNIRYNLLIMQPDQIHLHINTTNPLLILSVLTQYLK
KLVLVINKIKHPLLEPNLLAQSIAKKLEKRMPFRRATRKILDDFKRGIKIQVAGRLNGA
EIARTEWVREGKVPLHTIKAKVGYACERAQTIYGILGIKVWVCKMTIDTISDMLTRIRN
GNSVGHQVVKVNSTRINYRICRLLKSEGFIRQFETVGKRYIFIYLKYENESKKPIIRGLKR
VSKPGLRVYVRKRQIPLVLGGQGLALLSTSQGILTSLQAKKLGIGGELICYIWMIDHKV
VEIRRVCKVNKGGKTLSFRALVVVGNQNGTVGIGIGKASQVLNAIKKGQYRAIQNRIRI
PMTQTKTIPHKSEASYGASKVVLRPAAPGSGVIAGGAIRAVLELAGIKNILAKQIRSKNK
INNAKATVLSLKNLMSRRKRSRKRLPGADSVYNSYLVSLLSLRVLKNGKKKLADRIVYK
ALELISEKTNTDGLITLEKAVQNVSPIVELKPKRIGGATYQVPIEVKRLRATNLALKWL
LRYSRERAGKNMSFKLARELMDASKGIGSSIRRREEVHRMAEANKAFSYFKRMFRILLK
AYNSKVLNISAFIIQQELQRLDTVLKGPIRLPVKKRYYSLIRSPHIDKHSQEQFEIRRHK
WIFDLEVPQNYVNLLGSFPPGVEISVMPTVQQLIRNERQILKKKSKAGALKACPQRRG
VCTRVYIVTPKKPNSAMRKVARIRLTSGFEVTAHIPGEGHNLQEHSVVLIRGGRVKDL
PGVRYRVIRGALDTVGVTKRTNGRSKYGARKARMGKKKINLSLGFACIHSTFNNTIISI
TDPKGNVITWASAGSSGFKGSRKKTQYAAQMAAKNASAKALKLGIRKVGVILNGPGN
GREIAIKGIHSTGLEIISIEDKTGVPHNGCRAPKRRRIMVRIAGVDLPDNKAIKISLTYIY
GIGHTRANEILSTTNIDETKTKLLADTDISKLRDLIENYTTESDLKRLVALNIKRLIEINC
YRGRRHIQSLPLRGQRTRTNAQTRKRTAKSK-
MGLKEKIGIVVSTKMQKTIVVLVENKFRHPRYAKIMVRTKRYLVHDEENKAKIGDRVV
MLQSRPLSKLKRWTLEMAKKSLIQRQIRREDLVRRYSEVRQGLKNELKTAKSFRAKME
LHIRLQKLPRDSSKTRLRNRCWKTGRGRSVFRDFGLCRIVVRDMANQGFLPGVIKSS
WMNRSTWKVPFVAHHLLKKVNKVSNETIKTWSRASVILPTMVGCTIAVYNGRQHVP
LFISDQLIGHRLGEFVPTRTFRSHKNDRKARRMIKSAKKKVRVGRRNAKINKSYKSSIK
TLLKRYRQNLKDFLQTLISSLYSRIDKAAKKKVFHINKAARQKSRIVSSLKKMTQIFDEK
NVCIPVTLIKVGPCIVTQIKTVDKDGYNAVQLGFGFDKPANLPMKGHLAKSGITNCQ
HLREYYIEKEDFQTGQKFDITNFQSGQYVSIKGKSIGKGFSGTVKRYKFGRGPMSHGS
KNHRAPGSIGAGTTPGRVFPGKRMAGRLGGKPVTIKRLPILRIDTNENVLVVKGSVPG
KTGNLLNLIMAKQKGTRIIIHLECTNCRTSSKRAEGVSRYTTMKNRRNTPSRLERKKF
CPYCNSHTIHRETKMTQIFDEKNVCIPVTLIKVGPCIVTQIKTVDKDGYNAVQLGFGF

DKPANLPMKGHLAKSGITNCQHLREYYIEKEDFQTGQKFDITNFQSGQYVSIKGKSIG
KGFSGTVKRYKFGRGPMSHGSKNHRAPGSIGAGTTPGRVFPGKRMAGRLGGKPVTI
KRLPILRIDTNENVLVVKGSVPGKTGNLLNLIMQKFITYTPLVYGKQLKLNVLTSGNY
LLHKDLLRHLNSQRQGTVSTKTRSEVRGGGRKPWRQKGTGRARAGSNRSPLWKGG
GVTFGPKPRQFELKVNKKERRLALQTLIYNKRNKLVIINNLLTPKTKAFLKICISMDQK
ILVVTSKKTPTLKLATQNLKNVELISAGHLNTLSLLRAQQIILTPLAINDIKEIYMLRIRL
KRCGRKHAPSYRIVLAPSRARRDGKAIEELGYYNPLRNQLEYKKERILVRIQQGAKPSQ
TVKNFLIKVLMILKYPLITEKANLLDSKNQYIFVVESKTDKQTIKKAFEYVFSVKVSSVN
TMTLPRKKVRKPRYKKAIIKLAPGYNLEFFGMIVDKNERPEIAVGDTIKIGILLIEGTKE
RTQYFEGTVISIKSREGRKTIRVRKISQGVGIEKVFLLDSPRIVGIERKKVTKVRRAKLYF
LRNLGSKTKKLKDES
>Fistulifera_solaris_AP011960.1
MIKKEYPKLKKIVVNRGLGVSAQNTNILKKNLEEFEKITGQKPVVSRAKKAIAGFKIRE
DMELGISVTLRGEKMYTFLTKLLFFTFAQIRDFRGLSLRSFDKAGNYTFGLKEQLIFPE
VEYDEVDQVQGCTINIVLDHNSPKNGMILFKFLRFPLMVRVKRGNVARKRRNKILGIA
SGYRGAHSALFRVANQQVMKALRYSYVDRKKKKRVFRRIWISRINAASRLISYSKLIHKF
KKSNIDLNRKMLSQIAVLDASTFKALIMSIRLYKSYTPGTRNRALSAFSEITKSKPEKTLI
RKNQRNKGRNNRGVITIRHRGGGHKRLYRLIDFRRNKYGVEGIVASIEYDPNRNARIA
LINYLDGEKRYILHPKNLNVGDKIFAGPEAPLSIGNSLPLEQIPLGTSIHNIELIPNKGG
QIVRSAGTSAKILAKENNYITLRLPSKEIRLVRKECFATIGEVGNNDAFLVQSGKAGRT
RWLGKRPTVRGSVMNPCDHPHGGGEGRTPIGRSRPVTPWGKPALGMKTRKTKKSYI
LRRMSRIGKLPIKIPDSVTIDHRESEITVKGKFGTLQRSVPDIISLDGKLNVIKKIKALHG
LYRTLINNMVIGVSEQFTITLNLKGVGYRAAVQGNEIVLSLGYSHPVKIEIPTNISVEVL
QNTTINLKSCDKESLGLFAANIRSWRKPEPYKGKGILYKNERILRKAGKSGKLTFLPTQ
SNRNWFIIDCKDQKLGRIATIIATLLKGKVKPYYFPSIDTGDYVILINAESILIETATHHI
VNQPGRPGSALRNVSDCLPKFTIERAVKGMLTQSKKRVMRRLRIFNDENHTHVAQN
PIKIMAKKITALIKLALPAGKATPAPPVGPALGQHGVNIAAFCKEYNARTSEKAGLIIPV
EISVFEDRSYTFILKTPPASVLLANAAKVKKGSSTPNRVSVGSITKAQLEEIANIKLPDLN
TTKISSAMRIVEGTARNMGISIMLSPKRTKYRKYHRGRMRGTKTRGNEVCFGNFGLQ
ALEPSWITSRQIEAARRTITRYTKRGASLWIRIFPDKTVTARAAESRMGSGKGAVDYWV
CVVKPGTILFEISSVPEEVARAAFNLAAYKLPIKTKFIIKMICPQTMLTVADNTGAKKV
MCIRVLGGSKKFAKIGDTIIAVVKEATPNMPVKRSDVVRAIVVRTTKTIRRQDGMFIRF
DDNAAVIVNMENNPRGTRVFGPVAREIRDKNYSKIVSLAPEVLMYAIVEISGRQFWIET
GKYYDFNRIPTGKQITLNRVLLLNKVLIGQPYLEVIKGKILEHLRGKKTIVYKMRPKKK
TRKKQGHRQDLTRVLIMVKAVAKYIRMSPHKIRRVLDQIRGRSYQEALMILEFLPYDA
GGPIWQVVHSAAANAKHNYGLDKKKLVIDEIFADEGPKLKRIRPRAQGRAYKILKPTC

HITVVMMPKSDIHPKWFKETKVLYNGKPLCIIGSTKKELQVDLWLANHPFYTKSQVL
VDSEGRVEKFMKRYKLMAHKKGAGSTKNGRDSNAKRLGVKKFGGETVRAGNILIRQ
RGMSFKPGQNVGYGKDFTLFALVDGTVKFDVNIIMKVRPSVKKMCENCRLIKRHGKI
RVVCSNPKHKQRQGMAVPKKRTSKAKKNARKANWKRKGYYAAQKSLSLAKSMLKQT
SFVYMIKCLKSEKEQGRFVINALRAGQGITIGNQLRRVLLGDLGGMAISAVRIAGITHE
FSTIPGVREDILEILLNLKGIVLKSEFGRLKVQGPAVVTADLIQLPSNLEIINPNHYIATIS
TSNILEIEFKFEYGTGYKLAYLQLDTVFMPIQKVDFKIENITERLLLDIWTNGSISPNEA
LESAAQVIVDLFTLLIAIEELQLSVRAYNCLKKAQINTVGDLLQYSPLQELFGRKSADE
VFSTLKNQNTLIGKKQLRQLLAWSFTNYDSMQACALADELKYLGFKYASQAGISINIE
DLKIPFVKNLMLEKANQEILNAEKIYLKGKITNVERFQKIIDTWSLTSESLKEQVIYYFK
NYDPLNSVYIMAFSGARGNLSQVRQLVGMRGLMSDPSGQIMNLPIKKNFREGLTITD
YLMSGYGARKGIVDTALKTANSGYLTRRLIDVGQDILIREKDCLTTHSFLFIVGRILSK
PVFLIHANVQLTPQIIQQCKKVNEFYIRSPLTCNLYRAICQKCYGWDLANENLVDIGE
AIGILAGQSIGEPGTQLTMRTFHTGGIFTEARQQIISPVNGIVKFYNILETVPLRTNRGE
DVSIAKNSGSLVIIKLIQLEVVKNTILFPKNNQFVLKDTVIGELLNVNKQILSDTCGEIFL
NNLIWILYGKLYNSPNNFYPDYKLNRSYIFRTKIVSHYRGNSKNYLLTGGITLGEELHK
VNCEILLVGDFIFELIPGLFSKTSGMVIILVQTICVKCGKLYYPGEVILSNISVKNLAFCE
LIRPVEIYEFLNLVSNILSLIQSCLLLQSNQFIDRYTLLGYLEALTNSLEIVKFKIKTKQIFL
ISNEDCFTVKRNNDKKDFINVNETGKVIIVNKTFLTLQKGRPYFFQEVNSVFCKNSEFI
EEGETIGLLNLEKEITGDIVQGLPRIEEILEARKKSTSQKKVQKLGTAIKINPHKLLKVY
FNYYEGSYKSFKKVQAFILNSVQSVYQSQGVSINDKHLEVIIKQMTTKVLITYEGNTPL
LRREVIDLYHIRYINQIVQLQCYIPLLLGITKAALNNPSFISAASFQETTRVLTKAAIEGRI
DWLRGLKENIIIGHLIPAGTGMYINALPDFLAMQRISFCWFITEGLNEELSLFSRIQDFQ
NTEYIMFGEEYRLIKPSYNLLIARKYSGNYRAQLVIPIEVRNKIVKYQKQFPVITFPLMT
TDATFIINGCERVIVSQIIRSPGVYFLYTATLIPEYGSWIRFSFQKIIIQLDKVNQKPILHL
LKEMGLTEIYQNLEHSEFFYFSEFSKIFDPSYYRLGRVGRLKLNNRLNVQINERFQTITY
EDIFAIIDKLINLTITKDDIDHLKNRRVRSVGELLQNLLQIGFQRLIRKLRNQTNKFIVA
TVREFFGSSQLSQYLDQTNPLSSLTHRRRISGLGPGGFDRDRISFAVRDIHPSHYGRIC
PIETPEGQNVGLIASLTTCARVNKSGFLETPFWRVINGKVIKTGSPIYLTADIEDFYKIA
PADIAVNYLIKNLIPVRYKQDFVNVTPSEVDFIAISPVQVVSVAASLIPFFEHDDANRAL
MGSNMQRQSVPLIFPQKPIVGTGLENQISIDSGLTLNAQLAGTITSVTANKIVVTYRL
QKYLRSNQQTCINQRPIVWKGETVQSGQILTDGPAITSGELSLGQNVLVGYMPWQG
YNFEDAILVSERLVYDDVFTSIHIERYKIEIDRNSETSETKNIPNLLNHLNEDGIVKVGT
FVKPGDILVGKVVSSNTSEQLPESKLLRAIFVKDNSYKMPVIETVTFNRIFIAQIRKIQV
GDKIAGRHGNKGIISRILPRQDMPFLPDGTPVDILLNPLGVPSRMNVGQLYECLLGLA
GDKLNCRFKILPFDEMYGLEVSRILINKKLRQASKNESWLFNPYAPGKIVLIDGRTGIEF

ENPVTVGNAYMLKLIHLVDDKMHARATGPYSLITQQPLRGKAQHGGQRFGEMEVW
ALEGFGAAFTLKELLTIKSDDMQGRNETLNAIPKFGIPESFKVLLHELRSIGLDMSTYK
IEKFSEVEVNLI---

MGYINLIYPVTHVWYINSRPNFMALLLIKRIKGAPVIKKELEKLDLKAEIFRMRCFILVC
TKIREQSIKRIRILENLVGTNSNPAWMILTILPVIPPALRPMIQLEGGRFATSDLNELYR
RIITRNNRLLRLLEIDAPQLIIRNEKRLLQEAVDTLIDNGKRGKIALSANNRPLKSLSDII
KGKQGRFRQNLLGKRVDYSGRSVIVVGPSLKLNQCGLPYEMAIELFQPFIILINQGAKL
IQQNSIINPVLKILKNHPIFLNRAPTLHRLGIQAFEPILVHGRAIKLHPLVCSAFNADFD
GDQMAVHIPLSLEAQAECYMLMLAPYNFLSPANGEPIIMPSQDMVLGCYYLTVNNIK
GLLNSSHYFANLNDVILAYNQNKLEIHSVIWVRIIIVQYLQTTTGRAILNYIIQKTLMSR
YRGPKLKITRRLGLPGLTTKKSNKITGKKTEYGVRLEEKQKLKFNYGLSESQLFRYVKE
ARRRQGVTGLILLQLLEMRLDTLCFTLGFAKTMAQARQLVNHGHITVNKKVVNIPSF
QCRLNDIISVKEQTSSKVLVENNLIPAHLKFDQNKLEATVMDYCDRDDVILQLDELLV
IEHYSRMGQKTHPLGFRLGITQEHRSTWYANLTQYAEILKEDDAIRTYIHKIIANVNIN
RNDQIQLNIHTGKPILVLLNIKNLLRQLIINVLEVEKVDLSASLLADLVAEQLEKRVAFR
RAIREALQRAKNGIKIQVSGRLNGAEIARSEWTREGRVPLQTLRADIDYATQEANTIYG
VLGIKVWLFKMVNDTISDMLTRIRNANMVKHQIVQIPATRMSLGIAEILKVEGFIEDFE
AYSKKYILISLKYIGKSRQPVICKIERVSKPGLRVYSKSSNLPKVLDNLGIAIISTSKGLMT
NIKARELGVGGEVLCYIWMFVERLIKISRVSKVTKGGKKLSFRAIVVVGDENGQVGVG
VAKADDVVNAFKKAKTDGRKNLIKLPITKSLSIPHNVLGTFGASNVIMRPSIEGSGVIA
GGAVRIVLEVAGVKNVIAKQLGSDSLLNNARAAICALENLMSRRNISKKRFPQPDPVYS
SYLVSLLTTRILKSGKKTIARKIVYQAFDIIRKKTNEDPLSVFEKAIRNASPIVEVKARRV
GGSTYQVPIEVSSFRATNLSLRWIIQFSNKRVGRSMAIKLANEIIDTANDIGNTIKKKEE
THRMAEANKAFAHFRRMIRVRLESFNHELVVSSCQKIMNTLQNTPLHSAGVVTLPTR
KRIYCVLRSPHVNKDSREHFEIRIHKRIVEINYDLPIFDLLSDLPPGVFYRIMPTIQQLVR
SPRIQIKKKTKSPALVNCPQRRGVCTRVYTTTPKKPNSAIRKVARVRLTSGFEVTAYIP
GVGHNLQEHSVVLIRGGRVKDLPGVRYHIVRGALDTGGVKGRTQGRSKYGVKKPKM
KKDKKKFTSGVVHIKSTFNNTIVTITNLTGDTVSWASGGSSGFKGARKSTPFAAQTAA
EKAALEALNTGMKNVEILVKGQGSGRETAIRAIEGAGFDIISIQDITSVPHNGCRPPKR
RRVMVRLVGVDLPRNKKITYGLTYIHGIGLTSAKKIVELTNIDETRVNDLTPEQAGLL
RQTLEDLQLEGDLRRFNGLNIKRLNEINCHRGKRHRNNLPVRGQRTRTNARTRRGS
KKTKKMPVKQEIGIVISNKMEKTIVVKVENRYPHPIYSKTLVKTKKYLAHDELEECNIG
DQVLIQECRPLSKRKRSKLVMAKKSMIEREKKRIKLTKKYALKRVTLLEQYRTASSFSSK
IEIHSKIQKLPRNSAKTRIRNRCWKTGRPRGVFRDFGLSRHVIREMAHQCLLPGVTKSS
WMSRSLKKSPFIAYHLLKKINKAKKDTITTWSRSSTILPSMVGFTIAVXNGKQHVPIFXS

DQLVGHKLGEFVSTKNFRSHKADKKTKKMNKSAEKRIKTNERNRLRNRLYKSSVRTL
TKTFLKNLEIYAQNLLNSIYSLIDKGTKKNVFHKNTASRKKSQLAGYLKAMTQIFDESG
NIIPVTILKVGPCVVTQVKTKSKDGYDSIQVGYGNPKALTQPELGHLQKSDIQPLRYL
KEFRVSENEFNLGQILDVNSFSPGQLINIQGKSIGKGFSGLQKRYNFSRGPMTHGSKN
HRAPGSIGMGTSPGRVLPGKKMSGQVGNKVTTIKKLKVIQVNLEENILVIKGSVPGKP
GNLLSIVMAKNKGARILITLECIECRSNNKRSQGVSRYHTQKNRRNNPERLELKKYCP
HCNKPTIHKEIKMTQIFDESGNIIPVTILKVGPCVVTQVKTKSKDGYDSIQVGYGNPK
ALTQPELGHLQKSDIQPLRYLKEFRVSENEFNLGQILDVNSFSPGQLINIQGKSIGKGF
SGLQKRYNFSRGPMTHGSKNHRAPGSIGMGTSPGRVLPGKKMSGQVGNKVTTIKKL
KVIQVNLEENILVIKGSVPGKPGNLLSIVMNKTLEYNIIVLDGQLALDVSDNANYIIHRA
LEHQNVLSHQYTASTKTRSEVRGGGRKPWKQKGTGRARAGSNRSPLWKGGGVIFGP
KPKVVNHKLNKKEFQLALRTLLFNKRESLVVFDNFIECKTKEFLNLIRNLDSKTLIILSN
ANDPIQLAVRNIPTVETVLATSLNIKALLRAQQIFLDETTLNLIKEIYMLKLRLKRVGK
KRSPSYRLVIMENTFRRDGRPIDEVGYYSPITKQFKFDAEKIQKWLTYGARPTETVSTL
LKKIIMIVKYPIITDKATRLLENNQYSFIVDRYSDKTSIKSAIESLFDVKVIKINTCRLPRK
QKRKPQYKKAIVTLSEGDVINLFTMKIEKSDLPKLSIGDNVKIGIKIIEGNKERVQFYEG
TIIAKKNSSLNTTVTVRKIFQGIGVERVFLLHSPKIDSITVLRSSKVRRAKLYYLRNLKGK
AGRLKQIF
>Florenciella_parvula_MK518352
MIKVIYPKIEKIQINRGLGVNAQNNKVLQKSIDEIRQITGQQPVVTIATKSIAGFKVREE
MPLGITTTLRREKMYTFLDRFINLTLPRIRDFQGLDPNKFDKNGNYNLGITDQLIFPEI
DYEQVDQMLGLNITIVTSAKNSKEGLALLNKIGIPFMVRIKRGNVARKRRKKVLSLAK
GFRGTHSRLFRVSNQQVMKALRYSYIGRKQKKRQFRRLWITRINASTRTLTYSRFMNK
IKTSNIALNRKMLSQLSILDPQTFSKIQMVIRIYKPNTPGTRNRGVSSFNEITKNKPERS
LITANHRARGRNNQGLITIRHRGGGHKRRYRVIDFKRNKHNIAARVASIEYDPNRNAR
ISLLHYQDGEKRYILTAQNIKVGTTLMSGPDAEIQIGNSLPLENIPLGLEVHNVELIPN
NGGQIVRAAGTSAKILAKEGKYVTLRLPSKEIRLIRKECYATIGVVSNGEHSNITIGKAG
KKRWLGIRPTVRGVVMNPCDHPHGGGEGRSPIGRKRPITPWGKAALGVKTRKRRKS
YIVRRMSRIGKLPVSVPKNVNVELTGQEIKVKGPHGELLHTIPSEIEVEDKVIVNRIARQ
KYGLTRALINNMVIGVSEKFERRLQMIGVGYRSQVSGKTLTLSVGFSHPVVFEAPKDIE
IKVEANTNIIISGPDKEKVGLLASQIRAVRPPEPYKGKGIRYLDEYVLRKAGKSGKMTFV
NSQKTKKWYVIDAEDQILGRLATQASTVLMGKNKNTYTPFLENGDYVIIINAEKIKTK
DKQKLYRNHSGYPGEKFSSLIERRPEKIIEQAIKGMLPKGGRTIYKNLKVYKGTEHPHT
AQSPELIMAKKVTAIIKLALPAGKATPAPPVGPALGQHGLNIAGFCKEYNAKTGDKG
DLIVPVEISVYEDRSYSFILKTPPASVLLVKAAKIPKGAGEPNKVVVGSITTAQLEEIAKV
KLPDLNTNKIESAMRIVAGTARNMGITIMLSPKRTKFRKQHRGRMRGKAYRNNKISF

GEYALQSQECGWITSRQIEATRRTITRSIKRGGKLWITVFPDKPVTARAAESRMGSGK
GTVDYWVAVIKPGTILFELAGVPLDAAQTAMRLAAYKLPVKTKFITKMIQPQTYLTIA
DNTGARKIMCIRILGGNRKYGRIGDVIIGVVKDASPNMPIKRSDIVRAVVVRTRHTVRR
NDGMSIRFDDNAAVIINKENNPRGTRIFGPIAREIRDKRFTKIVSLAPEVLMYAIVEIGG
TQVWVQEGQYFVTNKISTGTNISLSRVLLINELHLGYPYLTQVTGEIVEHFKGTKLIVY
KMKSKKKYRRKKGHRQSMTKILIMAKAFGRYIRMSPNKVRRVLNQIRGKNYKDALML
LEFMPYRACGPVWQVIYSAAANAQNNLNIDKENLYISEAFADQGPVLRRFRPRAQGT
GYGIRKPTCHISITLMPKKDIHPNWFEETQVYCDGQLVMTTSSTKPNLYIDVWSGNH
PFYTGSLKILDTEGRVERFMKKYGSMAHKKGAGSTKNGRDSNACRLGVKVYGGAEV
VAGNIIVRQRGLTFKPGTNVKCGRDFTLYAIKSGKVEFQINIVMKVRPSVKKMCANCR
VIKRSGTIRVICTNPKHKQRQGMAVPKKRTSRSKKNSRKATWMQKGNVQATKAYSLA
KSVLKSTSFLYMFQYFKSSVETGHFLLQSLEAGQGLTIGNALRRVLLSNLEGTAITGIKI
PGVAHEFSTIPNVREDVLEIFLNLKQIVLKSEFGTVSINGPGVITASSIKFDEDIEIVNPN
QYIATFSGNDTLTFDLKVEKGIGYQFAFLAIDAVFMPVLKVNYKINNTTESLILEVTTN
GSVSPEKALSEAAQKLMTWFSCLILIEELQLPVRAYNCLKRAGINSVDDLVNYSQIREIF
GKKSAQEVFQALKDKNSLFNKKELKNVIYTAFIDYGTARASYLADDLKELGFHYATKA
GISISVEDLKVPPIKKSLLSEAYKEIETSDAQYHRGEINTIERFQNVINTWNNTSETLKD
ELVSYFKTTDPLNSIYLMAFSGARGNLSQVRQLVGMRGLMSDPQGQIIDIPIIHNFREG
LTITDYIMSAYGARKGVVDTALRTADSGYLTRRLIDVAQDVIIREEDCLTKRSINIISGRI
SASNVFLVKKNQEIDIEIANRLYELHVLGIRSPLTCESTRSICQKCYGWNLSGGKVVDL
GEAVGIIAAQSIGEPGTQLTMRTFHTGGIFTDPSRQIRSQKSGIVSFSKNIQTRPARTM
YGKNILLLEKETFLTFTQSSEIKLSADSSIFVKDNDFVKEKELLAELPLTNQQILSPVSGE
IVLNQIVWVLNGNIYDIPDEFKANDEITEDGLINFKIKSKNEGIVKLILINGGLLIPKEVK
AINKNLLEVGSVITELLKGTVSNLDGFVETIIQQITIRPGKIFFPGEIIFEDVIVEYLSYVE
LLRPIYEFNIRNIINSSISLLKISLHVNESQFIEPQTILGKISLLPQQFSITKIKKTSARLMLV
NKRNYETYYSEKDEMNNIKINYSGQILKVSPFACKLHRGIPFFLTKETVLYKTKNDLVK
VGDTIGTIIFEQAVTGDIVQGLPKVEEILEARKPQDNNTIGNAVGQPLTVSPHNLLTT
YFEYYEAAYLSFRKIQKLLINKVQDVYGSQGVTIADKHIEVIIRQITSKVEVTTAGSTLLL
PGEVVELRQINYINSIMQKTNYKPMLLGITKASLLTESFISAASFQETTKILTAAAIEGKI
DWLRGLKENVIIGRLIPAGTGMFVTTLPDFVEIQRASFCWFLSKGLSEELENFSSITDF
NSIELTFFGYDYKIRKPKYDIIEAKKRDSTFGVRIYVPMSISIFNINNRTNVSIGELPLMT
DRGTFIINGCERVVINQIVRSPGIYYLYSATLIPNRGSWLNFEIDKLTVSVDKTDKIEIAK
FFAALGIEEIYGSLTHPEFLKKQEFTRIFDPKYYNIGNIGRYKINQRFNLNIPKNVTDLT
HQDIISIIDHLINLEYQEDDIDHLQNRRVRSVGELLQTQFRSGLTRLERILSERMTITLV
SIMREFFGSSQLSQFLDQTNPLAELTHKRRISGLGPGGFSRDHVSFAVRDIHPSHYGRI
CPIETPEGQNAGLISSLSTYARVNSFGFIETPFFQVRDGVVLKNLPAIYLTAEEEDNLKI

APGDTKLNRIVSKSLAIRYKQEFVLVSSVEAELMAVSTVQIISAATALIPFLEHDDANRA
LMGSNMQRQAVPLLYPTKPIIGTGLENQLASDAGIVQINYSPGRVHKVSGSMIQIKYP
LQKYARSNQDTCINQRPIVWEGEYVESGQIIADGPGTEHGELALGQNILVAYMPWEG
YNYEDAILVSERLVYADLFTSVHIEKYDIEVRETKAGIETRDLPNVLKNLDENGIIFKGIF
VSPGDILVGKVTPSEESDEIPEGRLLRAIFVIDNSLRVPVLDIRVFTRVFLAQMRKIQVG
DKMAGRHGNKGIISRILPRQDMPYLPDGTPIDLILNPLGVPSRMNVGQLFEGLLGLA
GENLNKRFKIVPFDEMYSPEASRILTNSALKKASTKNDWLFNNYSPGRISLTDGRTGD
MFDNPITVCKTYMLKLVHLVDDKIHARSTGPYSLVTQQPLGGRSQQGGQRFGEMEV
WALEAFGAAYTLQELLTIKSDDMHGRNEALNAIPKPGIPESFKVLILELQSLGLDIGMY
KIQKDLGLEIDIMMFDYIKINLASSQRIKEWGERTLP-
NGIVGEVTKPETINYRTFKPEMDGLFCERVFGPVKNWECYCGKYKICERCGVEVTESR
VRRHRMGHIELVSSVTHVWYLKGIPSYLSLLLTNNLNGAEAIKYLLSKLDLKKEIKKNR
LLLTLIDDTRDKIIRRIRLIENFIATGSNPSWMVLDVIPVIPPGLRPMVQLEGGRFATSD
LNELYRRVITRNNRLARLLKIYAPAIIVHNEKRMLQEAVDALIDNGKRGRKALGANNR
ALKSLSDIIEGKHGRFRQNLLGKRVDYSGRSVIIVGPSLKLNQCGLPHEIALELFQPFIIL
IDNGAKLIQTGPIIWAILNVLEGHPILLNRAPTLHRLGIQAFEPILVEGRAIRLHPLVCP
AFNADFDGDQMAIHVPLSLEAQAEARMLMLAPNNFLSPATGEPILLPSQDMVLGSYY
LTVQTHLKDKFANHYFADNADALLAYEQEKIGLHTPIWLKNKNIKYISTTPGRILLND
VISKNLMSRYRGPRLRIIRRLGLPGFTSKTTERTKKSSSEYGIRLQEKQKLRYNYGLSER
QLFSYVKEARRLPGATGSILLQLLEMRLDSVVFRLGMAPTIPSARQMITHGGLKVNGK
KVSIPSFQCKPKDSIEAKSK-
SVKDMITNSMIPSHLEFDKDNLKGNVLSIINRDDVGLQINELLIVEFYSRMGQKVHPL
GFRLVTTQKHRSVWFSEFENYPNFLEEDSLIREYLNTKISRIEIKRNSKIEVEIFSARPVLV
IYNLRKIIKTVIVNIVELTTPDAEAALIGDFIVEQLEKRVAFRRAIRQAIKRSVAGIKVQIS
GRLNGAEMARSEWIREGRVPLHTLRANIDYADKRANTIYGVLGVKVWLFKMVNDTIS
DMLTRIRNANNAKHHFVEVPFTKMTSNIVTILKSEGFIQDYEMVKDKTILISLKYTGKR
RTPIITTLKRVSKPGVRVYIGAKNLPRILGNLGIAIISTSQGLMTDAEARKMKVGGEILC
YIWMWSERVIQISRVTKVCKGGKKLSFRAVVVIGNENGQVGLGVGKADDVVMAITKA
ITDARRSIIKIFLTKTKTLPHVITGRFGACEVFIKPASQGTGVIAGSSIRTVLELAGVKNIS
AKQLGSDNLLNNAHATLTALKNLMSRRSVIKKRYPQPDSNYNSCLVSLLTARILKSGK
KSLADSIILETFALIQEKTDSDPLQVFEKAIQNITPLVEVKARRIGGSTYQVPLEVSRYR
GTNLALRWLIAAAKARAGRTISIKLASEIIDASNNVGNAMRKREETHRMADANKAFAH
FRRMIRLKLESFDHKLLDLSCKQIIESINATQAKVVGPIPLPTKQRIYCVLRSPHVNKDS
REHFEIRIHKRILDIYPQNNQAQSIINLPSGVSTEIMPTVQQLIRKPRKKIVKKTKSPALK
NCPQRRGVCTRVYTTTPKKPNSAIRKVARVRLTSGFEVTAYIPGIGHNLQEHSVVLLR
GGRVKDLPGVRYHIVRGTLDAGGVKDRTQGRSKYGVKKPKMKKVKLNNITGVVHIQ

STFNNTIVTITDTIGNTISWASAGSAGFKGARKGTPFAAQTASEAASLTALNLGLKKV
DIFVKGQGSGRETAIRAVQNIGLEIISIKDITPVPHNGCRPPKKRRVMVRIAGIDLPRAK
RVEYALTKIYGIGVISSQEILAKANVDDARTNDLTDENVSSIRQVIEEYKVEGDLRRETS
LNIKRLSEINCNRGRRHRQMLPLRGQRTRTNARARRGPKKTKKMATKEKTGIVVSNK
MNKTIVVLVENRYAHPVYGKTLKTSKRFMAHDEFQECNLGDQVTISETRPISRNKRW
KVKMAKQCMLQREAKRQKLVKKYSKKREEILAGLKTSKSLTEIFEGQKKLQRLPVNSS
AIRLTNRCWKTGRGRGFYRDFGLSRHVLREMAHECLLPGVFKASWMGRSLRKGPFV
AYHLLKKVNEMKKLVIQTWSRASTIIPSMVGHTIAVYNGKQHIPIFISETIVGHKLGEFA
PTRTFRSHKSDKKAKRMLKSAIKRIDVTKRNTLENKLYSSNVKTFTKKYLASLDEYVT
QNLSLTFSKIDKATKKNIFHKNTAARKKSALTKAFSFMTQIFTEDGNANPVTIIKVGPC
VVTQIKTTSTDGYNSIQIGYKQRQKLTKPELGHLDKTSIVPLKYLKEYPIDNEDFTLG
QIINVQDFEEGQFVDVTGKSIGKGFAGTIKRHHYTRGPMTHGSKNHRAPGSIGQGTS
PGRVFPGKKMAGQLGAKQITVAKLKILKIDSKENLLIVKGAVPGKPGNLLSIKMAKSK
GSRIVITLECTECRSNNKRSNGVSRYTTKKNRKNTPERLEVKKFCRYCNTHTPHKEIK
MTQIFTEDGNANPVTIIKVGPCVVTQIKTTSTDGYNSIQIGYKQRQKLTKPELGHLDK
TSIVPLKYLKEYPIDNEDFTLGQIINVQDFEEGQFVDVTGKSIGKGFAGTIKRHHYTRG
PMTHGSKNHRAPGSIGQGTSPGRVFPGKKMAGQLGAKQITVAKLKILKIDSKENLLIV
KGAVPGKPGNLLSIKMEKTLTFPIKLTGNELKVKDAETNNYVIHRAYLTQRLNERQG
TASTLTRAEVRGGGRKPLRQKGMGRARAGSNRSPLWKGGGVSFGPKPKLYSNKINRK
EWKLGLRSLLISKQNNIIIVDNLITYKTKNIIKILINLSEKTLFILPKIEKNLLRSTNNIKTI
KLTIASNLNLKQILLAKNLLVTKDSLKIIEDTYMLKMRFKRCGRKRQPMYRIVVMQAT
SPRDGKAIRELGFYNPLTKETKLDVENIVKSLNTGVQPTKTVKNLLLKVIMILKYPVIS
DKTTRLLETNKYTFMVDRRANKVTIKKVIEYVFDVNVTNVNTLMTPRKKRTLSQYKK
AIVTLKDGDKINLFPMKVEKEDLPNVRVGDTVRLGVEIREGKKTRTQAYEGVIISQKN
SGINKTITVRRVMQGIGVERCFLLHSPKIKTIEIKRSSKVRRSKLYYLRELSGKATRLKQR
F
>Guillardia_theta_AF041468.1
MLKEIYLKITKITLNRGLGEASKNNKILEASIKEFELISGQHPLINKARKSVAGFKIREGM
PVGISVTLRKKLMYTFLEKLIHLSLPRIRDFRGVSVKSFDGRGNYNLGIKEQLIFPEIEY
DQVDQVRGLDISITTTAKTQQEGIALLRALGMPFMVRIKRGNIARKRHKKILKLAKGF
RGSHSKLFRIANQQVMKALRYGYHGRKRRKREFRSLWITRINAAVRITNYSCFINSLKR
QHIALNRKMLAQLAVSDQNAFKQLTMGIRIYKSYTPGTRNRSSSDFVEITKSKPEKSLL
RKKLSCAGRNNRGLITVRHKGGGHKQRYRLVDFKRNKLDIPAIVASVEYDPNRNARIA
LLHYQDGEKRYILHPKKLAVGDKIYSGINVPIEIGNAMPLYNVPLGTAVHNVELIPGR
GGQIVRSAGTSAQVVAKDGQVVTIKMPSNEVRMIYKNCYATIGEVGNADIKNIRLGK
AGRKRWLGIRPSVRGVVMNPCDHPHGGGEGRSPIGRAKPVTPWGKPALGVKTRRQ

NKSCIIRSMSRIGKLPVKFSEKVTMKIDQDNIIVKGPKGELALGLSKNINITNTLFVEPQ
VLKLFGTYRAIINNMVVGVTKGFEKRLELQGVGYRAQLQGKDLSLSVGYSHPVVIKAP
TGINIAVENNTIVIISGISKELVGQIASNIRSIKPPEPYKGKGIRYVGEFVRKKAGKAGKM
TIIREGNLGKWFIIDATDKNLGRLASETAKTLVGKYDPLYAPNVNPKNIIVIINADKIRV
KKFDKFYYRHSGQVGETFNELQSRLPGRILEKAVKGILPKGGRELFNNLKVYAGSSHP
HEAQNPVALMAKKVVAIIKLALEAGKATPAPPVGPALGQRGVNIVMFCKDYNARTAD
KAGLIIPVEITVYEDKSYTFVLKTPPASVLLAKAAGVQKGSGNPKKTQVGSVTKKQVE
EIAQTKLPDLNTRRLESAIRIIEGTAKNMGIGVMLSPKRTKFRKPHRGRLRGIATRGNT
LIFGDYGLQALEPIWLTSRQIEATRRTITRQVKRVGRLWIRVFPDKSISAKPPETRMGA
GKGAPEYWVAVIKPGHILFEINGVSQDLRYLAFKNASYKLPIKTKFISRMIQTQTYLTV
ADNSGAKKIMCIRILGGNRKYASIGDVIIGVVKDATPNMPVKRSDVVRAVIMRTKNTIR
RKDGMSIRFDDNAAVIINKENNPRGTRVFGPIARELRDKDFTKIVSLAPEVLMYAIIEAS
GKQFWVEPGRFYDFNSLNVGDKIALTRVLLINNITVGKPCLDVVEATVLGHLRGQKV
TVYKMQPKKKTRKKQGHRTNLTRLLIMAVPTAKYIRMSPSKIQRVLNQIRGKSYKESL
MILEFMPYAACKPVLQAVQSAGANAQHNKGINKNDLVVSLASVDNGPVLRRFRPRA
QGRGFKIQKFTSHIRIGVMPKQNLHPKWYAEAKVYCDGQLIMTIGSTKSELNVDIWS
GNHPFYTGSQKMLDTEGRVERFMRKYGLMAHKKGSGSTRNGRDSNAQRLGVKKYG
GEYVIAGNILIRQRGTNVKAGFNVGIGKDNTLYSLINGEVKFEISVYMKVVSSIKNRSK
DCQIVKRRGRIYVICTDPRLKVRQGMAVPKKRTSRSKTNSRFANWLNKSNLQAQRAIS
KAKSITNKKNTVNMIECLDSKRDWSRFVIDPLKPGQGTTLGNALRRTLLSELEGLAIT
AVRIAGVSHEFSTINGIREDVLEILLNLKEIVFSGQIGRLTVQGPAIVTARSFELPPEIKLI
DPEQYIATICGNNTLEMEFKIEVGSGYKIVFLQVDSVFMPVKKVNFRIEETNEQLILDI
WTNGSISAKEAISNAADNLINLFSPLILIEELQLSVRAYNCLKRAHIHSVADLLDYSQED
LIFGQKSAEEVIDALQKTNKVIDKKELKKLMAWAFSNYGTGRASFMADKIKDLGFHYA
TKAGLSLSVEDLRVPPIKRDLLLQANREIQQTEQLYERGEITTIERFQKVIDTWNNTSE
QLKDEVIKYFKQNDPLNPIYIMAFSGARGNISQVRQLVGMRGLMADPQGQIIDLPIKS
NFREGLTVTEYLISSYGARKGLVDTALRTADSGYLTRRLVDVAQDIIIREIDCGTQRGI
VLLIGRVLFETLYIGHINQDLDHDITNSIKCIKSVIVRSPLTCEAPRSVCQFCYGWNLAH
GSLVDLGEAVGIIAAQSIGEPGTQLTMRTFHTGGVFTELAEQIRAPFNGIIRYPKNLKI
RIIRTRHGNEGVILDENYKLNILNKTRLEFKQGTTLFISDNEEFKKGQIIGETKSRTRDL
VTETSGEVIYTNLLWILKGDVYNIPYNTDIQLKIKDTIISTFKTISEYNGIVRLLNLTGGI
IIPEETHEINKDLLLIKNNTTEIIKDVYCLNSGYVEIIVKEVIIKPGII----
KEVISTSTENKQIFLDFLRPVESYNILDLVKTYLIVVKTSLVIKDGQRIKKEDLIAETHLV
ARNKGILKSSQSTKKLLIMREEDTFRVPTNNATAGSVIASESGQVFEITKNEIILRKAYP
YLVSAGAILQIKDHELVQRNDTLAILVFERSKTGDIVQGLPRIEEILEARKPKEPCKLNV
LLAEPLTPNPHEMLNLYFNFYESAKLSLQKVQTYLVDEVQKVYQSQNVDISDKHIEVI

VRQMTSKVKVEDGGDTTLLPGELVELQQIENINEAMMLTTYCPILLGITKASLNTDSF
ISAASFQETTRVLTEAAIEGKADWLRGLKENVIIGRLIPAGTGMANRTLPDLVEIQRAS
FCWFLNEGLAEEIQSFSPIVNTGNLELHLFGDQYTLRYPKHNINECKRRDTTYSVQIY
VPAQLRETGVIKEQEVFIGDLPLMTDRGTFIINGAERVIVNQIVRSPGIYYTYSSSLISNR
GAWVKFETDRVWVRIDKTRKIPAHVFLKAMGLSDIYNGLRHPEYLKKTELSRFFDPKR
YDLGKVGRYKLNKKLNLSVPENVRVLTPQDTLAAIDYLINLKFETDDIDHLGNRRVRS
VGELLQNQVRIGLNRLERIIRERMTIKIIASIREFFGSSQLSQFMDQTNPLAELTHKRRIS
ALGPGGLNRDRAGFGVRDIHPSHYGRICPIETPEGPNAGLIGVLATHARINTYGFIEAP
FFKVQDGQVYNHSQPIYLTADQEDKYRIAPGDITLDRIATKIVPIKYRQEFTTTKPNQ
VDFIAVSPIQVISIATSLIPFLEHDDANRALMGSNMQRQAVPLLYPESPLVGTGLEAQA
ARDSGMVVVSIEDGQVTFVSGDKICVTYYLQKYQRSNQDTCINQRPTVWLGEDVIEG
QVIADGAATEGGELALGQNILVAYLPWEGYNYEDAFLINERLVYNDVYTSVHIEKYEIE
ARQTKLGSETRELPNVLRKLDENGIIVIGSWVEAGDILIGKVTPKGESDQPPEGKLLRA
IFVRDTSLRVPILDVRIFTRVYIAQSRKIQVGDKMAGRHGNKGIISRILPRQDMPYLPD
GTPVDLVLNPLGVPSRMNVGQIFECLLGLAAENLNKRFKITPFDEMHGAEASRVLVN
EKLNEAKTGENWLFDLRHPGKITLYDGRTGEAFDNPVTIGVSYMLKLVHLVDDKIHA
RSTGPYSLVTQQPLGGRAQHGGQRLGEMEVWALEAFGASYTLQELLTVKSDDMQG
RNETLNAIPRPGTPESFKVLMRELQSLGLDIGAYKIENLRGIEVDLMMFDYVKINLASP
QRIKKWGERRLP-
NGVVGEVTKPETINYRTLKPEMDGLFCERIFGPVKDWECHCGKYKVCERCGVEVAES
KVRRHRMGYIELAAPVTHVWYLKSLPSYISILLDIPLKGAEAIQKLLRDIDLEVEAESLR
EEILVVGIKKDKSIKRLRVIDNFIATRSDPTWMILTVLPVIPPDLRPMVQLDGGRFATS
DLNDLYRRVLNRNNRLIRLQEILAPEIIIRNEKRMLQESVDALIDNGRRGRTVMGANN
RPLKSLSDIIEGKQGRFRQNLLGKRVDYSGRSVIVVGPQLELNQCGLPREMALELFQP
FVILIQQGAKLIQKNLIVWNVLEVIQGHPILLNRAPTLHRLGIQAFEPILVEGRAIKLHP
LVCPAFNADFDGDQMAVHIPLSLEAQAEARMLMLAPYNFLSPATGDPIIMPSQDMVL
GCYYLTAENPSQQVNRSLYFSNFDDVLLAYETKLIKLHSYVWVRFLMVQYILTTPGRI
LLNNVLFDSLMSRYRGAVIKIIRRLGLPGLTRKTTTRTARKPSEYAIRLEEKQKLRFNY
GLTEKQLLQYVRTAKRIKGSTGEALLQLLEMRLDNVIFRLGMAPTIPAARQLVNHGHI
KVNNTRVSIPSYQCKAGDMISIRQHPKSQSIVKNYLMPNHLQIDKDNLTGKINGIIERD
WVA--
LNELLIVEYYSRMGQKVNPLGFRLRITSQHRSSWFATKESYPQLLEQDFKIRSYINREIS
KIEISRNNQLEVSVYTSRPGIIVTDILRLLISIRINVIELTNPDADANLIGEFIAQQLEKRV
AFRRATRQAIQKARQGIKVQVSGRLNGAEIARSEWVREGRVPLQTLRANIDYATKEAH
TTYGILGIKVWVFNMTNDTVSDMLTRVRNANLAKHQVVQVPATKMTKSIAHVLLEE
GFIESIEEVGNRQLLLSLKYKGREREPVINALKRISRPGLRVYANRKELPRVLGGLGIAVI

STSKGVLTDTKARTQGLGGEVLCYIWMWQERVIQVRRVTKVVKGGKKLSFRAIIILGN
ERGQVGVGVGKASDVIGAVKKAVTDGRKNLINIPLTNQNSIPHIVQGYSGAAKVIIKPS
APGSGVIAGGSVRTILELAGIKNILAKQLGSSNPLNNARAAANALINLMSRRSTTKKKL
ALPDPIYNSRLVNMLTVRILKEGKKHLAQRIIYNAFDIIKQRTGEDAILVFESAIKKVTP
LVEVKARRIGGSTYQVPMEVRAFRGTNLALRWITKYARERAGKSMSMKLANEIMDAA
NETGSSIRKREEIHRMAEANKAFAHYRRMIRIRLKGYETNLLLDSCNKIIETATRTNSVT
AGPIPLPTRRKIYCVLRSPHVNKDSREHFETRIHKRVIDIYEPNTTIDSLLDLPAGIDIDI
MPTIQQLIRSERQTI-
QKTKSPALKSCPQRRGVCTRVYTTTPKKPNSALRKVARVRLTSGFEVTAYIPGIGHNI
QEHSVVLLRGGRVKDLPGVRYHIIRGTLDAAGVKNRKQSRSKYGAKKPKMKKNKKVA
ANGVVHIQSTFNNTIVTLSTLEGDTVAWASSGAIGFKGAKKGTPFAAQIAAEKATKEA
ISQGMKKAEVLINGPGSGRETAIRALQAAGLEITLIRDITPVPHNGCRPPKKLRVMARI
SGIDLPKNKRIEIALTYIYGVGLSSSQKILEKANIDNKRCKDLDDNEVSLIRSIIENYPVE
GAAKRVESMNIKRLMEINCIRGKRHRVGLPLRGQRTRTNARTRRGAKKTKKMSIKER
LGLVISDKMDKTVVVSIANRVTHKRYGKIVTKTKKYKVHDPNNNCQVGDLILINETR
PLSKTKRWMFKMAKKSMIERERKREELVSKYEKKRLELKSKLKKTAEYEEKLEVYKKIQ
EIPRNAFPSRLRNRCWVTGRSRGYYRDFGLSRHVLREMVHDCLLPGVTKSSWMSRSL
SKGPYIAAHLLKKLNNVPDVVIKTWSRSSTILPNMVGATIAVYNGKQHVPVYISDQMV
GHKLGEFSPTRTFRSHKSDKKAKRMSASAIKRIELNERNRLRNKTYKSMLKTYYKKCIV
AIESNLRELVAITQSKIDKAVQKGIIHSNNGSAKKAKLTKRLKEMTQIFDDNGSAIPVTI
LKVGPCYVTNLKSDTKDNYNAIQIGYQQAKKLTKPQLGHLQVNNLPPLKHLKEYKV
DAHTFTIAQQLDVSIFELGQIVSVSGVSIGKGFAGTVKRHNFTRGPMTHGSKNHREP
GSIGQGSTPAKVHKGKKMAGRLGGHQVTTKNLTVVHLDKDNNVLVLKGCVPGKRG
NILSIKMAKGKGVRIVVTLEC---KTDQ----
GVYRYHTTKNRRNTPNRIELKKYCPLTQQHEIFKEIKMTQIFDDNGSAIPVTILKVGP
CYVTNLKSDTKDNYNAIQIGYQQAKKLTKPQLGHLQVNNLPPLKHLKEYKVDAHTF
TIAQQLDVSIFELGQIVSVSGVSIGKGFAGTVKRHNFTRGPMTHGSKNHREPGSIGQG
STPAKVHKGKKMAGRLGGHQVTTKNLTVVHLDKDNNVLVLKGCVPGKRGNILSIK
MQKFVTYNVLTNGQTLELNILTSGNYLIHKDITRQQIQQKQGTVSTKTRSEVRGGGR
KPWRQKGTGRARAGSNRSPLWKGGGVIFGPKPKTISKKLNKKERQLSLQTLLYNKRN
IISVIDNLFEPKTKAFIDFCIDLNQKVLIIVSQKTNGLKLSTRNVKNVELISASNLNTLSL
VKAKQILLTPLAVNEIKETYMLKLRLKKYGRKGQASYRLVVMPSTSKRDGRAIEELGFY
NPCTNETHINVERVKVRLSQGVKPTSTVKNLLDKVIMLVKYPLITDKATRLLELNQYT
FLTSRVATKTDIKNAIEFLFNVKVISINTCLLPLKRKRKPRYKKAVVTLEKNNTINLFSM
EHSNKTLPQLQVGDTVRLGVSITEGNKERIQATEGVIISKSNCGINLTITIRRVLQGIGI
ERVYLLHSPKLKSIEILRKSKIRRAKLYYLRTRSGKATRLQQRF

>Guinardia_striata_MG755796.1
MIKKEYPRLKKIQINRGLGLAAQNTNILKKSIEEFTKITGQKPIITKSKKAIAGFKIREDM
ELGLTVTLRGQKMYAFLTKLIFFTFAQIRDFRGLSVRSFDKAGNYTFGLKEQLIFPEIN
YEDVDQPQGFTITLVM--
SSPSNGMVLLKFLRFPLMVRVKRGNVARKRRKKILQLAKGYRGAHSRLFRVANQQVM
KALRYSYVGRKQKKRTFRRLWITRINAASKLSSYSRVIHNFKKSNIDLNRKMLSQIAVL
DSQTFTQLIMSIRLYKSYTPGTRNRALSSFDEITTATPEKKLIRANHRKKGRNNRGVITI
RHRGGGHKKKYRLIDFRRNKHNIEASVASIEYDPNRNARIALLHYEDGEKRYILHPNTL
NVGDSVVSGTEAPFAIGNCLPLEQIPLGTSIHNIELIPNRGGQIVRAAGTSAKILAKEGD
YVTLRLPSKEVRLIRKECFATIGEISFNDAFLVRSGKAGRTRWLGKRPTVRGSVMNPC
DHPHGGGEGRAPIGRSRPLTPWGKPALGIKTRKNKKSYIIRRMSRIGKLPIQIPENVEIS
CDGPEITVKGKFGTLQDTVPEILKVEGILVVGRNGRALHGLYRTLINNMVIGVSEKFTI
TLNLQGVGYRGAIQGTSLVLNLGYSHQVIIDIPEGISVEVVQNTKLNLSGCDKAALGL
FAAKIRSWRPPEPYKGKGIIYEGEVVKRKAGKSGKMTFIPSSDAKKWYIIDAAEKPLGR
VSTIIAAVLQGKHKVDYHPAMDTGDYVIVINAEAMHLSWAPKYRVYNPGRPGSSLKIF
FEKIPKRIVESAVKNMLPNGKQNFYKRLRVYSGSEHPHLAQNPIPLMPKKITALIKLAL
PAGKATPAPPVGPALGQHGVNIAAFCKEYNAKTGDQAGLIIPVEISVYEDRSYTFILKT
PPASVLLADAAQIKKGSSTPNRINVGSITKSQLEEIATIKLPDLNTTKLASAVKIVEGTA
RNMGISIMLSPKRTKYRKFHRGRMRGKATRGNEISFGDFALQALEPTWITSRQIEAAR
RTITRYTKRGASLWIRIFPDKTVTARAAESRMGSGKGAVDYWVATVKPGTIIFEMASV
PEEIARAAFKLASYKLPIKTKFIIRMIYPQTMLTVADNTGARKLMCIRVLGGNRKYAKV
GDTIIGVVKEALPNMPIKRSDVVRAVIVRTCKTIRRQDGMFIRFDDNAAVIVNLDNNP
RGTRVFGPVAREIRDKNFSKIVSLAPEVLMYAIVEISGRQFWVETGKYYDLNRIPTGQE
IILNRVLLLNELLVGKPYLDVIKGKVLEHLRGRKTIVYKMRPKKKTRKKQGHRQELTR
VLIMVKAVAKYIRMSPHKVRRVLNQIRGRSYQEALMILEFLPYGAGGPVWQVVHSAA
ANAQNNYDLDKKKLVIAEVYADEGPKLKRIRPRAQGRAYAILKPTCHITVVVMPKSEI
HPNWFPEAPVFYDGKLICYTGSTKPELNVDVWLPNHPFYTDSQTIIDTEGRVERFMK
KYGLMAHKKGAGSTKNGRDSNAKRLGVKRFGGQVVKAGNILVRQRGMKFKPGVNV
GCGKDFTLFALVDGTVKFDVNIIMKVRPSVKKMCDKCRIIKRHGKIMVICENAKHKQ
RQGMAVPKKRTSKAKKNARKANWKKKADKEAQKALSLAKSVLQTTSFIYMIKCLRSE
NEYGQFLINSLRAGQGITIGNLLRRTLLGDLGGTSITAVRIAGIRDEFSVITGVREDILEI
LLNLKGVVLKSKFGRLKVQGPAVITADLIQLPSEMELVNPNHYIATISTSNLLEIEFKFE
YGKGYKLAFLQMDAIFMPVQKVDFKIENITERLVLDIWTNGSITPEEAISEASQFIIDFF
QSIIAIEELQLSVRAYNCLKRAQINTIGDLLEYSPLQELFGRKSADEVFMTLKNQNTLV
SKKQLKQVLSWSFTKYGSIKACFLADELKYLGFKYATQAGISISIEDLRVPYVKSSMLQ
DANQEILNTEKICLKGKITDVERFQKIIDTWNITSELLKDEVVSYFKKYDPLNSVYIMAF

SGARGNLSQVRQLVGMRGLMSDPSGEIMNLPIKKNFREGLTITDYLMSGYGARKGIV
DTALKTANSGYLTRRLIDVAQDIIIREKDCLTNHSFLFILGRLLNKPVYIAEINTQITPN
LIRTFKRIENCYIRSPLTCSLYRSICQKCYGWDLACENLVDMGEAVGIIAGQSIGEPGT
QLTMRTFHTGGIFTEANQQLISPVNGTVQFSEVLKTSTLRTNRGENVLITESSGSLSII
TVIQLELPRNTILFINNNQFVKQDIIIGQLAGSIKQILSDSSGEIFMTQLLWILSGQVYQ
APNNFYQDYKINKSFIFRSKFVNEYPGIINDYLLHGGIVLPEETHTLNCDLLLIDSFIFEII
PNVYSQTSGVVKVIIQEIFLKPGNVFYPGELILNTIKIDQLSLCELIRPLEIYEVLNVVKN
TLNFIQSCLLVEPNQFINSHTILGYLEIVSKSLELVKFKSKSKQVFLISNNDCTVIQKEKN
KTEFLNINQTGKIIIQNEKFVTIQKGRPYFFQEVNSVFCKNGEFVKDGQTIGLLNFEKEI
TGDIVQGLPRIEELLEARKKKKNQKKIRKMGTTIKINPHSLLKIYFYYYEASYRSFKKIQ
ALILNSVQSVYQSQGVTIADKHLEVIIKQMTTKVLITHEGETPLLPREVIDLYHIKYINE
VVKTQCYVPLLLGITKAALNNPSFISAASFQETTRVLTKAAIEGRLDWLRGLKENIIIGH
LIPAGIGMYVTALPDFIEMQRVSFCWFISQGLTDELANFSSVLDFGNIEYIFFGQEYKLV
KPIYNASSAKRYTANYVAQLIMPIEMRNKRIMRQGRLPIANLPLMTTTATFIINGCERII
VSQVIRSPGVYFLYTATLIPEYGSWIRFGFLRVVIQIDKITKKPVIHLLREMGLKEISKNL
EHSDFFYFSEFSRIFDERYYRLGQIGRHKLNQKLNLKISHQLSTITYDDLFAIIDQLITLS
ITKDDIDHLKNRRVRSVGELLQQLFRIGFQRLSRKILSQVYKSVIATVKEFFGSSQLSQY
MDQTNPLASLTHKRRISGLGPGGFDRDRISFTVRDIHPSHYGRICPIETPEGQNVGLIA
SLTTCARINDSGFLETPFWRVINGKVLKTGKPIYLTADIEDFYKIAPADISTNYLTTSSIP
VRYKQDFITVSPSEVDFIAISTVQVVSAAASLIPFFEHDDANRALMGSNMQRQSVPLLI
PQRPIVGTGLENQIGADSGLTINAIKSGTVDFVSSKKIIIHYRLQKYQRSNQETCINQRP
IVWKGEKVESGQVLADGPSISGGELALGQNVTVAYMPWQGYNFEDAILINERLVYDD
VFTSIHIERYEIEIGLTNDGRETKHIPNIVQYLNEDGIISIGTFVKPGDILVGKVTPKDDS
EQLPEAKLLRAIFVRDSSFRMPVIRTIIFRRVFIAQIRKIQVGDKIAGRHGNKGIISRILPR
QDMPFLPDGTPVDIILNPLGVPSRMNVGQLYECLLGLAGQKLNRRFKILPFDELYGQ
EVSRILINKKLREASNNEAWLFNPYSPGKVVLIDGRTGKAFENPITVGNAYMLKLIHLV
DDKMHARATGPYSLVTQQPLGGKAQHGGQRFGEMEVWALEGFGAAYTLKELLTIK
SDDMQGRNDTLNAIPTSGIPESFKVLLQELRSIGLDISTYRLENFSELEVNLMMFDYIKI
KLASPTRIKQWGQRILP-
NGIIGEVQKSETINYRTFKPEMDGLFCERIFGPNKNLECACGKYKICDRCGVELTESRV
RRHRMGHINLVYPVTHIWYVNSRPNYMALLLIQRIKGTVIIKKELEKLNLNYEINDTRS
FINYCSKLRDQAIKRIRILENLLATGSNPAWMILSILPVLPPALRPMIQLEGGRFATSDL
NELYRRIITRNNRLLRLLEIDAPQLIIRNEKRMLQEAVDTLIDNGKRGKVALSANNRPL
KSLSDIIKGKHGRFRQNLLGKRVDYSGRSVIVVGPSLKLNQCGLPYEMATELFQPFIILI
NQGAKLIQQNPLIDPVLEVLANHPIFLNRAPTLHRLGIQAFEPILVQGRAIKLHPLVCS
AFNADFDGDQMAVHIPLSVEAQAECYMLMLAPYNFLSPANGEPIIMPSQDMVLGCY

YLTVNNIKNLLGSSHYFANLEDVILAYNQEQIELHSSIWVRYVIVKYLQTTTGRVIFNY
TIQKTLMSRYRGPRLRITRRLGLPGLTQKVSKKKKGKKTEYGLRLEEKQKLKFNYGIT
ESQLFRYVKEARRRKGVTGLILLQLLEMRLDTICFTLGFAPTMASARQLVNHGHITVN
GEVLNIPSFQCRIDDVIGIKPKSSSKNIVEENLTPSHLNFDKTKMEAKVLNYCSREDILL
ELDELLVIEYYSRMGQKTHPLGFRLGVTQEHKSSWYAKLNQYSNLLEEDDKIRTHLA
KLISNIKINRNDQIELNIETGRPALVLLNIKKILRQVTLNILEVEKVNLNASLIGDLVVKQ
LEDRVAFRRAIREAMQCAESGIKIQVSGRLNGAEIARSEWIREGRVPLQTLRADIDYAT
KEANTIYGVLGIKVWLFKMVTDTISDMLTRIRNANMVKHQIVQIPVTKMSLAIASILKE
EGYIEDFEQYKNKYLLISLKYKGKSREPVITTIKRVSKPGLRVYSGSKNLPQVLGDLGIA
VVSTSRGVMTNLKAKELGIGGEILCYIWMFVERLIKIGRVSKVTKGGKKLSFRAIVVVG
DENGQVGVGVAKADDVVNAFKKAKTDARKNLIQVPITKSLSIPHKVNGNFGACNIIM
RPSIEGSGVIAGGAVRTVLEVAGIKNVIAKQLGSDNLLNNARASIDALANLMSRRNISK
KRFPDPDPTYNSYLVSLLTKRILKSGKQTIAENIVNGAFEIIKAKTKEDPLMVFEKAIKN
ASPVVEVKARRIGGSTYQVPIEVNAYRATNLSLRWIIQYSRQRVGRTMAIKLASEIIDTA
NDIGNTIKKKEETHRMAEANKAFAHFRRMIRVRLESFNHELLMASCRKILNLTQNIEL
DNVGVVALPTDKRIYCVLRSPHVDKDSREHFELRVHKRVIEIYHDSDIFNLLSDLPSGV
LYRIMPTIQQLVRSRRIQIKKKTKSPALVDCPQRRGVCTRVYTTTPKKPNSAIRKVARV
RLTSGFEVTAYIPGIGHNLQEHSVVLIRGGRVKDLPGVRYHIIRGALDSGGVKDRTQR
RSKYGVKKPKMKKTKNNVTSGIVHIQSTFNNTIVTITNLTGDTIAWASAGSTGFKGAR
KGTPFAAQTAAEKASLDALSSGLKSVEILVKGQGSGRETAIRAIQGAGFEINSIQDITPV
PHNGCRPPKPRRVMVRLLGTDLPRNKRIEYALTYIHGIGLTSAKLIVKNAKIDQTRTD
DLTNEETIALRDELEDLKLEGDLRRFNGLNIKRLNEINCHRGKRHRNSLPVRGQRTRT
NARSRRGSKKTKKMPVKEKIGIVISDKMDKTVVIKVENRYPHPIYGKTVVKTRKYLVH
DENSECNIGDQILVQECRPLSRRKRWTLAMAKKSMVEREKKRIKLNNKYAAKRNLLL
QEYRSTNDFTLKLEIHSKIQKLPRNSAKNRIRNRCWKTGRPRGYYRDFGVSRHVLREM
AHQCLLPGVTKSSWMPRSLKKGPFVAYHLLKKIDNMKKSTIVTWSRSSTILPNMVGH
TIAVYNGKQHVPIFISDQLVGHKLGEFVSTRTFRSHKADKKTKRMNKSALKRIRIAKRN
RLQNKFYKSSVRTLIKMFFKRLEEYAQIILSSLYSLIDKGSKKKIFHKNTAARKKSQLALK
LKMMTQIFDETGNIIPVTVLKIGPCVITQVKTILNDGYNAIQIGYGNNKSLTQAELGH
LQKSNIQPLKYLKEFRVNNEEFEVGQILNVEAFTSGQLVNITGKSSGKGFSGLQKRHN
FARGPMTHGSKNHRLPGSIGMGTDPGRVLPGKKMAGQLGNKITNIKKLKIIQVNTEE
NILVVKGSVPGKPGNLLSIVMAKNKGTRILITLECTTCRSNNKRSAGVSRYITQKNRRN
TPERIELKKYCPNCNRTTIHKEIKMTQIFDETGNIIPVTVLKIGPCVITQVKTILNDGYN
AIQIGYGNNKSLTQAELGHLQKSNIQPLKYLKEFRVNNEEFEVGQILNVEAFTSGQLV
NITGKSSGKGFSGLQKRHNFARGPMTHGSKNHRLPGSIGMGTDPGRVLPGKKMAG
QLGNKITNIKKLKIIQVNTEENILVVKGSVPGKPGNLLSIVMNQLLKFPIIDGDSSLKLR

VQEKGNYLIHRAFVKQFIESRNGNANTKTRSEVRGGGRKPWQQKGTGRARAGSNRS
PLWKGGGVTFGPKTKTYKIKLNKKEWRLALQTLLFNKKNSIRVIKSFFEQKTKTLLNF
LNNIRNKILIVVPKKSLNLISTSQNLKNIDIIAADNLNIKSLLDSKYILLCTESLKIIEETYM
LKLRLKRIGRKRQPSYRLVVMTAETRRNGRAIEEVGYYSPITKESHFEAEKITRWLKNG
AQPTETVTNLLKKIIMNIKYPLITDKATRLLENNQYSFIVNPHSDKITIKAAIEYLFNVK
VIKVNTCHLPKKKRRKSHYKKAIVTLSEGDTINLFAMDLQKKDTPDLKIGDNVKIGVK
IVEGNKERVQFYEGTIIAKKNSSINTTITVRKVLQGIGIERVFLVHSPKVASIQVLRSSKV
RRAKLYYLRNLRGKASRLKQRF
>Heterosigma_akashiwo_strain_CCMP_452_EU168191.1
MLKEKYPKITKIQINRGLGAAASNNSTLQQTIEEIRIITGQQPIVTMAKKSIAGFKLREN
QPLGVTVTLRNTLMFSFLERLINLVLPRVRDFNGLNPNGFDQHGNYNFGLDNQLVF
PEISYESVDQQRGFNITIVTTAKTQVEGFNLLQSYGFPFMVRVKRGNVARKRRQKILIR
AKGFRGAHSKLFRTANQQVMKALRYSYIGRKQKKRQFRRLWITRINAAAKMIKYSVLI
NKLKNANVILNRKMLSQLAIVDPKTFSSLIMAIRIYRAYTPGTRNKVVSGFSEITQTKPE
KSLVNRVHRSKGRNNQGKITVRHRGGGHKRLYRNIDFKRNKFNIPAKVLSIEYDPNRN
ARIALICYKDGEKKYILHPQSLKIGDTIMAGKEASIQIGNALPLEYIPLGTEIHNIELHPQ
KGGQIVRCAGTSARILAKESDYITLRLPSKEIRLVHKSCYATIGQLSNSDHANIKLGKAG
RKRWLGRRPAVRGSVMNPCDHPHGGGEGRCPIGRARPVTPWGKPALGMKTRKNKK
SFIIRRMS-
LRRKRITIPPKVNVTFNKSILKFEGPKGNLELKIPNLVLIDGFINVNKKSKSLAGLICKEV
ANSLTGVLNNIEYRLQLRGVGYRAQIKGKQLELSLGYSHPNIIEISPELNVSVQANTEIII
EGVNKNFVGQLAAEIRRLRPPEPYKGKGVFYKGETILLKPGKSGKMTFVPSKTRKKWY
VIDANEKTLGRLATEVASFLRGKYKPYFTPYLDTGDYIIVINADKINVKKFEKLYRHHS
GRPGETFEKLQSRIPERIVETAVKGMLPKGGRAIFKKLYVYKGNLHPHEAQKPEKIMP
KKLTAIVKLALPAGKATPAPPVGPALGQHGVNIAGFCKEYNARTSDQNGLIIPVEISVF
EDRSYSFILKTPPASVLLARAANISKGASEPNRKKVGSITQSQLEEIAKIKLPDLNTTKID
SAMLIVEGTAKNMGISIMLLPKRTKFRKQHRGRLKGKACRGNKISYGDYALQALEPT
WLTSRQIEATRRTITRYTKRGGKLWITVFPDKPVTFRAAESRMGSGKGAVEYWVAVV
KPGKILFEMSGVPLELAKAAMKTASYKLPVKTKFITRMIQPQTLLTVADNTGAKKVM
CIRVLGGNRSYASIGDIIIGVVKDAIPNMATKRSDIVRAVVVRTKKTIRRKDGLSIRFDD
NAAVIINNDNNPKGTRIFGPIAREIRDRDFMKIVSLAAEVVMYAIIEASGRQFWIEPRKY
YTMNHVPLGTKVILNNILLVNEMQLGFPYLNVIEGIILNHFDGQKVLVYKMKPKKKY
KRKNGHRQKLTKLYIMAKAIAKYIRMSPTKVRRVLKQIQGLSYKEALMILEFMPYRAC
GPVWQVLHSAASNAQNNLGLSKQNLFIKSAFVNQGPTLKRFRPRSKGRGFKILKPTC
HINIIVMPKKNIHPQWFTDTKVYCDGQLVMTTSSTKPELKVDIWSGNHPFYTGTQRII
DSEGRVERFFKKYSLMAHKKGAGSTKNGRDSNSKRLGVKCFQNHKVSKGSIIIRQRGL

RVKAGENIGIGKDFTLYALEAGLVKFKVSIEMKVRPSVRKMCDKCRVIRRYRKVMVVC
VNPKHKQRQGMAVPKKRTSKAKKNARKSKWYNTANRAAKQAISLAKSTLKSTSFII-
IEILESDQEYGKFLINSLHPGQGTTVGSALRRVLLTDLPGLAIVGLRIANINTEFSSLPG
VREDILEIILNVKDIVIEGELGRLKLKGPGVVTAALIEFEEDIQIINPSQYIATIEVNSSIEM
EFLVQTGKNYRLVFLQIDAIFMPVTKVAFEVTSDNENLTLEIWTNGSISPIKALVFSASI
LENLATQLMPIEELILSVRAYNGLKRANIHSVSDLVQYSILKDIFGKKSLDEVVASLKNR
NKVVTKKYLKEILFWAFRNFGMARAGFLSDSLKKLGFYYATQGGVSISIEDLKIPPLKT
EIIDRTKKQVNNSELNCIRGELTEVERYQQLTNQWNTSSEVLKNKVVYYLKNFDPLNP
VYMMAFSGARGNLSQVRQLVGIRGLMSGPTGDIIDIPITTNFREGLSVTDYMISAYGA
RKGLVDTALKTADSGYLTRRLVDVAQDILIRETDCKTKKGILLLIGRILAKDIFFAYRN
QQISSLLAKRIYNFNKVLVRSPITCELSRSVCQTCYGWNLAYGKLVDLGEAVGVVAAQ
SIGEPGTQLTMRTFHTGGVFTESNGQIFSKISGQIRFPANLKACTGRTEQGDKVLIVE
NDTKIEIVFSIPIFVKKESMLFVKKDQFIKKDQIIAILAKTKQNVFSSVGGEILLLDVFWIL
SGKVLNIPYNKYKFENLKPSNLATIKLITSKTGFLTNFVLQGGILLPEETIRVNRRVRKV
GDWITQISGSIYNRKAGFVEFIVSKVIIKIGNVFFPGEKLLDEHVINQLTYTQLLQPVFL
YEFIQLVKRKLVLIKISTLFRNNQFIESNTIIGDINIAPKLTTIKQIKERKRKLFLVSTKDY
LNIFIEQKKFDSILSCYSGKILSNTNKNILLHKGKPFLFSKGAEIFWKNNEFIKSDDSLGV
LYYQRAQTGDIVQGLPKVEEILEARREKKIMTSTRFPGKPLNASPHTSLKYLNFYYEAA
YRSLRKSQSYLLNSIQSVYSSQGVDISDKHLEIIVKQIGSKVRVNTIGQSNIIPGEYIYLQ
HINKINTVLEKTDYYPVLLGITKASLVTESFISAASFQETVRVLADAAIQGKADWLRGL
KENVIIGRLIPAGTGMFITKLPDFLEIQRSSFCWFLLKGLSYELNSLSPIIDVKRVKLKLY
PQEFVLKPGRTTPICAKQNDSTYGVRIFLPAEVPTKKEDIKEKVFIGQIPLMTSTGSFIV
NGCERVIVNQIIRCPGLYYVSTVTIIAQRGSWLKFEFDKYWVRIDKEKKISIFDFLEGLNI
NEILSGLKSIAPLLK---
EKITDPNSYSLGIIGRLNLNRRLGLNISTRVHTLTIHDIFGIIDFF--
LSARSDDIDDLRNRRIRAVGELITSQCEIGLNRLERNILERTNFKLVSAIQEFFNSSQLSH
YMDQTNLLSETANKRRISALGPGGLNADRVTVAARDIHPTQYGRLCPIETPEGQNVG
LVSTLASYARINRNGFIQTPYFRVENGKILTQQPLIYLTAEQEENLKIAPADVKRGYLV
DDFIVTRFNQEFIITPSKLVDFISVSVIQIISVAASLIPFLEHDDANRALMGANMQRQAV
PLLYPRKPIVGTGIESQVAFDSRLVNIATKPGIVKYVSSQQIDIKYKLIKYRPSNQDTCL
NQRPLVWVGQSIKTGQVIADGPGTQSGELALGQNLTVAYMPWQGYNYEDAILVSDK
LVYQNLFTSIHIEECETEVQQTKTGEQTSDIPLVCKNLDENGIIKVGKYVYPGDILVGK
ITPKGEIDQLPEAKLLKAIFMRDSSLRVPVLDIKIFKKVFIAQISKLQVGDKIAGRHGNK
GVISRILPHQDMPFLPDGTSVDIILNPLGVPSRMNVGQIFECLLGLAGDQLNKRFKILP
FDEMYQNEASRILINQKLKDAAQNKPWLFSAYSPGKILLSDGRTGEKFDNPVLVGRSY
ILKLAHLVEDKIHARSTGPYSLITQQPVGGKSQNGGQRFGEMEVWALEAFGAAYTLQ

ELLTIKSDDMQGRDDVLNSIPKSSIPESFKVLMRELNALGLDITTYKVMFEEKNEINLM
MFDFIKINLASPEKIEIWAKRLLP-
VGVVGEITKTDTINYRTFKPETGGLFCEQVFGPIKTGACSCGAYKVCSVCGVEITDSR
VRRHRMGYINLLAGVSHVWYLKGRPSYLSLVLRERITGAEIIKTKLEQLSLKSEIVKSRT
NL--
NNRKRKRAIRRIRVLESFLLTKSKPFWMILSNLSVLPPGLRPMIQLEGGRFATSDLNEL
YRKIIMRNNRLGRLFNIDAPNVVIRNEKRLLQEAVDSLIDNGRRGQKIVDINNRPFKSL
ADSIAGKQGRFRQNLLGKRVDYSGRSVITVGPELKLHQCGLPYEIAVELFKPFLILIEQ
DAQIIAHDKLIKKLLNILRNQPVILNRAPTLHRLSVQTFQPILVTGSAILLHPLVCPPFN
ADFDGDQMAVHIPLSSKAKAEAISLLFAPCNFLSPATGSPVLLPSQDMLLGCYYLTTN
NLKGLKGANHYFSNFDDALLAYENKLVDLHALIWIRYIITTYIRTTPGRLIFNKVINQA
LMSRYRGPRVKILRRLGLSGLTQNESIRKGKQKGPYNLRLLEKQKLKYNYGVTEKQLL
NYVKKARRIKGATGLQLLQMLEMRLDTVAFRLGWGNSIPASRQLVNHGHILVNDRK
VNIPSFICKPGDKLTLKEKATNKQIISAN-
YPKFLELDKSKINGIVKEIVSREDFTISVDELLIVEYYSRMGHKTHPMGFRVGITKTHQS
SWFSNLKEYSKLLEEDFKLRESLLKFISKILINRNTQIQLEIHTAFPIIVLNFFEKTFKKIII
NLVEITEPYREAGLIGDYIVQQLEKRTQFKRVVKKAISLSSEGIKVQISGRLNGAEIARSE
WVRQGRVPLQTLRADIDYSYKTAQTTYGILGVKIWLFKMVNDTISDFITRIRNAILVR
HKIVQTPATKLTIAITKILKTEGFIEDFEIFKKNYLLIFLKYKDGGKKPSIKFLKRISKPGR
RIYVNNKNLPDTIGSFGIAIMSTSSGIMTDKKARVKNTGGEVLLYVYMINHKVVQIKRV
SKVVKGGKKMSFRAVVVVGGRTGFVGIGTGKADDVTNAINKAVAKGKRNLINIPITK
TETIPHSVTGIFGASNVIIRPSAPGSGVIAGSSIRTVLEVAGIRNVLAKQLGSSNLLNNAK
ATINGLNQLMSRRNISRKNFPSADPVYNSLLVSLLTARILKSGKKTLARRIIKDVFDLIE
NRTSLDPLVVFEKAVRNVTPIVEVKACRVGGSTYQVPIEVSGFRGTNLSLRWIVKAARS
RSGKTFSMKLANELIDASNLTGSAIRKKEETHKMAEANKAFAHFRRMIRVKIHAFDVK
TLNACCEKIIVAVKDTNILLKGPIPLPTKRRIYCVLRSPHVDKDSREHFEIKIHKKFLDIY
TVEDAASFLNQMPTGAFFEIMPTIQQLVRNRRQVIKQKTKSPALQGCPQRRGVCTRV
YTTTPKKPNSAIRKVARVRLTSGFEVTAYIPGIGHNLQEHSVVLVRGGRVKDLPGVRY
HIIRGALDSVGVKDRFQGRSKYGVKKPTMKKVKKNVVVGVVHIQASFNNTIVTITDL
GGNTLSTGSAGAVGFKGARKGTPFAAQLASEKAAEKATEYGLKKVEVLVKGQGSGRE
TAVRAIQNSDIEITAITDITSIPFNGCRPPKRRRVMVRIAGVDLPSQKKILYSLTAVYGIG
LHTAKKILEKAKVDEIRTFELDDDSVGNIRSVLENYTVEGDLRKLKSLNINRLVSINCFR
GKRHRQGLPLRGQRTRTNARTRRGSKKTKKMPIKERIGTVVSNKMDKTIVVAVESRF
KSPIYSKFKIRTKRYLAHDEFNKCNIGDQVLVSESRPLSRRKRWVLKMARKCMLERELK
RQKLVKKYKKKKEQLFKEFYTTEDFDKKLIVHGKIQKIPRNAAQNRLRNRCWRTGRP
RGFYRDFGLSRNMLREMGHQGLLPGVKKSSWMSRSLKKGPFVAYHLLKKVQKMKKE

IITTWSRSSTIIPPMLGHTIAVYNGKQHVPVFINEQVIGHKLGEFVPTRTFRSHKSDRKS
KRMIKSAQKRVLIAKRNEIRNRKYKSMVKTYTKKYFVLIENHITHAEGVVFSKIDKAVK
KNIFHKNTAARKKSQISMVIKKMTQIFDENGLAVPVTVVQSGPCIVTQIKNFSTDGYN
AIQIGFLAIKNTNKAEQGHFTKVGCQPQRILKEFKVESENFTLGQQIKVDCFEIGQFV
NVTGRSIGKGFSGLQKRYNFSRGPMTHGSKNHRLPGSIGAGTTPGRVLPGKKMAGQ
YGNKNVTISKLKVLNIDSKENLLLLKGAIPGKAGNIVSIAMAKNKGSRIIIVLECKECRT
NTQQAPGVSRYSTTKNRRNNSGRLELKKYCRYCNKHVFHKETKMTQIFDENGLAVP
VTVVQSGPCIVTQIKNFSTDGYNAIQIGFLAIKNTNKAEQGHFTKVGCQPQRILKEFK
VESENFTLGQQIKVDCFEIGQFVNVTGRSIGKGFSGLQKRYNFSRGPMTHGSKNHRL
PGSIGAGTTPGRVLPGKKMAGQYGNKNVTISKLKVLNIDSKENLLLLKGAIPGKAGNI
VSIAMQKSIKYTSFILGEQLELNVIESGNYLIHRNLLRHYSLQHQCTASTKTRGEVRGG
GRKPWRQKGTGRARAGSNRSPLWKGGGVIFGPKYRKKKLKINKKERRLALQTLLYNK
KNSSFILHELFAIKTKKFTEVLLKLNKKILVIVSEKTKFLKLSSKNLKNIELISASNLNIFSL
LKAKQIVLTTSALNIIKETYMLKLRLKKFGRKKLPSYRVVLMESCSRRDGKPIREVGFY
NPINNQIKLNTDEILTSLKNGAKPTKTVFNLLVKMLMAIKYPIITEKSTSLFEKNQYTF
VVDRNLDKEIIKKTIELLFNVKVTKINTSVLPKKKRRKPNYKKAIVKLASGDTIQLFPM
KVQKTDIPNLEVGDTVRLGLLIKEGNKERTQYYEGVTISKKNSGINKTITVRRIMQGIG
IERLFLIHSPKIASIEIKKSANVKRSKLYYLRNRSGKATRLKQKY
>Leptocylindrus_danicus_KC509524.1
MLKVLYPKLVKIQLNRGLGLAAQNSNILKKSIEEFSSITGQKPIVTKAKKANAGFKIRED
MDLGIKVTLRGEKMYTFLDKLINITLPQVRDFKGISSKSFDRDGNFSFAVKEQLIFPEL
SFEDVEQIRGTEITIVTTGNTKDASKALLSELGFPFMVRVKRGNVARKRRKKILHLAKG
YRGTHSRLFRVANQQVMKALRYSYIGRKQKKRIFRKLWITRINSASKLSKYSKVIHNFK
TSNILLNRKMLAHIALVDSSTFSTLIMSIRLYKSYTPGTRNRALSSFDEITTNKPEKSLIR
KNHRSKGRNNRGVITIRHRGGGHKRKYRLIDFRRNKYNVEATVASIEYDPNRNARIAL
LNYSDGEKRYILHPNTLTVGSKILSGTTIPASVGNSLPLSEIPLGTSVHNIELIPNRGGQI
VRAAGTSAKILAKEGNYITLRLPSKEIRLIRKECFATVGEISNSDAFLVQSGKAGRTRWL
GKRPTVRGSVMNPCDHPHGGGEGRAPIGRSRPLTPWGKVALGVKTRQTKKSYILRR
MSRIGKLPIKIPTDVDVNFNGADITVKGKFGTLENTIPNGIKIDEKLVLTRTNRALHGL
YRSLINNMVIGVSEQFKLTLNLQGVGYRAAVQGKDLVLNLGYSHQVIMPIPEGISSEV
VKNTTINLTGCNKADLGLFASKIRAWRPPEPYKGKGIIYEGEIVKRKAGKSGKMTYIPS
SNEKKWYLIDGDNKTLGRVATEIATILIGKHKVDYHPAVDNGDYVIVTNAKNIQLKE
DEKIYFSHSGRPGETVKALRARLPERIIEKAVKGMLPKGGREMFRRLKVFSDSTHVHT
AQNPQLFMAKKITALIKLALPAGKATPAPPVGPALGQHGVNIAAFCKEYNAKTAEKS
GLIIPVEISVYEDRSYTFILKTPPASVLLTNAAKIKKGSSTPPKTMVGSVTKAQLEEIATI
KLPDLNTTKLSSAIKIVEGTARNMGISIMLSPKRTKYRKFHRGRMRGKATRGNTVVFG

DYGLQALEPTWITSRQIEATRRTITRYTKRGGKLWIKIFPDKTVTARAAESRMGSGKG
AVDYWVAVVKPGTVIFELAGVSQEIARAAMNLAAYKLPIKTKFIIKMIYPQTLLTVADN
TGAKKIMCIRVLGGSRKYAKIGDTIIGVVKEATPNMPIKRSDVVRAVVVRTRKTIRRPD
GMFIRFDDNAAVIVNMDSNPRGTRIFGPVAREIRDKNFSKIVSLAPEVLMYAVLELSGR
QFWVEEGRHYEINRIPNGKEIILNRVLLVKELRIGKPYVEAVTAKVMKHLLGKKTVVY
KMRPKKKTRKKQGHRQKITRISIMVKAVAKYVRMSPQKVRRVLNQIRGKSYQEALMM
LEFLPYKASGPIWQVLHSAASNAKNNYDIDKKTLVIAEGYADEGPRLKRIRPRAQGRA
YKILKPTCHITIVVMPKSEIHPTWYPDAKVFCDGKVLFTTGSTVPEINVDVWSGNHPF
YTGSQTIIDSEGRVDRFMKKYGLMAHKKGAGSTKNGRDSNAKRLGVKRFGGQVVKA
GSILVRQRGMVFKPGVNVGYGKDFTLFALVDGTVKFDVNIIMKVRPSVKKMCDKCR
VIKRHGKIMVICPNPKHKQRQGMAVPKKRTSKAKKNARKATWKRKADKAAQKAFSL
ANSVLNKTSFIYMIKCLKSKKEYGQFLINSLNLGQGVTIGNLLRRILLSEVGGCAITAVR
IAGTTHEFSTLPGIREDILEILLNLKGIVLKSKFGRLKIKGPAVITADLIELPPNLEIINPN
HYIATISDANFLEIEFKFEYGTGYKLAFLEIDAVFMPVQKVDFKIENVTERLILEIWTNG
SISPATTIINASQFIINLFSSIIPIEELQLPVRAYNCLKRAQINTVGQLLKYSPLQEIFGRKS
ANEVFAALKNQNTLISKKQLKNLLAWCFTKYGAVQASLLADELKYLGFKYATQAGISI
SIEDLRVPPAKLSILTEAENEISNAEELALRGKTTEVERFQKVISTWTLTSETLKDEVVS
YFKTYDPLNSVYMMAFSGARGNISQVHQLVGMRGLMSDPSGEIIDLPIVKNFREGLRI
TDYLMSGYGARKGIVDTALKTANSGYLTRRLIDVAHDIIIREKDCNTEYSILFILGRLLS
KNVYIAKVNDQITPNLINLFQKIQVIFFRSPLTCNLNRSVCQKCYGWNLANENLVDLG
EAVGIIAGQSIGEPGTQLTMRTFHTGGIFTGGNKPIISPGDGVVKFSDVLKTSILRTSN
GENVLITENSGQIFLIDKVSIYLPSNSTLFITENQYIKQNAVIGQFVENTKQIVSDFSGEI
KITTLIWLLAGQVYNIPNNFYDDYIINKNYLFRIKLINRYAGFVNNYLLSGGIVLAEETFI
LNRDLLLVNEFIYEIVPNIFSKSMGIVKIIIQEVIIKPGKIFYSGESIHQTILINEPVFCEEIR
PLQLFEVQKIISETLQFINLCLLVENEQFINAYTAFGYLEICTKYKKLVQLKSKNKQLLV
ISEDDCIVVPKDKSNFNLTSTLKIGKYLTETDHSILLQKGRPYFFIEQNTIYCKNGTFIE
EGQPLGLLNFEKEVTGDIVQGLPRIEQLLEARKIESSPREFLNMMTTFKLNPHFLLNL
YFEQYEGTYRSFKKVQDLILSSVQSVYRSQGVTISDKHLEIILKQMTAKVKIFKEGDTP
LLPLEIIDLYQINYMNKIIFQSLYRPILFGITKTALNSPSFISAASFQDTTRVLTRAAIEGR
VDWLRGLKENVVIGHLIPAGTGMYITTLPDFVEIQRKSFCWFLSQGLSTEFETFGSIVD
MDNLEICFFNQEYILVKPTYSALESKHYDTSYSARIQMYLQVRDKKIILNRKIFLGYLPL
MTDAATFIINGCERVIVSQIIRSPGIYFLFNATLIPDRGSWVKFELLEIVIDINRNVKQSII
ALLQTMGLSEIYNNLQSPNYIYTPEFSKIFDERYYFLGKIGRYKINKRLNLKISEQFQTL
TYQDLFSIIDYLLELSKSNDDIDHLKNRKVRSVGELIQNQFRIGLQRLKRKGQETLVFS
LLTTLKEFFNSSQLSQYMDQTNPLSALTHKRRISSLGPGGLNRDRISFAVRDIHPSQYG
RICPIETPEGQNAGLVSSLTTCARINDLGFLVTPFWRVINGKVLKKGDPIYLTADEEDL

YKVAPADIFIDYLVKDLIPVRYKQNFITIPPSEVDFIAISSIQVVSAGAALIPFLEHDDANR
ALMGSNMQRQAVPLLSPQKPIVGTGIESQIAIDSNMGITAINDGYVDYVSGDKIVIQY
NLQKYNRSNQETCINQRPIVWPGEKIESGQMIADGPGIDSGELALGQNILVAYMPWQ
GYNFEDAILINERLIYDDVFTSIHIEKYEIDIIQTKEGSETKNIPNIVQQLDENGVIYKGSF
VKPGDILVGKVTPKGDSEQLPEAKLLRAIFVRDSSLRMPVIATEVFTRIFIAQVRKIQVG
DKIAGRHGNKGIISRILPRQDMPFLPDGTPIDVILNPLGVPSRMNVGQLYECLLGLAG
DNLNRRFKISPFDEMYGQEVSRILINQKLRKAAKNKAWIFNPYTPGKMVLVDGRTGK
EFDNPITVGKAYMLKLIHLVDDKIHARSTGPYSLVTQQPLGGKSQNGGQRFGEMEV
WALEGFGAAYTLQELLTIKSDDVQGRNNTLNAIKKSGLPESFKVLLQELRSIGLDVTT
YKLNKLTDIEINLMMFDYIKITLASPKHIKEWSQRLLP-
NGIVGEVTKAETINYRTFKPEMDGLFCERIFGPNKNLECHCGKYKICDRCGVEVTESR
VRRYRMGYINLIYPVTHIWYMKSRPNYMALLLIRQIKGAAAIQKELELLDVETEITKTR
EFITHVIYLLDKATKRIRILESFSSTGSHPSWLILSLLPVIPPGLRPMIQLEGGRFATSDL
NELYRRIITRNNRLIRLLEIDAPQLIIRNEKRMLQEAVDSLIYNGKRGKTAMGANNRPL
KSLSDIIKGKQGRFRLNLLGKRVDYSGRSVIVVGPGLKLNQCGLPFEIAIELFQPFIILIN
QGAKFIQQNILIEPVLEVFLNHPVFLNRAPTLHRLGIQSFEPILVPGRAIHLHPLVCSAF
NADFDGDQMAVHVPLSIQAQAESYILMLAPYNFLSPATGEPIILPSQDMVLGCYYLTA
NNISNLLGSNHYFANFEDILLAYNQEKVELHASIWVRYTIVQYLQTTTGRVIFNYTIQK
TLMSRYRGAKLRITRKLGLPGLTNKTSKKDNKKKTEYGLRLEEKQKLKFNYGLTESQ
LYRYIKEARRRQGVTGLILLQLLEMRLDTICFNLGFGNTIANARQIVNHGHVTVNGEV
VNIASFQCRIDDKISFKPTTVSKNLITENVLPTHLKFDSEKVEGQVTNHCSREDLMLEL
NELLVIEYYSRMGQKTHPLGFRLGITQEHKSAWYTNLKNYADLLKEDDQIRTYLTSLI
SNIHISRNDQIKLTLETGRPVLVLLNLKQLLRQLTIKVVEVDEINLDASLLGDLVVKQL
EDRIPFRRAVREAMQRAENGIKIQVSGRLNGAEIARSEWFREGRVPLQTLRADIDYAT
KEANTIYGILGIKVWIFKMVTDTISDMLTRIRNANMVKHQIVQIPTTKMSLGIASILKEE
GFIEDFESYSKKFILISLKYKGKEREPVICKIQRISKPGLRVYSNADKLPKVLNNLGIAIIST
SQGVMTNLNAKKLGIGGEVLCYIWMLVERLIKISRVSKVTKGGKKLSFRAIVVIGDEN
GQVGVGVAKADDVVNAFKKAKTDGIKNLIKVPITKSLSIPHTVHGNFGACKIIMRPSIE
GSGVIAGGAVRTVLEVAGIKNVIAKQLGSNNLLNNARAAVCALDNLMSRRNIAKKRFP
EPDSVYNSYLVSLLIARILKSGKKNLAQNIVNGAFEIIQKKTDQDPLLIFEKAIKNASPIV
EVKARRVGGSTYQVPIEVGAFRATNLALRWLLTYARQRGGRSMSIKLANEIIDTANEIG
NTIKKKEETHRMAEANKAFAHFRRMIRVRLESFDRQLLLDSCQKISSIVQKVDPKKITN
IPLPTNKRIYCVLRSPHVDKDSREHFEIRIHRRVLEIYPDSNIFDLLVDLPPGVFYRIMPT
IEQLVRSPRIQIRKKTKSPALVSCPQRRGVCTRVYTTTPKKPNSAIRKVARVRLTSGFEV
TAYIPGIGHNLQEHSVVLIRGGRVKDLPGVRYHIIRGALDSGGVKDRTQGRSKYGVKK
PKMKKEKKNFTAGVVHIQSTFNNTIVTISNITGDTISAASAGSVGFKGARKGTPFAAQ

MAAEKAASEAFSVGLKNVEILVKGQGSGRETAIRSIQSAGFEINSIQDITPVPHNGCRPP
KRRRVMIRLKGVDLPNRKRISYALTSIYGIGINSAKELVKAANIDATRTQDLTIEETVAL
KEVLEELKLEGDLRRFNGLNIKRLNEINCFRGQRHRNSLPTRGQRTRTNARSRRGAKK
TKKMPVKEKVGIVISNNMQKTVVIKVENRYSHPIYSKTVVKTKKYLVHDEINTCNIGD
QILVQESRPLSRRKRWTLVMAKKSMIERETKRIRLTKKYAEKRKTLLEEYRSAKEFKTK
LEIHSKIQKLPRNSAKVRIRNRCWKTGRPRGYYRDFGVSRHVLREMAHQCLLPGVTKS
SWMTRSVKKGPFVAYHLLKKIDAMKKDTIKTWSRASTILPMMVGHTIAVYNGRQHV
PVFISDQLVGHKLGELVTTRTFRSHKTDRKAKRMNKSALKRIKIAERNRLQNRYYKAS
ARTLIKLFVKQLETYAQTLLNSIYSLLDKGCKKKVYHRNTAARKKAQLAAQLKNMTQI
FNESGNVVPVTIVKVGPCVVTQIKTVLSDGYNAIQIGYLNNKVLTQPELGHLQKSNIQ
PLKYLKEFRVNNEDFTVGQIITTSSFNEGQLIDITGKSSGKGFSGLQKRHNFARGPMT
HGSKNHRAPGSIGMGTHPGRVLPGKRMAGQLGNKTVNIKKLKIIQVNTEENILVIKG
SVPGKPGNLLQIKMAKTKGTRILITLECTECRSNNKRSAGVSRYTTQKNRRNNPERIE
LKKYCPHCNKSTIHKEIKMTQIFNESGNVVPVTIVKVGPCVVTQIKTVLSDGYNAIQIG
YLNNKVLTQPELGHLQKSNIQPLKYLKEFRVNNEDFTVGQIITTSSFNEGQLIDITGKS
SGKGFSGLQKRHNFARGPMTHGSKNHRAPGSIGMGTHPGRVLPGKRMAGQLGNKT
VNIKKLKIIQVNTEENILVIKGSVPGKPGNLLQIKMQKILTFPVKLTGEELKLKKAHTIN
YVIQRAYLTQRSNERQGTANTLTRAEVKGGGRKPWRQKGTGRARAGSNRSPLWKG
GGVSFGPKPKLYSSKLNRKEWQLSLRSLLSAKQKDITIIDNFFLYKTSKIIKILINLFENT
LIIVPKIEEKLIKSTRNIKTIKLIVANNLNLKQVLLAKNLLITKDSLKTIEETYMLKLRLTR
IGRKRQPVYRLVVMQNTTRRDGRPIQLLGYYNPITKQYNFNSDKIKYWLKQGVQPTE
TVKSLLRMIKMIIKYPLITDKTTRLLENNQYSFIVNTQSDKTTIKQSIEYLFNVKVIKITT
SNLPRKKKRKPQYKKAIVTLSEGDTINLFAMNLQKENIPDIAIGDLVEIGVKIIEGNKER
VQYYQGNVIAKKNTSINTTITVRKVLQGIGVERIFLIHSPKVDSISILRSARVRRSKLYYL
RNLKGKASRLKQRF
>Mallomonas_splendens_strain_CCMP1782_MH795131
MLKTHYPKLEKIQLSAGLGLNAQNRVFLQKAIEEFRLITGQHPILTSARKSIAGFKIREG
MPLGLTVTLRSEKMYSFLEKLICLVFPRIRDFRGLSPTNFDKHGNYNFGISEQLVFPEI
DYDSVEQRRGFNITIVTTATNSREALFFLKELGFPFMVRVKRGNIASKRRKKYLHLAKG
FVGVNSKLATMAGEQVVQSLNFAYIGRKLKKRNFRRIWIYRINAASRANIYSIFIGCLRN
INIFLDRKILSLIAFHDLSSFNLIEMGIKIYKPYTPSTRNKTVLDFSNLSKVKPERSLIVSN
HRAKGRNNQGHITIRHKGGGHKRRYRIIDFKRNKFNIEGTVISIEYDPNRNSNIALICYT
DGEKKYILHPEKLNIGDKIFSGAQSKIQVGNALPLENIPLGTDIHNIELFPGKGGQLVR
SAGTSAKIMAKENNFVVLRLSSKEIRLFKKECFATIGKVSNSDFYNVSLGKAGRKRWLG
IRPTVRGSVMNPCDHPHGGGEGRSPIGKPRPLTPWGKPALGFKTRKKEKVYLLSKMS
RIGKKPIVIPKDVSITLVNRELIVQGKFGTLKRIVSENINIPGKLIITKNSRESHGLNRALI

QNMVDGVEKKFSKTLIAEGVGYRFQVEKNFLILNVGFTHPVQFQIPEDLSIKVESNTKI
QLFGIDKEKVGFFAAKIRNMRPPEPYKGKGILYEGEKIIRKAGKTGKMTLFPKQEIPKW
YVIDATSKTLGRLATEVSKLLRGKEKSYFTPGINQGNFVIILNAQKIQVKRSDKFYYRNS
TRPGETFDQLQKRIPSRIIEQAVWGCYQKGGRSYYRRLYIYSQNYVPSFSTEEKKKMAK
KIKAFVKLALPAGKATPAPPVGPALGQHGVNIAAFCKEYNAKTSEKVGLVIPVKITIYE
DKSYTFILKSPPASVLLTKFANIKKELHNQIKNKVEQLLLNKSKK-------------------------
------

MLSPKRTKYRKQQRGRLKGKACRNNKLDCGDYGIQALEPVWLTSRQIEAVRRTISRY
TKRTGKLWVKVFPDKPITARAEESRMGSGKGAVDYWVVVIRPGNILFEVTGVPKEIA
MEALKIACYKLPIKTRILTKMIQPQTYLTIADNTGAKKLMCIRVLGNNRKYANVGDIII
GVVKEALPNMAVKRSNVVRAVIVRTKNTIRRTDGMAIRFDDNAAVIVNVENNPKGTR
VFGPVAREIREKNFTKIISLASEVVMYAIAQTSGKQFLLKPGQWYDVDYIQVGDYIYIK
KVLFFREIQLGKPFLGSIPAKIIQQVKGKKITVLKTKPKKNYTRVRGHRQPYTRVQIMV
SASSKYIRISPNKINVIIMKIRGKSYKEALQILKYLPQRAGAIVWQTLYSAVSNAIHNYNF
QKENLIIQEAYVNQGPILKRMRPRARGRGFAIEKKISHLTIRVM-KKNLHPQWY-
KTKVFCDGKLILELGTTKEEIFVDIWSGNHPFYTGSQKIIDTEGRVEKFQRKYNLMAH
KMGAGSTRNTRESQSKRLGVKCTGSQQVKSGSILVRQRGTKFKPGRKVGCGRDHTL
YALVDGRVNFTVNILMKVRPSVKKMCENCRIIKRHGRIMVICLNPKHKQRQG----------
----

LTCIETYINYGCFVIEPLEAGQGITLGNALRRTLLSDLTGFSIVGVRINNLKHEFASIPGL
REDILEILLNLKEIIFKTSKGFLQVKGPRILTASMFQLPRNLKIVNPNQYIGTLVDTSELY
IEIDIENGKGYKLSTLFIDALFMPIRRVNYKTKLIKESLILEIWTNGSLTPKRSLQEALKIL
INLFYPL--
LTQTVTKKLLETLVHETFTQFGNFSSSSLLDSLKLLGFYYATIAGISINIEDLKTPDTKK
KFLENANFEIEKISKDWQQGFISDTERFQTIIDIWNIATESLKNRIIDYYQHFDPANNLY
IMAFSGARGNISQVRQLVGMRGLMSDQEGKIIDLPIQTNFREGLSSIDYIISSYGARKGI
VDTALKTADSGYLTRRLIYIAQDLVIRELNCGTTEGIIILIGRTLIYAKSLQDLNLSLKEV
DLIKLKSPVQLNVRSSLTCKSNGSICQKCYGWDLAQQKLISLGEAVGILAAQSIGEPGT
QLTMRTFHTGGIFTETTKQIFAPFSGKLILPSSLKTVSYRTNHGILVLKLQQESNLIILK
QEEIFLNIGSYLYISTTQFVKKGQVIAEYSLRSFLIYTSLAGEIKFEDVLWIASGKIFPLPE
KYLFPNTLNKKAFAKLKIISPYSGFLTGLIL--

IKIDFDNLVKFKFSFITKNSQYIDSYTVLGFLELFSDEGKIYSLRKKITTFFIVIEADVWKI
NSDQKTIKILHLSRSGFFLKKDGFKMIFQHAIPIFLTRGTILKYKQGDFVFEKKVLATLI
NYTQQTEDIVQGLPKIEELIEARKPKLSQETKFEVGKK--KKKHKLFGL-----------------
----------------------------GKVFI---

MFVNNIGDLSEAQRASFYRFLINGINEELTNFPNPFLAIKIFVSLYPNEIKLKGPNFSLNT
CLRLDITYAIQLFIKGEYKRNKVQVIQDIFFGEIPLMTEEGTFVISGCERIVISQIIRSPGIY
FIYTATIISNKGLWTKFILDRIYIKLMDYNKLFINDLIRYFGLREIYDSVKHPQRVRDGIF
QLFFNSGCFSIGEIGRYNINKKLGLNLPSNITYLTSHDFIGIIDGLMELKYYDEDIDHIK
NKQIRSIGELLQNQLRIGLYRLQKSLIEEIPFPPVNSIKEFFKTSQLSQYMDQINPLAELT
HKRRISVFGPNGLKRDHISTVIRDIHPSQYGRLCPIETSEGQNAGLIMSMALYGRINSL
GSIETPYFFLENGTFNSKKRTIFLNPEQESETKIAFGDVSLRKINNQYLSIKENYLFSVKK
PQEINFLTISPLQVVSLATSLIPFLEHDDANRALMGSNMQRQAVPLLYPQKAIVGTGL
ESIAILDSGMIIKSYCQGKVVFSSSLSIIVTYYLRKYYRSNQETSLNQRPLVWSGEEIFSG
QLIAEGPSTNDGELSLGRNLTIAYMPWEGYNYEDAIVINERILLDDCLTSVHIEEQETT
FTSSLLGPETNNLPHLRRHLNSDGIVRIGSYVKEHDILVGKLTSC-
EADLSPEARLLNALYFRDTSLKVPVIDVRISSTIYIAQIRKVKIGDKLSGRHGNKGIVSRI
LARQDMPYLPDGTPIDVIFNPLGVPSRMNVGQIFEGLLGFAGEKLGKRYKIFPFDEVY
GKEASRILVNQKLKEASLNSTWIFNPSYPGKILLKDGRTGEFFDNPITVGKSYILKLIHL
VEDKIHARATGPYSMITEQPLAGKSQKGGQRFGEMEVWAIEAYGCSNTLQELLTIKS
DDIDGRNDMYEAIPNPSIPESFSALIRELNALGLDFSLYKFENHPQKEKDLFMFDYLRIS
LASPNRIRSWAERILP-
NGIIGEVLKPETINFRTHQPEINGLFCEIIFGPIKNWKCKCGKYNICEECQVEIIEARVRR
YRMGYIDLTCPVTHLWYLKGVPNYLNILLSKDIKGAEIIKAALENLNLKTEIQNARSLV
ENTCIPEKSLIRRIRILESFLATKTNPSWMVLTVLPVLPPNLRPLLELESGRLVVADVNE
IYRLIITRNQRLFDFMHYMAPDIITVHGRRLLQEGVDSLIDNARLAKKKLALNNKALKS
LTEILEGKQGRFRQSLLGKRVDYSGRSVIIVGPNLRLNQCGIPYEMAIELFQPFLILLKT
KAQIIKKNPFVWSLLNLTKKYSVLLNRAPTLHRFGIQAFDPILILGQAIHLHPLVCTGF
NADFDGDQMAIHLPLYECSQLEARTMMRPSYNVLSPSNGEVILKPTQDMVIGSYYLT
LMINKNSFPIEKWFSNVQEALSAYYSKKITLHTRILVRYFVISFLETTPGRLIFSLNFQNS
LMARYTGPKIRIIRRLGLPGLTRKNIKNRRSSLDDYRERLYEKQKLRFNYGITEKQLYSY
YQQAKREPGSTGNLLLQLLESRLDCVIHRLGFAPTIPSARQIVNHGHIFVNGKLVNIPS
FLCQKEDIITVRDRKQSKNLIKVNFLPTHLKVDNENLIGQFICPVKRKDVLVRVNELK
VVEYYSRMGQKTHPKGFRLVTTEKHLSNWYGTKFYYSDLIKEDYYIRKKTQEFISKVEI
NRVEYVNIQIKALFPLLNLLTIRNFTKNYYIKIDFIKNPFEDATLIAKFISEQLEKRTPFRR
AVKQTIKKVRKGIKIQLSGRLNGIDIARSEWKREGRVPLHTLKAKIDYSHESAQTIYGVI
GIKVWLFA--------
MLTRIRNSCLARHSLSRVYYSKLNIAILKVLQAEGYIHSYTIESHLFIKIYLKYKGWIKKPL
FSNLQRISKPGKRVFSGYKNFQKFIDVLGIAIISTSSGVMSHIKASQLKKGGEILCFIGMY
EEKIIQIKRVTKVVKGGKKLTFRAVVIIGDNKRKVGVGIGRADDVNMAIDKAILHGKK

NLINVPLTLTYSVPHVIKVSSGACSVMLRPASQGTGVIAGGSIRTVLELAGIRNILAKQI
GSNNILNNAKTTILALNLLMSRRISTKKRFPERDSKYDNLLVSLLVNRILKNGKKRLAK
RIVYKAFEFVEYRTNQSPILILEKAIRNVSPRVQLKAKRVGGATYQVPTLLSKFRSTNIA
VRWIVEFSRKRSGKGMSLKLANELLEAAKGLGNAIKRKEETHKMAEANKAFIQSRRMF
RILLKSFDNDLIDFACNQLRSVLLKTESKVAGVVSLPIKIKKFCVLRSPHIDKDSREHFE
LRFYKRFIDLETSSLVIELLLELPAGVSCGLMPTIQQLIRFNRTKVINFTKTPGLKSCPQ
RRGICTRVYTTTPKKPNSALRKVARVKLTSGFEITAYIPGEGHSLQEHSIVLVRGGRVK
DLPGVRYKIIRGALDCTGVKDRQQGRSKYGARKPIMKKTKKSVTIGIANIKTTFNNTII
TISDFLGNTLCWASSGSSGFKGARKNTPFAAQTAARNAALKALEFGMEKIEIVVKGRG
NGRETSIRALKSAGLNILSIEDKTSVPHNGCRPPKKRRLMVRILGNNIPNKKKIYVALT
CIYGIGIPTSLKLLKKLKINELKVSELKEENISALRDSLEEFKLEGDLKRLISLNIKRLIDIN
SFRGRRHLKGLPVRGQRTRTNNRTSRRQSLFRKMVKKERIGVVVSNKPEKTIVVAIQM
CYQHRKYGKTLIQTKRYMAHDEENACKSGDLVLVEESSPFSRQKKWKLKMAKKSML
QREVKRKKLIKKYLQKRTNILENLRTQTSLENIFLLNEKLQKLPRNSSPIRLRNRCWKT
GKPRGYFRFFGLCRNALRELAHDCFLPGVTKSSWMTRSILKGPFIAYHLLNKLNDLKK
ETIKTWSRSSTILPLMIGHTISIYNGQKHVPIFITDQLVGHKLGEFVPTRTFRSHKTERK
VKRMKQRNRKIVTQNRRNKLINRRYSSTIKTLFKLFIQKTKNFLKKIVSNLYSMIDKAV
KKGVIHKNTAARKKSKIGQIYVKMTQIFTPKGDRIPVTVIKGGPCYVTQIKSKENCGY
NAIQLGYFESKNITKPNLGHFNKVNLPPFRYLKEYKTLMSDYEIGQKFSVEMFKIGEKV
NISGLTIGKGFTGNIKRHNFERGAITHGSKHHRAQGSLGSGTTPGRVFPGKRMSGHS
GVEKRTVLGLEIIDIDINENIIVVKGCIPGKSGNLVNITMAKNKGPRIVVTLECIECRKN
NKRSIGVSRYFSSKNRRNTPDKLELSKHCHYCNKHTIHKETKMTQIFTPKGDRIPVTVI
KGGPCYVTQIKSKENCGYNAIQLGYFESKNITKPNLGHFNKVNLPPFRYLKEYKTLMS
DYEIGQKFSVEMFKIGEKVNISGLTIGKGFTGNIKRHNFERGAITHGSKHHRAQGSLGS
GTTPGRVFPGKRMSGHSGVEKRTVLGLEIIDIDINENIIVVKGCIPGKSGNLVNITMKK
FLTYKVQLMSGVLELKCANTSNYLIHRALTQELTNKRSFTASTKTRSEVRGGGRKPW
KQKGTGRARAGSIRSPLWRGGGVIFGPKPKQVKLKLNRKEKNLAITTLLVNKYDQTF
VFENTNSLGTKQYVQLLIDMKRKTLVILSKDNDTFGTALDNIPTIDINAVDNLSVKSL
LNAEQIIFDSDLVGYLQFLYMLKIRLKKLGRKNKPFYRIVLMENLSKRDGKSIVELGYY
DPISKSLNIDKISLHKYINCGAYPTDTIRHLIYKMIMLIKYPCLTEKAINLYGNSQYTFIV
DKSLTKIQIKFIIQKLFNVNILSVNTCNLPTKTRRRSKYKKAYISLKEGEKIDLFNMQIEI
DKKLKINVGDIIRIGYLIPEGEKERTQYYEGLIIAIKNKGIGKSFLLRRTVQGIGIEQIFVL
NSPKILSIVKKQSSKVRRSKLYFLRNLRGKSAKLKVKL
>Dictyocha_speculumprot_MK561359
MTRDLYPKIEKIVINRGLGVNASNNKLLQKSIEEFRLITGQQPIVTIAKNSIAGFKVREQ
MSLGLKVTLRKEKMYTFLDRFINLTLPRIRDFQGLDTTKFDSDGNYNLGIKDQLIFPE

IEYEQVDQILGLNISIVTTAKSIEESKSLLTQIGLPFMVRIKRGNIARKRRKKVLKLAKGF
KGAHSKLFRVANQQVMKALKYSYVGRKQKKRVFRRLWITRINAITRRKPYNKFMHEIK
KENIGLNRKILSGLAILDPVTFDKINMVIRIYKPNTPGTRNRGVLVFSEITKNKPEKSLIR
VNFRKKGRNNRGIITIRHRGGGHKKRYRLVDFKRNKKGIPAQVQSIEYDPYRNARIAL
VTYEDGEKSYVLHPQTLKVGSVIMSGKKVEITVGNTLPLSSIPLGLDVHNIELVPNKG
GQIVRAAGTSARILAKEGSYVTLRLPSKEVRLIRQECNATIGIVGNGDSNKIKIGKAGRK
RWLGIRPTVRGIAMNPCDHPHGGGEGRSPIGRKRPITPWGKAALGVKTRSKNNSYIIR
RMSRIGKLLINIPSEVNVELQDQKIIVKGRHGELTHQIPMEILVESSIHITRIARQKHGLT
RSLINNMIIGVSEKFNRQLQMVGVGYRSQVSGNTLTLNVGYSHPVIFVSPPGVEFKVE
ANTNIFVSGINKEKVGLLASQIRSIRPPEPYKGKGIRYMDEKVLRKAGKSGKMTFISTQ
DTAKWYQIDATDHVLGRLSTEVSKLLIGKNKVNYTPFLESNNYVIITNAEKIKIKETQK
LYRRHSGRPGRTYKELKEKRPEAVIEKAIKGMLPKGGRKMFTNLKVYEGSIHPHLAQE
PQLIMAKKIVAIIKLALPAGKATPAPPVGPALGQHGANISAFCKEYNAKTGDKGDLIIP
VEISVYEDRSYTFILKTPPASVLLIKAANIQKGSGEPNKEIVGSVTMNQVEEIAKTKLVD
LNTSNLTAAVKIVAGTAVNMGIAIMLSPKRTKFRKPHRGKMRGKASRNNTISFGDYAL
QAQECAWITSRQIEATRRTITRTVKRGGKLWITIFPDKSVTARAAESRMGSGKGTPDY
WVAVVKPGTILFELSGVPLELAQKALSLAAFKLPIKTKILIKMIQPQTYLTVADNTGAK
NILCIRVLGNNKQYGNIGDIIIGVVKDASPNMGVKRSDIVRAVIVRTRHTLRRKDGMSI
RFDDNAAVIINKENNPRGSRIFGPIAREIRDANFTKIVSLAPEVLMYAIVEIGGTQVWV
QTGQHFITNKVPLGTSILLKRILMIKQLMLGFPYLNVITGTVIEHLTGPKLIIYKMKSK
KKYRRKKGHRQQLSKILVMVSASGKYLRMSPFKVRRVLNQIRGKRYKDVLMLLEFMP
YRACGPVWQVVYSAASNAQHNFNIDKEFLYVKEAYADQGPVLKRVRPRAQGQGFAIK
KPTCHISIVLMPKKEIHPKWFDETQVYCDGQLIMTTSSTRPKLTVDIWSGNHPFYNGS
LKILDTEGRVDKFYKKYGN---

MKVRPSVKKMCDNCRVIKRHGKIRVICTNPKHKQKQGMAVPKKRTSRSKKNSRKAT
WYNKANVKAKKAYSLACSVLKSPSFIYMFQFFNLEIETGHFLLQALDKNQSITIGNALR
RVLLSNIEGTAITGLKIENVLHEFSILPSLREDILELMLNLKQVILKKGYGTIDIQGPGIIT
ANAIKFSEEVDIINPNQYLATLTSDEWLKIELKGETGFGYSFSFLPIDAAFMPVLSVNFN
TKKNEESLILEITTNGSISPESALSEASNKLMSWFSKLILIEELQLSVRAYNCLKRKGINSI
EELVTYTKICEIFGKKSAQEVFQSLKDKNLLFSKKEIKDLVYLAFSNYGITKTTHLVDD
LKDLGFSYATKAGISISIEDLKVPPTKQDFLRSAFKEIDYSNSQYERGEINSIERLQKVIE
TWNNSGETLKESLIDYFRESDPLNSIFLMAFSGARGNVSQVRQLVGMRGLMSDSKGQ
IIDIPIINNFREGLTITDYIMSSYGARKGVVDTALRTADSGYLTRRLIDVAQDVIVREEE
CFSKSFVTILLGRTIAQDVYLIKQNQELTTPLLTKLFAAFNFKIRSPLTCMSIHSICRKCY
GWNLAYCKLVDLGEAIGITAAQSIGEPGTQLTMRTFHTGGIFTDSSRQVRARCPGVIS

LSREVTLKPTRTSYGKNLLLTGQSTTLFITKLDKIDISANTLIFFENGGFVKKNQLLAEL
PILNQQIVSKFSGEIFRYSILWILFCNVYDIPKHISNLTKTSSQALLNFKIIADESGLVNYL
LLDDGILIPQHITIVNRDLIIVCNFFVEIATDIYSRISGFVEIILRNIIIKPGNIFFPGEVVFD
DVKVDFPSFVQLLRPIYQFNLLPLFQSSLFLYQFSFFMKNLQYVEVGTILAKISLTIKQH
EILKFKYTRKRLSLVTTSHCKTYYSDFSTSDNLKVTHSGQILAINPFVSKIHKAMPLYLT
DKTRSFIESGTLILKGQVLGVISFNQVVTGDIIQGLPKIEEILEARKAKSNYRLGTPFGQ
QLSVDFHGLLEFWFTYHVAACLSFRKIQTLLVTKVQEVYSSQGVSIADKHIEVIIRQVT
SKVQVDNPGDTFLLPEESLDLKQINYINEVLSLHTYTPIILGITKASLLTESFISAASFQQ
TIRVLTKAAIEGKIDWLRGLKENVIIGQLIPAGTGMFITTLPDFIEIQRASFCWFLSKGL
SEELANFSVIEDLNSITLNFFGNDFRIQKPLLDIVETKARDRTFAVRIYVPVLISKVKNK
DKIYTFIGEIPLMTDRGTFVVNGCERVIVNQIVRSPGIYYC-
SATLIPFRGGWLGFEIDHGTVSFNKIQKVDLAKFLTLIGIKKIHSYFLHPEFFDCGFNSQ
IFDSKYFNLGHVGRFKLNQQLDLNIPITTNELTKQDLVIIIDGLINLFYGEDDIDHLKN
RRVRSVGELLQTQFRSGLMRFERILSERMTFSLVSMMREFFGSSQLSQFLDQTNPLAE
LTHKRRLSGLGPGGFNRDHVSLAVRDIHSSHYGRICPIETPEGKNAGLVSSLSSYARTN
SLGFIETPFFHVINGYVRKDQEAIYLTAEEEENLKIAPGDIKLGCIINSIVTVRYKTEFVNI
PVQDVQYIAVSTIQIVSAATSLIPFLEHDDANRALMGSNMQRQAVPLLYPNRPIIGTGL
ESQLAAEGGSAVINYCDGIVVEANSQFILIKYDLRKYIRSNQGTCIHQRPVVWKGEYVE
SGQVIADGPSTEGGELALGQNILIAYMPWEGFNYEDALLINERLIYSDLFTSIHIEKYDV
GVRETKTSIETRDLPNVLNYLDDNGIVKIGTFVVAEDILVGKVTPLEDSEEIPESRLLRA
IFVVDSSLRVPVLDIRIFTRIFLAQTRKIKVGDKMAGRHGNKGIISRILPREDMPYLNDG
TAVDLILNPLGVPSRMNVGQLFEGLLGFAGENLDKRFKILPFDEMFFLEASRILTNVQ
LKKAATKKSWIFDQLNPGRVYLTDGRYGEYFDNPITVCKSYMLKLVHQVQDKIHARA
TGPYSLVTQQPVGGRSRKGGQRFGEMEVWALEAFGAAYTLQELLTIKSDDIYGRNAT
LKSIPKAGIPESFKVLLLELQALGLDIGVYNIQKNFITEIDVTMFDYIKINLASPERIKKW
GQRILP-
TGFVGEVTKPDTLNYRTLKPEFDGLFCERIFGPIKNWECHCGKYRTCNRCGVEITDSR
VRRYRMGHIRLVSPVAHIWYLKGIPSYISLLLYNDLIGGQAIKYLLGNLNLVAELTDNR
TILTSLSDTRRQLIRRIRLIENFIATGSKPSWMVLTVLPVIPAGLRPMIQLDSGSFATSDL
NELYRRVITRNNRLAKLIKLYAPTLIIHNEKRMLQEAVDTLINNGKRGEKVLGSNNRPL
KSLSNIIEGKNGRFRKNLLGKRVDYSGRSVIIVGPSLKLNQCGLPCEIALELFQPFIILIN
NGARLIRIGPVIWTILNILKTHPIFLNRAPTLHRLGIQAFEPILVEGRAIKLHPLVCPAFN
ADFDGDQMAVHVPLSLEAQVEARTLMLSTNNFLSPATGEPILLPSQDMILGYYYMTV
DSLKPTTFADHYFSSISDVILAYSVNKITLHTPIWLRFEKSKYIQTTPGRVLVNQSVFKN
LMSRYRNAKLRVIRRLGLPGLTSKVPKKSKKSSSEYAIRLQEKQKLRFNYGISEKQLLNY
VKKAKRLPGATGTIILQLLEMRLDSIIFQLGFANTIAAARQIVSHGHIKVNGKKLNIPSF

QCKPNDVISIVDKKQSKNLISKNILKSHLDYNDQMKQGKVINMVDTKEIDLNINELLI
VEFYSKMGQKVHPLGFRLVTTQTHHSKWFSKFQLYPLLLEEDTAIREHLNKVISKIKIN
RNNKLKIEILSGRPVLVTYSLTQIIRAFVIKIIKLEEPDAEAALIGDFVVQQLEKRVAFRR
AIRKAIKRAVPGVKIQIGGRLNGAEIARKEWIREGRVPLHTLRADIDYAEKRANTIYGVL
GVKVWLFKMVNDTISDMLTRIRNANNAKQQLVEIPFTKMTSSLVSILETEGYLENSKV
LTRNTILVSLKYVGKKRKSVITSLKRVSKPGTRVYSGAKNLPKVLGNLGIAIISTSYGVV
TDKQARKLNVGGEILCYIWMFQEKVIQISRVTKVCKGGKKLAFRVVIAIGNENGQVGV
GVGKADDVVNAISKGITDAKRNLTNVILTKTKTIPHIVTGDFGACSVLVKPASQGTGV
IAGSSIRILLELAGIKNIVAKQLGSKNLLNNARATICAFENIMSRRSITKKRYPKRDAKYD
STLVSLFITRILRNGKKTLANSIVLDTFDAIETQTNTNPIQVFETSIKNVIPLVEVKARRI
GGSTYQVPLEVNQYRGTNLALKWIITAAKARAGRTIAEKLANEIMDASNGVGSAMKK
RQETHRMAEANKAFAHLRRMIRIELKSFDSQLINESCNQISENLKQTNSTISGPIALPTK
KRIYCVLRSPHVNKDSREHFEIRTYKRIVDIFPFTDTINLLRDLPSGIAAKIMPTIQQLLR
KKRKVTLQKSKSPALKNCPQKRGVCTRIYTTTPKKPNSAIRKVARVKLATGFEVTAYIP
GIGHNLQEHSAVLLRGGRVKDLPGVRYHIIRGTLDSGEVNDRRQGRSKYGTKKPKMK
RSKLGVTDGIVHIQSTFNNTIITITDSTGNTLSWASAGSTGFKGARKGTPFAAQTASEA
ACTTAFNLGLKKVEILIKGQGSGRETAIRAIQSVGLEITIIKDITPLPHNGCRPPKKRRV
MVRISGIDLQKKKRIEYALTGIYGIGLVSSQQILKKANVNDLRAYEIEDKDVSLIREIIKD
YQVEGDLRRKVSLNIKRLSEINCIRGRRHRQQLPLRGQRTRTNARARRGPKQTKKMA
VKEKIGIVVSDQMDKTVVVLVEKRYIHPIYGKTLKKNKRFMTHDDSNKCKIGDIVLIQ
ETIPLSKKKRWTVTMAKQCMIQREVKRQKLVLKYSNKRTTLLKELKLSNSLSDIFFVQ
KKLQRLPNNSAEIRLKNRCWRTGRSRGFYRDFGLSRHVLREMAHQCLVPGLTKSSW
MGRSIRKGPFVAYHLLKKIEVMQKQVIKTWSRSSTITPSMVGHTIAVYNGRQHVPIFIS
DQIVGHKLGEFAPTRTFRSHKSDKRIKRMIKSALKRIKLTQRNTLRNKVSTSMMRTYI
KLFITSCEEYLKQDLDIACSKVDKAKNQGILHKNTAMRKKSTLQSIYNKMTQVFMDN
GDANPVTIIKVGPCVVTQIKTVSTDGYDALQLGYHETRKCTKPELGHLNKANLASLK
YLKEYLINNEDFQLGQIIDVQTFKEGEFVDVTGKSIGKGFAGNQKRHHFTRGPMSHG
SKNHRAPGSIGQGTTPGRVYPGKKMAGHLGASTVTIKKLKILKVDTKQNLLILKGSVP
GKPGNLLSIRMAKTKGSRIIITLECTMCRTNNQRTKGVSRYTTMKNRKNSPTRLEIKK
FCRYCNQHLNHKEIKMTQVFMDNGDANPVTIIKVGPCVVTQIKTVSTDGYDALQLG
YHETRKCTKPELGHLNKANLASLKYLKEYLINNEDFQLGQIIDVQTFKEGEFVDVTGK
SIGKGFAGNQKRHHFTRGPMSHGSKNHRAPGSIGQGTTPGRVYPGKKMAGHLGAST
VTIKKLKILKVDTKQNLLILKGSVPGKPGNLLSIRMVTIPNYWIIIEKEFFKLGFSTNPD
YLIHKAFCLYRKANWVRNASTKTKSEVRGGGKKPWPQKGRGRARAGSIRSPLWRGG
GVIFGPKPIAYNPKLNNREYDRAFRVLLHEKQKRILTISLFNSGKTKYAKQIFKNVLNK
NIVISQQEYTLFLQSIKNLQNVNIVIDNCLTINDLLRSSWILMTAYSSHNLTLKNMLKM

RFKRVGRKRQPSYRLVVMLSTSPRDGKVIKELGFYNPITKELKLNLEGIQFQLNNGVK
TTKVVENLLKRLLMILKYPVISDKSTGLLESNKYTFMVDRHADKSVIKTAINSLFDVTV
LNVNTLNIPPKKRTKSKYKKAIITLKEGDTLDLFPMTVEKTDLPNIRVGDTVRLGVEIR
EGEKTRVQAYEGVVISQKNSGLSQTITVRRVMQGIGIERCFPIHSTKIKNIEIKRSSKVR
RSKLYYLRGLSGKATRLKQRF
>Monodopsis_sp._KX839260
MYKKIYPKLLKIQLNRGLGLGAQNRMVLQKTIEEVRLITGQQPIVTKAKKSIAGFKTRE
GMDLGVTVTLRNDLMYAFLEKLIHLVFPRIRDFRGLNPDSFDENGNYNLGIREQLVF
PDIDYNMIDQSRGYNISIVTSAKTPEEGRVLLQEFGFPFMVRVKRGNVARKSRKKVLGI
AKGYVGSHSRLFRIANQQVSRALRYSYSGRKDKKRWFRRLWITRINIASRNLTYSKLIYK
IKSEGLGLNRKMLAHLAVLDPKIWSVLAMGILFFKAYTPGTRHAVRPDFKELTGNKP
TKSLISNSHSSKGRNNQGRITIRHRGGGHKRRYRLIDFKRNKRNLIGKIISVEYDPNRNA
RISLVQYEDGEKRYILHPESAVTGNQIEAGPNSSLNVGNSLPLENIPLGYDVHNIELFP
NKGGQIARAAGSSAKILAKEGDYITLKLPSKEVRLVPKTCYATIGKVGNSSHMNLTLG
KAGRKRWLGKRPTVRGIVMNACDHPHGGGEGRSPIGRKHPCTPWGKPALGVKTRS
KKKTFIIRKMSRIGKQIIKIPTGVNVELTEKSIKVKGPKGEISRDIPSCLTIDSTLQISRTSR
EMHGLFRSLVSNMVIGSSNGFIKVLELKGVGYKASMAGKALTLSLGFSHPIKIEAPEGI
QISVEGNTIIKISGINKEKVGLIAQQIRSSRPPEPYKGKGVLYQGEFVQRKVGKSSKMTY
IPLGKERSWYLIDAENQTLGRLSTQIANLIQGKNQVIYTPGSDSKVHVIVINAEKIKVKL
SQKLYYKHTGKPGRSLSDLLARFPYRVIELSVKRMLPNGKSNLAKRLLVYAGNEHPHK
AQKPKEVMPKKIVAVIKLALKAGKATPAPPVGPALGQHGVNIALFCKDYNARTADKG
DLIIPVEISVYEDRSFSFILKTPPASVLLKVNLKIKKGSAKPNKIKVGSITRKGLEEVARIK
LPDLNTDNIDAAIRIIEGTAKNMGITIMLSPKRTKFRKQQRGRLRGKTIQKKHLVFGD
YGLQAQEPVWLTARQIEATRRTITRYTKRGGKLWIMVFPDKPITARAEESRMGSGKG
APQYWVAVIKPGKVLFELGGVPDNVAQQALRMAAYKLPIRTKLISAMIQPQTYLSVV
DNTGAKKLMCIRVLGSNRRYGRIGDIIIGVVKEAVPNMPIKRSDIVRAVIVRTRQPVQR
ADGMALRFDDNAAVIINTDNNPRGTRVFGPVAREIREKNFVKIASLAFEVVMYSIIESF
GRQFWVEPNKFQDFYNFKIKKVVLFDRVMFYSNVCLGRPLLNFIEGSLLPGVKKSKLV
VFKMRAKKGYKRKIGYRMTSRRVRFMAKAKARIIRMSPLKVRRVLKQIQGCSYEEALIL
LRFLPYRACHPVAKVLKSAAANAVSNYSIPRSYLQIDKAFVDKGPVLKRIRPRAKGRMC
PIKKRTSHISIVVMPKKNLHPNWFEKTKVYCDGKEIMTISSTKPELHVDIWSGNHPFFT
GSQKIIDTEGRVERFLKKYNMMAHKKGAGSTKNGRDSNAKRLGVKVVGGKFIQTGQI
IIRQRGYSFHAGENVGVGRDYTLYALKDGYVSYSVSIKMKVRPSVKKMCEKCRLIRRA
GKIRVICVNKKHKQRQGMAVPKKRTSRTKTKTRKAQWLKKADLKSKKAWSLGCSIA
NSNSFVLMRLVIEIEKRSFQFRIGPFKKTMGTTIGAAIRRTLLSLSKTVSITSA-----
CGNFFGGNSIREDLFELSLNLQRIHIKSSTARIQKVGPAIVTAQDLQLENGLEVVNPYQ

YICTLNSSYALDLHLMIMSPEVNQGLIVIVDPIYSSIQSCGFEVIQTEEFLRFVVISRGAIE
PAVAIEVAVRELIETLSILLDLRHLNLPTKLELFLRQEGFVSLQSLISVPLFRRILKHNDLI
TIEKSLNLLNQVITKKELEELLLWMFRNYGIKKTCMLAELLKEAGFNYATQGGISISLE
DLKVPRAKTPLVGSTNKELAEGETKYQRGELTSSEWFQKQVMAWNLTSELLKNEIVE
FFERTDPLNPLYMMTFSGARGNLSQVRQLIGMRGLMSDQKGEMIGSAIQKNFREGL
TVIDFLISSYGARKGLVDTAIRTADSGYMTRRLIDIAQDVVVRQFDCQSKYGVLILLGR
ILAKPLIIAHRGQEIDFHLSNKINLISIVSVRSPLTCQSYRSVCQTCFGWDLTQHRLIEIG
EAIGIIAAQSIGEPGTQLTMRTFHTGGVFSELSQQIRSNYSGQVEFANNLKTQFIRTTQ
GDYGQVIRNTSFFEIKEIIRIRVERDDLLMIKDKEFIKKGQPIVQSASELEELSTKFPGEIY
YEYVVWVLEGTLITLPEQLVRSYNFNTQVFAKTKLVIKSNPFTRYFIIKSGYVLPEETCK
QLPSLATLGNFIFWITPNIFSPVNGLIHYKFHDLSIQSGSFLFPGDRLFDFIQIDRIVYSQ
ILQPLTIYSIKNLVETGLFVIETISFVQPNQILEPYTLIGCSEVRGVYGSPVELRSQSQEL
MIVSKDHIRELYFDEKDFDTLILTIGGKILNSEGSKLQFRTGKPYLFTEGAKVYKNHKD
FAPENTVLGFVTYRIFKTQDIIQGLPKVEEILEARSSSDRAT-
AEKITDRFHVDPQQLLDISFDYFKATLRSLYKLASMFIKSIQSVYESQGIRIVDRHLEVIV
RQMIIKAKIEFSGNTPLVSGEYIDVQQLLILNTMLQNKYYKPAILGLTKAALTTESFISA
ASFQETVRILTQAAIEGRVDWLRGLKEKVIVGGLIPSGTGMFLAKMPDLVEIQKSSFC
WFLEEGLNEELKHLSSLAAFETFEVRFFHNEFYFHAPFRIPYESRRDKLSYSIRLYMPIEV
EDKRNIELHTLCLCELPLMTNRGTFVINGCERVILGQIVRSPGIYYTIGATLISYRGSWL
YFEYDRVWIKTDRIEKIPISILLEDIGSETMFSHLENSHYFEYWELNEIFEPRYYSLGKVG
RYKLNKKLGLSLPERAQTITLHDLVEIIDYLIGLRVLIDDIDSLENRRIRSIGELLQSQFS
QAFSRFGAFLDEKRDMPPISAVDEFFATNPLSQYLDQINPLAELIHKRRVTVLGPGGIP
FEKASLAIRDIHPSYYGRLCPIDTPEGEKAGLVASLATFVETDAQGFLKTPFFVIKQGKI
IGLN---
YLSVGQEDGKSIAMGDSLVGFLLTKRIGVKENDEFRITSPSEIKYLSISPFQLFSPAVGLI
PFFEHDDANRTLMGAHMQRQAVPLLRPQKPIVGTGIESHLAIESGSLVLSYGKGTVQ
FVSSNSIWIRYKLEKAQSSNQSTIITQRPIVWIGEQVEAGSVIADGPSTDEGELALGKNV
TVAYMPWAGYNYEDAIVVSERLVYENLFTSIHIEKLEIEIEDNDRGPDTKDLPESLRNL
DQNGLVYTGVYVQPGDALVGKLSPKRRENT--
YGRLLEELFVADTSLRVPTLDVRVLDRVFIANIRRIQIGDKMAGRHGNKGVISTVAPLA
DLPFLPDGTLVDIILNPLGVPSRMNVGQLFECLLGWAGEKLERRFKIFPFDEMYGME
ASRTLVYQKLSEIKSSVNKILKDSTPGKVILKDGRTGQPFDNPVTVGKPYMLKLIHLV
DDKIHARSIGPYSVITQQPLGGRSREGGQRFGEMEVWALEAYGTAHTLQELLTLKSD
DIPGRLRAYKAIPSPGIPESFRVLLRELRSLAVDIHALRFIAHSPNVKPVKMFEMVGVKL
ASPQKIREWSERKLP-
NGIVGEIRKPDTMNYRTGRPEPDGLLCEKIFGPLKSWECSCGQYKYCPSCGVEVTESH

VRRHRMGHVALNYPITHTWYLKGSPSYLSIILFNKRNGSKRILNMLESLDLKEEIKNVR
EELFNSSSRRERLLKRLRLLETFVATKTDPSWMILTVLPVLPPALRPVFELPNGTYMSS
EFNEHYRRIVMRNNRLLRFCEQIVPDFVVINEKILIQDAVDDLIDNGSRGRDSYGPRN
KPLRCLNDLIAGKEGRFRQNLLGKRVDYSGRSVIVVGPTLMLDQCALPYEMALNLFG
PYLIIIPRALTALQRNPIVWTLLLIVSVSLVLLNRAPTLHRLGIQAFRPILTTGRAIQLHP
LVCPGFNADFDGDQMGVHLPLTRKTQLEAYR-
MLSSNNLLSPATGRPILTPSQDIVLGWYYITTRTLPNRKGGNRYFTSFSEISEALEHDKI
SLHSTVWVKYITHCYIRTTPGRILINELLAAAIMSRYRGPRLRLQRRLGLTHLTRKRIKN
KKKVRSEYRMRLEEKQKLRFNYSITEAQLFRYVKEARRIKGSTGLIILQLLEMRLDTIVF
RLNLSPTILAARQLVSHAHVKVNNHVVTIPSFQCQPGDSIQLSDKKFIREIVEDNTLPY
HLAGSLKQLRGGVKKVVDRKDIRLPVNELLVVEYYSRMGQKVHPIGFRLGITEEHKSK
WYAKFSDYSRQLQADDELRIIWSNLRTNLIISYPDQIHLIIYTVNPILILSSLGKELKNLVI
VLKKVKYPHIQAGFLAQSLAKKLEKRMPFRRAVRTILGDFKKGLKVQVAGRLNGAEIA
RTEWVREGKVPLHTLQAKLEYATERAQTIYGILGIKIWMCKMKTDTISDMLTRIRNG
NLVGHKVVKIDSTRMTYNLTRLLRSEGFISQYETVGRRYIFIYLKYLANSNKPLIRGLKR
VSKPGLRVYVGSNEIPSILGGQGIAILSTSKGIITNVQARELGIGGELLCYIWMINHKVIE
IRRVCKVGKGGKTLSFRALIVVGNQKGIVGLGVGKASQVLNAIKKGQSKAIQNRIQIA
MTRTKTIPHKSEASFGAALVILRPAAPGSGVIAGGAIRTVLESAGIKNILAKQIRSKNKIN
NARATIVALNNLMSRRKRIKKRLPGPDSVYKSYLVSLLSLRVLKSGKKQLAERIVYRALE
YVKQKTNVEGLVTLEKAVQNVSPIVELKPKRIGGATYQVPIEVKRLRATNLALKWILR
FSRDRSGKNMSFKLARELMDASKGIGNSIRRREEVHKMAEANKAFSFFKRMFRIRLKAY
NSRVLRISSLLLQQELKRLDTLVKGPVRLPVKKRYYSLLRSPHIDKASQEQFEIRRHKWI
FDIQVPKNYINLLSSFPPGVDISIMPTVQQLIRNERTSLKKKSKAGALKACPQRRGVCT
RVYIVTPKKPNSAMRKVARIRLTSGFEVTAHIPGEGHNLQEHSVVLIRGGRVKDLPGV
RYRVVRGALDTVGVVKRTQGRSKYGARKAKMGKRKINLSLGFACIHSTFNNTIISITD
PKGNVLTWASAGSSGFKGSRKKTQYAAQMAAKNASAKALKLGIKKVGVILNGPGNG
REIAIKGIHATGLEIISIEDKTGVPHNGCRAPKRRRVMIRIAGVDLPDTKSIEIALTYIYGI
GRTTSKEILNSIDINETKTKALTDIEVGKLRDRIENYTTESDLKRLVALNIKRLIEINCFR
GRRHIQGLPLRGQRTRTNAQTRKKTAGRRKMGLKEKFGIVVSTKMQKTIVVLVENKF
RHPRYAKTMTKTKRYLVHDEDNAAKLGDKIIITQTRPLSKNKSWKLEMAKLSVIKRQ
TRRENLVRRYSEVRQQLKNELKKENSFKVKMELHIKLQKLPRDSSITRLKNRCWKTGR
GKSVFRDFGLCRNVVREMANQGLLPGVVKSSWMIRSTRKVPFVAYHLFKKINDLKTD
TIKTWSRASVILPSMVGFTIAVYNGRQHVPLFINDQLIGHRLGEFVPTRTFRSHKNDR
KTKRMIKSAKKRVLIGETNRRMNRFYKSSIKSLVKKYRTNIKNFLESLVSKIYSRIDKAAK
KKVFHLKKAARQKSRISKVLKKMTQLFDEKNASIPVTLIKIGPCTVTQIKTVPTNGYNA
VQLGFDLKKSLNKALEGHLKKSGALNSRYLKEYHVNKEEFELGQTFDVTNFSTNQFI

DIRGKSIGKGFAGTVKRHNFSRGPMSHGSKNHREPGSIGAGTTPGRVYPGKRMAGRL
GGKPTTLKRLAILRIDPEENLLVVKGSVPGKPGNLLSLTMAKQKGSRIIIHLECTTCRT
NNKRSGGVSRYTTRKNRRNTPARLEIKKFCPHCNSHTLHKETKMTQLFDEKNASIPV
TLIKIGPCTVTQIKTVPTNGYNAVQLGFDLKKSLNKALEGHLKKSGALNSRYLKEYHV
NKEEFELGQTFDVTNFSTNQFIDIRGKSIGKGFAGTVKRHNFSRGPMSHGSKNHREP
GSIGAGTTPGRVYPGKRMAGRLGGKPTTLKRLAILRIDPEENLLVVKGSVPGKPGNLL
SLTMATIPNYWIIIEKDFFKLGFSTNPDYLIHKAFCLYRKTNRIRNASTKTKSEVRGGGR
KPWAQKGRGKARAGSIRSPLWKGGGVTFGPKPIAYNPKLNNREYDRAFRVLLHEKQK
RILAISLFSSGKTKYAKQVFQNVLNNTIVVSPQEYTLFLQSIKNLKNINLIVDNKLSIND
VIRSSWLLMTAHSSHNLTLKDMLKIRLKRCGRKKLPSYRIVLVRSQSRRDGRAIEELGY
YNPMSNELVYNENRVKLRLSQGAQPSQTVKNFLVKLIMLLRYPLATEKVSNPFLKNTY
TFVVHPKASKKTIKQAFEYVFKVKVLSINTLMMPKKSRRLPTYKKAILKLAPEYSLDFF
GMSSENKKLPNVSVGDTVRIGVLLIEGNKERTQYFEGTVIAIKSREGKKSLRVRKLSQG
IGIEKLFLLDSPKVVSLERKKITKVRRAKLYFLRTLSTKTKKLKDQS
>Nannochloropsis_oceanica_KC598086
MYKKIYPKLVKIQLNRGLGLGAQNRMVLQKTIEEIRLITGQQPIITKAKNSIAGFKTRE
GMDLGVTVTLRSDLMYAFLEKLIHLVFPRIKDFRGLNPDSFDENGNYNLGIREQLVFP
DIDYNMIDQIRGYNISIVTSAKTPEEGRLLLQEFGFPFMIRVKRGNVARKSRKKVLGLA
KGYVGSHSRLFRIANQQVSRALRYSYSGRKDRKRWFRRLWITRINIASRNLNYSRLIYKL
KSESIGLNRKMLAHLAVLDPQIWATLGMGILFFKAYTPGTRHAVRPDFKELTGTKPT
KSLITSSHSSKGRNNQGKITIRHRGGGHKRRYRLIDFKRNKRNLLGRIISIEYDPNRNAR
IALVQYEDGDKRYILQPESVITGNQIESGPNSSLNIGNSLPLENIPLGYDVHNIELFPNK
GGQIARSAGSSARILAKEGDYVTLKLPSKEVRLVPKNCYATIGKVGNASHMNLTLGKA
GRKRWLGTRPTVRGVVMNACDHPHGGGEGRSPIGRKHPCTPWGKPALGVKTRSKK
KTFIIRKMSRIGKQIIKIPAGVNIQLTELNVKVKGPKGEIARDIPSCLNVQNTLQIFIRSR
EVHGLFRSLLSNMVIGSSAGFTKVLELKGVGYKANMDKTTLNLSLGYTHPIKIEASEGI
QISVESNTIIKISGVNKEKVGLIAQQIRSKRPPEPYKGKGVLYQGEVIQRKVGKSSKMTY
IPLGKERSWYLIDAENQTLGRLSTKIANLIQGKNQVTYTPGSDSKVHVVVINADKIKV
KLNQKFYYTHTGKPGRSLAELLARFPYRVIELAVKRMLPNGKSDLAKRLRVYAGNEHP
HKAQKPKEVMPKKIVAVIKLALKAGKATPAPPVGPALGQHGVNIALFCKDYNARTAD
KGDLVIPVEISVFEDRSFSFILKTPPASVLLKVNLQIKKGSAKPDKVKVGSISRQGLEEIA
RIKLPDLNTDNIDAAIRIIEGTAKNMGITIMLSPKRTKFRKQQRGRLRGRTIQKKHLVF
GEFGLQAQEPVWLTARQIEATRRTITRYTKRGGKLWIMVFPDKPITARAEESRMGSG
KGAPQYWVAVVKPGKVLFELSGVPDNVAQQALRMASYKLPIRTKLISAMIQPQTYLS
VVDNTGAKKLMCIRVLGSNRRYGRIGDIIIGVVKEAVPNMPIKRSDIVRAVIVRTRQPV
QRADGMSLRFDDNAAVIINTDNNPRGTRVFGPVAREIREKNFVKIASLAFEVVMYSIIE

SFGRQFWVEPDKFQDFYNFKLKKIVLFDRVMFYSNVYLGKPLLSFIEGSLLPGVKKSKL
VVFKMRAKKAYRRKIGYRMSSRRVRFMATAKAKIIRMSPLKVRRVLRQIQGCSYEEALI
LLRFLPYRACHPVAKVLKSAAANALSNYSIPRSYLQIEKAFVDKGPTMKRIRPRAKGRM
FPIRKRTSHISIVVMPKKNIHPSWFEKTKVYCDGKEIMTISSTKPELHVDIWSGNHPFFT
GSQKIIDTEGRVERFLKKYNMMAHKKGAGSTKNGRDSNAKRLGVKVVGGKFIQTGQI
IIRQRGYAFHAGENVGIGRDYTLFALKDGYVSYTVSIDMKVRPSVKKMCDKCRLIRRA
GKIRVICVNKKHKQRQGMAVPKKRTSRTKTKQRKAVWLKKADLMSKKAWSLGCSIA
SSNSFVLMRLVIEIEKRSFQFRIGPFKKTMGTTIGAALRRTLLSLSKTLAITSA-----
CGNFNNGNSIREDLFELSLNLQRIHIKSSTARIQKVGPAIITAQDLKLDEGLEVINPYQY
ICTLNGSYPLDLHIMISSPEVNQGSIIVVDPIYSSIQSCGFEVIQTEEFLKFVVVSRGDIEP
AAAIELAVLELQETLTILLDLKHLNLPVNLELLLRREGFVTLQNLISIPLFKRILKENDL
VSIEKSLNSLNQVITKKELEELLLWMFRNYGIRKTCMLAELLKEAGFNYATKGGLSISL
EDLKVPRTKTPLVSSTNKELANGETKYQRGELTSSEWFQKQVMAWNLTSEVLKNEIV
EFFERTDPLNPLYMMTFSGARGNLSQVRQLIGMRGLMSDQKGEMIGSAIQKNFREGL
TVIDFLISSYGARKGLVDTAIRTADSGYMTRRLIDIAQDVVVRQFDCQSKYGILILIGRI
LAKPLILANRGQEIDFHLSNKINLIPIISVRSPLTCQSYRSVCQTCFGWDLTQYRLIEIGE
AIGIIAAQSIGEPGTQLTMRTFHTGGVFSELSQQVRSNHSGQVEFANNLKTQFIRTTQ
GDYGQVILTTSFFEIREVVRIRVERDDLLMIRNQEFIKKGQPIVQSASDLEDLSTKFPGE
IYYEYIVWVLAGNLVTLPEQIVRSYKPNNQVFAKTKLVIKSNPFTRFFILEGGYLLPEEIY
KHITSLFHVGNFIYWIDQNIFSPINGLISYKINDLSIQSGSLLFPGEILFGQIKVNRIVYSQI
LRPITIYSIKSLIETGLFVIKTISSVKQNQILEPYTLIGCSEVRGIIGQPISLRSQSQELMIVS
NDHIRELYFDEKDFDNIVSTIGGKILSYTGSKIQFRIGKPYLFTEGAKVYKNHEDFAPES
TVLGFVTYRIFKTQDIIQGLPKVEEILEARSSSDRAL-
AEKLTERFHVDPQQLLDISFDYFKATLRSLYRLASMFIKAIQGVYASQGIKIVDRHLEVI
VRQMIIKAKIEFPGNTPLVSGEYLDVQQLLILNNMLEKKYYKPAILGLTKAALTTESFIS
AASFQETVRILTQAAIEGKVDWLRGLKEKVIVGGLIPSGTGMFLANMPDLVEIQKTSF
CWFLEEGLSEELRNLSSLADFETFEVRFFHNDFYFHAPFRIPNESRRDRLSYSIRLYMPI
EVEDKRTIELHTLCLCELPLMTNRGTFVINGCERVILGQIVRSPGIYYTIAATLISNRGS
WLYFEYDRVWIKTDRIEKIPISILLEDIGAHTMFSHLENSHYFEYWELSEIFEPKYYSLGK
VGRYKVNKKLGLSLPDKAQTLTLHDLVEIIDYLIGLRVLIDDIDSLENRRIRSIGELLQS
QFSQAFLRFGAFLDEKRDTPPISAVDEFFATNPLSQYLDQINPLAELMHKRRVTVLGP
GGIPFEKASLAIRDIHPSYYGRLCPIDTPEGEKAGLVASLATFVQTDAQGFLKTPFFSIN
KGKITGLD---
YLSVGQEDGKSIAMGDSLIGFLLTKRIGVKENDEFRITSPSEIEYLSISPFQLFSPAVGLIP
FFEHDDANRTLMGAHMQRQAVPLLRPQKPIVGTGIESHLAIESGSLVLSYSKGIVRFVS
SNSIWIRYKLEKAQSSNQSTIISQRAIVWIGEQVESGSVIADGPSTDEGELALGKNVTVA

YMPWAGYNYEDAIVVSERLVYENLFTSIHIEKLEIEIEDNDRGPDTRDLPESLRNLDEN
GLVYIGVYVQPGDALVGKLSPKRRENT--
YGRLLEELFVADTSLRVPTLDVRVLNRVFIANIRRMQIGDKMAGRHGNKGVISTLAPL
ADLPFLPDGTLVDIILNPLGVPSRMNVGQLFECLLGWAGEKLERRFKVFPFDEMYGT
EASRTLVYQKLSEITSEEEQILKKSTPGKVVLRDGRTGQPFDNPITVGKPYMLKLIHLV
DDKIHARSIGPYSVITQQPLGGRSREGGQRFGEMEVWALEAYGTAHTLQELLTLKSD
DISGRLRAYKAIPSPGIPESFRVLLRELRSLAVDIHALRFMAHSPNVKPVKMFEMVGVK
LASPQKIREWSERKLP-
NGIIGEIRKPDTMNYRTGRPEPDGLLCEKIFGPLKSWECGCGQYKYCASCGVEVTESH
VRRHRMGHVALNYPITHTWYLKGSPSYLSIILLRACNGSKKILNMLESLNLREEVKYVR
TELFHSSSKRDRLLKRLRLLETFLATKTEPSWMVLTVLPVLPPALRPVFELPNGTYMSS
EFNEHYRRIVMRNNRLLRFCEQIVPDFVVINEKILIQDAVDDLIDNGSRGRDSYGPRN
KPLRCLNDLIAGKEGRFRQNLLGKRVDYSGRSVIVVGPTLMLDQCALPYEMALNLFG
PYLIIIPRALTALQRNPIVWTLLLIVSVSLVLLNRAPTLHRLGIQAFRPILTTGRAIQLHP
LVCAGFNADFDGDQMGVHLPLTRKTQLEAYR-
MLSSNNLLSPANGRPLLTPSQDIVLGWYYITTRTLPNRKGGNRYFTSFSKISEALEHGT
ISLHSTVWIKYIISSYIRTTPGRILINELVAAAIMSRYRGPRLRLQRRLGLSHLTRKRVRN
KKKIRSEYRMRLEEKQKLRFNYCITEAQLFRYVKEARRIKGSTGLIILQLLEMRLDTIVF
RLNLSPTILSARQLVSHGHVKVNNHVVTIASFQCQPGDSIQLSDKKSIREIVEENALPY
HLAGSLKQLKGGVKKVVDRKDIRLPVNELLVVEYYSR-------------------------------------
----

MPFRRAVRTILSDFKKGMKVQVAGRLNGAEIARTEWVREGRVPLHTLQAKLDYATG
RAHTIYGILGIKIWMCKMKTDTISDMLTRIRNGNLVGHKVVKIDSTRMTYHITRLLRT
EGFISQYETVGRRYIFIYLKYSANPTRPLIRGLKRVSKPGLRVYVSNNQIPIVLGGQGIAI
LSTSQGIITNIQAKELGIGGELLCYVWMITHKVIEIRRVCKVGKGGKTLNFRALIVVGN
QKGIVGLGIGKASQVLNAIKKGQSRAIQNRIQIAMTRTKTIPHKSEASFGAAQVMLRP
AAPGSGVIAGGAIRTVLETAGIKNILAKQLRSKNKINNARATLVALSNLMSRRKRIKKR
LPGPDPIYNSYLVSLLSLRVLKSGKKQLAERIVYRALDYVKQKTSVEGLVTLEKAVQNV
SPIVELKPKRIGGATYQVPIEVKRLRATNLALKWILKFSRDRSGKNMSFKLARELMDAS
KGIGNSIRRREEVHKMAEANKAFSFFKRMFRIRLKAYNSRVLRISSLLLEEELKRLDTLV
KGPVRLPVKKRYYSLLRSPHIDKASQEQFEIRRHKWIFDIKVPKNYINLLSSFPPGVDISI
MPTVQQLIRNERTTLKKKSKAAALKACPQRRGVCTRVYIVTPKKPNSAMRKVARIRLT
SGFEVTAHIPGEGHNLQEHSVVLIRGGRVKDLPGVRYRVVRGALDTVGVTKRMQGR
SKYGARKAKMGKRKINLSLGFACIHSTFNNTIISITDPKGNVVTWASAGSSGFKGSRKK
TQYAAQMAAKNASAKALKLGIKKVGVILNGPGNGREIAIKGIHATGLEIISIEDKTGVP
HNGCRAPKRRRVMIRIAGVDLPDTKSIEIALTYIYGIGRTRSKEILSSIDINETKTKALTD

TEVSSLRERIESYTTESDLKRLVALNIKRLVEINCYRGRRHIQGLPLRGQRTRTNSQTR
KKTAGKRRMGLKEKFGIVVSTKMQKTIVVLVENKFRHPRYAKTMVKTKRYLVHDED
NAAKLGDKIVITQTRPLSKNKSWKLEMAKASVIKRQLRRENLVRRYSDLRRQLKNELK
KESSFRAKMELDIKLQKLPKDSSKVRLKNRCWKTGRGKSVFRDFGLCRNVVREMANQ
GLLPGVVKSSWMIRSTRKVPFVAYHLFKKINNLKTDTIKTWSRASVILPSMVGFTIAVY
NGKQHVPLFINDQLIGHRLGEFVPTKTFRSHKSDRKAKRMIKSAKKRVRIGERNRKM
NRFYKSSIKTLVKKYRTNIKTFLQTLISKIYSRIDKAAKKKVFHLRKAARQKSRISNVLKK
MTQIFDEKNASIPVTLIKIGPCTVTQIKTVPTDGYDAVQLGFDLKKSLNKALEGHLKK
SGALNSRYLKEYHVNREEFELGQTFDITNFSPNQFIDVRGKSIGKGFSGTVKRHNFSR
GPMSHGSKNHREPGSIGAGSTPGRVYPGKRMAGRLGGKPATLKRLAILKIDADENLL
VVKGSVPGKPGNLLSLTMAKQKGSRIIIHIECTTCRTNNKRSEGVSRYTTVKNRRNTP
ARLEIKKFCPHCNSHTVHKETKMTQIFDEKNASIPVTLIKIGPCTVTQIKTVPTDGYD
AVQLGFDLKKSLNKALEGHLKKSGALNSRYLKEYHVNREEFELGQTFDITNFSPNQFI
DVRGKSIGKGFSGTVKRHNFSRGPMSHGSKNHREPGSIGAGSTPGRVYPGKRMAGRL
GGKPATLKRLAILKIDADENLLVVKGSVPGKPGNLLSLTM--------------
LNEFEPKKSIGLIHKVYLSDLKNNRQYTASTKTKSEVRGGGRKPWKQKGTGRARAGS
NRSPLWVGGGVIFGPKPRIVFKKINKKEKKLAILSALYLNIKKIHFLENNFSIKTKNVIELI
VNENSKILIILEKTNKNFWLASRNLKNITLTTANCLNLKNLLHCNEILLSKPCLNVINSI
YMLKIRLKRCGRKKLPSYRIVLVKSQARRDGRAIEELGYYNPMSNELVYNQDRIKLRLS
QGAQPTLTVRNFLVKVIMLLRYPLATEKASNPFLKNSYTFVVDPKADKQTIKAAFEYV
FSVKVLSVNTLMMPKKSKRLPTYKKAILKLAPEYSLDFFGMSLTRPEFPTVSVGDTVRI
GVLLIEGNKERTQYFEGTIIAIKKREGKQSLRVRKLSQGIGIEKLFLLDSPKVVSIERKKI
TKVRRAKLYFLRTLGTKTKKLKDQS
>Neotessella_volvocina_strain_CCMP1781_MH795132
MLKKKYPKIEKIQISSGLGLNAQNRVFLQKSIDEIRAITGQQPILTYAKKSVAGFKIREG
MPLGLTVTLRREKMYSFLEKFISLVLPRIRDFRGINPYNFDKYGNYNLGISEQLVFPEID
YDSVEQRRGLNITIVTTTSNSKESMFLLKELGLPFMVRVKRGNIAVKRRRKYLKMGKG
FVGVHSKLAVRAGEQVIQGLNSAYIGRKLKKRTFRKLWIYRINAASRANIYSKLIGCLRN
INIFLDRKILSLLASIDLSSFNVVEMAIKLYKPYTPSNRNRSILDFSSLSKIKPEKSLIVSNH
RSKGRNNEGKITIRHKGGGHKRKYRLIDFKRNKLNITGTVISIEYDPNRNANISLINYSD
GEKRYILHPEKLNIGNNICSGPTANIEPGNALPLENIPLGTEIHNIEFFPGKGGQLIRAA
GTTAKVIAKENEFVIVRLSSKEIRLFKKECYATIGRISNSDFYNVRSGKAGRSRWFGIRPT
VRGSVMNPIDHPHGGGEGKCPVGRPQPLTPWGKPALGVKTRKKKKSFIIRSMSRIGK
REIIIPENVSVNIETNKIKVNGKHGLLERELSNLLKIDNKILIEKQANQLHGLSRVLIQN
MVTGVDKKFYKILIAEGVGYRFQVEKKLLTLNVGFTNPVKFEIPNDLEVKLESNTKIII
NGIDKEKVGLFAAKIRDIRPPEPYKGKGILYDGEKIIRKVGKTGRMTIFPKKEVPKWFVI

DATGKTLGRLVTEVSKILRGKDISFFTPGIDQGNFVVILNAEKIYIKENQKLYYRNSQR
PGETFKQIKRRKPVHIIERAVWGMLPKNGRQYYRRLYVYCSNDKSSLIAKKELQ-
MKKIKAFVKLALPAGKATPAPPVGPALGQHGVNISAFCKEYNSKTADKIGLIIPVKITIY
EDKSYSFILKSPPASVLLAKFANIKKGSSQPNKTNVATVTLEQIKEIATIKMNDLNTKNI
EKAILIIQGTAKNMGINIMLSPKRTKYRKHQRGRLKGKASRNNKLNKGEFGIQALEPV
WLTSRQIEAVRRTISRYTKRTGKLWIKVFPDKSVTARAEESRMGSGKGAVEYWVVVVK
PGTVLFEIKGVPEDIALQALRTACYKLPIKTRILKKMIQPQTYLTVADNTGAKKLMCIR
VLGNNRKSANIGDIIIGVVKVALPNMVIKRSNVVRAVIVRTKKTVRRTDGMAIRFDDN
AVVIINAENNPRGTRVFGPVAREIRDKNFTKIISLANEVVMFAITHNSGKQFLLEPGQW
YDVDFINGGDIIYMNKVLFLRKIQLGQPFLGSIPAKIIQQVKGKKIIVLKTKPKKNYTRT
RGHRQLYTRVQIMISATSKYIRISPTKVDVILKKIRGKTYKESLQILKYIPQKAGSIVWQT
LYSAVCNATNNFNYQKEKLIITEAFVNQGPILKRMRPRAQGRAFVIQKKISHLTIRVMA
KKEIHPEWY-
KTKVFCDGKLIFEVGGTKPELYVDIWSGNHPFYTGSQRIIDTEGRVEKFQRKYNIMAH
KMGAGSTKNTRDSKAKRLGVKCTGSQNVQKGSILVRQRGTKFKPGIKVGCGRDHTL
YALADGEVNFTVNIIMKVRPSVKKMCKDCRIIKRHGKVMVICINPKHKQRQG----------
----
LITCVEYGIKYGCFVIEPLNTGQGLTLGNSLRRTLLTDLTGFAISGVRINDLPHEFALIP
GLREDCLEILLNLKEIIFKTAQGFLNIKGPIILTAGLFKLPKNIQILNPNQYLGTIVDSNE
IYIEIDIESGKGYRLSTLYIDASFMPVKKVDYKIRLIKESLTLDIWTNGSITPQRALQEAIK
ILLNLYYPL--
ITQTVTKKILETIVHETFTQFGILSSSSLLDSLKLLGFYYATNAGISINIEDLRTPEAKKK
YIEIANKEIEHISEQWQQGFVSDIERFQNIIDNWNLATESLKNKIIDYYERFDPANNLYI
MAFSGARGNMSQVRQLVGMRGLMSDQEGKIIDIPIQTNFREGLSSIDYIISSYGARKGI
VDTALKTADSGYLTRRLIYIAQDLVIREQDCKTKQGVLFLIGRTLISARKNDKSIITLNK
DILNEFRSPLLLNVRSPLTCKSNGSICQKCYGWDLAQNKLVALGEAIGIIAAQSIGEPG
TQLTMRTFHTGGIFTETLKQIFAPFSGKILIPSSLKTISYRTNHGILVAKVQQESKVLLR
KETDIFLSIGTYLYISNSSFVKEGQLIAEYSIQSLSVYTMMEGEIRFQDVLWITSGKIFSLP
EDYLFPLILDKRPFALQKIVSPYSGKIQSFII--

VSLSFFDLTKIKFSSSIKTIQHIDKFTILGFLYFFPCEGKIYSLKKKIKTYFCITESDVWKIN
NEQKNINSIKFSRSGFFLYKDGFKMIFQNATPIFLSRGTILNYKQGDFVFKNQIFATLIN
YTQQTEDIVQGLPKIEELIEARKPKFSPETNLIIGEPITIDAHELLNIFFNYHKGIERSLSK
FQLILVNSIQAIYQSQGVDISSKHIEIIVKQMTSKVVIKDSGDTPLLPGELIRLSFITEIYK
AFQTIKFEPVLLSASNCSLNKDGFLSPAGFQETKRVLTKAAIEGSVDWLRGLKECIILG
RLIPAGSAMFVNNLGDLSEIQRASFYRFLLYGISEELATFPNPFLTLKLFVFLYTNNVKL

KGPMSLLEDCFHRDSTYAIQFYVPSEYNTKTIRVKQDIFFGEIPLMTEEGTFVINGCERI
VISQIIRSPGIYFLYTATVISNKGLWTKFILDSIFLKLTDFQKLFIFDLIRYFGFSEISDSLK
YSLHLGHFYLKIFYNSGCFSIGEIGRYKINKKLGINLPKDITYLTIYDFIGIIDGLIELKYY
DDDIDHIKNKQLRSIGELLQNQIRIGLYRIQKSLLEDIKAPPIAGFKEFFQTSQLSQFMD
QINPLSEMTHKRRISVFGPNGLKRDHISTVIRDIHPSQYGRICPIETSEGQNAGLIMSLT
LLGRVGTLGSLETPYFFMEKSHVFSDKSTIFLDPEQESDIKVAFCDISLKKVLSSYLSVK
DNYFFSAEKINEINFITTSPLQVVSLATALIPFLEHNDANRALMGSNMQRQAVPLLYP
QKPIIGTGLETIAILDCGMVIKTYSQGQVVFSSASHIEIKYFLRKYVRSNQETSLNQRPIV
WSGEQVFSGQIIADGPGTNDGELALGRNLTVAYMPWEGYNYEDAIIINERIILEDCLTS
IHIEEYETELNYSIIGIETRELNNLRRHLNNEGIVNIGSYVKEHDLLVGKLTPC-
EEDPSPEANLLRALYFKDTSLRVSVIDVRVLPSIYIAQKRKIVIGDKLSGRHGNKGIVSRI
LSRQDMPYLPDGTPIDIIFNPLGVPSRMNVGQIFESLLGLAGEKLGKRFKILPFDEVYE
KEASRILVNQKLKEAAPSYSWIYNEFYPGKILLRDGRTGEYFDNPITVGKSYILKLIHLV
EDKIHARATGPYSMITEQPLSGKAQNGGQRFGEMEVWALEAYGCSNILQELLTIKSD
DIDGRNDMYESIPNPSIPESFLALIRELHALGLDFSIRKFEGETQIEKDIFMFDYLKITIAS
PTRIRNWADRILP-
NGIVGEVTKPETISFRTHQPETGGLFCEKIFGPIKNWKCKCGQYNVCETCLVEITEAR
VRRYRMGYIELTCPVTHLWYLRGIPNYLCILLSQKKKGGEIIKAALEAIDLNYEIKLARS
VIQSNSNLDKTLIRRIRILESFLATKTNPSWMVLTTLPILPPNLRPLVELDQTRLVAAD
VNEIYRLIITRNQRLHDFIHFIAPDLITVHARKLLQEGVDSLIDNARLPKKALFLNNKPL
KSLTEILEGKQGRFRQSLLGKRVDYSARSVIIVGPTLRLNQCGLPYDMATELFQPFLIL
LKTKAQMIKKNPFIWRLLILTNKYSVLLNRAPTLHRFGIQAFDPIIILGQAIHLHPLVCT
GFNADFDGDQMAVHLPLYESSQLEARKMMRPCYNVLSPSNGDVILKPTQDMVIGSY
YLTILFSNSNFFVEKWFSNENDALSSFYQKKLNLHSPILVRYYLLTFLETTPGRLIFSKNF
KNSLMARYTGPKIRIIRRLGLPGLTRKNTL-
RRSSLDDYKERLFEKQKLRFNYGVTEKQLVAYFKEAKRLTGSTGNLLLELLESRLDCVI
YRLGFAPSIPSARQMVNHGHILVNGIAINIPSFLCQSNDLITVRNKIPSRKLISKNLLPKH
LEINTTLLTGRFLNPVKRKDVLVKINELKVVEYYSRMGQKTNPKGFRLVTTQNSLSE
WYGNKFIYPELIEEDFFIRTKVNEFISKIEINRIEYVNITISALFPIINLLNIRNLIKNYFIGIK
FIKNPFEDATLIAKFIAEQLEKRIPFRRAVKQTIKKVRKGVKVQVSGRLNGIDIARSEWK
RDGRIPLHTLKAKIDYTHQRAETVYGVIGIKVWLFVMTNDLVSDMLTRLRNASLARH
SFTTIKFSKLSLSILKVLQDEGYIKSYETENSYEIRVILKYKGWIKKPLFSKLQRISKPGLR
VFSGYKDFNKCLDILGTAIVSTSIGVMSHIKAKNLKKGGEILCYIGMYEEKIVQVKRVT
KVVKGGKKLTFRAIVIVGDNIHKVGVGIGRADDINIAIDKAVLNGKKNLINVPLTFKDS
IPHVVNASFGACQIMLRPASQGTGVIAGGSVKTVLELAGIKNILAKQFGSNSILNNAKV
TILALTFLMSRRISTKKRFPERDIKYDNLLVSLLVNRILKSGKKRLAKRIVYKSFDLIQYR

TNQNPIMLLEKAIRNISPRVQLKAKRVGGATYQVPTLLTKFRSTNIAIRWLVEFSRKRT
GKGMYLKLANELLESSKGLGNSIKKKEETHKMAEANKAFAQFRRMFRIFLKSFDNDLI
NNASKQIRLSLEKTDCELKGVVSLPVKCKRFCVLRSPHIDKDSREHFEIRFYKRFIDLQT
ESSVIDLLLELPAGVSCSLMPTIQQLVRLNRIPVKNLTKTPALKQCPQRRGICTRVYTT
TPKKPNSAIRKVARVKLTSGFEVTAYIPGEGHSLQEHSIVLIRGGRVKDLPGVRYKIIRG
ALDCTGVKDRQQGRSKYGARKPVMKKQKKSTGLGIVFIKTTFNNTIITIADSQGNTL
CWASSGTAGFKGARKNTPFAAQTAAKNAAAKAIELGMEKVEVVIRGRGNGRETSVRA
LKFAGLNILSIEDKTAVAHNGCRPPKKRRLMARILGNNLPNKKKIYVALTCIYGIGIPTS
KKILKSLNISNIRVSELSDGDISAIRDFLENFKLEGDLKRFVSLNIKRLIDINSFRGKRHIK
GLPVRGQRTRTNNRTSRRQSLFKRMTKKERIGIVVSNKPEKTIVVAIQTRYQHKKYAK
TLIKTKRYMAHDEENLCKSGDLVLLEESSPYSRHKKWKLKMAKKSMIQREIKRQKLVK
KYAKKRLDFLNELKKQKNLEDIFLLNEKIQKLPKNSISIRLRNRCWKTGRPRGFSRFFG
LSRNALRELAHNCFLPGIIKASWMTRSLKKGPFIAYHLLNKILHLKKETIKTWSRSSTIL
PIMIGHTISLYNGQKHVPIFISEQLVGHKLGEFVVTRTFRSHKTDRKIKRMKQRNKKL
VLQNKRNRMINRHYTTTIKNLSKLLISNRQEIRLILIKKFNSSVDKAVKKGVIHKNTAAR
KKSKLTRIIINMTQIFDEKGNRIPVTIIKIGPCYITQIKSIENCGYNAIQLGYLESKKLTKP
KLGHFNKVNLPPFRYLKEYKVLEFNYKIGQQINTDLFEIGQHVNITGFTIGKGYTGNIK
RNNFNRGAMSHGSKNHRLQGSLGAGTSPGRVFPGKKMSGQSGMEKKTVLGLEIMNI
DKNENLIVLKGCIPGKSGNLVSINMAKNKGPRLMITLECTECRKNNKRSTGVSRYLTQ
KNRRNTPEKLELTKYCKYCNKHTIHKETKMTQIFDEKGNRIPVTIIKIGPCYITQIKSIE
NCGYNAIQLGYLESKKLTKPKLGHFNKVNLPPFRYLKEYKVLEFNYKIGQQINTDLFEI
GQHVNITGFTIGKGYTGNIKRNNFNRGAMSHGSKNHRLQGSLGAGTSPGRVFPGKK
MSGQSGMEKKTVLGLEIMNIDKNENLIVLKGCIPGKSGNLVSINMQKFIKYIPLFTGKA
LQLSVLKSGNYLLHKDLLRHYNSQRQGTVSTKTRSEVRGGGRKPWRQKGTGRARAG
SNRSPLWKGGGVIFGPKPKTVSIKVNKKERRLALQTLLYNKKNNILVIENLLTSKTKSF
LDICVNLDQKVLIVVAEKTVPLKLATQNLKTVELILASNLNTFSLLNAKQILLTPLALT
TIKEIYMLKIRLKRFGRKKTPFYRIVLMENLSKRDGKCIRELGSYDPLRKILNFNRFELL
KAINEGAYPTNTARHLIYRMIMLIKYPSVTEKSINLYGNRQYTFIVDRNLRKIEIKYILEN
IFNITITKINTCILPIKQRRRPQYKKAYIKLKEGQQIDLFNMHIEKNEKNLIEVGDILRLG
YLIPEGDKERIQYYEGIVIAKQNRGINKSFTIRRTVQGIGIEQLFLVNSPKIISITKKQSSK
VRRAKLYFLRNLRSKSMNIKRKI

>Nitzschia_palea_NIES-2729_AP018511.1

MIKKEYPKLKKIQINRGLGLSAQNTNILKKNIEEFEKITGQKPLITRAKKAIAGFKIREN
MELGLSVTLRGEKMYAFLTKLLFFTFAQIRDFRGLSLRNFDKAGNYSFGLKEQLIFPEI
DYDDVDRTEGLTITIVLEQSSPKNGMILFKFLRFPLMVRVKRGNVARKRRKKILSLASG
YRGAHSILFRVANQQVMKALRYSYIGRKQKKRIFRRIWISRINATSRLISYSQLINKFKKS

NIDLNRKMLSQIAILDSSTFQSLIMSIRLYKSYTPGTRNRALSSFSEITKSKPEKSLVQKN
HRNKGRNNRGVITIRHRGGGHKRQYRLVDFKRNKYGIEGNVAAIEYDPNRNARIALIH
YTDGEKRYILHTKNLEVGNTIVSGLEAPFEVGNTLPLMNIPLGTSIHNIELIPNRGGQL
VRAAGTSAKILAKEGDYVTLRLPSKEIRLLRKECFATVGEVSNNDAFLVQSGKAGRTR
WLGKRPTVRGSVMNPCDHPHGGGEGRAPIGRTRPLTPWGKPALGIKTRKRKKSYILR
RMSRIGKLPITIPDTVTVNYSDSQIIVKGKFGTLETNIPETIGINSLLKISRELRSLHGLYR
TLINNMIIGVSEQFQLTLVLKGVGYRAAVQGKEITLSLGYSHPVKIEIPETISVEVVQNT
TINLKSCDKEKLGLFASNIRAWRRPEPYKGKGILYKNEQIIRKAGKAGKFTFLPIQNNR
NWYLIDCKGQTLGRLATTITSLLKGKVKSQYHPSVDTGDYIILVNADSIIIETSKHYLVY
NPGRPGHSLKNVSDCLPKLTIERAVKRMLSETSKRLMRRLRIYNDQNHHHQAQNPIEI
MAKKITALIKLALPAGKATPAPPVGPALGQHGVNIAAFCKEYNARTTDKIGLVIPVEIS
VYEDRSYTFILKTPPASVLLVNAAKIKKGSSTPNRVSVGSITKAQLEEIATIKLPDLNTT
KLSSAMKIVEGTARNMGISIMLSPKRTKYRKYHRGRMKGTASRGNEICFGNFGLQALE
PTWITSRQIEAARRTITRYTKRGAKLWIRIFPDKTVTARAAESRMGSGKGAVDYWVAV
VRPGTIIFEISSVPEEVAKAALNLASYKLPLKTKFINKMIYPQTMLTVADNTGAKKVMC
IRVLGGNKKYATIGDTIIAVVKEAIPNMPVKRSDVVKAIVVRTKKTIRRSDGMYIRFDD
NAAVIVNADSNPRGTRVFGPVAREIRDKNFSKIVSLAPEVLMYAIVEISGRQFWIETGK
YYDFNRIPTGKQITLNRVLLVNKLLIGKPYLEVIKGKILEHLRSKKTIVYKMRPKKKTRK
KQGHRQELTRVFIMVKAVAKYIRMSPHKIRRVIDQIRGRSYQEALMILEFLPYQAGGPI
WQVVHSAAANAKHNYGLDKKKLVINEIFADEGPKLKRIRPRAQGRAYKILKPTCHITV
VMMPKSDIHPTWSKDTPVLWDGKPLCFVGSTKKELQVDIWLANHPFYTKSQVVVDS
EGRVERFMKKYGLMAHKKGAGSTKNGRDSNAKRLGVKRFGGEKVRAGNILIRQRGM
KFKPGSNVGCGKDFTLFALVDGTIKFDVNIVMKVRPSVKKMCDKCRLIKRHGKVMVI
CSNPKHKQRQGMAVPKKRTSKSKKNARKANWKRKGYKAAQKSLSLAKSMLKTTSFV
YMIKCLKSEKEHGQFLINSLRPGQGITIGNQLRRVLLNDLGGVAISAVRIAGISHEFSTI
PGVREDILEILLNLKEIVLRSKFGRLKIQGPAVVTADLIQLPSTIEIVNSNQYIMTISTSNI
VEIEFKFESGTGYKLAYLQLDAIFMPIQKVDFKIENLTERLLLDIWTNGSISPNEAIKMA
AEITIDLFTALIAIEELQLSVRAYNCLKKAQINTVGDLLQYSPLQELFGRKSADEVFFTL
KNQNTLIGKKQLRQLLAWSFTNYDSMQACALADELKYLGFKYASQAGISISIEDLKIPF
VKNVMLEKANKEIRNAEKIYLKGKITDVERFQKIIDTWSLTSESLKDQIIYFFKNYDPLN
SVYIMAFSGARGNLSQVRQLVGMRGLMSDPSGQIMNLPIKKNFREGLTITDYLMSGY
GARKGIVDTALKTANSGYLTRRLIDVAQDILIREKDCLTTHSFLFVIGRVLSKSVFLAPK
NTQITYELIKQFQHVTNISIRSPLTCNLYRAICQKCYGWDLASENLVDIGEAIGILAGQS
IGEPGTQLTMRTFHTGGIFTEARQQIISPLNGIIQFYKTLETVILRTNRGEDVLVTKNS
GSLVLINIVEIEVLRNTILFPKNNQYILKDTVIGELINKNKQILSDRGGEIFINNLIWILSG
QFYNSPHNFYPDYKLNKSYIFRTKLVNHYSGFVNNYLLEGGLILSEEFHEVNCEILLVG

DFIFELIPGLFSKTSGLVMIVIKTIVIKSGKLYYPGENIFSNIIVKTLSFCELVRPIEIYEFLN
LISNALNFIQSCFLIQENQFLSNHTILGYLEAITNSLEIVKFKVKTKQIFLISNNDCLTIKK
SETKKDFINLAKTGKIIIENNTFFTLQKGRPYFFQDVNSVFCKNTEFVEAGKTIGLLNL
EKEITGDIVQGLPRIEEILEARKKNTSQKKVQKLGTSIQINPHKLLKVYFNYYEGSYKSF
KKVQLFILNSVQSVYQSQGVTINDKHLEVIIKQMTTKVLITYEGETPLLRREVIDLYHI
QYINQILEMEYYIPLLLGITKAALNNPSFISAASFQETTRVLTKAAIEGRIDWLRGLKENI
IIGHLIPSGTGMYINALPDFLAMQRVSFCWFITQGLNEELALFSKIQDFQNTEYIMFSE
EYSLIKPPYSLLIARKYSGNYKAQLVIPIEVRNKTIRYQNQFPIITLPLMTTYATFIINGCE
RVIVSQIIRSPGIYFLYSATLIPEYGSWIRFSFQKVIIQLDKINQKPVLSLLKEMGLTEIHQ
NLQYADFFYFSEFSRIFDPNYYRLGRVGRLKINNRLNLQLSERLQIITYEDIFAITDKLIN
LTISKDDIDHLKNRRVRSVGELLQNLFRIGFQRLVRKLRNQTNKFIVATIREFFGSSQLS
QYLDQTNPLSSLTHRRRISGLGPGGFDRDRISFAVRDIHPSHYGRICPIETPEGQNVGL
IASLTTCARVNKLGFLETPFWRVINGKVIKTGNPIYLTADIEDLYKIAPADIATNYLVKN
TIPIRYKQDFINVSPAEVDFIAISTVQVVSVAASLIPFFEHDDANRALMGSNMQRQSVP
LIFPQKPIVGTGLENQIAVDSGMTLNAQVSGIVSSVTANKIIIKYKLQKYLRSNQQTCIN
HRPIVWKGESIKSGQILTDGPAITNSELALGQNLLVAYMPWQGYNFEDAILISERLVYN
DVFTSIHIERYKIEIDRNSEMSETKMIPNLVKHLNDDGIVMVGTFVKPGDILVGKVISN
NTAEQLPESKLLRAIFVKDNSYRMPVIETVTFNRVFIAQIRKIQVGDKIAGRHGNKGIIS
RILARQDMPFLPDGTPIDIILNPLGVPSRMNVGQLYECLLGLAGDKLNSRFKILPFDE
MYGLEMSRILINKKLRQASRNQSWLFNPYAPGKMVLIDGRTGKEFENPITVGNAYML
KLIHLVDDKIHARATGPYSLITQQPLRGKAQHGGQRFGEMEVWALEGFGAAFTLKEL
LTIKSDDMQGRNETLNAIPQFGIPESFKVLLQELRSIGLDMSTYKIEKFSEIEVNLIMFS
YIKIKLASSFRILQWANRKLP-
NGFVGEVQKSETINYRTFKPEMDGLFCERIFGPSKSLECACGKYKICERCGVELTESRV
RRHRMGYINLIYPVTHVWYINSRPNFMALLLIKRIKGAPLIKRELEKLNLQSEIFRTRCF
VLVCTKIREQAIKRIRILENLIGTGSRPSWMILSILPVIPPALRPMIQLEGGRFATSDLNE
LYRRIITRNNRLLRLLEIDAPQLIIRNEKRLLQEAVDTLIDNGKRGKIALSANNRPLKSLS
DIIKGKHGRFRQNLLGKRVDYSGRSVIVVGPSLKLNQCGLPYEMAIELFQPFVILINQG
AKLIQQNLIIEPVLKILANHPIFLNRAPTLHRLGIQAFEPILVQGRAIKLHPLVCSAFNA
DFDGDQMAVHVPLSLEAQAECYMLMLAPYNFLSPANGEPIIMPSQDMVLGCYYLTV
NNIKNLNGSNHYFSSLDDVILAYSQNKIEVHSSIWVRYILVKYLKTTTGRVILNYTIQKT
LMSRYRGPKLKISRRLGLPGLTTKKSKKLTSKKTEYGIRLEEKQKLKFNYGLTESQLFR
YVKEARRRKGVTGLILLQLLEMRLDTLCFTLGFASSMAQARQLVNHGHITVNNKVINI
PSFQCRLNDTISVKEKSSSKNLIEANLIPSNLKFDSNSLKATLLDYCNREDIPLQLDELL
VIEYYSRMGQKTHPLGFRLGITQEHKSTWYANFNQYPSVLEEDDKIRTYINSIISNLNIS
RNDQIEVTIETGKPILVVLNLKKILRQVTIKVFEVEKVDLDAKLLADLVAEQLEKRIAFR

RAIREALQRAKNGIKIQVSGRLNGAEIARSEWIREGRVPLQTLRADIDYATSEANTIYGV
LGIKVWLFRMVTDTISDMLTRIRNATMVKHQIVQIPVTKMSLAITTILKEEGFIEDFEP
YKREYLLLSLKYTGKSRDSVINQINRISKPGLRVYKKSNELPKVLDDLGIAIVSTSKGVM
TNVKAKTLGIGGEVLCYIWMFVERLIKISRVSKVTKGGKKLSFRAIVVVGDENGKVGV
GVAKADDVVNAFKKAKTDGRKNLIELPLTKSLSIPHNVVGNFGACKVIMRPSIEGSGV
IAGGAVRIVLEVAGVKNVIAKQLGSNNLLNNARASVSALNNLMSRRNISKKRFPEPDSI
YNSYLVSLLISRILKSGKKTIAKNIVYEAFEIIKQKTNEDPLKIFEKAIRKASPIVEVKARRI
GGSTYQVPIEVSGFRATNLSLRWIIQYSKQRVGRSMSIKLANEIIDTANEIGNTIKKKEE
THKMAEANKAFAHFRRMIRVRLESFNHELLVASCKKISETLSNASLNSVGLVTLPTSKR
IYCVLRSPHIDKDSREHFEVRVHKRVLEIHYDSPIFDLLTDLAPGVFYRIMPTIQQLVRS
RRIQIKKKTKSPALVNCPQRRGVCTRVYTTTPKKPNSAIRKVARVRLTSGFEVTAYIPG
IGHNLQEHSVVLIRGGRVKDLPGVRYHIIRGALDTGGVKNRTQGRSKYGVKKPKMKK
DKSSFTSGVVHIQSTFNNTIVTITNLTGDTVSWASAGSSGFKGARKSTPFAAQTAAEK
AALIALSTGMKNVEILVKGQGSGRETAIRAIEGAGFEIKSIQDITSVPHNGCRPPKRRRV
MVRLVGVDLPRNKKIAYALTYIHGIGLTTAKKLIELAEIDDLRVYDLTTEQAVALRQTI
EDLKLEGDLRRFTGLNIKRLNEINCYRGKRHRNSLPVRGQRTRTNARSRRGAKKTKK
MPLKQQVGIVISNKMQKTIVVKIENRYPHPIYSKTLVKTKKYLAHDEQEQCNIGDQVL
VQECRPLSKRKRWKLIMAKKSMIEREKKRIRLEKKYSMKRKALLLEYQTALSFTSKIEIH
SKLQQLPRNSAKTRIRNRCWKTGRPRGVFRDFGISRHVFREMAHQCLLPGVTKSSWM
TRSLKKSPFVAYHLLKKINKMKKDTITTWSRSSTILPSMVGFTIAVYNGKQHVPVFISD
QYVGHKLGEFVSTRNFKTHKADRKGKRMNKSAKKRIEINKRNRLRNKYYKTSVRTLIK
LFFANLEIYVQDILNSVYSLMDKGTKKNIFHKNLAARKKAKLVASLKAMTQIFDETGNI
IPVTILKVGPCVVTQVKTEAKDGYNSIQVGYSRSKSLTQPELGHLQKSNIQPLKHLKEF
RITNQNFEVGQTLTVDLLSLGQFVDIQGKTIGKGFTGLQKRHNFARGPMTHGSKNH
RAPGSIGMGTTPGRVLPGKRMSGQVGNKVITVKKLKVIQFNLEENILVIKGSVPGKPG
NLLSIVMAKNKGTRILITLECTECRSNNKRSAGVSRYSTQKNRRNNPQRLELKKYCPH
CNKPTLHKEIKMTQIFDETGNIIPVTILKVGPCVVTQVKTEAKDGYNSIQVGYSRSKSL
TQPELGHLQKSNIQPLKHLKEFRITNQNFEVGQTLTVDLLSLGQFVDIQGKTIGKGF
TGLQKRHNFARGPMTHGSKNHRAPGSIGMGTTPGRVLPGKRMSGQVGNKVITVKK
LKVIQFNLEENILVIKGSVPGKPGNLLSIVMTKKIKFNIFI-
NGIIELTILKTGNYLIFENYLKFLKLKKNYLSSTKTKSEVKGGGRKPWKQKGLGRARVG
SIRSPLWRGGGITFGPKPKKISLKINKKEKQLALQILFYNRKNNILIIKDIFNIKTKNCLK
LFIDINKKILIIVSKNLINLKLSVKNLKNVKLVSFLNLNVLNLLKYKQIIMSLSSLDLLTTL
-
MLKLRLKRIGRKRSPSYRLVIMENATRRDGRPIEEVGYYDPISKKYKFDSEKIQKWLKD
GVQPTQTVLNLLKKIIMIIKYPIITDKATRLLEKNQYSFIVDRYSDKITIKAAIEYLFNVK

VIKVNTCRLPRKQKRKPQYKKAIVSLKEGDVINLFTMNVEKNNLPTLQIGDNVKVGV
KIIEGNKERVQFYEGTIIAKKNSSINTTITVRKTLQGIGIERIFLIHSPKVDSITVLRSSKVR
RSKLYYLRDLKGKASRLKQTF
>Nitzschia_sp._IriIs04_LC028895.1
MLKSEYPKIEKIILTRGIGLSSKNKELLNNHIFEFETITGQKPIITKAKRSISGFKIHKNLEI
GLSVTLRNKKMYAFLTKLLFFTFPQIKDFKGLSIRNFDKAGNYHFGINNQFIFPEINYN
HESQDLGFTISIILNQSNKKNNLILFKFLRFPFMIRVKKGTVGRRKHKKILLLAKGYKGA
HSSLFKIAKQQVLKGLKKSYIDRKLKKRFFRKIWILRLNAIAKFLNYNIFLFNLKKMNIQ
LNRKILSQISIYDFITLKNIVMNYKLYRT----
TINKNPFIFSNLTKIKPEKSLIIKNHQKKGRNHRGTITIRHRGGGHKQRYRIIDFKRLKY
NIKGIVKSIEYDPNRNANISLINYIDGIKKYILYPRNLKIGDTILSGLNAPLNIGSTLPLDK
IPAGKNIHNIELIPGKGGQLVRAAGTYARILAKNTHYVIVKLPSKEIRFLNKNCFATIGKI
GNENFHKYKKRKAGQIRWLGKRPTVRGTVMNPCDHPHGGGEGRTPIGRRNPKTPW
GKPALGIKTRKKKKSYILKKMSRIGKKLISIPNNVIVVSYKNFIIVKGKYGILVKKIPLELKI
DNSLIITKYFKSLYGLYRMLIFNMIKGVSVLFSKTLYLTGVGYKASIKEKKLILYLGYSHII
EHIIPKDIFIEIIKNNIIVLKSIDNEKLGIFAAKIKANRYPDPYKGIGILFKNEKINYKSGKV
GKITFLPNPKNRNWFILDCKYKSLGHISTFVVSLLLGKIKFDYYPSIDNGDYIILINTKYII
LKVKQYLFVNKPGHPGTSLKKYSDKILHLLVRKSIKGMLAP-
KKFLIKRLKIENDSYHKYSAQNPIYIMIKKIIASIKLELYAGKASPSSSIGPILGQYGVNIAL
FCKEYNEKTKNDLGFIVPIEISIYSDRSYSFILKTPPTSFLLLKELKIEKGSSEPNKQIIGYI
TTDTLKKIAKIKLNDLNTKKLNKAINIIKGTATAMGIIIMLYPKKLKYSKYHRGTLKGK
NYQENKLYFGKFGLLALEATWVTTNQLESVRKVISNSTQREAKLWFRIFPDKPITLRSS
ESRMGSGKGSLDHWVSPVKLGTIILEIDYVSKEIAKNSLKLAAHKLPMKLEFISKMIYPQ
TILNIADNTGVKKIMCIRVLNGSKKYAKLGDIIVGVVKEST-
NKLIKKSEIVQAVIVRTKKTINRYDGTYIRFDDNAAVIINSDKNPKGSRILGPIAYEVRKK
KFSKIISLASDILMYAIIEVNGRQFWIEEKNFYDFNRINLNKTIYFKKILLYRIILLGQPYLS
VIKGKILKHLLKKKILIYKFKSKKNYRRKKGYRHKITRIFIMSTFTIKYIKMSPYKLRRVA
NQLKNCSYKQALKILEFSSYRAAFPLWKTLNSFVSNIIYQQNIKVDNIFIEQIFINEGPKQ
KKFRIASRGRIHKILKSTSHITMTLMIKKKI--
KWISNSIVIFNGKILCLIGSTKSKLKIDVCLLNHPFYSKNKAISNSEGRIEKFMTKYKLMA
QKKGAGSTQNGRDSNKKRLGIKKFGGEKVLIGNILIRQRGMKFKPGLNVGCGKDFTL
FALSNGIVKFSIHIIMKVRSSVKKICKNCNVIKRKKKIMIICINLKHKQRQKMAVPKKRVS
KSKKRIHRKNWYKKSYKKIFKAISLSKSNINKIKLTLMIKCIKLQKEYGQFLITLTKSGQG
ITIGNSLRRILLNDLGGLAISSVRISNVKHEFSIIPGVREDILNVLLNLKGIIFKNKFGRLK
EKGPCVITASSFKLPNHIEIINSKHYIMTISESNVVEIEIKIEYGTGYKLAFLQIDSIFMPVK
KVNFKVENVKERLFLEIWTNGSITPNFALLSAAKIIINLFFNIIPIEQLELSKQTYNKLKTF

KINTVYDLLNFPYLVRFFDTTTIKEIKHTLKKNNSLIGKKEISQLLGWSFSTYNFVKTYC
LADELKLLGFKYSTKSGISLSIEDLQVTYNKLQFIKHINNFLLTIEKEYLENIITESERYQK
IVVLWDIFNNSLKDQTLTFFNNYDPFNSVYVMSSSGARGNLLQVKQLIAMRGLISNQK
GQLVNLPILKNFQEGLTITDYLMSGYGARKGVIDTSLKTATSGYLTRRLVEVAHNILIR
EKDCKSKYSLTVLIGCILNKPVLLIKKNTIITSKIIQFLNIIKKIYIRSPLTCILYNSICQKCY
GWNLANETLIDIGIAVGVLAGQSIGEPGTQLTMRTFHTGGVVNIDKKKIHYNHIGIIQ
YNKNLKIKKIRLNSGDFVNITTKEGSIILIKIKKLIIPKNTILFLKNNQYINNNNAIGELEN
RKKKILTKSSGEIKSNSLTWILEGELYNTFNNFYPDYKINKNFVFRSKIINLTNIFINFLVL
NNGSI------KTTKNIILINKFYFKIFQ-----------------IIKKKKLYYPGEIF---------
KFKKTFYKLRIYEIFNFIKIKLNILNICFLVKNYQYLSKFSLLSYLEFITNTEEIVKFKIKIKQ
LFIIYNKNCFIVNKLTNKNPILKHMNTGKILFSNKNYLIIQKGLLYITKQKNQFFYKDK
DFLNKNQLLGFILIEKNIINDIIQGLPLIEKYLEIKFKNKNNKQFLKFNFNFNINVHLLLY
NYFNYFTASYNSFKELQFYLLKNIQSIYQSQGVFINDKHLKIIITNMTSNVLVLNIGDSP
LIKYEIIDLYHANYINNTLIINIYVPILQGITKISLNNKSFLAASSFQSTISVLMKASLEGKI
DWLQGLKENIIVGNSIPAGTRMNSYIFNDFLKYQKESFCWFIYIGLDNQLNNISGLNIIP
IINIKIYSEESR-KLP-
IDPLLFQGFNKYSILEIKIPIEIYFKKILIKKDALIIDLPIMNSYGSFMINGCERVIVSQIIRSS
GIYFQISATLIPDYGSWIHFKLKQIFIKINEYLNFLIAYFNKSAFFNNSTLKLQN--------
YLNLFNKKNYSLGKFGRLKINDNLKLNL--
NFKNITYYDIQHIINKLELFSFYNDDIYHLKYKRIRFVGELLNNICNIGFKRILDDFIYEIS
ESAVSSINEFFTSSQLSQFLDQINPISYISHLRKISYFGPGGLKTEGIFFDIRDIHPSHYGKI
CIIETAEGKNVGLNLSLAITARINKLGFLESPFWRVINGKVINWGNPIYLTSELEDEYKIA
PADTLINYIEKDYITVRYKQNFINSLVSEIDFISITSLQTFSVAASLIPFFEHNDANRVLM
GSNMQRQSVPLLFPQKAIVGTGLEYQIAINSGMSLIASTNGIVENVTSNRISVKYKLSKY
VRSNQKTSLNQSPNIWKGEKIVKGQSITNGPAILDSELALGQNILVAYMPWNGYNFE
DAILINERLVYEDIFSSIHIEIYSIKVNPNKIQPEMKIHDTLIERLDNNGIIKIGTFVTSGDI
LVNKAIYISKEFQDPFVYLFNLLLIKDSSLTLPVIDIKVINQVIIAQIRKIEIGDKMAGRHG
NKGIVSKIVSRYDMPFLPDGTAIDIVLNPLGVPSRMNVGQLYECLLGLAGDKLNKRFK
ILPFDETFGLQTSRILINKKLRLASNNESWLFNPYTPGKLVLLDGRTGLQFENPITVGV
SYMLKLIHLAEDKIHSRSINTYKLDTQQPLRGKSNNGGQRFGEMEIWALEAFGAAFTL
KELITIKSDDVYGRSKVIFNITKFDISESLKVLIQELKALGLDISLMAFNNFINFEYII-
MFTSIRIKLASPLKILKWSYRKNG-
NNYVGLLKTSLGLDKDTLLPCRNGIFCDQIFGPISNYECVCGKYKSCDVCGVEINDSK
LRSHRMGCIKLLYPVAHFWYLKSFPNIINLLLIKSINGSSLLYKELKILDLNSELYKTKGF
LFLYNKLQRLAIQRIRILENLIGSGDPSWMILTLLPVIPPTLRALVQLEGGKFITSELNQ
LYQTIISRNIRLYYLIKLNSPWGMIKQSKRLLQEAVDSLIDNKKSNQTFLNSNNQPLKSL

SDIIKGKYGRLRQDLLGKRVNFSGRSVIVVEPKLKLIECGLPYNLAIELFKFFIVLNNNR
QEIFKFKALIIPILNLFKNYPIFINRAPTLHRLGVQAFKPILISGKAIKIHPLVCTAFNADF
DGDQMAIHIPISLESKIECYTLMLSSKNLLSPSNKNSIITPSQDMILGFSYLTLDNIRNLD
GANHYFINFEDALLAYYQNQLELHSSIWIKIILAQYLKTTIGRILLNYTIDQSLMSIFLSS
KFKVIRRLGLPGLTLKKINQKSNSKTEYMIQLKEKQKLKFNYILNENQLIKYIKQSKKKK
GITSLILLQLLEMRLDSICFNLGFAHSIMQARQIISHNHILINNHKINISSFQCKPNDVIG
VKKNSKSKYLINNNLIPFHIKFNSNLYQAIILDYCTKKDIQLNLNELSVIEYYSKMGQKI
NSLGFRLGKIKKHKSIWYSKFNNYQTILKEDIFIRQYLENLFLNVLIFRENKISIIVEVSNL
TIDIINIKKKLKKILIKFSEIENIFKNSKAITYLIINQLQKRISYKKIINNIFSKIKNGIKISIAG
RINGAELARYEWFKKGRIPLQTLRADIDFNFSKLHTSYGILGIKVWIFKMY--
IISHLINQIKQSIFLNHKILRVYKTKKSYFLVNILYKEGFIQNFEEINKKFLLIYLD-----
KIKQFKNIKVISKPGVRIYQKRKELKNFHIKKELMILSTSKGIITNKKAKYLNIGGEILFYI
YMFIEKIIKIRKVMHVLKKGKKINFQAIVITGNQKGKLGLGIAKGNDTNSAISKAKNKS
YKNLFEIPLTKSNSIFTSVIGEHSSAKILLKPSIEGFGIIAGGSTKLILQIAGIKNISAKQIGS
RNLLNNSYASINALKNLMPRKKIIKNKFPLLDYKYNSYLISLFILQILKSGKKNLARNIVY
NTCKIIKNQTNENPLYILEKAVINISPIVGIKSKNYKKSIVKIPQKIDYIRATKLSLKWLIKF
SKENKKKTMSLNLAYEIIKASKKLGNSFKEKQKIHKIAKSNKKFLYLKRMIKIKLEAFEYS
ALYLSEKKFINTILKNSLARLSIINLPVEKKIYCVLRSPHTDKDSREHFEMKKHKKIIYIFY
KSSIINLLQDLKPGVSCSIMPTIKQLIRLRRKKNIKKSKAPALINCPQRRGICIRAFTVNP
KKPNSAIRKVARIRLTSGFEVTAYIPGIGHNIQEHSIVLIRGGRVKDLPGVKYHIIRGVLD
SVGVLNRKTSRSKYGTKKLKMKNLKINNDEIILNIHSTRNNTIIIFSTINGKILFWSSGGR
VGFKNSKKKTAFASQLSLETLLKKNNILYKRKIHVILKGKGPGREPAIRVILKSKLKIKSF
KEITAIPFNGCRLPKLRRLMLKIIGINLPENKKVLYGLVNIYGIGNSIANKIITDLKIPNLK
IFELKDSKIKQIKEYL-
NLKLEGDLKSFHKLNIKRLIEINNYKGQRHLKKLPVRGQRTKTNAKTRRELKQKPNM
TINHKIGIVINKKMEKTVSVIVESKYKHKKYFKILVKLKKYLVHDEDNRCSIGDKILFKQ
CKPISKKKCWTLIMSKKNMIEREKKKFFLIKKYSLKRKNLLFEYKKQLNFSNKILVHSKL
QKCPKNSAKVRLRKICWKTGRSRGILRDFGLSRHIFREMAHNGLLPGIVKSSWMVKSS
KKNPFIAYFLLKKIVKSENKVILTWSRSSTILPFMVGCTIGVYNGKQHIPVYISSKLIGHK
LGEFVTTRKFRTHKLDKKKNKM----
KKSILLNNKKKLNNYYYLKNLKILQKKFNILLKNYLLVNLNYLYSFLDKSVKKNLLKKN
KASKKKAFFIKKLKTMTHLYDKFGFINPITILKIGPCIITNIKSIHKSNNILIQIGYKENNN
LTKSKLGQFKKFKIKPLKYLKEFSIKKNNFKIGQLLTLNNLSIGQSLNVTSQSSGKGFSG
LQKKHQFKLGPKTHGSKNYRKPGSIGMGTTPGRVLPGKKMSGQLGNKSVTVQNLK
VIKIELQKNLLFLKGSVPGKFGTLLSISM-
RKKKIRKIIILECTDCYSNNKRTYGISRYITEKNKQKTIKRLSLKKYCKFCNKSSIHKEIK

MTHLYDKFGFINPITILKIGPCIITNIKSIHKSNNILIQIGYKENNNLTKSKLGQFKKFKIK
PLKYLKEFSIKKNNFKIGQLLTLNNLSIGQSLNVTSQSSGKGFSGLQKKHQFKLGPKT
HGSKNYRKPGSIGMGTTPGRVLPGKKMSGQLGNKSVTVQNLKVIKIELQKNLLFLKG
SVPGKFGTLLSISMKKHINYRTVFLGKIIELEILKSGNYLVFKGLRKYYKLHYSKISSTKT
RGKVSGSGKKPWRQKGTGRARAGSIRSPLWRGGGVIFGPHPTINYSKLNKKEQKLIFQ
ILLYNKKTFTVIINNLYNIKTKEFLKICININKKILLIVSKKTIPLEKSINNIKTIKMVLDSEL
NSFDLLNTTQFLMTSNALLNIKKKLMLKLRLKRIGKKIQPIYRIVIANSKTKRNGKDIEK
IGYYNPIKKIFKINETLMKQWILKGAKPTKTVNNLLKKLFMLIKYPLITSKIRGSMKKNL
YSFLVDFNLNKKDIKNMIEYFFNVNIYKITLTTIPEKKICKSKYKKATIYIKAKKKRNIIH
MNL-NMNTKKINVGDIIDIKVIITEKDKKIYQFYTGIVIAKYK---
NISITVRKIIKGIGIEKIFLLDSPKIESINILKSLPFHKSKLYYLRNLKKKI-----KF
>Nitzschia_sp._NIES-3576_AP018508.1
MIKKEYPSIKKIQINRGLGLNAQNKKILKESINDFKMITGQTPIFTKTKKAISGFKTRRN
MVIGLTVTLRSKKMYSFLTKLILFTFSQIRNFHGLSLRSFDKAGNFTFGLKEQLIFPEIN
YNDNQNTQGFTITIVLNQKLPKNSVLLFKFLRFPLMVRVKRGNVLRKRHKKILSLSKG
YVGSHSRLFRTAKQQVMKALKYSYIDRKKKKRNFRKVWIIRINAMARLLSYNQLINFLK
KSNITLNRKMISQLNIINAIFMKQLIMVNYSYKPYTPGIRNRILISFFNINKIKPEKSLIKK
LHQKKGRNNRGIITSRHRGGGHKRRYRFIDFKQIKKNIKSTVVSIQYDPNRNANIALLQ
DIDGEKRYILHCKNLNIGDTL---
DNSSLNIGNTLPLKKIPIGKSIHNIELFPKKGRQLARAAGVSAKILAKNNKYVTLKLPSK
EIRLFNENCLATIGEVGNSAQNQIVSGKAGRIRWFGKRPTVRGAAMNPCDHPHGGGE
GKAPIGRSYPSTPWGKNTLGVKTRKKKKSYIIKNMSRVGKKPIIIPENVKIKFDKNFIIIE
GNFGILKKQIPEILIILNYLKLYKNSSSIHGLYRTLIANMIIGVSKKFFLVLILKGIGYRAFI
EKNKIILNLGYTHSIKIDIPENISINIEQNIKIQLSSIDKEKLGAFAAQIRSYRIPELYKGKGI
LYENEKILLKTVKTKKMTLFTTN-
SHIWYLIDCKNQSLGRLSTSITHILLKKNINTYSPSNKQKNYIVLINSKQLKHKKKIYYFC
YKPGHPGKSLKFF----
SSNTIKQTIKKMLPINRNKLIKQLKIYENEKHPHKGQININVMSKKIISVIKLKLQAGKA
TPAAPVGPVLGQQGINISAFCKEYNALTLNQIGTIVPVQIFIYEDKSYKFFIKTTPTSLL
LKNAINITKGSSTPNEKNTGIITKDQLKQIAVLKLNDLNTTNIDTAIKIIEGTAQSLGISI
MLSPKATKYNKYHYGHMKGKAIKGNKIVYGHLALQSLEPSWISSRQIETIRRVIIRHTK
RGVNLWIRIFPDKTVTSRASESRMGSGKGSVSHWVAVLKPGTIIIEINFTPLKIAMEALK
LANYKLPFKTKIITLMIYPQTILNIADNTGAKKLMCIRVLKKINKNAKVGDIIIGVVKEA
TPNTLIKRSDIVRAVIVRTKKTIMRTDGIHVRFDDNAAVIINNENNPRGTRIFGPIAYEI
RTNNFSKIVSLASEILMNAVIEISGRQHIIEKKKFYDFNNIKNRKYIFLNKVLYLYLVILGK
PYLNVIRGKIIRHLKSKKLLIYKMKRKKKMQKKQGHRQKLTRILI------------------------

--M----
NNKNWSYNSIVFYEKIPICLVGSTKKNIYIDTWIPNNSFYKKTQKFLNNEGRIKQFIKKY
KIMAHKKGAGSTKNGRDSNKKRLGVKHYHKEKIYAGNIIVRQRGTFLKPGFNVGCGK
DFTLFALVDGVINFKVTIIMKIRSSVKKICDKCRVIKRHGKIRVICLILKHKQRQKMAVP
KKKVSKMKRNLHRSAWKKKAFKKAQKALSIAKSNIKLKN-
LKMIEHLYSKKKYGSFLIKGLEINQGITIGNYFRRVLLNDIDGIAISGIRFSDITHEFSIIPG
VREDILEILLNVKGIILKGNVGRLKVQGPAIITAGSIILPKNIKIINPNHYIMTLSTSSYIEI
EFLIESGTGYKLSFLQLDAVFMPVQKVNFNVEINKEKLYIDIWTNGSISPVKAFLLATEI
SLNTFSAFMGLEKLDLSVRIYNCLKRANINTIGLLLKCSKLLNIFGEKSFEKVSEKLYNN
NNFIRKKDLHNLIKWTLSNYDIINTSLLIDKLKYLGFSYATQGAISISIEDLKVPANKKLL
LQKIESQIFKTENYYFEGKITEIERFQRILNLWEATNNFLKSQVIQYFKKYDPFNSVYIM
AFSGARGNITQVRQLIGMRGLMANQHGQIIDLPIVQNFQEGLTVTDYLISTYGARKG
LIDTALKTADSGYLTRRLVDVAQDIIVRENDCYTSSGLSFIVGRINCDNILLIKKNTEIT
LNIFNRLKKVTELKIRSPLTCKLYRSVCQKCYGWDLSNQNLVDIGSAVGILAGQSIGEP
GTQLTMRTFHTGGAVDIGTRSDSSPLTGVFQYCSDFEGLPFRTSIGSIGRIIKEAGSAIII
KILKLDLLPDDVIFLKNNQFISEGAPIGHLSQPEMLIRAIYSGEIELTNIIWVLSSKIIFSPK
NFYSDYKINKNSIFRSKINSNSNGLFNNFFLKGGLIINEEQYNINSRKIHIGNTISLIFGKK
KSRTSGLVQIKIANIKIKSGNLFFPGELNR-----
KTFLFFELKRFIEIYEIFNILSTDIKNLNISTLAQKKQYISIYTILSFLENITISIEIIKIKEKIKH
LSLILNSDCFQIKKSDIKSQYIKKNVIGKILFTTNDSYIIQKGMPYFFEMANLTYFNHGD
YILSGEILGFLNIKKRIANDIVQGLPQISAAFEARK-------
ALTMGIPLYINPHKFLQIYFTYYEAVYRSFKKVEFLILNFINETYKEQGVKINLKHFEIIIK
QMTGRVMITKSNDCDYFPGEIVSIYYIKYLISIFQQSIYVPLLFGISRTALSSPSFLSAASF
QETINVLKFAAIEGRVDWLRGLKENVITGNLLPVGSGMEAKIFPDMLEAQCHSFCWF
LSTGLKEEFSSVSKKYSYNDITYTILGGEYSLKLPKYGDSEKERLNFNNTIKIKVPIRIKIK
KNYIVIENLIIYFPMMTAYATFLVNGLERVILSQISRSPGIIFIYSAVLIPESGPWTVFKMK
EILLSSNRFSEISIFDTLRNIGLTEIYNNLTYSNWFYSKNFLKIFDPAQIPIGSIGRLKINNL
LNLPINESIISITYNDIFAIIDKFAEFMIKEDDKEHLKNKRIKLPGKILQDVIHRGFYRLIT
GLSRRNMTHIINSLSEFFTSSSLCQFLDQINPLSTLTHSRRISYYGNGGLKKGAVSLNVR
DIHPSQYGRICPVETVEGSNVGAVNSLAIYAKINKTGLLETPFWRVINGKVLKEANPLY
LTADIEDSYKIAAADTLINYLTDKYITVRYRQRFINVPPGEVDFLAISPIQIVSIAASLVPF
FEHNDANRVLMGSSMQRQSVPLLYSQKPIVGTGFETLVVTNSETILKSKSSGIVDFVSS
NKIIINYNLQKQAFSNQKTYVNQRPIVWKGETVKCNQSLTDGPSILDGELSLGRNLLV
AYMPWHGYNFEDAILINERLIYDNVFTSIHVEKYRTELGTNKNIKETKKIPKVTKNLDE
NGIIKVGTVVKSGDILAGKVALPPKKDERPISKLIRAAFSINMSLKLPIIRVSTSTTIWIVQ
IRKIQVGDKLAGRHGNKGVISKVLARQDMPFLPDGTPIDILLNPLGVPSRMNVGQLY

ECLLGLAGSNLNKRFKVLPFDEMYGPETSRILINKKLRQASQNKSWLFNPYMPGKMV
LIDGQTGIEFENPITVGNAYILKLIHLVDDKIHSRYIGPYSVITQQPVRGKALRGGQRF
GEMEVWALEGFGAAHSLRELLTIKSDNIIERDQSVSNIGNFGVPESFKVFLQELRAVGL
DIKTYKLKNFSSNEVQTLMFDYLKINLASPLEIMRWSHRYLP-
NGSIGEVLTNDTIDFKTRKVKYGGLFCQRIFGPIKKYQCGCGKYKVCEECGVEVINSL
ARRYKMGYIRLRFPILHFWYLKTEPNVFALLLIKDINGSSLIYKELKELNLHVEIQKTRD
LILECSEIRRRAITRIRLLENFVNTGSHPSWLVLTVLPVLAPELRPIRFLENGITIGSDLN
DAYRLIIMRNNRVDTLVQMGLPILFIRNERRGIQEAIDTLIDNKKRKRPHISINDSPLSSI
SDILRGKQGRLRQNLLGKRVNFSGRSVIVVEPKLKLNQCGLPYDMALELFKPFIILNKK
NQTLFRMDSEILPVLKILANHPIFLNRAPTLHRLGIQSFEPILIEGKAIKLHPSVCSAYNA
DFDGDQMAVHIPLTLEAQTESYNTMLAPYNMLSPATGEPTMTPTQDMILGCSYLTI
SNIKNLNGSNHYFSNLEDVLKAYFQNKIDLHSSIWVRYIMVQYLKTTAGRVIINYTISK
TLMSRYIGPKLKINRRLGLPGLTIKKSKKTKKSRTDYGLRLKEKQKLKFNYGLTEKQLS
SYMKKARKKKFFTSTILMQLLEMRLDNLCFTLGFAKTIQQARQLITHKHINVNKKIINI
PSFQCHRNDIISISNKKVSEDLVK-
NYYPSHIKFNNTLTEATILDYCRKADIQLTINELLVIEYYSRMGQKVHPIGFRLNINENY
KSLWYSNSKNYIEFLKEDNMIRIFFNNIISSIKIKRKNTITINIRTGNPYFLIKNIGQKLTQI
RIHITEIKKFFLETEIIANLIAEQIEKRIHFKRVIGNILQKVNIGIKILLSGRLNGAEIATNE
WVLKGSIPLQTLKININYSTAEASTIYGIIGIKVWMFKMLTDKISYILTKIKNAAKVKHE
TVIFPFTKNFLSISRILKQEGFIKDFRISK-KIIILL-
KYKRISNNSTFKEIKQISKPSLHIYTSFNDISKKIDQIGITLISTPKGILTNKEAKKLHIGG
EILCYILMLKERVIKITRVSKVTKGGRRMSFQAVIALGDKKGKIGIGIAKAKDTRDAIQK
AKSNGYKNLINFPITKSLSIHRSIIGKHKCSKILIKPAMNGSGIIAGGTARAILEMTGIKNI
VAKQLGSNNKLNNAYALFNGLKELMSRKKNLKKNYIEADSVYNNFLISLLISKVLKSGK
KSLANYIVYTMCSIIKAKTNKSPIDIIKKAIIKVTPKMELRDKKIRNRIKKVPIEVSSYRAT
YLALKWIIKFSKKRNEKTMSEKLAYEVLDILNNNSEIIKERKRIHRIAKSNKSFMYFKRMI
RIRIESYSYKSLVFAQKHIINTLKISSIFYISLVSLPIKKKIYCVLRSPHVDKDSREHFEIRTH
KKILDIYYDPVILDFLNNLSSEVSFCVMPTIQQLIRKNRTKTKNKTKSPALIKCPQRRGI
CARVFVTNPKKPNSAIRKVARIRLSSGFEVTAYIPGIGHTIQEHSIVLIRGGRVKDLPGV
KYHIIRGTLDVGSVQNRRQSRSKYGTKKPKMRGHITRFPKIIVYIHSTFNNTLVTLIRH
FGKTISWSSSGKSGFKNSKKKLSFAAQCTIDKLKSIMRKIAFNKVKVVVKGQGLGRETS
IRSIKNNGFLITSLQDRTPIAYNGCRSPKSRRLMIRILGVYLPENKKILYALTLIYGIGISR
ATQIIKSAKIESLKVYELKIKQINLLRQVLSYTLLENNLKRSTNLNIRRLIEIKSYRGRRHI
KRLPVRGQRTKTNASRKKKIKKTKK---

MAKKSMIEREKKRIFLEKKYSLKRKFLLIQYKNTKDIKDKFEIGLKIQKLPRNSSKIRIRNI

CWKTGRSRGFMRDFGLSRHIFRKMAHQGLLPGITKSSWMNRSLKKGPFVAYHLLKKI
KR-
RKEIIKTWSRSSTIIPSMVGFTIATYNGKRHIPIFITDQMIGHKLGEFVFTRFFRSHKSDK
KKKF--
MTQIFDGNGRVIPVSLLKVGPCIVTEINLYN----
NSVQIGYKSEKKIAKPVLGKLKKMNIEPLKHLKEFQVKNSIFKIGQILNLNIFKSEKYLTI
KGKTIGKGFTGLQKRHNFVRGPKSHGSKNYKKPGSIGMGTTPGRVFPHKKMSGQSG
NKMITIKNLEIIELNTKENILIVKGSVPGKNNNLLTIIMEKKKKARILVTLECKECNSEIK
RSKGISRYITKKNKQNTPEQLILKKYCKFCNKITVHKEIKMTQIFDGNGRVIPVSLLKV
GPCIVTEINLYN----
NSVQIGYKSEKKIAKPVLGKLKKMNIEPLKHLKEFQVKNSIFKIGQILNLNIFKSEKYLTI
KGKTIGKGFTGLQKRHNFVRGPKSHGSKNYKKPGSIGMGTTPGRVFPHKKMSGQSG
NKMITIKNLEIIELNTKENILIVKGSVPGKNNNLLTIIMKKNIEYKTTFLGKELGLKVLES
SNYLVFKGLRHYYNLYYGKISSTKTRGNVSGSGKKPWRQKSTGRARAGSTRSPLWRG
GGITFGPQPILRYTKINKKEQNLILQILLYNKKDSIIIINNLYTLKTKNFLRILIKLDKKIL
LVVSKKTIFLERTTKNIKNIKIILDSELDCFNLLTKEQFLITSTALSNIKKKLMLKLRLKR
VGKKHLPSYKLIIIKDKKKRSGKHIEILGYYNPFLKTYKFNITNTYKWLDKGIIPNKKTF
YLLKKYIMKIKYPYITSKIRQCIYGNQYCFLVNSSINKKDIKVNIEKFFGVNITKINTIKKP
AKKKHTNKYKKIIIQLKRNQNIKYFYMQIENKKLPSLNVGNTVKIFLEVFEGNKKRIQQ
YSGIIIAKKNSSINTTITVRKITNQIGIEKIFLIYSPKIIKIEVLKSVYIRKSKLYFLRNLKKKYI
SLKKLD
>Nitzschia_sp._PL1-4_AP018506.1
MIKQEYPSIKKIKINCGLGLDAQNKNILKENISNFENITGQKPIFTKSKKAISGFKTRKN
MIIGLTVTLRGKKMYDFLTKLLLFAFSQIRNFRGLSLRNFDKAGNFTFGLKEQLIFPEI
DYNNIKNLQGFTVTIILNQNLPKNGIILFKFLRFPLMVRVKRGNVSQKRHKKVLALAK
GYIGSHSRLFRVAQQQVMKALKYSYSDRKKKKRELRRIWIIRINAMVYSLSYNKAIHFFK
KNNIFLNRKMLSHLSIFNTIFLKQLIMIIHSYKPYTPGIRNRFLVNFTSISKIKPEKSLIKKL
HRKKGRNNRGIITSRHRGGGHKRRYRLIDFKRIKKDTKSFVKSIEYDPNRSANIALIQYT
DGKKKYILHCKNLNIGDII---
DSTSIRIGNNLPLERIPIGKSIHNIELLPKKGGQLVRAAGTFAKILAKNNKYVTLRLPSKE
IRLFNKNCTATIGEVGNSEHNQIKSGKAGRIRWFGRRPKVRGTAMNPCDHPHGGGE
GKAPIGRVQPLTPWGKGTLGIKTRKKKKSYILKNMSRIGKKSIIIPKDVNIKLIKNLIIVE
GPFGILKRQIPDIFSILSNLQLIKKSSSMHGLYRTLILNMIIGVSKKFFIVLLLKGIGYRASI
EKDQIILNLGYTHLIKIDIPKDISIIIEQNTKILINSINKEKLGIFAAKIRNYRTPEPYKGKGI
LYQNEHIILKNIKIKKMTFFIHK-
PNSWYLIDCKNQSLGRISTYITQVLLGKNITTYSPSSKKKNYVVLINTNFLRYKKKLYFF

CYKPGQPGRALKKF----
SINTIKQTIKRMLPMSRNKLIKQLKIYENNIYPHKGQVNQKIMTKKLISIIKLNLQAGKA
TPAAPIGPILGQQGINLSNFCKEYNALTMNKIGTIIPVKLFIYEDKSYKINIRTTPTSFLL
KDAAKIIKASSIPNEKNCGVITQNQIKEIALLKMSDLNTNSIDKAIKIIKGTAQNMGISIM
LSPKSTKYKKYHYKRKKGKAVKGNKIVSGQLALQSLEFSWISSRQIETIRRVIVRHTKRG
VNIWIRIFPDITITNRANESRMGSGKGSVSYWVAALKPGTILIEINLIPIEIVKKALELVRY
KLPFKTKIINKMIYPQTILNVADNTGAKKLMCIKILKKNKRAAKIGDIIIGVVKEAIPNM
LIKRSDIVKAVIVRTKKTIIRLDGIHIRFDDNAAVIINNDKNPRGTRIFGPIASEIRTKNFS
KIVSLASEILMNAIIEIDGKQHLIEEKKFYDFNHIKNKNILLLNKILSIYFMLLGKPYLEIIK
GKIIKHLKGKKLFIYKMKKKKKMRKKQGYRQKLTRVLI-----------------------------------
--M--
NNKKKNWFSKSIIFYENIPICLIGSTKKKLFIDTWLANNSFYRKKQDLLNNEGRIEQFIK
KYKFMAHKKGAGSTKNGRDSNKKRLGIKHYHGEKVFAGNILVRQRGTRIKAGYNVG
YGKDFTLFALIDGVLNFNVTIIMKVRSSIKKICDKCRLIRRQGRLRVVCINLKHKQRQK
MAVPKKKVSKMKRNIHRSLWKKKAFKKAKKFLSIAKSNLKNNGFLPVLKYLKHEKDR
GYFIINGLRVNEGSTIGTQFRRVLLNDLVGNSITGVRFKGVEHQYSVI-
SAREDVLEILLNTQGVILKGNVGTLKMEGPGVVTASCIKFPKNLKIINPNHYLMSIPES
ASVDLEFLIERRTGYRLAYVAADATFMPVHRVTFHTRNILEELRLDITTDGSISPAEAFI
AGARLTINLYSTFIGIEILELPPKIRNCLKKSGIHRIDSLIESSELLNIFGQKSLDIVLDELY
NNNNFIGKKDLSNLIKWSVSKYNLLSTSLLIEKLKYLGFYYATQGSISISIEDLKTPANKS
FLLKKIEYEIFKIEKIHQKGKITEVERLQRILNLWEVTNEFLKNQVIQYFKTYDPLNSVYI
MAFSGARGNISQVRQLVGMRGLMADQQGQVIDMPIIKNFQEGLTITDYLISSYGARK
GLIDTALKTADSGYLTRKLIDVSQDIIIREKDCYTIYDLVFILGRILSRNIYLFRKNTQITS
KIFETIKLINKLYIRSPLTCNLYRSVCQKCYGWDLTNQNLIDIGSAIGILAGQSIGEPGT
QLTMRTFHTGGVVDIATEHDCSPLTGVFQFSNDFKGLPYRTNLGNIVRITKRISSAIIIE
IFKLRLLPNSIIFLKNNQFIKEGSPLGDLSPTIQHIRALFSGEIDITKLIWILSSKIFFTPKN
LYNDYRINKSSIFRSKINSISNGLLGNSFLKGGLIVNEELYLIYYKKILIGDYISLITKNLVS
TTSGLVQIKIATVRIKSGKLFFPGELDMNN---
REILFFELIRSIKLYEILSIASSNMEIINICSLAQNNQYINIYMILTYLENITNSIEVIRIKNKI
KHLSLISNEDCFKLDKSDRKGNYIESNKIGKILFKNKTHYIVQKGMPYFFEATNLIFFN
QGDYILSGEIIGFLNLEKRIANDITQGLPQIDAALEGQVKQLNRTEAQHMGLPLEINP
HEFLKIYFTYYDATYRSFKKVELLILNFVNNTYKSEGVFINIKHFEVIIKQMTTKVLITDS
GSTSYYPQEIIDFYHLRKILSILKIKGYIPLLYGITKSSLNNPSFLSAASFQETTTILKKAAL
EGKLDWLRGLKENIIVGNLLPIGSGMNNSIFSNMLEAHRHSFCWFLTYSLKEELYLVSK
ASPLPKTNYIILGDEYTLNIPRYKESNKERIDNYNIIIIQIPIILKVKKNYLKRENLNLYLPI
MTTYATFLINDCERALISQISRRPGIYFIYSAIIIPDQGSWIHFKIKYILIQFNKFFEISVFNL

FKKIGLTEIYKNLHYANWFYSINFLKIFDPVEVSLGSTGRSRINEQLDLPINENLICITYE
DLFSIIDRLIELIIREEDIDHLKNRRIKLPGEILHNIIKLGFYRLYKNMSKKGFFPILITLIEF
FNSSPLSQLLDQTNPLSSLTHKRRISYYGPGGLKKGAATFKIRDIHPSQYGKICPVETV
EGSNVGLVNSLATCTKINKLGLLETPFWRVINGRVLKEIDPIYLTADVEDLYKIAAADT
LVNYLVNNLITARYRQRFISVVPAEIDFLAISTVQVVSIAASLVPFFEHNDANRVLMGSS
MQRQSIPLLFAQKPIVGTGLENQVVNNSEMVLRSESDGIVDFVSSDKITIKYKLDKYSF
SNKKTCINQKPIVWKGERIKHGQVLTDGPSIIDNELSLGRNLLVAYMPWHGYNFEDAI
IINERLIYDNIFTSIHIEKYKIEISK---
KGETRKVPRKIENLDENGIIKIGVVVKPGDILVGKVSPFLKKKESPIFRLMRAALTIDES
LKLPVINTETYSSIHIAQMRRIQVGDKLAGRHGNKGVISKILARQDMPFLPDGTPIDIIL
NPLGVPSRMNVGQLYECLLGLAGDKLNKRFKVLPFDEMYGLESSRILINKKLREASQN
KSWLFNPYMPGKMVLIDGRTGTEFENPITVGNAYILKLIHMVEDKIHSRFIGPYSRVT
QQPSQGKSLEGGQRFGEMEVWALEGFGATYTLREVLTIKSDAIDERESSLKKIIDFGIP
ESFRVFLQELRALGLNLKTYKIETFSANEIQTQMFDFIKIKLASAAQILRWSHRKLP-
NGFVGEVKNPKAFHRRTFKDIPGGLFCQRIFGPIKDYECSCGKYMVCKKCGVEIISSNA
RRYRMGYIKLRFPVFHFWYLKSKPNIIALLLVKQIGGSSLIYKELKALNTNFEIFKTRCFI
LVCTKIRAQAIKRIRILENFSSTGSHPSWITLTVLPIMSPELRPVTLLEGGVMIFSDLNEI
YRLIITRNSRVDELAQGGIPRLFLRNERRGIQEAIDILIDNQNSTKIYRNTHDRPLSSLSD
ILRSKKGRLRQNLLGKRVNFSGRSVIVVEPTLKLTQCGLPYDMALELFKPFIILNKEFDS
LFREDSKILPLLKVLSTHPVYLNRAPTLHRLGIQSFEPVLVEGKAIKLHPSVCSAYNADF
DGDQMGVHIPLTFRARLESYNTMLAPFNLLSPSTGESIVTPSQDMVLGCSYLTVNNIK
NLNGSNHYFSDIEDALKAYSQNKIEIHSTIWIRYVLVQYLKTTVGRAILNYTIYKSLMSR
YRGPRLKINRRLGLPGLTIKKSKKKKFLRTDYGLRLEEKQKLKFNYGLTEKQLSNYMK
KSRKQGYLTSTVLLQLLEMRLDNICFILGFAKTIQHARQLIAHKHINVNKRIVNIPSFN
CQLKDTISITEKKISKDLISKNYYPSYLEFNNSLFEATILDYCKREDIQLPINELLVIEHYS
RMGQKIHPIGFRLSTSNNYQSFWYSKIKKYIKYLKEDNYIRQSIHNQISHITISRIKKIYIYI
KTNDIFFSIKDLTIILEIIDFKIFKIQKVYLDSGLIVNLIADQIEKRIHFKRIMNSMLQKIKI
GVKILLAGRLNGSEIARTEWVLRGSVPLQTIEKNIQYSTAEANTIFGIIGIKVWISKMITD
RISYIITRIKNAVTIKHEILIFSFTKISLSIFRIFKKEGFIKDFKISK-KLIILL-
KYEKISNKPMFKKIKQISKPSLRTYMSSNKLSKLLDHNGITLLSTSKGILTNKRAKELHL
GGEILCYIWMLKERIIRITRVSKVTKGGKRINFQAVIALGNEKGKIGIGIAKAKDTRDAI
EKAKTNGYKTIINFPVTKSLSINHSIIGEYKSSKILVKPAKPGSGILAGGSARIILEVAGIK
NVVAKQFGSNNRLNNAYALFNALKDSMSRKRYLKEEFIKPDSIYNNFLISLFISKILKSG
KKVLANNIVYETCSILKKHFKEDPINIIKEAIKLASPKIELKDKKIRNSIKKIPVEISSYRAT
YLALKWIIKYSKKRTEKTMSKRLANEILEIVKNNSRTIKEKRRFHKIAKSNKSFTYFKRMI
YIKLDSYSNKSLIFAQKQIFNVLKIFNIFNINIVSLPTSRKVYCVLRSPHVDKDSREHFET

RIYKKILVVNYFSNITNILNKFSPEILVRIMPTIQQLIRKNRKKKKNRIKSPALANCPQRR
GICSRVFTTSPKKPNSAIRKVARVRVSSGFEITAYIPGVGHNIQEHAIVLIRGGRAKDLP
GVRYHIIRGTLDAGSVINRRQSRSRYGTKKPRMGGKRTQFPKLFIYVKSTYNNTLITLT
HHYKKTISWSSAGKVGFKNSKKSLPFAAQCIINKVKGYIRRVAYNKVKVILKGQGPGRE
ASIRSIRHKGFIITTLQDISPLSYNGCRSPKPRRIMIRILGVNLPKEKKILYALTLIYGIGIA
RAKQIIKITKLDTLKVYELNFKQIQLLRQILYYTLLENNLKRFTQLNIRRLIEINSFRGKR
HRKGLPVRGQRTKTNANTRKKNNIKKK---

MAKKSMIEREKKRLYLEKKFSLERKILLLQYKNTKNIKKKIEIALKIQKLPRDSSKIRIRN
RCWKTGRPRGFLRDFGLSRHVFRNMAHQGLLPGIIKSSWMKRSLKKGPFIAYHLLKKI
KKIEEKIIKTWSRSSTVIPSMIGFIIGVYNGKQHIPILISDQMVGHKLGEFVNTRFFRSHK
SDKKK--
MLQIFDKDGSIIPVSLLRVGPCIVTKIDIYN----
NSVQIGYKPDKEITKPILGKFKKLNIKPLKYLKEFKVKNKDFKIGQIINLEIFSSKEFVTIR
GKTIGKGFTGLQKRYNFVRGPKTHGSKNYRKPGSIGMGTTPGRVFSNKKMSGHSGN
TIVAIKNLKIVKINLEENILAVKGSIPGKINNLLWIIMEKKKSNRILITLECNECSLENKQS
KGVSRYLTKKNKQTTPERLILRKYCKFCKKATLHKELRMLQIFDKDGSIIPVSLLRVGP
CIVTKIDIYN----
NSVQIGYKPDKEITKPILGKFKKLNIKPLKYLKEFKVKNKDFKIGQIINLEIFSSKEFVTIR
GKTIGKGFTGLQKRYNFVRGPKTHGSKNYRKPGSIGMGTTPGRVFSNKKMSGHSGN
TIVAIKNLKIVKINLEENILAVKGSIPGKINNLLWIIMKKYIYYNTVFLGKKIKLKILKSGN
YLVFKGIRKYYKSYYSKISSTKTRAKVAGSGKKPWKQKGTGRARAGSIRSPLWRGGGVI
FGPQPTTNYSKLNKKEQKLILQILLYNKKSFITIINNLYIIKTKEFLKICINLNKKTILIVSE
KTLALEKATKNIKNIKIILDSNLNCFNLLNNSQFLITSTALYNIKKRLMLKLRLKKVGKK
HYPSYKFVISESKKSRNGKHLDILGYYNPFLKNYKFNINIVQKWLFLGINPTKKTLYLLK
KNPMKIKYPYITSKTRQYIFVNQYCLLVNSSMNKKDIKINIESFFNIAITKINLIKIPYKKI
GKKNYKKVIIELKKAQSLKYFQMNYQEKRIPFLNIGDTIQIYLEIFEGNKKRIQQYTGILI
SKRNNSTNTTITVRKTINQIGVEKIFPIYSPKILKIEVLKSVYCRKSKLYFLRKLQGKKNL
FRQKN
>Nitzschia_sp._PL3-2_AP018504.1
MIRKEYPSIKKIQINRGLGLNAQNKAILKESINEFETITGQKPIFTKTKKAISGFKTRKN
MIIGLTITLRGKKMYSFLTKLILFTFSQIRNFHGLSIRSFDKAGNFTFGLKEQLIFPEIDY
KNNLNIQGFTINIVLNQKLPKNGILLFKFLRFPLMVRVKRGNVLRKRHKKILSLSKGYV
GAHSKLFKIAKQQVMKALKYSYIDRKKKKRNFRKLWIIRINAIARFISYNLIITTLKKNNI
NLNRKMLSQISIFYAIFFKQLIMTIYSYKPYTPGIRNKILVNNINTTKIKPEKSLIKKLHRK
KGRNNRGIITSRHRGGGHKRRYRVIDFKRLKKNITSIVASIEYDPNRNANIALLHDSDG

EKRYILHCKDLNVGDNL---
NNLTLNNGNTLPLKKIPIGKSIHNIELFPNKGGQLVRSAGTFAKILAKNNNYVTLQLPS
KEIRLFNENCLATIGEIGNSEQNQIISGKAGRTRWFGRRPSVRGTAMNPCDHPHGGG
EGKAPIGRVHPLTPWGKNTLGVKTRRKKKSYIIQNMSRVGKKIINIPKNIKIEIDNNLIII
EGNYGILKKQIPNIFIILNYLQLNKKSSSIYGLYRTLILNMILGVSKKFFLILILKGIGYRAS
LNENQIILNIGYTHPIKIEIPKIISIKIEQNIKIELSSIDKEKLGTFAAKIRNYRIPEPYKGKGI
LYENEKILLKSIKTKRMTFFTTN-
SHRWYLIDCKNQSLGRLSTRITQILLKKNITTYSPSIKQKNYIVLINSKQLKYKKKIYYFC
YKPGHPGKSLKEF----
SSNSIKQSIKRMLPMNRNKLIKQIKIYENDNYPHKGQINKIVMSKKVISIIKLKLQAGKA
TPAAPVGPILGQQGININAFCKEYNALTLNQIGSIIPVKLFVYEDKSYKMLIKTIPTSLLL
MNAAKISKGSSMPNEKNSGIISKNQLKEIASLKMNDLNAKDLNNAIKIIEGSAISMGISI
MLSPKTTKYNKYHYGRMKGKAKKGNKIVYGQLALQSLESSWISSRQIETIRRVIARHT
KRGVNLWFRIFPDKTVTNRASESRMGSGKGSVTHWIAVLKPGTIIVEINFIPLEIAIEAL
KLVNYKLPFKTKIIELMIYPQTILNIADNTGAKKLMCIKILKKINKSAKVGDIIVGVVKD
AIPNMLIKRSDIVRAVIVRTKKTIIRKDGLHVKFDENAAVIINNDNNPRGTRIFGPIAYEI
RNNGFSKIVSLAPEIVMNAIIEISGKQHFIEKNKFYDFNTIKNKKYLYLNKILFIYFIILGK
PYLEVIRGKIIKHLKGKKLSIYKMK-RKKMQKKQGHRQKLTRILI-------------------------
----
MNNNNNNNKNWFYNSIVFYEKIPISLIGSTKKNIYIDTWLPNNPFYKKAQKLANNEGRIE
QFIKKYKFMAHKKGAGSTKNGRDSNKKRLGVKHFHNEKVFAGNIIVRQRGFSLKAGY
NIGYGKDFTLFALIDGTVQFKVSVIMKVRSSVKKICDKCRVIKRQGKIRVICTISKHKQR
QKMAVPKKKVSKMKRNIHKSTWKKKASIKTQKALSLAKSNINKKGFLAMVKYLDYKKI
YGCFLIKNLEINQSITLGNYFRRTLLNDLKGIAITGIRISNIQHEFSIIPGVREDVLELLLN
LKGIILKGNIGRLKICGPAIITAGSILLPKNIEIINPNHYIMTLSTSSCIEIEFLIESGTGYKL
AFLQIDATFMPIQKINFKAENNKEQLYIDIWTNGSISPINAFLLALQISINNFSIIMNLEK
LELSVRTYNCLKRAGINNIGLLLNYSKLLEIFGKKSLENVNNNLYNNNNLVKKKDLRN
LIKWILINYNNTNTSLLIDTLKNLGFFYATQGAISISIEDLKVPSNKKLLLKKIESEIFETE
NFYYQGKITEAEKLQYILNLWESTNELLRNQVIQFFKKYDPLNSVYIMAFSGARGNISQ
VRQLVGMRGLMADQHGQVIDLPIVQNFQEGLTITDYLISSYGARKGLIDTALKTADS
GYLTRRLIDVSQDIIIREKNCFTSSGLTFLLGRFICKNMYLFKKDTHITLDVFDILKKIN
NIRVRSPLTCDLYRSVCQKCYGWDLSNYNLIDMGSAIGILAGQSIGEPGTQLTMRTFH
TGGVVDVTTDYDSSPLTGIFQFCNNFKGLPFRTNSGVIGRIIKKKSSAIIIKILKLNLLPN
NIILLKNNQFIKEGSPIGDLSQTIKLIRSIFSGEVEIIKEIWVLSSKILFSPKNFYKTYRINKN
SIFRSKINFYQNGLINNFFLKGGFIFNEESYNINFEKILAGNKISLLAQGIKSKTSGLLQIIF
ADITIKTGNLFFPGEIKK-----

KKISLFKLKKYIEIYETLNIISSNLTLINLCLLVQKRQYLNLYSILAYLESITISLEIIKINEKL
KYFSLISNNDCFQVKKSEINEKYIKNNLVGKILFDTKDNYIVRKGIPYLFIK-
NLIFFNHEDYIMSGNILGFLNLKKRTASDIVQGLPQISAALEGRI-------
ACFIGIPIIINPHLFLKIYFTYYNAAYRSLKKVELLILNFINNTYQSQGVNINTKHFEIIIKQ
MTDKTIITKSNDSPFYSNDVVDLYHLKYINSVLKQYYYIPVVFGISRSALNNPSFLSAAS
FQETVSILSYAALEGRIDWLRGLKENVITGNLLPIGSGMKTNVFPDMLEAQCYSFCWF
LSYGLNEKLSSFSKKYFYHSLTYTILGNEYTLKLPKYTDSDKERLDFYNIIEIKVPIELELK
ASYIKLEKLIINVPMMTTYATFLVNSLERVIVSQISRSPGIIFVYSAVIIPEFGSWNLLKINS
IILESKGYPKISLFNLLRKGGLTEIYNNLIYADWFYLGNGLKVFDPSHIFLGLTGRLKIN
NLLDLPINEKITSVTYNDFFAIIDKFIEFILKEQDFDHLKNKRVKLPGRILQDVVHEGFC
HLIKGLSKRNIKHIIQTIGLFFNSNPLSQFLDQTNPLSLLTHTRRLSYYGSGGLKKGAVS
FNIRDIHPSQYGRICTVETAEGSNVGVVNSLTVYAKINKLGLLETPFWRVINGKVIKNA
NPIYLTADIEDSYKIATADNLINYLINKLITVRYKQRFISVLPAEVEFLSISTVQTVSIGAS
LVPFFEHNDANRVLMGSSMQRQSIPLLYSQKPIVGTGLETKVVDNSGMILKSQTFGT
VDFVSSDKIIISYNLEKHAFSNQKTYINQTPIVWKGEKVKPGQSLTNGPSILDGELSLG
RNLLVAYMPWHGYNFEDAILINERLIYDNVFTSIHVERYKIETNSGKKKKETRNIPRIIK
NLDKDGLVKPGTFVKSGDILVGKIIIAPKKEEKFASRLIRAIFTIDKSLKLPVAHVATTT
NVWVAQIRKIQVGDKLAGRHGNKGVISKILARQDMPFLPDGTPIDILLNPLGVPSRM
NVGQLYECLLGLAGINLNKRFKVLPFDEKYGSETSRILINRKLRQASQNKSWLFNPYM
PGKMILVDGQTGIEFENPITVGNAYLLKLIHMVEDKMHSRYIGPYSVLTQQPLKGKAL
KGGQRFGEMEVWALEGFGAVHSLREILTIKSDDLSKRETSVGNITKFGVPESFKVFLQ
ELRAVGLDIKAYRLNEFSENEIQIIMYDYLKITLASPTQILKWSHKFLS-
NGSIGEVLTTDTIDFKTFKIQNGGLFCQRIFGPVKSYKCACGKYKICEQCGVEIINSQV
RRYRMGYIKLRFPIFHFWYLKSKPNILGLLLIKNIKGSSLIYKELKALNINTEIFKTRCFIL
VCSKIKEEAIQRIRILENFKNTGSHPSWLALTILPVLAPDLRPILFLDNGVIIGSDLNEVY
KLIIMRNNRVDDMIQLGMPGVFLKNERRAVQEAVDTLIDNKKRSIPYYGMHDSPLTSI
SDSLRGKTGRLRQNLLGKRVNFSGRSVIVVEPKLKLNQCGLPYEMALELFKPFIIFNKK
NKTLFQINSEILPILKILANHPIFLNRAPTLHKLGIQSFEPILVEGRAIKLHPSVCSAYNA
DFDGDQMGIHIPLTLEAQAESYNTMFAPYNLLSSATGEPTMTPTQDMILGCSYLTIS
NIKNLNGSNHYFSELEDVLKAYSQNRLELHSNIWVRYIIVQYLKTTTGKVILNYTIYKIL
MSRYIGPKLKINRRLGLPGLTTKKLKQKKNLRTDYAIRLKEKQKLKFNYGLTEKQLSS
YMKKARKEKYFTSTILMQLLEMRLDNICFILGFAKTIQQARQLITHKHIYVNNKIVNIP
SFHCQLNDIISISKKQTSKNFLFKNYYPSHIIFDNSLIEAKIIDYCRKEDILLNINELLVIEY
YSRMGQKVHPLGFRLSLNNNYKSLWYPDYRKYIELLKEDNLIRTFFNNLISNIRIKRKNI
IETEIHTGDPYFLLKTIKKKLTKLKVNIIEVKNFFLETEIIANLIAEQLEKRIYFKRIIKNLL
QTIKIGIKILLSGRLNGAEIAGNEWVIKGSIPLQTLKVNINYSTAEANTIYGIIGIKVWMF

KMLIDKISYIITKIKNASKAKHETVIFPFTNLFLSLLRILKEEKFIKDFKVSK-KIIVLL-
KYNKFSNRSIFKNIKQISKPSLHIYKSFNNISSL---
TGITLLSTSKGILTDKKAKKLHIGGEPLCYIIMLKERIIRITRVSKVTKGGKKMRFQAVIA
LGNKKGKVGIGMAKSKDTRDAIEKAKANGYRNLINFPITKSLSIHSPIIGKYKSSKILIKP
AIIGSGIIAGGTARAIFETAGIKNIVAKQLGSNNKLNNAYALFNALKELMSRKKKLKQQ
YIKPDSIYNNFLISLLISKILKSGKKSLANHIVYKMCSILKNQTEENPIDILKKAIIIVSPKIE
LKDKKIGKRIRKTPVEITSYRATYLALKWIIKFSKKRNEKTMGQRLANEILDILDNNSET
IRERKRVHRIAKSNKSFVFFKRMVRVRIESYNHRSLNFAQKYIINTLKISSILFINLISLPM
TKKIYCVLRSPHVDKDSREHFEIRTHKRILDIYYNSNIVNFFDNLSPEIFFRVMPTIQQLI
RKNRKKIKNKTKSPALLKCPQRRGICARVFVTNPKKPNSAIRKVARIRLSSGFEVTAYIP
GIGHNIQEHSIVLIRGGRVKDLPGVKYHIIRGTLDVGSVPNRRQSRSKYGTRKPRMRN
HTTKFPKVIIYIHSTFNNTLITVIQYFGKTISWSSAGTSGFKNSKKNIPFAAQCAINNLKY
IIKKIAFNRVKVIIKGQGLGRETSIRSIRNNGFIITSLQDKTPVAYNGCRAPKHRRIMIRIL
GIDLPKDRKILYALTLIYGIGISRAKEIIKKTKINFLKVDELEIEQINILRDVLLYNLLENN
LKRFISLNIRNLIEINCYRGKRHIRGLPVRGQRTKTNASKKKNNKDFKK------------------
----
MAKKNMIEREKKRIYLEKKYSLKRKNLLIKYKSTKDIQEKFKLELKIQKLPRNSSKVRLR
NRCWKTGRPRGFLRDFGLSRHIFREMAHQGLLPGVIKSSWMNRSLKKGPFVAYHLLK
KIKRINKKIIKTWSRSSTIIPSMVGFTIAIYNGKRHIPIFISDQMVGYKLGEFVFTRFFRSH
KSDKKK--
MTQIFDKDGSVIPVSLLKVGPCVVIGINLYN----
KSIQIGYKPDKKLTKPVLGKFKKINIKPLKYIKEFQVKDNIFKIGQILNLNIFESEKNITIK
GKTIGKGFTGLQKRHNFVRGPKTHGSKNYRKPGSIGMGTTPGRVFPYKKMSGQLGN
KVITIKNLKIVKLDIKENILIVKGSIPGKNNNLLVITMQKKKKIRVLITLECNECSSNIKRK
KGISRYITEKSKRNTPERLNLKKYCKFCNRITLHKEIKMTQIFDKDGSVIPVSLLKVGPC
VVIGINLYN----
KSIQIGYKPDKKLTKPVLGKFKKINIKPLKYIKEFQVKDNIFKIGQILNLNIFESEKNITIK
GKTIGKGFTGLQKRHNFVRGPKTHGSKNYRKPGSIGMGTTPGRVFPYKKMSGQLGN
KVITIKNLKIVKLDIKENILIVKGSIPGKNNNLLVITMLTNIYYPTKITGKLLTIQILYNNN
YFIFNDIILHYYNNRQKTVLTKTRSQVRGGGIKPWKQKGTGRARAGSIRSPLWRGGG
VIFGPKNIKQKKKINKKERKLAIQILLYNKKKKSLIIQGLKLPKTKIFLKICIGTTQKILLI
VSRKTLFLKWATKNLKNIELMLVSNLNTLSLLKAHQLILTPKAIQKIQFFYMLKLRLKK
VGKRYFPSYKLVVINSKQKQNGKQINIVGYYNPFLKTYKFNVNNIYKYFYNGIKPNKKV
FYLLKKYTMKIRYPYVTSKVRQHIYGNQYCFIVDSYLKKESIKTCLEKFFNVTITKINIIN
RPPKKKRKKNHKKVIIQLKKNQNIKYFYMNFEKKEIPFLKTGDVIKIFLEISEGNKKRIQ
QYTGTIIAKKNTSINTTINVRKVINQIAVEKIFLIHSPIILRIEILESIPVKRSKLYFLRNFKK

----MKDYK
>Ochromonas_sp._CCMP1393_KJ877675
MLKTEYPKLEKILISSSLGLNAQNRTFLQNAIEEYRLITGQQPILTTAKKSIAGFKIREG
MPMGLYVTLRREKMYAFLEKLNKIVLPRIRDFRGLNPDQFDKHGNYNMGLAEQLVF
PEINYDSVEQRRGFTITIVTTAKNKAEGIFLLKELGVPFMVRVKRGNVANKRRKKSLKL
AKGYVGKHSRLSTFACEQVVQSLNFAYVGRRLKKRNFRRIWIYRINAASRVNIYSKFIGS
LRDLNVLLDRKILAYLAFSDLSAFNAIEMAIKIYKPYTATTRNRSVLDFSSLTKKKPEKS
LTVSNHRAKGRNNRGRITTRHKGGGHKRLYRLIDFKRNKYDIEGKVVAIEYDPNRNA
NIALIHYVDGEKRYILHPENLKIGDSILAGKSITPAVGNALPLDLIPLGSDVHNIELFPG
KGGQLMRSAGTSARVLAKENNFVVLRLSSKEIRLFKKECFATIGKVSNSDFYNVVLGK
AGRSRWLGIRPAVRGSVMNPIDHPHGGGEGRCPIGHARPLTPWGKPALGLKTRKGK
KSYIIRR---

MTIFPKKEVPKWFVIDANEKTLGRLATEASKLLRGKETTFFTPGVDQGNFVVILNADK
IKVKELQKLYYRNSQRPGESFQKLKDRIPSRILERAIWGMLPKGGREYYRRLYIYSNSEI
KYKKTKDGNSMAKKIKAFVKLALAAGKATPAPPVGPALGQHGVNIAGFCKEYNAQT
QDKAGLIIPVKITIYEDRSYSFILKSPPASVLLAKFANVSKGAAEPNREVVGTVTLDQVK
EIAQTKMNDLNTNNMEKAILIIQGTAKSMGIKVMLSPKRTKYRKMHRGRLRGKACRN
NKLNHGEYGIQALEPVWLTSRQIEAVRRTISRYTKRAGKIWIKVFPDKSVTARAEESRM
GSGKGAVEYWVVVIKPGNVLFELAGVSKEIAVQALKTASYKLPIKSKILIKMIQPQTYLS
VADNTGAKKLMCIRVLGNNRKYAKVGDIIIGVVKDAIPNMAIKRSNIVRAVIVRTRKTI
KRADGMALRFDDNAAVIINTENNPRGTRVFGPVAREIRDKKFTKIISLASEVIMYAIAQ
TSGKQFLLKPGQWYDVDYINAGDFIYLNKILFFRKIQIGKPFLLSIPAKIIQQVKGPKIT
VLKTKPKKKYTRKRGHRQSYTRVQIMVSATSKYIRISPTKVNIILGQIRGKSYKEALQIL
KYLPQKAGAIVWQTLYSAVSNATNNFDFEKEKLFISEAYVNKGPILKRMQPRAKGRAF
KIQKKISHITICVMAKKGLHPEWY-
NTKVYCDGKLVLEVGSTQEELYVDIWSGNHPFYTGSQKIIDTEGRVDKFQRKYNLMA
HKMGAGSTKNTRDSQSKRLGVKCNGTQKVKNGNILVRQRGTKFKPGKLVGCGRDH
TLYALSDGIVEFTVNIIMKVRPSVKRMCEKCRIIKRHGKILVICLNPKHKQTQG---------
----
LTCVESYIQYGCFLIEPLEIGQGITLGNALRRTLLSDLSGFAITGVRVNNLKHEFAIIEGL
REDILEVLLNLKELVFKGSKGFLNVKGPIIVTAGMFRLPKGIKIINPNQYICTIVDDSELY
LEIDIENGKGYRLTTLLIDAIFMPIKNVNYKIKLIKESLHLEIVSNGSISPKRSLQEGLKIL
MNLFYPL---
ITRTVTKKILETIVHETFSNFGSLSSSSLLDSLKFLGFYYATNAGISINIEDLKTPNVKKEF
LKTATDEMSFVSQQWEQGFVSDTERFQTIIDSWNIATESLKNRIIDYYQNFDPANNLY

IMAFSGARGNMSQVRQLVGMRGLMSDQEGKIIDLPIQTNFREGLSSIDYIISSYGARKG
IVDTALKTADSGYLTRRLIYIAQDLIIREINCKTKNGIIVILGRHLLDAKEHKFNNLLLD
EKVLNQLKKPLSLNIRSPLTCESNGSICQSCYGWDLAQNNLISLGEAVGIIAAQSIGEPG
TQLTMRTFHTGGIFTELLQQTVAPFSGRIELPLSLKTVAYRTNHGIIVSKLQQEANIIIT
IREEIFLDIGSFLYIQKSGFIKKGELISEYSARSFIIYTSIAGEIHFQDILWLASGKVFPIPEKI
VDSSILLKRSFASLKIVTPYDGMINKLFL---

LTIDISSLQKSKFSVITKNYQYVDKYTTIGMLYILPYEGRIYGIRKKITTYFLITENDVWK
IHSDQK--
NIFSFSKSGFLLKKDGFKMIFQNAVPIFLSRGTILNYKQGDFVLEKQLLATLVNYTQQT
EDIVQGLPKIEELIEARIPKFLEVFKIRIGEPITIDPHELLGILFQYHKGTFRSLNKFQLLL
VNSVQSIYQSQGVNISSKHIEIIVRQMTSKVVIKESGDTPFLPGEIIRLSLITEIYKALYSN
KFEPLFLSTTNSSLSKDGFLSAAGFQETKRVLTRAAIEGASDWLRGLKECVIIGRLIPAG
SAMFLNNIGDLGEAQRASYYRFLSKGISEELINFPNPFISIKVLVYLYTNDIKLKGPTFSI
DLSLKRDTSYSIQLYVPGEYSKSNIRIKQDIFFGEIPLMTEEGTFIINGCERVIISQIIRSPG
VYFIYTATVISNKGLWTKFVLDEIYIKLNDFDKLFIYDLIRYFGLNEVYDSLKYPLHLDN
AFLQLFFNSGCFSIGEIGRYKINKKLSLNLPKHITYLTAQDFIGIIDGLIELKYYDDDIDH
IKNKQIRSIGELLQNQIRIGFYRLQKSLLEGGTVPPVSAIKEFFKTSQLSQFMDQVNPLS
ELTHKRRISVFGPNGLKRDHISTVIRDIHPSQYGRLCPIETPEGQNAGLITSISMFGRISS
LGWIETPYFLMKNAAIFSNKQPIFLNPEQESETKIAFADVSVKMLTAEYLSVKENYSFSV
KKKTDVNFITTSPLQIISLATALIPFIEHDDANRALMGSNMQRQAVPLIYPQKPIVGTG
LEATAILDSSMVIKTYSEGIVLNSCAHSILIKYYLRKYYRSNQETSINQRPIVWSGEKVFS
GQIIADGPSTNDGELSLGRNLTIAYMPWEGYNYEDAIVINERVIFDDCLTSIHIEEHET
NLSYSVSGSETKNLPHLRRHLDREGIVKIGSYVKEHDILVGKLTPC-
EEDTSPEAKLLKALYFRDTSLRVPVIDVRIISTIYIAQVRKIQVGDKLAGRHGNKGIISRI
LPRQDMPYLPDGTPIDIIFNPLGVPSRMNVGQILECLLGLAGQKLGNRFKVSPFDEIY
GKEASRVFVNQKLKEAATDASWLFNKYSPGKILLRDGRTGEYFDNPITVGKSYILKLIH
LVEDKIHARATGPYSMITEQPLAGKSQKGGQRFGEMEVWALEAYGCSNTLQELLTIK
SDDIDGRNDMYEAIPTPSIPESFLALMRELHALGLDFSMNKFENYTESEKDIFMFDYLR
IRIASPKRIKSWSERTLP-
NGIVGEVLRPETINFRTHQPELYGLFCEKIFGPIKNWKCRCGKYNICEECHVEVIEARV
RRYRMGYIELTCPVTHLWYLKGVPNYLSILLKNSESGAEIIKAALENLNLPLQIKKTRSF
IDSIFLPDKSIIRRIRILESFLATKTNPSWMVLTTLPVLPPNLRPLVELESGRLVAADVNE
IYRLIITRNQRLSDFLRFVAPDLISIQGRKLLQEGVDSLIDNARLPKKTFCLNNKALKSL
TEILEGKQGRFRQSLLGKRVDYSGRSVIIVGPNLRLNECGLPYEMATELFQPFLILLKT
KAHIIKKNPFIWTLLGLTRKHCILLNRAPTLHRFGIQAFNPVIILGQAIHLHPLVCTGFN

ADFDGDQMAVHLPLYESSQLEARTMMRPSYNVLSPSNGEVILKPTQDMVIGCYYLTL
MIRNNRYKLQKWFGNETEALVAFYQKKLSIHTPILVRYYLITLLETTPGRLIFSINFKNL
IMVRYTGPKIRIIRRLGLPGLTRKSTKNRRSSLDDYKDRLLEKQKLRYNYGVTEKQLVA
YYKQAKKTKGATGTLLLELLEARLDCVVHRLGFAPTIPAARQLVNHGHILVNNRLVSI
SSFICQKGDIISIREKEKSKALIAGNFLPSHLEIDIDTLNGKFLTPVKRKDVLLRINELKV
VEYYSRMGQKTHPKGFRLVTTQKHLSDWYSNKLKYPSLIEEDFLIREKVDTTISKIEIN
RVEYVNITIHALYPILNLLNIRNLIKNYYVSIKFIKNPFEDATLIAKYIAEQLEKRIPFRRAV
KQTIRKVLKGLKVQVSGRLNGIDIARSEWKREGSVPLHTLKAKIDYTQQCADTLYGVI
GIKVWLFVMTNDLVSDMLTRIRNASLARHTFTYIQYSKLNIEILKVLKREGYIQNYQIE
SKKLIKAFLKYKGWIKKPFFSIIKRISKPGQRIFSGYKEFDKKIDVLGTAIISTSSGIMSHV
KATKLKKGGEILCYIGMYEEKIVQIKRVTKVVKGGKKMTFRAIVIIGDNKRKVGVGIGR
ADDVNLAIDKAILNGKKNLVTVPLTLEDSVPHVIKSSYGACTIMLRPASLGSGVIAGGS
VKTVLELAGIKNISAKQFGSNNILNNAKATVLALTALMSRRISTQKRFPEKDFKYDNLL
VSLLVNRILKSGKKRLARRIVYKAFELIEFRTNQNPILILEKAIRNISPRVQLKAKRVGGA
TYQVPTLLNKYRATNIAVRWVVEFSRKRSGKGMSLKLANELLEASKGMGNSIKRKDE
THKMAEANKAFAQFRRMFRILLRSFDNELINVASQQLRSVLLSTDCQVAGAVSLPTRI
KRFCVLRSPHVDKDSREHFEVRISKRFIDIVTDSSILDLLLELPSGVSCSLMPTIQQLVRL
SRKKIITPTKSPALKACPQRRGICTRVYTTTPKKPNSAIRKVARVKLTSGFEVTAYIPGE
GHTLQEHSIVLVRGGRAKDLPGVRYRVIRGALDSTGVKDRMQGRSKYGARRPVMKK
QKKLVTVGIANIKTTFNNTIVTICDILGNTICWSSSGTSGFKGTRKNTPFAAQTAARN
AALKAMEFGMEKVEVVINGRGNGRETSIRALKSAGLSIISIEDKTSIAHNGCRPPKKRRL
MVRLLGNNLSNKKKIYIALTCIYGIGIPKSLDILSRLNIENIKVADLTEENVSALRDILEEF
KLEGDLKRLISLNIKRLIDINSVRGRRHLKGLPVRGQRSRTNNRTSRRHSMFRNMVKK
ERLGIVVSDKPNKTIVVAIQTRYQHNKYTKTLIKTKRYMAHDEENTSKAGDLVLIEES
APFSKQKKWALKMAKKGMLEREYKRKKLVTKYSEKRKNLLNQLKQANSLEEKFIINEK
IQKLPRNSAPTRIRNRCWKTGRPHGYFRFFGLCRNAVRELANDGLLPGVTKASWMGR
SISKGPYIAYHLLNKIKKMSKETIKTWSRSSTIIPLMIGHTISVYNGKKHVPVFITDPLVG
HKLGEFVPTRTFKSHKTDRKVKRMKQRNRKIVTQNKRNRIINRRYSTAMKTLNKLFQ
QKIKSYILIIVKKFYSVVDKAVKKNVIHKNNAARRKSNVGKISSKMTQIFDKKGNIIPVTI
IKSGPCYVTQIKSEDNCGYNAIQVGYMEDKKLTKPNLGHFNKANLPPFRYLKEYKIMN
ENHNVGEVFSVDMFSIGENVNITGLTIGKGNTSNIKRNNFGRGPMGHGSKHHRLQG
SLGAGSTPGRVFPGKKMPGRMGTEQRTVKNLEIIDIDTTENLLIVKGCIPGKSGNLVS
VNMAKSKGPRIVITLECTECRDNHKRSEGVSRYLSSKNRRTTPDKLEMSKHCRYCNR
HTIHREIKMTQIFDKKGNIIPVTIIKSGPCYVTQIKSEDNCGYNAIQVGYMEDKKLTKP
NLGHFNKANLPPFRYLKEYKIMNENHNVGEVFSVDMFSIGENVNITGLTIGKGNTSNI
KRNNFGRGPMGHGSKHHRLQGSLGAGSTPGRVFPGKKMPGRMGTEQRTVKNLEII

DIDTTENLLIVKGCIPGKSGNLVSVNMQKFITYNSVINGKTLELNVLKSGNYLIHKDIL
RHQSSQRQGTISTKTRSEVRGGGRKPWRQKGTGRARAGSSRSPLWKGGGVIFGPKPR
KIILKLNKKERKLALQTLLYNKRNNISIIDNLLDPKTKTFYSLCINLDQKILIIVGEKTIPL
KLATRNIKNVELILASNLNTFSLLKAKQILMTPLAVKDIKEIYMLKIRLKRTGRKSKPFY
RIVLMENLSRRDGKSIAEIGYYDPLTKIINFDNIKLHKYLNHGAYPTNTVRHLIYKMLM
LIKYPSLTEKAINLYGDRQYTFIVDRSLRKTEIKYVIEKIFNVTILEVNTCILPTKTKRKPR
YKKTYVKLKEGDSIDLFNMRIENEFDVIPKPGDILRIGYKIPEGDKERIQFYEGLVISIKN
RTLSKTFKVRRTVQGIGVEQTFIFNSPKIVSITRKQASKVRRAKLYFIRELKGKASRLKIK
R
>Phaeocystis_antarctica_JN117275
MLKSFYPKIVTVSINRGFGEAAKNSKELDVSIKELAVITGQQPTVNNARKSVAGFKIRD
GMPVGLSVTLRNERMYDFLARLIHIVLPRIRDFRGISPNGFDGRGNYSLGLKDQLIFPE
ISYDDVAQLRGFDITIVTTAKTDEEALALLKGLGMPLMVRVKRGNVARNRRKKILKFA
KGFKGAHSRLFRTANQQVMKALIYSYVGRKRRKRDFKRLWLCRVNAAARILTYSKLK
NRLKQNSIDLNLKMLAQIALLDKGTFSLIVMAIRLYRAYSPGTRSRSSLFFDEITTNRPQ
KSLTVGKKACSGRNNRGVITLGDRGGGHKRKYRIIHFNRKEPNITARVISIEYDPNRNV
RIALLCYENGTKRYILCPRSLKVGMTVSSGSNAPIEIGSAMPLSSMPLGSTVHNVELTL
GKGGQLARSAGAYAQLIAKEGNFVTLKLPSGEIRLVHKQCYATLGQVGNIEFANVRL
GKAGRNRWLGRRPHVRGVVKNPIDHPHGGGEGRSPIGRPRPVNPWGKPALGTKTR
KATKSYILRSMSRIGKKYITMPQKVKVTLERQKISVDGPKGSLFRILPSVITLDNRLLLT
RLSQALYGLSRTLVANMVTGVSDGFSKKLQISGVGYRAQLDGKDLVLNMGYSHPVR
MIPPPRISITLEGPTTVIVSGIELDVVGEFAAKIRSVRPPEPYKGKGIAYEGEVIRRKAGK
TGK---

MLLPKRTKFRRLHRGRLKGIATKSNTVVFGDFGIQALEPIWLTSRQIEATRRSITRYVR
RSGKIWIRVFPDKPITERAAESRMGAGKGAPAYWVAVIKPGHILFEVNGLEKELALKVL
KTAAYKLPIKTKIVTRMIQPQTCLKVADNSGAKKLMCIRVLGSNRRYGFVGDVIIGVV
KDATPNLTVKRSDVVRAVIVRTKHSITRKDGTRLRFDDNASVIISKENNPRGTRVFGSI
ARELKEKGFTKIVSLAPEVLMYAIVKASGRQFWIEENRFYDLNKLPLGDTFTLNQILLV
KKLELGKPFLEKVEATVLRHLSGSKTRVYKMRPKKKTRKTFGFRAKLTRIYI----------
MSPSKVRRVLRQIQGKSYKDALILLEFMPYASCEPIIKVLRSAAANARNNMNLDETTLV
VKSAFADQGPVMKRFRPRAQGRAFRILKYTSHITIVMMPKNSIHPEYYSNAKVYCDGQ
LVMKVGSTKPVLKVDIWSGTHPFYTGSQKLIDTEGRVERFMRKYGIMAHKKGAGSTK
NGRDSNSQRLGIKVYGNQPVKAGGIIVRQRGLSVRPGRDIGIGKDYTLFALRSGIVEFT
VNVQMKVVSSIKNRSKDCQVVKRRGRIYLICSDPRLKVRQG----------------------------

----
MIQCLNSKTNLGKFCIEPLKKGQGVTIGNALRRVLLSDLPGLSIVGVHINNVSHEFSTI
PGLKEDIIEVLLNLKQIIFKGDNARLVFQGPGVITAQSLELPDEITLVEPCQYIASLTGR
TSLEMEVLIEPGYGYLTSFLAVDAVFMPVRKVNFFVETSTESLILEIATDGSIEPIEAISN
AALILENVFASLVMLADLLLSVRAYNALKRANVHNLSELLKYSKEDLLFGQKSADEVC
ESLQKTNGIINKRQLKNLMYCTFHNYGVVKSSIIADRVKNLTFHYATKSGISLSIEDLRV
PQKKRSLIGLTNNEVEATEQNYEVGNITNIERFQKVIDIWNNASNFLKEEVVTYFRESD
PFNSLYIMAFSGARGNISQVRQLVGMRGLMADPQGQIIDLPIKSNFREGLTVTEYIISSY
GARKGLVDTALRTADSGYLTRRLVDVAQDIIVREEDCYTQDGLLKMVGRLLAQPLT
VLPINTQLTSNLLDDLELIEQLKVRSPLTCNSIRSICRNCYGWHLAYSKLVDLGEAIGII
AAQSIGEPGTQLTMRTFHTGGVFSDLTQQIRAPFQGTLSYKTAEAANLVRTLHGTRS
FLIKENVTLYIKNCSKIKLPEGATLLSNTGQKVYTNQIIAEIKKDSKTVYTNISGEVFFQ
NLIWVLHGKRYSLPRSSELVAKLSGSIIAKKQILNNYSGIVKFFQITGGTVIPEETYRVES
SKLQTKDSTKEIIPKTFTKSSGILQVAGKEISIKPGEFIEPGKQIINNIIAQKLIYIELIRPVQ
KYKVVELLNTFLTLIATRFLVNKNQYVTSSSTLAQLEIFFPIKGTLGNTANVNEILILQD
KDIRPVPHFSKPSTSTKSEYSGQVYSVDKTNVYIRLGRPYLISQGAILEVESGSLVQRLD
KLATLIYEKLKTVDIVQGLPKVEEILEARKIKNPCILYVKLIEPLTISPHEKLETLFNYYEA
CRQSFRELQLFLVNEVQRTYLSQGVQIADKHIEIIVKQMTSKVRIEDSGDTILLPGELL
NLYQVEIITKSSLAVFYTPLLLGITKASLNSDSFISAASFQETTRVLTEAAIEGKKDWLH
GLKENVIIGRLIPAGTGMYKYLLPNLLEIQRISFCWFLEKGLVQEFEDFSLIRDLGELEL
NLSTKFYKIKRPKYTLEEAKRRDATYSVPIYIVAHLKRVE-
TSKAEVFLGDIPLMTNEGTFLVNGIERVIINQIVRSPGIYFNYIASLISNRGTWIKFEVGR
VQVKIDKARDLSAYSFLRALGLDTIFTNLDHPDYFQNADLSKFFDPKRYDLGYVGRYK
LNQTLELDIDEKIRILTSRDILQIINTLINFRISEDDIDHLGNRRIRSVGELLQNQIRIGLN
RLERTIRERMAIKLIASIREFFGSSPLSQFMDQTNPLAELTHKRRISVLGPGGITRDRAG
FAIRDIHPSQYGRICPVETPEGPNAGLIGSLSTYSRVNSYGFIETPFYKVASGKVLKELFP
IYLDATNEDKFRLAAGDLLTNYIHDSKVPVRHNQELINVSPQDIDYVAVSPIQVVSLAA
ALIPFLEHDDANRALMGSNMQRQSVPLLYSEAPLVGTGLEGQTARDSGMTILSLNCG
KVVNIVGDGIVVKYLLTKYRRSNQETCINQKPLVWVHEKIFMGQVIADGASTEGGEL
AVGQNILIAYTPWEGYNYEDAFIINERLIYDDLYTSLHIEKCEIDVRQTKLGLETKDLL
NAIRNLDENGIVSLGAWVESGDILVGKLTPKGESDYPPESKLLRAIFVRNTSLKVHVIDI
KIFSKVYLAQTRKIQIGDKMAGRHGNKGIISKILPRQDMPYLPDGTPIDMILNPLGVPS
RMNVGQIYECLLGLAAENFNTRFKVIPFDEMHGVEASRALISKKLIESAESKKWLFSKN
YPGKMVLTDGRTGQTFENPITVGKSYILKLVHLVDDKIHARSTGPYSLVTQQPLGGR
AQQGGQRLGEMEVWALEAFGAAYTLQELLTIKSDDMQGRNETLNAIPRPGTPESFK
VLMRELQALCLDIAAYTVSDQGDTEVNLM-FDYVKINLASPNRIQSWGQRILP-

NGCVGEVTKPETINYRTLKPEMNGLFCERIFGPVNDWECHCGKYKVCERCGVEVIES
KVRRHRMGFIKLASPVTHVWYVKARPSKIALLLGLSLKGAEAIKKLLEQLDLEQESKNI
RASLPFVRPERDKLIKKLRIIDQFIASGCNPSWMVLDIIPVLPPDLRPMVQLDGGRFAT
SDLNDLYRRVINRNNRLARLQEIMAPELIIRNEKRMLQEAIDALIDNGRRGKAVVGLN
NRPLKSLSDIIEGKQGRFRQNLLGKRVDYSGRSVIIVGPELKLNQCGLPREMAIELFQP
FVILIYDGAKIIQNNALAWEILQVMEGHPIFLNRAPTLHRLGIQAFEPILVEGRAIKLHP
LVCPAFNADFDGDQMAVHIPLCLEAQTEARLLMLAPNNFLSPATGDPILTPSQDMV
LGCYYLTANNPSQQKKNEPYFSDFKDVILAYQQAQIDLHTFVWVRFSFTKYIRTTPGR
ILLNEIFH---
MARYTGAKLRITRRLGLPGLTSKKGNSSQKKLTQYAIRLEEKQKIRFNYGLSEKQLMN
YIKQAKKIKGTTGTILLQLLEMRLDNLVFRLGLAPTIAAARQLVSHGHITVNQLPVSIP
SYQCQPGDKLSIKDNVTSKRLVNKYLIPQHLEFDKKNLSAKILGIIDREWVALKLNELF
VIEFYSRMGQKIHPTGFRIGINQPYQSTWFANYGVYSKVLQEDYKIRQFFEKEIAKLEIK
RKNKIELLIHATRPKLIALKLKSELRKVRLKVIQIAKSENESLLVARSLADQLEKRVAFRR
AMRQTAQRLKKGFKIQVSGRLNGAEIARAEWVREGRVPLQTIRADISYAHYEALTTYG
ILGIKVWIFDMTNDTIADMLTRIRNANLAKHQIVQIPSTKVTKAIAKVLLDEGLIESFEE
LDRGSLLISLKYDGVDRKPVIEKIQSISKPGLRVYSSSKKMPRVLGGFGTAIISTSRGLM
TDRQARSKCIGGEILCYIWMWQERVVAVQRVTKVVKGGKKMSFRAIVVVGNEQGKV
GVGVGKATDVINAVKKAVADGKKQVITVPLTGANSIPHATTGRFGAARLILRPSAPG
CGVIAGGATRTVLELAGVKNILSKQLGSNNLLNNARATIEGLSSLMSRRNRAKRILVQ
PDPIYGSRLVNLLIARILQSGKKSVAQKIVYTALEIITSKTEENPVLVLEKAVKNVTPQV
EVKARRVGGSTYQVPIEIRAYRGTNISLRWINESAKTRSGKSMSFKLANELIDASKESGN
AIKKREQTHRMAEANKAFAHFRRMLRIALKAYEPSLLDDSCRQIIESVRLEGTEVTGPV
PLPTKRRIYCVLRSPHVNKDAREHFEMRTHKRLIDIHSPSSVLESLLDIPSGVDVEIMPT
IQQLVRFERVTNKKTSKSPALKMCPQRRGVCTRVYTTTPKKPNSALRKVARVRLTSGF
EVTAYIPGIGHNIQEHSVVLIRGGRVKDLPGCRYHVVRGTLDAGGAKDRRNGRSKYG
GKKPKMKKSKKAPSLGVVHVKSTFNNTIINITDKQGNTLFWASAGGSGFKGAKKGTP
FAAQSAAEKAGSLAYEQGMRQTEVIVSGPGSGRETAIRALQGCGLEILLIKDITPVPHN
GCRPPKKRRVMVRISGVDLPRNKRIEIGLTYIFGIGLTSSKQIIASTNISDTVCMNLTDE
EVNVLRTIIEQYQTEGDLRRVYSLNIKRLTEIGSAQGRRHRVGLPVRGQRTRTNARTR
KGKIKTKKMVVKEKIGIVVSDKMVDTRIVAVSDRIIHKRYKKVVTRTKRYAVHDSEFNS
KAGDKVRIKQTVPISKTKTWAVAMAKQSMIQREIKRENLIAKYETKREVLKQQLNSD
DGYKVKLQIYKKFEKIPRNSSAVRHRNRCWVTGRSRGFYRDFGLSRHVVREMAHEGII
PGLKKSSWMTRSLKKGPFVASYVMEKVEAMKKDTIQTWSRTSTILPNMIGHTFAVYN
GKQHVPVFVTDQMVGHKLGEFSPTRTFRSHKKDKKSKR-------------------------------
----

MTQIFADDGTAVSVTLVQAETCQVSQIKTVDTDSYNAIQVSFAELSKITKPQLGHLKK
SGTKGFKYSGEFQIDDNSYKLGQMLDVNVFSTGQKVDITGKSIGKGFAGNQKRHNFS
RGPMTHGSKNHRLPGSIGAGSTPGRVYPGKKMAGQLGNKMITIKNSEILFISAPENIL
VLKGSLPGKKDNLLRIAMAKGKGARIQVTLEH---KCEL----
GTYRYTTVKNRRNTTERIELRKYSPVTKKHEVFKEIKMTQIFADDGTAVSVTLVQAET
CQVSQIKTVDTDSYNAIQVSFAELSKITKPQLGHLKKSGTKGFKYSGEFQIDDNSYKL
GQMLDVNVFSTGQKVDITGKSIGKGFAGNQKRHNFSRGPMTHGSKNHRLPGSIGAG
STPGRVYPGKKMAGQLGNKMITIKNSEILFISAPENILVLKGSLPGKKDNLLRIAMQKF
ITYTPLLNGKQLKLNILTSGNYLVHKDLIRHFGSQRQATSSSKTRSEVRGGGRKPWRQ
KGTGKARAGSNRSPLWKGGGVIFGPKPKKISLKLNKKERQLALQTLIYNKKNNILIIEN
LMTPKTKAFVKICINLEQKVLLVVTDKTSVLKLATQNLKNVELLAASSLNTLSILKARQ
IILTSAAINHIKESYMIKIRLKRYGRKQRPSYRIVAMDSRVKRDGRAIEELGFYNPLTDET
HLKFEPIIKRLKQGAQPTDTVHNIFVKAKMLVKYPILTEKTIQLLQSNQYSFAVDKKA
NKVSIKSAIEELFDVKVVSVNTSMRPLKRRRKPRHKRAIVTLAPENSITFFEMQYKKKD
LPDLCVGDTAIVTIYLEKKEKERTQAYEGVIIAKHRKEPNSTITVRKVSQEVGIEKVFLV
NSPWIKDIKVISRARVRRAKLYYLRERTGKSARLKRRF
>Phaeodactylum_tricornutum_EF067920.1
MIKQEYPKLKKIQINRGLGLAAQNTNLLKKNIEEFEKIAGQKPLITRSKKAIAGFKIRED
MELGLSVTLRGEKMYTFLTKLLFFTFAQIRDFRGLSLRSFDKAGNYTLGLKEQLIFPEI
DYDDVDQIQGFTINIILEHGSPKNGMILFKFLRFPLMVRVKRGNVARKRRKKILALASG
YRGAHSVLFRVANQQVMKALRYSYVGRKQKKRIFRRIWISRINAASRLISYSQLIHRFKK
SNIDLNRKMLSQIAVLDTVAFKTLLMSIRLYKSYTPGTRNRALSVFDELTKTTPEKSLI
RKNHRNKGRNNRGIITVRHRGGGHKRRYRLIDFKRNKYGVEGVIASIEYDPNRNARIA
LVNYTDGEKRYILQPKNLTVGDRILSGSGSPLNIGNTLPLNEIPLGSSIHNIELIPNRGG
QIVRAGGTSAKILAKDGDYITLRLPSKEIRLVRKECFATIGEVSNNDAFLVQSGKAGRT
RWLGKRPTVRGSVMNPCDHPHGGGEGRAPIGRTRPLTPWGKPALGMKTRKRKKSYI
LRRMSRIGKLPITIPENVDINYNDSEITVKGKFGTLQTQIPTVIKIDNILTVGRSVRALH
GLYRTLINNMVIGVSEQFEIILELKGVGYRAAVQNNEIILNLGYSHPVNIKIPNIISVEVV
QNTTINLKSCDKELLGLFAANIRAWRQPEPYKGKGILYKGEQIIRKAGKSAKLTFLPTK
NNRNWFIIDCKNQKLGRLATIIVSLLKGKTKPHYYPSIDIGDYVILLNADSIIIENSKHYI
VNKPGKPGSSLKNVSDCLPKFTIERAVKGMLSETTKRLMRRLNIYSESSHPHQAQKPIE
IMAKKITALIKLALPASKATPAPPVGPALGQHGVNIAAFCKEYNARTNDKAGLIIPVEIS
VYEDRSYTFVLKTPPASVLLANAAKIKKGSSNPNTVSVGSITKTQLEEIANIKLPDLNTT
KISSAMKIVEGTARNMGISIMLSPKRTKYRKYHRGRMRGAKTRGNEICFGNFGLQALE
PTWITSRQIEAARRTITRYTKRGAKLWIRIFPDKTVTARAAESRMGSGKGAVDYWVAV
VKPGTIIFEIGSVPEEIAKTALNLAAYKLPIKTKFIIKMIYPQTMLTVADNTGAKKVMCI

RVLGGNKKYAKIGDTIIAVVKEALPNMPVKRSDVVRAVVVRTKKSIRRQDGMYIRFDD
NAAVIVNMDNNPRGTRVFGPVAREIRDKNYSKIVSLAPEVLMYAIVEISGRQFWIETGK
YYDFNRIPTGKQITLNRVLLLNEILIGKPYLDVIKGKILEHLRGKKTIVYKMRPKKKTRK
KQGHRQELTRVLIMVTAVAKYIRMSPHKIRRVLDQIRGRSYQEALMILEFLPYDAGGPI
WQVIHSAAANAKHNSGLDKKKLIIDKVFADEGPKLKRIRPRAQGRAYKILKPTCHITV
VMMPKSDIHPTWFENTKVLCDGKPLCLIGSTKSELQIDMWLANHPFYTNSQVLIDSE
GRVEKFMKKYRLMAHKKGAGSTKNGRDSNAKRLGVKRFGGEQVTAGNILIRQRGTK
FKPGINVGCGKDFTLFALTNGIVKFDINIIMKVRPSVKKMCDKCRVIKRHGKIMVICSN
PKHKQRQGMAVPKKRTSKSKKNARKANWKRKGYKAAQKSLSLAKSMLKTTSFVYMI
KCLKSEKQHGQFLINSLNPGQGITIGNQLRRVLLGDLGGVAISAVRIAGITHEFSTIPG
VREDILEILLNLKGIICTSQFGHLKVQGPSVVTADLIQLPPGVEIINPNHYIATISTSNILE
IEFKFEYGSGYHLAYLQLDTIFMPVQKVDFKIENISERLFLDIWTNGSISPNDALESAAQ
VIIDLFTLLIAIEELQLSVRAYNCLKKAQINTVGDLLQYSPLQELFGRKSADEVFSTLKN
QNTLIGKKQLRQLLAWSFTNYDSMQACALADELKYLGFKYASQAGISISIEDLKIPFVK
NLMLEKANQEIINAEKIYLKGKITDVERFQKIIDTWSLTSESLKEQVIYYFKNYDPLNSV
YIMAFSGARGNLSQVRQLVGMRGLMSDPSGEIMNLPIKKNFREGLTITDYLMSGYGA
RKGIVDTALKTANSGYLTRRLIDVGQDVLIREKDCLTNHSFLFILGRVLSKPIHLVPAG
TQITPKLIEKFKYVKKFYIRSPLTCNLYRAICQNCYGWDLANENLVDIGEAIGILAGQSI
GEPGTQLTMRTFHTGGIFTEARQQITTPINGVIRFYKTLKTVLLRTNRGEDVLITKNS
GSVILIDLVQIEILRNTILFPKNNQYIVKDTVIGELLNTNRQILSDTCGEIFMNNLLWILS
GQLYNGPNNFYSDYKLNCSYIFRTKIINHYSGSVKNYLLNGGIILGEEVYKVNCEILLAG
DFIFELIPGLFSKTSGVVVILIHTISIKSGKVYYPGEIIFSHIPITKISFCELVRPIEIYEFLNLI
STILTLLQSCLLIQPNQFIDRYTNLGYLESKTNSLEIVKIKLKSKQILLISNQDCLTVKKE
KGKKDFINVNETGKIIIENENNLTLQKGKPYFFQDVNSVFCKNSEFIEEGETLGLLNLE
KEITGDIVQGLPRIEEILEARKKNTSQKKVQKLGTNIKVNPHKLLKVYFNYYEGSYKSF
KKVQSFILDSVQAVYQSQGVIINDKHLEVIIKQMTTKVLITYEGNTPLLRREVIDLYHI
QYINQIIQSQCYVPLLLGITKAALNNPSFISAASFQETTRVLTKAAIEGRIDWLRGLKEN
IIIGHLIPAGTGMYTNALPDFLAMQRISFCWFITQGLTEELALFSRIQDFQNTEYVMFG
EEYSLIKPSYSLLIARKYSGNYRAQLVIPIEVRNKVIRYHNQFPIITLPLMTTDATFIINGC
ERVIVSQIIRSPGVYFLYTATVIPEYGSWIRIGFQRVIIQLDKINQKPVLYLLKEMGLTEIY
QNLEYADFFYFSEFSRIFDPTYYRLGRVGRLKINSRLNLKMSERLQTITYEDIFAITDKLI
NLTISKDDIDHLKNRRVRSVGELLQNLFRIGFQRLGRKLRNQTNKFIVASIREFFGSSQ
LSQYLDQTNPLSSLTHRRRVSGLGPGGFDRDRISFAVRDIHPSHYGRICPIETPEGQNV
GLIASLTTCARVNKSGFLETPFWRVINGKVVKTGQPVYLTADIEDFYKIAPADIATNYL
TKNVIPVRYKQDFVNVTPSEVDFIAISTIQVVSVAASLIPFFEHDDANRALMGSNMQR
QSVPLLLPQKPIVGTGLENQIAIDSGMTLNSYSNGVVSSVTANKIVVNYKLQKYLRSN

QQTCINHRPIVWKGEQVKSGQILTDGPAITSSELSLGQNVLIGYMPWQGYNFEDAILI
NERLVYDDVFTSIHIERYKIEIDRNSDTLETKNIPNLIRHLNDDGIVTVGTFVRPGDILV
GKVISNNTSEQLPESKLLRAIFVKDNSYRMSVIETVTFNRVFIAQIRKIQVGDKIAGRHG
NKGIISRILSRQDMPFLPDGTPLDILLNPLGVPSRMNVGQLYECLLGLAGDKLNARFK
ILPFDEMYGLEISRILINKKLRQASKNESWLFNPYAPGKMVLIDGRTGKEFENPVTVGN
AYMLKLIHLVDDKMHARATGPYSLITQQPLRGKAQHGGQRFGEMEVWALEGFGAA
FTLKELLTIKSDDMQGRNETLNAIPKFGIPESFKVLLHELRSIGLDMSTYKINRFSEVEV
NLIMFDYIKIKLASPFRILQWANRKLP-
NGFVGEVQKSETINYRTFKPEMDGLFCERIFGPSKSLECACGKYKICERCGVELTESRV
RRHRMGYINLIYPVTHVWYINSRPNFMALLLVKRIKGAPLIKRELEKLNLKSEIFRMRYF
ILVCTKIREQAIKRIRILENLVGTGSSPAWMILTILPVIPPALRPMIQLEGGRFATSDLNE
LYRRIITRNNRLLRLLEIDAPQLIIRNEKRLLQEAVDTLIDNGKRGKIALSANNRPLKSLS
DIIKGKHGRFRQNLLGKRVDYSGRSVIVVGPSLRLNQCGLPYEMAIELFQPFIILINQG
AKLIQQNSIIDPVLKVLKSHPIFLNRAPTLHRLGIQAFEPILVQGRAIKLHPLVCSAFNA
DFDGDQMAVHIPLSLEAQAECYMLMLAPYNFLSPANGEPIIMPSQDMVLGCYYLTVN
NINGLLGSSHYFANLADVILAYSQSKLEIHSAIWIRLLIVQYVQTTTGRAILNYIIQKTLM
SRYRGPKLKISRRLGLPGLTTKKSNKLIGKKTEYGVRLEEKQKLKFNYGLTEKQLFRYV
KEARRRQGVTGLILLQLLEMRLDTLCFTLGFAKSLAQARQLVNHGHITINKKVVSIPS
FQCRLNDIISIKEKSSSKTLVETNIIPTHLRFDTVKLEAIVLDYCDRNDVSLQLDELLVIE
HYSRMGQKTHPLGFRLGITQEHKSTWYANFNQYANILKEDDKIRTYIHTIIANVRINR
NDQIQLNIETGKPILVLLNVKKFLRQLTINVLEVEKVDLDASLLADLVAEQLEKRIAFR
RAIREALQRAKNGIKIQVSGRLNGAEIARSEWIREGRVPLQTLRADIDYATQEANTIYG
VLGIKVWLFKMVNDTISDMLTRIRNANMVKHQIVQIPASKMSLAIAEILKQEGFIGDFE
TYEKKYLLISLKYVGKLRKPVICKIERVSKPGLRVYSNSKKLPRILDNLGIAIISTSKGVM
TNLKAKELGIGGEVLCYIWMLVERLIKISRVSKVTKGGKKLSFRAIVVVGDENGKVGV
GVAKADDVVNAFKKAKTDGRKNLIELPITKALSIPHNVAGNFGACKVIMRPSIEGSGVI
AGGAVRIVLEVAGVKNVIAKQLGSNNLLNNARASVCALQNLMSRRNISKKRFPETDAI
YNSYLVSLLISRILKSGKKTIAKKIVYQAFDIIKKKTNEDPLTVFEKAIRNASPIVEVKARR
VGGSTYQVPVEVTGFRATNLSLRWIIRYGDQRVGRSMAIKLANEIIDTANDIGNTIKKK
EETHKMAEANKAFAHFRRMIRVRLESFNHELLNSSCQKIVNILQNTLSNSIGVITLPTR
KRIYCVLRSPHVDKDSREHFEIRTHKRILEIYYNSPIFDLLSDLPPGVFYRIMPTIQQLVR
SRRIQIKKKTKSPALVNCPQRRGVCTRVYTTTPKKPNSAIRKVARVRLTSGFEVTAYIP
GVGHNLQEHSVVLIRGGRVKDLPGVRYHIIRGALDTGGVKGRTQGRSKYGVKKPKM
RKEKNSFTSGVVHIQSTFNNTIVTITNLTGDTISWASAGSSGFKGARKSTPFAAQTAAE
KAALEALSTGMKTVEILVKGQGSGRETAIRAIEGAGFDIISIQDITSVPHNGCRPPKRRR
VMVRLVGVDLPRNKRIAYALTYIHGIGLTSARKIIEIANINETRTVDLTTEETVALRQTL

EDLKLEGDLRRFNGLNIKRLNEINCHRGKRHRNNLPVRGQRTRTNARSRRGSKKTKK
MPVKQQIGIVISNKMQKTIVVKIENRYPHPMYSKTLIKTKKYLAHDELGECNIGDQVL
VEECRPLSKRKRWKLIMAKKSMIEREKKRIKLTKKYALKRATLLKKYQTEESFNLKLEL
HSKIQKLPRNSAKTRIRNRCWKTGRPRGVFRDFGLSRHVFREMAHQCLLPGVTKSSW
MPRSLKKSPFVAYHLLKKINQMKKDTITTWSRSSTILPSMIGFTIAVYNGKQHVPIFISD
QLVGHKLGEFVSTRNFRTHKADRKTKRMNKSAQKRIQVNERNRLQNRFYKSSVRTLI
KVFLKNLEIYLQKILSSVYSLIDKGTKKNVFHKNAAARKKAKLASSLKIMTQIFDESGNII
PVTILKVGPCVITQVKNQSKDGYDSIQIGYGNSKALTQPELGHLQKSNIQPLKYLKEF
RINQTEFQIGQVLNVDSFTPGQLVNIQGKSIGKGFSGLQKRHNFTRGPMTHGSKNHR
APGSIGMGTTPGRVLPGKKMSGQLGNKITTIKKLKIIQINSEENILVIKGSVPGKPGNL
LSIVMAKNKGTRILITLECTECRSNEKRSPGVSRYSTQKNRRNNPERLELKKYCSHCNK
TTLHKEIKMTQIFDESGNIIPVTILKVGPCVITQVKNQSKDGYDSIQIGYGNSKALTQP
ELGHLQKSNIQPLKYLKEFRINQTEFQIGQVLNVDSFTPGQLVNIQGKSIGKGFSGLQ
KRHNFTRGPMTHGSKNHRAPGSIGMGTTPGRVLPGKKMSGQLGNKITTIKKLKIIQI
NSEENILVIKGSVPGKPGNLLSIVMQQFVKYNPLINGQVLRLNTLNSGNYLIHKDILRH
YSSQRQGTVSTKTRNEVRGGGRKPWRQKGTGQARAGSNRSPLWKGGGVIFGPNPK
KVILKLNKKERKLALQTMLYNKKKSILIIENLIKPQTKRLVQICIILNQKLLIITSKTPNSL
KLSTRNLKNVELISATNLNTFSLLKAKQIILTPLAINDIKEMYMLKLRLKRTGRKRSPSY
RLVVMENTARRDGRPIEELGYYSPITKQYKFDVEKIKKWLDFGVKPTETVSSLLRKIIM
IIKYPIITDKATRLLENNQYSFVVDRYSNKITIKSAIEYLFNVKVIKINTCRLPRKQKRKP
QYKKAIVTLSEGDIINLFTMAIEKKDLPTLKIGDNVRIGVKIIEGTKERVQFYEGTIIAKK
NSSINMTITVRKILQGIGIERVFLVHSPKIDSITVLRSSKVRRAKLYYLRNLRGKASRLKQ
TF
>Pteridomonas_danica_NY
MIKKTYPILSKITLNRGLGIFAQNNLLLKMTINEFRLITGQHPFLTYSKKSIAGFKLRAD
SLIGVSVTLRKSKMYSFLDRLIHLSIPKLFDFKGFSKKNFDKHGNYNFSFKDQSLFPELE
AETIIQKLGLNISFIFSTKDITQNFIILSNLGFPFMIRVKKGNNGIKHHKKILKLAKGFFA
AHSKLFRTANTKIIKAFQYSYVGRKQKKRFFKNLWLSRINATIKIKSYTKFISKLKNNKIN
LNKKMLSQLMIINPKLITKLLMQI-
INNCITQRLNTFFFNFIKNKKKKNEKSLLIFKHVKNGRNNQGKITIRHRGGGHKKLY
RKIDLKRN-
LTLKGYVKAIHYDPNRSAKLALIIYSNGCKRYILWPQNLSFGNFILTNKIKILNIGNNLF
LKDIPVGFHIHNIEIKKNGGGQLVRAAGTSAKILTKQENYVIIRLPSKEIRLINKNCKATI
GVVGITT--
NTNYFKAGKKRWLGIRPTVRGVAMNACDHPHGGGEGKSPIGRKTPLTPWGKITLGK
KTRKTKNSFIIKRMSRIGKLCLFIPKNCKIILTPRHCHFLGSYGKLSQLIPKSLSLRHFQK

FSTLISEQQGLLHALLQNKIIGVSKQFEKKLELIGVGYRAYIDSEKLILLIGASYTTFFFIP
ISLTVNIITNTQIIIQGIDKHSICLFASKIRSLKPPEPYKGKGIKYFNESIIRKIGKSTSMTFF
ESKNLQKWYIINAKNKVIGRLASQISQILLSKNNVLYTPFLLSKNHIIIINSKEIIIKVLKKK
YFKYSGYPGNTFLDLKKQNPEKLIRHAVAGMLPKNGRHSFLNLYVYSSKTHSHVSQFP
KYLMILKILTIIKLELIAGKATPMPPIGPALGQYGLDISKFCKEYNNQTSTQSNTVIPVEI
TIFENRTFSFILKTPPTSQLLLKAAKIIKGSSEPNQKVVGSISHETLNKIVLVKLLDLNTK
NLNSAYNIILGTAKNMGITVMLKPKKIKFEKQHRGRMTGKDKHTSYPKVGKYFLQAK
ESQWISAKQIESTRRTIIHKLKREGKLFILFFPHKPVTTRVSESRMGSGKGNISHWVAVI
KSNMYLFALQGITKELAYEALKAGSYKLPIKTQIIIKMIKVQTYLKIVDNTGVKKILCIRL
LGTQQKIGIIGNCIIGSIKYSLPKSAFKKSDIVRGLIIRTRKPLRRLNGSRIYFRENAVILIN
KENNPIGTRIFGPILNEIRLKGFTKVASIASEIIMYAIIQLNGGQLWIENNIVFKFEKINEG
SFISLNKILLFQKIYIGQPFLQKILCIVMKKITLNKIQIYKMKAKKKYRKKKGYTHCFTYL
FYMTQSSLKYIPLSTKKIRFVLNEIQKKTYKETLMILELLPYKACEYIWQILNMAIANAN
FLYKNKKEHIYIEKTCLGAGPTQKRIKFKSQGRISIIKKKTCHITLFLM-
KKNLHPKWFSKTFVYYDNSLIKIVSSTKHQIYVDSWSGTHPFFNKSQISTTTRSQIDRF
LQKYKYMAHKKGAGSSKNGRDSVSKRLGIKISMGGTVKAGDILVRQHGLKFKPGLNV
GYGKDYTLYALQSGFLFYKVVIKMKVRSSIKKICKFCKILKRKKILRICCIIAKHKQRQK
MAVPKKRVSKSKKKKRLINKKKYIKSFIFNSFLLSCSILSNFS---
MIKQIKSQKLQGHFIISNLLKGQGLTIGNTFRRILLSNVFGTSIVGINIPGVTSEFSSIYGL
REDVFEFLLNLKDIILRSNYGIIKIKGPGILTASAIHFNSNIQIINPNQYLATIYHKENYIFE
IIALKNKGYIVNFLNIEAVYFSILNVNYRINFSNDSLLLEITTNGSILPNEAIIYSSKLLKNL
FNS--
KNHIFKKNDIKRSISESFLFYGKRRTVTLVNELKNLGFYYATKSGLSLGIEELKVSCIKDI
LLENANQILQYANFAFFSGFITTFERFILILNSWINATNFLTTQLVYFLQKLDPFNSLNL
MVFSGARGNLTQVSQLIGIRGLISDSNGQIMEIPILHNFSEGLTLTDYLLSTYGARKGI
VDTSLKTADAGYLTRRLIYSSKDLIIRDLDCFTKYFIKIVVGKTCAETIYIIYKNTIITKIIA
NRLRCKSFICIYSILTCNLSRSVCIKCYGWNLSSNKLITLGATIGINAAQSIGEPGTQLTM
RTFHTGGSFTIPSRQIHAKYTGFILFSQS-
KSIPIRIADGRQIQKLVQPSVLKLFAVWTFILSFGFYIYISNKSFILKNAIIAEIPVIFKTLIA
PHSGELSLFSMLYILEGIVYNIPFSTLLNSKFNVLILLYIKLCIFNNGLILKFFIKLPYFASN
LTYLYNIPIYYFNMFFQQLKHNIIFKNSVMFYILKSYLSIKITNLKFPGEILFKKFYINSITK
IEFFYPILLINIFNLLITTLWLLKFYPQFQIFEYLLTKMIIGMFLFFITELYFFNVFSFHSRV
LFLTDLHYFKQYYEISRIPFINFLYNGKIQLINSFSITIHKGTPYYITKNLQIYSHPKHFLLI
NEIFGFISFDQILTGDIIDGLSKIDNFFELRIPQNSTKLYISLGQSLTINFFEFLTLLFYYFL
STYFSFKRIQILLIKKIYYIYITQHVNLSSKHIAVIIRQLTTNIKIIWDYTNSFLVGEIITLIHI
LSINSIYSLFYYKPYLIGITKVSLNSKSFLSDSSFQEPTHILANAAIEGRIDWLYGLQENIII

GRCTPIGNFAFITTLPSLIEIQCSSFCWFLTQGLDYSFQFLQPNIFLFGINLMFWKKNYF
FIHSKTNLREIKRYNSTFLIKIYIPITFFCSNSLTDELICIGEIPLITNNGTFIINGYEKIIIQQ
LVRCPGIYFIYNLTIIPQTGTWLHLEMNSLTICIDKSQKFNICNFFELFSFSDTFKSLKQ
KASFRTIFKNHIFTQFFYNLGPIGRYKLTTEYALALFENQEIFTLKNLFQIIQYLITIRANI
DDFDSLKFKNVSTVGEILNFQFLASIKRLQRQFTSVLLDTLISSFRTFFVSSRLVQFLDQ
TNILADLSHRRRL--
LVSSTSSAVNVSTKMRDIHSSFFGRLCPIETAEGNNIGLSAALSVYARTNKYGFIETPFF
LLKDGFILFQFPAIYLTTYEEELYKVINFDVCVNKILKKFLNIRYKNNSFICLSTEIQLISIS
SLQNFSIGTALIPFLEHNDSTRALMGSNMQRQAIPLLFSSKPIIGTGLELQVATSLG--
LKSFLAGHVFEVTHNCILISYFLQKFIKSNQNTCISYSPLVWKGEFVFGGQIIAETFGTK
NGELSLGQNLLLAYMSWNGYNFEDSILINECLISLDVLTSLTISTFTINIQVIPNRSETA
DLPFQLKTLSSTGIIKKGTFIKPFDLLVGKVTRFTQ-
DELFFYLLFSNISINNTSIYATVLDVITRSRIFFAQIRKIQVGDKLAGRHGNKGIISKILSSE
DMPYLPNGKSIDLILNPLGVPSRMNIGQLFEGLLGFACSNFSTGVKILPFDEIFGDNSS
RLLIHNYLYKAKTKLFWLYNKIIPGKIILRDGKTGNTFDNPILICKSYILKLIHLSAEKLTI
RSTGPYSLITQQPVKGKSIIGGQRFGEMEVWALEAFGATYTLQEMLTLKSDDIIGRTH
ILESIMNNSLTESFKHLILELKALGLKISFYNNTTI-------
FMFDFLKITLASPKSLKFWGTRIRKNNSIVGQITKTEIILPFYNKPYPKGLFCEKIFGPVQ
NWECLCGKYKICEICDVEICISDRRRFQMGFISLSTDVAHIWYLHGIPSFLSLILNISKKG
ASAIKYLLERINLQTELLRCRLLLHFYKDSKKTLIKRIRLLENCIATNLQPQWMILTIIPV
LPPFLRPLLIEENGGIIISTLNELYLNVLKQNNRLINLLKYQASSLIINNERRLLQESIDILI
DNGKRINNVNNLHKTALTSLSSFLKGKFGHFRENLLGKRVDYSGRAVIIVNPTLELYQ
CGLPYQLTLELFKPFLSLINDSTTFFKTENAFLYFLKLCKNRLVLLNRAPTLHKLGIQA
FEPILVVGNAIMLHPLICSAFNADFDGDQMAVHLPLTCNSQREAKSLLFSPNNILSVT
TGEPAIIPSQDMIIGYSYLSSSICYLKYPKNFYFRTLTDIRRAFYQHYITLHTPIWVCSKF
KRFVQTTFGRLVLNNLFHNTLMVRYYGPKYKIVKSLGLPALTKRIVKHKTKKRSEYKK
RLLEKQKIRFNYGLSEVQLSKIVNNLYNVRTFKSQNLLTKLEMRLDNIIFSFGFTRTIAH
ARQLINHGKVYLNFKRLTIASYFCKISDIIFLNLTDSQKKQIQKFFLPLNLRYLAKPFSG
QIIKVIQPSKLNLVINERLVIEFYSRMGQKVNPLGFRLSTKKYNNKIWFKNFTEYSFFIKE
ETLIYKYLKEKINKIKFKRNKILIIFIYSIFPFLILLKFLKTKTIILIKNIKVKSPFTKANLLAN
AIAQQIKNRVSFQRILQIFIIKKILGIKIQLAGRLNGAEKAKTEWYRKGRIPLQTLFANIE
YSQQMVKTKYGIIGIKIWLFKMINDIISDLLTRIRNSLKVYAPIVCVPITRLSFSILLILKT
QKFINNFKIIKISIFLIDLKYIKNNTQSKILKLIRISKLSLRRYINSNLLFTPFNSLGFFIISTS
KGIMTHTKAKTLKLGGEILFFIQMYISKIVEVQPVTKVGKGRKLLRFRVCLIMGNKSGII
GFGTGKDKNFFNSKLKGIEKAKKNLIELSLTDHKTITSGVTGKFRSAFIILKPAKSGTGL
RAGNTIRTIFELAGITNIIAKQLGSCNILNNVVATFLALQSIMSRCSFTLKKKVFFDKYY

KSFLVTLLIQFLLKNGKKNIAMKIVYKTFIYIEEQTQKNALKIFEQALFNTKLLFEFKNN
KDKNNTDQIPYEITSFRSILLAIKTLIKEAKKSISYTFSIKLANEIIGAANGFGKLVKNAEA
NKLLAKTYQPFLYFDRMIIIKLQSFNITRLIITVTRICAILKKQKCNFSGPISLPTKKRKYC
LLKSPHINKTAREHFEIKIYKKILKIF-
PKLYINLNFFFPSGVDLKIMPTIQQLIRFSRAKKSKKIKVPALKNCPQRRGICTRVSVVS
PKKPNSALRKVARVRLTTGFEVTAYIPGIGHNLQEHSIVLVRGGRVKDLPGVRYHIIRG
ILDTNGVTDRKRGRSKYGTKKI-
MCTSKLTNKLGRIYINFTKNNLLFTITNLTGDVLMWTSSGVAGFKHSRKKTSLALQE
TILCCIKKLKKLNLRFVLIFIKQVGFNIDILLNLLDFNSLTILNIFEITTFSFNGCRLPKKR
KIMIQLFGLTLNTNKKIKNSLLNIYGLGFSSIKDILLKANINNKKIIELSKMHLIRVRKIIE
NYIIQKFVQRIIRTNILKLIRIKSRRGKRHQAMLPVHGQRTRTNARTRRGKKKTKNMFS
NYKIGYVISQNSFKTIRVIIISTFVHPRYKKLIKKINRFLVHDYTEKCQIGDKILISEQRPIS
KKKYFYVKMSKKSILERSKKKYKLNKVHHIKRKEIKIKQKNEKNIIKLFSLEKIIQKLVKN
SALTRFKRFCWVTGRNRGYYRDFSLSRHVLREFAHKGNLPGILKSSWMIKNLKKIPFIY
YKLLKKYNYNKKNSIKTWSKGSVILPCMINSTFTIYNGKQHISLVITDEHIGYKLGEFIP
TKFFYSHKKEKKLKKMIKQYKFNLKKIKRQTQLNKIYISRIKTFSKKCFIYIKKYITKNLN
LAYCALDKSLKKKIFHKNTISRKKSKLQTLVNLMTQIFTTTGETYAITLLKIFKGFIIQ--
QNFRLNLT---
VGYKSPIKCKKPNLYHFEKFNFIPFKLIKTYKIKKQKFSLGQIIDSTNFKISEVISVRGRTI
GKGFSGTCKRYNFNSGPLTHGSKNHRKPGSIGQGSTPGRVFPGKKLAGNLGNNYSTL
FNLPIIALNYTENFLIIKGTMGTLKTNLLINKMPKIKSLRKIINLVCITC-
QQNKDIAGFSHYTTVKNKKNTLKRLILRKFCFFCKSHQFYHEIKMTQIFTTTGETYAI
TLLKIFKGFIIQ--QNFRLNLT---
VGYKSPIKCKKPNLYHFEKFNFIPFKLIKTYKIKKQKFSLGQIIDSTNFKISEVISVRGRTI
GKGFSGTCKRYNFNSGPLTHGSKNHRKPGSIGQGSTPGRVFPGKKLAGNLGNNYSTL
FNLPIIALNYTENFLIIKGTMGTLKTNLLINKMKKFVKYNVINLGSVLNLSILKSGNYVV
HKDVSRQQIQQKQGTVSTKTRSEVRGGGRKPWRQKGTGRARAGSNRSPLWKGGGV
IFGPKTKKVKVKVNKKERRLALQTLLYSRQNDIFIIESLQTLKTKPFIEVCLFWDKKVLL
IVSEKTHQLKFSTRNIKNIELISVSHLNTLSLLKAKKIYITTNAITKIKETFMLKFRLIRGG
KQNNPFYKIGILDAKTKRNGQPLQILGFYNPIKKIIKLNIYILLKNLKTGVKLTYRLWIL
LLKLKMLKKPVTFNDKSIHYINSQKYSFLVNTCINKLFLKKIFEYLCKNYCKHINKLR-
IKKKFYNITLTKPI-NLFLIFIFQRI-
MKFEKSSILKLFIGDYITVGIKFPKNKKFRIQEYSGLIISKKNNNLNSTVQIYNIKKYSAIQ
YCFFLNSPTILFLKIHYSLKFKHSKLYYLYKK-----------
>Pseudo-nitzschia_multiseries_KR709240.1
MIKKEYPKLKKIQINRGLGLAAQNTSILKKNIEEFEKISGQKPIITRSKKAIAGFKIREDM

ELGLSVTLRGEKMYAFLTKLLFFTFAQIRDFRGLSLRSFDKAGNYTFGLKEQLIFPEINY
DDVDQARGCTITLVLEHSSPKNGMILFKFLRFPLMVRVKRGNVARKRRKKILSLASGY
RGAHSVLFRVANQQVMKALRYSYIGRKQKKRIFRKIWIRRINAASRAITYSQVINKFKKS
NIDLNRKMLSQIAILDSSTFQSLLMSIRLYKSYTPGTRHRALSAFTEITKAKPERILIKKN
HRNKGRNNRGVITIRHRGGGHKRRYRLIDFKRNKYGINGIVKSIEYDPNRNARISLINY
TDGEKRYILHSKGLNVGDTILSGSGSPFSIGNTLPLSEIPLGTSVHNLELIPNKGGQLV
RAAGTSAKILAKEGDYVTLRLPSKEIRLLRKECFATVGEISNNDAFLVQSGKAGRTRWL
GKRPAVRGSVMNPCDHPHGGGEGRAPIGRTRPLTPWGKPALGMKTRKKKKSYILRR
MSRIGKLPIIIPANVDVNWNGEIITVKGKFGTLETAIPQTINIENIITVSRSLRSMHGLYR
TLINNMVVGVSEQFEVTLILKGVGYRAAVQGKQIVLNLGYSHPVNIDVPETVSVEVVQ
NTTINLKSCDKELLGLFASNIRSWRRPEPYKGKGILYKNEQILRKAGKAGKLTFLPTQD
NCNWYVIDCSGQTLGRLATVIAGLLKGKNKAQYHPSVDTGDYVVLINAEAIVVETAK
HYIVRNPGRPGRSLRNVVDCLPRFTIERAVKRMLSNTGKRLMRRLKIYNGDTHVHQA
QNPITIMAKKITALIKLALPAGKATPAPPVGPALGQHGVNIAAFCKEYNAKTGDKAGL
IIPVEISVYEDRSYTFILKTPPASVLLVNAANIKKGSSKPNLIKVGSITKAQLEEIATVKLP
DLNTTKLESAMKIVEGTARNMGISIMLSPKRTKYRKYHRGRMRGKATRGNQICFGNF
GLQALEPTWITSRQIEASRRTITRYTKRGAKLWIRIFPDKTVTARAAESRMGSGKGAV
DYWVAVVKPGTIIFEISSVPEEVARAALSLAAYKLPLKTKFINKMIYPQTILTVADNTGA
KKVMCIRILGGNKKYAEIGDTIIAVVKEAIPNMPIKRSDVVRAIIVRTKKTIRRQDGMYI
RFDDNAAVIVNPENNPRGTRVFGPVAREIRDKNLSKIVSLAPEVLMYAIVEISGRQFWI
ESGKYYDFNRIPTGKQITLNRILLLNNVLIGQPYLEVIKGKILEHFRGKKKIVYKMRPKK
KTRKKQGHRQELTRVFIMVKAVAKYIRMSPHKIRRVIDQIRGRSYQEALMILEFLPYDA
GGPIWQVVHSAAANAKHNYGLDKKKLIINEIFADEGPKLKRIRPRAQGRAYKILKPTC
HITVVMMPKTDLHPEWFSQTPVLCDGKPLGLIGSTKPELQVDMWLANHPFYTKSQV
MIDSEGRVERFMKKYGLMAHKKGAGSTKNGRDSNAKRLGVKKFGGEKVKAGNILIR
QRGMKFKPGLNVGCGKDFTLFALTEGTIKFDINII------------------------------------
MAVPKKRTSKAKKNARKANWKRKGAVAAQKSFSLAKSMLKPTSFVYIVKCLKSDKEH
GQFLINSLKSGQGITIGNQLRRVLLNDLGGIAITAVRIAGVSHEFSTIPGVREDILEILLN
LKGVILRGNFGRLKIQGPMVVTADSIQLPSDLELVNPNHYIMTISTANVIEIEFKFEYGT
GYKLAYLQLDAIFMPVQKVDFKIENITERLFLDIWTNGSISPDEAFKLAAQVTIDLFSLL
IAIEELQLSVRAYNCLKKAQINTVGDLLQYSPLQELFGRKSSIEVFSTLKNQNTLIDKK
QLRQLLAWSFTSYDSMQACLLADELKYLGFKYASHAGISISIEDLKIPFVKNVMLEKAN
QEIYNSEKIFLKGKITDVERFQKIIDTWSLTSESLKNQIIYYFKNYDPLNSVYIMAFSGAR
GNLSQVRQLVGMRGLMSDPSGQIMNLPIKKNFREGLTITDYLMSGYGARKGIVDTAL
KTANSGYLTRRLIDVSQDILIREKDCLTPHSFVFILGRVLNTPIYLVKQNTQITPELIQT
FKKILEYSVRSPLTCQLYRAICQKCYGWDLANENLIDIGEAIGILAGQSIGEPGTQLTM

RTFHTGGIFTEARQQIISPMNGIIQFYKRLKTVILRTNRGEDVLVTKNAGSLVLIDLVKI
DILRNTILFPKNNQYLPKDTVIGEVINTNKQILSDTCGEIFINHLIWILSGQLYNSPNNF
YPDHKLNPSYVFRTKLINHYSGSVKNYLLTGGMILEEEIHKIKCEILFIGDFIFELIPNLFS
KTSGIVTIDTQIVSIKSGGVYYPGEALFSNVKIQNLSFCELVRPIQIYEFVNLIANLLLFID
PCFLVQKNQFVSNYNILGYLETLTNSLEIVKFKIKEKQIFLISNNDCIVVNKAETKSDFI
NLAKTGKIIIENAEFVTIQKGRPYFFQDVNSVFCKNTEFIESGKTLGLLNLEKEITGDIV
QGLPRIEEILEARKKNTSQKRAQKLGTPIKINPHKLVKVYFNYYEACHKSFKKVQLFIL
NSVQSVYKSQGVTINDKHLEVIIKQMTTKVLITYEGDTPLLQREVIDLYHIQYINNILLS
ECYVPLLLGITKAALNNPSFISAASFQETTRVLTKAAIEGRIDWLRGLKENIVIGHLIPA
GTGMYINALPDFLAMQRVSFCWFITQGLTEELALFSKIYDFQNTEYLIFSQEYLLIKPL
YNLIIAKKYNGNYRAQLVIPIEVRNKVVHYQNQFPIITLPLMTTYATFIINGCERVIVSQI
IRSPGIYFVYSATLIPEYGSWIRFNFQKLIIQLDKITQKPILILLKEMGLTEIYQNLYYSDF
FYFSEFSQIFDPNYYNLGKVGRLKINNRLSLKISNRLQIITYEDIFAIIDKLISLTISKDDID
HLKNKRVRSIGELLQNLFRIGFQRLVRKLRNQTNTFVIATIREFFGSSQLSQYLDQTNP
LSSLTHRRRISGLGPGGLNRDHISFSVRDIHPSHYGRICPIETPEGQNVGLIASLTTCAR
VNKLGFLETPFWRVINGKVLKTGNPIYLTAEVEDLYKIAPADIATNYLIKNIISVRYKQD
FINVTPSEVDFISISTIQVVSVAASLIPFFEHDDANRALMGSNMQRQSVPLIFPQKPIVG
TGLENQIAIDSGMTLNAHISGIVNSVTANKIIIKYKLQKYLRSNQQTCINQRPIVWKGE
KIQSGQILSDGPAITSSELSLGQNTLIAYMPWQGYNFEDAILISERLVYDDIFTSIHIERY
KIEINRNAEISEMKMIPNLVKHLNDDGIVRVGTFVKSGDILVGKVVSTNAYEQLPESKL
LRAIFVKDNSYRMPVIETVTFNRVFIAQIRKIQVGDKIAGRHGNKGIISRILARQDMPFL
PDGTPIDIILNPLGVPSRMNVGQLYECLLGFAGDKLNCRFKILPFDEMYGLEISRILINK
KLRQASKIKVGFLIP---------------------------MLQV--------------------------------------
KW---
MFNYIKIKLASPFRILQWSNRKLP-
NGFVGEVQKPETINYRTFKPEMDGLFCERIFGPSRSFECACGKYKICERCGVELTESRV
RRHRMGYISLVYPVTHVWYINSRPNFMALLLIKRIKGAPLIKRELEKLNLESEISRTRCF
VLVCTKIREKAIKRIRILENLIGTASEPSWMILSILPIIPPALRPMIQLEGGRFATSDLNEL
YRRIITRNNRLLRLLEIDAPQLIIRNEKRLLQEAVDTLIDNGKRGKIALSANNRPLKSLS
DIIKGKHGRFRQNLLGKRVDYSGRSVIVVGPTLKLNQCGLPYEMAIELFQPFIILINQN
AKLIQSDLTIKPVVQILKNHPIFLNRAPTLHRLGIQAFEPILVKGRAIKLHPLVCSAFNA
DFDGDQMAVHIPLSPEAQAECYMLMLAPYNFLSPANGEPIIMPSQDMVLGCYYLTA
NNIKGLLGSNYYFANLEDVILAYNQDKIEIHTSIWIRYVITQYLQTTTGRVILNYTIQKT
LMSRYRGPRLRLVRRFGLPGLTTKKSIKEGGQKTEYGIRLNEKQKLKFNYGLTEKQLF
RYVKEARRRQGVTGLILLQLLEMRLDTLCFTLGFAKSIAQARQLVNHGHITVNNKVIS
IPSFQCRLNDVISVKERSVSKNLVTNNLIPANLEFNDAKLEATVLDYCDRDDVQLQLN

ELLVVEYYSRMGQKTHPLGFRLGIVQEHKSVWYANFNQYANILEEDDKIRTYINTIISN
VKICRNDHIQLTIETGKPILVLLNLKKLLRQLTIKVSEIEFEDLDAKLLADLVAKQLEKR
VAFRRATRTVLQRARNGIKIQVSGRLNGAEIARSEWVREGRVPLQTLRADIDYATTEA
NTIYGVLGIKIWLFK---

MFAERLIKISRVSKVTKGGKKLSFRAVVVIGDEKGKVGVGVAKADDVVNAFKKAKAD
GHKNLIELPITKSLSIPHNVVGNYGACKVIMRPSIEGSGVIAGGAVRIVLEVAGVKNVIA
KQLGSNNLLNNARASICALQNLMSRRNISKKRFPETDSVYNSYLVSLLITRILKSGKKTI
AKKIVYDAFEIIQQKTNDDPLKVFENAIRNASPVVEVKARRVGGSTYQVPIEVSRFRAT
NLSLRWIIQYSKQRVGRSMAIKLANEIIDTANEIGNTIKKKEETHKMAEANKAFAHFRR
MVRIRLESFNHELLVSSCRKITQTLNSASLNSVGMVTLPTSKRIYCVLRSPHVDKDSRE
HFEIRTHKRILEIYSDSSVVDLLAELDSGVFCSIMPTIQQLVRSRRIAIKKKTKSPALVNC
PQRRGVCTRVYTTTPKKPNSAIRKVARVRLTSGFEVTAYIPGIGHNLQEHSVVLIRGG
RVKDLPGVRYHIVRGALDTGGVKDRRQGRSKYGVKKPKMRKDKSNLRNGVVHIQST
FNNTIVTITNPNGDTVSWASAGSSGFKGARKSTPFAAQTAAEKAALDAVSIGLKSVDIL
VKGQGSGRETAIRAIEGAGLEITSIQDITSVPHNGCRPPKRRRVMVRLVGVDLPRNKKI
AYALTYIHGIGLTSAKKIVEIADIDDTRVYDLTTEQAVALRQTLEDLKLEGDLRRFNGL
NIKRLNEINCHRGKRHRNSLPVRGQRTRTNARSRRGVKKTKKMPVKQQVGIVVSNK
MQKTIVVKIENRYPHPMYSKTLVKTKKYLAHDELETCNIGDQVLVQECRPLSKRKRW
KLVMAKKSMIEREKKRIRLEKKYAQKRTNLLNEYKNEGNFNKKMEIHSKIQQLPRNSA
KTRIRNRCWKTGRPRGVFRDFGLSRHVVREMAHQCLLPGVTKSSW--------------------
----

MNKSAKKRIEIAKRNRLQNLYYKTSVRSLTKLFFKTLKQ-
LQTILNSIYSYLDKGTKKRFFIKIQQLERKQDLLMSFQQMTQIFDESGNIIPVTILKVGP
CMITQIKTESKDGYNAIQVGYSNNKSLTQSELGHLQKSNIQPLKYLKEFRVPNDDFEV
GQVLNVDLLSVDQLVNIKGKTVGKGFSGLQKRHNFTRGPMTHGSKNHRAPGSIGM
GTTPGRVLPGKKMAGQLGNKLTTIKKLKVIQLNSEENILIIKGSVPGKPGNLLSITMAK
NKGTRILITLECTECRSNNKRSPGVSRYTTQKNRRNNPERLELKKYCPNCNKPTVHKE
IKMTQIFDESGNIIPVTILKVGPCMITQIKTESKDGYNAIQVGYSNNKSLTQSELGHLQ
KSNIQPLKYLKEFRVPNDDFEVGQVLNVDLLSVDQLVNIKGKTVGKGFSGLQKRHNF
TRGPMTHGSKNHRAPGSIGMGTTPGRVLPGKKMAGQLGNKLTTIKKLKVIQLNSEE
NILIIKGSVPGKPGNLLSITMIKTFEYSIVAQSNDFSLDVSDKAKYVVHRAIRTATTNRA
QATSSTKTRSEVRGGGRKPWKQKGTGRARAGSTRSPLWRGGGVTFGPKPKLINKKIN
KKEKQLALRTLLYNRNEQIKVVKEFFDAKTSDLLRTLLSENQKTLVISSKPNKNLQLSL
RNLKNFNYILANQLNVNEIAKANQIIIDELSFQVIKETYMLKLRLKRIGRKRAPSYRLVV
MENSARRDGRPVEEVGYYDPISKKSHFDIPKIEKWLSYGVQPTETVFNLLKKIV---------

----
MGVEKEDIPPVRIGDNIKVGVKIIEGSKERVQFYEGTVIAKKNSSINTTITVRKTFQGIGI
ERIFLIHSPKIDSIEILRSSKVRRSKLYYLRNLKGKASRLKQTF
>Pseudopedinella_elastica_MK518353
MFKELYPKIEKIQINRGLGVSAQNTKILKKSVDEFRSITGQQPVITKAKKSIAGFKLREE
MELGVTITLRRDRMYSFLDRLINLALPRIRDFQGLDPKKFDKYGNYTFGILDQLIFPEI
EYESVDKTLGFNVTIVTSAKNAKEGLALLTKMGLPFMVRVKRGNVARKRRKKVLSLA
KGFRGAHSRLFRVANAQVIKALKYSYVGRKNKKRQFRKLWIVRLNAASRIINYSKFIHKI
KKSNIELNRKMLAQLAIVDPATFEKLTMAIRIFKPMTPGTRNRGVSTFNEITKSKPEKT
LIRVNHRVKGRNNQGRITIRHRGGGHKKLYRLIDFKRNKYDILGKVASIEYDPNRNARI
ALIHYEDGEKRYILYPQNLKVNDFVSSGADAEIQIGNSLALEDIPLGLDVHNIELTPKR
GGQIVRAAGTSAKILAKEGNYVTLRLPSKEIRLVRKECFATIGVIGNGDFNNMTIGKAG
RTRWLGIKPTVRGVVMNPCDHAHGGGEGRSPIGRKRPITPWGKPALGVKTRKNNKS
YIIRRMSRIGKLPIGIPKNVNVTLNGTKIEIKGPNGSLDREIPELIGVDDKLIIVRLARQQ
YGLVRSLINNMVIGVSTKFEKKLQMIGVGYRAQVQGKELTLSVGFSHPVVFVIPQEID
VKVETNTNLTISGADKAAVGLLASQIRAVRPPEPYKGKGIRYTNEVVLRKAGKSGKMT
FIPSKNNKKWYLIDAENQTLGRLATKISRVLIGKEKAMYTPFLDTGDYIIVINAEKISLK
EKQKIYRNHSGRPGETLEKLRERRPEKIIEHAVKGMLPKGGRKLYTNLKVYKGEQHPH
DAQTPELLMAKKVVAIIKLALPAGKATPAPPVGPALGQHGANIAAFCKEYNAKTADK
GGLIIPVEISVYEDRSYSFILKTPPASVLLIKAANIQKGAGEPNKSTVGSITQIQLEEIAKT
KLPDLNTKNLESAMRIVKGTAKNMGITVMLSPKRTKFRKQHRGRMKGKAYRNNKISF
GEYALQSQEPGWITSRQIEATRRTITRSIKRGGKLWITIFPDKPVTARAAESRMGSGKG
AVDYWVAVIKPGNILFELGGVPLELAQKAMKLASYKLPVKTKFITKMIQPQTYLTVAD
NTGAKKIMCIRILGNNKQYGSIGDVIIGVVKEATPNMPVKKSDVVRAVVVRTSHTLRR
KDGMSIRFDDNAAVIINKENNPRGTRVFGPIAREIRDRNFTKIVSLAPEVLMYAIVEIGG
HQVWVEEGQHFITNKILTGSKISINRVLLVNNLHLGHPYLDSVTGEVVEHLRGSKVIV
YKMKSKKKYRRKKGHRQEMTKLLIMAKAVGRYIRMSPRKVRRVLNQIRGKTYKEALM
LLEFMPYRACGPVWQVIYSAAANAEHNLNLKKENLIISEAFADQGPVFRRFRPRAQGQ
GFGIRKPTCHITITVMPKKDIHPNWFDETQVYCDGQLVYTTSSTKPRLNVDVWSGN
HPFYTGSLTILDTEGRVERFLKKYGLMAHKKGAGSTKNGRDSNSNRLGVKIYGGASA
LAGNIIVRQRGLKFKPGSNIGVGKDFTLFALKTGTVEFQINIIMKVRPSVKKMCKDCRI
IRRQGVLRIICKNPKHKQRQGMAVPKKRTSRSKKNSRRATWVNKANTQAKKAYSLAN
SVLKSTSFIY---------------
MTNLTPGQGLTLGNALRRVLLANLEGNAITAIRIPNASHEFSLIPGIREDILEILLNLKQ
IILKTKSGKIIAKGPGIITANCLKFDNEVEIINPNQYIAAISTAQNIEFDIIAERGVGYQLA
FLQIDAVFMPVVKVNYKINNTNESLLLEITTNGSITPEKAVSEAAKKLMIWFSSLILIEEL

QLPVRAYNCLKRAGINSVDELIQYSQIKEIFGKKSADEVFQALKKKNQIFSKKELKSAVY
DAFTNYGITRSCLLADEMKELGFNYATTAGISISIEDLKVPPTKKKLLVESNSEIKKSDL
TYARGEINSVERFQKIIDTWNKTSETLKNNLVDYFRQTDPLNSIYLMAFSGARGNLSQ
VRQLVGMRGLMSDPNGQIIDIPIIHNFREGLTITDYIMSAYGARKGVVDTALKTADSG
YLTRRLIDVAQDVIIRENDCLTKRSIKLIIGRTSAQTIKIISNNQQITETIANKFNYIKSIKL
HSPLTCESTRSICQKCYGWNLSTCKTVELGEAIGIIAAQSIGEPGTQLTMRTFHTGGIF
TDPSRQIRSRTTGFFSFDPNIQIKLGRTMYGSKVTILEREAKFKITKTNETKLPADSSIFI
QNNSFVKTGDLIAELPLKNQQIIAPHSGQLIALNLAWIMESEVYNIPNNFKSNQNITKD
NLIQFKLISKKDGIVQKLVIKGGLLVPLEIHTINKDLLLVLTKLTELIKGIFSKTNGILQT
ILQEIQIKPGKVYFPGEIIFEDILIKYLSLIEILRPIQAFNIISLIESSIWLLKISIFVKKFEYIEL
QTLLGLISIVPTILKVQNIHKTNPKLLITSQKNYKTYYNEGSLMDKIKINCSGRILTQDP
FKFKVHQGTPFYVTPETGLTNGKNNFIKQGEVWGIINFEQIVTGDIVQGLPKIEEILEA
RKPQYPDKIGNILGQALTINPHNLLIVYFNYYDAAYMSFKNVQLLLIQKVQQVYNSQG
VSIADKHLEVIIKRISSKVQILNSGASALLPGEIIELKQINYINNILLQANYNPILLGITKAS
LLTDSFISAASFQETTKILTAAAIEGKIDWLRGLKENVIIGRLIPAGTGMFLTTLPDFVEI
QRASFCWFLLKGLSEELAKFSSITDLNNIELNFFGQEYKLRKPKYGILEAKRRDSTYGV
KIYVPMSITFFSKNPQTQAFIGELPIMTDRGTFIINGCERVVINQIIRSPGIYYLYSATLIP
NRGSWLKFEIDKLTISIDKTEKIKAELFFNTLNLTEILGNFTYPEFLENKELNKIFNPKYY
EIGNVGRYKLNDRLNLNIPQHINNLTIQDIFAIIDLLISFKYNKDDIDHLQNRRVRSVGE
LLQTQFRSGLTRLERILSERMTITFVSVMREFFGSSQLSQFLDQTNPLAELTHRRRISG
LGPGGFSRDHVSFAVRDIHPSHYGRICPIETPEGQNAGLISSLSVYARVNSFGFIETPFF
KVKNGIVLYKQPPIYLSAEEEEQLKIAPADTRIGKIIPEFITVRYNQEFIIVGAIEVELIGVS
TIQIVSSATALIPFLEHDDANRALMGANMQRQAVPLLYPTKPIIGTGLETQLATDAG
MVVISYTNGYVTYTSSKAITLTYALQKYIRSNQDTCINQRPIVWNGEYVKSGQIIADGP
GTDGGEIALGQNIIVAYMPWEGYNFEDALVVSERLIYADLFTSIHIEKYDLEIRETKAG
TETKDLPNVTKHLDENGIIYRGKFVKAGDILVGKVTPTEEADEIPEGRLLRAIFVIDNS
LRVPVLDIRIFSRIFLAQIRKIQVGDKMAGRHGNKGIISKILSRQDMPYLPNGQTVDLIL
NPLGVPSRMNVGQLFEGMFGFAGDTLDRRFKIFPFDEMYGPEASRILVNNSLKKAKS
KNHWIYNSSSPGKIRLKDGRTGEYFHNPITVCKSYILKLVHLVDDKIHARSTGPYSLVT
QQPLGGRSQQGGQRFGEMEVWALEAFGAAYTLQELLTIKSDDMNGRNEVLNAIPQP
GIPESFKVLILELQSLGLDISMYKMQKDVGMEVNIMMFDYIKINLASSKRIKQWGERKL
P-
DGIVGEVTKPETINYRTFKPEMDGLFCEKIFGPVKNWECYCGKYKVCERCSVEVTEAR
VRRHRMGYIELISPVTHVWYLKGIPSYLSLLLKNNLDGAEAIKYLLEKIDLKKEIIRNRLS
LQIINEKPEKIIKRIRLIENFIATNSHPVWMILNVIPVIPPGLRPMVQLDGGRFATSDLNE
LYRRVITRNNRLSRLLKIYAPAIIVHNEKRMLQEAIDALIDNGKRGRKALGANNRALKS

LSDIIEGKHGRFRQNLLGKRVDYSGRSVIIVGPSLKLNQCGLPHEIALELFQPFIILINNG
AKLIQTGPIIWTILNILKNHPVLLNRAPTLHRLGIQAFEPILVAGRAIQLHPLVCTAFNA
DFDGDQMAIHIPLSTEAQAEAKRLMLAPNNFLSPATGEPIILPSQDMILGCYYLTVDN
PNAKKSQNNYFSNYKDVLFAYEQKIITAHTPIWVRNELEKYIRTTAGRILLNKTIMKNI
MSRYLGPRLRITRRLGLPGLTRKTTKRAKKASSEFAIRLQEKQKLRMNYGVSEKQLST
YVKKARRTSGVTGTLILQLLEMRLDNIVYRLGLAPTIPSARQLVNHGHVLVNGKNLDI
PSFQCNPNDVISVKSK--
SIPFVKNQGLPSHLEFDNDKLVAKIKSIVAREEINLEINELFIVEYYSRMGQKVHPLGFR
LTTTQKHRSVWFAEFQNYPKLIEEDNAIRACLKTRIAKIEIKRNNKIEVTVHSGRPILVI
YELKNVVVNIIVNIVEIKNPDAEAELIGDFIVEQLEKRVAFRRAVRKGIGRAAQGIKVQV
AGRLNGAEMARTEWIREGRVPLQTLRADIDYAEKRANTIYGVLGIKVWLFKMVNDTI
SDMLTRIRNANNAKHHLVQIPATRSTRAISKILKEEGFIHDFEFLVQESILISLKYIGKER
KPIITKLERVSKPGLRIYSKAKNMPSVLGNLGISIISTSQGLMTNINAKALNIGGEILCTIY
MWNERVIQISRVTKVCKGGKKLSFRAVVVVGNEIGQVGVGVGKADDVINAITKGITD
ARRKVITVPITKTNSIPHLTIGNFGACQALIKPAREGTGVIAGSSIRTVLELAGIKNILAK
QIGPNNILNSARATICALESLMSRRSIVKKRYPLADSQYNSFLVSLLTARILKKGKKRLA
DSIVFETFELVRERTSSEPIQIFETAIKNTTPIVEVKARRIGGSTYQVPLEVSRYRGTTLA
LRWIIKAAKDRAGRTTSLKLASEIIDASNGIGNAVRKKDETHKMAEANKAFAHFRRMI
RIRLQSYDHKLLDNACQQIINEVEKTQSKVVGPIPLPTKKRVYCVLRSPHVNKDAREH
FEIRIHKRVLDIYPTSDTSNSLINLPSGVSTTIMPTIQQLVRSPRKTIKIKTKSPALQNCP
QRRGVCTRVYTTTPKKPNSAIRKVARVRLTSGFEVTAYIPGIGHNLQEHSVVLLRGGR
VKDLPGVRYHIIRGALDSGGVKNRNQGRSKYGVKKPKMKKAKLKIVAGVVHINSTFN
NTIVTITDPKGNTISWASAGSAGFKGARKGTPFAAQTASEAATSTAVNLGLKTVEVLV
KGQGSGRETAIRAVQNGGLEISAIKDITSVPHNGCRPPKKRRVMVRIAGTDLPRSKRIE
YALTKIYGIGIISSQNILAKANIDNIRTNDLEDSDVSKIREILEEYLVETDLKRKVNQNIK
RLSEINCVRGRRHRENLPMRGQRTRTNARTRRGAKKTKKMATKEKTGIVVSTNMDK
TIILIVEEKYAHPIYGKILKKTKRFMAHDENNECVLGDTVMISETRPLSRKKRWAVKMA
KQSMIQRELKRNKLVKKYAKKRKQALTNLKTAKNLTEIFSMQRTLQRLPGNSSPIRLK
NRCWKTGRSRGFYRDFGLSRHVLREMAHECLLPGVVKSSWMGRSLRKGPFVAYHLL
KKINEMKKSVIKTWSRASTILPSMVGHTIAVYNGKQHVPIFISDQIVGHKLGEFAPTRT
FRSHKSDKKARRMIKSAVKRINVTKRNNLRNKQYKSNIKTFTKKYLVALNTYAIKNLNI
VYSKLDKATKANVLNKNTAARKKSALKKAFNSMTQIFTETGDANPVTVIKIGPCSITQI
KSVATHGYTAIQIGYQPKRKLTKPELGHLEKTNAAPFKYLKEYRVPEHTFEVGQIIDV
NNFNEGEFVDVTGKSIGKGFAGNMKRHNYGRGPMAHGSKNHREPGSIGMGTTPGR
VFPGKKMGGRLGNQNVTISNLKIIKIDIQENLLIVKGAVPGKPGNLLSVRMAKSKGTR
LVITLECTECRSNTKRSAGVSRYTTKKNRKNTPERIELKKFCRYCNQHTNHKEIKMTQ

IFTETGDANPVTVIKIGPCSITQIKSVATHGYTAIQIGYQPKRKLTKPELGHLEKTNAA
PFKYLKEYRVPEHTFEVGQIIDVNNFNEGEFVDVTGKSIGKGFAGNMKRHNYGRGPM
AHGSKNHREPGSIGMGTTPGRVFPGKKMGGRLGNQNVTISNLKIIKIDIQENLLIVKG
AVPGKPGNLLSVRMMQTLNFEVLKETTKLTVRVQQGANYLVHKALTNQSLNRRNFT
AATKTRADVRGGGRKPWKQKGTGRARAGSNRSPLWKGGGVTFGPKPKKVYYKLNR
KERQLAIQTLICNKQKQILALTNLLTQKTKKITELIVDLNKQTLIVTSQFDKTLLKAVR
NLPHVNTVQFNHINLEKLIHANTIIIEAKAIEQMGELYMLKMRFKRGGKKRQPSYRIVL
MESTSARDSRAIKELGFYNPISKEVKLDVPSIVKYLKYGAQPTETVKNLLIKILMLIKYPV
ISDKTTRLIQGNKYTFMVDKRANKFTIKKIVEYVFDVNVINVNTLRSPRKKRTRPQYK
KAIVTLKAGDSINLFPMNIEKPNVPNIRVGDNVKLGVQITEGSKTRVQAYEGVVISIKN
TGINKTITVRKIMQGIGIERCFLIHSPKINSIQILSSAKVRRSKLYYLRNLSGKATRLKQRF
>Rhizochromulina_marina_NC_043890.1
MLKLQYPKIIKVQINRGLGTAAQNSKVLKKTVNEIRLISGQQPIVTIAKNSIAGFKIRED
MPLGVTVTLRREKMYSFLERLINLALPRIRDFQGLDPRKIDKHGNYSFGITDQLIFPEI
DYDQVDETLGFNITIVTTAKTTKESIALLQKLGLPF------------------------------
MFRVANQQVMKALKYSYMGRKRKKRQFRRLWISRINAETRSLSYSLYIHQLKQANIGL
NRKMLSQIAILDPTTFKQIIMAIRIYKPNTPGTRNRGVSSFRDITKTKPEKTLTKANQR
AKGRNNQGRITIRHRGGGHKRLYRIVDFKRNKDSILGIVATIEYDPNRNARIALVNYM
DGEKRYILAPQNLNIGDRICAGEKAEISIGNSLPLEQIPLGLDVHNIELVPYHGGQLVR
SAGTSAKILAKEGNYVTLRLPSKEIRVVRKECKATIGIVGNGDVTNLNIGKAGRTRWL
GIRPTVRGTVMNPCDHPHGGGEGRSPIGRKRPVTPWGKAALGVKTRKPKKSYIVRR
MSRIGKLPVKIPAKVKVDITNNQIIVKGPFGELSRNIPDGVSLDDRVIVTRFNRQKHGL
VRSLVNNMVIGVAKKFEIQLQMIGVGYRAQVQGKNLNLNIGFSHPVVFTIPDSIEIAIE
ANTNLTIRGIDKEQVGLIASQIRAMRPPEPYKGKGIRYTNEIVLRKAGKSGKMTYISTSS
KKKWYLIDARDQTLGRLAGKISVILMGKNRSTYSPHQNNGDSIIVINAEKIYVKENQK
MYRFHSGRPGESFKSLLARKPERIIENAVKGMLPKNGRQMLTNLKVYKGTLHPHDAQ
MPEVIMAKKVVAIIKLALPAGKATPAPPVGPALGQHGANIAAFCKEYNAKTADKVDLI
IPVEISVYEDRSYSFVLKTPPASVLLVKAANLKKGAAEPNKVTVGSVTLAQLEEIAKIKL
PDLNTQNVASAMRIVAGTARNMGITIMLSPKRTKFRKQHRGRMKGKAYRNNKISFGE
YALQAQEPTWLTSRQIEATRRTITRSLKRGGKLWITVFPDKPVTARAAESRMGSGKG
AVDYWVAVIKPGTILFELSGVSSDLAKNAMKLAAYKLPIKTKFLIKMIQPQTYLTVAD
NTGAREIMCIRILGNNKKYGYIGDVIIGVVKEATPNMPVKKSDIVRAVVVRTKKTLRR
NDGMSIRFDDNAAVIINKDNNPRGTRVFGPIAREIRDKSFTKIVSLAPEVLMYAIVEIGG
RQIWVENGNYFFTDRLSIGTNVSLTRVLLANKLHLGHPYLKGVVGEVLEHVQGPKLIS
YKMKSKKKYKRKQGHRQLLTKLLIMAKAISRYIRMSPKKVERVLNQVRGKSYKEALML
LEFMPYRSCGPVWQVIYSAAANAQNNYGLSKEDLYICEAKADKGPILRRFRPRAQGRG

YAIRKPTCHISIKLMVKKEIHPKWFEETQVYCDGQLIMVTSSTKPELQVDIWSGNHPF
YTGSLKILDTEGRVERFMKKYGS--------------------
MGVKVFGGAPILAGGIIVRQRGLNFKPGTNVGCGKDFTLFALKPGIVKFEVNVLMKV
RPSVKKMCTNCRVIKRNGIIRIICKNPKHKQRQG--

MFQCLESTVETSKFLLRSLRYGQGITIGNALRRVLLSDIMGTAITAVKIPTMVHEFSTV
DGIREDILEIFLNLKQVILKRSTGAINIKGPGIITASHIKFNKPIQIVNPNQYIATLGTATT
LIMELKVESDVGYRLGFLSLDAVFMPVINVNYKVNTSKEYLIIEITTNGSLTPAAALNEA
ATYLVSWFQGMILIEELQLPVRAYNCLKRVGIKSIEDLMNYTQIRNIFGKKSAQDVFRA
LETKNKIFTKKELKQVMYETFTNYGLKQASYLADNLKSLGFQYATKAGISISVEDLKIT
PTKSEILRMAQTEIRLSNDRYVRGEISSIERFQTVINVWNNTSESIKDSLVDYFTQTDPL
NSIYLMAFSGARGNLSQVRQLVGMRGLMSDPNGQIIDIPIVHNFREGLTITDYIMSSY
GARKGVVDTALKTADSGYLTRRLVDVAQDVIIREYDCSTSRSIKLIVGRTCGRNFRAF
AHDQQITKLMLDLLNNDFDIWIRSPLTCESMRSVCQLCYGWNLASGDLVNLGSAIGII
AAQSIGEPGTQLTMRTFHTGGIFTDPSRQIRAQESGLLRIPSDLKFRQGRTVYGTNIKI
LDQSTNLGLVHKTTVLLPPNSSLFLSSETFVKKGQLIAELPLTSQQIFAPNSGEVVPVE
LVWVLQGRVYEIPQNLLYFSSLNIDNFVTLKLTTRTSGMLEMFVFLSGLLLPEERYVIN
RDLLLVMSELIELIKGTFARIAGFLQIILQEITIKPGNLFYPGEVILSDILVESLSLIELIRPV
LDFYLFNLVSSDLNLSYLSLNVRNFQFVEKNSVLGKLSLRITQLETFALQDQVGKVLFT
TKSDYFTCYSERSLFDFLHLVKSGMPTKISPFSITLRRGTPVFLKEGMVLYKGQGSFIK
KDEALGLINFEQIITGDIVQGLPKIEEILEARKPQTSSRQGIKLGQPITVNPHLLLEVFF
NFYDAAYLSFKNLQLLLINKIQQVYNSQGVDIADKHLEIIVRQITSKVKITHPGATLLV
QGEIMELRQVNYINVIMAQNNYSPLLLGITKASLLTDSFISAASFQETTKILTAAAIEGK
VDWLRGLKENVIIGRLIPVGKGMFLTTLPDFVEIQRSSFCWFLLHGLREELENFSTISD
LRTIELNIFGEDYRIRKPSFSIAESKQRDGTYSVQIYVPMSIFVFNLNKRVNVLIGEIPLM
TDRGTFVINGCERVVINQILRSPGVYFIYTATVVPNRGSWLKFELNTILIYLDKTEKTSL
SDFLSALGMNDLRSSLTYPEFFQLLGFSRIFDPKIYDIGLVGRYKLNKTFNLNLPADLT
TLTNQDFTAILNKLICLNYKDDDIDHLQNRRVRSVGELLQIQFRSGLTRLERILSERM
TITLVSMMREFFGSSQLSQFLDQTNPLAELTHKRRISGLGPGGFSRDHVSFTVRDIHP
SHYGRVCPIETPEGQNAGLIASLASYARVNSFGFIETPFFQVKNGFVLKNYPAIYLTAD
EERQLKIAPADLVLNKIQQDNVTVRFQEELEIVPVTEVELMAVSAIQIVSAATALIPFLE
HDDANRALMGSNMQRQAVPLLYPTKPIVGTGLETQLAADTGMVVITYSDGVVNTV
TNSMILVRYALHKYMRSNQDTCVNQRPIVWEHEEVKSGQIIADGPGTDMGELALGQ
NILVAYMPWEGYNFEDALLVNERLVFADLFTSIHIEKYDLEVRETKVGLESSDIPNVLL
HLDENGIVKKGTFVTAGDILVGKVTPREEADEIPEGRLLRAIFMIDNSLRVPVLDIRVF
SRVFLAQMRKIQVGDKIAGRHGNKGIISRILPRQDMPFLPDGTAIDLILNPLGVPSRM

NVGQLFEGLFGLAGDCLNVRFKLQPFDEMYGPEASRTLINQTLKSASKSFPWLMNFN
APGKLNLCDGRTGQYFDNPLTVCKSYILKLVHLVDDKIHARSTGPYSLVTQQPLGGR
SQQGGQRFGEMEVWALEAFGAAYTLQELLTIKSDDMPGRNSALNAIPKPGIPESFKV
LILELQSLGLDIGTYQIHENFGIEIDLMMFDYIKINLASPTRIKEWGTRTLP-
NGVVGEVQKPETINYRTFKPEMDGLFCEKIFGPVRNWECHCGRYRVCERCNVEVTES
RVRRHRMGYINLIAPVTHLWFLKGIPSYLSLVLTNNSNGAEAIDYLLKNLDLGLEVQK
NRLALLLFDDTREKLVRRIRLLENFIATESHPSWMILKVIPVIPPGLRPMVQLDGGRFA
TSDLNELYRRVITRNNRLARLLKIYAPAIIVHNEKRMLQEAVDALIDNGKRTRKAHGA
NNRPLKSLSDIIEGKHGRFRQNLLGKRVDYSGRSVIIVGPDLKLNQCGLPHEVAIELFQ
PFIILIEKGAKLIQAGSSIWVILNVLSNHPVLLNRAPTLHRLGIQAFEPILVEGRAIKLHPL
VCSAFNADFDGDQMAIHVPLSSEAQQEARELMLAPNNFLSPATGDPILLPSQDMVLG
CYYLTVNNPKYKTQVPHYFINTSDVLVAYKNNLLDLHSPVLIRTHQFDFVQTTPGRIL
FNRALLKHSMSRYRGPKIRLIRKLGLPGLTTKTTTRFKKSSSEYGLRLQEKQKLRYNYG
VSEQQLLNYVKEARRLPGATGSLLLQLLEMRLDNIVFRLGFAPTIPAARQFVSHGSILV
NGRRVDIPSFQCQVNDVISVKDKPKACSVAKQNLVPSHLEVDVENLKGKVISVVVRD
EVNIPINELLIVEFYSRMGQKTHPLGFRLVTNQKPRSTWFANFKLYPNLIEEDTKIRKF
LATYIAKTEINRNNKIEIEIHCARPILVLYQIKSIIRTALINIVEIKYPDTEAALIGDFIVEQL
EKRVAFRRAIRKAIKRTVPGIKVQVSGRLNGAEIARSEWIREGRVPLQTLRADIDYAKKE
AYTIYGILGIKIWLFKMVNDTISDMLTRIRNATNAKHHLVQVPLTKMTKSIAEILKNEG
FIDDFEIIVEDSLVISLKYNGQKRKPVITKLQRVSKPGVRVYAGSRTLPVILNHLGIAIIST
SQGVMTDSQARKLKIGGEVLCYVWMWNERVIQISRVTKVCKGGKKLSFRAVIAIGNE
NGQVGVGIGKADDVINAITKATSSAKRRIIRVTLTKNATISHVATGIFGACKVLIKPAT
QGTGVIAGSSIRTVLELAGIRNILAKQLGSNNLLNNARATIVALESLMSRRNIAKKRYPK
PDPNFKSSLVSLLNARILKNGKKQLATKIIIETFAIVEEKTKTPALEVFEKAIKNTTPLVE
VKARRIGGSTYQVPLEVNRYRGTTLALRWLIKASVDRSGRTMSFKLANEIMDAANSIG
NAVRKREETHRMAEANKAFAHFR-
MIRIRLEAFDHRILNLACQQILDKIHTTQSKIVGPVTLPTKKRIYCVLRSPHVNKDARE
HFEIRVHKRILDVYPASEAAYSLINLPSGVTTTIMPTIQQLIRDPRKAVLKKTKSPALRD
CPQRRGVCTRVYTTTPKKPNSAIRKVARVRLTSGFEVTAYIPGIGHNLQEHSVVLLRG
GRVKDLPGVRYHIIRGALDSGGVKNRTQGRSKYGVKKPKMRKQKLNINIGVVHIQST
FNNTIVTVTDVSGNTISWGSAGSSGFKGARKGTPFAAQTASENACLTALNLGLKKVDI
LVNGQGSGRETAIRAIQNSGLEILSINDITSVPHNGCRPPKKRRVMVRIAGIDLPRTKRI
DYALTSIFGIGQNSAQIIVEKAKIDTIRVNELTDDDVSKIREIIEEFSVEGDLKRTTNLTI
KRLSEINCYRGRRHRQQLPVRGQRTRTNARSRRGSKKTKKMATKEKTGIVVSTSMEK
TIVIAVENRYAHPIYGKTIKKTQRFMAHDENSFCKLGDKVIIIETRPLSRKKRWTLKMA
KQSMLQREFKRQKLVRKYRAKREQIVYDLKTAKNLKDIFVLQRSLQKLPGNSLPIRLR

NRCWKTGRSRGFYRDFGLSRHVLREMAHDCLLPGVTKSSW-----------------------------
----------MVGHTIAVYNGKQHVPVLFQTKLLLHQL-DLFVLM-------------
MIKSAIKRIKIANRNNLQNKIYLSKIKTSTKNYFSVLSDLAIKSLSLLFSRIDKAVKRQVLH
KNTAARKKARLSTKLNQMTQIFTEDGSANPVTVIKVGPCVVTQIKTIETDGYNAIQIG
YQNTKHLTKPELGHLKKSQIQPLRYLKEYQIEENDFTLGQIINVQNFKEGDYVDISGK
SIGKGFSGNQKRHNFSRGPMTHGSKNHREPGSIGQGSTPGRVFPGKKMAGQLGGKT
TTVSKLEVLKIDSKENLLIVKGSVPGKPGNLLSIKMAKAKGTRLVITLECTECRSNNQR
KSGVSRYTTKKNRKNTPDRLELKKFCKYCNQHTSHKEIKMTQIFTEDGSANPVTVIKV
GPCVVTQIKTIETDGYNAIQIGYQNTKHLTKPELGHLKKSQIQPLRYLKEYQIEENDF
TLGQIINVQNFKEGDYVDISGKSIGKGFSGNQKRHNFSRGPMTHGSKNHREPGSIGQ
GSTPGRVFPGKKMAGQLGGKTTTVSKLEVLKIDSKENLLIVKGSVPGKPGNLLSIKM
QKFVTYNVLKSGQILKLTVLTFGNYLIHKDISRQQIQQKQGTVSTKTRGEVRGGGRK
PWKQKGTGRARAGSNRSPLWKGGGVIFGPKPRVKLLKLNQKERKLSLQTLLYSKRNN
ISVIDNLLEPKTKVFCDFCIDLNQNLLIIVSKKTNSLKLSTRNLKNVELISASNLNSLCLL
KAKQILLTPLAINDIKEIYMLKMRLKRCGRKRFPSYRLVIMPSSARRDGKAVKELGFYN
PITKEIKLDITTITSYLNSGVQPTRTVRNLLIKIIMLIKYPVISDKATRLLEKNKYTFLVD
RRANKTLVKTAIEYIFDVKVKKINSLNLPKQKRTKPQYKKVIVTLAEGTNINLFPMHVE
KKDLPMISVGDTVRLGVEIKEGEKIRIQAFEGVVIAKKNHGINKTITVRRVLQGIGVER
CFLVNSPKIKTIELIRSSKVRRSKLYYLRDLAGKATRLKQKF
>Rhizosolenia_fallax_MG755802
MIKKEYPKLKKIQINRGLGLAAQNTNILKKSIEEFTKITGQKPIITKSKKAIAGFKIREDM
ELGLTVTLRGEKMYTFLTKLIFFTFAQIRDFRGLSVRSFDKAGNYTFGLKEQLIFPEIDY
EDVVQVQGFTITLVIDSNSKKNGMVLLKFLRFPLMVRIKRGNIARKRRKKILQLAKGY
RGAHSRLFRVANQQVMKALRYSYVGRKQKKRTFRKLWITRINAASKLLSYSQVIHNFK
KSNIDLNRKILSQIAILDNPTFNKLVMSIRIYKSYTPGTRDRALSSFDEITKVKPEKKLIKS
NHRKKGRNNRGIITVRHRGGGHKKRYRSIDFRRNKHDIEATVAAIEYDPNRNARIALV
KYKDGEKRYILHPSNLNVGDSIMSGINSPFKNGNCLPLEQIPLGSSIHNVELSPNRGGQ
IVRAAGTSAKILAKEGNYVTLRLPSKEIRLIRKECFATIGEISFDDAFLVRSGKAGRTRW
LGKRPTVRGSVMNPCDHPHGGGEGKAPVGRTRPLTPWGKPALGIKTRKKKKSYILR
RMSRIGKLPIKIPTNVDITCNGSEISVKGKFGLLHNTLPNCLNIDGILIVDKTNRALHG
LYRTLINNMVIGVSEQFTIRMQLIGVGYRGVIQGKKLVLNLGYSHQVIMEIPDGILVEII
QNTKLELKGCNKEELGLFASKIRSWRPPEPYKGKGILYEGEHIVRKAGKSGKMTFIPIS
KSKKWYIVDADNKPLGRVATTIAKILQGKHKVDYHPSIDTGDYVIIINAQTMYLSWSP
KYRVYNPGKPGSSLKLVFEKVPKRIIESSVKNMLPSGRKTFYKRLKVYNDSQHSHEAQ
NPVLFMAKKIVALIKLALPAGKATPAPPVGPALGQHGVNIAAFCKEYNAKTGDKIGLII
PVEISVYEDRSYIFILKTPPASVLLAEAANIKKGSSTPNRINVGSITKAQLEEIANIKLPDL

NTNKINSAMKIVEGTARNMGISIMLSPKRTKYRKFHRGRMKGKAVRGNEISFGNFGL
QALEPTWITSRQIEAARRTITRYTKRGASLWIRIFPDKTVTARAAESRMGSGKGAVDY
WVAVVKPGTILFEIASVPEDIARAALNLAAYKLPIKTKFIVKMIYPQTVLTVADNTGAR
KIMCIRVLGGNRKYGRIGNTIIGVVKEALPNMPIKRSDIVRAVIVRTCKTIRRQDGMYIR
FEDNAAVIVNAENNPRGTRVFGPIAREIRDKNYSKIVSLAPEVLMYAIVEISGRQFWVE
TGKYYDLNRIPTGQEILLNRVLLFNEILIGKPYLDVIKGKVLQHLRGRKTLVYKMRPKK
KTRKKQGHRQELTRVLIMVKAVAKYIRMSPHKVRRILNQIRGRSYQEALMILEFLPYSA
SGPVWQAIYSAAANAQNNYNLDKKKLFIDEVYANEGPKLKRIRPRAQGRAFRILKPTC
HITVVVMSKPEIHPTWFKESPVFFDGKLICYVGSTKPKLTVDVWLQNHPFYTDSQTVI
DTEGRVERFMKKYGLMAHKKGAGSTKNGRDSNSKRLGVKKFGGHFVKAGNILVRQR
GMKFKPGINVGSGKDFTLFARVDGIVKFDVNII------------------------------------
MAVPKNRTSKAKSRTRKANWKIKANKKAQKALSLAKSVLQTTSFIYMIKCLKSEKEYG
RFLITGLRPGQGITIGNLLRRILLGDLVGTCITAVRFASFKHEFSTIKGVREDILEILLNL
KGIVLKSKFARLKIQGPAVLTADLIQLSPLVEIVNPNHYIATISTSNLLEIELKIEHGTSYK
LAFLQIDAVFMPVKKVDFKIENVSERLIIDVSTNGSLTPEEAISETLEFVVNFFNSLIPIEE
LKLSVRPYNCLKRAQINTIGDLLEYSPLQELFGRKSADEVFMTLKNQNVLISKKQLKQI
LSWSFSKYGSIKACFLADELKILGFKYATYAGISISIEDLRVPYIKQTMLENANQEILNTE
KIYLKGKITNVERFQKIIDTWNITSEMLKDEVVSFFKKYDPLNSVYIMAFSGARGNLSQ
VRQLVGMRGLMSDSNGEIMNLPIKKNFREGLTVTDYLMSGYGARKGIVDTALKTAN
SGYLTRRLIDVAQDIIIREKDCLTQHSFIFILGRLLNKPIYISEINTQITPNLIRTFKRIKKV
YIRSPLTCSLYRSICQKCYGWDLASENLVDMGEAIGIIAGQSIGEPGTQLTMRTFHTG
GIFTETNQQLISSKNGFIIFSDVLKTSPLRTNRGENVLVTKSSGSLSIIDIIQLELPRNTIL
FVKNKQYVKQNSLIGQLAGNIKQILSNSSGEVFITQLLWILSGQLYKAPNNFYPDYKIN
QSYIFRSKLVNQYSGFINNYLLKGGLILAEETHNLICKLLLINSLVFELVPNVYSQTSGIV
KIIIQEISIKPGNVFYPGEIILNTIEIIQPVLCELIRPIDLYEITNLIRNSFDFIKTCLIVELNQ
YVSPHTVLGYLEIINQALELVKLKSKNKQIFIISNNDCVTIEKNKNKTDLLNINQIGKIII
DNGKILTIQKGYPYLFQEVNNVFCKNGEFLESGQIIGTLNFEKEITGDIVQGLPRIEELL
EARKKKKNQKKIRKIGTTVKINPHSLLKIYFNYYEASYRSFKKIQVSILNSVQSVYESQGV
VIADKHLEIIIKQMTTKVLITHEGETPLLPREVIDLYHIKYINEIIRIQYYVPLLLGITKAA
LNNPSFISAASFQETTRVLTKAAIEGRLDWLRGLKENIIIGHLIPAGIGMYITALPDFIEM
QRISFCWFISEGLKDELTNFSSILDFGNIEYIFFGQEYKLVKPFYNSLNAKKHSTNYIAQL
IMPIEMRNRQIIRRGRLSIANLPLMTNSATFIINGCERIIVSQVIRSPGVYFLYTATLIPEY
GSWIRFGFLRIVVQIDKIIKKPITHLLREMGLKEISKNLKHSDYFYFSEFSRIFDSRYYRLG
KIGRYKLNQRLNLKISKEFSSITYDDIFGIIDYLITLAITKDDIDHLKNRRVRSIGELIQQL
FRVGFQRLSRKILSQVYKSIITTVREFFSSSQLSQYMDQTNPLASLTHKRRISGLGPGGF
DRDRISFSVRDIHPSHYGRICPIETPEGKNVGLISSLTTCARVNESGFLETPFWRIINGK

VIKTENPIYLTADVEDFYKIAPADISTNYLTKSSIPVRYKQDFITVNPSEIDFIAISTVQVV
SVAASLIPFFEHDDANRALMGSNMQRQSVPLMIPKRPIIGTGLENQIAADSGFTINTIK
SGVVDFVSSKKIIVIYHLQKYQRSNQETCINQRPIVWKGERVRSGQIIADGPSMNGGEL
ALGQNILVAYMPWHGYNFEDAILINERLVYDDVFTSIHIERYEIKVNLTNENRETNNIP
NLTQNLNENGIISIGTFVKSGDILLGKVTPKDDSDQVPEAKLLRAIFVRDSSFRMPVLR
IIIFRPVFIAQIRKIQVGDKIAGRHGNKGIISRILPRQDMPFLPDGTPIDIILNPLGVPSRM
NVGQLYECLLGLAGNKLNRRFKILPFDELYGEEVSRILINKKLREASKNEAWLFNPYSP
GKIVLIDGRTGKEFENPITVGNAYMLKLIHLVDDKIHARATGPYSLVTQQPLGGKAK
HGGQRFGEMEVWALEGFGAAYTLKELLTIKSDDMQGRNDTLDAIPTSGIPESFKVLL
QELRSIGLDISTYQLEKFSEIEVNLMMFDYIKIKLASPTRIKQWGQRLLP-
NGIVGEVQKSETINYRTFKPEMDGLFCERIFGPNKNLECACGKYKICDRCGVELTESR
VRRHRMGHINLIYPVTHIWYLNSRPNYMALLLIKRIKGSVIIKKELEKLNLSHEINESRN
FINYCSKLRDQAIKRIRILENLLATGSNPAWMILTVLPVLPPALRPMIQLEGGRFATSD
LNELYRRIIIRNNRLLKLLQIDAPQLIIRNEKRMLQEAVDTLIDNGKRGKVALDGNNRP
LKSLSDIIKGKHGRFRQNLLGKRVDYSGRSVIVVGPNLKLNQCGLPYQMAIELFQPFII
LINQGAKLIQRNQLIDPVLKVLTNHPIFLNRAPTLHRLGIQAFEPILVQGRAIKLHPLV
CSAFNADFDGDQMAVHVPLSFEAQAECYMLMLAPYNFLSPANGSPIITPSQDMVLGC
YYLTVNSIKGLLGSTHYFLNLEDVILAYNQEQIELHSLIWVRYIIVKYLQTTTGRALFNY
TVQKTLMSRYLGPRLRITRRLGLPGLTNKQSKKKNSKKTQYGLRLEEKQKLKFNYGIT
ENQLFSYVKEARRRKGVTGLILLQLLEMRLDTICFRLGFAPTMASARQLVNHGHITIN
GEVLNIPSFQCRINDIVSIKQKTSSKNLVEENLVALHLSFDKEKNEAKVLNYCSRDDILL
ELDELLVIEYYSRMGQKTHPLGFRLGIVQEHKSSWYTKLNQYSNLLEEDDKIRTYLNK
LISNIFINRNDQIELNIETGRPVLVLLKIKKMLRKITINILEVEKVNLNASLLGNFVVKQL
ETRVAFRRAIREAMQYANNGIKIQVSGRLNGAEIARSEWIREGRVPLQTLRANIDYATK
EANTIYGILGIKVWLFKMVTDTISDMLTRIRNANLLKHQIVQIPTTKMSLAIASILKEEG
FIEDIKKYPNRYFIILLKYKGKSREPVIQKIKRVSKPGLRVYSGINNLPQVLGNLGIAIIST
SKGVMSNFTAKELGIGGEILCYIWMFLERLIKISRVSKVTKGGKKLSFRSIVVIGDENGV
VGVGVAKANDVINAFKKAKTDARKNLITVPITKSLSIPHNVIGDFGACKIIMRPSIEGSG
VIAGGAVRTVLEVAGVKNVIAKQLGSDNLLNNARASIVALSNLMSRRNISKKRFPEVD
PKYNSYLVSLLIQRILKSGKKTIAKNIVNGAFEIIKTKTNEDPLVIFEKAIKNASPIVEVKA
RRIGGSTYQVPIEVSSFRATNLALRWVIKNSRSRVGRRMSIKLANEIIDTANDIGNTIKK
KEETHRMAEANKAFAHFRRMIRVRLESFNHELLQISCRKILDFTQNIDLDNVGIISLPT
NKRIYCVLRSPHVDKDSREHFELRIHKKIIEIYYESDIFNLLLDLPSGVLYSIMPTIQQLV
RSRRIQIKKKTKSPALVDCPQRRGVCTRVYTTTPKKPNSAIRKVARVRLTSGFEVTAYI
PGIGHNLQEHSVVLIRGGRVKDLPGVRYHIVRGALDSGGVKDRTQRRSKYGVKKPKM
KKSKNINTSGIVHIQSTFNNTIVTITNLAGDTISWASAGSTGFNGTRKSTPYAAQTAAK

KASLDAMNFGLKSVEIEVKGQGSGRETAIKAVQAAGFEINAIQDITPISHNGCRPPKRR
RVMIRLIGIDLPRNKRIEYALTYIHGIGLTSAKTIVKNAKISTTRTDNLTNDETIAIRNEL
EDLKLEGDLRRFNGLNIKRLNEISCHRGKRHRNNLPVRGQRTRTNARTRKGTKKTKK
MPVKEKIGIVISNKMEKTAVVKVENRYPHPIYNKTVVKTRKYLVHDEYNKCNIGDQILI
QEYRPLSRRKRWLAMMAKKSMIEREKKRILLNKKYANKRSLLLNEYKATENFNVKLEI
HSKIQKLPRNSAKIRIRNRCWKTGRPRGYYRDFGVSRHVLREMAHQCLLPGVTKSSW
MSRSLKKGPFIAYHLLKKIDKLKKSTIITWSRSSTILPDMVGHTIAVYNGKQHVPIFISD
QLVGHKLGEFVSTRTFRSHKTDKKSKRMNKSALKRIRIGERNRLQNRFYKSNVRKLTK
LFYERLEFYVYAIVNTLYSIIDKGYKKNVFHINTASRKKSTIDSELKKMTQIFDESGKIIP
VTVLKIGPCIITQVKTVLKDGYNAIQIGYGNRKSLTQPELGHLQKSNIQPLKYLKEFKI
NNEDFQINQVLNVETFTTGQLVDVRGKSSGKGFSGVQKRHNFARGPMTHGSKNHR
APGSIGMGTTPGRVLPGKKMAGQLGNKIINIKKLKIILIDSKENILVVKGSVPGKPGNL
LSII--
MTQIFDESGKIIPVTVLKIGPCIITQVKTVLKDGYNAIQIGYGNRKSLTQPELGHLQKS
NIQPLKYLKEFKINNEDFQINQVLNVETFTTGQLVDVRGKSSGKGFSGVQKRHNFAR
GPMTHGSKNHRAPGSIGMGTTPGRVLPGKKMAGQLGNKIINIKKLKIILIDSKENILV
VKGSVPGKPGNLLSIIMKQTLNYKVHTSETSITLSVQTNPKYLLHRAIVAQNNGLRQG
TSSCKTRSEVRGGGKKPWKQKGTGNARSGSSNSPLWKGGGVAFGPQPRSYAKKMNT
KEWRLALNTALQSRFDKTVVVEDFSNPKTKNAVEVLADNSSKTLIILSDVNDNVNLSL
RNLSNVTLLRSNTLNIRDILLANKIIITDKALQNIQEVYMLKLRLKRIGRKRQPSYRLVI
MPSERRRNGRAIEQVGYYSPITKEVHFKVEKIIYWLKVGAQPTQTVANLLKKIIMNIKY
PLITDKATRLLENNQYSFIVDPKSDKITIKSAIEYLFDVKVIKVNTCHLPKKKKRKSHYK
KAIVILSEGDTINLFEMTIKKKEIPNIQIGDSVKIGVKIIEGERERIQFYEGTVIAKKNSSIN
TTITVRKVLQGIGVERIFLIHSPKVDSISILRSSKIRRAKLYYLRNLRGKASRLKQKF
>Sargassum_confusum_MG459429
MLKFLYPKLVKIQINRGLGGDGQNNKILQKSIEEIRIITGQHPIITKAKNSIAGFKVREE
MILGVTVTLRNKKMYAFLEKLIHLVLPRIRDFRGLSLKGFDRNGNYNFGLKEQLVFPE
INYENIDQIRGLNISIVTTAKTKSEGVALLKEFGFPLMVRVKRGNVARKRRKKILKLAK
GFRGSQSRLFRIANQKVMKSLVYAYIGRKEKKRIFRKLWIARINSSARGINYSHFIYKLK
QSKILLNRKMLSQLAILDPSAFSNLIMTIRICKVYTIGTRNTIFPAFDDITKSQPERNLIV
KNHRKKGRNNKGRITIRHHGGGHKRRYRTIDFKRRKHDRVGYVYSIEYDPNRNARIA
LVHYDNGEKNYIICPDSLKIGSKILSGPDAPIEIGNALPLENIPLGTSVHNIELSFNKGG
QMVRAAGTAARILAKENNYVTLRLPSKEIRLIHKSCYATIGTVSNKDHNNIKLGKAGR
KRWLGIRPTVRGSVMNPCDHPHGGGEGRATIGRPKAYTPWGKSTLGVKTRKKAKSY
IVRSMSRIGKLPIKVPSNVQVEINKQMIHISGPHGKLFRKISDSILVQNLITITKIANQLY
GLTRTLINNMVIGVSQKFTRTLLLQGVGYRAQVSDTNLILNLGYSHPILIPIPVGIDIKI

EKNIVIIITGFDKELVGQLGATIRSKRPPEPYKGKGILYKNEIIKRKIGKSGKMTFIPSKQ
KKQWYIIDAKNKCLGRLATEISILLRGKKKVIFTPTQNLGDSIIIINAEKIFVKRTQKLYF
RHSGRPGETFEDLQMRIPERILEKAIKGMLPKNGRKFFKNLRVYKGSKHPHVSQKPQL
IMGKKVTSIIKLALSAGKAVPAPPVGPALSQHGVNIAAFCKEYNARTADQSGLIIPVEIS
VYEDRSYTFILKTPPASVLLIKALNIKKGAAEPNKQIVGSIEKSEIVKIAQIKLSDLNTKNI
DSAFKIIAGTAKNMGITIMLSPKRTKFRKQHRGKLKGKVFNRSTITFGDYAIQALEPT
WLTSRQIEATRRTITRYTKRGGKLWITIFPDKPITARAAESRMGSGKGAVDYWVAVIK
PGTILFELSGVPLKLAKDALQIASYKLPIKTKFLSRMIQPQTYLTVADNTGAKKIMCIR
VLGGRRKYATIGDIIVGVVKEATPNMLTKRSDIVKAVVIRTKKSVSRRDGTSISFDDNA
AVIINTDKNPKGTRIFGPIAREIRDKDFTKIVSLAPEVIMYAIIEASGRQFWVEPNKFIEL
NRLPLGSTILIKRILFVKNTFIGQPYLTILKGIISKHFLGSKILVFKMKPKKKYRKKIGHR
QKLTRLLIMAKAISKYIRISSTKVRRVLDQIRGRSYLEALMLLQFMPYRACGPIWQVLN
SAAANAEHNNGLSKEELKVKIAFADQGPSLRRFRPRAQGRGYQIRKPTCHITIILMVKK
KLHPEWFENTKVYYDGQLIMIVGSTKPELHVDIWSGNHPFYTGSQRIIDTEGRVERFL
KKYKIMAHKKGAGSTKNGRDSKSKRLGVKCFHGHQVRAGNILIRQRGLKFKPGQNV
GIGRDYTLYALKEGLVFFKISVFMKVRPSVKKMCENCRIIRRHGRVQVICVNLKHKQR
QGMAVPKKRRSKSKGKIKLAIWKGKGRKMANRALSLAKSILESK-
FIFMCKCVDLDLNYGHFVFTALEKSEGTTLGNVLRRTLLSNLYGFRIVGVRIAGIKSEF
ASLEGVREDLFEIILNLKELVILNYYGRLKAYGPAIITAASLELPNNIKILNSSNHLLTISD
KSLIELEIKIEAGKSYVLAFIPIDSSFAPISKVNWYLKSNNEELHLEIYTNGTLLPHQALL
QARTVIGYMLSTINSLYSLGLSTRIIKALKKVNINFTQDLIQYPLLLGILGIKSVEEIKEKL
NKSNNIINKKELKKIIEWAFNNYGQRKAAYFVDQLKEMGFKYATKSGISISIEDLRIPAS
KVTLMQSATKEVFFTESRANNGAITEVERFQKIISIWNRTSEELKERLIDFLKKNDPLNS
VYVMAFSGARGNISQVRQLIGMRGLMSDPNGQIIDRAITANFREGLSITDYIISSYGAR
KGLVDTAIKTADSGYLTRRLVEVAQSIIISQLDCQTKRGIVLLYGRILASSICIGFRNQLV
TSSLIEQIIFIPKILIRSPLTCECRRSICQHCYGENLASGNLVELGETVGLIAAQSIGEPGT
QLTMRTFHTGGVFTQLTRQGRAECSGYVIFLPTLKVIPYRTSYGEDVVMSENQSSLKI
IKDIKVKVESRTLILVNNHNYIKRNQVLFEAAPKIKDIYVKEGGEIILKKIFWVLSGRVFS
LSNKARKLEKIYKQALAQSKIITTIGGFILHCKIYGGIFIPEATIQTNTNDFRFGSYIQEIF
PNYFATIAGFLKLQIKCVTIKPGEVYFPGELVGNQFSIKVLSYLEYIRPIIRYEFIQILDSTI
TIIFLNLIVEDNQFTDPYSILASFDTLIFNNFVYTIKTKKSNMLLTTKSDYQEIFLDTAQ
LSIFCIKNSGLLKSVIGNKITLHLGEPYFFSKGALIRKLPGDYIKKQENFGQLIYERLKTG
DIVQGLPKIADILEARRPKYTDELITKIGQPLTLNPHTLLHIYFHYFESAYRSLKKLQIFL
LMSVQDIYMSQGVIISGKHVELIVREMTHKVYIEYPGKTTFLPGDIIDLDQAQYINNSL
KTRGFRPILLGITKSSLKTDGFLAAASFQETTRVLTHAALQGKTDWLRGLKENAITGR
LIPAGTGMFAMRLPDLSEVQRVSFCWFLTEGIAEELINYPSIMNKSGIELLIYGQEYKIH

YPNFNILSALQKRGNFNLRIYVLMSLKKKEFSKKERVFFGEIPLMTEKGTFIFNGCERVI
VSQIIRSPGLYYFYEGTIISKYGSWLTFELEYIWVKVDKQYKIPINWFLVGFFLEEIYQTV
QNYEFLSKRELEKILNKNFYDLGEVGRIKLNKKLAINLPINIRIITSLDILKVIDKLISISFY
NDDIDNLENRRVRSVGELLQLQIRVALNRLKKNIIASEKLSILASIREFFGLSPLSQYFDE
INALAQLTHKRRISSLGPGGLNSEHVSFAARDIHPTQYGRLCPIETPEGQRAGLISSLAT
HAQINKYGFITTPFFRVYKGKVLKDSSPIYLTAEKEKMYKVASADVLLYFFQDTSVPVR
FYNEFIIVEGTNVDFIAVSPIQIIAAGASLIPFLEHNDANRALMGSNMQRQAVPLLYPKK
PIIGTGIEHQIAIDSLVVIINLLNGIVDSVSSDQIIILYFLDKYRRSNQETIINQKPLVWPG
EIVKPGQTIADGPGTDGGELALGQNLMVAYMPWEGYNYEDAILVSERLLYNDLFTSI
HIERYELQLMDNSMKIETKSVPNIILNLDINGIIKKGTFVKGGDILVGKITPIMEEEELP
ESKLLRAIFPEDTSLRVPVIEVQTASRILIAQTKKIKIGDKLSGRHGNKGVISKLIPTYDM
PFLPDGTTIDVILNPLGVPSRMNVGQIFECLLGFAGDYLNHRFKVLPFDEMYRKNASR
IFINKTLKSAATNKPWIFNKSFPGKVILKDGRTGEIFNNPVLVGKPYILKLIHLVDKKM
HARSTGSYSLITQQPLGGKSKHGGQRFGEMEVWALEAFGVAYTLQELLTIKSDDING
RHEVFNAIPEPSIPESFKVLLRELNALGLDITTHQLRKSSSFNVNLLMFEYIRINLASPQR
IKEWSQKTLP-
TGVIGEVTEPETINYRTHKPEKGGLFCEKIFGPVKNWECACGRYKICEICGVELTESRV
RRHRMGYIKLLTPVVHIWYLKGAPSFLASILILKYEGAEILYDKLKNIDLKIEIETNRNIM
FCSTKVVEAAIKRIRIFENFLSTNTRPEWMILQFLPVLPPGIRPMVKLENGRFATSDLNE
LYRKIIIRNKRLKRLLSVHAPSIVIITEKRMIQESVDTLIDNGKRGSKVVDANRRPLKSLA
DIIEGKQGRFRQNLLGKRVDYSGRSVIIVGPKLQLNQCGLPYEMAIELFLPFIILLSQGA
KIIHSNPFIWYLTEILSKHPIILNRAPTLHRLGVQAFDPVLVKGRAIQLHPLVCPAFNAD
FDGDQMAVHVPLSFESQLETRLCLLAPNNFLSPATGEPNIQPSQDMILGFYYLTTHN
RLGLKGANNYFSSFYEVLSAYQHKQLKVHSPIWVRCLLVQYIRTTPGRIIFNQCLNDIL
MVRYRGPRLKIIKKLGLPGLTRKIIIKKKKSKSAYKIRLNEKQKLRYNYGITESQLLTYVK
EARRRKGLTGFLLMQLLEMRLDNIIFRLGLVPTIPAGRQFVSHGHVLVNRKKVNIPSF
QCRPNDIISFSSKSKITNLLKTNLSTSHLKFDEQSLTATINRLVTQKDLSFKINDMLVIE
YYSRMGHKTHPLGFRLGGLQDDRSLWYSGVNTYISFLKNDYQIREYIYSFITKIIIRNDK
KVYVEIQSVVPRIILDKLQLLIRKVLINIVEIEQPYQEAGILVDILVKKLEERVVYRKCVRE
ILQRYTKGIKIQIAGRLNGAEIARIEWVREGRVPLQTLRADIDYSYKTAQTTYGILGIKL
WLFKMTTDRIADMLTRIRNANLVRHQIVRVIKTKIIFSLTKILKEEGYISSFEEINKKYLL
LCLKYHNQKREPIITGIKRISKPGLRVYVNKSNLPNILNNLGIAILSTSKGIITNHKAKKL
GIGGEVICYIWMWEQRVVKVSRVSKVVKGGKKISFRATVVIGDMEQKVGVGVGKAKE
VSTAVKKAETDAKKNIINIPIAEGKTIPHSMIGVAGGSKIFIRPAVQGTGVIAGGSVRIVL
ELAGIKNILSKQLGSNNPLNNARATLMALKDLMSRRTNKKRKFPNEDPVYNSFLVSL
MISKILKNGKKTIAEKILYEAFDIIKQKTDTQPLKIFEIAIKNVSPTVKIKAKRIGGSTYQV

PIEVKKFRGINLGLGWILQFAKARSGKTIAIKLANEIIDASKGYGNAIRKRHETHRMARS
NKAFAHFRRMVRIILQSFSDLRLYRSCDVILNVLSQEKTNISGPVSFPTKKRSYCVLRSP
HVNKDSREQFEIRRYKKMIDIESNSAIINTLLDLFPGVSTKLMPTIQQLIRLKRRKIQPR
TKSPALKSCPQRRGVCTRVHTITPKKPNSAIRKVARVRLTSGFEVTAYIPGIGHNLQEH
SVVLLRGGRVKDLPGVRYHIIRGTLDTTGVKDRRQGRSKYGMKKPIMKKIKRTIVTG
AMHVHATFNNTIVTVSDLDGNTLSWASAGTVNFKGSRKSTPFASQKAAEKAALIAKE
YGLKRLEISIKGSGPGREAAIRAVYQKGIKIVSIKDNTSIPYNGCRPPKRRRVMVRLLGR
DLANNKKIKYALTTIYGIGLSRSKEILEIAGLHGIRVKDLSDEEISSLRKVLENYQLEGDL
LRLTNLNIKRLMENGSIKGRRHRAGLPVRGQRTRTNARTRRGGKKTKKMVKLQRLGI
VISDRMQKSVVVAVEYRYKHQFYSKIVVRTKRYLAHDEENNCNIGDEVIVEESRPLSKR
KRWIVKMAKKSLIQRERKREKLVSRYMERRKFLLLERKRTTNFVQQTKINRKIQKLPR
NSSKIRLRNRCWRTGRTRGVYRDFGLCRHMIREMALNCILPGVKKASWMARSFRKGP
FIAYHLLQKIEKMKKNMIKTWSRSSIIIPSMIGHTIAVYNGKQHIPLFISEQIIGHKLGEF
VPTRNFRSHKSDRRLKRMNKAAKKRIKINKRNNIRNNSYNSLMKTSKKKFINNVEIYVK
ESLILAIRQIDRAAKKKIIHKNTAARKKSNLYKKISIMTQVFNKDGLALPVTVIRVGNCFI
TQLKTESINGYNAIQIGYLKQIRINKAELGHLTKNGLPPLLHLQEYKVPDNDYSLGQII
NVNYFKIGQLVNVSGKSIGKGFSGNQKRHKFKRGPMTHGSKNHRAPGSIGPGTTPGR
VLPGKKMAGQLGARRITVSKVRILGIDEKQNLLILKGSIPGKSGNLLSISMAKNKGTRII
ITLRCTTCKSIKRLSTKKLNYTTTKNRRNTPDRLELKKFCPNCNNHTIHKEIKMTQVF
NKDGLALPVTVIRVGNCFITQLKTESINGYNAIQIGYLKQIRINKAELGHLTKNGLPPL
LHLQEYKVPDNDYSLGQIINVNYFKIGQLVNVSGKSIGKGFSGNQKRHKFKRGPMTH
GSKNHRAPGSIGPGTTPGRVLPGKKMAGQLGARRITVSKVRILGIDEKQNLLILKGSIP
GKSGNLLSISMKKILTFTIKLKGDTLKVKELDTNNYVIQRAYLTQRLNERQGTGNTLT
RAEVRGGGRKPWRQKGMGRARAGSNTSPLWKGGGVSFGPKPKLYLNKINRKEWKL
GLRNLLVAKEKNITVIENIITYKTKNIIKILINLAKDTLIILPTIQKELLQSTSNIKTIKLTL
ANNLNLKQILLAKNLLVIKDSLKIIEDTYMLKIRFKRSGRKKQPFYKIVVMDVRTKRDG
KALEELGFYNPITKTLNINCERTIIRLKNGVQPTEVVKNLLRKIFMLIKYPVTTPKTLLL
LENNQYTFMVDPTLNKFNIKKSIEFLFNVKVIKVNSCNLPKKKRRRPRYKKVVVKLAN
NDKIELFSMLVEKKSVPKVFVGDTVKIGVLIKEGNKERIQYYQGIILAKTNSGINLTISV
RKIFQGIGIERKFLIHSPKFESLQIIKSARVRRSKLYYLRKTTTKGSRLKQRF
>Spumella_sp._NIES-1846_AP019363
MI---
YTKLLKINISTTFKSYTQNLEYIKEAIQEIYLITGQVPYLTKPKKNISNFNLSKNKGIGAT
VNLNKKNKYIFLEKLITLILPKIKDFNGLSYTQFDKYGNFHFRLTSQYIFPEISILTTKYIK
GFNIIFNLKNKNLATSKIFLNLLNFPLMIRITHGYTTKKYKKKILKLTKGYRGTASNLST
YAIEQLTQSYNYAYIGRKLKKRYFKTLWISRINITTKYIQYSFFLNILKKNNIFLNRKLLA

YLAYNDLPSFKYLTMLLKIFKPLTPSLRHTKLLKNLNLAINNPEKSLLKKYIPKTGRNN
QGIITSRHKTGGHKKRYRIIDFTFKNYNIPGIVINLEYDPLRTANIALIHYIDGTKGYILA
PEFLMKKMILFKS----
YNVGTSFFLFQIPVGSEIYNIEFIPGNGGKIARAAGTSAKLLLKEKNHVIILLNSTELRIFS
NKCKAILGKVSNSQYKNINYGKAGRKHWLGIRSKVRGSAMNAVDHPHGGGEGKSSIG
LIQPRTPWSKIALGMKTRKKNKSFILKSMTLITK--
LSIPQNINLQHFKNIIYFKGPYGLKKLIYLNYVLILNTLIIQKKAKAYYGMLSKIIQNIFYG
LLYTYTQTLFLKGIGYKFIKEDKKLFIKLGYSHLLSLKIPKAITINIETQTKLIGLCIDKNV
LSLFINRIRQFKLPNKYTGQGVLYPNEIIKLKNGKAHKMTIILKNEKHKIYTINATGYFL
GRLITIISQLVKGKILNLYTNTLLPKIYIIVYNIKHILIKSFQKLYYFSSQRPGKTYNQLFL
YSPYKILKYSILKMLTKNKKKFLNYIYLYSKNL-
SKIFINHKNIMIKKIKANIKLVLTGGKATPTPPIGPALGQHGINIMNFCKDYNNQTSMF
IGVIIPVNITIYEDKTYTLILKSSPTSNLLKKALNVLKGSAIPNKTFIGTITFDQLFEIANF
KLKDLNTKNIQKAISIIQGTAKSLGIKIM--
PTHTTLVKKHLASLHGKSQANNCFPQSLYAVESLDRFYISKKMLEYLTALFKKNLKKF
ATVYCMVHPYLAVTARAKEARMGSGKGSICNWVMPVKPHRILWTIKTIIKDIVKKLIK
TIKYKLVAKLNLLCHMIYIQTLLNVADNSGAQKVLCINILGHNRKFGLIGDNIRVSIKK
TKPTKNFKKGEVMDATIVRTKKVITRNNGFSFSFFENVIIIVNKNFVPSFTRILGTIPKE
LKNSRCTKLLSLADNLIMYIFLFLNGKQFIIEKNKWYDIDYIKSNTYIYLTKILFFKKFILG
NPFIKSLLAKILYTYNSKKIIVLKTKPKKHYTKIFGHKQKFTRIFFMFIYTIFNIKQSQKK
NLKFIKKFKNLSIINFLNKLKLLNLKLSIILQKNIYFIIYLINKNFQIKKEHLWLSNIYINKSS
KLKRSHIRAKGEIVKVEKHFSHLTFNL---

MAHKVGTGSTRNNRDSNSKRLGIKCIGYQKIKKGNIIVRQRGTKYKPGLNIGIGKDYT
LFALKTGILIYYIII--

MISCIYNKIKQSYFIIQPINIGHGITLGNLFRRILLSEMIGYGITKLKINNINSEFQKIFYIRE
DILEIILNLKEIKFKSTKGNLLQKGPIIITAGMFKLSKNIKILNPYQYICTLITNKYLNINL
EINCGYGYKIAYIDLDTIYTPVKKIMYNVRIIKESLFLDILTNGTITPTRCLFESFLYLVN
TFYSL---
SSNPYNKQLLENLILTSFIKFGNISSAKLLNSLKNLGYYYATQGGISLSFQDFLLSHIKTC
LQNTLHDHEKNINIKIIENKYDLIESYEATLDLWNEITNLLKVKIIDYYQNYTPLNNLFI
MCTSGARGNLAQLVQLLGFRGLMSDADGNIIDVPVVNSFYSGLSLLDYIICSYGSRKG
VVDTALKTADSGYLTRRLVYVLHDLILTEVDCQTQNGILIFIGRTLV----
LIYYNKPLTYKLFFYLRNITILKFRSSLTCTIYNNICQTCYGWELNNFRKITIGSNIGVIA
AQAIGEPGTQLTMRTFHTGGTYEFEIELIRAPETGKLILP--------

KYSTFSNNILTSSKSTIYLIKILALKCPKNMTYDYPLISYINKNDIIGYYPVITTSILTPMSG
KITYYNYFWLKAGRIITIPNKYNSFTKLFNKPFAYLNLITPYSGILNTLEF------------------
-------KLQQNIF--
LNLYKLLFFSKNYQFFDAYSLLGYLTIYTYTGSLYFASKKFSKLYLVTELDTYKVYSDPI
Y------
NIYNTYIKKEGLTYIYQYLFPVILPNNTLINFYNSDFFEENCCIGVLAHKSNAAADIVQG
LPKLEDLLNGVKSYDTNLVKYILGEPYTISLEKLLLTFYYYHKGLKISLLKFNIIFLNTFL
GIYDAQNIFVHYTYFEILIKYLISYGEIFYCGNTNFYYMELIDLKYIYELFKVYNSSLIIPRN
FSILTINSLKSGFLTISSYQNTRFLLVDKTLRGVTDWITGLKEHLITGQLLHTGSTM----
----
---SLLLK---

YTNCFIQLFYNTEIAQFGDQVNILADAYQKRKLTYFGDYGLPDDHITLDIRNIHYTYY
GRLCPVETSEGGDSGLVSSLALYTRVNHASSLMTPYYLISNNIFLNKNSIIYLDLLQESS
LYVNYLDMIIIVLTNHFSMIRFSELFYNIITAETKFISVSPSSLLSLSVALIPFLEHNDGTR
TLMGANMQRQALPLLYAKKAIIGT--
QAEIYINSDYIIISYSQGEVIYSSSNIIKIKYLLHSYKFTAQYTVKNQIPIVWLGEKIYAGQI
LADGTGTHQGEYALGRTLNVAYMPWDGYNYEDSILISEKLIYNNILTSIYIDILDINLN
KSLLLSDTANIKIYWLNLDFNLFITFQTFIKDIEILLNKIVIKNNSIILNQNQLFEKIFFKY
QSINFPVINIMNFYNLYIGTLKKIQVGDKLSGRHGNKGIISRIVNIQDMPYLSDGTPMD
IILNPLGIPSRMNLGQLYEGLLGIIAEKLGKRYKISSFDENSNDMNSSRLIYMKLKEIFYH
FN-
LFQTFCYEKFLLIDGRTGEFFDNPIFIGKSYIMKLNHLIDKKIHTRETGPYQFITQQPTV
GKLNDGGQRFGEMEMWALESYGCSHTLQELLYSKSDDMLSREFFTLSFGISHLTDTF
LNLIIHFKSLGLDLSFKKINSFLEL-VSLLMIDYVQIYLASPLKIKLWAIRYTK-N-
IIGEIKTPQILDIITQTPSLFGLFCPKIFGPLENYKCLCKSYTICDFCGTELNYTLQRRFR
MGFIDLTYPAIHSWYLYGNPNYLRMFLIKSLYGNESILSGLDNLNLLFEIFKKRNFYEK
NVIPAKELLRTIRIFESFYSNNISPKWMLFTALPVLPPAIRPYLDQTSNEIETNPLNLYYSI
ILKKKKSLTYFSQKLSPKLIIQTAKYSLQKYVEALIDNARLHVEGIDDTLQPTQCLTGFL
EGKAGKFRNLVLGKRIDYSGRSVITVGPNLGFNDCGIPYKILITIFKPLLYLINNEEKILH
TQSIVLNLLHILQYSTVILNRAPTLHRYNIQAFQPKLTLTKTIILNPLVCSGFNADFDG
DQMALYLPCYNLSQKEINLLMKPLYNIISISSNKLVYRPTQEMIMGCYYLTLIIKDFPNII
KNWFNSESLALLAYMQKRISLYTPILIKYILLTFLETTVGHIIFNLNYFNSLMSKYLGPKI
RLIRKYGLPSLTVRSSKNRQTTIDEYKEHLIEKQKLRFNYCLTDNDIQHYYYKLKTLKN
SQK-
RFLNYIEFRLDCLLYRLGFAKTILQARQYINHNHILVNNKIINLPNYICNLYDIISINKLK

NSKKVVYQNILPSYLEIESANLYGCIINYFNYKEALVHIDELKILQLYSLMGQKTNPLLF
RQIINSNYYSNWYTNKNNYINFLKHDYIIRKKLETFLSDILIETSKIITFELYYYNPSNIIN
VITKKVTTLTFNIKLINTIFSNLTLIAKYMALQLPKKINIKRLINYIFDNVEQGFKILINGR
INGEQMARNETRTDGRIPLHSLKYEIKYKNYYALTIFGILGFELWLF-
MSPFLISQLFTHINNACKIYLKHCFSPYSKTNFAILNILKKTGIIKDYNISNSLVLNIYLLY
FYDTIISSFQILKVISKPQQRIYCSYKNFPTLRYNQSFLILSTSLGIMTHNTAQSLKIGGEI
LGYVEMDFQKILEVKRVTKVVQGGKRLIFRIITVIGDTINKVGLGIGRSTLYNRALQNSI
THAQSNLFIVPKTYNNSISIICKQTYGNTLLYFHPAPNNTGVIAGSCVKIILELAGFKNIY
VKQHGSNNILNNARATILALKKLMIRKKPSVASSSKKLKTSQKLILDLFLNRILKKGKKS
VAKKLFLNTLKYLKYQPKLNPFIILETAIKKVLPTIELKTQYLEENLIYKPNFINKYKAFN
YAIKVILQISFTHSIKNYSKKLAKELLETFLGKSKSIKKKEEMFKDIEKLKNLD-
LERMIRLILKTFNLKLLNIVNTLFKQKLKELKIKHTNFIILPIVHKKFCVLKSPHVDKDA
REQFEIKLYKAIVDLNIFSTLLKILLEIPSGIIYTLMPTLQQLIRKKRSKKIKKRKILALSFC
PQKRGQCIKLTIVTPKKPNSALRKIARVSLSNKSEITAFIPGEGHNLQIHSNVLVRGGRV
KDLPGVYYKIIRGKYDCGGIKNRKQGRSKYGTKKQSMRKLKSLNIQAKLHAYVTYHN
LILTLCKVSGKIIGWESCGTLGFKNAKKRSPYAIQNTVLNISQLLKSLNIYNIYLIFNGPI
TNREIIYETLISKQIKIFKISEKINIPYNGCRLPKIRYIMNKILGVNLKNNKQIWIALTQIFG
IGKTTSLKILKTLKINTLKVEVLSIKNYLSLVKFLENYILEGNLKRLIYLHIQNLIDIKSYR
GIRHEKNLPVRGQRTRTNAKTQKKLRKSKKMITKYRIGLVI-
NKTQKTVKIQFKMFFFNKLLKKKTFKTKYYQVHDPFNICTFGDIILIKKISPISTLKSWK
LSMIRLAKIEKFKNQKKIIIKYKTLRNNLFQNSKKVKTLLNYIYLIKKFEELPKNSSFFRLR
NYCWKTGKTRGIYRFFGLCRNVIREFAYNNFLPGIIKSSWMKK--
KYNFYISYKLLKKLLKFKKKIIKTWSRTSTIIPLMLGLTIYVYNGKKHIPIYINEYKLGYKL
GEFSPTRYFGKHKAK--QKK---
-----------MSQIYTKAGKLVPTTYLKLGICYLTEFKTILTHKYNAIKISLKNHKILKQ------
----
KEFSVKNNKFTLYQVFTILDLRNSNIVTVQSFSKGKGYTGNIKRHNFNRGPMSHGSKH
HRLQGSLGAGTTPGRVFPGKRMSGQMGFTKITHKNLKILELDNIKNRIYLHGNVAGT
LNTLVLIKMLKNKKKRKIITLQCDSCIEKKKRTSGITYYKTTKNIKTTTFQLKLKKHCS
KCNKHCIFNEIHMSQIYTKAGKLVPTTYLKLGICYLTEFKTILTHKYNAIKISLKNHKIL
KQ-----------------
KEFSVKNNKFTLYQVFTILDLRNSNIVTVQSFSKGKGYTGNIKRHNFNRGPMSHGSKH
HRLQGSLGAGTTPGRVFPGKRMSGQMGFTKITHKNLKILELDNIKNRIYLHGNVAGT
LNTLVLIKM---------------
KFNKLKNCISLIHKIYTLIQLKKKVALATTKTKAEVKGSGKKPWKQKGTGKARAGSIRS
PLWRGGGIIFGPKYHSIFLKFNKYEKKLCLLYIFKLKEKNIFIINKIL---------------

KKLFSYINKNYLFLFTSINNFKTFLINTIYNINILNLLNAKYIFISFFTLNLLKYI-
MLKIRLKILGTPKNKIYKIIVLDT---
KKNKIIKKLGNYNAQFKYFTCNKIELLKYLNNGAYVTSSLRYLISKLY--------------------
--M---
KINLNKIKIGDFIQIKYLSETIRKPIISEYKGIIISKKNIILLKTIQYKTI-----
IYIRLDINNPNIFSIKKLNIIKSLKAKLYYLKFLE-----ISELI
>Synura_petersenii_MH795128
MLKTRYPKLEKIQISCGLGLNAQNRVFLQKATEEIRLITGQQPILTSAKNSVAGFKIRE
GMPLGLTVTLRREKMYAFLEKFIHLVLPRIRDFRGLSSTNFDKEGNYNLGISEQLIFPEI
DYDNVDQRRGFNITIVTSSKTPAEGFFLLKELGFPFMVRVKRGNIASKRRRKYLSLARS
FVGANSRLAIMAGEQVRQGLNFAYIGRRLKKRNFRRVWIYRINAASRTNIYSLFIGCLR
NLNIFLNRKILALIAFYDLASFNFIEMGIKTYKPYTPSNRTRSVLDFSFLSKNKPEKSLLV
SNQRAKGRNNQGRITTRHKGGGHKRRYRLIDFKRNKYEIKGEVFSIEYDPNRNSNIALI
SYSDGEKRYILHPENLKVGDFISSGKKSEVKIGNSLPLENIPLGTEIHNIELFPGKGGQL
VRSAGTYAKVIAKENNFVILRLSSKEIRLFKKECFATIGKVSNSDFYNVVSGKAGRSRWL
GKRPSVRGAVMNPVDHPHGGGEGRCPIGKPQPRTPWGKPALGVKTRKKRKSFIIRS
MSRIGKKAIFLPKDVVVTLTNNQIIIKGKYGVLQKTLSDKLAFNEKIVISKKSKEAHGLY
RALLQNMVNGVDKKFSKTLIAEGVGYRFQLEKNKIILNVGFTHPVAFEIPEDLSIKLES
NTKIQLSGIDREKVGFFAAKIRDMKPPEPYKGKGILYEGEKILRKAGKTGKMTLFPKQ
EVPKWFVVDATGKTLGRLATEVSKLLRGKEVSFFTPGVDQGNFVVILNAQKIEVKEQ
QKLYYRNSRRPGETFNQLKNRMPTRIIEQAVWGMLPKGGRNYYRRLFVYIDSNINYF
DSTEKKIMAKKIKAFVKLALPAGKATPAPPVGPALGQHGVNIAAFCKEYNAKTAEKIG
LIIPVKITIYEDRSYSFILKSPPASVLLAKFANVKKGSSQPNKEIVGNVTLEQVKEIATIKM
NDLNTNNMEKAILIIKGTAKSMGIKIMLSPKRTKYRKQQRGRLKGKACRNNKLDRGE
YGIQALEPIWLTSRQIEAVRRTISRYTKRIGKLWIKVFPDKSVTARAEESRMGSGKGAVE
YWVVVIRPGNILFEITGVPKEIAMEALKVACYKLPIKTRILSKMIQPQTYLTIADNTGAK
KLMCIRVLGNNRKYANVGDIIIGVVKDALPNMAVKRSSVVRAVVVRTKKTIRRIDGMG
IRFDDNAAVIINTENNPKGTRVFGPVAREIREKNFTKIISLATEVVMYAIAQTSGKQFLL
KPGQWYDVDYINVGDFISIKKILFFRAIQLGEPFLESIPAKIIQQVKGKKIIVLKTKPKKK
YTRTRGHRQPYTRVQIMVSATSKYIRISPTKIAIIISKIRGKTYKEALKILKYLPQKAGSIV
WQTLYSAVSNATNNFDLVKENLVIKEAYVNQGPILKRMRPRAQGRAYAIQKKMSHVT
IRVMAKKGLHPEWF-
NTKVYCDGKLVLEVGTTKKELSVDIWSGNHPFYTGSQKIIDTEGRVEKFERKYNLMA
HKMGAGSTRNTRDSQAKRLGVKCIGSQKVKNGSILVRQRGTKFKPGIKVGCGRDHT
LYALADGIVNYTVNILMKVRPSVKKMCVNCRIIKRHGKIMIICINPKHKQRQG----------
----

LTCLENYIKYSCFLIEPLEIGNGITIGNSLRRTLLSDLTGFSVIGVRINNLKHEFDTVPGL
REDVLEVILNLKEIVFKTSKGFLLQKGPLILTAGMFQLPKNIQILNPNQYIGTIVDTSEI
YLEIDIEHGKGYRLATLFVDGLFMPIKRVNYKIKLIKESLIFEIWTNGSITPKRSLQESLK
LLLNLYYPLLKIIKGLKWLKLHKSLKSSKLVKLAQFFKLLNKQRKQNKKNQTLFFKKIK
KLTRTVTKKILEAIVHETFTQFGNLSSSSLLDSLKLLGFYYATTAGISINIEDLKTPDSKK
EFLDNANKEIDFVSNQWQQGLISDTERFQTIIDTWNLATEGLKNRIIDYYQNFDPAN
NLYIMAFSGARGNMSQVRQLVGMRGLMSDQEGKIIDLPIQTNFREGLSSIDYIISSYGA
RKGIVDTALKTADSGYLTRRLIYVAQDLVIREINCSTKEGIIFLIGRTLLSIKSPKILDQT
LNEELLQELIFPLTLNVRSSLTCKSNGSICQKCYGWDLAQGKKISLGEAVGIIAAQSIGE
PGTQLTMRTFHTGGIFTETNKQIFAPFSGKFLIPKTLKAFSYRTNHGVLVLKLQQETK
FTILEKKEIFLNVGSYLYIATTSFVKKGQLIGEYSLQSFSIYTTIAGEVKFQDVLWIASGKI
FSLPEKMLSLKNLFNKALAKLKIISPSKGFINSFFI--

VHFEFTSLIKIKFSFLVRNHQYIDEHTVIGSIEIFSYEGLVYSIRYKMRTFFFITESDIFKIN
SDQKTINFLIFSRSGFFLKKDGFQIIYQHAIPIFLTRGTILNYNQGDFVLENKMFATLIN
YTQQTEDIVQGLPKIEELIEARKPKMGNEKN-
SLGEPITIDAHELLDLLFQYHRGTHRSLLKFQLVFVNSIQSIYQSQGVNISSKHIEIVVR
QMTSKVKIKEPGDTPFLPGELIRLSLLNEICYIFKSTKYEPVLVSTTTSSLSKDGFLSAAG
FQETKRVLTKAALEGSCDWLRGLKECIILGRLIPAGSAMFVNNISDLSEAQRGSFYRFLI
NGISEELVNLPNPFFAIRVLLYLFQNEIKLHGPLVNISDCLERDLTYSIQFFIKGEYNQIK
IRVKQDIFFGEIPLMTEEGTFIISGCERIIISQIIRSPGIYFHYTATIISNKGLWTKFILDQIY
MKLNEFNKLFIYDLLRYFGLSEISDSLKYPLHLTNAVIRLFYNSACFSIGEIGRYKINQKL
GLNLPKDITYLTAHDFIGIIDGLIELKYYDDDIDHIKNKQIRSIGELLQNQIRVGFSRLK
KFLTQGSSFPPVSAIKEFFKTSQLSQYMDQINPLAELTHKRRISVFGPNGLKRDHISTVI
RDIHPSQYGRLCPIETSEGQNAGLIMSLSLYGRVNSLGSLETPYFFMENGKIFSKTRPIF
LNPEQEANIKIAFGDLALKKVQKEYLSIKDNYVFSVQKVEEINFITTSPLQVVSLATALI
PFLEHNDANRALMGSNMQRQAVPLLYPQKPIVGTGLESSSILDSGMVIKTYCEGIVFF
ASSSSIIITYYLRKYYQSNQETSFNQRPLVWSGEKVFSGQIIADGPSTHDGELSLGRNLT
IAYMPWEGYNYEDAIVINERILLDDCLTSVHIEQYETNLSYGIEGSETNNIPYLQRHLNI
DGIVKIGSYVKEHDVLVGKVMPC-
EEDTSPEAKLIRALYFQDTSLKVPVMDVRVVSTISIAQIRKLQLGDKLSGRHGNKGIVS
RILARQDMPYLPDGTPIDIIFNPLGVPSRMNVGQIFECLLGLAGEKLGKRFKVTPFDEI
YGKEASRILVNQKLKEAATNCSWVFNDVSPGKILLKDGRTGEFFDNPITVGKSYILKLI
HLVEDKIHARAIGPYSMITEQPLAGKSQKGGQRFGEMEVWALEAYGASNTLQELLTI
KSDDIDGRNDMYESLPSPSIPESFLALIRELNALGLDFCLKKFEGYTDIEKDIFMFDYLQ
ISIASPKRIRSWADRTLP-

TGIVGEVQRPETINFRTHQPETGGLFCEKIFGPIKNWKCKCGKYNICEDCFVEIIESRV
RRYRMGYIELTCPITHLWYLKGVPNYLRLVLFEKNKGSEILKAALESIDLKKEIERQRSFI
NKESSPEKSMIRRIRILESFLSTKTNPSWMILTVLPVLPPNLRPLVELENGRLVAADVNE
IYRLIITRNQRLFDFMQYIPPELITTHGRKLLQEGIDSLIDNARLSKKTLCLNNKPLKSL
TEILEGKQGRFRQSLLGKRVDYSGRSVIIVGPNLRLNQCGLPYEMGVELFQPFLLLLK
TKATILKRNPFVWSLLSLTEKHSILLNRAPTLHRFGIQAFDPLIVLGQAIHLHPLVCTG
FNADFDGDQMAVHLPLYESSQLEAKTMMRPSYNVLSPSNGEVILKPTQDMVIGSYYL
TLMITKNKFFLQKWFSTEFQALSAFSQKKITLHTPILVRYYFLTFLETTPGRVIFSLNFK
NSIMARYTGPRIRIIRRLGLPGLTRKIIQNRRSSLDDYRERLLEKQKLRFNYGLTEKQLV
SYYKEAKRETGSTGNILLQLIESRLDCVIYRLGFAPTIPSARQIVNHRHILVNGKEINIPS
FLCQEGDIITVKDKKESKTLVGGNFLPTHLQVESETLIGKFLSPVKRKDILVRINELKV
VEYYSRMGQKTHPKGFRLVTTQNHLSTWYGDKFLYSNLIKEDYLIRSKIEDFISKIEINR
IEYVTITLHALFPNLTLLNIRNFIKNFFLNIKFIKNPFEDARLIAKFIADQLEKRTPFRRAV
KQTIKKVRKGVKVQVSGRLNGIEIARSEWKRDGRVPLHTLQANIDYTHQRAETIYGVI
GIKVWLFVMTNDLVSDMLTRIRNACLARHNFARVRYSKVNLSILKVLQGEGYIKNFEI
EEEKTVKIFLKYKGWIKKPLFSTIRRISKPGQRVFCGYKNFQKLIDVLGTAIISTSSGIMN
HKKATQLKKGGEIICYIGMYEEKVIQIKRVTKVVKGGKKLTFRAIVIVGDNKRKVGVGI
GRADDVNMAISKAILYGKKNLINVPLTLKFSVPHVVKAGYGACKIMLRPASQGTGVIA
GGSVRTVLELAGIRNILAKQFGSSNILNNAKATILALTLLMSRRISTKKRFPEKDSKYDN
LLVSLLVNRLLKSGKKRLSKRIVYKAFEFIQFRTNQNPILLLEKAIRNISPRVQLKAKRVG
GATYQVPTLLSKFRSTNIAVRWIVEFSRKRSGKGMSLKLANELLEAAKGLGNALKKKE
ETHKMAEANKAFAQFRRMFRILLRSFNNDLIDLACKQLRSLLIKTDCELKGVVSLPTKI
KRFCVLRSPHIDKDSREHFELRIYKRFIDLSTNSVIIDLLLELPAGVSCSLMPTIQQLVRL
NRLQNINFTKTPALKSCPQRRGICTRVYTTTPKKPNSAIRKVARVKLTSGFEITAYIPGE
GHSLQEHSIVLVRGGRVKDLPGVRYKIIRGALDCTGVKDRKQSRSKYGARKPVMKKQ
KKSVTVGIANIKTTFNNTIVTISDFLGNTLCWASAGSSGFKGARKNTPFAAQSAARNA
ALKALELGMEKIEIVIKGRGNGRETSVRALKATGLNILSIEDKTSVAHNGCRPPKKRRL
MARILGNNLSNKKKIYVALTCIYGIGIPTSKKILKTLQINDLKVAELKEENISALRDFLEE
FKLEGDLKRLVSLNIKRLIDINSFRGRRHLKGLPVRGQRTRTNNRTSRRQSLFKKMAK
KERIGIVVSNKPQKTIVVAIQTRYQHPKYGKILIKTKRYMAHDEEKKCNSGDLVLLEES
PPFSRHKKWILKMAKKSMLQREVKRQKLIKKYFEKRQRILNQLKKTSSLEEIFILNKNL
QKLPKDSSPIRLRNRCWKTGKPRGYSRFFGLCRNALRELAHDCFLPGVTKASWMSRSI
KKGPFIAYHLLKKINVLKKEIIKTWSRASTILPLMIGHTISVYNGQKHIPVFITDQLVGH
KLGEFVPTRTFRSHKTERKVKRMKQKNRKIVTQNNRNRMINRRYISTIKTLAKLLIKN
MKEITSKILNTFFSFLDKAVKKGILHKNTVARKKSKIRRSFDPMTQIFNSKGERIPVTIIK
GGPCYVTQIKSTENAGYNAIQLGYLESKKLTKPQLGHFSKVNLPPFRYLKEYTTLELN

YSLGEKFSVEMFNIGQFVNITGFTIGKGFTGNIKRHNFSRGAMTHGSKHHRAQGSLGS
GTTPGRVFPGKKMSGHSGMQKRTILSLEVIDIDLKENLLVIKGSIPGKSGNLVSITMAK
NKGPRITITLECTECRKNKKRSPGVSRYLSTKNRRNTPEKMELSKHCRYCNKHTIHKE
TKMTQIFNSKGERIPVTIIKGGPCYVTQIKSTENAGYNAIQLGYLESKKLTKPQLGHFS
KVNLPPFRYLKEYTTLELNYSLGEKFSVEMFNIGQFVNITGFTIGKGFTGNIKRHNFSR
GAMTHGSKHHRAQGSLGSGTTPGRVFPGKKMSGHSGMQKRTILSLEVIDIDLKENLL
VIKGSIPGKSGNLVSITM--------------
LSDSLEKQAIGVIHRVYLSQLKNSRKYLASTKTKSEIRGGGRKPWRQKGTGRARAGST
RSPLFVGGGVIFGPKPRTVFKKINKQEKKLATFLSLFLKIREFKFFDEKFSIKTKKVCKIL
VISEKKILFVLAKPNKNFWLSARNLKNIEITTASCMNIEQILKANSILLSRECFELIKLSYM
LKIRLKKLGRKKKPFYRIVLMENLSKRDGKSLVEFGFYDPITKIINIDKIALSKYLNFGAY
PTNTVRHLITNMIMLIKYPSLTEKSMNLYGNRQYTFIVDRSLTKTEIKYGIQKIFNVSIT
DINTCNLPIKKRRRTLFKKAYIKLKEGEKIDLFNMSIEKKKNPLVEIGNIIRIGYLIPEGEK
ERTQYYEGLVIAIKNRGIGKSFLIRRNVQGIGIEQIFLLNSPKIISIVKKQSSKVRRSKLYFL
RELRGKSTRLKIKF
>Thalassiosira_oceanica_GU323224.1
MIKKEYPKLKKISINRGLGLAAQNSNILKKSITEFTRITGQKPLITRAKKAVAGFKIREN
MELGLSSTLRGEKMYNFLTKLIFFTFAQIRDFRGLSVRSFDKAGNYTFSLKEQLIFPEIE
YDDVDQIQGLSVTLVVDSSTPKNGMILLKFLRFPLMVRVKRGNVARKRRKKILQLAK
GYKGAHSRLFRVANQQVMKALRYAYVGRKQKKRVFRKLWISRINASARQTTYSQLIN
CLKENKINLNRKMLAQMAVLDYSSFYEIIMSIRLYKAYTPGTRNRALSSFSEITTGKPEK
SLIRKNHRQKGRNNRGVITIRHRGGGHKKQYRLIDFKRNKYNVPAIVNSIEYDPNRNA
RIALVHFVDGEKRYILHPNNLNVGDTILSGKGISLDIGNTLPLEEIPLGTSVHNIELIPN
RGGQIVRSAGTSAKILAKEGNYVTLRLPSKEIRLIRKECFATIGEISNNDAFLVQSGKAG
RTRWLGKRPTVRGSVMNPCDHPHGGGEGRAPIGRSRPLTPWGKPALGKKTRKTKKS
YILRRMSRIGKLPVKLPASVDVTNDNSLLTIKGKFGTLEKTIPEIFVVNGTLIVRRTNKA
LHGLYRTLINNMVIGVSEQFLKTLMLQGVGYRASVQGKTLVLNLGFSHPVNIDIPEAI
NVEVVQNTTINIKACDKEQLGLFAAQVRSWRPPEPYKGKGILYKDEQILRKAGKSGK
MTFIPSTNKRKWYVIDCKDKQLGRLASSIVPLLTGKTKSIYHPSLDVGDYVILINSEELK
IRDVERFHVYEPGRPGSSLKRLVNILPKRIIENCVFNMLPNGKKHLVKRLKVYQGAVH
PHAAQDPKVLMPKKITALIKLALPAGKATPAPPVGPALGQHGVNIAAFCKEYNAKTT
EKAGLIIPVEISVYEDRSYTFLLKTPPASVLLANAAKVKKGSSTPNRVNVGSVTQAQLE
EIANIKLPDLNTTKISSAVRIVEGTARNMGITIMLSPKRTKYRKYHRGRMRGKATRGNE
VTFGQYGLQALEPSWITSRQIEATRRTITRYTKRGASLWIRIFPDKTVTARAAESRMGS
GKGAVDYWVATVKPGTILFEISSVPEEVARAAFNLAAYKLPIKTKFIIRMIYPQTMLTV
ADNTGAKKIMCIRVLGGNRKYGKIGDTIIGVVKEAIPNMPVKKSDVIRAVIVRTSKTIR

RPDGMYIRFDDNAAVIVNMENNPRGTRVFGPVAREIRDKNFSKIVSLAPEVLMYAIVEI
SGRQFWIEAGKYYDLNRIPTGKQIKLNRVLFVNELLIGKPYLEVVEGKILKHFRSRKTV
VYKMRPKKKTRKKQGHRQDLTRVLIMVKAVAKYVRISPHKVRKVLDQIRGRSYQEAL
MILEFLPYDAGSPIWQVVHSVAANAKNNYNLDKKKLIISEIFADEGPKLKRIRPRAQGR
AFKILKPTCHITVVVMPKPEIHPTWFPEATVLCEGKTLCSIGSTKPQLQLDVWLGNHP
FYTDSQTLVDSEGRVERFMKKYGLMAHKKGAGSTKNGRDSNANRLGVKRFGGEKVK
AGSILVRQRGMKFKPGSNVGSGKDFTLFALVDGTVKFDVNIIMKVRPSVKKMCDKCR
VIKRHGKIMVICKNPKHKQRQGMAVPKKRTSKAKKNSRKANWKGKAAKSAQKSLSLA
KSILKTTSFVYMIQCLKSETDYGQFLIDSLNSGQGITNGNLLRRVLLGDLEGIAITGVRI
AGVKDEFSLIPGVREDILEILLNLKGVILKSNFGRLRIQGPAVITASSIQLPPEIETINPNH
YIATISTSDIVEIEFKIESGRQYRLAFIETDAIFMPVQKVDFKVENIKERLFLDVWTNGSI
APQDAIISASNSIIAFFQSIIAIEELQLSVRPYNCLKRARINTIGDLLEYSPLLELFGRKSAD
EVFATLKNQNTLINKKQLKELLAWSFSKYDAMQASLLADELKYLGFKYATQAGISISIE
DLKVPATKNEMLEKANQDILNAEKICLKGKITDVERFQKIIDTWSLASESLKENVVSYF
KIYDPLNSVYIMAFSGARGNLSQVRQLVGMRGLMADPSGEIMRVPIKKNFREGLTITD
YLMSGYGARKGIVDTALKTANSGYLTRRLIDIAQDIIIREKDCHTTTSFSVIIGRVLAKS
VYIAIASTQLTAKLLEIFKKILRFHIRSPLTCNLYHSICQMCYGWDLSNQNLVDLGEAV
GILAGQSIGEPGTQLTMRTFHTGGIFTEARQQVISPVNGIIKFSKLLKTIILRTNRGDEV
LLTKNSGSLILIKIIQIELLRNTMLFIKNNQYVKKSAIIGELISTEKQILSDTAGEIFITQLL
WILSGQVYQAPNNFYTDYKINKSYIFRTKVINQHPGHIKNYLINGGIILSEETYKLNCDI
LLVGNFIFEVIPNIISKTAGIINVIIEEIAIKEGKVYYPGEILFNNVEITQPSLCELIRPFTIYE
IVNLISQSINLLQSCLLFEPKQFIDSYTNLGYLEHLTKSVEIVKFKSKKKQVFLISNDNCI
TVDKKKNKTELLNVNQIGKILIDNGKFLTIQKGRPYFFQEVNNVYCKNGEFIEKGEVI
GLLNFEKEITGDIVQGLPRIEELLEARKRKTNQKKIQKLGTTIKINPHNLLKIYFNYYEA
SYRTFKKIQLLILNAVQSVYQSQGVSIANKHLEVIIKQMTTKVLITHEGHTPLLPREVV
DLYHIQYINQIIEAHYYVPLLRGITKAALNNPSFISAASFQETTRVLTKAAIEGRVDWLR
GLKENIIIGHLIPSGTGMYTTALPDFIEMQRVSFCWFIAQGLNEELSTFSRIYDFQNTEY
ILFGQEYSLVKPVYNIVRAKKYTANYSAQLIIPLEVRNKKIKYHNKFPIINLPLMTSAATF
VINGCERVIVSQIIRSPGVYFLYTATLIPEYGSWVRFGFQKIIIQLDKVTQKPIIHLLKEM
GVNEICQNLQNSEFFYFSEFSRIFDPAYYRLGKIGRSKLNNRLNIQLSKRIVTITYQDIFA
IIDKLITLSISKDDIDHLKNRRVRSVGELLQNLFRIGFQRLSRKLRSQTNKFIVATIREFF
GASQLSQYMDQTNPLSALTHRRRISGLGPGGFDRDRISFAVRDIHPSHYGRICPIETPE
GQNVGLIASLTTSARVNESGFLETPFWRVLNGKVIKTGNPIYLTADIEDFYKIAPADIST
NYLTKELIPIRYKQDFLTVTPSEVDFIAVSPIQVVSVAASLIPFFEHDDANRALMGSNM
QRQSVPLLLPQKPIVGTGLENQIALDSGMVINAQRDGIVSSVTADKIIILYDLQKYQRS
NQETCINYRPIVWEGEQIKSGQMLTDGPGIISSELSLGQNVLVAYMPWQGYNFEDAIL

INERLVYEDIFTSIHIERYEIEIDRTAEISETNNIPNLVKNLNEDGIVTIGTFVKPGDILVG
KIVPKDDSEQLPESKLLRAIFVRDTSFRMPVIETLTFNRIFIAQIRKIQVGDKIAGRHGN
KGIISRILARQDMPFLPDGTPIDIILNPLGVPSRMNVGQLYECLLGLAGHKLNRRFKIL
PFDEMYGPEVSRILINKKLRQASNDEAWLFNPYSPGKMVLLDGRTGKEFDNPITVGN
AYMLKLIHLVDDKMHSRATGPYSLVTQQPLGGKAQHGGQRFGEMEVWALEGFGAS
FTLKEILTIKSDDMEGRNETLNSIPTSGIPESFKVLVQELRSIGLDLSTYRIDEFGEIELN
LIMFDYIKIKLASPMRILEWSHRKLP-
NGFVGEVQKSETINYRTFKPEMDGLFCERIFGPSKSLECACGKYKICERCGVELTESRV
RRHRMGHINLIYPVTHVWYTNSRPNYVALLLIKRIKGAVLIKRELEKLNLDQEIFKTRN
FITICSKLRDQSIKRIRILENLLTTGVNPAWMIITILPVIPPALRPMIQLEGGRFATSDLN
ELYRRIITRNNRLLRLLEIDAPQLIIRNEKRMLQEAVDTLIDNGKRGKIALSASNRPLKS
LSDIIKGKHGRFRQNLLGKRVDYSGRSVIVVGPSLKLNQCGLPYEMAIELFQPFIILINQ
GAKLLQQNPSIDTVLKVLMNHPIFLNRAPTLHRLGIQAFEPILVQGRAIKLHPLVCSAF
NADFDGDQMAVHVPLTLEAQAECYMLMLAPYNFLSPANGDPIIMPSQDMVLGCYYL
TVNNINGLLGTNHYFADLNDVILAYNQNQIEIHTSIWVRYTIVQYLQTTTGRVLLNYT
IKTTLMSRYRGPKLRITRRLGLPGLTQKTSKKTGKKKTEYGLRLEEKQKLKFNYGITES
QLYHYIKEARRRKGVTGLILLQLLEMRLDTICFSLGFAPTIAAARQIVNHGHITVNNKV
VDIPSFQCQINDVIGVKAKSTSKNIIENNIPPTHLILDKSKLEGTVKNYCDRNELLLDL
NELLVIEYYSRMGQKTHPLGFRLGITQEHRSSWYANFKHYSTLLKEDDQIRTYLNKFIS
NVHINRNDQIELNIETGRPILVLANIKKFLHQITINVIEVENINSNASLIADLVVQQLED
RVAFRRAIREGLKCADNGIKIEVSGRLNGAEMARSEWIREGRVPLQTLRADIDYATKEA
NTIYGVLGVKVWLFKMVTDSIADMLTRIRNANMVKHQIVEIPATKMSRAIAIILKNEG
FIENFEIYISQYLLLSLKYKGQSRERVITKIKRISKPGLRVYANSKTLPRVLDGLGIAILSTS
KGVMTNTQAKELGVGGEVLCYIWMFVERLIKISRVTKVTKGGKKLSFRAVVVIGDEN
GQVGVGVGKAEDVVNAFKKAKTDGRKNLIQVPITKALSIPHGVVGDKGACKIIMRPSI
EGSGVIAGGAVRTVLEVAGIKNVIAKQLGSDNLLNNARAAIVGLESLMSRRNISKKRFP
KADPTYNSYLVSLLVTRILKSGKKNLAQNIVNGAFEIIKSKTNEDPLVIFEKAIRNASPV
VEVKARRIGGSTYQVPIEVSSFRATNLALRWIIQYSKQRVGRTMSSKLASEIIDTANDIG
NTIKKKEETHKMADANKAFAHFRRMIRVRLESFNRELLNTSIQKISEIIRDTGVYKAGV
ASLPTDKRIYCVLRSPHVNKDSREHFEIRTYKRIIEIYYDANIFDLLVDLPPGVFYRIMPT
IQQLIRSKRIQINKKTKSPALVNCPQRRGVCTRVYTTTPKKPNSAIRKVARVRLTSGFE
VTAYIPGEGHNLQEHSVVLIRGGRVKDLPGVRYHIVRGALDSGGVKDRTQRRSKYGV
KKPKMKKNRTNLGTGVVHIQSTFNNTIVTITNITGDTISWASSGSSGFKGARKGTPFA
AQTAAEKAALDALSLGIKTVEILVKGSGSGRETAIRSIEGAGLEILSIQDITPVPHNGCR
PRKRRRVMVRLLGIDLPKNKRIEYALTYIHGIGLTSAKKIVKLAEINETRTNEITVEQSV
ALRNILEELKLEGDLRRFNGLNIKRLNEINCHRGKRHRNSLPVRGQRTRTNARSRRGS

KKTKKMPVKEKIGIVVSNKMQKTVVVKVESRYPHPIYSKTMIKTSKYLAHDEMSECNI
GDQVLVRECRPLSKKKRWSVAMAKKSMIEREKKRIKLHKKYDSKRQMLLNEYNNTSD
FNLKLEIHSKIQRLPRNSSKIRIRNRCWKTGRPRGFYRDFGVSRHVLREMAHQCLLPG
VTKSSWMSRSLKKGPFVAYHLLKKINKMKKDVITTWSRSSTILPNMVGFTIAVYNGRQ
HVPVFISDQLVGHKLGEFVSTRTFKSHKADKKTKRMNKSAKKRILISKRNNLQNRFYK
SSVKTLTKKFLNDLEVFLEKTLASIYSLIDKGLKKNVYHKNTAARKKAKLAALLKNMT
QIFDESGNIIPVTILKVGPCIVTQVKTTLNDGYNAIQVGYGNTKSLTQPQLGHLQKSN
IQPLKYLKEFRVNEADFSVGQVLNVDSFIEGQLINVTGKSIGKGFSGLQKRYNFSRGP
MTHGSKNHRAPGSIGMGTDPGRVLPGKKMAGQLGNKMTNIKKLKIVQVNNNENIL
VVKGAVPGKPGNLLSIVMAKNKGARILITLECTECRTNNKRSSGVSRYLTQKNRRNNP
QRIELKKYCPHCNKPTIHKEIKMTQIFDESGNIIPVTILKVGPCIVTQVKTTLNDGYNAI
QVGYGNTKSLTQPQLGHLQKSNIQPLKYLKEFRVNEADFSVGQVLNVDSFIEGQLIN
VTGKSIGKGFSGLQKRYNFSRGPMTHGSKNHRAPGSIGMGTDPGRVLPGKKMAGQL
GNKMTNIKKLKIVQVNNNENILVVKGAVPGKPGNLLSIVMQKSIEFN-------
IKLNVLDSGNYLIHRDILRQQISQKQGTVSTKTRSEVRGGGKKPWQQKGTGRARAGS
SRSPLWRGGGVIFGPKPRMTVLKLNKKERKLAVQTLLYNKRKNILVSAKLFTNKTKKF
YSFCIDLNKTKLLLVSESSKTFWLATRNLKNVEVISSANLNTLSLLKAERILITPIALNNI
KEIYMLRLRLKRNGRKRQPTYRLVIMENTTRRNGRPVDEVGYYNPITKESYFNEEKIA
KWLNHGVKPTTTVFQLLLKLI-
IIKYPIITDKATRLLANNQYSFVVNPKSDKPTIKAAIEYLFNVKVVKINTAHLPKKKKRK
SHYKKAIVTLSEGDTINLFAMNLHKPNLPKIQIGDSIKLGVKIIEGNKERVQFYEGTVIA
KKNSSINTTITVRKVLQGIGIERIFLIHSPKIASIEIIRHAKVRRSKLYYLRNLRGKASRLKQ
RF
>Tisochrysis_lutea_MF795089.2
MIKKLYPKLLKISVNRGLGEESRNSKELDINLKELAAITGQQPTINEARKSIAGFKIRDG
MPIGASVTLRKERMYSFLERLIHITLPRIRDFRGVSAECFDGRGNYNLGLKDQLIFPEIS
YDDVNQLQGFDISIVTSAKTDEEAYSLLKYLGMPLMVRVKRGNVAIKRRKKIMKLAK
GFKGAHSRLFRTANGQVMKALVYSYIGRKRRKRDFKRLWICRVNAASRALTYSKLRNL
MRNSSLVINVKMLAQLALFDKKAFCELIMAIRLYRAYSPGTRSRSDVYFTDLTKIKPEK
SLTFGKKACSGRNNRGLITLKGRGGAHKRKHRIVDFKRKSSSIVARVATIEYDPNRNAR
LALLHYDNGVKKYILAPRSLKIGMEVCAGKDAPIEIGNALPLASIPLGSTVHNVELTLG
KGGQLARAAGTYAQIIAKEGDFVTLKLPSNEVRLVYKECYATLGQVGNVDAINVCLG
KAGRSRWLGKRPKVRGVVKNPIDHPHGGGEGRSPIGRSKPVTPWGKPALGIKTRQAK
KSYILRGMSRIGKQYISIPDKVTVKLEGQKVLVDGPKGKLTRILPSFITIDRKIFLNKLAQ
ALYGLSRTLLGNMVTGVSTGWHKKLKIAGVGYRAQLDGKDLVLNMGYSHPVKMITP
PTLTVSVENPTNITVSGMEKEIVGEFAAKIRAVRPPEPYKGKGIAYENEIIRRKAGKTGK

MLLPKRTKFRKMHRGRLKGIATRANTVVFGDYGLQALEPIWLTSRQIEATRRSITRYV
RRTGKIWIRVFPDKSVTERAAESRMGSGKGAVSYWVAVIKPGTVLFEIDGLNRETAYQ
VLKLAAYKLPIKTKIISRMIQPQTCLNVADNSGAKKLMCIRVLGTNRRYGHVGDVIIGV
VKDATPNLTVKRSDVVRAVIVRTKQSVRRKDGSRLRFDDNASVIINKENNPRGTRVFG
PIARELKDNGFTKIVSLAPEVL---

MFLAVSKYVRMGPNKVRRVLRQITGKSYAEALLLLEFLPYASCEPIIKVLRSAVANAKD
RSGADESKLIVKQAFANQGPVMKRFRPRAQGRAYRILKPTSHITIVVMPKPSIHPDWY
PNAKVYCDGQLVLKVGSTKPSLNVDIWSGNHPFYTGSQTIIDTEGRVERFMRKYGIM
AHKKGAGSTKNGRDSNSKRLGVKVYGNQAVKAGGIIVRQRGLTFKPGANVGVGKDY
TLFALKEGIVKFEVSLAMKVVSSIKNRSKDCQVVKRRGRIYLICSDPRLKVRQG----------
----
MVHCLQSETETAKFCIDPLDKGQGVTLGNALRRTLLSSIPGVAILGVRISGVEHEFSIV
PGVKEDALEILLNLKQIIFKGQITRLNIQGPGIVTAQDIDLPSGLELVDKHQYIATITTT
MNLEMEFLLEQGRNYTLLFLAVDAIFAPVKKVNFFVETSKERLILEIQTNGSITPWIAL
NSAAETLKQMFSFGILIEELELSVRAYNCLKRAQIHTIGELLQYSKDNLLFGQKSADEV
CENLNEKNSLINKRSLKDLMYQAFLNYGIVKSSIIADRVKNLTFHYATKSGISISVEDLH
VPYKKRELIGLTSNEVEITKKRYDVGGITSVERFQKTIDIWNNANNFLKDEVLTYFRES
DPLNPLYIMAFSGARGNISQVRQLVGMRGLMSNPQGQIIDLPIQSNFREGLNVTEYIIS
SYGARKGLVDTALRTADSGYLTRRLVDVAQDIIVREEDCGTRESLAVLKGRLLAEPFF
VAPANTEINENFLTYLKLKDTLKVRSPLTCSAIRSVCRNCYGWHLSHSKLVDLGEAVG
IVAAQSIGEPGTQLTMRTFHTGGVFSDLTKQVRSPISGTVQFNENTRASLFRTMHGKI
GFRVKDAIHLLVTNTISLDIPEESLLLVNSKQEIYENEIIAEIQKEQKEIYSEASGEVFFNN
LIWILQGSFYNLPSFNGTKLKPKNTFLSRQGIVNRYAGQIKLFDLTGGIVIPENTYKAV
RNPFKVKNGDTEILPNVFSPIGGLVQIVEQEVLVKPGEFVQPGDFIINKIIAQRLVFLEL
VRPVTVYSVVNLLQTFLVVTNVRYFVADGQYISAQTLIAQVETAVHCSSTLASNENFK
EILILQSTDLKKISLEGQTTDLIKSPYSGQVFQIENNYLLIRLGRPYLISAGTILHVNSGA
LVKDGDILATLVYGTIKTTDIVQGLPKVEELLEARKVNHTTLLFVNVADPLTISPHTK
LEILFTYYEACRLSFKNLQLFLIGEIQKTYRSQGVDIADKHVEIIVKQMTSKVCIEDSGT
TTFLPGEVLNFQKMAAITTAANQKLYSPLLLGITKASLNSDSFISAASFQETTRVLTEA
AIEGRKDWLNGLKENVIIGRLIPAGTGMYKYFLPNLLDVQRASYCWFLEKGLVQELET
FSVIRDLGDLELNFATKFYTIKAPKYTLDEVKRKDSTYSVRIFIRAELLDGTEAQEKQVF
LGEIPLMTNSGTFVVNGIERIIINQIVRSPGIYYTYTASLISNRGTWVKFEIDKIYIKIDKA

KKFSIYLFLKALGLEEIFANIENSRYFSKTDLAKFFDPKKYDLGYVGRHKLNQTLNLNI
DSDIRILTSRDLISIVNQLIKFRFVSDDIDHLGNRRIRSIGELMANQIRIGLNRLERIVRER
MAIKLAASIREFFGSNPLSQFMDQTNPLAELTHKRRLSVLGPGGIARDRAGFVVRDIH
PSQYGRICPVETPEGPNAGLIGSLATYGKINSYGFIETPFYKVKQGKVLRNSPPLYLDAI
TEDQFKLAAGDVATDFIKDPIIPVRHRQEIITVPRAEVEYMAISPIQVVSLATSLIPFLEH
DDANRALMGSNMQRQSVPLLYADAPLVGTGLESQTARDSGMVILSFNCGKVISLAK
GQIIVGYPLTKYQRSNQDTCINQKAVVSLHEKVTRGLVIADGTSTETGELAVGQNILI
AYMPWEGYNYEDAFVINERLLYDDLHTSIHIEKFETESRQTKLGPETRELPNIVSKLDE
NGIIHIGSWVESGDILVGKLTPKGESDYLPEGKLLRAIFVRNTSLKLSVLDVKIFSKVYIA
QIRKIQIGDKIAGRHGNKGIISKILPRQDMPYLPDGTPVDILLNPLGVPSRMNVGQIFE
CLLGLAAEHLNSRFKIIPFDEMNGVEASRGLINNTLTEAAAVQPWVFSNHHPGKIMLT
DGQTGEVFDNPVTVGRAYVLKLVHLVDDKIHARSTGPYSLVTQQPLGGRAQQGGQ
RLGEMEVWALEAFGAAYTLQELLTIKSDDMQGRNETLNAIPRPGTPESFKVLLRELQ
ALCLDIAAYKIDERGDKEINLM-FDYIKINLASPSRIREWGERTLP-
NGIVGEITKPETINYRTLKPEMNGLFCERVFGPVNDWECHCGKYKVCERCGVEIIDSK
VRRHRMGFIELASPATHVWYVKGRPSKIALLLDITVKGADAIKTLLKNINLQQSAGEIR
EILPFARFFREKLIRRLRVIDQFLASKFDPAWMVLDILPVLPPDLRPMVQLDGGRFATS
DLNDLYRRVINRNNRLARLQEIVAPELIVRNEKRMLQEAVDALIDNGKRGRAVVGLN
NRPLKSLSDIIEGKQGRFRQNLLGKRVDYSGRSVIIVGPELKLNQCGLPREMAVELFQP
FVILIYEGAKILQKNPLTWEILDVMEGHPILLNRAPTLHRLGIQSFEPILVSGRAIRLHPL
VCPAFNADFDGDQMAVHIPLSLEAQTEARLLMLAPNNFLSPATGDAILTPSQDMVL
GCFYLTANNPAQQLPTNYYFANFEDAIMAYQHSKIHLHAFIWVRCITSQFIRTTPGRIL
LNEIFQ---
MARYRGAKLRITRRLGLPGLTSKITKRTPKKLTQFGVRLEEKQKLRYNYGVSEKQLRN
YIKQAKSVKGTTGTILLQLLEMRLDNLVFRLGLAPTIAAARQLVNHKHIKVNNSMVSI
PSYQCQPGDVISVKDSSASKALVTTYLSPQHLDFDKKNLSAKVLGIVDREWVALKLNE
LFVIEYYSRMGQKTHPTGFRIGVNKSHDATWFANYGAYSQILKEDHQIRKFFEKEISKL
EIKRKNQLELLIHAARPKAIVTQIKKLTKQIRIKVIQVNKMETESTLVARALAEQLEKRV
AFKRAMRQISQRLKKGFKLQVSGRLNGAEMARDEWLREGRVPLQTLRADISYATARA
YTTYGVIGIKVWIFNMVNDTIADMLTRIRNANLAKHQIVQIPLTKVTKNIAKVLLAED
LINSCEELKRSSLLLSLKYNGKARVPSIQKIQRISKPGLRVYSSAKKMPRVLGGFGTAIVS
TSKGLMTDKEARQQQIGGEILCYIWMWEERVVSVERVTKVVKGGKKLSFRAVVVVG
DQQGKVGVGVGKASDVSTAVRKAVTDGKKNIISVPLTSSNSIPHKINGRFGAAKLVLR
PSAPGCGVIAGGAVRIVLELAGVQNILSKQLGSNSLLNNARATIDGLSNLMSRRSKSKR
ILAQPDPIYNSRLVTLMVIRVLRSGKKSVAQRIVYKALEIIASKTDENPILILETAVKNVT
PQVEVKARRVGGSTYQVPLEIRAYRGTNISLKWLIEFARDRSGRGMATKLANEIMDAA

KNTGGAIRRKEQTHKMAEANKAFAYFRRMLRIALKAYETSLLNESCTQILEAVEKGGI
KAIGPIPLPTKRRIYCVLRSPHVNKDAREHFEIRTHKKIIDIYKPTAVMENLLDLSAGVD
IEIMPTIQQLVRFERQTGEKKTKSPALIACPQRRGVCTRVYTTTPKKPNSALRKVARVR
LTSGFEVTAYIPGIGHNIQEHSVVLIRGGRVKDLPGCRYHVVRGSLDASGVKNRKQGR
SKYGGKRPKMKKSKKTVNTGVVHIKSTFNNTIVNITDKQGNTLFWASAGGCGFKGA
KKSTPFAAQSAAEKVGSMAFEQGMRQAEVIISGPGNGRETAIRALQGCGLEVLLIKDI
TPVPHNGCRPPKKRRVMVRISGVDLPRNKRVQIGLTSIFGIGYTSADKILAEAGISDIRC
SDLSDEQVASLRAIIENYQTEGDLRRLYSLNIKRLTEIGSAAGRRHRVNLPVRGQRTRT
NARTRKGKVKTNKMVAKEKIGTVVSDQMLNTRIVAVSDRISHKQYRKVITRTKRYVA
HDINCGAKIGDKVKIQETRPISKTKNWVLVMAKQSMIQREEKRERLIRKYSAKRKALK
NNLKLVSGYQEKLTIYKKLESLPPNSAPNRHRNRCWATGRSRGFYRDFGLSRHVLRE
MAHEGLIPGLTKSSWMTRSLKKGPFVASHLMAKIEKMKKDAIKTWSRASTILPTMIGH
TFAVYNGQQHVPVFVTDQMVGHKLGEFSPTRTFRSHKKDKKSKR----------------------
----
MTQTFDSNGEVVPVTLVKADACQICQIKTVETDGYNAIQVGYIALSKISKPLQGHLEK
SGAKGYRKFGEFRVDNDDYTLGVELTAASFSPGQKVRVTGTSTGKGFAGNQKRHNF
SRGPMTHGSKNHRLPGSIGAGSTPGRVYPGKKMAGQLGNKKITIKNSEILFVSSEENIL
VLKGSLPGKVKNILKISMAK-KGARIQVTLEH---RCEA----
GVYRYHTTKNRRNTTERLELKKYSPVTKKHETFKEIKMTQTFDSNGEVVPVTLVKAD
ACQICQIKTVETDGYNAIQVGYIALSKISKPLQGHLEKSGAKGYRKFGEFRVDNDDYT
LGVELTAASFSPGQKVRVTGTSTGKGFAGNQKRHNFSRGPMTHGSKNHRLPGSIGA
GSTPGRVYPGKKMAGQLGNKKITIKNSEILFVSSEENILVLKGSLPGKVKNILKIS--------
----

MLKIRLKRFGRKAQPCYRIVVTDSRVKRDGRAIEEVGFYNPLTDETHLKFDRIVERLKT
GAQPTETVRNILLKAKMLIKYPILTEKTSRLIEQNQYSFAVDKKADKVSIKAAIEELFDV
KVASVNTAHMPLKKRRRAQHKRAIVTLAPENSITFFEMNYKKEQVPTLKIGDTVKVVI
YLSGKEKERIQVYEGVIISKHTNPTNATITVRKMFQGGGIEKVFLLNSPWIKEITSLSSA
KVRRGKLYYLRDRMGKSARLKRSF
>Triparma_laevis_AP014625.1
MLKTLYPKVEKIQINRCLGLAASNNNILKKSIEEFTAITGQKPVVTKSRKAIAGFKIREQ
MELGVTVTLRGEKMYAFLDKLMNITLPQIRDFRGITSKGFDKNGNLNLGLVEQLIFP
EIEYDDVDQMRGFDINIVTTAKTKEEGKALLKNLGFPFMVRVKRGNVARKRRKKILH
LAKGYRGAHSRLFRVANQQVMKALRYAYVGRKQKKRTFRKIWITRINSASKLLTYSKLI
HMFKKSNIDLNRKMLAQIAVLDPMTFNKLVMSIRLYKAYTPGTRNRILSSFDEITTNT
PEKSLIQKNQRNKGRNNRGVITIRHRGGGHKKRYRLIDFKRNKYNVQAKVASIEYDPN

RNARIALLHYIDGEKRYILQPNKLNVGSFVISGQDVPLVIGNSLPLASIPLGTSVHNVEL
IPNRGGQIVRAAGTSAKILAKEGDFVTLRLPSKEIRLIRKECFATIGEVSNNEAILVQSGK
AGRTRWLGKRPTVRGSVMNPCDHPHGGGEGRTSVGRTRPLTPWGKPALGIKTRNK
KKSYIIRRMSRIGKLPIKVPNGVEINYNVPNLTVKGKFGELQQVIPDSIIIEGNLIVSRNS
QALHGLYRSLINNMVTGVSEQFKITLLLQGVGYRAAVQGKKIILNLGYSHQVELDIPN
GITINVIKNTTVVLEGCDKEQLGLFASKIRAWRPPEPYKGKGIRYENEIVKRKAGKSGK
MTFIPSSDQKKWFLVDATDKTLGRLSTEVANILQGKNKVNFYPSVDMGDYVIITNAE
KIRVKEEAKLYRRHSGRPGETLKAVRSRIPERILEKAIKGMLPKGGRKMYTRLKVVQGS
KHKYIAQKPELIMPKKITALIKLALPAGKANPAPPVGPALGQHGVNIAAFCKEYNAQT
GDKGGLIIPVEISVYEDRSYTFILKTPPASVLLAKAAQIKKGSATPNSVNVGSISKTQLEE
IANIKLPDLNTNNIVSAMKIVEGTARNMGISIMMSPKRTKYRKFHRGRMRGKATRGN
TIAFGDYGLQALEPSWITSRQIEATRRTITRYTKRGGKLWIKIFPDKSVTARAAESRMG
SGKGAVDYWVATVKPGTVIFELGGVPLEVAQVAMKLAAYKLPIKTKFITKMIQPQTV
LTVADNTGARKIMCIRVLGGNKKYASVGDTIIGVVKESIPNMPVKRSDIVRAIIVRTRK
TIRRNDGMFIRFDDNAAVIVNPDSNPKGTRVFGPVAREIRDKNFSKIVSLAPEVLMYAI
IEVSGRQFWIETGKYYDLNRIPTGTQITLNRVLLFNDLLIGKPYLEVVEGKILEHLRGR
KTIVYKMRPKKKTRKKQGHRQDLTRVLIMTRAVAKYIRMSPFKVRRVLDQIRGRSYQ
EALMMLEFMPYRSCGPIWQVLHSAAANAQNNFGLEKKNLIIDVAYADEGPKLKRIRP
RAQGRAYKILKPTCHITIVVMPKREIHPTWYPDAKVFCDGKLVFTTGSTKPELHVDV
WSGNHPFYTGSQKIIDSEGRVDRFMKKYGLMAHKKGAGSTKNGRDSNANRLGVKRY
GGETVKAGNILVRQRGMKIKPGINVSYGKDFTLYALEHGVVKFDVNILMKVRPSVKK
MCDKCRVIKRKGRVMVICENTKHKQRQGMAVPKKRTSKSKKNARKSVWKNKANKQ
AQKALSLAKSVLKSTSFIYMIKCLKSEIEYGQFLLDSLSPGQGITIGTLLRRILLGNLGGT
AVTAVRIAGATHEFSTLEGVREDILEILLNLKGIVFKNNLGRLKIQGPAVITADCLQLPS
GLEIVNPNHYIATISTTNILEMELQFEYGTGYKLAFLEVDAIFMPVQKVDFKVETNNE
NLILDIWTNGSITPEEAIFQSSQLIINLFEQLISIEELQLSVRAYNCLKRAQINSISDLLKYS
PLQEIFGQKSADEVFDALKNQNTLVSKKQLKQILSWTFTNYGSVQGSFLADELKYLGF
KYSTKAGISISIEDLRIPPIKNVMLENSTNNILKTEKTCLKGDITEVERFQKVIDTWNVT
SETLKDEVVSYFRNYDPLNSVYMMAFSGARGNLSQVRQLVGMRGLMSDPNGEIMDL
PIKQNFREGLTITDYLMSGYGARKGIVDTALKTANSGYLTRRLIDVAQDIIIREKDCLT
NRSILFILGRILNKTIYLVEKETCITPTIIKLLKYIKKILVRSPLTCELNRSVCQKCYGWNL
ANENLVDLGEAIGIIAGQSIGEPGTQLTMRTFHTGGIFTGSNQQIISSVDGVLEFSEFL
KPLEFRTKLGDNVLKTENSGSLIIT--
LKIEIPAQTLLFSKNGDEIKKGTLLGQVVETNKQVLSQLSGELVLSSLAWILSGKLLSVP
NNIQENYKLLKNSLSRIKLINKNAGTVTAYTFTGGVLVPEETHIINRDILKVSEFVYEIVS
NIFSTIPGIIHIILQEINIKPGELFYPGEVIFDNLEIPQLSSSELIRPVYLYKVLKLFEQHLDF

LDISLIVQNNQFINSSTLLGYLELKNESYNLLKLKTRTKKLLCIRDKDCLSIPKDNSTLN
FINLLHTGKILTINKNNVLIQKGRPYFFKG-
DELFCQNGDLIELNQNIGKLSFEKEITGDIVQGLPRVEQILEARKLKDKEKEKPKLDHS
LTINLHALLHLYFIHYEAAYRSIKKVQGVILNLIQSVYQSQGVTISDKHLEVIIKQMTTK
VKVAHEGDTPLLANELVDLQQMKYINESIQTDFYRPVLLGITRASLNTDSFISAASFQE
TTRVLTKAAIEGKVDWLRGLKENVIIGRLIPAGTGMFITTLPDFIEIQKTSFCWFISHGL
TEELSNFSSILDFGNIEVCIFANEYKLKKPKYSEFESKQRDSTYSARIYIPIEIRDRQITNR
KKVFIGTLPLMTNKATFIINGCERVIISQIIRSPGIYF-
YKGTLISNRGSWLKFHIRDIEIEIDKSTKISAVELLHEMGLTEISQSVKYP--
LYFSVFLKIFDRRYYDLGHVGRLKVNKKLQLNISEKTNTLTYQDIIAILDYLITITNEKD
DIDHLKNRKVRSVGELLQNQFRVGLNRLERTIKERMTIALIATMKEFFGSSQLSQFMD
QTNPLSALTHKRRISGLGPGGLNRDRISFAVRDIHPSHYGRICPIETPEGQNAGLIASL
TTCARVNCYGFIETPFWKVTKGRVIKTGKPIYLTADLEDNFKIAPADINIGILTKQLIPV
RYKQDFITVSPQDVDFIAVSTVQVVSAAAALIPFFEHDDANRALMGSNMQRQSVPLL
LPQKPIVGTGLESQVAIDSGMVITAFRRGIIIDVSSKHIIVKYELQKYNRSNQDTCINHR
PIVWVGEKVESGQVLADGPSTNGGELSLGQNITVAYMPWQGYNFEDAILINERLVYE
DVFTSIHIEKYDIEVRQTKVGAETRDIPNIIQNLDEHGIIYKGAFVKPGDILVGKITPKG
ESDQLPEGKLLRAIFVRDTSLRVPVLDIKIFDRIFLAEIRKIQVGDKIAGRHGNKGIISRIL
PRQDMPYLPDGTPVDVILNPLGVPSRMNVGQLYECLLGLAGHKLNRRFKILPFDEM
YGPDTSRILINKKLRQASKNEAWIFNPYSPGKIVLIDGRNGEPFENPVTVGKAYMLKLV
HLVDDKIHARSTGPYSLVTQQPLGGKSQHGGQRFGEMEVWALEAFGAAHTLQELLT
VKSDDMQGRNDALNAIPKSGIPESFKVLIRELQAIGLDISTYKMGELSNTEVDLMMFD
YIKIKLASPDRIREWGERLLP-
NGIVGEVTKPETINYRTFKPEMDGLFCERIFGPTKNWECHCGKYKICERCGVEVTESR
VRRHRMGHINLISPVTHIWYLKSVPSFIGILL-----GAEFIKNALGNLDLLKESTLARAIV-
-
EKKDRETEIKRVRILENFISTGANPDWMILSVLPVIPPGLRPMVQLEGGRFATSDLNEL
YRRVITRNNRLIRLIEIYAPEIIIRNEKRMLQEAVDALIDNGKRGKKALGANNRPLRSLS
DIIKGKQGRFRQNLLGKRVDYSGRSVIVVGPSLKLNQCGLPYEIAIELFQPFIILITQGA
KFIHQNPLVDNVLKVLVNHPIFLNRAPTLHRLGIQAFEPILVEGRAIKLHPLVCSAFNA
DFDGDQMAVHVPLSLEAQAEAYILMLTPYNFLSPATGEPIIMPSQDMILGCYYLTAN
NVKSLTGSNHYFADFEDAILAYEQNKINLHALIWVRYLLVQYLQTTTGRIIFNSTIQKT
LMSRYRGPKLRITRRLGLPGLTQKTTTRTNTKKTEYGIRLEEKQKLKFNYGLTESQLF
QYIKEARRRKGVTGLILFQLLEMRLDTICFSLGFASTIAGARQIVNHGHVTVNGKLVNI
ASFQCKPNDIIGIKNKTSSKNLIQTNLIPSHLMFNETKFEGKVNDYCRREDILLDLNEL
LVIEYYSRMGQKTHPLGFRLGITQSHKSAWFANLNQYANLLQEDDKIRTYFKPVISDI

KINRNDQIELSIETARPILVLSNLKKSLRQIRINILEVENADLNASFIADYVVEQLEKRIAF
KRAMRKAMQRAVGGIKIQVSGRLNGAEMARSEWIREGRVPLQTLRADIDYASKEANT
IYGILGIKVWLFRMVNDTISDMLTRIRNANMVKHQIVEIPFTKMSNNLAIILKEEGFIED
FETYSRNFILISMKYKGTERLPVISKIKRVSKPGLRIYSNKSSLPNILGNLGIAIISTSQGL
MTNIKARQLGIGGEVLCYIWMLVERLVQISRVSKVTKGGKKLSFRAIVVIGDENGKVG
VGVAKADDVINAFKKAKTDGQKHLISVPLTKTLSIPHNVSGRFGACKIIMRPSIEGSGV
IAGGAVRTVLEVAGVKNVIAKQLGSNNLLNNARAAVDALDNLMSRRNISKKRFPKAD
PIYNSYLVSLLTARILKSGKKNLAQKIVHEAFEIIKSRTKQEPLEIFEQAVKNSSPLVEVK
ARRVGGSTYQVPIEVNGFRATNLALRWIIKYSRERTGRTMAMKLANEIIDASNEVGSTI
KKKEETHRMAEANKAFAHFRRMLRVRLESFNHELLTESCNKIIESVKTLESNTVGPIYL
PTDKRIYCVLRSPHVDKDSREHFEIRIHKRILDIYYDPNIIELLSDLPAGVLYQIMPTIQQ
LVRVKRAQIRKKTKCPALVNCPQRRGVCTRVYTTTPKKPNSAIRKVARVRLTSGFEVT
AYIPGIGHNLQEHSVVLIRGGRVKDLPGVRYHIIRGALDSGGVKDRTQGRSKYGVKKP
KMKKDKRIIPLGVVHIQSTFNNTIVTISDIKGDTLSWASAGSTGFKGARKSTPFAAQTA
AEKAALDAVNLGLKKVEIVVKGQGSGRETAIRSIQGAGLEIISIKDITPVPHNGCRPPKR
RRVMVRLLGVDLPRNKRIEYALTYIHGIGLTTAKKLVADSKISDRRTDDLSTEETVALR
ENLEDLKLEGDLRRFNGLNIKRLNDINCHRGKRHRTSLPVRGQRTRTNARARRGSKR
TKKMPVKEKVGIVISNKMDKTIVVNVESRYSHPIYSKIMVKTKKYLAHDENQECNIGD
QVLVKECRPLSRKKRWTLEMAKKSMIQREIKRIKLNKKYSEKRSRLLVEYSTANTFESKI
EIHNKIQKLPLNSSKIRIRNRCWKTGRPRGYYRDFGVSRHVLREMAHQCLLPGVTKSS
WMSRSIKKGPFVAYHLLKKIDQQKKDTIKTWSRASTILPMMVGHTIAVYNGKQHVPV
FISDQLVGHKLGEFVSTRTFRSHKTDRKAKRMNKSAVKRIKINERNRLVNRTYKGNVK
SIIKVYLDNLKTYVDESLKLAYSRIDKATKRNVFHKNKAARKKSQLANYLKMMTQIFD
ESGNIVPVTVIKVGPCVVTQIKTTLTDGYNAIQIGYGNAKTLTQPQLGHLQKSNIQPL
KYLKEYKVDNEDFNVGQIINAESFNSGEFISISGKTSGKGFQGLQKRYNFARGPMTHG
SKNHRAPGSIGMGTSPGRVLPGKKMAGQLGNKQTSIKKLKIVQVNIDENILVVKGSVP
GKPGNLVSIAMAKNKGSRVLITLECTDCRSNNKRSPGVSRYTTQKNRRNNPDRIELKK
YCPNCNRHTLHKEIKMTQIFDESGNIVPVTVIKVGPCVVTQIKTTLTDGYNAIQIGYG
NAKTLTQPQLGHLQKSNIQPLKYLKEYKVDNEDFNVGQIINAESFNSGEFISISGKTSG
KGFQGLQKRYNFARGPMTHGSKNHRAPGSIGMGTSPGRVLPGKKMAGQLGNKQTS
IKKLKIVQVNIDENILVVKGSVPGKPGNLVSIAMQKFVTYSVLTDGQVLELKVLNSGN
YLIHKDITRHKVLAKQGTGSSKTRSEVRGGGRKPWRQKGTGRARAGSNRSPLWKGG
GVIFGPKPKVATFKLNQKERKLALQTLLYNNRNYISFVENLFIIKTKGLVEILFSLHEKT
LIIVSKKTPALLLSARNIKNVELISASTLNTLSLIKSKNILLTVSALNDIKEIYMLKLRLKR
VGRKRQPAYRLVVMTNDTRRDGRPVDQVGYYNPITKESKFDNEKIIGWLNQGVQPT
ETVENLLKKIIMIIKYPIITDKATRLLENNKYSFIVDPKSDKLTIKHSIEYLFDVKVIKVNT

CQLPKKKRRKSHYKKAIVTLSEGNTINLFAMDLQKSDLPTLEVGDGVKIGVKIIEGTK
ERIQFYEGTIIAKKNSSVNTTITVRKILQGIGVERVFLIHSPKVNSIQVLRSSKVRRSKLYY
LRNLRGKASRLKQRF
>Uncultured_Pelagomonas_JX297813
MLRTDYPKLIKIKVNRGLGLAAQNTAVLNKTINEFRVITGQQPVVTVARNSVATFKIR
EEMPLGVTVTLRGDKMYSFLDRFINIALPRSRDFQGLNPQGFDKFGNYTVGLTEQLL
FPEISYDSVDQARGFNITFVTNAKTREAGAMLLREFGLPIMVRIKRGNVARKRRKTVL
KLAKGYKGAHSRLFRVSNQQVMKARRYSYVSRKLFKRVQRRNWISCINSQIRRTTYSQ
VCSKFKQNKIALNRKMLSEIIINDPLTFDYLVMVIKLYKPMTPGTRTRSIIDYSDLSKVK
PEKSLLRPKPKNAGRNNRGVITVRHRGGGHKQKYRKIDFLRNKFNVAGKVATIEYDP
NRNTKIALIHYSDGDKRYILCPEGLQIGAEVMSGPDVPIQVGNALPLSMIPLATEIHNI
ELNIFQGGKIVRAAGAYATLLAKVGDYVTLRLPSKEVRLVHKNCYATIGRLSNADAYG
VRLGKAGRTRWMGRRPVVRGVVMNPCDHPHGGGEGRSPIGRTKPYTPWGKPALGI
KTRAKCKSLIIRRMSRIGKLPITLPKGVTLAVDDNLVSIKGPLGELNRQITPEVHVEDQ
VFVLKRTRALHGLTRTLLNNMVLGVHKKFEIDLELKGVGYRCQAAKDKVTLSLGFSH
PIILPLPKDVEVTVEANTNIKVAGINKEEVGFIASKIRSFRPPEPYNGKGVLYKGEQVLR
KAGKTGKITQIKTADQLGYAQVSSKILNIGHLEKVGVPLLRHEYKVSDTNELTLGQNL
IFEAGNLVDVSIGAGLQKRHFGRPGGT-------TPGRVYPG--
KKMAGQLKQVTIKKLKIVKGAV-
PGKPGNLISIMAKKIVAIIKLALPAGKATPAPPVGPALGQHGLNIMNFCKEYNARTAD
KGDLIIPVEISAFEDRSFSFILKTPPASVLLAKAANIQKGSGEPNTQKVGSITTAQLEEIA
KTKLPDLNAKKLSSAMKIVEGTARNMGITIMLSPKRTKFRKQQRGRMKGRAMRNNK
VAFGEYGLQVQECGWLTSRQLEATRRTMTRYTKRGGKIWIKIFPDKSITARAAESRM
GSGKGAPEYWVAVVKPGNVIFEISGVKPEVARQAMNLAAYKLPLKTKFISKMIQPQTQ
LTIADNTGARKIMCIRVLGGNKNYASVGDIIIGVVKESAPRMPVKRSDIVRAVVVRT----
----
MYAVIEIGGKQMLVEEGKYYAINRLPHGSSLNINRILFCNRRVVGHPYLEVVKATILEH
FKDKKITVFKMKPKKKLRWTRGHRQLQTRIMIMAQAVARYIRMSPTKVRRVLNQIRG
CTYEEALIILEFLPYRACEPVWQVVQSAAANVQSKHGLTKKDLTIREVFVSPGPVLKRF
RPRAQGRAFAIRKPTCHITAVVMPKKDIHPEWYDNTEVYCDGKLVYTVSSTKEKLNV
DIWSGNHPFYTGSKKLLDTEGRVDRFMKKYNLMAHKKGAGSTKNGRDSNAKRLGV
KCCGGQVVKAGNILMRQRSFKILPGINVGSGRDNTLYALADGVVKFELNIVMKVRPS
VKKMCPKCRVIRRFGVIRVICSNPKHKQRQG---
MFEKVESCSDTSRFRLQGLAQGQGLTIGNTLRRVLLTELEGTAITAVQIKGINNEFATI
PGVREDVLEILLNLKQIKFQGLLTSIVLKGPQLVTADHLVLPPTLNILNPNHYIATISE
QTNLELELKIESGTGYLVLFLNIDVLFTPVKNVNYEIRDITEILDLEITTDGSITPLEALN

KSSQILQALFGGLILIEELQLSVRSYNCLKRVNILTIADLVQYSVLKEIFGQKSANEVVKK
LQDYNKTINKKELKSIVHSAFQSYGIVKATNLAECLKKEGFSFATQAGISISVEDLKVPP
TKNSLFLKNNKQINLAYFYEKRGNINEVERFQKVIDTWHTTSEILKNQLVDFFKSTDP
LNPVYMMAFSGARGNLSQVRQLVGMRGLMSDPNGQIIDLPIKANFREGLSITDYVISS
YGARKGIVDTALKTADSGYLTRRLVDVAQHVIIRELDCETKNGVRVYFGRVLAKSVYI
LERNTPLTLSKLRKINF-
TPLILRSPLICEASNSICQKCYGWNLSQGKLVELADAVGVIAAQSIGEPGTQLTMRTFH
TGGVFTESSNQIYSAIDGQILFSSTLKTSTSRTIYGEIILKAINSSEFFIFTLKKYIIKPEMLL
FVNNKSFVKKGDVLAELPLTNKRIFTPISGEVQFTNLIWIAAGEIYDILSKKKK-
VKVVKNAIAQTKIISPIGGIVQLFLLGSSVLCPLSGNSKALK--------------------------
KNINIESFKILYPGEILFNKIEISHLSYCQIILPLKQGIIFSETILNLNSNVISYLIRPNQIIEPY
SIVATSNIITELINIKNFKNSLNRFLVSSSKDYKKLSFPVQKFDDSLFDYSGYIIPQKSSDSI
IRISAPFFVSQGTRILTRHGSLIKKGESLCQLVYTRVISDDIVTGLPRIEQLLEGRLEKIN
DETIEKVAQPLDVHPHNVLQVYFHYYKATKRSITAIQILLLNLVQDVYRSQGIYISDKH
VEIIVRQITSKVKIKVNNETTFTVGEVVEFDQIVYINRALRSTEYEPVLLGITKVSLMTES
FISAASFQETTRILTQAAVEGKVEWLRGLKENVILGRLIPAGTGMFVTTIPHFSETNLN
SFCWFLEYGLSDELRNFASALNLEKLNIRVYSEEFVLKRPKYSSVQCKRYDLTYAIRIYV
PIELIEEG--NEYLAFFGEIPLMTEEGTFIINGCERVIINQIIRSPGIYY-
YSATLISDRGSWLKFEFETVQIRLNKATTVDFYHLLETCGITDLNKSIQQTSISDFDNY
LQIFNEKVYSLGKIGRKKLNSRLGLSIPDSITRVTIDDVIAISNYLVDLHANLDDIDNLR
NRRVRSIGELLQIQYGIGLSRLERSINERLMIAIIASIKEFFGSSQLSQFMDQTNPIASLTH
KRRISSLGPGGLNRDRLSLAVRDIHPSHYGRICPVETPEGQNAGIIASLACYARINESGF
LETPFFKMKQGQVLDNQLPIYLTTEEEEVIETAPADRVLSSLNEQLVPIRYKNEFSLVPI
NQVKLVSVSPLQNFSVATSLIPFLEHNDANRALMGSNMQRQSVPLLYPHKPIVGTGL
EHQLATDSGAVLINKFDATVKYVSSRLISIEYKLQKYARSNQDTCINQRPLVWPNENV
TSGQIIADGPGTNGGELALGQNLLVAYMPWEGYNYEDAILVNERLVQEDLFTSIHIEK
FDLEIRQTTFGVETRDLPNVVSNLDSNGIVCKGTFVKFGDILVGKVTPKDETDQLPE
GRLLRAIFVSDNSLRVPVINVRVFRRIFIAQIRKIQVGDKIAGRHGNKGIVSRILPRQDM
PFLPNGMPVDLLLNPLGVPSRMNVGQIFECLLGLAGSKLNRRFKITPFDEMYGTETS
RNLVNGYLQKAATNEAWLYNSSLPGRIVLRDGRTGKLFDNPVLVGKTYMLKLIHQV
DDKMHARSTGPYSLITQQPLGGKSRHGGQRFGEMEVWALQAFGCAYTLQELLTLKS
DDMEGRNEVLNAIPKPGIPESFKVLIRELHSLGLDISLYKMDKVSETEVDLLMFDYIKI
NLASPERIKQWTSDTVR-
STGSGEVTKSETINYRTFKPEMGGLFCERIFGPVKSWECHCGKYKVCERCGVEVTESR
VRRHRMGYIKLEYPVAHIWYVRSFPSYISLLLFRHFDGAELISDLLENLDLEKVVNSCRT
SYTTASSDKEKLIKRIRILENFIATKSDPTWMLLSIIPVLPPGLRPMVQLEGGRFATSDL

NELYRRVINRNNRLRRFFEIYAPEIIIRNEKRMLQEAVDALIDNGHREKKAVGLNNRPL
KSLSDILSGKQGRFRQNLLGKRVDYSGRSVIIVGPELKLNQCGLPYEIACELFQPFLILI
QLGAKLIQEKERIWEALNVLEGFPIFLNRAPTLHRLGIQAFEPIIVDGRAIRLHPLVCPA
FNADFDGDQMAIHVPLSIESQAESYLLMLGPNNFMSPATGEPILLPSQDMVLGSHYL
TSQSRSNFKGSYQYFGNVESVLQAYNCDKIALHSSIWLRFTLVTYILTTPGRVLFNNLI
YSVLMSRYRGPKIRIVRRLGLPGLTNKTTTREKQNNSSYAVRLQEKQKLRYHYGLSEK
QLLSYVKEARRLKGSTGSVLLQLLEMRLDNIVYRLGIGNTIPASRQIVNHGHIYVNGKK
VTIPSFQCLPNDVIEVRDKKVSKELANQFLLPEYLEFEKETLKGKVKRIAENTETNLKI
NELLVVEYYSRMGQKVHPLGFRLNTTQKHKSIWFAQLNQYSALLEQDEIIRNYIKTNI
ADIEIERTNNIFLKIYVAKPLIILTKLKALLNKISIDVFEIMEPDTHAILLAQFIANQLVRR
VAFKRAVRKAIERAAQGIKVQVGGRLNGAEIARTEWVKEGRMPLQTLRAKIDYATASA
KTIYGILGVKVWLFKMVNDVISDMLTRIRNASKVKHHLVQVPFTKMTLAIAQVLKEE
NFIEDFQEFGRKFLLILLKYKGKGREPAIMTLKRISKPGLRVYANVKELPVVLGNFGVA
LMSTSKGVMTNFKAAKLGIGGEVLCYIWMWVRKVVKVRRITKVVKGGKKLRFGALVI
IGNEKGLVGVGVGKADDVVEAVKKAANDGRRNIIMVPLTKTFSIPHVSTGQYGASNV
LLKPASAGSGVIAGGSVRTLLEAAGVQNIMAKQLGSNNLLNNARATICALEALMSRRK
IIKKRFPKPDPVYNNYLVSILVSRLIKDGKKTLAQNIVCDALDIIAYKTESDPIEVFEQAV
KNVTPLVEVKARRFGGATYQVPMEVTSFRGTNLALRWIVKASRSRTGKTIALKLAAEL
MDASKNSGDAMRKKQDTHKMAEANKAFAHFRRMTRVKLEAFDTKLLTEACKSLIEG
ISSINGIAKGPIPLPTKKRIYCVLRSPHVDKKSREHFEIRIHKRLLDIYTPQNIADSFLAIP
PGVSIKLMPTIQQLIRNKRQSLKKKTKSPALKNCPQRRGVCTRVYTTTPKKPNSATRK
VARVRLTSGFEVTAYIPGIGHELQEHASVLIRGGRVKDLPGVRYHIIRGALDSGAVKNR
TQGRSKYGVKKPNMKKQKKNIVSGVVHINSTFNNTIITITDLSGNTISWSSAGSVGFK
GARKGTPFAAQTAAEAASASAMEKGLRKVEVLIRGQGSGRETSVRALEVLGLDIVSVK
DITPIPHNGCRPPKRRRVMVRISGIDLPKSKRIEYALTSVYGIGLTSSRNILSTANVDAK
RTNVLGDTEVMAIREVIENYKVEEDLRRQTKQNIVRLSQINCVKGRRHRQNLPVRGQ
RTRTNSRTRRGARLM--
MVLKQKIGIVVSDKMNKSIVVAIDTRYKHNLYGKIMAKTKRYLVHDPENSCSIGDTVL
VEEHPPISAKKRWIFKMAKKSMVEREKKRQRLILKYREKRRNLLNAIKTSDSFSETLQL
QKKLQKLPLNSAPNRLRNRCWKTGRPRAVYRDFGLSRHVLREMAHDGLLPGVIKSS
W---

MAKTKGVRVIITLECTSCRTNDKRSNGVSRYTTMKNRRNTPDRLEIKKFCPHCNRHT
NHKEIK--

MLKLRLKRGGRKGQPSYRVVVMEARSRRNGRPVENLGYYNPLSKEFLVNSDRVKLRL
NQGVLPTRTVATLLKKVIMEIKQIATTKKALWSLEDKKYTFLASPFVTKEWMKRAIES
CYNVRVIKINTSNLPTKKKRKAKLKKVIVTLDPNDEIPLFS-------------------------------
----
>Undaria_pinnatifida_KP298002
MLKFLYPKLVKIQLNRGLGVDGQNNKTLQKSVDEMRLITGQQPILTKAKKSIAGFKV
REEMVLGITVTLRSKKMYAFLEKLIHLVLPRIRDFRGLSLKGFDRNGNYNFGLKEQLV
FPEISYENVDQIKGFNISIVTTAQTKIEGIALLKEFGFPLMVRVKRGNVARNRRNKILKL
AKGFCGAHSSLFRVANQQVIKSLIYAYRGRKEKKRIFRQLWITRINAAVSNLKYSKFIHN
LKRSKILLNRKMLSQLAILDPSAFSSIVMAIRICKVYTVGTRNTIFSSFDEITKTKPEKKLI
GRNHRKKGRNNQGLITTRHHGGGHKRRYRMIDFKRKKHDILGNVFAIEYDPNRNAR
IALIYYENGDKTYIICPDSLKIGSKIMSGPNAPVEIGNSLPLENIPLGTSIHNIELSYNKGG
QIVRAAGTSARILAKENNYVTVRLPSKEIRIVHKSCYATIGTVSNRDSNNVKLGKAGRK
RWLGIRPTVRGSVMNPCDHPHGGGEGRATIGRPKAYTPWGKAALGVKTRKKDKSYI
VRSMGRIGKLPIKVPSNVQVEINQNNIEVSGPHGKLFRKISDVISVDNIIYVKRIANQLY
GLSRTLINNMVVGVSQKFERTLELQGVGYRAQLKGKDLVLNLGYSHPILIAIPLGIEIK
VEKNVGIIITGFDNELVGQLAATIRSKRPPEPYKGKGILYKGEIIKRKVGKSGKMTFIPS
KLKQQWYIIDAKDKCLGRLATEVSNLLRGKNKSIFTPSQDVGDYIIIVNASQINVKRVQ
KLYFRHSGRPGESFEDLQIRIPERILEKAIKGMLPKNGRKLFTKLKVYKGEEHPHMSQK
PQKIMAKKVTSIIKLALSAGKAVPAPPVGPALSQHGVNISAFCKEYNAKTADQIGLIIPV
EISVYEDRSYTFILKTPPASVLLVKATNIKKGASEPNRQIVGSITADEIRKIAEIKLPDLNT
KSLDSAVKIIGGTAKNMGITIMLSPKKTKFRKQHRGRLKGKASRGNKISFGDYAIQALE
PTWLTSRQIEATRRTITRYTKRGGKLWITVFPDKPVTARAAESRMGSGKGSVDYWVA
VVKPGTILFELSGVPLKLAKEAMIIASYKLPIKTKFLINMIQPQTYLTVADNTGAKKIMC
IRVLGGRRKYAKLGDIIIGVVKEATPNMLTKRSDIVKAVVIRTKKAASRKDGTSIRFDD
NAAVIINMDKNPKGTRIFGPIAREIRDKDFTKIVSLAPEVIMYAIIEASGRQFWVEPKKFI
EFNRLILGSTILIRRVLFVKNTLIGQPYLNVVKGVVSKHFLGPKILVFKMKPKKKYRKKI
GHRQKLTRLLIMAKAVSKYIRISSHKVRRVLDQIRGRSYLEALMLLQFMPYRACGPIW
QVLNSAAANAENNNGLNKEDLIVKTVFADQGPVLRRFRPRAQGRGYQIRKPTCHITII
LMPKKNIHPKWFNDTKVYYDGQLIMIVGSTKPELHVDIWSGNHPFYTGSQRIIDTEG
RVERFLKKYKIMAHKKGAGSTKNGRDSKSKRLGVKCFQGHPVEAGNILIRQRGLSFKP
GDNVGIGKDYTLYALTAGLVSFKISVSMKVRPSVKKMCEKCRIIRRHGRVQVICTNLK
HKQRQGMAVPKKRRSKAKGRTRLSNWKSKGRVAADKALNLAKSIVRST-

FLFMCKCVDLDLNYGHFVFTSLEQSEGTTIGNMLRRALLSNLYGFRITGVRVAGINNE
FTPLEGVREDLLEIILNLKEIIIYDQYGLLKAQGPAIITAGAITLPNGIKVLNPSTHLLTIS
DESIIELSIKIEYGKSYILAFIPIDSNFAPVLKVNSYIKPTNEELHLEIFTNGTLLPHQALIQ
ARNMIGYMLSTIGSLKGLGLSNRIIKALNKVNINFTWDLMEYPSLISILGPKSVEEIKNK
LTLSNNIINKKELKKIIEWAFKNYGQRKAVYFVDQLKEIGFEYATKSGISISIEDLKISPAK
SALMEIASNDVFLAESQANNGEITEVERFQRIIYIWNSTSEELKDRLIEFFKKNDPLNSV
YIMAFSGARGNIAQVRQLVGMRGLMSDPNGKIIDRAIGANFREGLSITDYIISSYGARK
GLVDTAIKTADSGYLTRRLVEVAQSIIISELDCKTERGIFLLNGRILALTIFLALRNQEIT
PSVIEELVFINKVLIRSPLTCECRRAVCQQCYGWNLALGTLVELGETVGLIAAQSIGEP
GTQLTMRTFHTGGIFTELIRQGRAQCSGYINFLPELKISPYRTNYGQDAVVSENQSWL
AIIEIVKVRVEARTIILVNNNNYIKRNQVLFEAAPKIKEICAKEPGEIFLKKIFWVLSGQVF
SIANRVRKSEKIYKQAIAQSKMVTTIGGFTTKCKVYGGIFIPEALVQTTSNDFRFGDYVI
EIFPNYITNIEGFIDFRIKYISIKPGEVYYPGELVLDKFEVKVLSYIEYIRPITRYEFIQFVDS
PLTIISLNLFVEEKQFIDPYTVVGSFDTIFFDNFVYNVKIKKSNILLTTKSDYEEIFLDSN
QFKLFLIKNSGLMEQVSGNKITLHLGQPYFFSTGALIRKIPGDYIKGQENFGQLVYERL
KTGDIVQGLPKIDNILEARKPKYTSNLSTLVGQPLTLNPHTLLHVYFRYFESAYRSLKK
LQTLLLTSVQAIYISQGVIISGKHVELIVREMTHKVYIEYPGKTNFLPGDIIDLDQAQYI
NLCIKNNGFRPILLGITKSSLKTDGFLAAASFQETTRVLTRAAIQGKTDWLRGLKENAI
TGRLIPAGTGMFPIILPDLSEVQRISFCWFLTEGLPEELTNCPSILNKSGIELIIYGQEYKI
YYPSFAILNALKKKGNYNLRVYVLMSLKKNEFSSKERVFFGEIPLMTEKGTFIFNGCER
VIVSQIIRSPGIYYFYEGTIISKHGSWLTFELEYIWVKFDKQYKIPINWFLVGFSLEEIYQT
VQNYEFLRKRELERLLNKSLYDLGEVGRIKLNKKLGISLPLNIRILTSLDILKAIDKLIHIS
SYNDDIDNLENRRVRSVGELLQLQVRVALNRLRKKITTDEKLAVLSVIREFFGLSPLSQ
YFDEINALAQLTHKRRISSLGPGGLNSEHVSFAARDIHPTQYGRLCPIETPEGQKAGLI
ASLATHAKINNYGFITTPFFRVYKGKVLRELGPIYLTAEQETIYRVASGDVLLEFFQTT
TVPVRFYNEFIIVDSVSVDFIAVSPIQIIAIGASLIPFLEHDDANRALMGSNMQRQAVPL
LYPKKPIIGTGIEHQIAADSQVVIINLLNGVVDSVSADRIIILFFLDKYRRSNQETIINQKP
LIWTGEIVKPGQIIADGPGTDGGELALGQNLTVAYMPWEGYNYEDAILVSERLVQED
LFTSIHIEKYELQLVDDSASIETTSIPNIICNLDTNGIIKKGTFVNPGDILVGKITPIVEDE
ELPESKLLRAIFPEDTSLRVPVIEIQTASRISIAQIKKIRIGDKISGRHGNKGVISKIIPIHD
MPFLPDGTVVDVILNPLGVPSRMNVGQIFECLLGFAGDYLNRRFKVVPFDEMYRPNA
SRILINKTLKKAATNKPWLFNKSSPGKVLLTDGRTGEIFDNSVLVGKPYILKLIHLVDK
KMHARSTGSYSLVTQQPLGGKSKHGGQRFGEMEVWALEAFGVAYTLQELLTIKSDDI
NGRHEVFNAIPEPGVPESFKVLLRELNALGLDITTHQIGKLNSYKVNLLMFEYVKINLA
SPERIKEWAEKILP-
NGIVGEVTEPETINYRTHKPEKGGLFCEKIFGPVKNCECTCGRYKICEICGVELTESRV

RRHRMGYINLLSPVVHIWYLKGSPSFLSTILVSKYDGAEILYDRLKRIDLKVEIASNRNI
MFCSNKAVEDAIKRIRIFENFLTTNTRPEWMVLHFLPVLPPGIRPMVKLDNGRFATSD
LNELYRKIIIRNKRLKRLLSVHAPSVVILTEKRMIQEAVDTLIDNGKRGSKVLDANKRPL
KSLADIIEGKQGRFRQNLLGKRVDYSGRSVIIVGPQLELNQCGLPYEMAIELFLPFIILLS
QGAKIIYSNSFIWYLTEILRKHPIILNRAPTLHRLGVQAFDPVLVRGRAIQLHPLVCPAF
NADFDGDQMAVHIPLSFESQLETRLCLLAPNNFLSPSTGEPNIQPSQDMVLGFYYLT
AQNRMGLKGSNNYFSNFNEVITAYEQKQLQLHSSIWVRCLLVQYIRTTPGRIIFNQCI
NNILMVRYRGPRLKIIKRFGLPGLTRKVVLKKKTPKSAYKIRLNEKQKLRYNYGITESQ
LLRYVKEARRRKGLTGFLLMQLLEMRLDNIIFRLGLAPTIPAARQIVNHGHVLINKKR
VNIPSFQCRPNDSISFSTKPKTVALLKSNLLNSHLEFNQKLLTGKILNLVNRKDLRFKIN
DMLVIEYYSRMGHKTHPLGFRLGIVQEDRSLWYSGTNSYISFLKEDYTIREYISQFITKL
IIKRNKRLYIEIQSVVPRIVLDELSALLKKVLINIVEITKPYTEASILVDVLVKKLEERVSFR
KCIREILRRYTKGIKVQIAGRLNGAEIARTEWVREGRVPLQTLRANIDYSYKVAQTTYG
ILGIKIWLFKMTTDIIADTLTRIRNANLVRHQIVRVIKTKVTYSVVKILKEEGYISNFEEI
NRKYLLLCLKYHSQKRQPIITGIKRISKPGLRIYVNKSNLPNVLSNLGIAILSTSKGILTN
NKAKKLGVGGEVICYIWMWEQRVVKISRVSKVVKGGKKISFRATVVVGDMEERVGVG
VGKAEEVSTAIKKAETDAKKNVLTIPIAEGKTIPHAMVGIAGGSKVFIRPAVPGTGVIA
GGSVRIVLELAGIKNILSKQLGSNNPLNNARATIVALKSLMSRRTNKKRKFPIADSVYD
SFLVNLMISKILKSGKKTVAQKIIYEAFDIIKERTNSQPLKIFEKAVKNVSPAVKIKAKRV
GGSTYQVPIEVKKFRGINLGLCWIIQFARARSGKTIAIKLANEIIDASKGSGNSIRKKDET
HRMARSNKAFAHFRRMIRIILQSFNHLVLNECCKKILDALANEKLNVAGPISFPTKKRS
YCVLRSPHVNKDSREQFEIRRYKKIIDIHSDSEIVNTLLDLSPGVSTELMPTIQQLIRVR
RKKIQPRTKSPALKSCPQRRGVCTRVHTITPKKPNSAIRKVARVRLTSGFEVTAYIPGIG
HNLQEHSVVLLRGGRVKDLPGVRYHIIRGTLDTIGVKDRRQGRSKYGMKKPVMKKM
KRTIVTGTMHVHATFNNTIVTVSDLNGNTLSWASSGTVDFKGSRKGTPFASQKAAEK
AALIAKSYGLKRLEVVIKGSGPGREPAIRAVYQKGIRIVSIKDVTFIPYNGCRPPKRRRV
MVRFLGIDLANNKKIKYALTGIYGIGLSRAEEILDSAGLKGVRTKDLSDEDISNLRKVLE
NYQLEADLFRLTNLNIKRLMENGSIKGRRHRAGLPVRGQRTRTNARTRRGGKKTKN
MVKLQRLGIVISNKMQKSVVVAVEYRYKHKFYSKIIVRTKRYLAHDAEDICNIGDEILL
EESRPLSKKKRWIVKMAKQSMIEREKKRERLVIKYREKRKSLLLEFKKANTFSDQMEVH
KKIEKLPRNSSRIRLRNRCWRTGRSRGVYRDFGLCRHMVREMGHNCLLPGVRKASW
MARSFRKGPFIAYHLLQKIEKMKKTSIKTWSRSSMIIPAMIGHTISVYNGKKHIPLFISD
QIIGHKLGEFIPTRNFRSHKSDRRTKKMTKSSKKRIKINKRNRIQNNSYKSLIKSYEKKH
LSLIKTSLRASFSSVVSQIDKAVKKNIIHKNTGIRKKTNLFRKTFPMTQVFDKDGLALP
VTVISIGSCFITQIKTEKLNGYNAVQIGHSKTVNLKKAELGHLKKNGLPPLSDLCEYKV
MDENYSLGQVLNVNHFEIGQFVNVTGKSIGKGFSGNQKRHKFKRGPMTHGSKNHRA

PGSIGPGTTPGRVFPGKLMAGQLGAKKITVSKLEILGIDSKQNLLILKGNVPGKPGNL
LNIVMAKNKGARIIITLRCTNCKKTNKKYKKKIDYTTTKNRRNTPDRLELKKFCPNCN
SHTQQKEIKMTQVFDKDGLALPVTVISIGSCFITQIKTEKLNGYNAVQIGHSKTVNLK
KAELGHLKKNGLPPLSDLCEYKVMDENYSLGQVLNVNHFEIGQFVNVTGKSIGKGFS
GNQKRHKFKRGPMTHGSKNHRAPGSIGPGTTPGRVFPGKLMAGQLGAKKITVSKLE
ILGIDSKQNLLILKGNVPGKPGNLLNIVMQKILTFPVKLTGEELKLKEANTINYVIQRA
YLAQRSNERQGTANTLTRAEVKGGGRKPWRQKGTGRARAGSNRSPLWKGGGVSFG
PKPKFYSSKINRKEWQLSLRSLLIAKQKDITIVDNFFLYKTSNIIKVLINLFENTLIVVPKI
DEKLLKSTRNIKTIKLIVANNLNLKQILLAKNLLITKDSLKTIEETYMLKIRFKRGGRKK
QPFYRIVVMDARTKRDGKVLEELGFYNPMDKTFHINRERTILRLANGVQPTEVVKNL
LRKGFMLIKYPVTTIKTNLLLENNQYTFLVDPKLNKVSIKKAIEFLFDVSVIKVNSCNLP
KKKRRRPRYKKVIVKLAKDNKIDLFSMSVEKKNLSKIFVGDTVKIGVFIKEGNKERIQYY
QGIILGKNNSGINLTISVRKVFQGIGVERNFLIHSPKFESIEVIKSSRVRRSKLYYLRSIVG
KGSRLKQRF
>Vaucheria_litorea_EU912438
MINLMYPKLIKIQINRGLGIDAQNNSLLKKSIEEFRTITGQQPIVTKAKKSIATFKVRET
MNLGVTVTLRGNKMYSFLEKLIHLVLPRIRDFRGLSIKGFDKNGNFNFGIREQLVFPEI
NYDIINQTRGLNITIVSTAKTKDEGIALLKEFGFPLMVRIKRGNVAKKRRKKILNLAKG
YQGAHSRLFRLANQQVMKSLRYSYMNRKQKKRKFRQLWITRINSSTRFTKYSTFIYNL
KQSKILLNRKMLSQLSIIDPTAFTNLIMLIQVCKANTPGTRNSVLLKFDEITKNKPEKT
LIKKLHRKKGRNNNGRITIRHRGGGHKRLYRTIDFKRNKLNIKGKIHSIEYDPNRNTKI
ALVHYEDGEKRYILCPDKLIVGSYIESGENIKSQIGNSLPLAYIPLGTFIHNIELSPNKGG
QIVRAAGSFARILAKDDKYITLRLPSKEIRLIQSSCYATIGIISNRDAANVVLGKAGRKRW
LGIRPTVRGSVMNPCDHPHGGGEGRAPIGRVKPYTPWGKSALGTKTRKKNKSFILRS
MSRIGKLPINIPNTVNVKIENQIINVMGPHGQLKKKISDFLIINNEIIVLRQARSLHGLYR
TLINNMIIGVSNKFEQRLEIKGVGYRSQVQDTTLILNLGFSHPIQLQIPVDIEIVIEANV
NILIRGINKETVGQFAATIRNKRPPEPYKGKGILYKEEIIKRKVGKSGKMTFISSNTQSQ
WFIFDANEECLGRLATKVAIILQGKNKNFYNPAQNIGDFVIIINAEKIKVKNLQKNYMR
HSGRPGENFEQLHIRLPERILEKAIKGMLPKNGRRLFTKLKVYAGESHPHKAQKPQMI
MAKKITAIIKLALPAGKAMPSPPVGPALSQHGVNIASFCKEYNAKTADKGDLIIPVEISV
YQDRSYTFILKTPPASILLSKAANIKKGSSEPNKQIVASISRIKLEEIAQIKLPDLNTMKLT
SAIKIIEGTAQNMGIGIMLSPKRTKYRKEHRGRLKGKASRGNKLNFGDYGLQALEPIWI
TSRQIEATRRTITRYTKRGGKLWITIFPNKPVTARAAESRMGSGKGSVDYWVAVVKPG
TILFELSGVSPLIAKEALKIASSKLPIKVKIIIKMIQSQSYLTVADNTGAKKIMCICVLGGN
RQYAKVGDIIIGVVKEAIPNMPIKRSDIIRAVVVRTKKAIKRIDGTLIQFDDNAAVIINLE
KNPRGTRIFGPIAREIRDKEFSKIVSLAPEVLMYGIIEASSRQYWIEPGKIIDINNLPIGSTI

LLNQILFARQIFIGRPYLNIVKATIEKHFLGPKIIVYKMKPKKKYKRKNGHRQHLTRLMI
MSKAIAKYIRISPTKVRRILNQIRGRSYQEALMILEFLPYKACDPIWKVLNSAAANAQNN
FNIKKQNLFVKIIFADQGPTLRRMRPRAQGRGYKILKPTCHITVILMAKKNIHPQWFS
NTKVFCDGQLIMIVGSTKSELNVDIWSGNHPFYTGSQRIIDAEGRVERFLKKYEFMAH
KKGAGSTKNGRDSNAKRLGVKCYQDHKVKAGNIIVRQRGLKYKPGKNIGIGKDFTLY
SLINGLVKFKIYILMKVRPSVKKICTKCRIIKRKGHVRIICVNSKHKQRQGMAVPKKRVS
KSRSKARKAVWIRQAKQASQKSLSLAKSILKQTSFIYMLEFINL-
LVFGKYIIKPLNLGMGVSIGNLFRRILLTQMPRTNITGVRFAGITNEFSTIKGVREDVLE
ILLNLKQIIFKGFYVRIKITGPTVITSSCLQLPDEITLVNPSQYIATITNDSTLEFELKIESE
KKSNLAFLEVDKITSPIRQILFDVYKTQEELVLQIWTDGSITPIDALITSVKLVQNLFTQ
IL---
EYNRTIDKKQIKQIMSWAFNDFGTMKASYLADKIKDIGFKYSTKAGLSINIEDLKVPPI
KKHVIKYANRKVIDAEIEVERGKITEVERFQKVIKTWNNTSETLKDQVISYFRKTDPLN
SIYIMAFSGARGNISQVRQLIGMRGLMADPNGQIINLPIITNFREGLTITDYIISSYGARK
GLVDTALRTADSGYLTRRLIDVAQDMITREKNCNTKNGINLIVGRVLAFPIDIANSDQ
AITPKLAQQISLLKRVIVRSPLTCSSSRSICQQCYGWNLAYGNLIDLGEAVGIIAAQSIGE
PGTQLTMRTFHTGGVFTEPSRQIRAQYSGKILFSKNLKTRITKTQYGEMSSISENEANL
ILIVRTKVTIFPNTLIFIRNKSFVKKNEIILEVISNTTKIYAKHSGEIFLKTLLWILSGELYTI
PQEVKVNSFISKRSLAQSKVINIREGHINQCIIYGGIFIPEATYKIDKNPLYVGEYIQELFK
NNFVNISGIVEIILNEILIKPGQIMYPGEILTNEIIIKRLSYTEGIYPIIRYEIIQIIKQKIYAM
NIKFLVQNKQFIEPYTNLISFTIQIQRKNLINIKEQQRKILLISTDDYKTIYIEQNKLGKG
CFKNSGFITSIYGNQIKLYKGDPYLFSEGAQIKKRPGDLINKGEKLGQLIYERVRTEDII
QGLPKVEEILEARKPKYKSSEWIAVGHPLNINSHTLLNTYFSYYKSAYRSFRKIQSMLLN
SVQAVYYSQGVFISDKHIEIIIKQMTGKVQIITSGDSPLLSDELVELKQVYYINNCLK--
LFKPIILGITKASLKTNSFISAASFQHTTKILTEAAIRGKTDWLRGLKENVIVGRLIPAGT
GMHITNLPDLLEIQKSSFCWFLSKGLPQELSKFSSIFDLGDLELKIYGNEYKFKKPEFSH
LIAKQKKLTYSIKVYIFIELIDQFILKKQKILLCEIPLMTNKGTFIINGVERIIVNQLVRSPG
LYYIYSATIITNRGAWLTFELKSIWVRINKKEKIPINVFLAGLDLKEIYQTIKNYKFLINRD
LSQILNPKYYDLGEIGRIKINKKLDLNLSLSIRTLTLKDILKIIDSLIEIRLYNDDIDSLINR
RVRCVGELIQLQIRIGLNRLKRNTIEKLSTNVIAVLNEFFGSSQLSQFMDETNPLAELT
HKRRISALGPGGLNTEHVTFSTRDIHPTQYGRLCPVQTPEGKNAGLISSLASYAKINH
YGFIETPFFKVIDAQVFKKIIPVYLSAEQEKLYKIAPADIKLDYIEKYIIPVRYNEEFTLAT
RHEVEFVAISPVQIISIAAALIPFLEHDDANRALMGSNMQRQAVPLLWPQKPVVGTGL
EAQIAIDSTTGILNYHTGIVTSVTANEIIVKYKVEKYRRSNQNTCVNQKAIVWPGQLVN
SGQILADGPATDEGELALGQNLTVAYMPWEGYNYEDAILVSERLVLLDLFTSIHIEKY
EIAIRNTRSGMETSQIPNVIRNLDENGLIRKGSLVEPYDILVGKVTPKDKSDELPEGKL

MKAIFVRDTSLRVPVIDILLLSKVFIAEVRKVKIGDKIAGRHGNKGIISKILPIEDMPYLP
DGKIIDIIFNPLGVPSRMNVGQIFECLLGFAGDNLNKRFKILPFDEMYSSEASRILINNK
LKEAATKKSWLFNEYSPGKVLLKDGRTGENFDNPILVGKTYILKLIHLVDDKLHARSI
GPYSLITQQPLGGKSQKGGQRFGEMEVWALEAFGAAYTLQELLTVKSDDIQGRNNV
LYAIPKPGVPESFKVLLRELRALGLDITTHQIMKLTSKEVDLFMFDYININLASPERIIE
WAEHVLP-
NGVIGQVKSPETLNYKTLKPVMEGLFCERIFGPTKSWECYCGKYKTCERCGVEITEAR
VRRHRMGYINLLTPVTHVWYLKGVPSYLSIILINEFDGAEIILDLLKDINLNEQIKINRDI
ILQYPIKLKQATRRIRILESFLITHSKPEWMILTVLPVIPPGLRPLVQLEGGQFVVSDLN
ELYRKLIYRNDRLANFLSSSSPGIIVRSEKRLVQEAVDALIDNGKRGPKAVDINNRPLKS
LADTIEGKQGRFRLNLLGKRVDYSGRSVIIVGPHLELNQCGLPYEMAVELFYPFIALIN
KGAKIIQKNTFLWKLIEIIQGHPILLNRAPTLHRLGIQAFEPVLIQGRAIQLHPLVCPAF
NADFDGDQMAVHIPLSLEAQTEARILMFAPNNFLSPATGDPIIQPTQDMLLGCYYLT
VDNRPNLKGANHYFSNFDEVLCAYEQNQVDIHSSIWVRCCIVRYIRTTPGRIIFNKNL
NSLLMARYLGPRLKIIRRFGLPGLTSKIITKKKLQKSDYKIRLFEKQKLRYNYGLTERQL
RKYVSEARRKKGSTAFYLMQLLEMRLDTIIFRLGLAPTIPAARQFVNHGHILINKKKINI
PSFQCHPTDIITIKNKKGTKQLVEKSLLPNHLTLNTKLLQAEVKDIVIRKDLSFEVNDL
LIVEYYARMGHKTHPLGFRLGITQQHKSAWYSKLSNYSTLIKEDFAIRKIIYDYVTKIFI
TRNDKINLEIQTAKPVIVLDNLCNLLKKTLINIIEIKDIFNYADLLADMLVLQLENRIPF
RRAMKRILEYVKKGIKIQIAGRLNGAEIARTEWLREGQVPLQTLRADIDYSFKTAQTIY
GIIGIKIWIFKMIKDDISDMLTRLRNANMVRHQIVEIPKTKMTLAIAKILKEEGYILKFEE
FKRNFLLLCLKYNGSKRKPILTTVVRISKPGLRIYVNHKNIPQVLGNLGIAIISTSKGVIT
NHTAKNLGIGGEVICYIWMWEQRVVQISRVVKVVKGGKKLSFRATVIIGNKNSHVGV
GVGKADEVSTAIQKAVNHARKNLIQIPLTKTKTIPHNIIGIDGASKVLIRTAGPGTGVI
AGSSIRIVLELVGVQNILAKQLGGNNLLNNARATIAALKGLMSRRSNSKREFPNPDPIY
NSYLVSLLTVRILKNGKKTLAQKIIQKAFSNIKDRTNEEPLIIFEKAIKNASPIVEVKATR
RRGSTYQVPVEVSGFRATNLALRWLVKFAKERSGRSMAVKLANELIDTSKGLGNTIRK
KEETHRMAEANKAFSHYRRMIRISLYSFNSKILEEACKKIFSIISPIIENIKGPIPLPTRKRIY
CVLRSPHVDKDSREQFEIRRYKRIIDIYPESQTLDTFLELPPGVSFNVMPTIQQLIRSPR
QKIYRRSKSPALKNCPQRRGVCTRVYTTTPKKPNSAIRKVARVRLTSGFEVTAYIPGIG
HNLQEHSVVLIRGGRVKDLPGVRYHIIRGALDSASVKNRLQGRSKYGTKKPKMKKIKR
NIPSGIVHIHSTFNNTIITVTDLKGNSVAWASAGTAGFKGTRKGTSFAAQLAAKKATL
QAIDSGLKKVKVLVKGRGPGRETAIRALQTTGVKIVSIKDITPIPYNGCRPPNRRRVMV
RLIGIDLPKGKYINYALTSIYGIGISRAEEILSKIGLDKLKVEDLSDTNISALRKILEEYQLE
GDLRRLKSLNLKRLMEIGSIKGRRHRVGLPVRGQRTRTNARTRRGTKKTKKMSKTEK
IGIVISNKMNKSIVAEVEYRYRDPIYSKIIVSTKRYMAHDEINECNIGDKVLLEPSRPLSK

KKRWVVKMAKQSIIQRQLRREKFIEKYKKKRRNLLKEFSITKDFNIKISIHKKIEKLPRNS
IKIRSKNRCWKTGRGRGFYRDFGLCRHMIRELAHNGILPGVYKASWMGRSLKKGPFIA
YHLLKKIEQMEKKIIKTWSRASTILPIMIGHTIAVYNGKQHVPIYISEQIIGHKLGEFVIT
RNFRSHKNNRKIKKMNNSAIKRIKTSERKRKENLPYKSLVKTYIKKYFKTLEDLAKIALN
LVFSKIDKAKKKNIFHKNSAARKKSKLMKALFKMTQVFYKEGLAVPVTVIRIGPCLVTK
IKTTTTDGYNAIQIGYCQIRKLTKGQLGHLKKVGLPPLKYLTEYNFKDNEFYVGQILTI
NNFNLGQLVNISGLSIGKGFSGTCKRYNFKRGPMTHGSKSHKAPGSIGPGTTPGRVFP
GKKMPGQHGNKKVTILKLKILGIDTEENLLVVKGSVPGKPGNFLNITMSKNKGSRILV
TLECTNCSINNKVKAGINRYITKKNRKNTPSRIELNKFCPYCNTHTLHKETKMTQVFY
KEGLAVPVTVIRIGPCLVTKIKTTTTDGYNAIQIGYCQIRKLTKGQLGHLKKVGLPPL
KYLTEYNFKDNEFYVGQILTINNFNLGQLVNISGLSIGKGFSGTCKRYNFKRGPMTHG
SKSHKAPGSIGPGTTPGRVFPGKKMPGQHGNKKVTILKLKILGIDTEENLLVVKGSVP
GKPGNFLNITMNKIINFPIKLTNDNLTLNIPNKSNYLLHRALQTQLTNERQGTSNTKT
RAEVQGGGRKPWKQKGTGRARAGSNRSPLWRGGGVIFGPKIKKFSNKINRKEWRLSL
RLLLLNKQKNITIINTILDFKTKNIIQYIIDPLQKLVIIIPNYDENLRRGTKNLKNVQLLK
ANCLNIKDILKAEHIFIDINSLKIIEETYMLKLRLKRCGRKKQPFYKIVVMNANSRRDGF
AIEELGFYNPLNKTFKINKGRTIYRLKQGVQPTATLIDLLKKILMLIKYPLITDKTIQLL
NNNQYTFIVDPKISKNKIKQTLEYIFNIKIIKVNTCQIPRKKRRKPNYKKAIVKLSKNYTI
NLFSMIIEKITIPNLFVGDNIKIGFLIKEGNKERTQFYEGIIISQKNTGINKMITIRKVFRGI
GIERTFLIHSPKLVSIERIKSSKIRRSKLYYLRTLHGKAIRLKQKF
>Vischeria_sp._KX839261
MYKKIYPKLLKIQINRGLGLAAQNKTILQKTIEEIRLITGQQPIVTKAKKSIAGFKTREG
MDLGVTVTLRNDFMYAFLEKLIHLVFPRIRDFRGLSPDSFDEYGNYNLGIREQLVFPEI
DYNMIDQLRGYTISIVTTAKTPEEGRILLQEFGFPFMVRIKRGNVARKHRKKILELAKG
YVGSHSRLFRIANQQVFKALKYSYSGRKNKKRWFRRLWISRINIGARNSTYSDLMYKIK
SNNIGLNRKMLAHLAVVDPKVWSLLV--

MSRIGKQLIRIPSGVNIKLTEKNILVKGPKGEIERSIPSCLSVDNSLQVLISSREMHGLFR
SLVSNMVIGTSKGFTKILELKGVGYKANIDKKALNLALGFSHPVRIEAPPGIELSVENN
TIIKINGIEKEKVGLIAQQIRSIKPPEPYKGKGILYKAEVIQLKVGKSSKMTYIPSGKERS
WFIVDATDQTLGRFSTQIANLLQGKHQTTYTPGFDNKVHVIVINAEKIRFKLTQKLYY
THTGKPGRSLEVLFSKYPSRVIELAVKRMLPNGKTDLPKRLKVYSGGNHPHQAQKPK
EMMPKKIVAVVKLALRAGKATPAPPVGPALGQHGVNIALFCKEYNARTADKTDLIIPV
EISIYEDRSFSFILKTPPASVLLKVNLQINKGSSKPNKVKVGSITKKGLEEIARIKLPDLNT

DNIQSAIKIIEGTAKNMGITIMLSPKRTKFRKQQRGRLRGKTIQKKSLAFGEYGLQAQE
PVWLTARQIEATRRTITRYTKRGGKLWIMVFPDKPITARAEESRMGSGKGAPQYWVS
VVKPGKVLFELAGVAESIAVHALRMAAYKLPIKTKLISAMIQPQTYLNIVDNTGAKKL
MCIRILGSNRRYGRVGDIIIGVVKEAVPNMAVKRSDIVRAVIVRTRQPIQRSDGMAIQF
DDNAAVIINLDNNPRGTRVFGPVAREIREKNFVKIASLAFEVVMYSIIESFGRQFWVEP
KKFQDFSNFKLPTSVIFDRVMFFTNIELGRPILNYVQGSLLPGVKKSKLLVFKMRSKKK
YRRKIGHRISSRRVRFMAKAKARMIRMSPLKVRRVLNQIKGCSYEEALVLLRFLPYRAC
HPVAKVLKSAAANALNNYSIPRSYLQIEKAFVENGPILKRIRPRAKGRMYPIKKRTSHICI
VVMIKKNIHPKWFVKTKVYCDGQSIMTISSTKPELHVDIWSGNHPFFTGSQKIIDTEG
RVERFLKKYNMMAHKKGAGSTKNGRDSKAKRLGVKVVGGQFVQLGQIIIRQRGSAF
QAGENVGTGKDYTIYALKEGHVVYTVSIDMKVRPSVKKICEKCRLIKRAGKLRVICTN
KKHKQRQGMAVPKKRTSRTKTKTRKAQWFRKAYIASQKAWSLSCSIANSNSFVLMKL
VIEIEKRSFQFRIGPFKKTMATTIGSALRRTLLSMTKTLAITSV-----
CGNFHEGNSIREDLFELSLNLQHVHIKSSIGRLKKKGPAIITAKHLQLEEGLEIVNPNQ
YICTVNESYTIDLALMINSPGTNQGTIIIVDPIDSAFQSCGFEVIQTDEFLRFVVISRGGI
EPAIAVSLAVQELKEILSILLDIKHLKLPEYLELFLRREGFVSVNNLISVPLLKRILNKIDIN
MIEESLNLLNQIITKKDLEDILAWMFRNYGIRKTCMLGELLKEAGFNYATQGGLSIHL
EDLKVPRSKTSLVSLTNTELEKGETLYKRGELTSSEWFQKQVMAWNLTSEVLKNEIV
DFFERTDPLNPLYMMTFSGARGNLSQVRQLIGMRGLMSDQKGEMIGSAIQRNFREG
LTVIDFLISSYGARKGLVDTAIRTADSGYMTRRLIDIAQDVVVRQFDCQSKYGILILLG
RILAKPIFLAHRGQEIDFVLLKLIKKIQIVSVRSPLICQSYRSVCQTCFGWDLTQHRLIEI
GEAIGIIAAQSIGEPGTQLTMRTFHTGGVFSELSQQIRSKFSGQVKFAENLKTNFVRTN
QGDYSQVIINTSFFNILEVLRIKVERDDLLMIKNGEFIKKGQPIVQLASQLEELTTKFSG
EIYYEYIVWVLGGTLVILPEKLIRSFSHPKYVFAKTKFVIKSNFSTFLYILKGGFILPEESY
DAFPKIFQVANFVEKISSNIYSPVNGLIQLKLTNLIIKSGGFFFAGDNLFGRILLNKIVYSE
LLRPLVYYNVKNIIEKVLFASQTISTVRINQILEPYTVVSFSEFRGLESKALDIKSKFQELL
LVSNHHVRSVYFDEKEFDLVVLTLGGKILNKKGSKIDFRIGKPYLFTEGAKVYKNHED
FAPENTVLGFVTYRIFKTQDIIQGLPKVEEILEARSPSNQPHSQIEITDRFHIDPQQLLD
IYFNYFKATLRSLYRLASMFIKSIQGVYESQGIKIVDRHLEVIVRQMIVKTKIEYPGNTPL
VSGEYIDVQQIVSLNKMLQKKFYKPAILGLTKAALTTESFISAASFQETIRILTQAAIEGK
VDWLRGLKEKVIVGGLIPCGTGMYLANIPDLIEIQKSSFCWFLEEGLSDELKQLSTLG
DFETVEIRFFHNEFYFHQPFRIPYESRRNKLSYSIKLYMPIEVQNKVQIDLHTLCLCELP
LMTDRGTFVINGCERVILGQIVRSPGIYYTIGATLISNRGSWLYFEYDRIWIKTDRIEEIP
ISILLEDIGNQTIFSHLENSHYFEYWELSEIFDARYYSLGKVGRYKVNKKLGINLPQRVQ
TLTLHDIVEIVDYLIGLRVLVDDIDSLENRRIRSVGELLQVQFSHAFLRFGAFLEEKRD
MPPISAVDEFFATNPLSQYLDQINPLAELIHKRRVTVLGPGGIPFEKASLAIRDIHPSYY

GRLCPIDTPEGEKAGLVASLATFVHTDNQGFLKTPFFSIRQGKIIGLN---
YLNVGQEDGKTVGMGDSLVGFLLTKNIGVKENDEFRLTAPSEVNYLSVSPFHLFSPA
VGLIPFFEHDDANRTLMGAHMQRQAVPLIRPQKPIVGTGIESNLAIESGSLLLSYTKGI
VKFVSSNSIWISYRLEKFQASNQSTILNQRSIVWVGEYVESGSIIADGPSTDEGELALGK
NVTVAYMPWGGFNYEDAIVVSERLVYENIFTSIHIEKLEVEIEDNDRGPDTRDLPESLR
NLDENGLVYIGAYVQPGDAIIGKLSPKRRENT--
YGRLLEELFVSDTSLRVPILDVRVLDRIFIANIRRMQIGDKMAGRHGNKGVISNVAPLS
DLPFLPDGTLVDIILNPLGVPSRMNVGQLFECLLGWVGEKLEKRFKVFPFDEIYSLEAS
RSLVYKKFEEIKSNQQILLNRFSPGKVILRDGRTGLPFDNPITVGKPYMLKLIHLVDDK
IHARSIGPYSVITQQPLGGRSREGGQRFGEMEVWALEAYGTAYTLQELLTLKSDDIPG
RLKAYKAIPSPGIPESFRVLLRELRSLAVDIHALRFIAFSPNLKPVKMFEMVGVRLASPEK
IREWSERKLP-
NGIVGEIRKPDTMNYRTGRPEPDGLLCEKIFGPLKSWECACGQYNYCQNCGVEVTES
HVRRHRMGHVTFKYPITHTWYLKGSPSYLSIVLLELTKGSQLILKMLKSFDLNDEIKQ
LRQDLSHSSSKRERLLKRLRLLETFVATKTDPSWMVLKVLPVLPPALRPVFELPNGTY
MSSEFNEHYRRIVMRKNRLMKFCEQIVPEFVVINEKILIQDAVDDLIDNGSRGRESYGP
RNKALRCLNDLIAGKEGRFRQNLLGKRVDYSGRSVIVVGPTLMLDQCALPYEMALNL
FGPYLIIIPRALTALQRNPIVWTLLILVSVSLVLLNRAPTLHRLGIQAFRPVLTTGRAIQL
HPLVCPGFNADFDGDQMGVHLPLTRKTQLEAYR-
MLSSNNLLSPATGRPILTPSQDIVLGWYYLTTQTLPYTKGANRYFTTFGEISEALEHEK
IYLHSSIWLKYITSAYIRTTPGKILINELLAAAIMSRYRGPRLRLQRRLGLNHLTRKRIRN
KKKVRSEYRMRLEEKQKLKFNYSITETQLFRYVKEARRIKGSTGLIILQLLEMRLDTIVF
RLNFSPTILAARQLVSHGHVKVNDQIVTIPSFQCQPGDSIQISDTKSIRAIVDENALPYH
LAASVKQLRGGVKKVVDRRDIRLPVNELLVVEYYSRMGQKVHPLGFRLGITEEHKTK
WYANFSNYANQLKLTDELRNVWINLRTNIVITYPNQIQITIFCVNPLVILSSLGKELRN
LVVILKKVKTPFIEPNFLLQSVSKKLEKRMPFRRAIRSTLTDFKKGIKIQVAGRLNGAEI
ARTEWVREGKVPLHTLQAKLEYSTARAQTIYGILGLKIWICKMTNDTISDMLTRIKNG
NLVGHKVVKVDSTRINYQLIKLLRAEGLIRQYETVGRHFIFVYLKYSTNPTRPIIRGLKR
VSKPGLRVYYVNTNQIPTILGGQGLAVLSTSKGIMTNFKAKDLGIGGELLFYIWMISHK
VVEIRRVCKVGKGGKTLSFRALVVVGNQRGIVGIGIGKAGEVLNAIKKGQYKAIQNRI
QIPITRTKTIPHRSEACFGAAHVMLRPAAPGSGVIAGGAGRAVLELAGIKNILAKQLRS
KNKINNARATLLALNNLMSRRKRIKKRLPGPDPIYNSYLVSLLSLRVIKSGKKQLAERIV
YKALDYIKQKTNLDGLITLEKAVQNVSPVVELKPKRIGGATYQVPIEVKRLRATNLAL
KWLLKYSRDRSGKNMSFKLARELMDASKGIGNSIRRREEVHKMAEANKAFSFFKRMFR
IRLKAYNSRVLRISSLLLQKELAKLDTFLKGPIRLPIKKRYYSLLRSPHIDKDSQEQFEIRR
HKWIFDIQVPKNYINLLSSFPPGVDISIMPTVQQLIRNKRRTIKKKSKAGALKACPQRR

GVCTRVYIVTPKKPNSAMRKVARIRLTSGFEVTAHIPGEGHNLQEHSVVLIRGGRVKD
LPGVRYRVIRGALDTVGVAKRMQGRSKYGARKLKMGKRKINLSLGFACIHSTFNNTII
SITDPKGNVLTWASAGSNGFKGSRKKTQYAAQMAAKNASAKALKLGIKKVGIILNGP
GNGREIAIKGIHATGLEIISIEDKTGVPHNGCRAPKRRRVMIRIAGVDLPDTKSIEIALTY
IYGIGRTRSKEILNLLKINETKTKFLTDKDISQLRELIESYTTESDLKRLVSLNIKRLVEIN
CYRGRRHIQGLPLRGQRTKTNAQTRKKTASKRRMGLTEKIGIVVSTKMQKTIVVIVE
NKFQHPRYAKTVIQTKRYLVHDEDNTAKLGDKILMTQTRPLSKNKRWRLEMAKFSVI
KRQIRRENLVRRYYELRQKLKHELKEVASFKAKMDLHIKLQKLPRDSSKTRLRNRCWK
TGRGKAVFRDFGLCRHMVREMANQGLLPGVCKSSWMIRSTKKVPFVAYHLLKKINR
LKTDTIKTWSRASVILPIMVGYTIAVYNGRQHVPIFISDQLIGHRLGEFVPTRTFRSHK
NDRKAKRMIKSAKKRVRVGQRNLKLNRFYKSTVKTLVKKYRIAIKSFLKSLVSKIYSRID
KAAKKKVFHPRKAARQKSRINKILKKMTQIFDEKNACIPVTLIKIGPCIVTQIKTLASDG
YNAIQLGFGINNKSTKALQGHLKKSGTPECHFLKEYHINQEDFELGQKFDITNFLSVQ
YIDVRGKTIGKGFAGTVKRYGFGRGPMTHGSKNHRAPGSIGAGSTPGRVYPGKRMA
GRLGGKQTTIKRLTVLKIDQEENLLVVKGCIPGKPGNLISLTMAKQKGSRIIIHLECTS
CRTNNKRAEGVSRYTTTKNRRNTPARLEQKKFCPYCNSHTLHKETKMTQIFDEKNA
CIPVTLIKIGPCIVTQIKTLASDGYNAIQLGFGINNKSTKALQGHLKKSGTPECHFLKEY
HINQEDFELGQKFDITNFLSVQYIDVRGKTIGKGFAGTVKRYGFGRGPMTHGSKNHR
APGSIGAGSTPGRVYPGKRMAGRLGGKQTTIKRLTVLKIDQEENLLVVKGCIPGKPG
NLISLTMTTISNYWIIIEKEFCELSFSTNPDYLIHKAFCLYRKTKWIRNASTKTKSEVSGG
GRKPWAQKGRGKARAGSIRSPLWRGGGICFGPKPMLYNPKLNNREYDHAFRTLLVE
KQKSIIPISLVESGKTKSAKQIVLDLIGKTIIVSPEEFTIFLKGIKNISNLNLVKDNELTLH
HILRSNYILITAYSSYNLTRKNMLKIRLKRCGRKKSPSYRIVLIPSQSKRDGRAIEELGYY
NPLRNLLVYNKERIQLHLSQGAQPSQTVRNFLIKIIMILKYPLATEKASNPFLKNSYTFI
VDSRADKETIKSAFEYVFKVKVIKVNTLITPKKLKRLPSYKKAIIKLAPEYSLDFFGMSSV
ESKFSTIGVGDTIRVGVLLIEGNKERTQFFEGTVIAIKKREGRRILRVRKLSQGIGIEKLF
LMDSPKVVSIERKKITKVRRAKLYFLRGLSTKPKKLKDQS

>rbcL gene dataset

>HQ710603.1 Rhizochromulina marina CCAP 950/1

CTTGCTTATTCCGTTTACACACAACCAGGTGTAGATCCAGTTGAAGCCTGCAGC
TGTTGCGGTGATCTCTACTGCCTTGGACAGTAGTATGGACGGATTTTAACAGCT
TGTGATGTTTATCTGCAAAAGCGTATCGTGTAGATCCAGTTCAGAGCAATTTTT
TGGTTATATTGCAATTCGAGGAAGGTTCGATTGCAAACAACAGCTTCAATTTTG
GGAACTCGGGTTCAAAGCCTAAAAGCACTTCGTTTAGAAGATATGCGTATTCCT
ATGCCTATCTGAAAACGTTCCAAGGTCCTGCGACAGGTGTAGTTGTGGAACGTG
AACGTCTAAACAACTTTGGGCGACCAGTTCTAGGGCTACAGTGAAGCCGAAATT
AGGTCTTTCTGGTCGTAACTATGGTCGTGTAGTATATGAAGGTTTAAAGGGTGG
TCTAGATTTCTTAAAAGATGATGAGAACATCAACTCACAACCATTTATGCGTTGG
CGTGAACGTTTCCTTTATGTACAAGAAGGTATTACACGTGCATGTGCTTCAACA
GGGGAAATTAAAGGTTCATATATGAATATGACGCTTCTACGATGGAAGACGCAT
ATGAACGTGTGAGTACGCAAAAGAATTAGGAACAATTATTGTTATGGTTGACCT
GTATGGGTTACACTGCCTTACAATCTTGTTCTATTTGGGCGCGTAAGAAGATTT
AATTCTTCACTTACACCGTGCAGGTAACTCGACATATGCACGTCAAAAAAACCAT
GGTATTAACTTCCGTGTAATTTGTAAATGGATGCGATGGCTGGGCTGGATCATA
TTCATGCCGGTACAGTAGTTGGGAAACTAGAGGGTGATCCAAATGGTTAAAGGT
TCTACAACACGCTACTTGATACAGTGACAGATAACTTGCACAAGGTCTATTCTTT
GCACAAGATTGGGCATCATTACGTAAATGTCTACTGTAGCTTCTGGTGGTATCC
ACTGTGGACAAATGCATCTACTAAATTACTTATGATGATTGGTACTTCAGTTTG
GTGGTGGGACAATTGGGCATCCTGACGGTATTCAAGCTGGTGCAACTGCAAACC
GAGTGGCTTTAGAAGCTATGGTATTAGCGCGTAACGAAGGTCGGATTATGTTAA
TGAAGGCCCAGAGATCCTTCAACAAGCTGCAAAAATGTGTGGTCCTTTACAAAC
GGCATTAGATCTATGGAAAGGAATTTCATTAACTACAGCTCTACGGATACGGCT
GACTTTGCTGAGACACCACTGCAAACATGTAA

>AB081642.1_Pteridomonas_danica

CGTTACGAAAATCCCATATGCCTGGGAGAATATAAAACAGATGTTCTTGCTCATT
CCGTTTACACTCAACCAGGTGTAGATCCAGTGGAAGCCCGCAGCTGTAGCGGTG
ATCTCTACTGCACTGGACTGTTGTATGGACTGACCTTAACTGCATGTGATATCT
ACCTGCTAAAGCTTACCGTGTAGATCCAGTTCATCATAATTCTTTGGTTACATTG
CAATGTGATATCGAAATCTTAGCTAACAACTGCTTCTATTTTGGTAACTTGGTTT
CAAAGCTTTAAAGCACTTTCTCTAGAACATATGCGTATTCCTTTGCATATCTTAA
AACATTCCAAGGACCAGCAACAGGTGTAGTTGTAGAACGTGAGCGTTTAAACTG

CTTCGGTCGTCCATTACTAGGGCTACAGTTAAGCCTAAACTAGGTCTTTCTGGT
CGTAACTACGGTCGTGTGGTATACGAAGGTCTTAAAGGTGGTCTAGACTTCTTA
AAGGATGATGAGAACATTAACTCTCAACCATTCATGCGTTGGCGTGAGCGTTTC
CTTTACGTTCAAGAAGGTATTACACGTGCTTCAGCAGCTACTGGTGAAGTAAAG
GGTTCGTACATGAACATCACGCCGGTACTATGGAAGATGTATATGAGCGTGCGA
ATACGCTAAAGATCTTGGTACTGTCATTGTGATGATCGACCTGTATGGGTTACA
CAGCTATTCAATCATCAGCAGTTTGGGCACGTAAAAAGATATGATTCTTCACCTT
CACCGTGCAGGTAACTCAACTTATGCACGTCAAAAAAATCACGGTATTAACTTCC
GTGTAATTTGTAAATGGATGCGATGGCTGGTGTTGACCACATTCACGCAGGTAC
TGTAGTAGGTAAATTAGAGGGAGATCCAAATCGTTAAAGGTTCTACCACACTCT
ACTAGATGTTACATCTGATAACTTACCCAAGGTATATTCTTCGCTCAAGATTGGG
CGTCATTACGTAAGTGTCTACAGTAGCTTCTGGTGGTATTCACTGTGGTCAAAT
ACACCTCTTTCACTACCTTT--

>AB081643.1_Ciliophrys_infusionum

CGTTACGAAAATCCCATATGCCTGGGAAACTTTAAAACAGATGTTCTGGCATATT
CCGTTTACACTCAACCAGGTGTAGATCCAGTGGAAGCCCGCAGCTGTAGCGGTG
ATCTCTACTGCACTGGACTGTTGTATGGACTGACCTTAACAGCTTGTGATGTTT
ATCTGCGAAAGCTTACCGTGTAGATCCAGTCCAATGTAATTCTTTGGTTACATC
GCAATGTGATATCGAGATCATTGCTAACGACAGCTTCGATCTTGGTAACTTGGT
TTCAAAGCATTAAAGCGCTTCGTTTAGAAGATATGCGTATTCCTTTGCATACTTA
AAAACTTTCCAAGGTCCAGCAACAGGTGTTGTTGTAGAACGTGAGCGTTTAAAT
AACTTCGGTCGTCCAATTCTTGGGCTACTGTAAAACCTAAGCTAGGTCTTTCTG
GCCGTAACTATGGTCGTGTAGTATACGAAGGTCTAAAAGGTGGTCTAGACTTCC
TAAAAGATGACGAGAACATTAACTCTCAACCATTCATGCGCTGGCGTGAGCGTT
TCCTTTATGTTCAAGAAGGTATTACGCGTGCTTCAGCTTCTACGGGTGAAGTTA
AAGGTTCGTACATGAACATGACGCCTCAACTATGGAAGATGCTTATGAGCGTGG
GAATACGCAAAAGAACTTGGTACAATCATTGTGATGGTTGACCTGTATGGGTTA
CACAGCGATTCAATCAGCAGCTATTTGGGCACGTAAAAAGATATGATTCTTCATT
TACACCGTGCAGGTAACTCTACGTATGCACGTCAAAAAAATCACGGTATTAACTT
CCGTGTAATTTGTAAATGGATGCGATGGCTGGTGTTGACCACATCCACGCAGGT
ACTGTAGTAGGTAAGTTAGAGGGAGACCCAAATCGTTAAAGGTTCTACAACACT
CTACTAAACGTTACGACTGATAACCTACCCAAGGTTTATTCTTTGCTCAAGATTG
GGCGTCACTACGTAAATGTCTACTGTAGCTTCTGGTGGTATTCACTGTGGTCAA

ATGCACCTCTTAAACTACCTATGACGACTAGTTCTTCAATTTGGTGGTGGTACA
ATTGGACACCCAGATGGTATTCAAGCTGGTGCAACAGCTAACCGTGTAGCGCTT
GAAGCTATGGTTCTAGCACGTAACGAAGGTCGGACTACGTTGCTGAAGGTCCAG
AGATTCTTCAAGAAGCTGCAAAAATGTGTGGTCCTCTACAAACAGCTCTAGATCT
ATGGAAAGGTATTTCATTAACTACAGTTCTACGGATACAGCTGACTTTGCTGAG
ACTCCACTGCAAACGTGTA-
>AB097409.1_Helicopedinella_tricostata

CTGGGAGCACATAAAACTGATGTATTAGCTTATTCCGTTCACACACAACCAGGT
GTAGATCCAGTAGAAGCCTGCTGCTGTAGCGGTGATCTCTACTGCACTGGACTG
TTGTATGGACAGATTATAACAGCTTGTGAGCGCTACCTGCTAAAGCTTACCGTG
TAGATCCAGTCCGGAGTAATACTTTGCATTTATCGCAATGTGATATTGAAATCTT
ATCTAACAACAGCTTCTATCTCGGTAACTTGGATTCAAAGCCTTAAAGCACTTGC
TCTAGAAGATATGCGTATTCCTATGCATACCTTAAAACATTCCAAGGTCCAGCAA
CTGGTGTAGTTGTAGAGCGTGAGCGTCTAAACTGTTTCGGTCGTCCTTTATTAG
GGCAACTGTAAAACCTAAATTAGGTCTTTCTGGTCGTAACTACGGTCGTGTAGT
ATATGAAGGTCTTAAAGGTGGTTTAGATTTCCTTAAAGATGATGAGAATATTAA
CTCTCAGCCATTTATGCGTTGGCGTGAACGTTTCCTTTACGTACAAGAAGGTAT
CATGCGACCATCTGCGGCTACTGGTGAAGTTAAAGGTTCTTACCTTAACGTTAC
GGAGCTACAATGGAAGATGTTTATGAGCGTGCGAGTATGCTAAAGAATTAGGTA
CTGTTGTAATTATGATTGACTTGTATGGGTTATACTGCAATTCAGTCAATCGCA
TTATGGTCTCGTAAAAAGATATGATTCTTCACCTTCACCGTGCTGGTAACTCTAC
TTATGCACGTCAAAAAAATCATGGTATTAATTTCCGTGTAATTTGTAAATGGATG
CGATGGGTGGTGTTGACCATATCCATGCTGGTACTGTAGTAGGTAAGTTAGAAG
GTGACCCAAATGGTTAAAGGTTCTATAATACTTTATTAGATGTTAAGACAGATAA
CCTACCCAAGGTTTATTCTTCGCTCAAGATTGGGCTTCATTACGTAAGTGTATG
CAGTAGCATCAGGTGGTATCCATTGTGGTCAATTACACCACTTTAACTATCTAT
GACGATTAGTTATGCAATTTGGTGGTGGTACAATCGGTCACCCAGATGGTATCC
AGGCTGGTGCAACTGCAAATCGTGTTGCTCTTGAAGCAATGATTTTAGCCCGTA
ACGAAGGTCGGATTATATGTCTGAAGGTCCAGCAATTCTTCAAGCAGCTGCTAA
AATGTGTGGTCCATTACAGACAGCTTTAGATTTATGGAAAGGTATTTCTTTAAC
TATAGTTCTACAGATACAGCAGATTTTGCTGAAACTAAACTGCAAACCGA---
>AB097410.1_Dictyocha_fibula

CTGGGAGAACTTAAAACGGATCTTCTTGCTTATTCCGTTTACACACAAAACGGT
GTAGACCCTGTTGAAGCCTGCAGCCGTAGCGGTGATCTCTACAGCACTGGACTG

TTGTTTGGACAGATCATAACAGCTTGTGATGTTTACCTGCTAAAGCTTATCGTG
TAGACCCAGTCCCACGAAGTACTTCGGTTATGTAGCAAATCGACATTGGAATCA
TAGCTAACGACAGCATCTATCTTGGTAACTTGGTTTCAAAGCGTTAAAGCTCTT
CGTTTAGAAGATATGCGTATTCCTTCGCATACTTAAAGACTTTCCAAGGTCCTG
CAACAGGTGTAGTTGTTGAGCGTGAGCGTATGGACAAATTTGGTCGTCCATTAC
TTGGGCTACTGTTAAGCCAAAATTAGGTCTTTCTGGTAAGAACTACGGTCGTGT
TGTGTTCGAAGGTCTTAAAGGTGGTTTAGACTTCCTTAAAGATGACGAAAATAT
TAACTCACAAGCATTTATGCGTTGGAGAGAGCGTTTCCTTTACTGTCAAGAAGG
TATTCAACGTGCTGCAACTGCAACAGGTGAAGTTAAAGGTAGTTACCTTAACGT
TACGCTGGTACTATGGAAAATGTTTATGATCGTGCGAGTATGCTAAAGAACTTG
GAAGTATCATTATTATGATGGACCTGTATGGGTTACACAGCAATCCAATCAATT
GCGCTTTGGGCACGTAATAAGACATGATCCTTCACTTACACCGTGCAGGTAACT
CTACATACGCTCGTCAAAAGAACCACGGTATTAACTTCCGTGTAATTTGTAAATG
GATGCGATGGCTGGTGTTGACCATATTCACGCAGGTACAGTTGTTGGTAAGCTA
GAGGGTGACCCTAATGGTTAAAGGTTCTATAACACATTACTTGATGTTAAGACT
TTTAACCTTCCCAAGGTATCTTCTTCGCACAAGATTGGGCTTCTTTACGTAAGT
GTATGCAGTAGCTTCTGGTGGTATTCACTGTGGTCAAATGCACCATTTTAACTA
CTTATGACGATTAGTTCTACAATTTGGTGGTGGTACAATTGGA---------------------
----

>AB280607.1_Pseudochattonella_verruculosa

ACTCTTAAAACAGATGTACTAGCACATTCCGTTCACACTCAACCAGGTGTTGACC
CTGTAGAAGCCTGCAGCAGTTGCGGTGATCTCTACTGCACTGGACTGTTGTATG
GACTGACCATAACTGCTTGTGATGTTTACCTGCGAAAGCTTACCGTGTAGACCC
TGTCCAACGCAATTCTTTGGTTACGTTGCAATGTGACATTGAAATCTTTGCTAA
CTACAGCATCAATCTTGGTAACTTGGTTTCAAGGCCTTAAAGCTCTTCGTCTAG
AAGATATGCGTATTCCTTTGCTTACTTAAAGACTTTCCAAGGTCCAGCAACTGG
TGTAGTTGTAGAGCGTGAGCGTATGGATAAGTTCGGTCGCCCTATGCTAGGGC
AACTGTTAAGCCTAAGCTAGGTCTTTCAGGTAAGAACTACGGTCGTGTAGTATT
CGAAGGTCTTAAAGGTGGTCTTGACTTCCTTAAGGATGACGAGAACATTAACTC
TCAACCATTCATGCGTTGGAGAGAGCGTTTCCTTTACTGTCAAGAAGGTATCCA
ACGTGCAATGGCAGCAACTGGTGAAGTTAAGGGTTCTTACATGAACATCACGCT
GGTACTATGGAAGATATCTACGAGCGTGCGAGTACGCCAAAGATCTTGGTGCAG
TTGTAGTTATGATTGACCTGTATGGGTTATACTGCAATTCAATCAACAGCAATCT

GGGCTCGTAAGGCGACATGCTTCTTCACCTTCACCGTGCTGGTAACTCAACATA
TGCACGTCAAAAGAACCACGGTATTAACTTCCGTGTAATTTGTAAGTGGATGCG
ATGGCTGGTGTTGACCATATTCACGCTGGTACAGTTGTAGGTAAGCTAGAAGGT
GATCCAAATGGTTCAAGGTTCTACTCTACTCTACTTTCTGTTAAGTCAGATAACC
TACCAAAGGCCAATTCTTCGCTCAAGATTGGGCATCTCTACGTAAGTGTATGCA
GTAGCTTCTGGTGGTATTCACTGTGGTCAAATGCACCACTTTAACTACCTATGA
TGATTAATTCTACAATTTGGTGGTGGTACAATTGGTCACCCAGACGGTATCCAA
GCCGGTGCTACTGCTAACCGTGTTGCTCTAGAAGCAATGGTTATCGCACGTAAC
GAAGGTCGGACTACGTAAATGAAGGCCCTGAAATTCTTCAGGAAGCAGCTAAAA
TGTGTGGTCCTCTTCAAACTGCATTAGATCTATGGAAAGGTATTTCATTAACTA
CACTTCTACAGATACAGCAGACTTTGCAGAAACTCCACTGCAAACGTATAA
>AB280611.1_Rhizochromulina_sp._CCMP237

GAAACCTTTAATCAGATGTACTTGCTTATTCCGTTTACTCACAACCGGGTGTAG
ATCCAGTGGAAGCCTGCAGCAGTAGCGGTGATCTCAACAGCTCTGGACAGTAGT
TTGGACAGATTATAACAGCTTGTGATGTTTACCTGCAAAAGCTTACCGTGTTGA
TCCAGTCCAATGTAATTCTTTGGTTACATCGCAATGTGACATTGAAATCATTCCA
AACTACAGCTTCAATTTTGGTAACTTGGTTTCAAAGCTTAAAAGCTCTTCGTCTA
GAAGATATGCGTATTCCTACGCGTACCTACGTACCTTCCAAGGTCCAGCAACAG
GTGTTATCGTAGAGCGTGAGCGTCTAAACAACTTTGGTCGTCCAATTCTAGGGC
TACTGTAAAACCTAAGCTAGGTCTTTCAGGTCGTAACTATGGTCGTGTAGTTTA
TGAAGGTCTAAAAGGTGGTCTTGACTTCTTAAAAGATGATGAAAACATCAACTTT
CAACCATTCATGCGTTGGCGTGAGCGTTTCCTTTATGTACAAGAAGGTATCACA
CGTGCTCAAGCATCTACTGGTGAAGTTAAAGGTTCATACATGAACATGACGCAG
CAACGATGGAAGATGCATATGAGCGTGGGAATATGCTAAAGAGCTTGGTACAAT
TATTGTGATGGTTGACTTGTATGGGTTATACTGCATTACAATCTGCGGCTATCT
GGGCTCGTAAGAAGATATGATTCTTCACTTACACCGTGCAGGTAACTCTACATA
TGCACGTCAAAAAAATCATGGTATTAACTTCCGTGTAATCTGTAAGTGGATGCG
ATGGCTGGTGTTGACCATATTCACGCAGGTACTGTTGTAGGTAAACTAGAAGGT
GACCCAAATGGTTAAAGGTTCTACAATACTTTATTAGACGTAGAAACACCTAACT
TAGCCAAGGTTTATTCTTTGCACAAGCATGGGCATCTTTAAGAAAATGTCTTCA
GTTGCGTCAGGTGGTATCCACTGTGGTCAAATGCACATTTTAAACTACCTATGA
TGACTAGTTCTT---

>EF455923.1_Tribonema_missouriense_strain_UTEX_B2549

ATGTCTAAAAACGTACAAGAGCGTACTCGAATCAAAAGTGAACGTTATGAAAATC
CCTTATGCCTGGGAGATATTAAAACAGATATTTTAGCATATTCCGTTTACTCTCA
ACCAGGTGTAGACCCTGTAGAAGCCAGCTGCTGTAGCGGTGATCTCAACTGCAC
TGGACAGTAGTATGGACGGATTATAACAGCTTGTGATATCTACCTGCGAAAGCA
TACCGTGTAGATCCTGTCCGAAGTAATTCTTCGCTTACATTGCAATGTGAAATT
GAAATCATCGCAAACAACTGCATCTATTTTGGTAACTTGGCTTCAAAGCTTTAAA
GCGTTACGTTTAGAAGATATGCGAATTCCTTTGCATACTTAAAAACTTTCCAAGG
TCCTGCTACAGGTCTAGTTGTGGAAAGAGAAAGAATGGACAAATTTGGTCGTCC
ATTATTAGGGCAACTGTAAAACCAAAATTAGGTCTTTCAGGTAAAAACTACGGTC
GTGTAGTATATGAAGGTCTTCGTGGTGGTCTTGACTTCTTAAAAGATGATGAAA
ACATTAACTCTCAACCATTCATGCGTTGGAGAGAACGTTTCCTTTACTGTGTTG
AAGGTATTAACCGTTCATCTGCATCTACAGGTGAAGTTAAAGGTTCTTACTTAAA
CGTAACGCTGGTACAATGGAAGACATGTACGAACGTGCGAATACGCAAAAACTT
TAGGCTCAATCATTATTATGATCGACCTGTATTGGTTACAGTGCTATCCAATCAA
TGGGTATTTGGAGTCGTAAAAAGACATGATCCTTCACTTACACCGTGCTGGTAA
CTCAACTTACGCACGTCAAAAAAACCACGGTATTAACTTCCGTGTAATTTGTAAG
TGGATGCGATGGCTGGTGTTGACCACATCCACGCAGGTACTGTAGTAGGTAAAT
TAGAAGGTGACCCTAATGGTTAAAGGTTCTACCACACTTTATTAGACGTTACAAC
AGATAACCTTCCCAAGGTTTATTCTTCGCAATGGACTGGGCTCTTTTACGTAAA
ACTGTACAGTAGCTTCTGGTGGTATCCACTGTGGTCAAATGCACCATTTTTACT
ACTTATGATGACTAGTTCTTCAGTTTGGTGGTGGTACAATTGGTCACCCAGATG
GTATCCAAGCAGGTGCAACAGCAAACCGTGTAGCTTTAGAATCAATGGTATTAG
CTCGTAACGAAGGTCGGACTACTTAAACGAAGGACCTCAAATCTTACGTGATGC
TGCTAAAATGTGTGGACCACTAAAAACAGCATTAGATTTATGGAAAGATATTACT
TTGATTACACTTCTACAGATACTCCAGATTTCGTAGAAACTGCACAGAAAGTAAG
TAA
>AM850238.1_Pseudochattonella_farcimen

ATTCTTCGGTTACGTTGCAATGTGACATTGAAATCTTTGCTAACTACAGCATCAA
TCTTGGTAACTTGGTTTCAAAGCCTTAAAGCTCTTCGTCTAGAAGATATGCGTA
TTCCTTTGCTTACTTAAAGACTTTCCAAGGTCCAGCAACTGGTGTAGTTGTAGA
GCGTGAGCGTATGGATAAGTTCGGTCGCCCTATGCTAGGGCAACTGTTAAGCCT
AAGCTAGGTCTTTCAGGTAAGAACTACGGTCGTGTAGTATTCGAAGGTCTTAAA
GGTGGTCTTGACTTCCTTAAGGATGACGAGAACATTAACTCTCAACCATTCATG

CGTTGGAGAGAGCGTTTCCTTTACTGTCAAGAAGGTATCCAACGTGCAATGGCA
GCAACTGGTGAAGTTAAGGGTTCTTACATGAACATCACGCTGGTACTATGGAAG
ATATCTACGAGCGTGCGAGTACGCTAAAGATCTTGGTGCAGTTGTAGTTATGAT
TGACCTGTATGGGTTATACTGCAATTCAATCAACAGCAATCTGGGCTCGTAAGG
CGACATGCTTCTTCACCTTCACCGTGCTGGTAACTCAACATACGCACGTCAAAAG
AACCACGGTATTAACTTCCGTGTAATTTGTAAGTGGATGCGATGGCTGGTGTTG
ACCATATTCACGCTGGTACAGTTGTAGGTAAGCTAGAAGGTGATCCAAATGGTT
CAAGGTTCTACTCTACTCTACTTTCTGTTAAGTCAGATAACCTACCAA------------

>EU912438.1_Vaucheria_litorea
ATGTCTAAGTCTGTACAAGAGCGTACTCGAATTAAAAGCCAACGTTATGAAAATT
CCTTATGCTTGGGATTTATTAAAACGGATGTATTAGCGTATTTCGTTTACTCGC
AACCTGGTGTAGATCCTGTAGAAGCCAGCAGCTGTTGCGGTGATCTCAACTGCG
CTGGACAGTAGTATGGACTGATTATAACTGCTTGTGATATTTATCTGCAAAAGC
ATATCGTGTAGATCCAGTCCGAAGTAATATTTTGCATATATTTCAAATTGAAATT
GAAATCATAGCAAATAACTGCATCAATCTTGGTAATTTGGATTTAAAGCTTTAAA
GCTTTACGTTTAGAAGATATGCGAATTCCTTTGCATATTTAAAAACTTTTCAAGG
TCCTGCTACAGGTTTAGTTGTAGAAAGAGAAAGAATGGATAAATTTGGACGTCC
ATTATTAGGGCTACGGTAAAACCGAAATTAGGTCTTTCAGGTAAAAACTATGGT
CGTGTAGTATATGAAGGACTTCGTGGTGGTTTAGATTTCTTAAAAGATGATGAG
AATATTAATTCTCAACCATTTATGCGTTGGAGAGAGCGTTTTCTTTACTGTATG
GAAGGTGTTAATCGTGCAGTAGCAGCTAGTGGTGAAGTTAAAGGTTCATACCTT
AATACAACGCAGGTACAATGGAAGAGATGTATGAACGTGCGAATATGCTAAAAG
TATAGGCTCAATTATTGTAATGATTGACCTGTATTGGTTATACTGCTATTCAATC
TATGTCAATTTGGGCTAGAAAAGCGATATGATTTTACATTTACACCGTGCAGGT
AATTCTACATATGCACGTCAAAAAAATCATGGGATCAATTTCCGTGTAATTTGTA
AATGGATGCGATGGCAGGTGTAGATCACATTCATGCTGGTACTGTTGTAGGTAA
ATTAGAAGGTGACCCTAATGGTTAAAGGTTTTATAATACATTATTATTAACGAAA
TTAGATAATCTTTCCAAGGTGTATTTTTTGAAATGGATTGGGCAGCATTACGTA
AAACTGTACTGTAGCATCTGGTGGTATTCACTGTGGACAATTACATCATTTTTAT
TATTTATGATGATTTGTACTTCAATTTGGTGGTGGTACTATTGGACATCCAGAT
GGTATCCAAGCAGGTGCTACTGCTAATCGTGTAGCTTTAGAAGCTATGGTATTA
GCACGTAATGAAGGCCGGATTATATGAATGAAGGACCACAAATTTTACGTGATG

CTGCTAAAACTTGTGGACCGTTAAAAACTGCATTAGACTTATGGAAAGATATTAC
TTTGATTACACTTCTACTGATACCCCAGATTTCGTAGAAACGGCACACAAAGTAG
ATAA
>HQ710599.1_Apedinella_radians_culture_CCMP1767

CTTGCTCATTCCGTTTACACTCAACCAGGTGTAGACCCTGTAGAAGCCTGCAGC
TGTAGCGGTGATCTCAACTGCACTGGACTGTTGTATGGACTGACCTTAACTGCT
TGTGATATCTACCTGCGAAAGCTTACCGTGTAGATCCAGTCCAGGGTAATTCTT
CGGTTACATTGCAATGTGAGTTCGAAATCATAGCTAACAACTGCATCAATCTTG
GTAACTTGGTTTCAAGGCTTTAAAGCTCTTCGTTTAGAAGATATGCGTATTCCT
ACGCATACCTAAAAACATTCCAAGGTCCAGCAACTGGTGTAATTGTTGAGCGTG
AGCGTTTAAACCAATTCGGTCGTCCACTTCTAGGGCAACTGTTAAGCCTAAATTA
GGTCTTTCTGGTCGTAACTACGGTCGTGTAGTATACGAAGGTCTTAAAGGTGGT
TTAGACTTCCTAAAAGATGATGAGAATATTAACTCACAGCCATTCATGCGTTGGC
GTGAGCGTTTCCTTTACGTACAAGAAGGTATTACTCGTGCTTCTGCAGCAACAG
GTGAAGTTAAAGGTTCATACCTAAACGTAACGCAGCTACTATGGAAGAAGTTAT
CGAGCGTGCGAGTACGCAAAAGAAATCGGTACAATCATTATCATGATTGACTTG
TATGGGTTACACAGCTATCCAATCTATTGCACTTTGGGCTCGTAAGAAGATATG
ATTCTTCACCTTCACCGTGCAGGTAACTCAACTTACGCACGTCAGAAAAACCACG
GTATCAACTTCCGTGTAATCTGTAAATGGATGCGATGGCTGGTGTTGACCATAT
TCACGCTGGTACTGTTGTAGGTAAATTAGAAGGTGATCCTAATGGTTAAAGGTT
CTACAACACTTTACTAGATGTTAAGACAGATAACCTACCCAAGGTTTATTCTTCG
CTCAAGATTGGGCTTCATTACGTAAATGTATGCAGTAGCTTCAGGTGGTATCCA
CTGTGGTCAGCTACACATCTTAAACTACCTATGACGACTAGTTATGCAATTTGG
TGGTGGTACAATTGGTCACCCAGATGGTATTCAAGCTGGTGCTACTGCAAACCG
TGTTGCTCTTGAAGCAATGGTTATGGCACGTAACGAAGGTCGGATTACCTAGCT
GAAGGTCCACAAATTCTTATGGATGCAGCTCGTATGTGTGGTCCTCTACAAACA
GCATTAGATCTATGGAAAGGTATTTCATTAACTACACTTCTACAGATACAGCTGA
CTTTGCGGAAACTCCACTGCAAACGTATAA
>HQ710604.1_Rhizochromulina_sp._CCMP237

CTTGCTTATTCCGTTTACTCACAACCGGGTGTAGATCCAGTGGAAGCCTGCAGC
AGTAGCGGTGATCTCAACAGCTCTGGACAGTAGTTTGGACAGATTATAACAGCT
TGTGATGTTTACCTGCAAAAGCTTACCGTGTTGATCCAGTCCAATGTAATTCTT
TGGTTACATCGCAATGTGACATTGAAATCATTCCAAACTACAGCTTCAATTTTGG
TAACTTGGTTTCAAAGCTTAAAAGCTCTTCGTCTAGAAGATATGCGTATTCCTAC

GCGTACCTACGTACCTTCCAAGGTCCAGCAACAGGTGTTATCGTAGAGCGTGAG
CGTCTAAACAACTTTGGTCGTCCAATTCTAGGGCTACTGTAAAACCTAAGCTAG
GTCTTTCAGGTCGTAACTATGGTCGTGTAGTTTATGAAGGTCTAAAAGGTGGTC
TTGACTTCTTAAAAGATGATGAAAACATCAACTCTCAACCATTCATGCGTTGGCG
TGAGCGTTTCCTTTATGTACAAGAAGGTATCACACGTGCTCAAGCATCTACTGG
TGAAGTTAAAGGTTCATACATGAACATGACGCAGCAACGATGGAAGATGCATAT
GAGCGTGGGAATATGCTAAAGAGCTTGGTACAATTATTGTGATGGTTGACTTGT
ATGGGTTATACTGCATTACAATCTGCGGCTATCTGGGCTCGTAAGAAGATATGA
TTCTTCACTTACACCGTGCAGGTAACTCTACATATGCACGTCAAAAAAATCATGG
TATTAACTTCCGTGTAATCTGTAAGTGGATGCGATGGCTGGTGTTGACCATATT
CACGCAGGTACTGTTGTAGGTAAACTAGAAGGTGACCCAAATGGTTAAAGGTTC
TACAATACTTTATTAGACGTAGAAACACCTAACTTAGCCAAGGTTTATTCTTTGC
ACAAGACTGGGCATCTTTAAGAAAATGTCTTCAGTTGCGTCAGGTGGTATCCAC
TGTGGTCAAATGCACATTTTAAACTACCTATGATGACTAGTTCTTCAGTTTGGT
GGTGGTACTATTGGTCACCCTGATGGTATTCAAGCAGGTGCAACTGCTAACCGT
GTAGCTCTTGAAGCAATGGTTCTAGCTCGTAACGAAGGTCGGATTACCTAGCTG
AAGGTCCAGCGATTCTTCAAGAAGCTGCAAAAATGTGTGGTCCGTTACAAACAG
CTTTAGATCTATGGAAAGGTATTTCATTAACTACAGTTCTACAGATACTGCTGAT
TTCGCG----------------------
>KY433579.1_Dictyopteris_divaricata
ATGCCTGAGAATGTGCAGGAAAGAACTAGGATTAAGAGCGAACGTTACGAAGAT
TCCTTATGCCTGGGAGATACTAAAACTGATATTCTAGCTCATTCCGATCACACTC
AGCCCCGCGTTGATCCGGTAGAGGCCTGCCGCAGTAGCGGAGATCTCAACTGCT
CTGGACTGTTGTTTGGACTGATTATAACAGCCTGTGACATCTATCAGCAAAAGC
CTATCGAGTAGATCCTGTCCGAAGTAGTTCTTTGCATATATTGCAATGTGATAT
TGAAATCTTAGCGAATAACAGCTTCAATTTTGGTAATTTGGTTTCAAAGCCTTAA
AGCATTACGTTTAGAAGATATGAGAATTCCTATGCTTATTTAAAAACGTTCCAAG
GTCCTGCTACTGGTGTGGTCGTGGAAAGAGAAAGATTAGATAAATTTGGTCGTC
CGTTATTAGGGCAACAGTAAAACCTAAATTAGGTCTTTCTGGGAAAAACTATGG
GCGTGTTGTTTATGAAGGGTTACGTGGTGGACTTGACTTCCTGAAGGATGATG
AAAATATTAATTCACAACCTTTTATGCGTTGGAAAGAACGTTTCCTATACTGTAT
GGAAGGTGTTAACCGTGCTGCTGCTGCAACAGGAGAAATCAAAGGTTCGTACCT
TAACATAACTCTGCTACAATAGAACAAATGTATGAACGTGCGAATATGCAGATTC
ATTAGGTACAGTTATTGTTATGGTCGATTTGTATCGGTTATACTGCAATTCAAA
CGATGGCTATTTGGGCACGAAAAGCCAAATGATTTTACATTTACATCGTGCCGG
AAATTCTACATATGCTCGCCAAAAAAATCATGGTATTAATTTCCGTGTAATTTGT

AAATGGATGCGATGTCAGGGGTGGATCATATCCATGCTGGTACTGTTGTAGGTA
AATTAGAAGGTGATCCTAATGGTAAGAGGTTCTATAACACTTTACTTTTAACAGA
ATTAAATAACTTAGCGAAGGGCTATTCTTTGATATGGATTGGGCATCTTTAAGA
AAATGTGTTCAGTAGCATCTGGAGGAATTCATTGTGGACAGATGCATCACTTTT
ACTATTTATGATGATTAGTATTACAGTTTGGTGGTGGTACTATTGGTCACCCAG
ATGGTATCCAATCTGGTGCAACAGCAAACCGTGTTGCCTTAGAAGCTATAGTTT
TAGCTCGAAATGAAGGTCGGATTATGTAGAAGAAGGTCCAGAAATTTTACGTAA
TGCTGCAACAACATGTGGTCCACTAAAAGCAGCTTTAGATTTATGGAAAGATAT
TACATTGAATATACTTCAACAGATACACCTGACTTCGTTGAGGTTCCACAACAGG
TAATTAA
>MG459429.1_Sargassum_confusum
ATGTCTGAGAACGTACAGGAAAGAACTCGAATAAAAAGTGAGCGTTATGAAAAT
ACCATATGCTTGGGAGATAGTTAAACTGATATTCTAGCTCATTTCGTTAACTCTC
AACCAGGTGTAGATCCTGTAGAAGCCGGCTGCTGTTGCGGTGATCTCTACAGCA
CTGGACAGTAGTATGGACAGATTATGACGGCTTGTGATATCTACCAGCGAAAGC
ATATCGTGTCGATCCTGTCCGAAGTAATATTTCTCATATATTGCAATGTGATATT
GGAATCTTTGCAAACAACTGCATCTATCTCGGTAACTTGGTTTTAAAGCTTTAAA
GCATTACGTTTAGAAGATATGAGAATTCCTACGCTTACCTTAAAACGTTCCAAGG
TCCCGCTACAGGTGTCGTCGTGGAAAGAGAAAGATTAGATAAATTTGGACGTCC
TTTATTAGGGCTACTGTTAAACCTAAATTAGGTCTTTCAGGTAAAAATTATGGTC
GTGTTGTTTACGAAGGTTTAACAGGTGGTCTTGATTTCCTTAAAGATGATGAAA
ACATTAATTCGCAACCATTTATGAGATGGAAAGAGCGTTTCTTATATTGTATGG
AAGGTGTTAACCGTTCAGCAGCAGCTACAGGTGAAGTTAAGGGTTCTTATCTTA
ATGTTACGCAGCAACAATGGAAAATATGTATGAACGTGCGAATACTCTCATGCTA
TTGGTAGTGTTATTTGTATGATTGATTTGTGTTGGATATACAGCAATCCAAACT
ATGGCAATTTGGGCTCGAAAAGCGAGATGATTTTACATTTACATCGTGCGGGTA
ACTCTACTTATGCACGTCAAAAAAATCATGGTATTAACTTTAGGGTTATTTGTAA
ATGGATGCGATGTCTGGTGTAGATCATATTCATGCAGGTACAGTTGTAGGGAAA
CTAGAAGGAGATCCTGATGGTTAGAGGTTCTATAATACGCTATTATTAACTGAG
TTAAATAATTTAGCGAAGGGCTATTTTTCGATATGGATTGGGCATCGCTTCGAA
AATGTGTTCTGTAGCGTCAGGTGGTATTCATTGTGGTCAAATGCATCACTTTTA
TTATTTACGATGATTTGTTCTACAATTTGGAGGTGGTACAATTGGTCACCCTGA
TGGTATACAAGCCGGTGCGACAGCAAATCGTGTTGCTTTAGAAGCAATGGTTCT
AGCTAGAAATGAGGGTCGGATTATGTTGGTGAAGGACCGGAAATTTTACGTACC
GCAGCAAGTACTTGTGGACCGTTAAAAGCAGCTTTAGATCTATGGAAAGATATT
ACTTTGAATATACTTCAACAGATACACCTGATTTTGTTGAAGTAGCACTGAAAAT

CCTTAG
>NC_012898.1_Aureococcus_anophagefferens
ATGTCTCAATCTGTATCTGAACGTACAAGAATCAAAAGTGAACGTTACGAAAATC
CCATACGCCTGGGAAGCAATTAAACTGACCTTTTAGCGTATTCCGTTCACACACA
ACCAGGTGTTGACCCAGTTGAAGCCTGCTGCTGTTGCGGTGATCTCAACTGCGC
TGGACTGTTGTATGGACTGACTATAACTGCTTGTGATGTATACCTGCTAAAGCA
TTCCGTGTAGATCCAGTCCGGCGTAATTCTTCGCGTACATCGCAAGGTGACATT
GGAATCATAGCGAACGACTGCTTCTATTTTGGTAACTTGGTTTCAAGGCCTTAA
AGCGTTACGTTTAGAAGATATGCGTATTCCCACTCTTACTTAAAAACATTCCAAG
GTCCTGCGACTGGGGTTGTTGTTGAGCGTGAGCGTATGGATAAATTCGGTCGT
CCATTATTAGGGCTACTGTTAAGCCTAAGTTAGGTTTATCTGGTAAGAACTACG
GACGTGTAGTATTCGAAGGTTTAAAAGGTGGTTTAGACTTCTTAAAGGATGATG
AGAACATTAACTCACAACCATTCATGCGTTGGCGTGAGCGTTTCATCTACTGTG
TAGAGGGTATCAACCGTGCCGCTGCTGCAACTGGTGAAGTTAAAGGTTCTTACT
TAAACATTACGCTGCAACAATGGAAGAAATGATCATTCGTGCGAGTACGCTAAA
GAGTTAGGTTCTATCATTATCATGATTGACTTGTATTGGTTACACAGCTATCCAA
ACTATGGCTATCTGGTCTCGTGAGAAGATATGCTTTTACATTTACACCGTGCTG
GTAACTCAACTTACGCTCGTCAAAAGAGTCACGGTATTAACTTCCGTGTAATCTG
TAAGTGGATGCGATGGCTGGTGTTGACCACATTCACGCTGGTACTGTTGTAGG
TAAGTTAGAAGGTGATCCAAATGGTTCAAGGTTCTACGATACTTTATTAAAAACT
AAGTTAGCTGATTTACCAAGGGTATCTTCTTCGACATGGACTGGGCTGCTTTAC
GTAAGTGTATGCAGTAGCTTCTGGTGGTATCCACTGTGGTCAAATGCACCATTT
AAACTACTTATGACGACTTATCTTACAATTCGGTGGTGGTACAATTGGTCACCC
TGATGGTATCCAAGCCGGTGCAACTGCTAACCGTGTAGCTTTAGAAGCTATGGT
ATTAGCTCGTAACGAAGGTCGAACTACGTTGAGGAAGGTCCTCAAATCTTACGC
GACACAGCTAAAATGTGTGGTCCTTTACAAACAGCTTTAGACTTATGGAAGGAT
ATTAGTTTAACTACGCGTCTACTGATACAGCTGACTTCGCTGAGACACCACTGC
AAACGTATAA
>NC_012903.1_Aureoumbra_lagunensis
ATGTCTCAATCTGTATCAGAACGTACACGAATCAAGAGCGAACGTTATGAAAATC
CCGTATGCCTGGGAAGTCATTAAACTGATCTTCTTGCATATTCCGTTCACACGC
AACCAGGGGTAGATCCTGTTGAAGCCAGCTGCAGTAGCGGTGATCTCAACAGCT
CTGGACTGTTGTATGGACTGACTATAACTGCTTGTGACGTATACCTGCAAAAGC
TTACCGTGTAGATCCTGTCCGGGGTAATTCTTTGCATATATCGCAAGGTGATAT
TGGAATCGTAGCTAATGACAGCATCTATCTTGGTAACTTGGTTTCAAAGCTTAA
AAGCGTTACGTTTAGAAGATATGCGTATTCCCACTCTTACTTAAAGACTTTCCAA

GGTCCTGCTACTGGTGTGATCGTAGAGCGTGAGCGTTTAGACACATTCGGTCG
TCCACTTTTAGGGCTACAGTAAAACCTAAATTAGGTTTATCTGGTAAGAACTACG
GTCGTGTAGTTTTCGAAGGTTTAAAAGGTGGTTTAGACTTTTTAAAGGATGATG
AAAACATTAACTCGCAACCTTTCATGCGTTGGCGTGAGCGTTTCTTATACTGTA
TCGAAGGTATTAATCGTGCCGTTTCAGTAACAGGTGAGGTTAAGGGTTCTTACT
TAAACATTACGCTGGTACTATGGAAGAAATGTACACACGTGCGAGTACGCTAAG
GAACTTGGTTCAATTATTGTAATGATCGATTTGTATCGGTTACACAGCTATTCAA
AGTATGGCTATTTGGGCTCGTAATAAGATATGGTTTTACACTTACACCGTGCTG
GTAACTCAACATATGCACGTCAAAAGACTCATGGTATTAATTTCCGTGTAATTTG
TAAGTGGATGCGATGGCTGGTGTTGACCATATCCACGCAGGTACTGTTGTAGGT
AAGTTAGAAGGTGACCCAAATGGTTCAAGGTTCTACGATACTTTATTATTAACTC
ACTTAAATGATTTACCAAGGGTATTTTCTTCGATATGGATTGGGCATCTTTACG
TAAGTGTATGCTGTTGCATCGGGTGGTATCCATTGTGGTCAAATGCATCATTTA
AATTACTTATGATGATTTGTATTACAGTTTGGTGGTGGTACAATTGGTCACCCT
GATGGTATCCAAGCTGGTGCTACTGCAAACCGTGTAGCTTTAGAAGCTATGGTA
TTAGCACGTAATGAAGGTCGAACTATGTAGAGGAAGGTCCTCAAATCTTACGTG
ATACTGCTAAGATGTGTGGTCCATTACAAACTGCATTAGATTTATGGAAAGATAT
TAGTTTAACTACGCATCAACTGATACTGCAGATTTCGTTGAGACTGCACAGCTAA
CGTATAA
>NC_027746.1_Triparma_laevis
ATGTCTAACTCTGTATCAGAACGGACTCGAATCAAAAGTGACCGTTACGAAAATT
CCATACGCCTGGGAGCACATAAAACTGATATCCTAGCATATTCCGTTTACACTCA
ACCAGGTGTAGATCCAGTAGAAGCCAGCTGCTGTAGCGGTGATCTCAACAGCTC
TGGACTGTAGTTTGGACAGATTATAACAGCTTGTGAAAGATACCAGCTAAAGCA
TACCGTGTAGATCCAGTCCAAAGTAATACTTTGCTTTTATCGCAATGTGATATC
GAAATCTTACCTAACAACTGCATCTATTTTGGTAACTTGGTTTCAAAGCTTTTCT
GCGTTACGTTTAGAAGATATGCGTATCCCCACTCGTACTTAAAAACTTTCCAAGG
TCCTGCAACTGGTATTATTGTAGAACGTGAACGTTTAGATAAGTACGGTCGTCC
TGTATTAGGGCTACTGTAAAACCTAAATTAGGTCTTTCTGGTAAAAACTACGGT
CGTGTAGTTTATGAAGGTCTTAAAGGTGGTTTAGACTTCTTAAAAGATGATGAA
AACATTAACTCTCAACCATTCATGCGTTGGAGAGAACGTTACTTAAACTGTATGG
AAGGTATCAACCGAGCTGCAGCCGCTACTGGTGAAATTAAAGGTTCTTACTTAA
ACATTACGCTGCAACTCAAGAAGAAGTATACAAACGTGCGAGTATGCTAAAGCTC
TTGGTTCTGTAATTATTATGATCGATTTGTATGGGTTACACAGCAATTCAAACT
GCTGCTATCTGGGCTCGTGATAAGACATGATCTTACATTTACACCGTGCAGGTA
ATTCAACTTACGCTCGTCAAAAAAACCATGGTATTAACTTCCGTGTGATTTGTAA

ATGGATGCGATGTCTGGTGTAGATCATATCCATGCTGGTACTGTAGTAGGTAAA
TTAGAAGGTGATCCTAATGATTAAAGGTTCTACGATGTTTTACGTTTAACGAAT
TTAGATAACTTACCTTTGGTATTTTCTTCGAAATGGATTGGGCAAGTTTACGTA
AATGTATGCTGTAGCTTCAGGTGGTATTCACTGTGGTCAAATGCATCATTTATA
CTACCTAAGATGATTAGTATTACAATTCGGTGGTGGTACAATTGGTCACCCTGA
TGGTATCCAAGCAGGTGCTACAGCTAACCGTGTTGCTTTAGAAGCAATGGTATT
AGCTCGTAACGAAGGTGCGACTACTTTAACGATGGTCCTCAAATCTTACGTGAT
GCAGCTAAAACATGTGGTCCTTTACAAACAGCTTTAGATTTATGGAAAGATATTA
GTTTAACTATACTTCTACAGATACAGCTGATTTCGCTGAAACACCACTGCTAACG
TATAA
>NC_043890.1_Rhizochromulina_marina
ATGTCTCAATCTGTATCAGAACGTACACGAATTAAAACTGAGCGTTACGAAAATC
CCATATGCCTGGGAAACCTTAAAACAGATGTTCTTGCATATTCCGTTTACACTCA
ACCAGGTGTAGATCCAGTGGAAGCCAGCAGCTGTAGCGGTGATCTCTACTGCAC
TGGACTGTTGTATGGACTGACCTTAACAGCATGTGATGTTTATCTGCGAAAGCT
TACCGTGTAGATCCAGTCCAATGTAATTCTTTGGTTACATCGCAATGTGATATC
GAAATCATTGCTAACTACAGCTTCTATCTTGGTAACTTGGTTTCAAAGCATTAAA
GCGCTTCGTTTAGAAGATATGCGTATTCCTTTGCATACTTAAAAACTTTCCAAG
GTCCAGCAACAGGTGTTGTTGTAGAACGTGAGCGTTTAAATAACTTCGGTCGTC
CAATTCTTGGGCTACTGTAAAACCTAAGCTAGGTCTTTCTGGCCGTAACTATGG
TCGTGTAGTATACGAAGGTCTAAAAGGTGGTCTAGACTTCCTAAAAGATGACGA
GAACATTAACTCTCAACCATTCATGCGCTGGCGTGAGCGTTTCCTTTATGTTCA
AGAAGGTATTACGCGTGCTTCAGCTTCTACGGGTGAAGTTAAAGGTTCGTACAT
GAACATGACGCCTCAACTATGGAAGATGCTTATGAGCGTGGGAATACGCAAAAG
AACTTGGTACAATCATTGTGATGGTTGACCTGTATGGGTTACACAGCGATTCAA
TCAGCAGCTATTTGGGCACGTAAAAAGATATGATTCTTCATTTACACCGTGCAG
GTAACTCTACGTACGCACGTCAAAAAAATCACGGTATTAACTTCCGTGTAATTTG
TAAATGGATGCGATGGCTGGTGTTGACCACATCCACGCAGGTACTGTAGTAGGT
AAGTTAGAGGGAGACCCAAATCGTTAAAGGTTCTACAACACTCTACTAAACATTA
CGACTGATAACCTACCCAAGGTTTATTCTTTGCTCAAGATTGGGCGTCACTACG
TAAATGTCTACTGTAGCTTCTGGTGGTATTCACTGTGGTCAAATGCACCTCTTA
AACTACCTATGACGACTAGTTCTTCAATTTGGTGGTGGTACAATTGGACACCCA
GATGGTATTCAAGCTGGTGCAACAGCTAACCGTGTAGCGCTTGAAGCTATGGTT
CTAGCACGTAACGAAGGTCGGACTACGTTGCTGAAGGTCCAGAGATTCTTCAAG
AAGCTGCAAAAATGTGTGGTCCTCTACAAACAGCTCTAGATCTATGGAAAGGTA
TTTCATTAACTACAGTTCTACGGATACAGCTGACTTTGCTGAGACTCCACTGCAA

ACGTGTAG
>NC_043929.1_Dictyocha_speculum_CCMP1381
ATGTCTCAATCTGTATCAGAACGTACACGAATTAAAAGCGACCGTTATGAAAATC
CCTTATGCCTGGGAGCACGTAAAACTGACCTACTTGCTTATTCCGTTCACACACA
ACAAGGTGTAGATCCGGTTGAAGCCAGCAGCCGTAGCGGTGATCTCAACCGCTC
TGGACTGTTGTATGGACAGATCTTAACAGCATGTGATGTTTATCTGCTAAAGCT
TATCGTGTAGATCCTGTCCAACGAAATATTTTGGATATGTTGCAAGTTGATTTC
GAAATCATTGCCAATGACAGCGTCTATTTTGGTAACTTGGTTTCAAAGCGTTAA
AGCTCTTCGTCTAGAAGATATGCGTATCCCTTTGCTTACCTAAAAACATTCCAAG
GACCTGCAACTGGTGTTGTTGTAGAGCGTGAGCGTATGGATAAATTTGGTCGTC
CTTTATTAGGGCGACAGTTAAGCCTAAATTAGGTCTTTCTGGTAAAAACTATGG
TCGTGTAGTTTTCGAGGGTCTTAAAGGTGGTCTTGATTTCCTTAAAGATGATGA
GAATATTAACTCACAAGCGTTCATGCGTTGGAGAGAACGTTTCCTTTACTGTCA
AGAAGGTATTCAACGTGCCGCAGCAGCTACTGGTGAAGTTAAAGGTAGCTACCT
TAATGTTACGCTGGTACTATGGAAGCCGTTTACGAACGTGCGAATATGCTAAGG
AGCTTGGTAGTATTGTTATTATGATTGACCTGTATGGGTTATACAGCAATTCAA
TCAATTGCTTATTGGGCTCGTAATAAGATATGATTCTTCACTTACACCGTGCAG
GTAACTCTACATATGCACGTCAAAAGAACCATGGAATTAACTTCCGTGTAATTTG
TAAGTGGATGCGATGGCTGGTGTTGATCATATCCATGCGGGTACAGTTGTTGG
TAAATTAGAAGGTGATCCTAATGGTTAAAGGTTCTATCATACATTACTTGATGTT
AAGACTGATAACCTTCCCAAGGTTTATTCTTCGCCCAAGATTGGGCTTCTCTAC
GTAAATGTCTGCAGTAGCCTCTGGTGGTATTCATTGTGGTCAAATGCACCATTT
TAACTATTTATGATGATTAGTTCTTCAGTTTGGTGGTGGTACAATCGGACATCC
TGATGGTATTCAAGCCGGTGCTACTGCAAACCGAGTAGCTTTAGAGACTATGGT
TCTTGCTCGTAACGAAGGTCGGACTACGTTGCAGAAGGTCCTGAAATCTTACAA
GATGCAGCTAAAATGTGTGGTCCTCTACAGACAGCTTTAGATCTTTGGAAAGGT
ATTAGTTTAATTATGCATCAACTGATACTGCTGATTTCTCTGAGATAGCACAGCT
AACGCATAA
>NC_044407.1_Florenciella_parvula_CCMP2471
ATGTCTAACTCTGTATCAGAACGTACACGAATTAAAAGCGAACGTTACGAAAATC
CCTTACGCCTGGGAACTCATAAAACTGATGTTCTAGCTCATTCCGTTTTCACACA
ACCAGGGGTTGACCCTGTAGAAGCCTGCAGCAGTAGCGGTGATCTCAACTGCAC
TGGACTGTTGTATGGACTGACCATAACAGCTTGTGATATTTACCTGCGAAAGCA
TACCGTGTTGACCCTGTCCAACGCAATTCTTTGGTTACATCGCAATGTGATTTC
GGAATCATTGCAAACTACTGCATCAATCTTGGTAACTTGGTTTCAAGGCTTTAA
GGCTCTTCGTCTAGAAGATATGCGTATCCCTTTGCTTACTTAAAGACTTTCCAA

GGTCCAGCTACAGGTGTTGTAGTAGAGCGTGAGCGTATGGATAAGTTTGGTCG
TCCTCTTCTTGGGCTACTGTTAAGCCTAAGCTAGGTCTTTCAGGTAAGAACTAC
GGTCGTGTAGTATATGAAGGTCTTAAGGGTGGTCTTGATTTCCTTAAAGATGAT
GAGAACATTAACTCTCAAGCATTCATGCGTTGGCGTGAGCGTTTCCTTTACTGT
CAAGAAGGTATCCAACGTGCTGCTGCAGCTACTGGTGAAGTTAAAGGTTCATAC
CTTAACGTTACGCTGGTGATATGGAAGCAGTTTACGAGCGTTGAACTACGCTAA
AGAACTTGGTACTATCGTTGTAATGATTGACCTGTATGGGTTACACTGCAATTC
AATCAGCAGCTAAGTGGGCTCGTAAGAAGATATGATTCTTCACCTTCACCGTGC
TGGTAACTCTACATATGCACGTCAAAAGAACCACGGTATTAACTTCCGTGTAATT
TGTAAGTGGATGCGATGTCTGGTGTTGACCATATTCACGCTGGTACAATCGTTG
GTAAGCTAGAAGGTGACCCTAATGGTTCAAGGTTCTACGAGACACTACTATGTG
TTAAGTCAGATAACCTACCAAAGGTCTATTCTTCGCTCAAGATTGGGCTTCTCTA
CGTAAGTGTTTACAGTAGCTTCTGGTGGTATTCACTGTGGTCAAATGCACCACT
TTAACTACCTTTGATGACTAGTACTACAATTCGGTGGTGGTACAATTGGTCACC
CTGATGGTATCCAAGCAGGTGCGACTGCTAACCGTGTTGCTCTAGAAGCAATGG
TTATGGCTCGTAACGAAGGTCGGATTACGTAGCTGAAGGTCCTGAGATTCTTCA
AGACGCTGCTAAGATGTGTGGTCCTCTTCAAACAGCTCTAGATCTATGGAAAGG
TATTTCATTAACTACACTTCTACAGATACAGCTGACTTTGCGGAAACTCCACTGC
AAACGTATAA
>NC_044408.1_Pseudopedinella_elastica_CCMP716
ATGTCTCAAAACGTATCAGAACGTACACGAATTAAAAGCGAGCGTTACGAAAATT
CCATATGCCTGGGAACACATAAAACTGATGTTCTTGCTCATTCCGTTTACACTCA
ACCAGGTGTGGATCCAGTAGAAGCCTGCAGCTGTAGCGGTGATCTCAACTGCAC
TGGACTGTTGTATGGACTGACCTTAACTGCTTGTGATATCTACCTGCGAAAGCT
TACCGTGTAGACCCTGTCCAACGTAATTCTTTGGTTACATCGCAATGCGAGATC
GAAATCATAGCTAACTACTGCTTCTATTTCGGTAACTTGGTTTCAAAGCTTAAAA
GCTCTTCGTTTAGAAGATATGCGTATCCCTATGCTTACCTTAAGACATTCCAAG
GTCCAGCTACAGGTGTAGTTGTAGAGCGTGAGCGTATGAACTCATTCGGTCGTC
CACTACTTGGGCAACTGTTAAGCCTAAATTAGGTCTTTCTGGTCGTAACTACGG
TCGTGTAGTATATGAAGGTCTTAAAGGTGGTCTAGACTTCCTTAAAGATGATGA
GAATATTAACTCTCAACCATTCATGCGTTGGCGTGAGCGTTTCCTATACGTACA
AGAAGGTATCACTCGTGCATCAGCTGCTACAGGTGAGGTTAAAGGTTCTTACCT
TAACATTACGCTGGTACTATGGAAGATACTTATGAGCGTGGGAGTACGCAAAAG
AGATTGGTACTATCGTTGTAATGGTTGACCTGTATGGGTTACACAGCATTACAA
TCATCAGCTATCTGGGCTCGTAAGAAGATATGATTCTTCACCTTCACCGTGCTG
GTAACTCTACATATGCACGTCAGAAAAACCACGGTATCAACTTCCGTGTAATCTG

TAAGTGGATGCGATGGCAGGTGTTGACCATATTCACGCAGGTACAGTTGTAGGT
AAGTTAGAGGGTGATCCTAATGGTTAAAGGTTCTACAACACTTTATTAAACGTTA
AGACAGATAACCTTCCCAAGGTTTATTCTTCGCTCAAGATTGGGCTTCACTACG
TAAGTGTATGCAGTAGCATCAGGTGGTATCCACTGTGGTCAGTTACACCACTTT
AACTACCTATGACGACTAGTTATGCAATTCGGTGGTGGTACAATTGGTCACCCA
GATGGTATCCAAGCAGGTGCAACTGCAAACCGTGTAGCTCTTGAAGCTATGGTT
CTTGCACGTAATGAGGGTCGGACTATTTATCAGAAGGTCCACAAATCTTAATGG
ATGCTGCTAGAATGTGTGGTCCATTACAAACAGCTCTAGATCTATGGAAAGGTA
TTTCATTAACTACACTTCTACAGATACAGCAGACTTTGCAGAAACTCCACTGCAA
ACCGATAA
>HQ710601.1_Pseudopedinella_sp._CCMP1476

CTTGCTCATTCCGTTTACACTCAACCAGGTGTAGACCCTGTAGAAGCCTGCTGC
TGTAGCGGTGATCTCAACTGCACTGGACTGTTGTATGGACTGACCTTAACAGCT
TGTGATATCTACCTGCTAAAGCATACCGTGTAGATCCAGTCCAGACCAATTCTTT
GGTTATATTGCAATTCGAAGAAGGTTCTTTAGCTAACAACGGCATCAATTTTGG
TAACTTGGTTTCAAAGCTTTAAAGCACTTCGTCTAGAAGATATGCGTATTCCTTT
GCATACCTTAAAACATTCCAAGGACCTGCAACTGGTGTAATTGTAGAACGTGAG
CGTTTAAACTGCTTTGGTCGTCCATTATTAGGGCTACAGTTAAGCCAAAATTAG
GTCTTTCTGGTCGTAACTACGGTCGTGTAGTATTCGAAGGTCTTAAAGGTGGTC
TTGATTTCTTAAAGGATGATGAGAATATTAACTCTCAACCATTCATGCGTTGGC
GTGAGCGTTTCCTTTACGTTCAAGAAGGTATTACACGTGCTTCAGCAGCTACTG
GTGAAGTAAAAGGTTCTTACCTAAACATCACGCTGGTACTATGGAAGATGTATA
TGAGCGTGCGAGTACGCTAAAGAAATTGGTACTGTTATTGTAATGATCGATTTG
TATGGGTTACACAGCTATTCAATCATCTGCAATTTGGGCACGTAAGAAGATATG
ATTCTTCACCTTCACCGTGCTGGTAACTCAACTTATGCACGTCAAAAAAATCATG
GTATTAACTTCCGTGTAATTTGTAAATGGATGCGATGGCTGGTGTTGACCATAT
TCACGCAGGTACTGTAGTAGGTAAATTAGAAGGTGATCCTAATGGTTAAAGGTT
CTACCACACTTTATTAGATGTTACATCTGATAACTTACCCAAGGTATTTTCTTCG
CTCAAGATTGGGCTTCATTACGTAAATGTTTACAGTAGCTTCTGGTGGTATTCA
CTGTGGTCAATTACACCAACTTCATTACCTGTGATGATTAGTTCTACAATTTGGT
GGTGGTACAATTGGTCACCCAGATGGTATCCAAGCTGGTGCTACTGCAAACCGT
GTTGCTCTTGAAGCAATGATTATGGCACGTAACGAAGGTCGGACTATGTTCAAG
AAGGTCCAGCAATTCTTCAAGCAGCAGCTAGAATGTGTGGTCCTCTACAGACAG
CATTAGATCTATGGAAAGGTATTTCATTAACTACACTTCTACAGATACACCTGAT
TTCGCAGTAACTCCACTGCAAACGTATAA

>AB510943.1_Pseudopedinella_pyriformis_strain_NIES1381

TGACCTTAACTGCTTGTGATATCTACCTGCAAAAGCATACCGTGTAGACCCTGT
CCAACGTAATACTTTGGTTACATCGCAATGTGAGATCGAAATCATAGCTAACAAC
AGCTTCTATTTCGGTAACTTGGTTTCAAAGCGTTAAAGCTCTTCGTTTAGAAGA
TATGCGTATTCCTATGCTTACCTTAAAACATTCCAAGGTCCAGCTACAGGTATCG
TTGTAGAACGTGAGCGTCTAAATAACTTTGGTCGTCCACTACTTGGGCAACTGT
TAAACCGAAATTAGGTCTTTCAGGTCGTAACTATGGTCGTGTAGTATATGAAGG
TCTTAAAGGTGGTCTTGACTTCCTTAAAGATGACGAGAACATCAACTCTCAACCA
TTCATGCGTTGGCGTGAACGTTTCCTTTACGTACAAGAAGGTATCATGCGTGCA
TCAGCTGCTACAGGTGAAGTTAAAGGTTCATACCTTAACATTACGCTGGTACTA
TGGAAGATACTTATGAGCGTGGGAGTATGCAAAAGAGATTGGTACGATTATTGT
GATGGTTGACCTGTATGGGTTACACAGCACTTCAATCAGCTGCTATTTGGGCTC
GTAAGAAGATATGATTCTTCACCTTCACCGTGCAGGTAACTCTACATATGCACGT
CAAAAAAACCACGGTATCAATTT---

>AB081639.1_Pedinella_sp.

ACACTTAAAACTGATGTTCTTGCTCATTCCGTTTACACTCAACCAGGTGTAGACC
CTGTAGAAGCCTGCTGCTGTAGCGGTGATCTCAACTGCACTGGACTGTTGTATG
GACTGACCTTAACAGCTTGTGATATCTACCTGCTAAAGCATACCGTGTAGATCCA
GTCCATCGTAATTCTTTGGTTATATTGCAAATCGATATCGAAATCTTAGCTAACA
ACGGCATCAATTTTGGTAACTTGGTTTCAAAGCTTTAAAGCACTTCGTCTAGAA
GATATGCGTATTCCTTTGCATATCTTAAAACATTCCAAGGACCTGCAACTGGTG
TAGTTGTAGAACGTGAGCGTTTAAACTGCTTTGGTCGTCCATTATTAGGGCTAC
AGTTAAGCCAAAATTAGGTCTTTCTGGTCGTAACTACGGTCGTGTGGTATTCGA
AGGTCTTAAAGGTGGTCTTGATTTCTTAAAGGATGATGAGAATATTAACTCTCA
ACCATTCATGCGTTGGCGTGAGCGTTTCCTTTACGTTCAAGAAGGTATTACACG
TGCTTCAGCAGCTACTGGTGAAGTAAAAGGTTCTTACCTAAACATCACGCGGGT
ACTATGGAAGATGTATATGAGCGTGCGAATACGCTAAAGATCTTGGTACTGTTA
TTGTAATGATCGATTTGTATGGGTTACACAGCTATTCAATCATCTGCAGTTTGG

GCACGTAAGAAGATATGATTCTTCACCTTCACCGTGCTGGTAACTCAACTTATG
CACGTCAAAAAAATCATGGTATTAACTTCCGTGTAATTTGTAAATGGATGCGATG
GCTGGTGTTGACCATATTCACGCAGGTACTGTAGTAGGTAAATTAGAAGGTGAT
CCTAATGGTTAAAGGTTCTACCATACTTTACTAGATGTTACATCTGATAACTTAC
CCAAGGTATTTTCTTCGCTCAAGATTGGGCTTCATTACGTAAGTGTTTACAGTA
GCTTCTGGTGGTATTCACTGTGGTCAATTACACCACTTTCATTACCTTTGATGA
TTAGTTTTACAATTTGGTGGTGGTACAATTGGTCATCCAGATGGTATCCAAGCT
GGTGCTACTGCAAACCGTGTTGCTCTTGAAGCAATGATTATGGCACGTAACGAA
GGTCGGACTATGTTCAAGAAGGTCCAGCAATTCTTCAAGCAGCAGCTAGAATGT
GTGGTCCTCTACAGACAGCATTAGATCTATGGAAAGGTATTTCATTAACTACACT
TCTACAGATACACCTGATTTCGCAGTAACTCCACTGCAAACGTA---
