# Supplementary material for: Highly Reduced Plastid Genomes of the Non-photosynthetic Dictyochophyceans Pteridomonas spp. (Ochrophyta, SAR) Are Retained for tRNA-Glu-Based Organellar Heme Biosynthesis
Source: Front Plant Sci. 2020 Nov 27;11:602455. doi: 10.3389/fpls.2020.602455 (PMC7728698; doi:10.3389/fpls.2020.602455)
Supplement: Supplementary file 2 [file Data_Sheet_2.PDF]

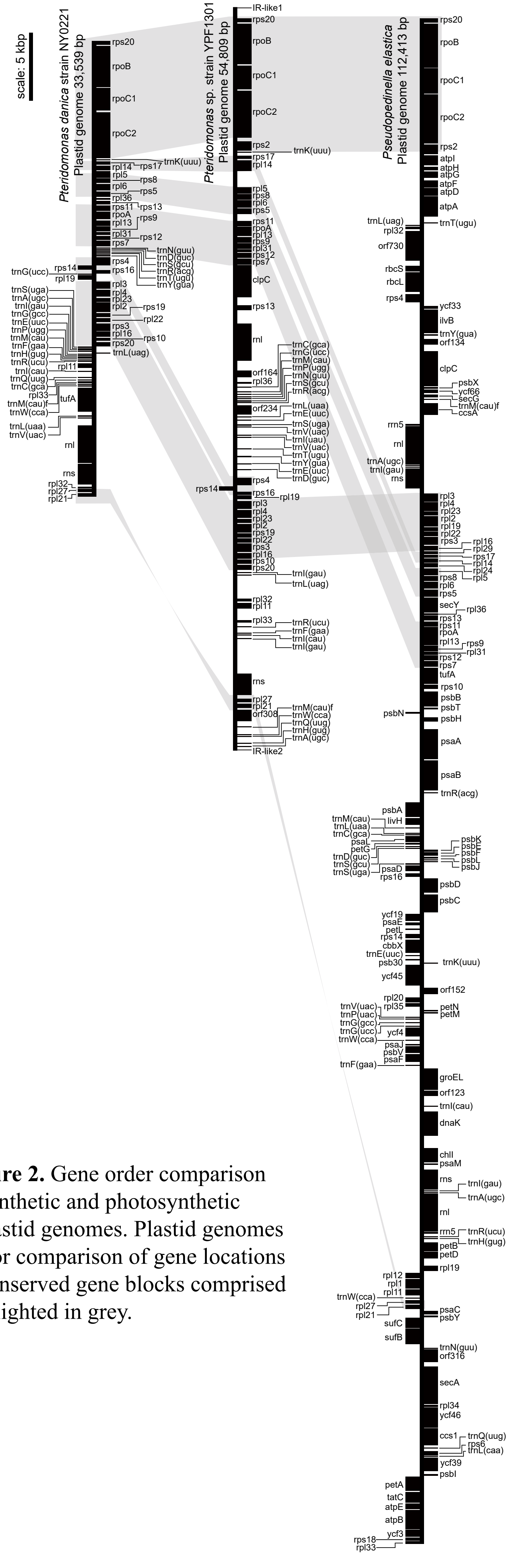

Supplementary Figure 2. Gene order comparison between non-photosynthetic and photosynthetic dictyochophycean plastid genomes. Plastid genomes are shown as linear for comparison of gene locations (see also Fig. S1). Conserved gene blocks comprised of > 2 genes are highlighted in grey.
